# Unidirectional gene pairs in archaea and bacteria require overlaps or very short intergenic distances for translational coupling via termination-reinitiation and often encode subunits of heteromeric complexes

**DOI:** 10.3389/fmicb.2023.1291523

**Published:** 2023-11-09

**Authors:** Madeleine Huber, Nico Vogel, Andreas Borst, Friedhelm Pfeiffer, Svetlana Karamycheva, Yuri I. Wolf, Eugene V. Koonin, Jörg Soppa

**Affiliations:** ^1^Institute for Molecular Biosciences, Biocentre, Goethe-University, Frankfurt, Germany; ^2^Computational Biology Group, Max-Planck-Institute of Biochemistry, Martinsried, Germany; ^3^National Center for Biotechnology Information, National Library of Medicine, National Institutes of Health, Bethesda, MD, United States

**Keywords:** *Haloferax volcanii*, *Escherichia coli*, translational coupling, termination-reinitiation, overlapping gene pairs, cotranslational folding, heteromeric complexes, frame sensitivity

## Abstract

Genomes of bacteria and archaea contain a much larger fraction of unidirectional (serial) gene pairs than convergent or divergent gene pairs. Many of the unidirectional gene pairs have short overlaps of −4 nt and −1 nt. As shown previously, translation of the genes in overlapping unidirectional gene pairs is tightly coupled. Two alternative models for the fate of the post-termination ribosome predict either that overlaps or very short intergenic distances are essential for translational coupling or that the undissociated post-termination ribosome can scan through long intergenic regions, up to hundreds of nucleotides. We aimed to experimentally resolve the contradiction between the two models by analyzing three native gene pairs from the model archaeon *Haloferax volcanii* and three native pairs from *Escherichia coli*. A two reporter gene system was used to quantify the reinitiation frequency, and several stop codons in the upstream gene were introduced to increase the intergenic distances. For all six gene pairs from two species, an extremely strong dependence of the reinitiation efficiency on the intergenic distance was unequivocally demonstrated, such that even short intergenic distances of about 20 nt almost completely abolished translational coupling. Bioinformatic analysis of the intergenic distances in all unidirectional gene pairs in the genomes of *H. volcanii* and *E. coli* and in 1,695 prokaryotic species representative of 49 phyla showed that intergenic distances of −4 nt or −1 nt (= short gene overlaps of 4 nt or 1 nt) were by far most common in all these groups of archaea and bacteria. A small set of genes in *E. coli*, but not in *H. volcanii*, had intergenic distances of around +10 nt. Our experimental and bioinformatic analyses clearly show that translational coupling requires short gene overlaps, whereas scanning of intergenic regions by the post-termination ribosome occurs rarely, if at all. Short overlaps are enriched among genes that encode subunits of heteromeric complexes, and co-translational complex formation requiring precise subunit stoichiometry likely confers an evolutionary advantage that drove the formation and conservation of overlapping gene pairs during evolution.

## Introduction

Neighboring genes in genomes are transcribed either in the same direction and are accordingly unidirectional (→ →), or in the opposite direction and are either convergent (→ ←) or divergent (← →). Genes can overlap in all three configurations. Overlapping genes have been first observed in viruses, but subsequently were found to exist in all three domains of life (Normark et al., 1983; Wright et al., 2022). In archaea and bacteria (collectively, prokaryotes), the number of overlapping gene pairs strongly correlates with the genome size, and about 27% of all protein-coding genes are involved in overlaps (Johnson and Chisholm, 2004; Huvet and Stumpf, 2014). In prokaryotes, in contrast to eukaryotes, 84% of the overlapping gene pairs are unidirectional, whereas convergent and divergent overlaps are comparatively rare (Fukuda et al., 2003; Johnson and Chisholm, 2004). Most gene overlaps in prokaryotes are short, and a comparative genomics analysis indicated that all of the annotated 715 unidirectional gene overlaps longer than 60 nt were due to misannotations of gene starts or ends, out of 38,563 unidirectional gene pairs of 338 microbial genomes that were included in the study (Pallejà et al., 2008). Initially, it was thought that the evolutionary advantage of gene overlaps was genome compaction. This indeed might be the case for viruses where the limited space within the virion, low replication fidelity and selection for rapid replication severely constrain the genome size (Johnson and Chisholm, 2004). However, in prokaryotes, the constraints on the genome size are far more relaxed, and moreover, given that most of the overlaps are short and could only minimally streamline the genome, compaction is unlikely to be the primary factor driving the evolution of gene overlaps (Soppa, 2014). Gene overlaps are not strongly conserved in evolution, and therefore, might in part evolve via genetic drift (Fukuda et al., 2003). However, a widely considered potential advantage of unidirectional gene overlaps is translational coupling of the two overlapping genes.

Translational coupling occurs when, in a pair of adjacent genes, translation of a downstream gene depends on translation of the upstream gene. Evidently, translational coupling applies only to unidirectional genes and requires that both genes were situated on the same polycistronic mRNA. Polycistronic transcripts of operons are common in prokaryotes, in contrast to eukaryotes. Already nearly 40 years ago it has been demonstrated that translation of the downstream gene *trpA* is coupled to the translation of the upstream gene *trpB* in *E. coli* tryptophan operon (Aksoy et al., 1984), and since then, translational coupling has been reported for various additional gene pairs in *E. coli* and other bacteria (Pallejà et al., 2009).

Two distinct molecular mechanisms for translational coupling have been proposed:

1. The translation initiation region (TIR) is involved in long-range base pairing or pseudoknot formation with sequences within the open reading frame (ORF) of the upstream gene resulting in inhibition of translation initiation of the downstream gene. When the upstream gene is translated, the ribosome unwinds the inhibitory structure and frees the TIR of the downstream gene, so that other ribosomes can initiate translation (Petersen, 1989; Lesage et al., 1992; Rex et al., 1994; Chiaruttini et al., 1996). Hereafter we refer to this mechanism as “Upstream gene Translation-dependent *de Novo* Initiation” (UTNI). Because different ribosomes initiate translation at the two genes, the efficiencies of the two respective TIRs determine the amounts of proteins that are produced, and translation of the downstream gene can outperform translation of the upstream gene. For example, coupled translation of the *rplJL* gene pair results in a fivefold excess of RplL over RplJ (Lesage et al., 1992).
2. A mechanistically different molecular mechanism of translational coupling is known as “termination-reinitiation” (TeRe). In this case, the same ribosome (or at least the small subunit) that terminates translation of the upstream gene reinitiates translation of the downstream gene. This mechanism has been well studied in various eukaryotic viruses (reviews see (Powell et al., 2008; Powell, 2010)). In these systems, the 80S ribosome terminates translation of the upstream gene, and the 60S large subunit dissociates from the mRNA, while the 40S small subunit stays bound at so called “termination upstream ribosome binding sites” (TURBS). The TURBS lengths are virus-specific and vary between 40 nt and 90 nt. Then, initiation factors and a new 60S subunit are recruited and translation is reinitiated at the downstream gene. For the first studied example in bacteria, the *trpBA* gene pair, it was reported that an overlap of the *trpB* stop codon and the *trpA* start codon was required for efficient TeRe (Das and Yanofsky, 1989).

However, a contrasting model was put forward for the coat and lysis gene of the *E. coli* phage fr (Adhin and Van Duin, 1990). The two genes overlap by 34 nt, and when additional start codons were introduced near the stop codon of the upstream gene, the start codon closest to the stop was always used. These findings prompted the scanning model of TeRe, which holds that the undissociated 70S ribosome scans the mRNA in both directions after termination (Adhin and Van Duin, 1990). Recently, it has been proposed that the 70S-scanning initiation is a common mechanism of translation in bacteria (Yamamoto et al., 2016). This model was supported by experiments with a bicistronic transcript containing two reporter genes encoding fluorescent proteins, where reinitiation at the downstream gene occurred despite a 73 nt intergenic region between the two genes. In another study, the intergenic distance between the two reporter genes was varied from 150 nt to 850 nt, and it was shown that translational coupling occurred despite long intergenic regions, and the coupling efficiency dropped only about 1.4-fold for every 100 intergenic nucleotides (Levin-Karp et al., 2013).

Thus, sharply contrasting experimental results on translation reinitiation have been presented, some suggesting that the stop and start codons of neighboring genes have to overlap for efficient TeRe to occur, and others that ribosomal scanning over long intergenic regions was possible after translation termination.

We sought to quantify the distance dependence of TeRe using several native gene pairs of the model bacterium *E. coli* and the model haloarchaeon *Haloferax volcanii*. For both species, a two reporter gene system was used that was recently established to characterize several aspects of TeRe (Huber et al., 2019). Intergenic distances of varying length were introduced by site-specific mutagenesis, and the efficiency of TeRe was quantified. In addition, requirements for the minimal length of the upstream gene sequence required for TeRe were determined. These experimental approaches were complemented by bioinformatics analysis of the intergenic distance distribution of 2,661,236 native gene pairs in diverse archaea and bacteria. We further show that gene pairs with very short overlaps are enriched in genes encoding subunits of heteromeric complexes, compared to gene pairs with longer intergenic distances.

## Results

### Distance dependence of the efficiency of TeRe in *Haloferax volcanii*

**Figure 1:**
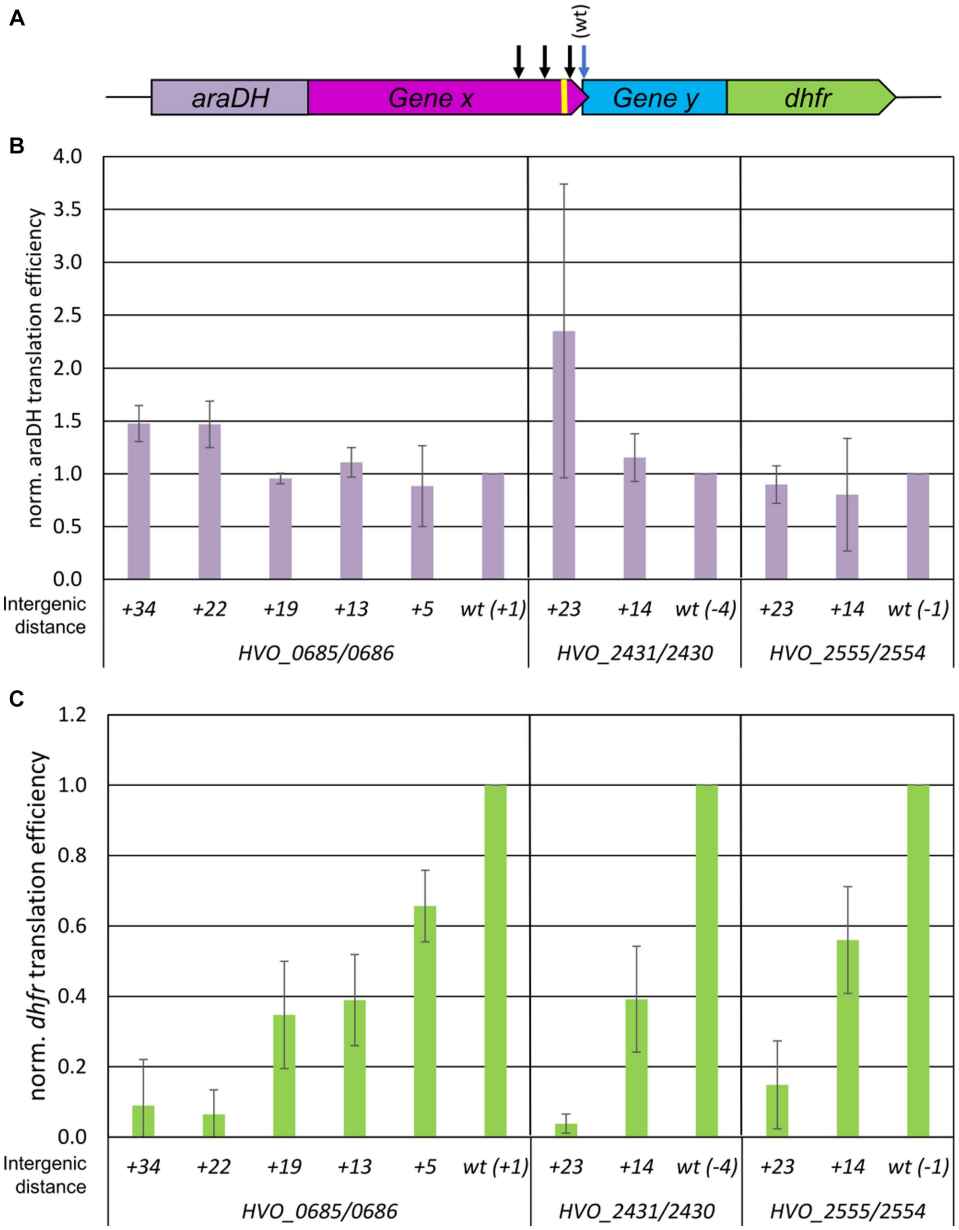
Influence of intergenic distances on the efficiency of TeRe in *H. volcanii*. **(A)** Schematic overview of the two reporter gene construct used to quantify the reinitiation efficiencies. Gene x and y are native gene pairs of *H. volcanii*, *araDH*-gene x and gene y-*dhfr* represent translational fusions. The black arrows schematically indicate that additional stop codons were introduced by mutagenesis in gene x to generate intergenic distances of various sizes. The yellow bar represents the Shine Dalgarno motif. **(B)** Normalized translational efficiencies of the *araDH*-gene x fusions. The identities of the three gene pairs (HVO_numbers) and the lengths of the intergenic regions are shown at the bottom. Protein levels were quantified using a AraHD enzyme assay, transcript levels were quantified by Northern blots, translational efficiencies were calculated as the ratio of both values, and the results were normalized to the value of the wildtype. Three biological replicates were performed, and average values and their standard deviations are shown. **(C)** Normalized translational efficiencies of the y-*dhfr* fusions. The identifies of the three gene pairs (HVO_numbers) and the lengths of the intergenic regions are shown at the bottom. Protein levels were quantified using a DHFR enzyme assay, transcript levels were quantified by Northern blots, translational efficiencies were calculated as the ratio of both values, and the results were normalized to the value of the wildtype. Three biological replicates were performed, and average values and their standard deviations are shown.

Recently, we established a reporter gene system for the analysis of termination-reinitiation in the model haloarchaeon *H. volcanii* (Huber et al., 2019), which was extended to a two reporter gene system in this study. Briefly, translational fusions of the upstream and downstream genes of selected *H. volcanii* gene pairs were constructed as follows: (1) The reporter gene *araDH* was fused to the 3′-region of the selected upstream gene, and, (2) the 5′-part of the downstream gene was fused to the reporter gene *dhfr* (compare Figure 1A for a schematic overview). The AraDH and DHFR protein levels were quantified using the corresponding enzymatic assays, and the *araDH* and *dhfr* transcript levels were quantified using Northern blotting. The translational efficiencies were then calculated as the ratio of both values. The analyses were performed with cultures in the mid-exponential growth phase to ensure steady-state levels of proteins and transcripts. This double reporter gene approach was applied in the current study to quantify the distance dependence of the TeRe efficiency. To establish the approach, the gene pair *HVO_0685/HVO_0686* was chosen, with the native intergenic distance of +1 nt. Premature stop codons were introduced into the upstream gene by site-specific mutagenesis, yielding gene pair versions with intergenic distances of +5 nt, +13 nt, +19 nt, +22 nt and + 34 nt. The normalized translational efficiencies of the *araDH-HVO_0685* translational fusions were nearly identical for all six constructs, as expected (Figure 1B). However, quantification of the translational efficiencies of the *HVO_0686-dhfr* translational fusions revealed a strong dependence of the efficiency of translational coupling on the intergenic distance. Indeed, introducing an intergenic distance of only 13 nt led to a 60% reduction in reinitiation efficiency, and a distance of 22 nt abolished reinitiation completely (Figure 1C).

Two additional gene pairs were selected to determine whether this extreme distance dependence was typical for *H. volcanii*. These two gene pairs had native overlaps of −4 and − 1 nt, respectively. Based on the results obtained for *HVO_0685-HVO_0686*, mutant versions with intergenic distances of 14 nt and 23 nt were generated, and translational efficiency was quantified for all variants (Figures 1B,C). These gene pairs also showed an extremely strong distance dependence of the reinitiation efficiency which dropped to near zero for the intergenic distance of 23 nt. For the intergenic distance of 13/14 nt, the reinitiation efficiency varied for all three analyzed gene pairs and was substantially lower compared to the native gene overlap, varying from 40 to 60%.

Thus, analysis of three gene pairs from *H. volcanii* consistently demonstrated an extreme distance dependence of the efficiency of translational coupling.

### Distance dependence of the efficiency of TeRe in *Escherichia coli*

**Figure 2:**
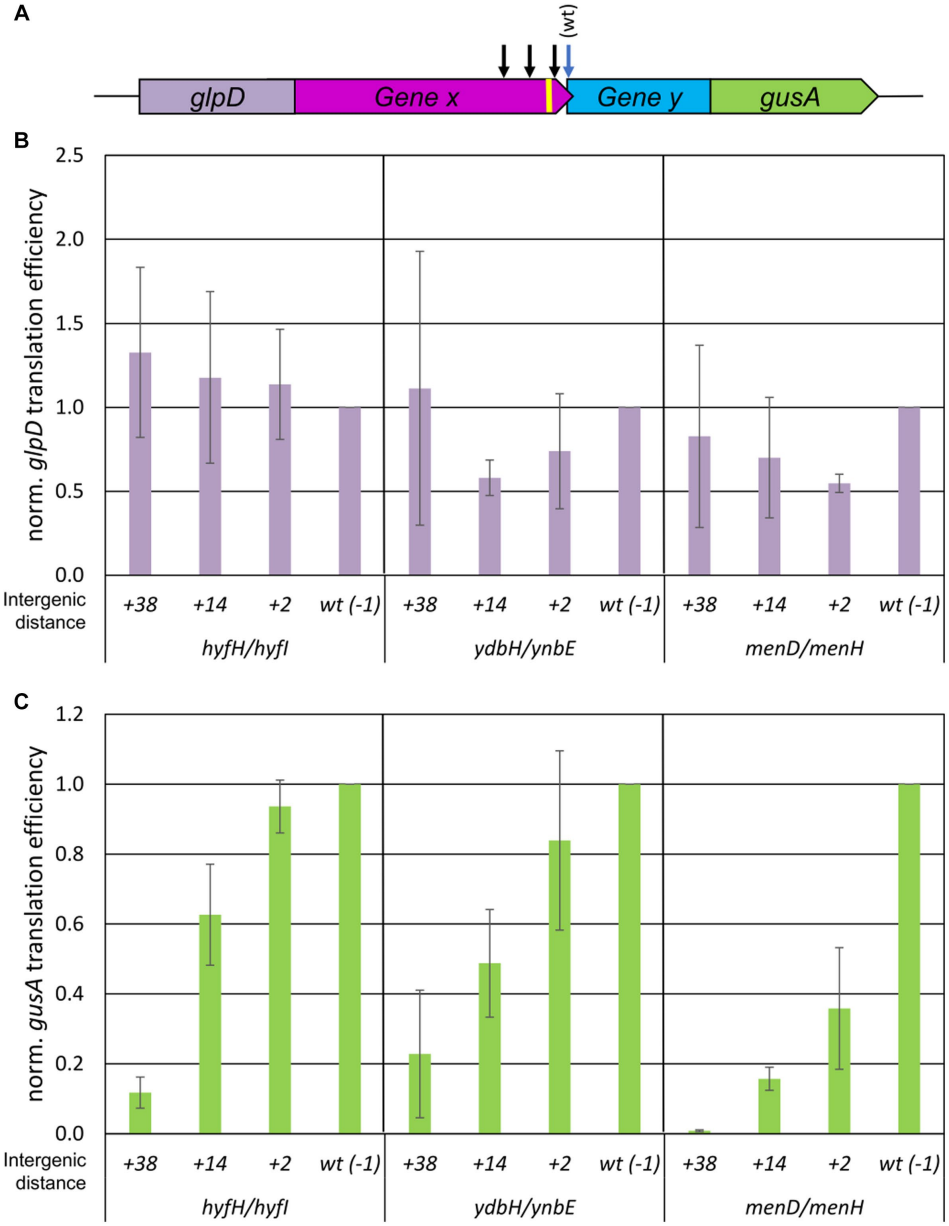
Influence of intergenic distances on the efficiency of TeRe in *E. coli*. **(A)** Schematic overview of the two reporter gene construct used to quantify the reinitiation efficiencies. Gene x and y are native gene pairs of *E. coli*, *glpD*-gene x and gene y-*gusA* represent translational fusions. The black arrows schematically indicate that additional stop codons were introduced by mutagenesis in gene x to generate intergenic distances of various sizes. The yellow bar represents the Shine Dalgarno motif. **(B)** Normalized translational efficiencies of the *glpD*-gene x fusions. The identifies of the three gene pairs and the lengths of the intergenic regions are shown at the bottom. Protein levels were quantified using a GlpD enzyme assay, transcript levels were quantified by Northern blots, translational efficiencies were calculated as the ratio of both values, and the results were normalized to the value of the wildtype. Three biological replicates were performed, and average values and their standard deviations are shown. **(C)** Normalized translational efficiencies of the y-*gusA* fusions. The identifies of the three gene pairs and the lengths of the intergenic regions are shown at the bottom. Protein levels were quantified using a GusA enzyme assay, transcript levels were quantified by Northern blots, translational efficiencies were calculated as the ratio of both values, and the results were normalized to the value of the wildtype. Three biological replicates were performed, and average values and their standard deviations are shown.

The same approach using translational fusions of native gene pairs with two reporter genes was used to analyze the distance dependence of the efficiency of TeRe in *E. coli*. In this case, the reporter genes *glpD* and *gusA* were used, which encode the enzymes glycerol-3-phosphate dehydrogenase and β-glucuronidase, respectively (compare Figure 2A for a schematic overview). Again, protein levels were quantified with enzymatic assays, transcript levels were quantified by Northern blotting, and translational efficiencies were calculated as the ratios of both values. Three native overlapping gene pairs of *E. coli* were chosen, i.e., *hyfH-hyfI* encoding two subunits of a hydrogenase, *ydbH-ynbE* with no annotated function, and *menD-menH* encoding two enzymes involved in menaquinone biosynthesis. As observed previously, transcripts containing long untranslated regions are highly unstable in *E. coli*, in contrast to *H. volcanii* (Huber et al., 2019). Therefore, only the last 99 nt of the upstream genes and the first 30 nt of the downstream genes were used for each gene pair; these constructs were stable even in the absence of translation, ensuring that the ribosomes performed TeRe on the native gene sequences.

For each of the overlapping gene pairs, three mutants with premature stop codons in the upstream gene were generated, resulting in intergenic distances of +2 nt, +14 nt, and + 38 nt, respectively, instead of the native −1 nt overlap. The translational efficiencies of the four upstream gene fusion transcripts of *hyfH* (*glpD-hyfH*) were nearly identical, independent of the intergenic distance (Figure 2B). The variance was somewhat larger for the fusion transcripts of the other two upstream genes (*glpD-ydbH* and *glpD-menD*), but substantial amounts of fusion proteins were generated in all cases. Quantification of the translation efficiencies of the variants of the downstream gene fusions (*hyfI-gusA*, *ynbE-gusA*, and *menH-gusA*) showed that, similar to *H. volcanii*, the reinitiation efficiency in *E. coli* was strongly distance-dependent (Figure 2C). An intergenic distance of 38 nt led to a 80–90% reduction for two gene pairs and completely abolished reinitiation for the *menD-menH* gene pair. The results for the shorter intergenic distances were more variable and gene pair-specific. For example, the shortest intergenic distance of 2 nt resulted in decreases of the reinitiation efficiencies of 5, 20, and 70%, respectively, for the three gene pairs, compared to the native overlap.

### Short 3′-regions of upstream genes are sufficient for TeRe

**Figure 3:**
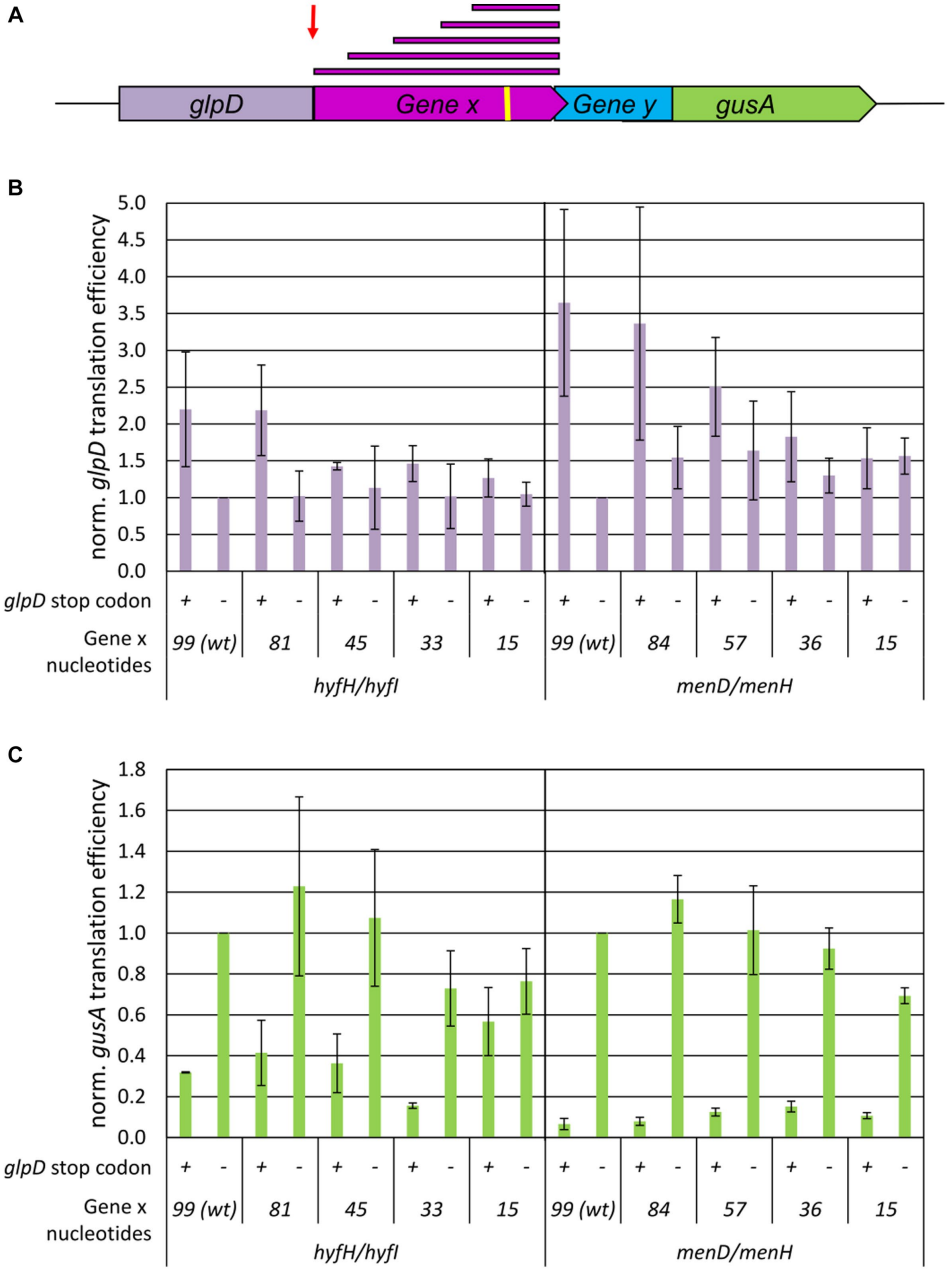
Influence of the upstream gene fragment lengths on the efficiency of TeRe in *E. coli*. **(A)** Schematic overview of the two reporter gene construct used to quantify the reinitiation efficiencies. Gene x and y are native gene pairs of *E. coli*, *glpD*-gene x and gene y-*gusA* represent translational fusions. The red bars on top represent the various fragment lengths of gene x that were fused to *glpD*. The red arrow points to the additional stop codon at the end of the reporter gene *glpD* in the negative controls. The yellow bar represents the Shine Dalgarno motif. **(B)** Normalized translational efficiencies of the *glpD*-gene x fusions. The identifies of the two gene pairs and the lengths of the gene x 3′-fragments are shown at the bottom. Protein levels were quantified using a GlpD enzyme assay, transcript levels were quantified by Northern blots, translational efficiencies were calculated as the ratio of both values, and the results were normalized to the value of the wildtype. Three biological replicates were performed, and average values and their standard deviations are shown. **(C)** Normalized translational efficiencies of the y-*gusA* fusions. The identifies of the two gene pairs and the gene x 3′-fragments are shown at the bottom. The presence or absence of the additional *glpD* stop codon is indicated by “+” and “−“. Protein levels were quantified using a GusA enzyme assay, transcript levels were quantified by Northern blots, translational efficiencies were calculated as the ratio of both values, and the results were normalized to the value of the wildtype. Three biological replicates were performed, and average values and their standard deviations are shown.

We next sought to determine whether the length of the 3′-part of the upstream gene had an effect on termination-reinitiation. The two gene pairs *hyfH-hyfI* and *menD-menH* were chosen, because for a 14 nt long intergenic region the reinitiation efficiency was 60% for the former example, while it was below 20% for the latter example (see above). Translational fusions with *glpD* were generated containing the last 99 nt, 81 nt, 45 nt, 33 nt, and 15 nt of *hyfH* and similar lengths for *menD* (for a schematic overview compare Figure 3A). As negative controls, variants were generated with a premature stop codon at the end of the *glpD* reporter gene, which do not allow translational coupling. The translational efficiencies of all variants were quantified as described above. Most of the translational *glpD-X* fusions showed similar translational efficiencies (Figure 3B). For both gene pairs, the variants containing 99 nt and 81/84 nt, respectively, of the upstream gene were translated more efficiently than the variants containing shorter regions of the upstream gene. Nevertheless, the results showed that the transcript was stable and protein was translated from the fused upstream gene in all cases. Analysis of the *menH-gusA* downstream gene fusions revealed closely similar translational efficiencies, irrespective of the length of the *menD* fragment (right panel of Figure 3C). Notably, the last 15 nt of *menD* were sufficient for termination-reinitiation to occur. Comparison with the negative controls containing an additional stop codon at the end of the *glpD* reporter gene and thus lacking the translational fusion between *glpD* and the *menD* fragment further demonstrated that termination-reinitiation had occurred and *de novo* initiation could be ruled out.

The results for the *hyfH-hyfI* gene pair were similar, with two exceptions. For the fusions with only the last 15 nt of *hyfH*, high translational efficiencies were observed for *hyfI-gusA* in the absence of a stop codon at the end of *glpD* (in which case the ribosomes reach the gene overlap of *hyfH-hyfI* and terminate there) as well as in the presence of a stop codon, resulting in a 15 nt intergenic distance between *glpD* and *hyfI.* Obviously, the ribosomes can overcome this distance and reinitiate. These gene-specific differences between the *hyfH-hyfI* and *menD-menH* gene pairs shown in Figure 3C corroborated the differences observed in the experiments discussed above for a + 14 nt intergenic region (Figure 2C). A second gene-specific difference was that the translational efficiencies of the negative control construct were somewhat higher for *hyfH-hyfI* than for *menD-menH*, as already observed previously (Huber et al., 2019). Apparently, this difference was observed because some level of *de novo* initiation (10–30%) occurred at *hyfI*, but not at *menH*. Taken together, the results indicate that, for both gene pairs, the last 15 nt of the upstream gene were sufficient for translational coupling via termination-reinitiation.

### Unidirectional gene pairs typically overlap by 4 nucleotides in *Haloferax volcanii* and in *Escherichia coli*

**Figure 4:**
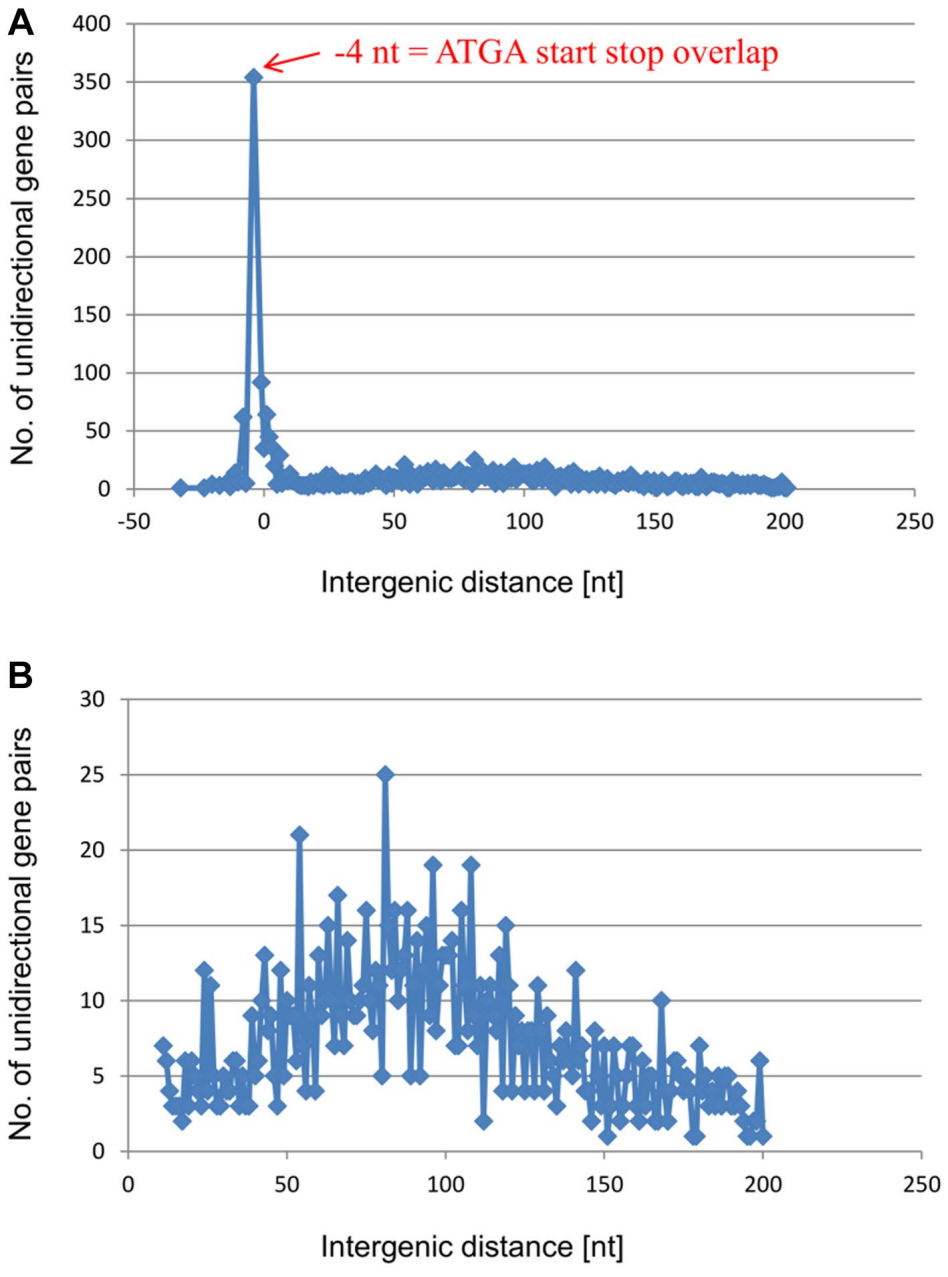
Distribution of intergenic lengths of unidirectional genes in the genome of *H. volcanii*. **(A)** Distribution of all unidirectional gene pairs with distances from −20 nt (overlap) to +200 nt. **(B)** Distribution of the gene pairs with distances between +10 and + 200 nt.

The results described above show, for six native gene pairs from an archaeon and a bacterium, that reinitiation is much more efficient for overlapping genes than for genes separated even by short intergenic distances. However, at least for *E. coli*, various polycistronic transcripts lacking gene overlaps have been described (Pallejà et al., 2009). In these cases, either translation of the distal gene is initiated *de novo*, or translational coupling requires long-range interactions. To assess the frequencies of the two initiation modes, we analyzed in detail the distributions of the intergenic distances in the unidirectional gene pairs of *H. volcanii* and in *E. coli*. In *H. volcanii* by far the most common configuration is the −4 nt overlap (*A**TG*A or *G**TG*A) which was observed in 354 gene pairs (Figure 4 and Supplementary Table S1). In addition, nearly 100 gene pairs have a − 1 nt overlap (TA*A**TG*, TG*A**TG*, or TA*G**TG*), and 62 gene pairs have an overlap of −8 nt, whereas longer overlaps are rare. Short intergenic distances, from 0 nt to 6 nt, are also represented by 20 to 60 occurrences for each length. Altogether, the region from −8 to +6 accounts for nearly 750 of the 2,511 unidirectional gene pairs. Thus, about 30% of the unidirectional gene pairs of *H. volcanii* are organized in gene pairs that allow efficient TeRe (Figure 4A). In addition, the distribution includes a shallow, broad peak of intergenic distances between about +50 nt to +150 nt that encompasses about 950 gene pairs, that is, about 38% of the unidirectional gene pairs (Figure 4B). Intergenic distances of more than 50 nt have enough room to accommodate the terminator of the upstream gene and the basal promoter elements (TATA box and BRE) of the downstream gene. Therefore, most of these genes are likely to be expressed as separate transcripts.

**Figure 5:**
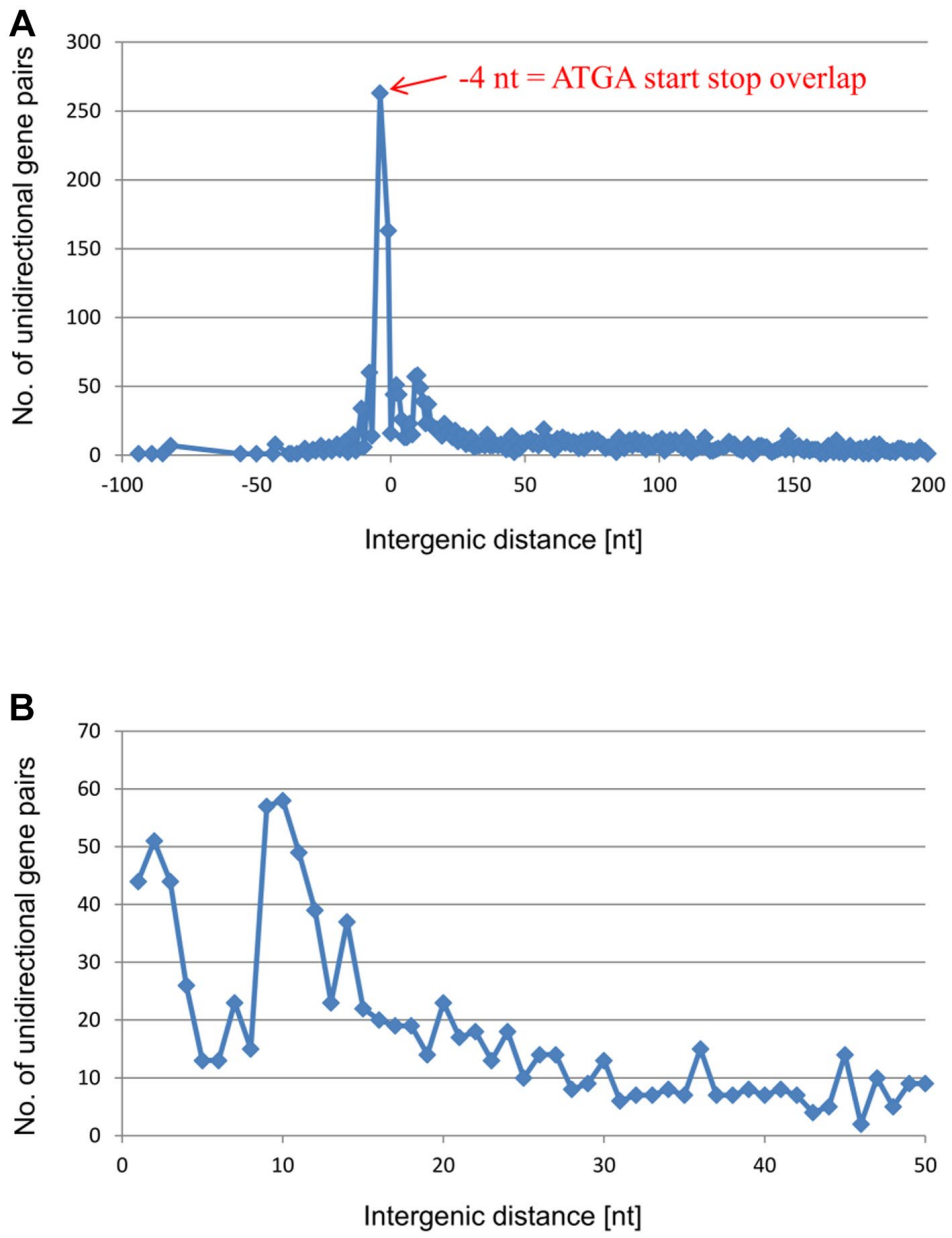
Distribution of intergenic lengths of unidirectional genes in the genome of *E. coli*. **(A)** Distribution of all unidirectional gene pairs with distances from −90 nt (overlap) to +200 nt. **(B)** Distribution of the gene pairs with distances between +1 and + 50 nt.

Also in *E. coli*, the −4 nt gene overlap *A**TG*A is the most common configuration in the unidirectional gene pairs, followed by the −1 nt and − 8 nt overlaps (Figure 5A and Supplementary Table S2). The region from −8 to +6 accounts for 707 gene pairs of the 2,987 unidirectional gene pairs (24% of the unidirectional pairs), not much lower than the corresponding values for *H. volcanii.* These observations suggest that *E. coli* also makes ample use of translational coupling via TeRe. However, the distributions of the remaining intergenic distances were notably different between the two species. The shallow peak of distances from +50 nt to +150 nt that is present in *H. volcanii* (Figure 4B) was missing in *E. coli* (Figure 5B). Instead, there was a small peak around +10/11 nt (Figure 5B). Altogether, there are 203 gene pairs with intergenic distances from +9 nt to +12 nt (7% of the unidirectional). Intergenic distances in this range are too short to accommodate a terminator and a promoter, suggesting that these gene pairs are expressed as single, polycistronic transcripts. However, the intergenic distances in such transcripts are long enough to harbor Shine-Dalgarno (ribosome-binding) motifs (Ringquist et al., 1992). Therefore, translation of the genes in these gene pairs is likely to be initiated independently or, alternatively, translational coupling depends on long-range interactions or/and pseudoknot formation. Indeed, for 201 of the 203 gene pairs with intergenic distances of +9 to +12 nt the transcript had been experimentally characterized, and 194 of the 201 gene pairs were indeed found on polycistronic transcripts (Supplementary Table S3) (Keseler et al., 2021).

### Distributions of intergenic distances in unidirectional gene pairs in 1,695 representative species of 49 phyla of bacteria and archaea

**Figure 6:**
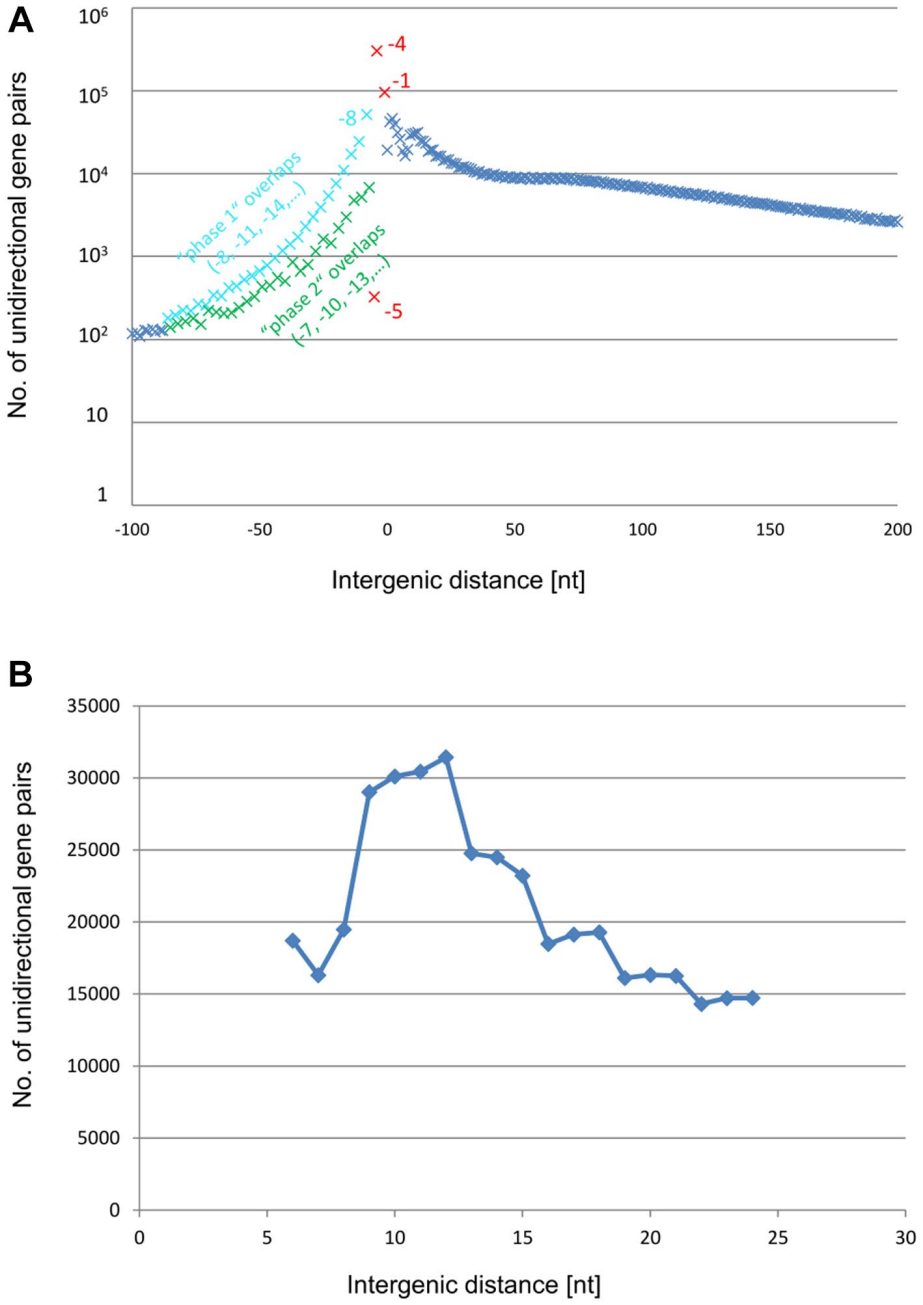
Distribution of average intergenic lengths of unidirectional genes. 2,661,236 unidirectional gene pairs in 1,695 prokaryotic species of 49 phyla. **(A)** Distribution of all unidirectional gene pairs with distances from −100 nt (overlap) to +200 nt. The results for the phase 1 overlaps are shown in blue, the results for the phase 2 overlaps are shown in green, and the results for the −5, −4, and −1 overlaps are highlighted in red. **(B)** Distribution of the gene pairs with distances between +6 and + 24 nt.

The analyses described above revealed shared as well as species-specific features of the distribution of the intergenic distances in unidirectional gene pairs in *H. volcanii* and *E. coli*. To explore more general trends, we expanded the analysis of these distances to a representative set of bacterial and archaeal genomes. To this end, we analyzed the distance distributions across 2,661,236 unidirectional gene pairs from 1,695 representative archaeal and bacterial species of 49 phyla. All these genes belonged to COGs (Clusters of Orthologous Genes) and were present as syntenic gene pairs in at least 100 species. Such conserved genes are typically well annotated, so that the intergenic distances should be considered reliable. Obviously, the −4 nt overlap (*A**TG*A or *G**TG*A) is on average by far most common throughout all major groups of prokaryotes, followed by the −1 nt overlap (TA*A**TG*, TG*A**TG*, or TA*G**TG*) and the −8 *ATG*NNTGA overlap (Figure 6A and Supplementary Table S4). All distances outside of the narrow window of −8 nt to +6 nt, a window indicative of TeRe, are at least an order of magnitude less common than the −4 overlap.

A −2 nt overlap is conspicuously absent because such an overlap with a canonical NTG initiation codon would imply an NNT stop codon (N*NT**G*), which does not exist in the known genetic codes, whereas usage of a canonical TRR stop would require a non-existent RRN start (T*RR**N*).

The frequency of the −5 nt overlap is more than 1,000-fold lower than that of the −4 nt overlap. The paucity of −5 nt overlaps was expected because all start codons contain a G in the third position, whereas all three stop codons start with a T. This incompatibility between translation start and stop seems to completely rule out a − 5 nt overlap. That some −5 overlaps were nevertheless observed, might be explained by annotation errors and/or by rare use of non-canonical start codons. The non-canonical start codon ATT can be involved in −5 nt overlaps with all three stop codons (*AT**T*GA, *AT**T*AG, *AT**T*AA). In an experimental analysis of the efficiencies of all 64 codons, ATT was the seventh best start codon, with about 0.3% efficiency compared to the best start codon ATG (Hecht et al., 2017).

As expected, gene pairs with long overlaps (between about −10 nt and − 60 nt) were far less frequent (by a factor of 4) than those with the short (−4 nt / -1 nt / -8 nt) overlaps. Unexpectedly, however, among these long overlaps, those in phase 1 (−8, −11, −14,…) were several fold more common than those in phase 2 (−7, −10, −13,…). Possible causes of this difference are addressed in the Discussion.

The distribution of intergenic distances included a small peak around +10, indicating that polycistronic transcripts apparently lacking TeRe or, alternatively, translating the downstream gene at a low efficiency are not uncommon (Figure 6B). Nevertheless, altogether, there were 679,023 predicted polycistronic transcripts with overlaps, apparently conducive to TeRe, compared to 193,477 predicted transcripts likely lacking TeRe. Thus, translational coupling via TeRe appears to be far more common across a broad range of bacteria and archaea than coupling via long-range interactions or *de novo* initiation at the downstream gene.

Notably, the variation of the intergenic distances between orthologous gene pairs is almost entirely dominated by the gene-specific (as opposed to phylum-specific) contribution. Decomposition of intergenic distances for 214 widely-occurring COG pairs (present in 25 or more phyla) produces the COG pair-specific and phylum-specific components with the former having 13-fold as large standard deviation than the latter (38.2 and 2.9 bp respectively).

### Overlapping and closely-spaced unidirectional gene pairs are enriched in genes encoding subunits of heteromeric complexes

The dominance of overlapping unidirectional gene pairs prompts the question of the evolutionary advantages of translational coupling via TeRe, which would override the disadvantage of the translation initiation region of the downstream gene being located within the coding region of the upstream gene. A plausible explanation could be the requirement for precise stoichiometry between the subunits of various heteromeric complexes (Huber et al., 2019). To explore this possibility, we manually inspected all unidirectional gene pairs in the genomes of *H. volcanii* and *E. coli*, and collected the available data on whether they encoded complex subunits. Based on the experimental and bioinformatics results described above, intergenic distances of −8 nt to +10 nt were supposed to be predictive for TeRe, while distances of more than 20 nt were supposed to be predictive for independent translation. Intergenic distances of +11 nt to +20 nt were omitted, because the experimental data have shown a high gene-specific variance in the efficiency of coupling.

We found that, in *E. coli*, 30.4% of all gene pairs with distances from −8 nt to +10 nt encoded subunits of heteromeric complexes, compared to 6.8% of the unidirectional gene pairs with longer intergenic distances of more than 20 nt, a 4.5 fold enrichment (Table 1). This difference was even greater for *H. volcanii*, where the corresponding fractions were 45.2 and 5.6%, respectively, a 8.1-fold enrichment that was highly statistically significant (χ^2^ test *p*-values of 2 × 10^−54^ for *E. coli* and 6 × 10^−74^ for *H. volcanii*). Thus, in both species, overlapping gene pairs preferentially encode subunits of heteromeric complexes, indicating that translational coupling via TeRe might be, at least in part, driven by selection for precise stoichiometry of interacting proteins.

**Table 1:** Putative formation of heteromeric complexes.

Protein class	*H. volcanii*		*E. coli*	
	No.	fraction	No.	fraction
Unidirectional gene pairs with short distances from − 8 to + 10:				
Heteromeric complex	253	45.2%	249	30.4%
No complex formation	307	54.8%	571	69.6%
Sum	560		820	
Unidirectional gene pairs with longer distances of > +20 nt:				
Heteromeric complex	51	5.6%	111	6.8%
No complex formation	859	94.4%	1,519	93.2%
Sum	910		1,630	
Enrichment of complex formation in gene pairs with short distances:				
	8.1-fold		4.5-fold	

We next tested whether a similar enrichment was detectable among the 2,661,236 unidirectional gene pairs from 1,695 representative species discussed above. The gene pairs were grouped into 2,972 syntenic COG pairs (Supplementary Table S5). Again, two groups were compared, namely, all COG pairs with median overlaps or intergenic distances of −8 nt to +10 nt vs. all COG pairs with median intergenic distances greater than 20 nt. The former group represents gene pairs with putative translational coupling via TeRe, whereas the latter group includes gene pairs assumed to lack TeRe. In this analysis, the inference that a gene pair encodes subunits of heteromeric complexes was solely based on the gene designations, that is, all *xyzA* gene products were assumed to interact with *xyzB* gene products (where A and B refer to the upstream and downstream genes in a gene pair). This inference might not be fully accurate (in particular, it could be overly conservative in the identification of gene pairs as heteromeric complex subunits because gene nomenclature does not always follow this convention), but both groups were large, containing 1,506 COG pairs and 991 COG pairs, respectively, so that differences could be expected to be relevant. In the former group, 35.5% of the gene pairs were predicted to encode subunits of heteromeric complexes based on the shared genes names, compared to 22.5% in the latter group. The enrichment of 1.6-fold identified in this analysis was not as pronounced as it was in *E. coli* or *H. volcanii*, but was nevertheless considerable. To assess the statistical significance, a bootstrap analysis was performed (details see Methods). All of the 1,000 resampling replications showed an enrichment of heteromeric complex subunits in the gene pairs with distances of −8 nt to +10 nt compared to longer distances of >20 nt. The enrichment factors were approximately log-normally distributed across the 1,000 replications, providing for an estimate of the statistical significance of enrichment (value of p of 5 × 10^−6^). Thus, these observations confirm that, across a broad diversity of bacteria and archaea, efficient formation of heteromeric complexes could be at least one of the factors behind the dominance of gene overlaps and translational coupling via TeRe.

## Discussion

Contradictory models have been proposed to explain the molecular mechanism of translational coupling. The scanning model for translational reinitiation posited that the post-termination 70S ribosome scanned along the mRNA until it reached a start codon (Adhin and Van Duin, 1990; Levin-Karp et al., 2013; Qin et al., 2016; Yamamoto et al., 2016). However, another study that also used a synthetic operon consisting of genes for two fluorescent proteins, produced a dramatically different result (Tian and Salis, 2015). Gene overlaps of up to −25 nt and small intergenic regions of up to +25 nt were analyzed, and even short intergenic regions of only +3 nt were found to lead to an about tenfold decrease of coupling efficiency, compared with the −4 nt overlap.

In contrast to these studies, which used phage genes (Adhin and Van Duin, 1990) or synthetic operons (Levin-Karp et al., 2013; Tian and Salis, 2015; Qin et al., 2016; Yamamoto et al., 2016), we studied native pairs of *E. coli* and *H. volcanii* genes. In all three analyzed cases, the reinitiation efficiencies turned out to be extremely distance dependent such that an intergenic distance of +14 nt caused a 40–80% drop in reinitiation efficiency, whereas an intergenic distance of +38 nt (almost) completely abolished reinitiation. These results suggest that the efficiency of ribosomal scanning at native pairs of *E. coli* genes is extremely low and is limited to very short intergenic distances. In congruence with these results indicating that reinitiation is primarily a local event, we also showed that the last 15 nt of the upstream gene are sufficient for translational coupling to occur.

The experimental study of translation reinitiation was complemented by extensive bioinformatic analyses of intergenic distance distributions in diverse bacteria and archaea. Initially, we analyzed in detail the genomes of *H. volanii* and *E. coli*, the two model species used for the experimental analyses. In both cases, the −4 nt overlap was by far the most frequent distance between two neighboring genes, followed by −1 and − 8 overlaps. These findings are generally compatible with previous observations (Wright et al., 2022).

However, to our knowledge, one distinct feature of the intergenic distance distributions has not been described thus far, namely, the small peak around the intergenic distance of +10 nt that was observed in the *E. coli* distribution (Figure 5B). The downstream genes in these pairs might be translated via translational coupling with very low efficiencies, or, more likely, via independent initiation.

This peak around +10 nt is missing in the distribution for *H. volcanii* which instead features a broad peak from about +50 nt to +150 nt, with a maximum around +80 nt (Figure 4B). These distances leave enough room to accommodate the transcription termination signal of the upstream genes and the transcription initiation signals (TATA box and BRE) of the downstream genes. Therefore, most likely, these gene pairs are expressed as separate transcripts. Supporting this view, a recent RNA-Seq analysis of the *H. volcanii* transcriptome has indeed shown that the majority of the transcripts are monocistronic (Laass et al., 2019). Only about one third of the *H. volcanii* transcripts are bicistronic, and polycistronic transcripts are rare. In most of these bicistronic and polycistronic transcripts, the coding sequences of the two genes overlap or are separated by very short intergenic distances, suggesting that their translation is coupled via TeRe.

We extended the bioinformatic analyses to 2,661,236 unidirectional gene pairs from 1,695 archaeal and bacterial species of 49 prokaryotic phyla, which is, to our knowledge, the largest analysis of intergenic distances so far available (Figure 6). By far the most common intergenic distance is the −4 nt gene overlap, followed by the −1 nt and the −8 nt overlap. Therefore, translational coupling via TeRe seems to be ubiquitous in prokaryotes.

The sharp decline of intergenic distance frequencies on both sides of the small region encompassing the −8/−4/−1 nt overlaps is another indication that 70S scanning over large distances does not commonly occur. Ribosomal profiling of a wildtype *E. coli* strain and a strain in which the ribosome recycling factor (RRF) was depleted showed that, in the absence but not in the presence of RRF, post-termination 70S ribosomes were enriched in the 3’-UTRs of transcripts (Saito et al., 2020). The number of 70S ribosomes in 3’-UTRs could be reduced by a high salt treatment, indicating that these 70S ribosomes were not translating. Unidirectional gene pairs with small overlaps were analyzed specifically. It was found that the ribosome density was similar in the wildtype and the RRF-depleted strain, indicating that RRF does not play a role in the translation of overlapping genes. From these results, the conclusion was made that reinitiation was not the dominant mechanism for translational coupling in *E. coli*, and *de novo* initiation was more common (Saito et al., 2020). However, this conclusion would hold true only if RRF was required for ribosome dissociation such that reinitiation would occur through the mRNA-bound 30S subunit, whereas the 50S subunit is exchanged. Although this is the reinitiation mechanism in some eukaryotic viruses (Powell et al., 2008; Powell, 2010), the mechanism in bacteria remains unknown. Should short overlaps allow the 70S subunit to re-initiate after repositioning by 1 or 4 nt, RRF would not be needed for TeRe. The high frequency of gene pairs with short overlaps in 1,695 species of diverse bacteria and archaea appears to be a strong argument in favor of translational coupling, in contrast to *de novo* initiation at the downstream gene. Reinforcing this view, a recent study that measured the translation kinetics in *E. coli* found that the 30S and the 50S subunits spent the same time on transcripts, ruling out 30S scanning as a mechanism for reinitiation (Metelev et al., 2022). Instead, it was concluded that more than 50% of the 70S complexes re-initiated translation on polycistronic mRNAs without dissociation. A RRF-independent “transient idling” of post-termination 70S ribosomes followed by reinitiation at a nearby start codon has already been proposed in an earlier study, which analyzed a series of mutants of the *phoA* gene of *E. coli* (Karamyshev et al., 2004).

It seemed surprising that there was a considerable difference in occurrence between phase 1 overlaps and phase 2 overlaps for longer gene overlaps from about −10 nt to about −70 nt (Figure 6). A probable explanation for this several-fold phase difference can be gleaned from the route of evolution of overlapping gene pairs. It is generally assumed that overlapping genes evolved from originally non-overlapping unidirectional gene pairs with short intergenic distances (Fukuda et al., 2003; Cock and Whitworth, 2010; Huvet and Stumpf, 2014; Wright et al., 2022). The most frequent mechanism is the mutation of the stop codon of the upstream gene to a sense codon, so that the next in-frame stop codon has to be used, which by chance is located somewhere within the ORF of the downstream gene. Typically, this leads to a long overlap of the two genes without translational coupling. Because translational coupling is of evolutionary advantage (see below), additional mutations leading to very short overlaps allowing efficient TeRe are positively selected. Such mutations are possible for phase 2 overlaps between the two genes, leading to the observed enrichment of the −4 nt and −1 nt overlaps with concomitant depletion of long phase 2 overlaps. The higher occurrence of the −4 nt overlap can be explained because a single mutation is necessary to transform an ATG start codon to the *A**TG*A – 4 nt overlap, while typically two mutations are needed to transform an ATG start codon to the TG*A**TG* − 1 nt overlap. In stark contrast, such mutations are not possible for phase 1 overlaps between the two genes, because −2 nt overlaps and −5 nt overlaps are impossible based on the sequences of stop and start codons (see above). The shortest possible overlap which can be generated from long phase 1 overlaps is the −8 nt overlap. However, the generation of a new stop codon typically requires three mutations, and, in addition, the −8 nt overlap has a lower TeRe efficiency than shorter overlaps. Together, these two reasons explain the lower enrichment of −8 nt overlaps and the higher retention of long phase 1 overlaps, compared to long phase 2 overlaps. Notably, in this scenario the evolutionary advantages of TeRe drive both the enrichment of gene pairs with very short overlaps and the phase difference of gene pairs with long overlaps.

In our previous work, we showed that translation at 14 native unidirectional gene pairs from *H. volcanii* and *E. coli* was strictly coupled (Huber et al., 2019). One possible advantage of translational coupling is the efficient co-translational formation of heteromeric complexes. Multiple lines of evidence point to co-translational complex formation in bacteria as well as in eukaryotes (Duncan and Mata, 2011; Marsh et al., 2013; Shieh et al., 2015; Wells et al., 2016; Natan et al., 2017; Shiber et al., 2018) although such evidence is currently missing for archaea. We analyzed whether gene pairs with short overlaps or very short intergenic regions were enriched in genes encoding subunits of heteromeric complexes, compared to gene pairs with longer intergenic distances. Indeed, a highly significant, 4.5-fold enrichment was observed for *E. coli* gene pairs, and an even more pronounced 8.1-fold enrichment for *H. volcanii* gene pairs. Across 1,695 species from 49 bacterial and archaeal phyla, a lesser, even if also highly significant, 1.6-fold enrichment of heteromeric complex subunits was observed. The lower enrichment factor in the large gene set might be due to the low accuracy of gene annotation for many genomes which we had to use for the automatic assignment of gene products to the same complex.

## Conclusion

The convergence of experimental observations on three gene pairs from the archaeon *H. volcanii* and the bacterium *E. coli*, and extensive bioinformatics analysis strongly suggest that translation reinitiation in polycistronic mRNAs enabled by short gene overlaps is common among bacteria and archaea. This translational coupling of adjacent unidirectional genes was found to be extremely distance-dependent such that an intergenic distance of only 20 nt nearly completely abolished reinitiation at the downstream gene. Overlapping gene pairs are substantially enriched in genes encoding subunits of heteromeric protein complexes suggesting that selection for precise stoichiometry of interacting proteins is one of the factors that drive the evolution of this gene arrangement.

## Materials and methods

### Archaeal and bacterial strains, media, and growth conditions

The *H. volcanii* strain H26 (Allers et al., 2004) was used as a wildtype in this study. It contains a deletion of the *pyrE* gene and is thus auxotrophic for uracil. This enables a forward selection for the presence of *pyrE*-containing plasmids in uracil-free medium, and a reverse selection for plasmid-absence in medium with uracil and 5-fluororotic acid (5-FOA). In addition, the gene *HVO_1279* encoding dihydrofolate reductase has been deleted in the genome (Gäbel et al., 2013). This enables the usage of a plasmid-encoded copy of the *dhfr* gene as a reporter gene. A plasmid-encoded copy of the *araDH* gene (*HVO_B0032*) was used as a second reporter gene, which is possible because the chromosomal copy is not transcribed in the absence of arabinose. *H. volcanii* was grown in complex medium as described (Huber et al., 2019).

The *E. coli* strain JW3389-1 was obtained from the Keio collection (Baba et al., 2006). It contains a chromosomal deletion of the *glpD* gene, and, therefore, allows to use a plasmid-bound *glpD* gene as a reporter gene without any background. To enable the simultaneous use of the *gusA* gene as a reporter gene, the chromosomal *gusA* copy has also been deleted (Wegener et al., 2016). *E. coli* was grown in SOB^+^ medium as described (Huber et al., 2019).

### Generation of double reporter gene plasmids and mutagenesis

General molecular biology methods were performed according to Green and Sambrook (2012). The sequences of all oligonucleotides used for cloning and mutagenesis are listed in Supplementary Table S6.

For the determination of reinitiation efficiencies in *H. volcanii* a shuttle vector was used that contains replication origins and selection genes for *E. coli* and *H. volcanii*, and that has been described previously (Huber et al., 2019). In the previous study only the *dhfr* gene was used as a reporter gene, which had been used in translational fusions to the downstream genes of several native gene pairs of *H. volcanii* (Huber et al., 2019). To generate a double-reporter gene vector, the *araDH* gene encoding arabinose dehydrogenase was amplified from the genome of *H. volcanii* and cloned as a translational fusion to the upstream genes of selected native gene pairs (the oligonucleotides are listed in Supplementary Table S6). The cloned regions of all overlapping gene pairs are summarized in Supplementary Table S7.

For the determination of reinitiation efficiencies in *E. coli*, a shuttle vector was used that contained replication origins and selection genes for *E. coli* and *H. volcanii*, and that has been described preciously (Huber et al., 2019). It contained translational fusions of the *glpD* reporter gene with the upstream genes of selected gene pairs and the respective downstream genes with the *gusA* reporter gene. Because non-translated transcripts are very unstable in *E. coli*, only 99 nt of the upstream genes and 30 nt of the downstream genes were used (Huber et al., 2019). The cloned regions of all overlapping gene pairs are summarized in Supplementary Table S7.

For the generation of mutants containing premature stop codons in the ORF of the upstream genes, the gene pairs were excised from the expression vectors and cloned into the small *E. coli* vector bluescript pSK+.^1^ The quickchange site-directed mutagenesis kit (see Footnote 2) was used to introduce premature stop codons into the upstream genes of selected gene pairs with the oligonucleotides listed in Supplementary Table S6. The cloned regions of all constructs are summarized in Supplementary Table S7. The sequences of the resultant mutated vectors were verified by sequencing. Subsequently the mutated gene pairs were excised from pSK+ and cloned back into the expression vectors. The sequences were again verified, and the shuttle vectors were introduced into *H. volcanii* or *E. coli*, respectively, for the determination of the reinitiation efficiencies.

### Determination of translational efficiencies

Cultures of *H. volcanii* were grown to mid-exponential growth phase (4–5 × 10^8^ cells mL^−1^) in the medium described above. Aliquots were removed for the quantification of the specific enzymatic activities of AraDH and DHFR as well as for the quantification of the transcript levels as described below. These values were used to calculate normalized translational efficiencies (see below). In each case at least three biological replicates were performed, and average values and their standard deviation were calculated.

Cultures of *E. coli* were grown to mid-exponential growth phase (OD_600_ about 0.6) in the SOB^+^ medium described above. The P_BAD_ promoter was induced with 0.2% (w/v) arabinose for 30 min. Aliquots were removed for the quantification of the specific enzymatic activities of GlpD and GusA as well as for the quantification of the transcript levels as described below. These values were used to calculate normalized translational efficiencies (see below). In each case at least three biological replicates were performed, and average values and their standard deviation were calculated. Supplementary Figure S1 shows typical results and gives an overview of the workflow for quantification of the translational efficiencies.

### Quantification of specific reporter enzyme activities

The volume activities of the four reporter enzymes (kat/mL) were quantified using enzymatic assays. The protein concentrations (mg/mL) were quantified using the BCA assay^2^ with a standard curve comprised of various concentrations of BSA (bovine serum albumin). The specific activities (kat/mg) were calculated by dividing the two values. Detailed protocols for the four assays have been published previously (Wegener et al., 2016; Huber et al., 2019) and have been deposited at the Nature Protocol Exchange Database at the following websites:

DHFR: https://protocolexchange.researchsquare.com/article/pex-534/v1

AraDH: https://doi.org/10.21203/rs.3.pex-2413/v1

GlpD: https://protocolexchange.researchsquare.com/article/pex-472/v1

GusA: https://protocolexchange.researchsquare.com/article/pex-473/v1

In short, the DHFR activity was quantified following the oxidation of NADPH at 340 nm using an extinction coefficient of 6.22 mM^−1^ cm^−1^, the AraDH activity was quantified following the reduction of NAD^+^ at 340 nm using an extinction coefficient of 6.22 mM^−1^ cm^−1^, the GlpD activity was quantified following the reduction of the artificial electron acceptor MTT at 570 nm using an extinction coefficient of 17 mM^−1^ cm^−1^, and the GusA activity was quantified following formation of p-nitrophenol (from para-nitrophenyl-glucuronic acid) at 405 nm using an extinction coefficient of 218 mM^−1^ cm^−1^.

### Quantification of transcript levels

The samples for RNA isolation were taken simultaneously with the samples for determination of the specific reporter enzyme activities. Total RNA was isolated using the RNeasy Mini kit (Qiagen, Hilden, Germany). Relative transcript levels were quantified by Northern blot analyses. A detailed description of the procedure has been published previously (Wegener et al., 2016; Huber et al., 2019) and has been deposited at the Nature Protocol Exchange Database: https://protocolexchange.researchsquare.com/article/pex-535/v1.

The oligonucleotides that were used for the generation of digoxigenin (DIG)-labeled probes via PCR are listed in Supplementary Table S6. After hybridization, an enzyme-coupled anti-DIG antibody and the chemoluminescence substrate CDP-Star (Roche, Mannheim, Germany) were used to assay the probes, and X-ray films were used to detect the emitted light. The films were scanned and the signals were quantified using the software ImageJ.^3^ The signals were normalized to the amounts of 16S rRNA. Three biological replicates were performed, and average values and their standard deviation were calculated.

### Calculation of normalized translational efficiencies

Translational efficiencies were calculated by dividing the specific reporter enzyme activities (see above) by the relative transcript levels (see above). A strain with an empty vector was used as a negative control, and its average value was subtracted from the values of all samples containing reporter genes. For a better visualization, the results of the mutants were normalized to that of the wildtype. A detailed description of the procedure has been published previously (Huber et al., 2019) and has been deposited to the Nature Protocol Exchange Database: https://protocolexchange.researchsquare.com/article/pex-553/v1. Supplementary Figure S1 shows typical results and gives an overview of the workflow for quantification of the translational efficiencies.

### Computational analyses of the *Haloferax volcanii* and *Escherichia coli* genomes

Gene distance analyses of the *H. volcanii* and *E. coli* genomes were performed by custom PERL scripts. For *H. volcanii* wildtype strain DS2, the internal, manually curated genome annotation from 19-JUN-2019 was used (the theoretical proteome of this annotation is available via zenodo under DOI 10.5281/zenodo.3565631). For *E. coli* strain K-12 MG1655, GenBank accession U00096 was used (downloaded on 24-SEP-2020).

Genome annotations were converted to an internal format where each coding region is represented by three values, start position, stop position, and coding strand. Start position is always smaller than stop position. Thus, the start position of a gene encoded on the forward strand corresponds to the protein N-terminus (first base of start codon) while the start position of a gene encoded on the reverse strand corresponds to the last base of the stop codon.

For *H. volcanii*, where an elaborate genome annotation is available, a first cleanup step was performed. If a gene is targeted by a transposon, the affected gene is split into two fragments, with the transposase gene of the transposon located internally. Such gene-internal transposases were removed from the list of protein-coding genes before further processing.

Protein-coding genes were sorted along each replicon by the start position of the coding region and gene pairs were extracted from the sorted list. Each gene pair was assigned to one of three categories as serially encoded, divergently encoded or convergently encoded. Only serially encoded gene pairs were analyzed further.

For *H. volcanii*, where an elaborate genome annotation is available, a second cleanup step was performed. Disrupted genes (pseudogenes) may be terminally truncated, thus lacking a start codon and/or a stop codon. Gene pairs involving truncated gene termini were removed from the list of gene pairs before further processing.

In case of serially encoded gene pairs, one gene begins at its start codon and one gene terminates at its stop codon. The gene distance was computed as the position difference between the stop codon of one gene and the start codon of the other gene. In case of a gene overlap, this distance is a negative value.

From these results, various statistical values were computed for serially encoded gene pairs. It should be noted that genes for stable RNAs (e.g., rRNAs, tRNAs) were not taken into account in this analysis. Thus, some gene distance values were too large. Due to the low number of stable RNA genes, we expect only a small impact on the statistical results, especially as large gene distances were not in the focus of the current analysis.

Gene pairs and their distance were logged into output files in *ad-hoc* formats.

Statistical significance of the enrichment with heteromeric complex subunits among the gene pairs at distances of −8 nt to +10 nt vs. distances greater than 20 nt was performed using a χ^2^ test on a 2×2 contingency table (complex status vs. distance).

### Computational analyses of 1,695 representative genomes of 49 prokaryotic phyla

A collection of completely sequenced prokaryotic genomes, available at NCBI as of November 2021, contained assemblies, belonging to 1,965 recognized genera in 9 archaeal and 40 bacterial phyla. Supplementary Table S8 contains the names of all species and their phylogenetic grouping. A representative genome assembly (the largest) was selected for each genus. Annotated proteins were assigned to COGs using PSI-BLAST search with CDD COG profiles as queries. Pairs of co-directed genes, where both genes were assigned to a known COG, were collected; the distances between the stop codon of the upstream gene and the start codon of the downstream gene were determined from the existing genome annotation. Phase for non-overlapping genes (separated by a distance k≥0 ) was defined as p=k−3k/3 ; phase for overlapping genes (separated by a distance k<0 ) was defined as p=k+3−k/3 .

311,918 gene pairs representing 241 widely distributed COG combinations (present in at least 25 distinct phyla) were selected to determine phylum-specific correction factors. To this end, distances for the *k*-th observation that is classified as belonging to the *i*-th COG pair in the *j*-th phylum were modeled as $d_{k\left[i,j\right]}=C_{i}+P_{j}+e_{k}$ , where $C_{i}$ is the vector of 214 COG pair-specific factor values, $P_{j}$ is the vector of 49 phylum-specific factor values (with $P_{\mathrm{Proteobacteria}}$ defined to be 0) and $e_{k}$ is a deviation, unaccounted for by these two factors. Iterative minimization of $\Sigma e_{k}^{2}\;$ across all observations using the *optim*() function in R yielded the phylum-specific correction factors that were subtracted from observed distances to obtain the taxonomically adjusted distances. These adjusted distances were used to calculate the mean distance for all COG pairs, accounting for the uneven distribution of these pairs across the bacterial and archaeal taxa.

To estimate the statistical robustness of the enrichment with heteromeric complex subunits among the gene pairs at distances of −8 nt to +10 nt vs. distances greater than 20 nt the following test was performed. First, 2,972 COG pairs were classified according to their gene names into likely subunits of heteromeric complexes (991 pairs) and otherwise (1,506 pairs). These COG pairs are represented by 1,016,930 gene pairs in the dataset. Then on each resampling iteration a bootstrap-like sample of 2,972 COG pairs (where each pair was selected with a probability of 1-*e*^−1^ ≈ 0.63) was taken; then a sample of 1,016,930 gene pairs (with replacement) was taken out of gene pairs, representing the selected COG pairs. Relative enrichment of subunits of heteromeric complexes among the genes at distances of −8 nt to +10 nt compared to distances greater than 20 nt was calculated for each sample.

Distribution of the logarithms of the 1,000 enrichment values, obtained for independent samples, can be used to estimate the robustness of the observed enrichment to the gene sampling. An observed z-score (the difference between the mean and zero, divided by the standard deviation) of 4.4 corresponds to the normal approximation *p*-value of 5×10^−6^.

## Data availability statement

The original contributions presented in the study are included in the article/Supplementary material, further inquiries can be directed to the corresponding author.

## Author contributions

MH: Data curation, Formal analysis, Investigation, Methodology, Writing – review & editing. NV: Data curation, Formal analysis, Investigation, Methodology, Visualization, Writing – review & editing. AB: Formal analysis, Supervision, Visualization, Writing – review & editing. FP: Conceptualization, Formal analysis, Investigation, Methodology, Writing – review & editing. SK: Formal analysis, Investigation, Methodology, Software, Writing – review & editing. YW: Conceptualization, Formal analysis, Investigation, Software, Supervision, Writing – review & editing. EK: Conceptualization, Funding acquisition, Project administration, Resources, Supervision, Writing – original draft, Writing – review & editing. JS: Conceptualization, Formal analysis, Funding acquisition, Supervision, Writing – original draft, Writing – review & editing.

## Footnotes

^1^ www.agilent.com

^2^ www.thermofischer.com

^3^ http://rsweb.nih.gov/ij
