# Supplementary material for: Unidirectional gene pairs in archaea and bacteria require overlaps or very short intergenic distances for translational coupling via termination-reinitiation and often encode subunits of heteromeric complexes
Source: Front Microbiol. 2023 Nov 9;14:1291523. doi: 10.3389/fmicb.2023.1291523 (PMC10666635; doi:10.3389/fmicb.2023.1291523)
Supplement: Supplementary file 1 [file Data_Sheet_1.PDF]

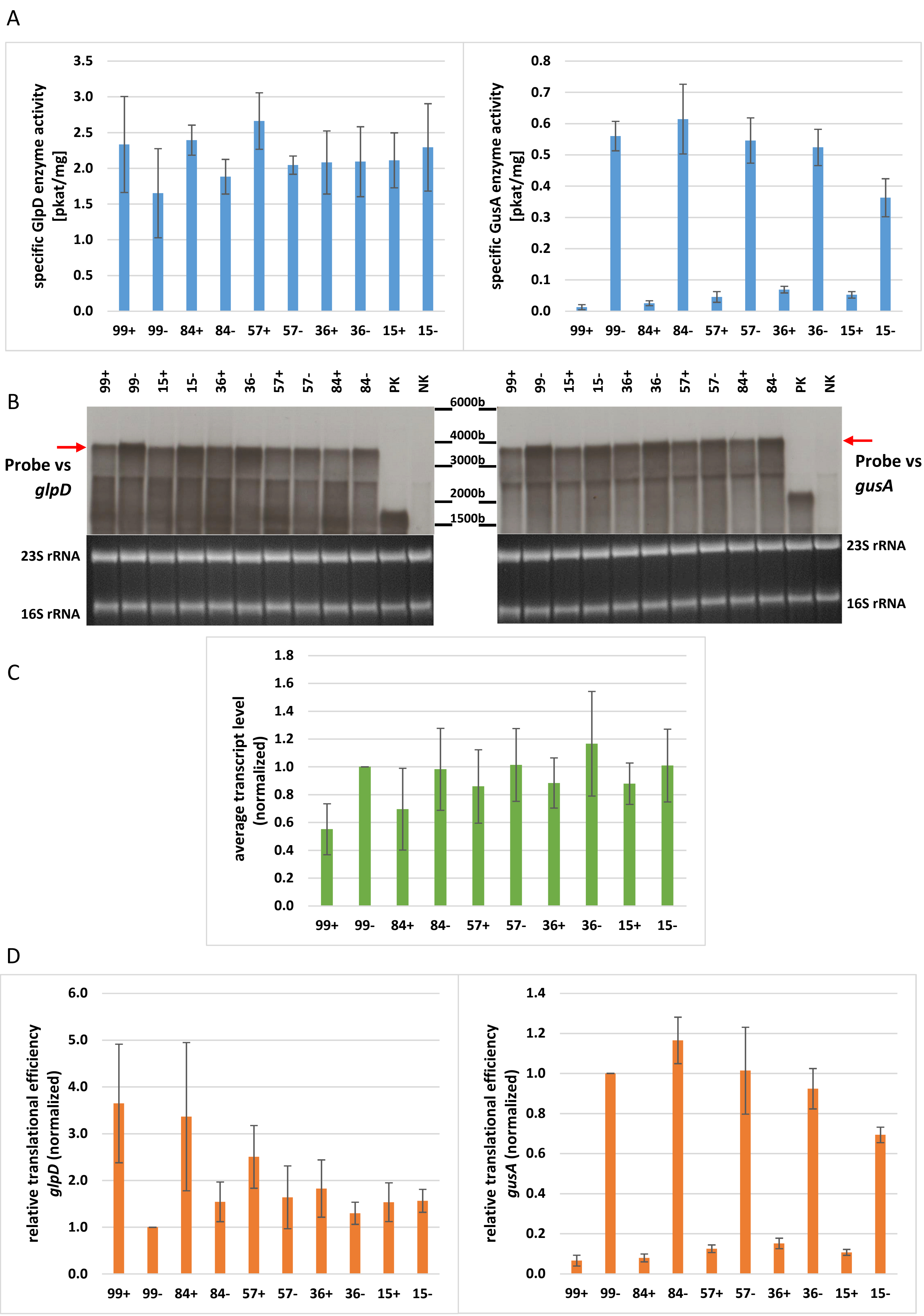
A
specific GlpD enzyme activity [pkat/mg]
3.5
3.0
2.5
2.0
1.5
1.0
0.5
0.0
99+ 99- 84+ 84- 57+ 57- 36+ 36- 15+ 15-
specific GusA enzyme activity [pkat/mg]
0.8
0.7
0.6
0.5
0.4
0.3
0.2
0.1
0.0
99+ 99- 84+ 84- 57+ 57- 36+ 36- 15+ 15-
B
99+ 99- 15+ 15- 36+ 36- 57+ 57- 84+ 84- PK NK
Probe vs *glpD*
6000b
4000b
3000b
2000b
1500b
Probe vs *gusA*
23S rRNA
16S rRNA
C
average transcript level (normalized)
1.8
1.6
1.4
1.2
1.0
0.8
0.6
0.4
0.2
0.0
99+ 99- 84+ 84- 57+ 57- 36+ 36- 15+ 15-
D
relative translational efficiency *glpD* (normalized)
6.0
5.0
4.0
3.0
2.0
1.0
0.0
99+ 99- 84+ 84- 57+ 57- 36+ 36- 15+ 15-
relative translational efficiency *gusA* (normalized)
1.4
1.2
1.0
0.8
0.6
0.4
0.2
0.0
99+ 99- 84+ 84- 57+ 57- 36+ 36- 15+ 15-

Supplementary Figure S1: Overview of the calculation of translational efficiencies based on representative results for the quantification of reporter enzyme activities and transcript levels. An example of the *menD/menH* gene pair of *E.coli* is shown. The enzyme assays were performed as described previously (PMID: 31488843). A) Exemplary results for the specific enzyme activities are shown for reporter genes GlpD (left side) and GusA (right side). +: Stop codon at the end of *glpD*, inhibiting translational coupling. -: translational fusion of *glpD* and *menD*, enabling translational coupling of *menD/H*. B) Top panel: Exemplary Northern blot for *glpD* (left side) and *gusA* (right side). Bottom panel: the corresponding 23S and 16S rRNA. The red arrows indicate the position of the full-length transcript. C) The amount of full-length transcript and the corresponding 16S rRNA were quantified densitometrically using ImageJ and the relative transcript levels were calculated. The average relative transcript levels were normalized to the wild type (99-). D) From the measured specific enzyme activity (A) and the calculated relative transcript levels (C), relative translational efficiencies (enzyme activity/transcript level) for *glpD* (left side) and *gusA* (right side) are calculated. Average values and SD of three biological replicates are shown in A, C and D. One representative of a Northern blot is shown in B.
