# Supplementary material for: Unidirectional gene pairs in archaea and bacteria require overlaps or very short intergenic distances for translational coupling via termination-reinitiation and often encode subunits of heteromeric complexes
Source: Front Microbiol. 2023 Nov 9;14:1291523. doi: 10.3389/fmicb.2023.1291523 (PMC10666635; doi:10.3389/fmicb.2023.1291523)
Supplement: Supplementary file 2 [file Table_1.DOCX]

**Supplementary Table S1.** Prediction of heteromeric complex formation for gene products of unidirectional gene pairs of *H. volcanii*. The genome of *H. volcanii* wild-type DS2 was manually inspected. For all unidirectional gene pairs it was predicted whether their gene products are known to be subunits of heteromeric complexes (“CMPLX yes”) or whether no information about complex formation exists (“CMPLX not”). For all unidirectional gene pairs four result lines were generated, i.e. 1) the number of the gene pair and the intergenic distance of the genes, 2) the prediction of complex formation, 3) the HVO_number and functional annotation of the upstream gene, and 4) the HVO_number and functional annotation of the downstream gene.

>NGHB#3 142

CMPLX not

HVO_0003 polD1 DNA-directed DNA polymerase D exonuclease subunit DP1

HVO_0004 - NamA family oxidoreductase

//

>NGHB#5 108

CMPLX not

HVO_0006 - acetyltransferase domain protein

HVO_0005 gufA1 GufA family transport protein (probable substrate zinc)

//

>NGHB#7 224

CMPLX not

HVO_0008 lysC aspartate kinase

HVO_0009 tnaA tryptophanase

//

>NGHB#9 48

CMPLX not

HVO_0011 purO inosine-5'-monophosphate cyclohydrolase, archaeal-type

HVO_0010 - MJ0936 family phosphodiesterase

//

>NGHB#10 368

CMPLX not

HVO_0013 cgi121 KEOPS complex subunit Cgi121

HVO_0011 purO inosine-5'-monophosphate cyclohydrolase, archaeal-type

//

>NGHB#11 -4

CMPLX not

HVO_0014 hel308a ATP-dependent DNA helicase Hel308a

HVO_0013 cgi121 KEOPS complex subunit Cgi121

//

>NGHB#13 253

CMPLX ---

HVO_0015 ferC ferredoxin (4Fe-4S)

HVO_0016 - conserved hypothetical protein

//

>NGHB#15 93

CMPLX ---

HVO_0018 - DUF2391 family protein

HVO_0017 - conserved hypothetical protein

//

>NGHB#18 103

CMPLX yes

HVO_0021 thiP ABC-type transport system permease protein (probable substrate thiamine)

HVO_0020 thiQ ABC-type transport system ATP-binding protein (probable substrate thiamine)

//

>NGHB#19 9

CMPLX yes

HVO_0022 thiB ABC-type transport system periplasmic substrate-binding protein (probable substrate thiamine)

HVO_0021 thiP ABC-type transport system permease protein (probable substrate thiamine)

//

>NGHB#27 6

CMPLX not

HVO_0029 uvrB UvrABC system protein B

HVO_0030 - NUDIX family hydrolase (homolog to 8-oxo-dGTP phosphatase)

//

>NGHB#28 223

CMPLX ---

HVO_0030 - NUDIX family hydrolase (homolog to 8-oxo-dGTP phosphatase)

HVO_0031 - conserved hypothetical protein

//

>NGHB#29 14

CMPLX ---

HVO_0031 - conserved hypothetical protein

HVO_0032 - TSUP family transport protein

//

>NGHB#30 69

CMPLX not

HVO_0032 - TSUP family transport protein

HVO_0033 - UspA domain protein

//

>NGHB#35 50

CMPLX ---

HVO_0036 - conserved hypothetical protein

HVO_0037 - phosphodiesterase domain protein

//

>NGHB#37 81

CMPLX not

HVO_0039 rad3b DNA repair helicase Rad3

HVO_0038 cyc1 cytochrome P450

//

>NGHB#40 83

CMPLX not

HVO_0042 argE probable [ArgW]-ornithine hydrolase

HVO_0041 argF ornithine carbamoyltransferase

//

>NGHB#41 -4

CMPLX not

HVO_0043 argD probable [ArgW]-glutamate semialdehyde aminotransferase

HVO_0042 argE probable [ArgW]-ornithine hydrolase

//

>NGHB#42 -4

CMPLX not

HVO_0044 argB probable [ArgW]-glutamate kinase

HVO_0043 argD probable [ArgW]-glutamate semialdehyde aminotransferase

//

>NGHB#43 54

CMPLX not

HVO_0045 argC probable [ArgW]-L-glutamate phosphate reductase

HVO_0044 argB probable [ArgW]-glutamate kinase

//

>NGHB#44 -4

CMPLX not

HVO_0046 argX probable glutamate--argW ligase

HVO_0045 argC probable [ArgW]-L-glutamate phosphate reductase

//

>NGHB#45 1

CMPLX not

HVO_0047 argW probable biosynthetic carrier protein ArgW

HVO_0046 argX probable glutamate--argW ligase

//

>NGHB#46 320

CMPLX not

HVO_0048 argH argininosuccinate lyase

HVO_0047 argW probable biosynthetic carrier protein ArgW

//

>NGHB#47 1

CMPLX not

HVO_0049 argG argininosuccinate synthase

HVO_0048 argH argininosuccinate lyase

//

>NGHB#48 274

CMPLX ---

HVO_0050 - hypothetical protein

HVO_0049 argG argininosuccinate synthase

//

>NGHB#49 93

CMPLX ---

HVO_0051 kef2 Kef-type transport system (probable substrate potassium)

HVO_0050 - hypothetical protein

//

>NGHB#50 -1

CMPLX yes

HVO_0052 - probable Kef-type transport system accessory protein

HVO_0051 kef2 Kef-type transport system (probable substrate potassium)

//

>NGHB#51 43

CMPLX ---

HVO_0053 - conserved hypothetical protein

HVO_0052 - probable Kef-type transport system accessory protein

//

>NGHB#52 6

CMPLX ---

HVO_0054 glyS glycine--tRNA ligase

HVO_0053 - conserved hypothetical protein

//

>NGHB#53 -8

CMPLX not

HVO_0055 - CBS domain protein

HVO_0054 glyS glycine--tRNA ligase

//

>NGHB#54 132

CMPLX ---

HVO_0056 - conserved hypothetical protein

HVO_0055 - CBS domain protein

//

>NGHB#55 61

CMPLX ---

HVO_0057 - conserved hypothetical protein

HVO_0056 - conserved hypothetical protein

//

>NGHB#56 2

CMPLX ---

HVO_0058 dppF1 ABC-type transport system ATP-binding protein (probable substrate dipeptide/oligopeptide)

HVO_0057 - conserved hypothetical protein

//

>NGHB#57 -4

CMPLX yes

HVO_0059 dppD1 ABC-type transport system ATP-binding protein (probable substrate dipeptide/oligopeptide)

HVO_0058 dppF1 ABC-type transport system ATP-binding protein (probable substrate dipeptide/oligopeptide)

//

>NGHB#58 -4

CMPLX yes

HVO_0060 dppC1 ABC-type transport system permease protein (probable substrate dipeptide/oligopeptide)

HVO_0059 dppD1 ABC-type transport system ATP-binding protein (probable substrate dipeptide/oligopeptide)

//

>NGHB#59 6

CMPLX yes

HVO_0061 dppB1 ABC-type transport system permease protein (probable substrate dipeptide/oligopeptide)

HVO_0060 dppC1 ABC-type transport system permease protein (probable substrate dipeptide/oligopeptide)

//

>NGHB#60 212

CMPLX yes

HVO_0062 dppA1 ABC-type transport system periplasmic substrate-binding protein (probable substrate dipeptide/oligopeptide)

HVO_0061 dppB1 ABC-type transport system permease protein (probable substrate dipeptide/oligopeptide)

//

>NGHB#62 84

CMPLX ---

HVO_0063 - conserved hypothetical protein

HVO_0064 - DUF296 family protein

//

>NGHB#63 2

CMPLX not

HVO_0064 - DUF296 family protein

HVO_0065 polD2 DNA-directed DNA polymerase D large subunit

//

>NGHB#66 70

CMPLX not

HVO_0067 - small CPxCG-related zinc finger protein

HVO_0068 panE 2-dehydropantoate 2-reductase

//

>NGHB#68 78

CMPLX not

HVO_0070 - NifU C-terminal domain protein

HVO_0069 - AlkP-core domain protein

//

>NGHB#69 44

CMPLX ---

HVO_0071 - conserved hypothetical protein

HVO_0070 - NifU C-terminal domain protein

//

>NGHB#70 158

CMPLX ---

HVO_0072 - conserved hypothetical protein

HVO_0071 - conserved hypothetical protein

//

>NGHB#73 136

CMPLX not

HVO_0075 - major facilitator superfamily transport protein

HVO_0073 recJ1 single-stranded-DNA-specific exonuclease RecJ1

//

>NGHB#74 1

CMPLX not

HVO_0076 hemD uroporphyrinogen-III synthase

HVO_0075 - major facilitator superfamily transport protein

//

>NGHB#75 -4

CMPLX not

HVO_0077 sirA uroporphyrin-III C-methyltransferase

HVO_0076 hemD uroporphyrinogen-III synthase

//

>NGHB#76 1

CMPLX not

HVO_0078 hemC hydroxymethylbilane synthase (porphobilinogen deaminase)

HVO_0077 sirA uroporphyrin-III C-methyltransferase

//

>NGHB#78 75

CMPLX ---

HVO_0079 - conserved hypothetical protein

HVO_0080 - conserved hypothetical protein

//

>NGHB#80 142

CMPLX not

HVO_0082 - Abi/CAAX domain protein

HVO_0081 hemL glutamate-1-semialdehyde 2,1-aminomutase

//

>NGHB#81 192

CMPLX not

HVO_0083 - GlnK-type ammonia transport regulator

HVO_0082 - Abi/CAAX domain protein

//

>NGHB#82 3

CMPLX not

HVO_0084 amt1 transport protein (probable substrate ammonium)

HVO_0083 - GlnK-type ammonia transport regulator

//

>NGHB#83 379

CMPLX not

HVO_0085 - GlnK-type ammonia transport regulator

HVO_0084 amt1 transport protein (probable substrate ammonium)

//

>NGHB#84 -4

CMPLX not

HVO_0086 amt2 transport protein (probable substrate ammonium)

HVO_0085 - GlnK-type ammonia transport regulator

//

>NGHB#85 358

CMPLX not

HVO_0087 hemB porphobilinogen synthase

HVO_0086 amt2 transport protein (probable substrate ammonium)

//

>NGHB#87 75

CMPLX ---

HVO_0091 - PHP domain protein

HVO_0090 - conserved hypothetical protein

//

>NGHB#92 103

CMPLX ---

HVO_0096 - arNOG08307 family NADH-binding domain protein

HVO_0095 - conserved hypothetical protein

//

>NGHB#93 124

CMPLX ---

HVO_0097 - conserved hypothetical protein

HVO_0096 - arNOG08307 family NADH-binding domain protein

//

>NGHB#95 81

CMPLX not

HVO_0098 - small CPxCG-related zinc finger protein

HVO_0099 - helicase domain protein

//

>NGHB#96 404

CMPLX ---

HVO_0099 - helicase domain protein

HVO_0099_A - conserved hypothetical protein

//

>NGHB#98 77

CMPLX not

HVO_0101 gptA1 probable phosphoribosyltransferase

HVO_0100 - NMD3 family protein

//

>NGHB#100 6

CMPLX ---

HVO_0102 htpX1 HtpX-like protease

HVO_0103 - conserved hypothetical protein

//

>NGHB#101 258

CMPLX ---

HVO_0103 - conserved hypothetical protein

HVO_0104 radA DNA repair and recombination protein RadA

//

>NGHB#102 250

CMPLX not

HVO_0104 radA DNA repair and recombination protein RadA

HVO_0105 - FAD-dependent oxidoreductase

//

>NGHB#103 10

CMPLX not

HVO_0105 - FAD-dependent oxidoreductase

HVO_0106 - DUF1641 domain protein

//

>NGHB#105 72

CMPLX not

HVO_0108 mscS8 mechanosensitive channel protein MscS

HVO_0107 iscU iron-sulfur cluster assembly protein

//

>NGHB#106 73

CMPLX not

HVO_0109 sufS1 cysteine desulfurase

HVO_0108 mscS8 mechanosensitive channel protein MscS

//

>NGHB#109 0

CMPLX not

HVO_0112 - tetratricopeptide repeat protein

HVO_0111 - DUF424 family protein

//

>NGHB#111 26

CMPLX not

HVO_0113 - probable phosphatase (histidine phosphatase family protein)

HVO_0114 thpR RNA 2',3'-cyclic 3'-phosphodiesterase

//

>NGHB#112 150

CMPLX not

HVO_0114 thpR RNA 2',3'-cyclic 3'-phosphodiesterase

HVO_0115 rpl39e 50S ribosomal protein L39e

//

>NGHB#113 3

CMPLX yes

HVO_0115 rpl39e 50S ribosomal protein L39e

HVO_0116 rpl31e 50S ribosomal protein L31e

//

>NGHB#114 3

CMPLX not

HVO_0116 rpl31e 50S ribosomal protein L31e

HVO_0117 tif6 translation initiation factor aIF-6

//

>NGHB#115 81

CMPLX not

HVO_0117 tif6 translation initiation factor aIF-6

HVO_0118 rpl20e, rplX 50S ribosomal protein L20e

//

>NGHB#116 -4

CMPLX not

HVO_0118 rpl20e, rplX 50S ribosomal protein L20e

HVO_0119 pfdA prefoldin alpha subunit

//

>NGHB#117 32

CMPLX not

HVO_0119 pfdA prefoldin alpha subunit

HVO_0120 ftsY signal recognition particle receptor FtsY

//

>NGHB#119 33

CMPLX not

HVO_0122 serA1 probable D-2-hydroxyacid dehydrogenase

HVO_0121 - LysE family transport protein

//

>NGHB#121 109

CMPLX not

HVO_0123 srp54 signal recognition particle 54K protein

HVO_0124 - MgtE family transport protein

//

>NGHB#122 0

CMPLX yes

HVO_0124 - MgtE family transport protein

HVO_0125 - MgtE family transport protein

//

>NGHB#123 74

CMPLX ---

HVO_0125 - MgtE family transport protein

HVO_0126 - conserved hypothetical protein

//

>NGHB#125 2

CMPLX not

HVO_0128 - UPF0200 family protein

HVO_0127 - UPF0201 family protein

//

>NGHB#127 530

CMPLX ---

HVO_0129 - DUF307 family protein

HVO_0131 - conserved hypothetical protein

//

>NGHB#128 69

CMPLX ---

HVO_0132 - conserved hypothetical protein

HVO_0131_B - small CPxCG-related zinc finger protein

//

>NGHB#129 305

CMPLX ---

HVO_0133 ths1, cct1 thermosome subunit 1

HVO_0132 - conserved hypothetical protein

//

>NGHB#130 222

CMPLX not

HVO_0134 dim2 probable ribosome biogenesis protein Dim2

HVO_0133 ths1, cct1 thermosome subunit 1

//

>NGHB#131 93

CMPLX not

HVO_0135 rio1, pkn2 RIO-type serine/threonine protein kinase Rio1

HVO_0134 dim2 probable ribosome biogenesis protein Dim2

//

>NGHB#132 101

CMPLX not

HVO_0136 tif1A1 translation initiation factor aIF-1A

HVO_0135 rio1, pkn2 RIO-type serine/threonine protein kinase Rio1

//

>NGHB#135 92

CMPLX not

HVO_0138 tyrS tyrosine--tRNA ligase

HVO_0137 - alpha/beta hydrolase fold protein

//

>NGHB#139 8

CMPLX not

HVO_0141 - stomatin family protein

HVO_0142 - small CPxCG-related zinc finger protein

//

>NGHB#141 54

CMPLX not

HVO_0144 rnz ribonuclease Z

HVO_0143 phzF2 PhzF family protein

//

>NGHB#143 -1

CMPLX not

HVO_0145 rfcC replication factor C small subunit

HVO_0146 psd probable archaetidylserine decarboxylase

//

>NGHB#144 240

CMPLX not

HVO_0146 psd probable archaetidylserine decarboxylase

HVO_0147 ureB urease beta subunit

//

>NGHB#145 -4

CMPLX yes

HVO_0147 ureB urease beta subunit

HVO_0148 ureC urease alpha subunit

//

>NGHB#146 -4

CMPLX yes

HVO_0148 ureC urease alpha subunit

HVO_0149 ureA urease gamma subunit

//

>NGHB#147 -4

CMPLX yes

HVO_0149 ureA urease gamma subunit

HVO_0150 ureG urease accessory protein UreG

//

>NGHB#148 -1

CMPLX yes

HVO_0150 ureG urease accessory protein UreG

HVO_0151 ureD urease accessory protein UreD

//

>NGHB#149 -1

CMPLX yes

HVO_0151 ureD urease accessory protein UreD

HVO_0152 ureE urease accessory protein UreE

//

>NGHB#150 -4

CMPLX yes

HVO_0152 ureE urease accessory protein UreE

HVO_0153 ureF urease accessory protein UreF

//

>NGHB#151 89

CMPLX ---

HVO_0153 ureF urease accessory protein UreF

HVO_0154 - conserved hypothetical protein

//

>NGHB#153 101

CMPLX ---

HVO_0156 trmG10 tRNA (guanine(10),N(2))-dimethyltransferase

HVO_0155 - conserved hypothetical protein

//

>NGHB#155 58

CMPLX ---

HVO_0157 - conserved hypothetical protein

HVO_0158 tbp1 TATA-binding transcription initiation factor

//

>NGHB#156 79

CMPLX ---

HVO_0158 tbp1 TATA-binding transcription initiation factor

HVO_0159 - conserved hypothetical protein

//

>NGHB#157 45

CMPLX ---

HVO_0159 - conserved hypothetical protein

HVO_0160 - Abi/CAAX domain protein

//

>NGHB#161 -4

CMPLX ---

HVO_0164 - conserved hypothetical protein

HVO_0163 - ArsR family transcription regulator

//

>NGHB#167 72

CMPLX not

HVO_0169 - TrmB family transcription regulator

HVO_0170 hjc Holliday junction resolvase Hjc

//

>NGHB#175 -4

CMPLX ---

HVO_0178 - hypothetical protein

HVO_0176 cat1 transport protein (probable substrate cationic amino acids)

//

>NGHB#177 165

CMPLX not

HVO_0179 - Lrp/AsnC family transcription regulator / TrkA domain protein

HVO_0180 rlmE, ftsJ 23S rRNA (uridine-2'-O-) methyltransferase

//

>NGHB#178 149

CMPLX ---

HVO_0180 rlmE, ftsJ 23S rRNA (uridine-2'-O-) methyltransferase

HVO_0181 - conserved hypothetical protein

//

>NGHB#180 56

CMPLX ---

HVO_0183 - probable transport protein (probable substrate queuosine precursor)

HVO_0182 - conserved hypothetical protein

//

>NGHB#181 -8

CMPLX not

HVO_0184 - CopG domain protein

HVO_0183 - probable transport protein (probable substrate queuosine precursor)

//

>NGHB#182 119

CMPLX not

HVO_0185 tatCt Sec-independent protein translocase protein TatCt

HVO_0184 - CopG domain protein

//

>NGHB#184 112

CMPLX not

HVO_0186 tatCo Sec-independent protein translocase protein TatCo

HVO_0187 - small CPxCG-related zinc finger protein

//

>NGHB#189 54

CMPLX ---

HVO_0191 mutS5a DNA mismatch repair protein MutS

HVO_0193 - conserved hypothetical protein

//

>NGHB#192 60

CMPLX not

HVO_0194 orc9 Orc1-type DNA replication protein

HVO_0195 rpiA ribose-5-phosphate isomerase

//

>NGHB#195 147

CMPLX not

HVO_0197 purE2 LarB family protein

HVO_0197_A - small CPxCG-related zinc finger protein

//

>NGHB#196 94

CMPLX not

HVO_0197_A - small CPxCG-related zinc finger protein

HVO_0198 - UPF0213 family protein

//

>NGHB#197 62

CMPLX not

HVO_0198 - UPF0213 family protein

HVO_0199 pmm2 phosphohexomutase (phosphoglucomutase / phosphomannomutase)

//

>NGHB#199 83

CMPLX ---

HVO_0201 - GNAT family acetyltransferase

HVO_0200 - conserved hypothetical protein

//

>NGHB#200 8

CMPLX not

HVO_0202 samp2 ubiquitin-like modifier protein SAMP2

HVO_0201 - GNAT family acetyltransferase

//

>NGHB#203 120

CMPLX not

HVO_0205 tspO TspO family protein

HVO_0204 - sensor box histidine kinase

//

>NGHB#205 104

CMPLX not

HVO_0206 alaS1 alanine--tRNA ligase

HVO_0207 - alpha/beta hydrolase fold protein

//

>NGHB#206 114

CMPLX not

HVO_0207 - alpha/beta hydrolase fold protein

HVO_0208 guaAa2 glutamine amidotransferase (homolog to GMP synthase subunit A)

//

>NGHB#207 175

CMPLX not

HVO_0208 guaAa2 glutamine amidotransferase (homolog to GMP synthase subunit A)

HVO_0209 acd1 glutaryl-CoA dehydrogenase

//

>NGHB#208 108

CMPLX ---

HVO_0209 acd1 glutaryl-CoA dehydrogenase

HVO_0210 - conserved hypothetical protein

//

>NGHB#209 202

CMPLX ---

HVO_0210 - conserved hypothetical protein

HVO_0211 ferB1 ferredoxin (3Fe-4S)(4Fe-4S), zinc-containing

//

>NGHB#210 44

CMPLX not

HVO_0211 ferB1 ferredoxin (3Fe-4S)(4Fe-4S), zinc-containing

HVO_0212 - glyoxalase domain protein

//

>NGHB#211 122

CMPLX not

HVO_0212 - glyoxalase domain protein

HVO_0213 dpsA2 ferritin/Dps domain protein

//

>NGHB#212 191

CMPLX not

HVO_0213 dpsA2 ferritin/Dps domain protein

HVO_0214 - L-lactate dehydrogenase

//

>NGHB#214 81

CMPLX not

HVO_0216 bioN ABC-type transport system permease protein (probable substrate biotin)

HVO_0215 - iron-sulfur protein (4Fe-4S)

//

>NGHB#215 -7

CMPLX yes

HVO_0217 bioM ABC-type transport system ATP-binding protein (probable substrate biotin)

HVO_0216 bioN ABC-type transport system permease protein (probable substrate biotin)

//

>NGHB#216 0

CMPLX yes

HVO_0218 bioY biotin transport protein BioY

HVO_0217 bioM ABC-type transport system ATP-binding protein (probable substrate biotin)

//

>NGHB#218 53

CMPLX not

HVO_0219 cmi4 conditioned medium-induced protein 4

HVO_0220 mcm ATP-dependent DNA helicase MCM

//

>NGHB#219 100

CMPLX ---

HVO_0220 mcm ATP-dependent DNA helicase MCM

HVO_0221 - conserved hypothetical protein

//

>NGHB#220 150

CMPLX ---

HVO_0221 - conserved hypothetical protein

HVO_0222 - conserved hypothetical protein

//

>NGHB#221 -4

CMPLX ---

HVO_0222 - conserved hypothetical protein

HVO_0223 - conserved hypothetical protein

//

>NGHB#222 51

CMPLX ---

HVO_0223 - conserved hypothetical protein

HVO_0224 - conserved hypothetical protein

//

>NGHB#223 -4

CMPLX ---

HVO_0224 - conserved hypothetical protein

HVO_0225 minD1 MinD/ParA domain protein

//

>NGHB#227 68

CMPLX ---

HVO_0229 - conserved hypothetical protein

HVO_0228 - ParA domain protein

//

>NGHB#228 88

CMPLX ---

HVO_0230 hit1 histidine triad family protein (homolog to bis(5'-nucleosyl)-tetraphosphatase)

HVO_0229 - conserved hypothetical protein

//

>NGHB#229 288

CMPLX not

HVO_0231 udg1 uracil-DNA glycosylase

HVO_0230 hit1 histidine triad family protein (homolog to bis(5'-nucleosyl)-tetraphosphatase)

//

>NGHB#230 0

CMPLX ---

HVO_0232 - conserved hypothetical protein

HVO_0231 udg1 uracil-DNA glycosylase

//

>NGHB#231 54

CMPLX ---

HVO_0233 prsA ribose-phosphate pyrophosphokinase

HVO_0232 - conserved hypothetical protein

//

>NGHB#232 60

CMPLX ---

HVO_0234 - conserved hypothetical protein

HVO_0233 prsA ribose-phosphate pyrophosphokinase

//

>NGHB#233 96

CMPLX ---

HVO_0235 - SWIM zinc finger domain protein

HVO_0234 - conserved hypothetical protein

//

>NGHB#235 77

CMPLX not

HVO_0236 trm1 tRNA (guanine(26)-N(2))-dimethyltransferase

HVO_0237 - UPF0761 family protein

//

>NGHB#236 1

CMPLX not

HVO_0237 - UPF0761 family protein

HVO_0238 - probable PAP2-type phosphatase

//

>NGHB#240 279

CMPLX not

HVO_0242 pepB1 aminopeptidase (homolog to leucyl aminopeptidase / aminopeptidase T)

HVO_0241 - small CPxCG-related zinc finger protein

//

>NGHB#246 42

CMPLX ---

HVO_0247 - conserved hypothetical protein

HVO_0248 - transport protein (probable substrate phosphate/sulfate)

//

>NGHB#247 -4

CMPLX ---

HVO_0248 - transport protein (probable substrate phosphate/sulfate)

HVO_0249 - conserved hypothetical protein

//

>NGHB#250 -1

CMPLX not

HVO_0251 grx2 glutaredoxin

HVO_0252 bcp2 peroxiredoxin domain protein

//

>NGHB#251 93

CMPLX not

HVO_0252 bcp2 peroxiredoxin domain protein

HVO_0253 yrdC L-threonylcarbamoyladenylate synthase

//

>NGHB#254 3

CMPLX ---

HVO_0255 - polyamine aminopropyltransferase

HVO_0256 - conserved hypothetical protein

//

>NGHB#256 206

CMPLX ---

HVO_0258 - XerC/D-like integrase

HVO_0257 - conserved hypothetical protein

//

>NGHB#257 -4

CMPLX ---

HVO_0259 - hypothetical protein

HVO_0258 - XerC/D-like integrase

//

>NGHB#258 283

CMPLX ---

HVO_0259A - conserved hypothetical protein

HVO_0259 - hypothetical protein

//

>NGHB#260 11

CMPLX not

HVO_0260 - homolog to phage PhiH1 repressor protein

HVO_0261 - homolog to phage PhiH1 repressor protein

//

>NGHB#261 44

CMPLX ---

HVO_0261 - homolog to phage PhiH1 repressor protein

HVO_0262 - hypothetical protein

//

>NGHB#266 1

CMPLX ---

HVO_0265 - conserved hypothetical protein

HVO_0266 - UPF0395 family protein

//

>NGHB#269 -4

CMPLX not

HVO_0268 - homolog to virus structural protein HGPV1-VP2

HVO_0269 - homolog to virus structural protein HGPV1-VP3

//

>NGHB#270 -4

CMPLX ---

HVO_0269 - homolog to virus structural protein HGPV1-VP3

HVO_0270 - conserved hypothetical protein

//

>NGHB#271 6

CMPLX ---

HVO_0270 - conserved hypothetical protein

HVO_0271 - homolog to virus structural protein HRPV1-VP4

//

>NGHB#272 33

CMPLX not

HVO_0271 - homolog to virus structural protein HRPV1-VP4

HVO_0272 - homolog to HGPV1-ORF5

//

>NGHB#273 -7

CMPLX not

HVO_0272 - homolog to HGPV1-ORF5

HVO_0273 - homolog to HGPV1-ORF6

//

>NGHB#274 -4

CMPLX not

HVO_0273 - homolog to HGPV1-ORF6

HVO_0274 - homolog to virus protein HRPV1-VP8

//

>NGHB#275 235

CMPLX not

HVO_0274 - homolog to virus protein HRPV1-VP8

HVO_0275 - ISH5-type transposase ISHvo11

//

>NGHB#276 102

CMPLX not

HVO_0275 - ISH5-type transposase ISHvo11

HVO_0276 - ISH5-type transposase ISHvo11

//

>NGHB#277 30

CMPLX not

HVO_0276 - ISH5-type transposase ISHvo11

HVO_0276_A - homolog to HGPV1-ORF9

//

>NGHB#278 159

CMPLX ---

HVO_0276_A - homolog to HGPV1-ORF9

HVO_0277 - conserved hypothetical protein

//

>NGHB#279 326

CMPLX ---

HVO_0277 - conserved hypothetical protein

HVO_0278 - ISH18-type transposase ISHvo9

//

>NGHB#281 186

CMPLX not

HVO_0282 - DASS family transport protein

HVO_0280_A - small CPxCG-related zinc finger protein

//

>NGHB#282 71

CMPLX not

HVO_0283 ashA archaea-specific helicase AshA

HVO_0282 - DASS family transport protein

//

>NGHB#283 150

CMPLX not

HVO_0284 - cupin 2 barrel domain protein

HVO_0283 ashA archaea-specific helicase AshA

//

>NGHB#286 172

CMPLX ---

HVO_0287 - conserved hypothetical protein

HVO_0286 - ADP-ribose pyrophosphatase

//

>NGHB#289 164

CMPLX not

HVO_0291 rpap3 rpa-associated protein

HVO_0290 - CopG domain protein

//

>NGHB#290 0

CMPLX yes

HVO_0292 rpa3 replication protein A

HVO_0291 rpap3 rpa-associated protein

//

>NGHB#292 54

CMPLX ---

HVO_0293 - conserved hypothetical protein

HVO_0294 manC mannose-1-phosphate guanylyltransferase

//

>NGHB#293 41

CMPLX ---

HVO_0294 manC mannose-1-phosphate guanylyltransferase

HVO_0295 - conserved hypothetical protein

//

>NGHB#295 111

CMPLX not

HVO_0297 tfx Tfx-type DNA-binding protein

HVO_0296 - probable oxidoreductase (short-chain dehydrogenase family)

//

>NGHB#296 76

CMPLX not

HVO_0298 - TRAM domain protein

HVO_0297 tfx Tfx-type DNA-binding protein

//

>NGHB#300 130

CMPLX not

HVO_0301 - receiver/sensor box histidine kinase

HVO_0302 - DUF373 family protein

//

>NGHB#302 303

CMPLX not

HVO_0304 etfA1 electron transfer flavoprotein alpha subunit

HVO_0303 idsA2 bifunctional short chain isoprenyl diphosphate synthase

//

>NGHB#303 -4

CMPLX yes

HVO_0305 etfB1 electron transfer flavoprotein beta subunit

HVO_0304 etfA1 electron transfer flavoprotein alpha subunit

//

>NGHB#304 69

CMPLX not

HVO_0306 - probable transmembrane glycoprotein / HTH domain protein

HVO_0305 etfB1 electron transfer flavoprotein beta subunit

//

>NGHB#306 39

CMPLX ---

HVO_0307 - conserved hypothetical protein

HVO_0308 - DUF1628 domain protein

//

>NGHB#309 -14

CMPLX yes

HVO_0310 atpH A-type ATP synthase subunit H

HVO_0311 atpI A-type ATP synthase subunit I

//

>NGHB#310 174

CMPLX yes

HVO_0311 atpI A-type ATP synthase subunit I

HVO_0312 atpK A-type ATP synthase subunit K

//

>NGHB#311 21

CMPLX yes

HVO_0312 atpK A-type ATP synthase subunit K

HVO_0313 atpE A-type ATP synthase subunit E

//

>NGHB#312 -4

CMPLX yes

HVO_0313 atpE A-type ATP synthase subunit E

HVO_0314 atpC A-type ATP synthase subunit C

//

>NGHB#313 -4

CMPLX yes

HVO_0314 atpC A-type ATP synthase subunit C

HVO_0315 atpF A-type ATP synthase subunit F

//

>NGHB#314 6

CMPLX yes

HVO_0315 atpF A-type ATP synthase subunit F

HVO_0316 atpA A-type ATP synthase subunit A

//

>NGHB#315 4

CMPLX yes

HVO_0316 atpA A-type ATP synthase subunit A

HVO_0317 atpB A-type ATP synthase subunit B

//

>NGHB#316 129

CMPLX not

HVO_0317 atpB A-type ATP synthase subunit B

HVO_0318 - receiver/sensor/bat box HTH-10 family transcription regulator

//

>NGHB#317 198

CMPLX not

HVO_0318 - receiver/sensor/bat box HTH-10 family transcription regulator

HVO_0319 atpD1 A-type ATP synthase subunit D

//

>NGHB#318 81

CMPLX not

HVO_0319 atpD1 A-type ATP synthase subunit D

HVO_0320 - small CPxCG-related zinc finger protein

//

>NGHB#321 39

CMPLX ---

HVO_0322 minD4 MinD/ParA domain protein

HVO_0323 - conserved hypothetical protein

//

>NGHB#324 459

CMPLX not

HVO_0325 - small CPxCG-related zinc finger protein

HVO_0326 ribK riboflavin kinase, CTP-dependent

//

>NGHB#325 -4

CMPLX not

HVO_0326 ribK riboflavin kinase, CTP-dependent

HVO_0327 ribB 3,4-dihydroxy-2-butanone 4-phosphate synthase

//

>NGHB#329 79

CMPLX not

HVO_0331 fadM1 proline dehydrogenase

HVO_0330 - DUF502 family protein

//

>NGHB#331 66

CMPLX not

HVO_0332 carS CDP-2,3-bis-(O-geranylgeranyl)-sn-glycerol synthase

HVO_0333 pyrE2 orotate phosphoribosyltransferase

//

>NGHB#332 74

CMPLX not

HVO_0333 pyrE2 orotate phosphoribosyltransferase

HVO_0334 apt2 purine phosphoribosyltransferase (adenine phosphoribosyltransferase, xanthine-guanine phosphoribosyltransferase)

//

>NGHB#333 186

CMPLX not

HVO_0334 apt2 purine phosphoribosyltransferase (adenine phosphoribosyltransferase, xanthine-guanine phosphoribosyltransferase)

HVO_0335 uraA3 xanthine/uracil permease family transport protein

//

>NGHB#334 91

CMPLX ---

HVO_0335 uraA3 xanthine/uracil permease family transport protein

HVO_0336 - conserved hypothetical protein (nonfunctional)

//

>NGHB#335 102

CMPLX ---

HVO_0336 - conserved hypothetical protein (nonfunctional)

HVO_0337 grx1 glutaredoxin

//

>NGHB#340 20

CMPLX not

HVO_0341 - DUF87 domain protein

HVO_0342 crcB1 putative fluoride ion transport protein CrcB

//

>NGHB#341 -4

CMPLX yes

HVO_0342 crcB1 putative fluoride ion transport protein CrcB

HVO_0343 crcB2 putative fluoride ion transport protein CrcB

//

>NGHB#342 844

CMPLX not

HVO_0343 crcB2 putative fluoride ion transport protein CrcB

HVO_0346 rpoH DNA-directed RNA polymerase subunit H

//

>NGHB#343 -4

CMPLX yes

HVO_0346 rpoH DNA-directed RNA polymerase subunit H

HVO_0347 rpoB2 DNA-directed RNA polymerase subunit B''

//

>NGHB#344 1

CMPLX yes

HVO_0347 rpoB2 DNA-directed RNA polymerase subunit B''

HVO_0348 rpoB1 DNA-directed RNA polymerase subunit B'

//

>NGHB#345 6

CMPLX yes

HVO_0348 rpoB1 DNA-directed RNA polymerase subunit B'

HVO_0349 rpoA1 DNA-directed RNA polymerase subunit A'

//

>NGHB#346 -8

CMPLX yes

HVO_0349 rpoA1 DNA-directed RNA polymerase subunit A'

HVO_0350 rpoA2 DNA-directed RNA polymerase subunit A''

//

>NGHB#347 2

CMPLX not

HVO_0350 rpoA2 DNA-directed RNA polymerase subunit A''

HVO_0351 nusA transcription elongation factor NusA

//

>NGHB#348 54

CMPLX not

HVO_0351 nusA transcription elongation factor NusA

HVO_0352 mscS6 mechanosensitive channel protein MscS

//

>NGHB#349 227

CMPLX not

HVO_0352 mscS6 mechanosensitive channel protein MscS

HVO_0353 rps12 30S ribosomal protein S12

//

>NGHB#350 2

CMPLX yes

HVO_0353 rps12 30S ribosomal protein S12

HVO_0354 rps7 30S ribosomal protein S7

//

>NGHB#353 297

CMPLX not

HVO_0356 tef2 translation elongation factor aEF-2

HVO_0357 - ACT domain protein

//

>NGHB#354 -4

CMPLX not

HVO_0357 - ACT domain protein

HVO_0358 hom1 homoserine dehydrogenase

//

>NGHB#355 190

CMPLX not

HVO_0358 hom1 homoserine dehydrogenase

HVO_0359 tef1a1 translation elongation factor aEF-1 alpha / peptide chain release factor aRF-3

//

>NGHB#356 2

CMPLX not

HVO_0359 tef1a1 translation elongation factor aEF-1 alpha / peptide chain release factor aRF-3

HVO_0360 rps10a 30S ribosomal protein S10a

//

>NGHB#357 603

CMPLX yes

HVO_0360 rps10a 30S ribosomal protein S10a

HVO_0362 - SWIM zinc finger domain protein

//

>NGHB#358 184

CMPLX ---

HVO_0362 - SWIM zinc finger domain protein

HVO_0364 - conserved hypothetical protein

//

>NGHB#360 -11

CMPLX ---

HVO_0365 - conserved hypothetical protein

HVO_0363 - conserved hypothetical protein

//

>NGHB#361 -8

CMPLX ---

HVO_0366 - conserved hypothetical protein

HVO_0365 - conserved hypothetical protein

//

>NGHB#362 1

CMPLX ---

HVO_0367 - conserved hypothetical protein

HVO_0366 - conserved hypothetical protein

//

>NGHB#363 -4

CMPLX ---

HVO_0368 - conserved hypothetical protein

HVO_0367 - conserved hypothetical protein

//

>NGHB#364 -1

CMPLX ---

HVO_0369 - hypothetical protein

HVO_0368 - conserved hypothetical protein

//

>NGHB#365 -1

CMPLX ---

HVO_0370 - hypothetical protein

HVO_0369 - hypothetical protein

//

>NGHB#366 -1

CMPLX ---

HVO_0371 - conserved hypothetical protein

HVO_0370 - hypothetical protein

//

>NGHB#367 10

CMPLX ---

HVO_0372 - conserved hypothetical protein

HVO_0371 - conserved hypothetical protein

//

>NGHB#368 3

CMPLX ---

HVO_0373 - conserved hypothetical protein

HVO_0372 - conserved hypothetical protein

//

>NGHB#370 -4

CMPLX ---

HVO_0374 - conserved hypothetical protein

HVO_0375 - small CPxCG-related zinc finger protein

//

>NGHB#371 -8

CMPLX ---

HVO_0375 - small CPxCG-related zinc finger protein

HVO_0376 - conserved hypothetical protein

//

>NGHB#372 -4

CMPLX ---

HVO_0376 - conserved hypothetical protein

HVO_0377 - conserved hypothetical protein

//

>NGHB#373 -4

CMPLX ---

HVO_0377 - conserved hypothetical protein

HVO_0378 - conserved hypothetical protein

//

>NGHB#374 568

CMPLX ---

HVO_0378 - conserved hypothetical protein

HVO_0379 - integrase family protein

//

>NGHB#376 -11

CMPLX ---

HVO_0379_B - hypothetical protein

HVO_0379_A - conserved hypothetical protein

//

>NGHB#377 367

CMPLX ---

HVO_0380 - conserved hypothetical protein

HVO_0379_B - hypothetical protein

//

>NGHB#379 109

CMPLX ---

HVO_0381 - CopG domain protein

HVO_0382 - conserved hypothetical protein

//

>NGHB#380 -4

CMPLX ---

HVO_0382 - conserved hypothetical protein

HVO_0383 - HTH domain protein

//

>NGHB#381 -4

CMPLX ---

HVO_0383 - HTH domain protein

HVO_0384 - conserved hypothetical protein

//

>NGHB#382 -4

CMPLX ---

HVO_0384 - conserved hypothetical protein

HVO_0385 - XerC/D-like integrase

//

>NGHB#385 96

CMPLX ---

HVO_0386 - conserved hypothetical protein

HVO_0387 - conserved hypothetical protein

//

>NGHB#387 120

CMPLX not

HVO_0389 - small CPxCG-related zinc finger protein

HVO_0388 rnr, vacB ribonuclease R

//

>NGHB#391 119

CMPLX not

HVO_0392 sepF probable SepF protein

HVO_0393 uvrA UvrABC system protein A

//

>NGHB#392 137

CMPLX ---

HVO_0393 uvrA UvrABC system protein A

HVO_0394 - conserved hypothetical protein

//

>NGHB#395 75

CMPLX not

HVO_0395 - WD40/YVTN domain protein

HVO_0396 - FAD-dependent oxidoreductase (homolog to geranylgeranyl reductase)

//

>NGHB#397 165

CMPLX ---

HVO_0398 - conserved hypothetical protein

HVO_0397 - homolog to glycine cleavage system protein T

//

>NGHB#399 70

CMPLX ---

HVO_0399 - conserved hypothetical protein

HVO_0400 - conserved hypothetical protein

//

>NGHB#400 192

CMPLX ---

HVO_0400 - conserved hypothetical protein

HVO_0401 - UspA domain protein

//

>NGHB#401 77

CMPLX not

HVO_0401 - UspA domain protein

HVO_0402 - DUF124 family protein

//

>NGHB#402 84

CMPLX not

HVO_0402 - DUF124 family protein

HVO_0403 glcK glucokinase

//

>NGHB#403 446

CMPLX not

HVO_0403 glcK glucokinase

HVO_0405 - NifU C-terminal domain protein

//

>NGHB#405 -4

CMPLX ---

HVO_0407 - conserved hypothetical protein

HVO_0406 aubA probable ribonuclease FAU-1

//

>NGHB#412 6

CMPLX not

HVO_0415 uvrD repair helicase UvrD

HVO_0414 - peptidase M24 family protein

//

>NGHB#413 158

CMPLX not

HVO_0416 - small CPxCG-related zinc finger protein

HVO_0415 uvrD repair helicase UvrD

//

>NGHB#414 91

CMPLX not

HVO_0417 cxp metal-dependent carboxypeptidase

HVO_0416 - small CPxCG-related zinc finger protein

//

>NGHB#415 194

CMPLX not

HVO_0418 - homing endonuclease domain protein

HVO_0417 cxp metal-dependent carboxypeptidase

//

>NGHB#416 106

CMPLX not

HVO_0419 - M20 family amidohydrolase (homolog to succinyl-diaminopimelate desuccinylase)

HVO_0418 - homing endonuclease domain protein

//

>NGHB#418 117

CMPLX not

HVO_0420 mpcT, htr14 transducer protein MpcT

HVO_0421 - arNOG04375 family protein (homolog to PilT-type ATPase)

//

>NGHB#421 2

CMPLX ---

HVO_0423 - conserved hypothetical protein

HVO_0424 - ABCE1 family ribosome recycling factor

//

>NGHB#422 136

CMPLX not

HVO_0424 - ABCE1 family ribosome recycling factor

HVO_0425 - glyoxalase domain protein (homolog to catechol-2,3-dioxygenase)

//

>NGHB#423 54

CMPLX not

HVO_0425 - glyoxalase domain protein (homolog to catechol-2,3-dioxygenase)

HVO_0426 - UPF0098 family protein

//

>NGHB#425 0

CMPLX not

HVO_0428 - UspA domain protein

HVO_0427 rtcA RNA 3'-terminal phosphate cyclase

//

>NGHB#426 81

CMPLX not

HVO_0429 amzA archaemetzincin

HVO_0428 - UspA domain protein

//

>NGHB#428 31

CMPLX not

HVO_0430 - UPF0146 family protein

HVO_0431 - HAD superfamily hydrolase

//

>NGHB#429 49

CMPLX ---

HVO_0431 - HAD superfamily hydrolase

HVO_0432 - conserved hypothetical protein

//

>NGHB#430 91

CMPLX ---

HVO_0432 - conserved hypothetical protein

HVO_0433 npdG F420H2:NADP oxidoreductase

//

>NGHB#432 234

CMPLX not

HVO_0435 hisE phosphoribosyl-ATP pyrophosphatase

HVO_0434 - major facilitator superfamily transport protein

//

>NGHB#433 78

CMPLX not

HVO_0436 pdxT pyridoxal 5'-phosphate synthase subunit PdxT

HVO_0435 hisE phosphoribosyl-ATP pyrophosphatase

//

>NGHB#434 55

CMPLX not

HVO_0437 secG, sec61b protein translocase subunit SecG

HVO_0436 pdxT pyridoxal 5'-phosphate synthase subunit PdxT

//

>NGHB#436 113

CMPLX not

HVO_0438 trxA1 thioredoxin

HVO_0439 rpl40e 50S ribosomal protein L40e

//

>NGHB#438 1

CMPLX ---

HVO_0441 - beta-lactamase domain protein

HVO_0440 - conserved hypothetical protein

//

>NGHB#440 -4

CMPLX ---

HVO_0442 - conserved hypothetical protein

HVO_0443 - UPF0215 family protein

//

>NGHB#441 24

CMPLX not

HVO_0443 - UPF0215 family protein

HVO_0444 udg3 uracil-DNA glycosylase

//

>NGHB#443 -11

CMPLX yes

HVO_0446 phnC1 ABC-type transport system ATP-binding protein (probable substrate phosphate/phosphonate)

HVO_0445 phnE1 ABC-type transport system permease protein (probable substrate phosphate/phosphonate)

//

>NGHB#444 95

CMPLX yes

HVO_0447 phnD1 ABC-type transport system periplasmic substrate-binding protein (probable substrate phosphate/phosphonate)

HVO_0446 phnC1 ABC-type transport system ATP-binding protein (probable substrate phosphate/phosphonate)

//

>NGHB#446 54

CMPLX not

HVO_0448 hisH imidazole glycerol-phosphate synthase subunit HisH

HVO_0449 pheA prephenate dehydratase

//

>NGHB#447 143

CMPLX not

HVO_0449 pheA prephenate dehydratase

HVO_0450 hsp20A Hsp20-type molecular chaperone

//

>NGHB#448 116

CMPLX not

HVO_0450 hsp20A Hsp20-type molecular chaperone

HVO_0451 hsp20B Hsp20-type molecular chaperone

//

>NGHB#449 214

CMPLX not

HVO_0451 hsp20B Hsp20-type molecular chaperone

HVO_0452 leuS leucine--tRNA ligase

//

>NGHB#450 123

CMPLX ---

HVO_0452 leuS leucine--tRNA ligase

HVO_0453 - conserved hypothetical protein

//

>NGHB#452 298

CMPLX not

HVO_0455 ths2, cct2 thermosome subunit 2

HVO_0454 ala alanine dehydrogenase

//

>NGHB#453 185

CMPLX ---

HVO_0456 - conserved hypothetical protein

HVO_0455 ths2, cct2 thermosome subunit 2

//

>NGHB#454 63

CMPLX ---

HVO_0457 - conserved hypothetical protein

HVO_0456 - conserved hypothetical protein

//

>NGHB#455 655

CMPLX ---

HVO_0458A - conserved hypothetical protein

HVO_0457 - conserved hypothetical protein

//

>NGHB#456 1

CMPLX ---

HVO_0459 ridA1 enamine/imine deaminase

HVO_0458A - conserved hypothetical protein

//

>NGHB#457 84

CMPLX ---

HVO_0460 - conserved hypothetical protein

HVO_0459 ridA1 enamine/imine deaminase

//

>NGHB#458 39

CMPLX ---

HVO_0461 cydB cytochrome bd ubiquinol oxidase subunit II

HVO_0460 - conserved hypothetical protein

//

>NGHB#459 -8

CMPLX yes

HVO_0462 cydA cytochrome bd ubiquinol oxidase subunit I

HVO_0461 cydB cytochrome bd ubiquinol oxidase subunit II

//

>NGHB#467 96

CMPLX not

HVO_0469 - AstE domain protein

HVO_0470 thrC1 threonine synthase

//

>NGHB#469 125

CMPLX ---

HVO_0472 - DUF2892 family protein

HVO_0471 - conserved hypothetical protein

//

>NGHB#472 21

CMPLX not

HVO_0475 - probable S-adenosylmethionine-dependent methyltransferase

HVO_0474 pimT protein-L-isoaspartate O-methyltransferase

//

>NGHB#473 96

CMPLX not

HVO_0476 - UCP015877 family protein

HVO_0475 - probable S-adenosylmethionine-dependent methyltransferase

//

>NGHB#474 138

CMPLX not

HVO_0477 pepB3 aminopeptidase (homolog to leucyl aminopeptidase / aminopeptidase T)

HVO_0476 - UCP015877 family protein

//

>NGHB#479 75

CMPLX not

HVO_0481 gap2 glyceraldehyde-3-phosphate dehydrogenase (NAD) (phosphorylating)

HVO_0480 pgk phosphoglycerate kinase

//

>NGHB#482 85

CMPLX not

HVO_0484 rpl16 50S ribosomal protein L16

HVO_0483 - ATP-grasp fold protein

//

>NGHB#486 128

CMPLX not

HVO_0487 - FMN-binding domain protein

HVO_0488 - HTH domain protein

//

>NGHB#487 70

CMPLX not

HVO_0488 - HTH domain protein

HVO_0489 - small CPxCG-related zinc finger protein

//

>NGHB#488 124

CMPLX not

HVO_0489 - small CPxCG-related zinc finger protein

HVO_0490 - small CPxCG-related zinc finger protein

//

>NGHB#489 117

CMPLX not

HVO_0490 - small CPxCG-related zinc finger protein

HVO_0491 - FMN-binding domain protein

//

>NGHB#490 102

CMPLX not

HVO_0491 - FMN-binding domain protein

HVO_0492 boa1 HTH-10 family transcription regulator

//

>NGHB#491 114

CMPLX not

HVO_0492 boa1 HTH-10 family transcription regulator

HVO_0493 - HTH domain protein

//

>NGHB#492 117

CMPLX ---

HVO_0493 - HTH domain protein

HVO_0494 - conserved hypothetical protein

//

>NGHB#494 169

CMPLX not

HVO_0497 cspA1 cold shock protein

HVO_0496 - DUF2270 family protein

//

>NGHB#495 159

CMPLX not

HVO_0498 cspA2, cmi9 cold shock protein

HVO_0497 cspA1 cold shock protein

//

>NGHB#496 565

CMPLX ---

HVO_0500 - conserved hypothetical protein

HVO_0498 cspA2, cmi9 cold shock protein

//

>NGHB#497 45

CMPLX ---

HVO_0501 - VanZ family protein

HVO_0500 - conserved hypothetical protein

//

>NGHB#499 394

CMPLX ---

HVO_0502 - conserved hypothetical protein

HVO_0504 - DUF192 family protein

//

>NGHB#500 40

CMPLX ---

HVO_0504 - DUF192 family protein

HVO_0505 - conserved hypothetical protein

//

>NGHB#501 104

CMPLX ---

HVO_0505 - conserved hypothetical protein

HVO_0506 - ABC-type transport system ATP-binding/permease protein

//

>NGHB#502 43

CMPLX not

HVO_0506 - ABC-type transport system ATP-binding/permease protein

HVO_0507 cre1 creatininase domain protein

//

>NGHB#504 88

CMPLX ---

HVO_0509 - NUDIX family hydrolase

HVO_0508 - conserved hypothetical protein

//

>NGHB#505 -4

CMPLX not

HVO_0510 mptD dihydroneopterin aldolase, archaeal-type

HVO_0509 - NUDIX family hydrolase

//

>NGHB#511 164

CMPLX not

HVO_0515 - small CPxCG-related zinc finger protein

HVO_0516 - SGNH-type esterase (homolog to rhamnogalacturonan acetylesterase)

//

>NGHB#512 393

CMPLX ---

HVO_0516 - SGNH-type esterase (homolog to rhamnogalacturonan acetylesterase)

HVO_0518 - conserved hypothetical protein

//

>NGHB#513 188

CMPLX ---

HVO_0518 - conserved hypothetical protein

HVO_0519 rpa2 replication protein A

//

>NGHB#514 192

CMPLX not

HVO_0519 rpa2 replication protein A

HVO_0520 hstA archaeal histone

//

>NGHB#515 -1

CMPLX not

HVO_0520 hstA archaeal histone

HVO_0522 hda1, hdal HdaI-type histone deacetylase

//

>NGHB#518 244

CMPLX ---

HVO_0528 - ISH3-type transposase ISH51

HVO_0527 - conserved hypothetical protein (nonfunctional)

//

>NGHB#520 81

CMPLX not

HVO_0529_A - homolog to pHK2-ORF2

HVO_0530 tsgA1 ABC-type transport system periplasmic substrate-binding protein (probable substrate sugar)

//

>NGHB#521 10

CMPLX yes

HVO_0530 tsgA1 ABC-type transport system periplasmic substrate-binding protein (probable substrate sugar)

HVO_0531 tsgB1 ABC-type transport system permease protein (probable substrate sugar)

//

>NGHB#522 -4

CMPLX yes

HVO_0531 tsgB1 ABC-type transport system permease protein (probable substrate sugar)

HVO_0532 tsgC1 ABC-type transport system permease protein (probable substrate sugar)

//

>NGHB#523 120

CMPLX yes

HVO_0532 tsgC1 ABC-type transport system permease protein (probable substrate sugar)

HVO_0534 tsgD1 ABC-type transport system ATP-binding protein (probable substrate sugar)

//

>NGHB#527 108

CMPLX ---

HVO_0538 idr2 SirR/DtxR family transcription regulator Idr2

HVO_0537 - conserved hypothetical protein

//

>NGHB#528 -4

CMPLX ---

HVO_0539 - conserved hypothetical protein

HVO_0538 idr2 SirR/DtxR family transcription regulator Idr2

//

>NGHB#529 197

CMPLX ---

HVO_0540 - conserved hypothetical protein

HVO_0539 - conserved hypothetical protein

//

>NGHB#530 158

CMPLX ---

HVO_0541 citB2, acnA aconitate hydratase

HVO_0540 - conserved hypothetical protein

//

>NGHB#531 130

CMPLX ---

HVO_0542 - conserved hypothetical protein

HVO_0541 citB2, acnA aconitate hydratase

//

>NGHB#532 98

CMPLX ---

HVO_0543 trxA8 thioredoxin

HVO_0542 - conserved hypothetical protein

//

>NGHB#534 319

CMPLX ---

HVO_0544 - major facilitator superfamily transport protein

HVO_0545 - conserved hypothetical protein

//

>NGHB#535 110

CMPLX ---

HVO_0545 - conserved hypothetical protein

HVO_0546 - small CPxCG-related zinc finger protein

//

>NGHB#537 127

CMPLX ---

HVO_0548 nhaC1 probable NhaC-type sodium/proton antiporter

HVO_0547 - conserved hypothetical protein

//

>NGHB#538 136

CMPLX not

HVO_0549 kdgK1 2-keto-3-deoxygluconate kinase / 2-keto-3-deoxygalactonate kinase

HVO_0548 nhaC1 probable NhaC-type sodium/proton antiporter

//

>NGHB#539 88

CMPLX ---

HVO_0550 - conserved hypothetical protein

HVO_0549 kdgK1 2-keto-3-deoxygluconate kinase / 2-keto-3-deoxygalactonate kinase

//

>NGHB#540 133

CMPLX ---

HVO_0551 mutLb DNA mismatch repair protein MutL

HVO_0550 - conserved hypothetical protein

//

>NGHB#541 -4

CMPLX yes

HVO_0552 mutS1b DNA mismatch repair protein MutS

HVO_0551 mutLb DNA mismatch repair protein MutL

//

>NGHB#543 -4

CMPLX yes

HVO_0553 basB chemotactic signal transduction system periplasmic substrate-binding protein BasB

HVO_0554 basT, htr3 transducer protein BasT

//

>NGHB#544 206

CMPLX not

HVO_0554 basT, htr3 transducer protein BasT

HVO_0555 htr15a transducer protein Htr15

//

>NGHB#545 296

CMPLX not

HVO_0555 htr15a transducer protein Htr15

HVO_0556 - ISH18-type transposase ISHvo10 (nonfunctional)

//

>NGHB#549 106

CMPLX ---

HVO_0561 rpl15e 50S ribosomal protein L15e

HVO_0560 - conserved hypothetical protein

//

>NGHB#550 228

CMPLX not

HVO_0562 malG ABC-type transport system permease protein (probable substrate maltose)

HVO_0561 rpl15e 50S ribosomal protein L15e

//

>NGHB#551 -4

CMPLX yes

HVO_0563 malF ABC-type transport system permease protein (probable substrate maltose)

HVO_0562 malG ABC-type transport system permease protein (probable substrate maltose)

//

>NGHB#552 5

CMPLX yes

HVO_0564 malE ABC-type transport system periplasmic substrate-binding protein (probable substrate maltose)

HVO_0563 malF ABC-type transport system permease protein (probable substrate maltose)

//

>NGHB#556 26

CMPLX not

HVO_0567 amyA1 glycoside hydrolase domain protein

HVO_0568 - TrmB family transcription regulator

//

>NGHB#558 535

CMPLX not

HVO_0570 - histidine kinase

HVO_0569 rio2, pkn1 RIO-type serine/threonine protein kinase Rio2

//

>NGHB#559 48

CMPLX ---

HVO_0571 - conserved hypothetical protein

HVO_0570 - histidine kinase

//

>NGHB#561 54

CMPLX not

HVO_0572 lpl lipoate--protein ligase domain protein

HVO_0573 apn1 endonuclease 4

//

>NGHB#562 81

CMPLX not

HVO_0573 apn1 endonuclease 4

HVO_0574 - probable S-adenosylmethionine-dependent methyltransferase

//

>NGHB#570 2

CMPLX not

HVO_0582 - CopG domain protein

HVO_0581 ftsZ2 cell division protein FtsZ, type II

//

>NGHB#571 212

CMPLX not

HVO_0583 - CopG/DZR domain protein

HVO_0582 - CopG domain protein

//

>NGHB#573 65

CMPLX not

HVO_0585 - probable oxidoreductase (aldo-keto reductase family protein)

HVO_0586 - HAD superfamily hydrolase

//

>NGHB#574 -10

CMPLX not

HVO_0586 - HAD superfamily hydrolase

HVO_0587 cbiB adenosylcobinamide-phosphate synthase

//

>NGHB#575 -10

CMPLX not

HVO_0587 cbiB adenosylcobinamide-phosphate synthase

HVO_0588 cobS adenosylcobinamide-GDP ribazoletransferase

//

>NGHB#576 -1

CMPLX not

HVO_0588 cobS adenosylcobinamide-GDP ribazoletransferase

HVO_0589 cobY adenosylcobinamide-phosphate guanylyltransferase

//

>NGHB#577 -10

CMPLX not

HVO_0589 cobY adenosylcobinamide-phosphate guanylyltransferase

HVO_0590 cobT probable nicotinate-nucleotide-dimethylbenzimidazole phosphoribosyltransferase

//

>NGHB#578 -11

CMPLX not

HVO_0590 cobT probable nicotinate-nucleotide-dimethylbenzimidazole phosphoribosyltransferase

HVO_0591 cobD1 L-threonine-O-3-phosphate decarboxylase

//

>NGHB#579 -8

CMPLX not

HVO_0591 cobD1 L-threonine-O-3-phosphate decarboxylase

HVO_0592 cbiZ adenosylcobinamide amidohydrolase

//

>NGHB#580 99

CMPLX not

HVO_0592 cbiZ adenosylcobinamide amidohydrolase

HVO_0593 cobD2 L-threonine-O-3-phosphate decarboxylase

//

>NGHB#583 -4

CMPLX ---

HVO_0595 minD2 MinD/ParA domain protein

HVO_0596 - conserved hypothetical protein

//

>NGHB#584 64

CMPLX ---

HVO_0596 - conserved hypothetical protein

HVO_0597 - OsmC domain protein

//

>NGHB#585 107

CMPLX ---

HVO_0597 - OsmC domain protein

HVO_0598 - conserved hypothetical protein

//

>NGHB#588 -4

CMPLX not

HVO_0600 - ACT domain protein

HVO_0601 hom2 homoserine dehydrogenase

//

>NGHB#590 541

CMPLX not

HVO_0603 aroD2 3-dehydroquinate dehydratase

HVO_0602 aroD1 3-dehydroquinate dehydratase

//

>NGHB#593 221

CMPLX not

HVO_0606 - MenG family protein

HVO_0605 - IclR family transcription regulator

//

>NGHB#594 204

CMPLX not

HVO_0607 - Bug domain protein

HVO_0606 - MenG family protein

//

>NGHB#598 -1

CMPLX not

HVO_0610 - DUF1468 family protein

HVO_0611 - DUF112 family protein

//

>NGHB#599 535

CMPLX not

HVO_0611 - DUF112 family protein

HVO_0612 - UspA domain protein

//

>NGHB#600 134

CMPLX not

HVO_0612 - UspA domain protein

HVO_0613 - beta-lactamase domain protein

//

>NGHB#602 -4

CMPLX ---

HVO_0615 - conserved hypothetical protein

HVO_0614 - conserved hypothetical protein

//

>NGHB#603 -8

CMPLX ---

HVO_0616 - conserved hypothetical protein

HVO_0615 - conserved hypothetical protein

//

>NGHB#604 -8

CMPLX ---

HVO_0617 - conserved hypothetical protein

HVO_0616 - conserved hypothetical protein

//

>NGHB#605 -8

CMPLX ---

HVO_0618 - probable secreted glycoprotein

HVO_0617 - conserved hypothetical protein

//

>NGHB#606 -4

CMPLX not

HVO_0619 pilC1 type IV pilus biogenesis complex membrane subunit

HVO_0618 - probable secreted glycoprotein

//

>NGHB#607 -4

CMPLX yes

HVO_0620 pilB1 type IV pilus biogenesis complex ATPase subunit

HVO_0619 pilC1 type IV pilus biogenesis complex membrane subunit

//

>NGHB#608 221

CMPLX not

HVO_0621 - sensor box histidine kinase

HVO_0620 pilB1 type IV pilus biogenesis complex ATPase subunit

//

>NGHB#609 153

CMPLX not

HVO_0622 ubiA3 UbiA family prenyltransferase

HVO_0621 - sensor box histidine kinase

//

>NGHB#611 97

CMPLX ---

HVO_0623 - MoaD family protein

HVO_0624 - conserved hypothetical protein

//

>NGHB#613 108

CMPLX ---

HVO_0626 - conserved hypothetical protein

HVO_0625 - DMT superfamily transport protein

//

>NGHB#615 147

CMPLX yes

HVO_0627 dppDF2 ABC-type transport system ATP-binding protein (probable substrate dipeptide/oligopeptide)

HVO_0628 dppA2 ABC-type transport system periplasmic substrate-binding protein (probable substrate dipeptide/oligopeptide)

//

>NGHB#616 25

CMPLX yes

HVO_0628 dppA2 ABC-type transport system periplasmic substrate-binding protein (probable substrate dipeptide/oligopeptide)

HVO_0629 dppB2 ABC-type transport system permease protein (probable substrate dipeptide/oligopeptide)

//

>NGHB#617 -4

CMPLX yes

HVO_0629 dppB2 ABC-type transport system permease protein (probable substrate dipeptide/oligopeptide)

HVO_0630 dppC2 ABC-type transport system permease protein (probable substrate dipeptide/oligopeptide)

//

>NGHB#618 6

CMPLX ---

HVO_0630 dppC2 ABC-type transport system permease protein (probable substrate dipeptide/oligopeptide)

HVO_0631 - conserved hypothetical protein

//

>NGHB#620 -4

CMPLX not

HVO_0633 cirC KaiC-type circadian clock protein CirC

HVO_0632 - sensor box histidine kinase

//

>NGHB#621 152

CMPLX not

HVO_0634 orc2 Orc1-type DNA replication protein

HVO_0633 cirC KaiC-type circadian clock protein CirC

//

>NGHB#623 -4

CMPLX ---

HVO_0635 - DUF4129 domain protein

HVO_0636 - conserved hypothetical protein

//

>NGHB#624 -4

CMPLX ---

HVO_0636 - conserved hypothetical protein

HVO_0637 - DUF11/DUF58 family protein

//

>NGHB#625 -13

CMPLX ---

HVO_0637 - DUF11/DUF58 family protein

HVO_0638 - conserved hypothetical protein

//

>NGHB#627 474

CMPLX not

HVO_0641 bluB 5,6-dimethylbenzimidazole synthase BluB

HVO_0639 moxR1 AAA-type ATPase (MoxR subfamily)

//

>NGHB#629 137

CMPLX ---

HVO_0642 - conserved hypothetical protein

HVO_0643 - conserved hypothetical protein

//

>NGHB#631 207

CMPLX not

HVO_0645 - DUF192 family protein

HVO_0644 leuA1, cimA 2-isopropylmalate synthase / (R)-citramalate synthase

//

>NGHB#633 100

CMPLX ---

HVO_0646 - conserved hypothetical protein

HVO_0647 guaAa1 GMP synthase (glutamine-hydrolyzing) subunit A

//

>NGHB#634 176

CMPLX not

HVO_0647 guaAa1 GMP synthase (glutamine-hydrolyzing) subunit A

HVO_0648 - DUF2070 family protein

//

>NGHB#636 180

CMPLX not

HVO_0650 - DUF3194 family protein

HVO_0649 - small CPxCG-related zinc finger protein

//

>NGHB#637 0

CMPLX not

HVO_0651 pfdB prefoldin beta subunit

HVO_0650 - DUF3194 family protein

//

>NGHB#638 43

CMPLX not

HVO_0652 pcc1 KEOPS complex subunit Pcc1

HVO_0651 pfdB prefoldin beta subunit

//

>NGHB#639 1

CMPLX not

HVO_0653 rpoP DNA-directed RNA polymerase subunit P

HVO_0652 pcc1 KEOPS complex subunit Pcc1

//

>NGHB#640 34

CMPLX not

HVO_0654 rpl43e 50S ribosomal protein L43e

HVO_0653 rpoP DNA-directed RNA polymerase subunit P

//

>NGHB#645 -1

CMPLX not

HVO_0659 pth peptidyl-tRNA hydrolase

HVO_0658 truD tRNA pseudouridine(13) synthase TruD

//

>NGHB#647 129

CMPLX not

HVO_0660 - Yip1 domain protein

HVO_0661 dcd dCTP deaminase

//

>NGHB#648 -4

CMPLX not

HVO_0661 dcd dCTP deaminase

HVO_0662 thiR transcription regulator ThiR

//

>NGHB#649 12

CMPLX not

HVO_0662 thiR transcription regulator ThiR

HVO_0663 - probable S-adenosylmethionine-dependent methyltransferase

//

>NGHB#650 66

CMPLX ---

HVO_0663 - probable S-adenosylmethionine-dependent methyltransferase

HVO_0664 - conserved hypothetical protein

//

>NGHB#654 0

CMPLX not

HVO_0668 oadhB3 2-oxoacid dehydrogenase E1 component beta subunit

HVO_0667 - homolog to NAD kinase

//

>NGHB#655 -4

CMPLX yes

HVO_0669 oadhA3 2-oxoacid dehydrogenase E1 component alpha subunit

HVO_0668 oadhB3 2-oxoacid dehydrogenase E1 component beta subunit

//

>NGHB#657 96

CMPLX not

HVO_0670 - YqjG-type gamma-glutamylcysteinyl-hydroquinone reductase

HVO_0671 - molybdopterin-binding domain protein (nonfunctional)

//

>NGHB#659 61

CMPLX ---

HVO_0673 - conserved hypothetical protein

HVO_0672 - conserved hypothetical protein

//

>NGHB#660 46

CMPLX ---

HVO_0674 - 4-phosphopantoate--beta-alanine ligase

HVO_0673 - conserved hypothetical protein

//

>NGHB#661 55

CMPLX not

HVO_0675 - pantoate kinase

HVO_0674 - 4-phosphopantoate--beta-alanine ligase

//

>NGHB#662 54

CMPLX not

HVO_0676 - DUF2078 family protein

HVO_0675 - pantoate kinase

//

>NGHB#663 351

CMPLX not

HVO_0677 aspS aspartate--tRNA(Asp/Asn) ligase

HVO_0676 - DUF2078 family protein

//

>NGHB#665 76

CMPLX not

HVO_0678 - cupin 2 barrel domain protein (nonfunctional)

HVO_0679 - DUF4013 family protein

//

>NGHB#672 1

CMPLX ---

HVO_0685 - DUF3100 family protein

HVO_0686 - conserved hypothetical protein

//

>NGHB#673 46

CMPLX ---

HVO_0686 - conserved hypothetical protein

HVO_0687 - M20 family amidohydrolase

//

>NGHB#674 105

CMPLX ---

HVO_0687 - M20 family amidohydrolase

HVO_0688 - conserved hypothetical protein

//

>NGHB#675 83

CMPLX ---

HVO_0688 - conserved hypothetical protein

HVO_0689 smc chromosome segregation protein Smc

//

>NGHB#676 -8

CMPLX not

HVO_0689 smc chromosome segregation protein Smc

HVO_0690 scpA chromosome segregation and condensation protein ScpA

//

>NGHB#677 32

CMPLX ---

HVO_0690 scpA chromosome segregation and condensation protein ScpA

HVO_0691 - conserved hypothetical protein

//

>NGHB#679 45

CMPLX not

HVO_0693 - auxin permease family transport protein

HVO_0692 - probable 6-oxopurine nucleoside phosphorylase

//

>NGHB#680 162

CMPLX not

HVO_0694 gptA2 probable phosphoribosyltransferase

HVO_0693 - auxin permease family transport protein

//

>NGHB#682 107

CMPLX not

HVO_0695 - small CPxCG-related zinc finger protein

HVO_0696 phzF1 PhzF family protein

//

>NGHB#684 54

CMPLX not

HVO_0698 nop10 tRNA/rRNA pseudouridine synthase complex protein Nop10

HVO_0697 - PAC2 family protein

//

>NGHB#685 4

CMPLX not

HVO_0699 tif2a translation initiation factor aIF-2 alpha subunit

HVO_0698 nop10 tRNA/rRNA pseudouridine synthase complex protein Nop10

//

>NGHB#686 18

CMPLX not

HVO_0700 rps27e 30S ribosomal protein S27e

HVO_0699 tif2a translation initiation factor aIF-2 alpha subunit

//

>NGHB#687 3

CMPLX yes

HVO_0701 rpl42e 50S ribosomal protein L42e

HVO_0700 rps27e 30S ribosomal protein S27e

//

>NGHB#691 -8

CMPLX not

HVO_0704 - probable glycosyltransferase, type 2

HVO_0705 - GtrA family protein

//

>NGHB#692 346

CMPLX not

HVO_0705 - GtrA family protein

HVO_0707 - probable iron-sulfur protein (2Fe-2S)

//

>NGHB#694 -4

CMPLX not

HVO_0709 pabA aminodeoxychorismate synthase component 2

HVO_0708 pabC aminodeoxychorismate lyase

//

>NGHB#695 -4

CMPLX yes

HVO_0710 pabB aminodeoxychorismate synthase component 1

HVO_0709 pabA aminodeoxychorismate synthase component 2

//

>NGHB#696 114

CMPLX ---

HVO_0711 - conserved hypothetical protein

HVO_0710 pabB aminodeoxychorismate synthase component 1

//

>NGHB#697 43

CMPLX ---

HVO_0712 aroE shikimate dehydrogenase (nonfunctional)

HVO_0711 - conserved hypothetical protein

//

>NGHB#699 97

CMPLX not

HVO_0714 - homolog to sodium/calcium antiporter

HVO_0715 - sodium/calcium antiporter

//

>NGHB#700 57

CMPLX not

HVO_0715 - sodium/calcium antiporter

HVO_0716 dtdA D-aminoacyl-tRNA deacylase

//

>NGHB#701 157

CMPLX not

HVO_0716 dtdA D-aminoacyl-tRNA deacylase

HVO_0717 ftsZ1 cell division protein FtsZ, type I

//

>NGHB#702 180

CMPLX not

HVO_0717 ftsZ1 cell division protein FtsZ, type I

HVO_0718 secE, sec61c protein translocase subunit SecE

//

>NGHB#703 1

CMPLX not

HVO_0718 secE, sec61c protein translocase subunit SecE

HVO_0719 spt5, nusG transcription elongation factor Spt5

//

>NGHB#707 70

CMPLX not

HVO_0723 - nicotinamide mononucleotide deamidase

HVO_0722 - DUF457 family protein

//

>NGHB#710 87

CMPLX not

HVO_0726 endV, nfi endonuclease 5

HVO_0725 - probable oxidoreductase (short-chain dehydrogenase family)

//

>NGHB#711 1

CMPLX not

HVO_0727 - rhomboid family protease

HVO_0726 endV, nfi endonuclease 5

//

>NGHB#712 199

CMPLX ---

HVO_0728 - conserved hypothetical protein

HVO_0727 - rhomboid family protease

//

>NGHB#713 201

CMPLX ---

HVO_0729 ipp, ppa inorganic pyrophosphatase

HVO_0728 - conserved hypothetical protein

//

>NGHB#716 -8

CMPLX ---

HVO_0732 rnhA2 ribonuclease H, type 1

HVO_0731 - conserved hypothetical protein

//

>NGHB#718 84

CMPLX not

HVO_0733 tfb4 transcription initiation factor TFB

HVO_0734 nreA DNA repair protein NreA

//

>NGHB#719 35

CMPLX ---

HVO_0734 nreA DNA repair protein NreA

HVO_0735 - conserved hypothetical protein

//

>NGHB#721 79

CMPLX not

HVO_0737 - Abi/CAAX domain protein

HVO_0736 - DUF302 family protein

//

>NGHB#722 2

CMPLX ---

HVO_0738 - conserved hypothetical protein

HVO_0737 - Abi/CAAX domain protein

//

>NGHB#725 -4

CMPLX not

HVO_0741 polX DNA-directed DNA polymerase X

HVO_0740 - DUF82 family protein

//

>NGHB#726 -4

CMPLX ---

HVO_0742 - conserved hypothetical protein

HVO_0741 polX DNA-directed DNA polymerase X

//

>NGHB#729 205

CMPLX not

HVO_0745 cetZ2, ftsZ3 FtsZ family protein CetZ, type III

HVO_0744 - homolog to carboxylate-amine ligase

//

>NGHB#731 -4

CMPLX ---

HVO_0746 - conserved hypothetical protein

HVO_0747 - conserved hypothetical protein

//

>NGHB#732 32

CMPLX ---

HVO_0747 - conserved hypothetical protein

HVO_0748 pilB2 type IV pilus biogenesis complex ATPase subunit

//

>NGHB#733 -7

CMPLX yes

HVO_0748 pilB2 type IV pilus biogenesis complex ATPase subunit

HVO_0749 pilC2 type IV pilus biogenesis complex membrane subunit

//

>NGHB#734 54

CMPLX ---

HVO_0749 pilC2 type IV pilus biogenesis complex membrane subunit

HVO_0750 - conserved hypothetical protein

//

>NGHB#735 -23

CMPLX ---

HVO_0750 - conserved hypothetical protein

HVO_0751 - conserved hypothetical protein

//

>NGHB#736 -4

CMPLX ---

HVO_0751 - conserved hypothetical protein

HVO_0752 - conserved hypothetical protein

//

>NGHB#737 -4

CMPLX ---

HVO_0752 - conserved hypothetical protein

HVO_0753 - conserved hypothetical protein

//

>NGHB#738 58

CMPLX ---

HVO_0753 - conserved hypothetical protein

HVO_0754 - conserved hypothetical protein

//

>NGHB#739 -4

CMPLX ---

HVO_0754 - conserved hypothetical protein

HVO_0755 - homolog to NAD-dependent epimerase/dehydratase

//

>NGHB#740 101

CMPLX not

HVO_0755 - homolog to NAD-dependent epimerase/dehydratase

HVO_0756 - DHH/RecJ family phosphoesterase

//

>NGHB#741 91

CMPLX not

HVO_0756 - DHH/RecJ family phosphoesterase

HVO_0757 - probable oxidoreductase (aldo-keto reductase family protein)

//

>NGHB#744 82

CMPLX not

HVO_0759 - major facilitator superfamily transport protein

HVO_0760 - probable glycosyltransferase, type 2

//

>NGHB#745 -4

CMPLX ---

HVO_0760 - probable glycosyltransferase, type 2

HVO_0761 - conserved hypothetical protein

//

>NGHB#746 29

CMPLX ---

HVO_0761 - conserved hypothetical protein

HVO_0762 - NUDIX family hydrolase

//

>NGHB#749 282

CMPLX ---

HVO_0764 - conserved hypothetical protein

HVO_0765 - FAD-dependent oxidoreductase

//

>NGHB#751 -4

CMPLX not

HVO_0767 - small CPxCG-related zinc finger protein

HVO_0766 hsp20D Hsp20-type molecular chaperone

//

>NGHB#754 64

CMPLX ---

HVO_0770 - conserved hypothetical protein

HVO_0769 - TRAM domain protein

//

>NGHB#755 96

CMPLX ---

HVO_0771 - beta-lactamase domain protein

HVO_0770 - conserved hypothetical protein

//

>NGHB#756 114

CMPLX not

HVO_0772 - NP_1176A family transcription regulator

HVO_0771 - beta-lactamase domain protein

//

>NGHB#757 121

CMPLX not

HVO_0772A - small CPxCG-related zinc finger protein

HVO_0772 - NP_1176A family transcription regulator

//

>NGHB#759 70

CMPLX not

HVO_0773 - probable S-adenosylmethionine-dependent methyltransferase

HVO_0774 - probable glycosyltransferase, type 2

//

>NGHB#762 54

CMPLX ---

HVO_0776 - HTH-10 family transcription regulator

HVO_0777 - conserved hypothetical protein

//

>NGHB#763 162

CMPLX ---

HVO_0777 - conserved hypothetical protein

HVO_0778 ths3, cct3 thermosome subunit 3

//

>NGHB#764 187

CMPLX not

HVO_0778 ths3, cct3 thermosome subunit 3

HVO_0779 cat6 transport protein (probable substrate cationic amino acids)

//

>NGHB#766 -1

CMPLX not

HVO_0781 - S-adenosylmethionine hydroxide adenosyltransferase family protein

HVO_0780 - UPF0126 family protein

//

>NGHB#767 1

CMPLX not

HVO_0782 nadM nicotinamide-nucleotide adenylyltransferase

HVO_0781 - S-adenosylmethionine hydroxide adenosyltransferase family protein

//

>NGHB#769 6

CMPLX not

HVO_0783 lon ATP-dependent protease Lon

HVO_0784 - Abi/CAAX domain protein

//

>NGHB#770 102

CMPLX ---

HVO_0784 - Abi/CAAX domain protein

HVO_0785 - conserved hypothetical protein

//

>NGHB#773 -4

CMPLX not

HVO_0787 trpC indole-3-glycerol-phosphate synthase

HVO_0788 trpB tryptophan synthase beta subunit

//

>NGHB#774 -1

CMPLX yes

HVO_0788 trpB tryptophan synthase beta subunit

HVO_0789 trpA tryptophan synthase alpha subunit

//

>NGHB#775 24

CMPLX not

HVO_0789 trpA tryptophan synthase alpha subunit

HVO_0790 fba2 2-amino-3,7-dideoxy-D-threo-hept-6-ulosonate synthase

//

>NGHB#776 92

CMPLX not

HVO_0790 fba2 2-amino-3,7-dideoxy-D-threo-hept-6-ulosonate synthase

HVO_0791 ogt2 probable methylated-DNA--protein-cysteine methyltransferase

//

>NGHB#777 750

CMPLX not

HVO_0791 ogt2 probable methylated-DNA--protein-cysteine methyltransferase

HVO_0792 aroB 3-dehydroquinate synthase, type II

//

>NGHB#779 115

CMPLX not

HVO_0794 zim CTAG modification methylase

HVO_0793 - small CPxCG-related zinc finger protein

//

>NGHB#780 63

CMPLX not

HVO_0795 tfb6 transcription initiation factor TFB

HVO_0794 zim CTAG modification methylase

//

>NGHB#782 20

CMPLX not

HVO_0796 - ABC-type transport system periplasmic substrate-binding protein (probable substrate iron/cobalamin)

HVO_0797 - probable inosine/xanthosine triphosphatase

//

>NGHB#786 82

CMPLX not

HVO_0801 - stomatin family protein

HVO_0800 - HTH domain protein

//

>NGHB#787 139

CMPLX not

HVO_0802 - NfeD domain protein

HVO_0801 - stomatin family protein

//

>NGHB#788 79

CMPLX not

HVO_0803 - homolog to restriction system mrr

HVO_0802 - NfeD domain protein

//

>NGHB#789 -4

CMPLX ---

HVO_0804 - conserved hypothetical protein

HVO_0803 - homolog to restriction system mrr

//

>NGHB#790 265

CMPLX ---

HVO_0805 - conserved hypothetical protein

HVO_0804 - conserved hypothetical protein

//

>NGHB#791 44

CMPLX ---

HVO_0806 pykA pyruvate kinase

HVO_0805 - conserved hypothetical protein

//

>NGHB#792 114

CMPLX ---

HVO_0807 - conserved hypothetical protein

HVO_0806 pykA pyruvate kinase

//

>NGHB#793 71

CMPLX ---

HVO_0808 - conserved hypothetical protein

HVO_0807 - conserved hypothetical protein

//

>NGHB#794 130

CMPLX ---

HVO_0809 metS methionine--tRNA ligase

HVO_0808 - conserved hypothetical protein

//

>NGHB#795 290

CMPLX ---

HVO_0810 - conserved hypothetical protein

HVO_0809 metS methionine--tRNA ligase

//

>NGHB#796 48

CMPLX ---

HVO_0811 panD aspartate 1-decarboxylase

HVO_0810 - conserved hypothetical protein

//

>NGHB#797 129

CMPLX not

HVO_0812 ppsA phosphoenolpyruvate synthase

HVO_0811 panD aspartate 1-decarboxylase

//

>NGHB#798 117

CMPLX not

HVO_0813 - small CPxCG-related zinc finger protein

HVO_0812 ppsA phosphoenolpyruvate synthase

//

>NGHB#804 86

CMPLX not

HVO_0818 thrC2 threonine synthase

HVO_0819 sirR SirR/DtxR family transcription regulator SirR

//

>NGHB#805 -4

CMPLX not

HVO_0819 sirR SirR/DtxR family transcription regulator SirR

HVO_0820 - Gdt1 family protein

//

>NGHB#806 86

CMPLX not

HVO_0820 - Gdt1 family protein

HVO_0821 - LysE family transport protein

//

>NGHB#807 135

CMPLX not

HVO_0821 - LysE family transport protein

HVO_0822 gldA glycerol-1-phosphate dehydrogenase (NAD(P))

//

>NGHB#808 269

CMPLX not

HVO_0822 gldA glycerol-1-phosphate dehydrogenase (NAD(P))

HVO_0823 - DUF420 family protein

//

>NGHB#809 105

CMPLX not

HVO_0823 - DUF420 family protein

HVO_0824 - probable glycosyltransferase, type 1

//

>NGHB#811 144

CMPLX not

HVO_0826 - peptidase M42 family protein

HVO_0825 hcpA halocyanin

//

>NGHB#813 13

CMPLX ---

HVO_0827 - MTH865 family protein

HVO_0828 - conserved hypothetical protein

//

>NGHB#814 39

CMPLX ---

HVO_0828 - conserved hypothetical protein

HVO_0829 - peptidase S9 family protein

//

>NGHB#815 57

CMPLX not

HVO_0829 - peptidase S9 family protein

HVO_0830 mmcB methylmalonyl-CoA mutase subunit B (cobalamin-binding subunit)

//

>NGHB#816 -4

CMPLX not

HVO_0830 mmcB methylmalonyl-CoA mutase subunit B (cobalamin-binding subunit)

HVO_0831 ygfD YgfD family GTPase

//

>NGHB#817 87

CMPLX not

HVO_0831 ygfD YgfD family GTPase

HVO_0832 - alpha/beta hydrolase fold protein

//

>NGHB#818 31

CMPLX ---

HVO_0832 - alpha/beta hydrolase fold protein

HVO_0833 - conserved hypothetical protein

//

>NGHB#820 -4

CMPLX not

HVO_0835 acaB1 acetyl-CoA C-acetyltransferase

HVO_0834 - DUF35 family protein

//

>NGHB#822 143

CMPLX not

HVO_0836 ywaD1 probable M28 family peptidase (homolog to aminopeptidase YwaD)

HVO_0837 nadK2 probable NAD kinase (polyphosphate/ATP)

//

>NGHB#824 137

CMPLX ---

HVO_0839 - conserved hypothetical protein

HVO_0838 - conserved hypothetical protein

//

>NGHB#825 -8

CMPLX ---

HVO_0840 - conserved hypothetical protein

HVO_0839 - conserved hypothetical protein

//

>NGHB#826 39

CMPLX ---

HVO_0841 petD cytochrome bc1 complex cytochrome b/c subunit

HVO_0840 - conserved hypothetical protein

//

>NGHB#827 10

CMPLX yes

HVO_0842 petB cytochrome bc1 complex cytochrome b subunit

HVO_0841 petD cytochrome bc1 complex cytochrome b/c subunit

//

>NGHB#828 3

CMPLX ---

HVO_0843 - conserved hypothetical protein

HVO_0842 petB cytochrome bc1 complex cytochrome b subunit

//

>NGHB#829 -1

CMPLX ---

HVO_0844 hcpB halocyanin

HVO_0843 - conserved hypothetical protein

//

>NGHB#830 85

CMPLX ---

HVO_0845 - conserved hypothetical protein

HVO_0844 hcpB halocyanin

//

>NGHB#836 26

CMPLX ---

HVO_0852 - NP_1176A family transcription regulator

HVO_0852_A - conserved hypothetical protein

//

>NGHB#837 -4

CMPLX ---

HVO_0852_A - conserved hypothetical protein

HVO_0853 mre11 DNA double-strand break repair protein Mre11

//

>NGHB#838 -4

CMPLX yes

HVO_0853 mre11 DNA double-strand break repair protein Mre11

HVO_0854 rad50 DNA double-strand break repair ATPase Rad50

//

>NGHB#840 27

CMPLX ---

HVO_0856 - conserved hypothetical protein

HVO_0855 - HTH domain protein

//

>NGHB#841 48

CMPLX ---

HVO_0857 - conserved hypothetical protein

HVO_0856 - conserved hypothetical protein

//

>NGHB#843 218

CMPLX not

HVO_0858 polB1 DNA-directed DNA polymerase B (intein-containing)

HVO_0859 sufC Fe-S cluster assembly ATPase SufC

//

>NGHB#844 12

CMPLX not

HVO_0859 sufC Fe-S cluster assembly ATPase SufC

HVO_0860 sufB1 SufB domain protein

//

>NGHB#845 3

CMPLX not

HVO_0860 sufB1 SufB domain protein

HVO_0861 sufB2, sufD SufB domain protein

//

>NGHB#846 143

CMPLX not

HVO_0861 sufB2, sufD SufB domain protein

HVO_0862 - ferritin domain protein

//

>NGHB#847 4

CMPLX not

HVO_0862 - ferritin domain protein

HVO_0863 - SirR/DtxR family transcription regulator

//

>NGHB#852 108

CMPLX not

HVO_0869 gltB glutamate synthase (ferredoxin) large subunit

HVO_0868 - GalE family epimerase/dehydratase

//

>NGHB#853 157

CMPLX not

HVO_0870 proS proline--tRNA ligase

HVO_0869 gltB glutamate synthase (ferredoxin) large subunit

//

>NGHB#857 71

CMPLX not

HVO_0874 - zinc-dependent nuclease

HVO_0875 - DUF1628 domain protein

//

>NGHB#858 63

CMPLX not

HVO_0875 - DUF1628 domain protein

HVO_0876 mgsA methylglyoxal synthase

//

>NGHB#859 136

CMPLX ---

HVO_0876 mgsA methylglyoxal synthase

HVO_0877 - conserved hypothetical protein

//

>NGHB#861 69

CMPLX not

HVO_0879 - DUF371 family protein

HVO_0878 nthB endonuclease 3

//

>NGHB#863 90

CMPLX ---

HVO_0880 - conserved hypothetical protein

HVO_0881 sppA1 signal peptide peptidase SppA

//

>NGHB#864 43

CMPLX not

HVO_0881 sppA1 signal peptide peptidase SppA

HVO_0882 - DUF373 family protein

//

>NGHB#866 88

CMPLX ---

HVO_0884 - probable oxidoreductase (aldo-keto reductase family protein)

HVO_0883 - conserved hypothetical protein

//

>NGHB#868 22

CMPLX ---

HVO_0885 - small CPxCG-related zinc finger protein

HVO_0886 - hypothetical protein

//

>NGHB#870 -4

CMPLX yes

HVO_0888 korA oxoglutarate--ferredoxin oxidoreductase alpha subunit

HVO_0887 korB oxoglutarate--ferredoxin oxidoreductase beta subunit

//

>NGHB#873 -4

CMPLX yes

HVO_0891 nosF ABC-type transport system ATP-binding protein (probable substrate copper)

HVO_0890 nosY ABC-type transport system permease protein (probable substrate copper)

//

>NGHB#874 -8

CMPLX yes

HVO_0892 nosD ABC-type transport system periplasmic substrate-binding protein (probable substrate copper)

HVO_0891 nosF ABC-type transport system ATP-binding protein (probable substrate copper)

//

>NGHB#876 186

CMPLX not

HVO_0893 mmcA1 methylmalonyl-CoA mutase subunit A

HVO_0894 acs1 acyl-CoA synthetase

//

>NGHB#880 46

CMPLX ---

HVO_0898 - hypothetical protein

HVO_0897 - conserved hypothetical protein

//

>NGHB#882 123

CMPLX yes

HVO_0899 livJ1 ABC-type transport system periplasmic substrate-binding protein (probable substrate branched-chain amino acids)

HVO_0900 livH1 ABC-type transport system permease protein (probable substrate branched-chain amino acids)

//

>NGHB#883 -4

CMPLX yes

HVO_0900 livH1 ABC-type transport system permease protein (probable substrate branched-chain amino acids)

HVO_0901 livM1 ABC-type transport system permease protein (probable substrate branched-chain amino acids)

//

>NGHB#884 -4

CMPLX yes

HVO_0901 livM1 ABC-type transport system permease protein (probable substrate branched-chain amino acids)

HVO_0902 livG1 ABC-type transport system ATP-binding protein (probable substrate branched-chain amino acids)

//

>NGHB#885 -4

CMPLX yes

HVO_0902 livG1 ABC-type transport system ATP-binding protein (probable substrate branched-chain amino acids)

HVO_0903 livF1 ABC-type transport system ATP-binding protein (probable substrate branched-chain amino acids)

//

>NGHB#886 2

CMPLX not

HVO_0903 livF1 ABC-type transport system ATP-binding protein (probable substrate branched-chain amino acids)

HVO_0905 trxA6 thioredoxin

//

>NGHB#888 54

CMPLX ---

HVO_0906 - conserved hypothetical protein

HVO_0904 - UspA domain protein

//

>NGHB#891 62

CMPLX ---

HVO_0908 - conserved hypothetical protein

HVO_0907 coxA1 cox-type terminal oxidase subunit I

//

>NGHB#892 102

CMPLX ---

HVO_0909 - conserved hypothetical protein

HVO_0908 - conserved hypothetical protein

//

>NGHB#893 45

CMPLX ---

HVO_0910 - conserved hypothetical protein

HVO_0909 - conserved hypothetical protein

//

>NGHB#895 -4

CMPLX not

HVO_0911 drg GTP-binding protein Drg

HVO_0912 - TIGR04206 family protein

//

>NGHB#898 154

CMPLX not

HVO_0914 - glyoxalase domain protein

HVO_0915 artA archaeosortase A

//

>NGHB#901 87

CMPLX ---

HVO_0917 - DoxX domain protein

HVO_0918 - conserved hypothetical protein

//

>NGHB#902 102

CMPLX ---

HVO_0918 - conserved hypothetical protein

HVO_0919 - small CPxCG-related zinc finger protein

//

>NGHB#903 90

CMPLX not

HVO_0919 - small CPxCG-related zinc finger protein

HVO_0920 - probable iron-sulfur protein (2Fe-2S)

//

>NGHB#906 96

CMPLX not

HVO_0922 - ISH3-type transposase ISH51 (nonfunctional)

HVO_0924 - ISH5-type transposase ISHvo11

//

>NGHB#907 340

CMPLX ---

HVO_0930 - conserved hypothetical protein

HVO_0929 trm5, trmM tRNA (guanine(37)-N(1))-methyltransferase

//

>NGHB#913 -8

CMPLX not

HVO_0935 fdhA formate dehydrogenase alpha subunit

HVO_0936 - YqeC family protein

//

>NGHB#915 73

CMPLX not

HVO_0938 - UspA domain protein

HVO_0937 - FMN-binding domain protein

//

>NGHB#916 30

CMPLX ---

HVO_0939 - conserved hypothetical protein

HVO_0938 - UspA domain protein

//

>NGHB#917 66

CMPLX ---

HVO_0940 cpx P-type transport ATPase (probable substrate copper/metal cation)

HVO_0939 - conserved hypothetical protein

//

>NGHB#918 -4

CMPLX ---

HVO_0941 - conserved hypothetical protein

HVO_0940 cpx P-type transport ATPase (probable substrate copper/metal cation)

//

>NGHB#919 1

CMPLX ---

HVO_0942 cbaE ba3-type terminal oxidase subunit CbaE

HVO_0941 - conserved hypothetical protein

//

>NGHB#921 0

CMPLX yes

HVO_0943 cbaD ba3-type terminal oxidase subunit CbaD

HVO_0944 cbaB ba3-type terminal oxidase subunit II

//

>NGHB#922 -1

CMPLX yes

HVO_0944 cbaB ba3-type terminal oxidase subunit II

HVO_0945 cbaA ba3-type terminal oxidase subunit I

//

>NGHB#923 -4

CMPLX not

HVO_0945 cbaA ba3-type terminal oxidase subunit I

HVO_0946 cbaC cbaC protein

//

>NGHB#924 181

CMPLX ---

HVO_0946 cbaC cbaC protein

HVO_0947 - conserved hypothetical protein

//

>NGHB#925 134

CMPLX ---

HVO_0947 - conserved hypothetical protein

HVO_0948 - UspA domain protein

//

>NGHB#929 188

CMPLX ---

HVO_0952 cynT carbonic anhydrase

HVO_0951 - hypothetical protein

//

>NGHB#930 22

CMPLX not

HVO_0953 - DUF304 domain protein

HVO_0952 cynT carbonic anhydrase

//

>NGHB#931 -4

CMPLX not

HVO_0954 - DUF304 domain protein

HVO_0953 - DUF304 domain protein

//

>NGHB#932 83

CMPLX ---

HVO_0955 - conserved hypothetical protein

HVO_0954 - DUF304 domain protein

//

>NGHB#933 -10

CMPLX ---

HVO_0956 - metallophosphoesterase domain protein

HVO_0955 - conserved hypothetical protein

//

>NGHB#934 -4

CMPLX ---

HVO_0957 - conserved hypothetical protein

HVO_0956 - metallophosphoesterase domain protein

//

>NGHB#935 106

CMPLX ---

HVO_0958 - conserved hypothetical protein

HVO_0957 - conserved hypothetical protein

//

>NGHB#936 113

CMPLX ---

HVO_0959 - DUF2071 family protein

HVO_0958 - conserved hypothetical protein

//

>NGHB#938 1

CMPLX not

HVO_0960 - probable oxidoreductase (aldo-keto reductase family protein)

HVO_0961 - chimeric protein (nonfunctional)

//

>NGHB#939 26

CMPLX not

HVO_0961 - chimeric protein (nonfunctional)

HVO_0962 - HTH domain protein

//

>NGHB#940 -1

CMPLX ---

HVO_0962 - HTH domain protein

HVO_0963 - conserved hypothetical protein

//

>NGHB#941 214

CMPLX ---

HVO_0963 - conserved hypothetical protein

HVO_0964 trxA3 thioredoxin

//

>NGHB#942 60

CMPLX not

HVO_0964 trxA3 thioredoxin

HVO_0965 deoA AMP phosphorylase

//

>NGHB#943 49

CMPLX not

HVO_0965 deoA AMP phosphorylase

HVO_0966 - ribose-1,5-bisphosphate isomerase

//

>NGHB#947 282

CMPLX not

HVO_0970 rbcL ribulose-bisphosphate carboxylase

HVO_0969 htr39 transducer protein Htr39

//

>NGHB#948 128

CMPLX not

HVO_0971 hel308b ATP-dependent DNA helicase Hel308b

HVO_0970 rbcL ribulose-bisphosphate carboxylase

//

>NGHB#949 228

CMPLX not

HVO_0972 pilA1 pilin PilA

HVO_0971 hel308b ATP-dependent DNA helicase Hel308b

//

>NGHB#950 215

CMPLX not

HVO_0973 aspC3 pyridoxal phosphate-dependent aminotransferase

HVO_0972 pilA1 pilin PilA

//

>NGHB#951 -4

CMPLX not

HVO_0974 ribH 6,7-dimethyl-8-ribityllumazine synthase

HVO_0973 aspC3 pyridoxal phosphate-dependent aminotransferase

//

>NGHB#953 171

CMPLX not

HVO_0975 - TIGR03663 family protein

HVO_0976 purK 5-(carboxyamino)imidazole ribonucleotide synthase

//

>NGHB#954 6

CMPLX not

HVO_0976 purK 5-(carboxyamino)imidazole ribonucleotide synthase

HVO_0977 purE N5-carboxyaminoimidazole ribonucleotide mutase

//

>NGHB#955 199

CMPLX not

HVO_0977 purE N5-carboxyaminoimidazole ribonucleotide mutase

HVO_0978 nuoA NADH dehydrogenase-like complex subunit A

//

>NGHB#956 -4

CMPLX yes

HVO_0978 nuoA NADH dehydrogenase-like complex subunit A

HVO_0979 nuoB NADH dehydrogenase-like complex subunit B

//

>NGHB#957 -4

CMPLX yes

HVO_0979 nuoB NADH dehydrogenase-like complex subunit B

HVO_0980 nuoCD1 NADH dehydrogenase-like complex subunit CD

//

>NGHB#958 -1

CMPLX yes

HVO_0980 nuoCD1 NADH dehydrogenase-like complex subunit CD

HVO_0981 nuoH NADH dehydrogenase-like complex subunit H

//

>NGHB#959 -4

CMPLX yes

HVO_0981 nuoH NADH dehydrogenase-like complex subunit H

HVO_0982 nuoI NADH dehydrogenase-like complex subunit I

//

>NGHB#960 105

CMPLX yes

HVO_0982 nuoI NADH dehydrogenase-like complex subunit I

HVO_0983 nuoJ1 NADH dehydrogenase-like complex subunit J1

//

>NGHB#961 -4

CMPLX yes

HVO_0983 nuoJ1 NADH dehydrogenase-like complex subunit J1

HVO_0984 nuoJ2 NADH dehydrogenase-like complex subunit J2

//

>NGHB#962 -1

CMPLX yes

HVO_0984 nuoJ2 NADH dehydrogenase-like complex subunit J2

HVO_0985 nuoK NADH dehydrogenase-like complex subunit K

//

>NGHB#963 3

CMPLX yes

HVO_0985 nuoK NADH dehydrogenase-like complex subunit K

HVO_0986 nuoL NADH dehydrogenase-like complex subunit L

//

>NGHB#964 -4

CMPLX yes

HVO_0986 nuoL NADH dehydrogenase-like complex subunit L

HVO_0987 nuoM NADH dehydrogenase-like complex subunit M

//

>NGHB#965 0

CMPLX yes

HVO_0987 nuoM NADH dehydrogenase-like complex subunit M

HVO_0988 nuoN NADH dehydrogenase-like complex subunit N

//

>NGHB#968 43

CMPLX not

HVO_0990 - DHH/RecJ family phosphoesterase

HVO_0991 - CBS/parB domain protein

//

>NGHB#970 30

CMPLX ---

HVO_0993 - small CPxCG-related zinc finger protein

HVO_0992 - conserved hypothetical protein

//

>NGHB#974 95

CMPLX not

HVO_0996 - DUF21/CBS domain protein

HVO_0997 pepQ1 probable Xaa-Pro dipeptidase

//

>NGHB#975 57

CMPLX ---

HVO_0997 pepQ1 probable Xaa-Pro dipeptidase

HVO_0998 - conserved hypothetical protein

//

>NGHB#977 26

CMPLX not

HVO_1000 acdA acetate--CoA ligase (ADP-forming)

HVO_0999 - DRTGG domain protein

//

>NGHB#979 210

CMPLX ---

HVO_1001 - conserved hypothetical protein

HVO_1002 - conserved hypothetical protein

//

>NGHB#980 163

CMPLX ---

HVO_1002 - conserved hypothetical protein

HVO_1003 gufA2 GufA family transport protein (probable substrate zinc)

//

>NGHB#982 133

CMPLX ---

HVO_1005 - ABC-type transport system permease protein

HVO_1004 - conserved hypothetical protein

//

>NGHB#983 -4

CMPLX yes

HVO_1006 - ABC-type transport system ATP-binding protein

HVO_1005 - ABC-type transport system permease protein

//

>NGHB#987 52

CMPLX ---

HVO_1008A - conserved hypothetical protein (nonfunctional)

HVO_1009 - probable oxidoreductase (aldo-keto reductase family protein)

//

>NGHB#989 12

CMPLX not

HVO_1011 - thioredoxin domain protein

HVO_1010 - DUF457 family protein

//

>NGHB#990 66

CMPLX ---

HVO_1012 - conserved hypothetical protein

HVO_1011 - thioredoxin domain protein

//

>NGHB#991 27

CMPLX ---

HVO_1013 ctaB heme o synthase

HVO_1012 - conserved hypothetical protein

//

>NGHB#994 -4

CMPLX not

HVO_1016 - JAB domain protein

HVO_1015 ribL FAD synthase

//

>NGHB#996 106

CMPLX not

HVO_1017 - ketosamine kinase domain protein

HVO_1018 han Hef-associated 3' exonuclease Han

//

>NGHB#998 126

CMPLX not

HVO_1020 - HEAT-PBS family protein

HVO_1019 - phospholipase D domain protein

//

>NGHB#1000 63

CMPLX not

HVO_1021 - probable carbon-nitrogen hydrolase

HVO_1022 - NamA family oxidoreductase

//

>NGHB#1001 101

CMPLX ---

HVO_1022 - NamA family oxidoreductase

HVO_1023 - conserved hypothetical protein

//

>NGHB#1003 -1

CMPLX yes

HVO_1025 acaB2 acetyl-CoA C-acetyltransferase catalytic subunit

HVO_1024 - acetyl-CoA C-acetyltransferase small subunit

//

>NGHB#1006 168

CMPLX not

HVO_1028 - probable oxidoreductase (short-chain dehydrogenase family)

HVO_1027 tatAo Sec-independent protein translocase protein TatAo

//

>NGHB#1007 361

CMPLX not

HVO_1030 - DUF4382 domain protein

HVO_1028 - probable oxidoreductase (short-chain dehydrogenase family)

//

>NGHB#1008 83

CMPLX not

HVO_1031 trxB1 thioredoxin-disulfide reductase

HVO_1030 - DUF4382 domain protein

//

>NGHB#1009 119

CMPLX not

HVO_1032 trm9 tRNA (carboxymethyluridine(34)-5-O)-methyltransferase

HVO_1031 trxB1 thioredoxin-disulfide reductase

//

>NGHB#1010 84

CMPLX not

HVO_1033 pilC3 type IV pilus biogenesis complex membrane subunit

HVO_1032 trm9 tRNA (carboxymethyluridine(34)-5-O)-methyltransferase

//

>NGHB#1011 1

CMPLX yes

HVO_1034 pilB3 type IV pilus biogenesis complex ATPase subunit

HVO_1033 pilC3 type IV pilus biogenesis complex membrane subunit

//

>NGHB#1012 99

CMPLX ---

HVO_1035 - conserved hypothetical protein

HVO_1034 pilB3 type IV pilus biogenesis complex ATPase subunit

//

>NGHB#1013 94

CMPLX ---

HVO_1036 - cupin 2 barrel domain protein

HVO_1035 - conserved hypothetical protein

//

>NGHB#1015 129

CMPLX ---

HVO_1037 - conserved hypothetical protein

HVO_1038 udg4 uracil-DNA glycosylase superfamily protein

//

>NGHB#1017 84

CMPLX not

HVO_1040 - DnaJ domain protein

HVO_1039 - beta-lactamase domain protein (nonfunctional)

//

>NGHB#1019 26

CMPLX not

HVO_1041 - beta-lactamase domain protein

HVO_1042 rpoL DNA-directed RNA polymerase subunit L

//

>NGHB#1021 139

CMPLX not

HVO_1044 hisF imidazole glycerol-phosphate synthase subunit HisF

HVO_1043 - DUF1684 family protein

//

>NGHB#1023 109

CMPLX ---

HVO_1045 - conserved hypothetical protein

HVO_1046 - conserved hypothetical protein

//

>NGHB#1024 95

CMPLX ---

HVO_1046 - conserved hypothetical protein

HVO_1047 qor2 NADPH:quinone reductase

//

>NGHB#1027 -1

CMPLX not

HVO_1049 - PHP domain protein

HVO_1050 asnB asparagine synthase (glutamine-hydrolyzing)

//

>NGHB#1029 101

CMPLX not

HVO_1052 tfb1 transcription initiation factor TFB

HVO_1051 - NUDIX family hydrolase

//

>NGHB#1031 0

CMPLX yes

HVO_1053 gatC, aatC aspartyl/glutamyl-tRNA(Asn/Gln) amidotransferase subunit C

HVO_1054 gatA, aatA aspartyl/glutamyl-tRNA(Asn/Gln) amidotransferase subunit A

//

>NGHB#1032 129

CMPLX not

HVO_1054 gatA, aatA aspartyl/glutamyl-tRNA(Asn/Gln) amidotransferase subunit A

HVO_1055 trkA1 TrkA domain protein

//

>NGHB#1033 -1

CMPLX yes

HVO_1055 trkA1 TrkA domain protein

HVO_1056 trkH1 Trk-type transport system (probable substrate potassium)

//

>NGHB#1035 117

CMPLX yes

HVO_1058 trkA2 TrkA domain protein

HVO_1057 trkH2 Trk-type transport system (probable substrate potassium)

//

>NGHB#1036 85

CMPLX not

HVO_1059 - START domain protein

HVO_1058 trkA2 TrkA domain protein

//

>NGHB#1038 80

CMPLX ---

HVO_1060 - conserved hypothetical protein

HVO_1061 trxB2 oxidoreductase (homolog to thioredoxin-disulfide reductase)

//

>NGHB#1039 94

CMPLX not

HVO_1061 trxB2 oxidoreductase (homolog to thioredoxin-disulfide reductase)

HVO_1062 coaBC phosphopantothenoylcysteine decarboxylase / phosphopantothenate--cysteine ligase

//

>NGHB#1041 -4

CMPLX yes

HVO_1064 mrpF Mrp-type sodium/proton antiporter system subunit F

HVO_1063 mrpG Mrp-type sodium/proton antiporter system subunit G

//

>NGHB#1042 -8

CMPLX yes

HVO_1065 mrpE Mrp-type sodium/proton antiporter system subunit E

HVO_1064 mrpF Mrp-type sodium/proton antiporter system subunit F

//

>NGHB#1043 -1

CMPLX yes

HVO_1066 mrpD1 Mrp-type sodium/proton antiporter system subunit D1

HVO_1065 mrpE Mrp-type sodium/proton antiporter system subunit E

//

>NGHB#1044 -4

CMPLX yes

HVO_1067 mrpC Mrp-type sodium/proton antiporter system subunit C

HVO_1066 mrpD1 Mrp-type sodium/proton antiporter system subunit D1

//

>NGHB#1045 -4

CMPLX yes

HVO_1068 mrpB Mrp-type sodium/proton antiporter system subunit B

HVO_1067 mrpC Mrp-type sodium/proton antiporter system subunit C

//

>NGHB#1046 -4

CMPLX yes

HVO_1069 mrpA Mrp-type sodium/proton antiporter system subunit A

HVO_1068 mrpB Mrp-type sodium/proton antiporter system subunit B

//

>NGHB#1048 254

CMPLX ---

HVO_1070 - ArsR family transcription regulator

HVO_1071 - conserved hypothetical protein

//

>NGHB#1049 158

CMPLX ---

HVO_1071 - conserved hypothetical protein

HVO_1072 apt1 purine phosphoribosyltransferase (adenine phosphoribosyltransferase, xanthine-guanine phosphoribosyltransferase)

//

>NGHB#1050 100

CMPLX not

HVO_1072 apt1 purine phosphoribosyltransferase (adenine phosphoribosyltransferase, xanthine-guanine phosphoribosyltransferase)

HVO_1073 - ThiJ/PfpI domain protein

//

>NGHB#1051 81

CMPLX ---

HVO_1073 - ThiJ/PfpI domain protein

HVO_1074 - conserved hypothetical protein

//

>NGHB#1055 74

CMPLX not

HVO_1078 - homolog to sulfate adenylyltransferase small subunit (nonfunctional)

HVO_1077 dph6 diphthine--ammonia ligase

//

>NGHB#1056 124

CMPLX not

HVO_1079 - homolog to sulfate adenylyltransferase small subunit

HVO_1078 - homolog to sulfate adenylyltransferase small subunit (nonfunctional)

//

>NGHB#1057 137

CMPLX ---

HVO_1080 - conserved hypothetical protein

HVO_1079 - homolog to sulfate adenylyltransferase small subunit

//

>NGHB#1058 167

CMPLX ---

HVO_1081 grx3 glutaredoxin

HVO_1080 - conserved hypothetical protein

//

>NGHB#1059 83

CMPLX not

HVO_1082 pyrE1 homolog to orotate phosphoribosyltransferase

HVO_1081 grx3 glutaredoxin

//

>NGHB#1060 -4

CMPLX not

HVO_1083 gdh glucose 1-dehydrogenase

HVO_1082 pyrE1 homolog to orotate phosphoribosyltransferase

//

>NGHB#1061 329

CMPLX not

HVO_1084 purB adenylosuccinate lyase

HVO_1083 gdh glucose 1-dehydrogenase

//

>NGHB#1063 113

CMPLX not

HVO_1085 purNH phosphoribosylglycinamide formyltransferase / phosphoribosylaminoimidazolecarboxamide formyltransferase

HVO_1086 pcrB2 (S)-3-O-geranylgeranylglyceryl phosphate synthase 2

//

>NGHB#1064 45

CMPLX not

HVO_1086 pcrB2 (S)-3-O-geranylgeranylglyceryl phosphate synthase 2

HVO_1087 - UspA domain protein

//

>NGHB#1065 125

CMPLX not

HVO_1087 - UspA domain protein

HVO_1088 folCP folylpolyglutamate synthase / 7,8-dihydropteroate reductase / dihydropteroate synthase

//

>NGHB#1066 66

CMPLX not

HVO_1088 folCP folylpolyglutamate synthase / 7,8-dihydropteroate reductase / dihydropteroate synthase

HVO_1089 - NRDE domain protein

//

>NGHB#1067 25

CMPLX not

HVO_1089 - NRDE domain protein

HVO_1090 tfeB, rpc34 transcription initiation factor TFE beta subunit

//

>NGHB#1069 6

CMPLX not

HVO_1092 rnp2 ribonuclease P protein component 2

HVO_1091 psmA1 proteasome alpha subunit

//

>NGHB#1070 9

CMPLX not

HVO_1093 - probable S-adenosylmethionine-dependent methyltransferase

HVO_1092 rnp2 ribonuclease P protein component 2

//

>NGHB#1071 -17

CMPLX not

HVO_1094 rnp3 ribonuclease P protein component 3

HVO_1093 - probable S-adenosylmethionine-dependent methyltransferase

//

>NGHB#1072 138

CMPLX not

HVO_1095 - probable secreted glycoprotein

HVO_1094 rnp3 ribonuclease P protein component 3

//

>NGHB#1073 50

CMPLX not

HVO_1096 dapE succinyl-diaminopimelate desuccinylase

HVO_1095 - probable secreted glycoprotein

//

>NGHB#1074 -4

CMPLX not

HVO_1097 dapF diaminopimelate epimerase

HVO_1096 dapE succinyl-diaminopimelate desuccinylase

//

>NGHB#1075 20

CMPLX not

HVO_1098 lysA diaminopimelate decarboxylase

HVO_1097 dapF diaminopimelate epimerase

//

>NGHB#1076 -4

CMPLX not

HVO_1099 dapD 2,3,4,5-tetrahydropyridine-2,6-dicarboxylate N-succinyltransferase

HVO_1098 lysA diaminopimelate decarboxylase

//

>NGHB#1077 -4

CMPLX not

HVO_1100 dapB 4-hydroxy-tetrahydrodipicolinate reductase

HVO_1099 dapD 2,3,4,5-tetrahydropyridine-2,6-dicarboxylate N-succinyltransferase

//

>NGHB#1078 -4

CMPLX not

HVO_1101 dapA 4-hydroxy-tetrahydrodipicolinate synthase

HVO_1100 dapB 4-hydroxy-tetrahydrodipicolinate reductase

//

>NGHB#1081 87

CMPLX ---

HVO_1104 - conserved hypothetical protein

HVO_1103 - conserved hypothetical protein

//

>NGHB#1083 230

CMPLX ---

HVO_1105 - MiaB-like tRNA modifying enzyme

HVO_1105A - conserved hypothetical protein (nonfunctional)

//

>NGHB#1084 48

CMPLX not

HVO_1108 gar1 tRNA/rRNA pseudouridine synthase complex protein Gar1

HVO_1107 - DUF1119 family protein

//

>NGHB#1085 -1

CMPLX not

HVO_1109 srp19 signal recognition particle 19K protein

HVO_1108 gar1 tRNA/rRNA pseudouridine synthase complex protein Gar1

//

>NGHB#1086 96

CMPLX not

HVO_1110 btuF ABC-type transport system periplasmic substrate-binding protein (probable substrate cobalamin)

HVO_1109 srp19 signal recognition particle 19K protein

//

>NGHB#1088 -4

CMPLX yes

HVO_1111 btuC ABC-type transport system permease protein (probable substrate cobalamin)

HVO_1112 btuD ABC-type transport system ATP-binding protein (probable substrate cobalamin)

//

>NGHB#1089 84

CMPLX not

HVO_1112 btuD ABC-type transport system ATP-binding protein (probable substrate cobalamin)

HVO_1113 cetZ3, ftsZ5 FtsZ family protein CetZ, type III

//

>NGHB#1090 86

CMPLX not

HVO_1113 cetZ3, ftsZ5 FtsZ family protein CetZ, type III

HVO_1114 - UPF0278 family protein

//

>NGHB#1092 -1

CMPLX ---

HVO_1116 - HTH domain protein

HVO_1115 - conserved hypothetical protein

//

>NGHB#1094 94

CMPLX not

HVO_1117 boa2 HTH-10 family transcription regulator

HVO_1118 - small CPxCG-related zinc finger protein

//

>NGHB#1097 -4

CMPLX not

HVO_1120 - TIGR04031 family protein

HVO_1121 ahbC, pqqE1 Fe-coproporphyrin synthase AhbC

//

>NGHB#1098 79

CMPLX ---

HVO_1121 ahbC, pqqE1 Fe-coproporphyrin synthase AhbC

HVO_1122 - conserved hypothetical protein

//

>NGHB#1102 116

CMPLX not

HVO_1126 hemAT2, htr10b transducer protein HemAT

HVO_1125 cysS cysteine--tRNA ligase

//

>NGHB#1103 66

CMPLX ---

HVO_1127 - conserved hypothetical protein

HVO_1126 hemAT2, htr10b transducer protein HemAT

//

>NGHB#1105 -1

CMPLX ---

HVO_1128 cbiX2 sirohydrochlorin cobaltochelatase

HVO_1129 - conserved hypothetical protein

//

>NGHB#1107 55

CMPLX ---

HVO_1131 trm112 methyltransferase activator Trm112

HVO_1130 - conserved hypothetical protein

//

>NGHB#1108 108

CMPLX not

HVO_1132 purA adenylosuccinate synthase

HVO_1131 trm112 methyltransferase activator Trm112

//

>NGHB#1115 220

CMPLX not

HVO_1139 fdfT squalene synthase

HVO_1138 coxC1 cox-type terminal oxidase subunit III

//

>NGHB#1122 -8

CMPLX ---

HVO_1146 - conserved hypothetical protein

HVO_1145 rps1e 30S ribosomal protein S1e

//

>NGHB#1123 -4

CMPLX ---

HVO_1147 recJ2 probable replication complex protein RecJ2

HVO_1146 - conserved hypothetical protein

//

>NGHB#1124 4

CMPLX not

HVO_1148 rps15 30S ribosomal protein S15

HVO_1147 recJ2 probable replication complex protein RecJ2

//

>NGHB#1126 1

CMPLX not

HVO_1150 - IS200-type transposase HfIRS11

HVO_1151 - IS1341-type transposase HfIRS11 (nonfunctional)

//

>NGHB#1127 125

CMPLX not

HVO_1151 - IS1341-type transposase HfIRS11 (nonfunctional)

HVO_1153 - probable secreted glycoprotein

//

>NGHB#1129 -4

CMPLX not

HVO_1155 - probable secreted glycoprotein

HVO_1154 - probable secreted glycoprotein

//

>NGHB#1130 53

CMPLX ---

HVO_1156 - conserved hypothetical protein

HVO_1155 - probable secreted glycoprotein

//

>NGHB#1131 -4

CMPLX ---

HVO_1157 - conserved hypothetical protein

HVO_1156 - conserved hypothetical protein

//

>NGHB#1132 -4

CMPLX ---

HVO_1158 - conserved hypothetical protein

HVO_1157 - conserved hypothetical protein

//

>NGHB#1133 -1

CMPLX ---

HVO_1159 pilC4 type IV pilus biogenesis complex membrane subunit

HVO_1158 - conserved hypothetical protein

//

>NGHB#1134 0

CMPLX yes

HVO_1160 pilB4 type IV pilus biogenesis complex ATPase subunit

HVO_1159 pilC4 type IV pilus biogenesis complex membrane subunit

//

>NGHB#1135 384

CMPLX not

HVO_1162 tatAt Sec-independent protein translocase protein TatAt

HVO_1160 pilB4 type IV pilus biogenesis complex ATPase subunit

//

>NGHB#1137 81

CMPLX not

HVO_1163 bcp3 peroxiredoxin

HVO_1164 - HD family hydrolase

//

>NGHB#1139 -4

CMPLX not

HVO_1166 - CRM domain protein

HVO_1165 mscS1 mechanosensitive channel protein MscS

//

>NGHB#1140 111

CMPLX not

HVO_1167 rnp4 ribonuclease P protein component 4

HVO_1166 - CRM domain protein

//

>NGHB#1142 99

CMPLX not

HVO_1168 - probable glycosyltransferase, type 1

HVO_1169 - probable glycosyltransferase, type 1

//

>NGHB#1144 60

CMPLX not

HVO_1171 - DUF2797 family protein

HVO_1170 - glycine-rich protein

//

>NGHB#1145 124

CMPLX not

HVO_1172 - GalE family epimerase/dehydratase

HVO_1171 - DUF2797 family protein

//

>NGHB#1147 84

CMPLX not

HVO_1173 trm56 tRNA (cytidine(56)-2'-O)-methyltransferase

HVO_1174 tfeA transcription initiation factor TFE alpha subunit

//

>NGHB#1148 0

CMPLX not

HVO_1174 tfeA transcription initiation factor TFE alpha subunit

HVO_1175 - DUF2110 family protein

//

>NGHB#1149 4

CMPLX ---

HVO_1175 - DUF2110 family protein

HVO_1176 - conserved hypothetical protein

//

>NGHB#1150 87

CMPLX ---

HVO_1176 - conserved hypothetical protein

HVO_1177 - ADP-ribose pyrophosphatase

//

>NGHB#1155 119

CMPLX ---

HVO_1181 phoU1 PhoU domain protein

HVO_1182 - conserved hypothetical protein

//

>NGHB#1157 173

CMPLX ---

HVO_1184 - DUF1511 family protein

HVO_1183 - conserved hypothetical protein

//

>NGHB#1159 67

CMPLX ---

HVO_1185 - conserved hypothetical protein

HVO_1186 - HTH domain protein

//

>NGHB#1160 43

CMPLX not

HVO_1186 - HTH domain protein

HVO_1187 - START domain protein

//

>NGHB#1161 442

CMPLX ---

HVO_1187 - START domain protein

HVO_1188 - conserved hypothetical protein

//

>NGHB#1166 -4

CMPLX yes

HVO_1194 livM2 ABC-type transport system permease protein (probable substrate branched-chain amino acids)

HVO_1195 livG2 ABC-type transport system ATP-binding protein (probable substrate branched-chain amino acids)

//

>NGHB#1167 -4

CMPLX yes

HVO_1195 livG2 ABC-type transport system ATP-binding protein (probable substrate branched-chain amino acids)

HVO_1196 livF2 ABC-type transport system ATP-binding protein (probable substrate branched-chain amino acids)

//

>NGHB#1169 39

CMPLX not

HVO_1198 - UspA domain protein

HVO_1197 - RIO-type protein kinase domain protein

//

>NGHB#1174 102

CMPLX ---

HVO_1203 arlD1, flaD1 arl cluster protein ArlD

HVO_1202 - conserved hypothetical protein

//

>NGHB#1175 12

CMPLX ---

HVO_1204 - hypothetical protein

HVO_1203 arlD1, flaD1 arl cluster protein ArlD

//

>NGHB#1176 91

CMPLX ---

HVO_1205 cheD taxis cluster protein CheD

HVO_1204 - hypothetical protein

//

>NGHB#1177 21

CMPLX not

HVO_1206 cheC taxis cluster protein CheC

HVO_1205 cheD taxis cluster protein CheD

//

>NGHB#1178 -4

CMPLX not

HVO_1207 cheY response regulator CheY

HVO_1206 cheC taxis cluster protein CheC

//

>NGHB#1179 148

CMPLX ---

HVO_1208 - conserved hypothetical protein

HVO_1207 cheY response regulator CheY

//

>NGHB#1181 213

CMPLX not

HVO_1209 - HTH domain protein

HVO_1210 arlA1, flgA1 archaellin A1

//

>NGHB#1182 10

CMPLX yes

HVO_1210 arlA1, flgA1 archaellin A1

HVO_1211 arlA2, flgA2 archaellin A2

//

>NGHB#1185 -17

CMPLX not

HVO_1213 arlCE, flaCE arl cluster protein ArlCE

HVO_1214 arlF, flaF arl cluster protein ArlF

//

>NGHB#1186 0

CMPLX not

HVO_1214 arlF, flaF arl cluster protein ArlF

HVO_1215 arlG, flaG arl cluster protein ArlG

//

>NGHB#1187 -4

CMPLX not

HVO_1215 arlG, flaG arl cluster protein ArlG

HVO_1216 arlH, flaH arl cluster protein ArlH

//

>NGHB#1188 0

CMPLX yes

HVO_1216 arlH, flaH arl cluster protein ArlH

HVO_1217 arlI, flaI archaellar motor/biogenesis protein ArlI

//

>NGHB#1189 4

CMPLX yes

HVO_1217 arlI, flaI archaellar motor/biogenesis protein ArlI

HVO_1218 arlJ, flaJ archaellar motor/biogenesis protein ArlJ

//

>NGHB#1190 -4

CMPLX not

HVO_1218 arlJ, flaJ archaellar motor/biogenesis protein ArlJ

HVO_1219 cheF2 taxis protein CheF2

//

>NGHB#1191 75

CMPLX ---

HVO_1219 cheF2 taxis protein CheF2

HVO_1220 - conserved hypothetical protein

//

>NGHB#1193 3

CMPLX not

HVO_1222 cheR protein-glutamate O-methyltransferase CheR

HVO_1221 cheF1 taxis protein CheF1

//

>NGHB#1194 -1

CMPLX not

HVO_1223 cheA taxis sensor histidine kinase CheA

HVO_1222 cheR protein-glutamate O-methyltransferase CheR

//

>NGHB#1195 2

CMPLX not

HVO_1224 cheB protein-glutamate methylesterase / protein-glutamine glutaminase CheB

HVO_1223 cheA taxis sensor histidine kinase CheA

//

>NGHB#1199 72

CMPLX not

HVO_1227 uraA1 xanthine/uracil permease family transport protein

HVO_1228 hcpE DUF5059 domain / halocyanin domain protein

//

>NGHB#1201 80

CMPLX ---

HVO_1230 mscS9 mechanosensitive channel protein MscS (nonfunctional)

HVO_1229 - conserved hypothetical protein

//

>NGHB#1203 98

CMPLX not

HVO_1231 - DUF457 family protein

HVO_1232 - putative AhpD family alkylhydroperoxidase

//

>NGHB#1204 85

CMPLX not

HVO_1232 - putative AhpD family alkylhydroperoxidase

HVO_1233 cspA3 cold shock protein

//

>NGHB#1206 49

CMPLX not

HVO_1235 cre2 creatininase domain protein

HVO_1234 phr3 homolog to cryptochrome/photo-lyase

//

>NGHB#1207 69

CMPLX not

HVO_1236 acs3 acyl-CoA synthetase

HVO_1235 cre2 creatininase domain protein

//

>NGHB#1208 63

CMPLX not

HVO_1237 - DUF583 domain protein

HVO_1236 acs3 acyl-CoA synthetase

//

>NGHB#1210 43

CMPLX ---

HVO_1238 - probable oxidoreductase (aldo-keto reductase family protein)

HVO_1238A - conserved hypothetical protein

//

>NGHB#1212 56

CMPLX ---

HVO_1240 - conserved hypothetical protein

HVO_1239 - major facilitator superfamily transport protein

//

>NGHB#1213 -4

CMPLX ---

HVO_1241 prrC SCO1/SenC/PrrC family protein

HVO_1240 - conserved hypothetical protein

//

>NGHB#1216 -1

CMPLX not

HVO_1244 trxA4 thioredoxin

HVO_1243 - homolog to cytochrome c-type biogenesis protein CcdA

//

>NGHB#1217 147

CMPLX not

HVO_1245 - thioredoxin domain protein

HVO_1244 trxA4 thioredoxin

//

>NGHB#1221 36

CMPLX not

HVO_1248 - DUF1918 family protein

HVO_1249 - DUF54 family protein

//

>NGHB#1223 81

CMPLX not

HVO_1251 - DMT superfamily transport protein

HVO_1250 - redoxin domain protein

//

>NGHB#1228 82

CMPLX ---

HVO_1256 - conserved hypothetical protein

HVO_1255 - major facilitator superfamily transport protein

//

>NGHB#1229 7

CMPLX ---

HVO_1257 moxR2 AAA-type ATPase (MoxR subfamily)

HVO_1256 - conserved hypothetical protein

//

>NGHB#1230 -8

CMPLX ---

HVO_1258 - conserved hypothetical protein

HVO_1257 moxR2 AAA-type ATPase (MoxR subfamily)

//

>NGHB#1231 -8

CMPLX ---

HVO_1259 - conserved hypothetical protein

HVO_1258 - conserved hypothetical protein

//

>NGHB#1232 -4

CMPLX ---

HVO_1260 - von Willebrand factor type A domain protein

HVO_1259 - conserved hypothetical protein

//

>NGHB#1233 -1

CMPLX ---

HVO_1261 - conserved hypothetical protein

HVO_1260 - von Willebrand factor type A domain protein

//

>NGHB#1236 -4

CMPLX not

HVO_1264 - DUF304 domain protein

HVO_1263 - DUF304 domain protein

//

>NGHB#1238 -4

CMPLX not

HVO_1265 - Lrp/AsnC family transcription regulator

HVO_1266 aspC2 pyridoxal phosphate-dependent aminotransferase

//

>NGHB#1239 106

CMPLX not

HVO_1266 aspC2 pyridoxal phosphate-dependent aminotransferase

HVO_1267 - UspA domain protein

//

>NGHB#1240 157

CMPLX ---

HVO_1267 - UspA domain protein

HVO_1268 - conserved hypothetical protein

//

>NGHB#1241 50

CMPLX ---

HVO_1268 - conserved hypothetical protein

HVO_1269 - conserved hypothetical protein (nonfunctional)

//

>NGHB#1243 221

CMPLX ---

HVO_1271 - receiver box response regulator

HVO_1270 - conserved hypothetical protein

//

>NGHB#1244 153

CMPLX not

HVO_1272 - TrmB family transcription regulator

HVO_1271 - receiver box response regulator

//

>NGHB#1245 141

CMPLX not

HVO_1273 guaB1 inosine-5'-monophosphate dehydrogenase

HVO_1272 - TrmB family transcription regulator

//

>NGHB#1247 6

CMPLX ---

HVO_1273_A - conserved hypothetical protein

HVO_1274 - conserved hypothetical protein

//

>NGHB#1248 42

CMPLX ---

HVO_1274 - conserved hypothetical protein

HVO_1275 - UPF0047 family protein

//

>NGHB#1249 95

CMPLX not

HVO_1275 - UPF0047 family protein

HVO_1276 - small CPxCG-related zinc finger protein

//

>NGHB#1250 510

CMPLX ---

HVO_1276 - small CPxCG-related zinc finger protein

HVO_1278 - conserved hypothetical protein

//

>NGHB#1251 108

CMPLX ---

HVO_1278 - conserved hypothetical protein

HVO_1279 hdrA, folA1 dihydrofolate reductase

//

>NGHB#1252 93

CMPLX not

HVO_1279 hdrA, folA1 dihydrofolate reductase

HVO_1280 - major facilitator superfamily transport protein

//

>NGHB#1254 1

CMPLX not

HVO_1282 - PtpS family protein

HVO_1281 - probable glycosyltransferase, type 1

//

>NGHB#1255 6

CMPLX not

HVO_1283 - oxidoreductase (homolog to zinc-containing alcohol dehydrogenase / threonine 3-dehydrogenase)

HVO_1282 - PtpS family protein

//

>NGHB#1256 3

CMPLX not

HVO_1284 arfA, gch3 GTP cyclohydrolase 3

HVO_1283 - oxidoreductase (homolog to zinc-containing alcohol dehydrogenase / threonine 3-dehydrogenase)

//

>NGHB#1257 70

CMPLX ---

HVO_1285 - conserved hypothetical protein

HVO_1284 arfA, gch3 GTP cyclohydrolase 3

//

>NGHB#1259 105

CMPLX not

HVO_1286 - HAD superfamily hydrolase

HVO_1287 pfpI protein deglycase PfpI

//

>NGHB#1260 78

CMPLX ---

HVO_1287 pfpI protein deglycase PfpI

HVO_1288 - conserved hypothetical protein

//

>NGHB#1262 98

CMPLX not

HVO_1290 - metal-dependent hydrolase domain protein

HVO_1289 - OsmC domain protein

//

>NGHB#1264 48

CMPLX not

HVO_1291 - fumarylacetoacetase family protein

HVO_1292 - DUF1648 family protein

//

>NGHB#1265 41

CMPLX not

HVO_1292 - DUF1648 family protein

HVO_1293 - LacC domain protein

//

>NGHB#1266 -8

CMPLX not

HVO_1293 - LacC domain protein

HVO_1294 - GMC family oxidoreductase

//

>NGHB#1267 94

CMPLX not

HVO_1294 - GMC family oxidoreductase

HVO_1295 hisC histidinol-phosphate aminotransferase

//

>NGHB#1268 -4

CMPLX not

HVO_1295 hisC histidinol-phosphate aminotransferase

HVO_1296 adk2 probable adenylate kinase

//

>NGHB#1269 -4

CMPLX not

HVO_1296 adk2 probable adenylate kinase

HVO_1297 agsA, pgsA2 probable archaetidylglycerolphosphate synthase

//

>NGHB#1270 337

CMPLX ---

HVO_1297 agsA, pgsA2 probable archaetidylglycerolphosphate synthase

HVO_1297A - conserved hypothetical protein

//

>NGHB#1271 -1

CMPLX ---

HVO_1297A - conserved hypothetical protein

HVO_1298 - sensor box histidine kinase

//

>NGHB#1272 70

CMPLX not

HVO_1298 - sensor box histidine kinase

HVO_1299 - cro/C1 family transcription regulator

//

>NGHB#1273 150

CMPLX not

HVO_1299 - cro/C1 family transcription regulator

HVO_1300 tpiA1 triosephosphate isomerase

//

>NGHB#1275 63

CMPLX not

HVO_1302 polY DNA-directed DNA polymerase Y

HVO_1301 - DUF87 domain protein

//

>NGHB#1276 122

CMPLX not

HVO_1303 - Lrp/AsnC family transcription regulator

HVO_1302 polY DNA-directed DNA polymerase Y

//

>NGHB#1277 117

CMPLX not

HVO_1304 porB pyruvate--ferredoxin oxidoreductase beta subunit

HVO_1303 - Lrp/AsnC family transcription regulator

//

>NGHB#1278 3

CMPLX yes

HVO_1305 porA pyruvate--ferredoxin oxidoreductase alpha subunit

HVO_1304 porB pyruvate--ferredoxin oxidoreductase beta subunit

//

>NGHB#1279 199

CMPLX not

HVO_1306 aroC chorismate synthase

HVO_1305 porA pyruvate--ferredoxin oxidoreductase alpha subunit

//

>NGHB#1282 66

CMPLX not

HVO_1308 aroA 3-phosphoshikimate 1-carboxyvinyltransferase

HVO_1307A - phosphodiesterase/nucleotide pyrophosphatase domain protein (nonfunctional)

//

>NGHB#1285 166

CMPLX ---

HVO_1311 - probable oxidoreductase (short-chain dehydrogenase family)

HVO_1310 - conserved hypothetical protein

//

>NGHB#1287 87

CMPLX not

HVO_1312 tyrA prephenate dehydrogenase

HVO_1313 - probable S-adenosylmethionine-dependent methyltransferase

//

>NGHB#1288 104

CMPLX not

HVO_1313 - probable S-adenosylmethionine-dependent methyltransferase

HVO_1314 surE1 5'-nucleotidase SurE

//

>NGHB#1289 310

CMPLX ---

HVO_1314 surE1 5'-nucleotidase SurE

HVO_1316 - conserved hypothetical protein

//

>NGHB#1293 96

CMPLX not

HVO_1320 - LysE family transport protein

HVO_1319 osmC peroxiredoxin OsmC, lipoyl-dependent

//

>NGHB#1294 372

CMPLX not

HVO_1322 aroQ chorismate mutase

HVO_1320 - LysE family transport protein

//

>NGHB#1295 -4

CMPLX not

HVO_1323 aroK shikimate kinase, archaeal-type

HVO_1322 aroQ chorismate mutase

//

>NGHB#1297 -1

CMPLX ---

HVO_1324 - conserved hypothetical protein

HVO_1325 - conserved hypothetical protein

//

>NGHB#1300 100

CMPLX ---

HVO_1327 cdc48c AAA-type ATPase (CDC48 subfamily)

HVO_1328 - hypothetical protein

//

>NGHB#1302 71

CMPLX ---

HVO_1330 - conserved hypothetical protein

HVO_1329 - probable S-adenosylmethionine-dependent methyltransferase

//

>NGHB#1304 91

CMPLX ---

HVO_1331 - ArsR family transcription regulator

HVO_1332 - conserved hypothetical protein

//

>NGHB#1307 -4

CMPLX ---

HVO_1334 - conserved hypothetical protein

HVO_1335 - conserved hypothetical protein

//

>NGHB#1309 4

CMPLX yes

HVO_1337 rpap1 rpa-associated protein

HVO_1336 rpe rpa-associated phosphoesterase

//

>NGHB#1310 -4

CMPLX yes

HVO_1338 rpa1 replication protein A

HVO_1337 rpap1 rpa-associated protein

//

>NGHB#1314 134

CMPLX not

HVO_1341 arfC 2,5-diamino-6-(ribosylamino)-4(3H)-pyrimidinone 5'-phosphate reductase

HVO_1342 trxA2 thioredoxin

//

>NGHB#1320 -4

CMPLX not

HVO_1348 nnrDE bifunctional NAD(P)H-hydrate repair enzyme Nnr

HVO_1347 moaC probable cyclic pyranopterin monophosphate synthase

//

>NGHB#1322 43

CMPLX not

HVO_1349 - major facilitator superfamily transport protein

HVO_1350 - ArsR family transcription regulator

//

>NGHB#1323 87

CMPLX not

HVO_1350 - ArsR family transcription regulator

HVO_1351 rad3a DNA repair helicase Rad3

//

>NGHB#1327 83

CMPLX not

HVO_1355 - UPF0058 family protein

HVO_1354 mutS5b DNA mismatch repair protein MutS

//

>NGHB#1329 141

CMPLX not

HVO_1356 - sensor box histidine kinase

HVO_1357 - receiver/bat box HTH-10 family transcription regulator

//

>NGHB#1331 94

CMPLX not

HVO_1359 - small CPxCG-related zinc finger protein

HVO_1358 - receiver box response regulator

//

>NGHB#1332 109

CMPLX not

HVO_1360 - TrmB family transcription regulator

HVO_1359 - small CPxCG-related zinc finger protein

//

>NGHB#1333 131

CMPLX not

HVO_1361 - TSUP family transport protein

HVO_1360 - TrmB family transcription regulator

//

>NGHB#1334 -4

CMPLX ---

HVO_1362 - conserved hypothetical protein

HVO_1361 - TSUP family transport protein

//

>NGHB#1335 495

CMPLX ---

HVO_1363 - DUF4341/DUF395 family protein

HVO_1362 - conserved hypothetical protein

//

>NGHB#1336 -4

CMPLX not

HVO_1364 - DUF4341/DUF395 family protein

HVO_1363 - DUF4341/DUF395 family protein

//

>NGHB#1337 0

CMPLX not

HVO_1365 - rhodanese domain protein / beta-lactamase domain protein

HVO_1364 - DUF4341/DUF395 family protein

//

>NGHB#1339 -4

CMPLX not

HVO_1366 - UPF0033 family protein

HVO_1367 - DsrE domain protein

//

>NGHB#1341 159

CMPLX not

HVO_1369 - DUF124 family protein

HVO_1368 - beta-lactamase domain protein

//

>NGHB#1342 105

CMPLX not

HVO_1370 proA gamma-glutamyl phosphate reductase

HVO_1369 - DUF124 family protein

//

>NGHB#1343 -8

CMPLX not

HVO_1371 proB glutamate 5-kinase

HVO_1370 proA gamma-glutamyl phosphate reductase

//

>NGHB#1344 -8

CMPLX not

HVO_1372 proC pyrroline-5-carboxylate reductase

HVO_1371 proB glutamate 5-kinase

//

>NGHB#1345 105

CMPLX not

HVO_1373 acd4 acyl-CoA dehydrogenase

HVO_1372 proC pyrroline-5-carboxylate reductase

//

>NGHB#1346 90

CMPLX not

HVO_1374 acs4 acyl-CoA synthetase

HVO_1373 acd4 acyl-CoA dehydrogenase

//

>NGHB#1347 92

CMPLX not

HVO_1375 menE o-succinylbenzoate--CoA ligase

HVO_1374 acs4 acyl-CoA synthetase

//

>NGHB#1348 99

CMPLX not

HVO_1376 mce methylmalonyl-CoA epimerase

HVO_1375 menE o-succinylbenzoate--CoA ligase

//

>NGHB#1350 7

CMPLX ---

HVO_1377 - UPF0145 family protein

HVO_1378 - conserved hypothetical protein

//

>NGHB#1352 307

CMPLX ---

HVO_1380 mmcA2 methylmalonyl-CoA mutase subunit A

HVO_1379 - conserved hypothetical protein

//

>NGHB#1354 82

CMPLX not

HVO_1381 - probable methyltransferase

HVO_1382 nac nascent polypeptide-associated complex protein

//

>NGHB#1355 -4

CMPLX not

HVO_1382 nac nascent polypeptide-associated complex protein

HVO_1383 trmI tRNA (adenine-N(1))-methyltransferase TrmI

//

>NGHB#1357 40

CMPLX ---

HVO_1385 tfs1, rpoM1 transcription elongation factor TFS

HVO_1384 - conserved hypothetical protein

//

>NGHB#1358 93

CMPLX ---

HVO_1386 - conserved hypothetical protein

HVO_1385 tfs1, rpoM1 transcription elongation factor TFS

//

>NGHB#1359 118

CMPLX ---

HVO_1387 fadA1 enoyl-CoA hydratase

HVO_1386 - conserved hypothetical protein

//

>NGHB#1362 25

CMPLX ---

HVO_1390 - conserved hypothetical protein

HVO_1389 - probable S-adenosylmethionine-dependent methyltransferase

//

>NGHB#1363 172

CMPLX ---

HVO_1391 - NUDIX family hydrolase

HVO_1390 - conserved hypothetical protein

//

>NGHB#1366 -4

CMPLX ---

HVO_1394 nhaC3 probable NhaC-type sodium/proton antiporter

HVO_1393 - conserved hypothetical protein

//

>NGHB#1368 129

CMPLX not

HVO_1395 - M20 family amidohydrolase (homolog to indole-3-acetyl-aspartate hydrolase)

HVO_1397 - histidine kinase

//

>NGHB#1370 139

CMPLX not

HVO_1398 tsgC11 ABC-type transport system permease protein (probable substrate sugar)

HVO_1396 - FAD-dependent oxidoreductase (homolog to geranylgeranyl reductase)

//

>NGHB#1371 -4

CMPLX yes

HVO_1399 tsgB11 ABC-type transport system permease protein (probable substrate sugar)

HVO_1398 tsgC11 ABC-type transport system permease protein (probable substrate sugar)

//

>NGHB#1372 -4

CMPLX yes

HVO_1400 tsgD11 ABC-type transport system ATP-binding protein (probable substrate sugar)

HVO_1399 tsgB11 ABC-type transport system permease protein (probable substrate sugar)

//

>NGHB#1373 223

CMPLX yes

HVO_1401 tsgA11 ABC-type transport system periplasmic substrate-binding protein (probable substrate sugar)

HVO_1400 tsgD11 ABC-type transport system ATP-binding protein (probable substrate sugar)

//

>NGHB#1374 184

CMPLX not

HVO_1402 pmm3 phosphohexomutase (phosphoglucomutase / phosphomannomutase)

HVO_1401 tsgA11 ABC-type transport system periplasmic substrate-binding protein (probable substrate sugar)

//

>NGHB#1376 79

CMPLX ---

HVO_1403 acyP acylphosphatase

HVO_1404 - conserved hypothetical protein

//

>NGHB#1378 345

CMPLX ---

HVO_1406 - DUF429 family protein

HVO_1405 - conserved hypothetical protein

//

>NGHB#1379 40

CMPLX not

HVO_1407 - HTH domain protein

HVO_1406 - DUF429 family protein

//

>NGHB#1381 145

CMPLX not

HVO_1408 - DoxX domain protein

HVO_1409 - YwrF family protein

//

>NGHB#1382 110

CMPLX ---

HVO_1409 - YwrF family protein

HVO_1410 - conserved hypothetical protein

//

>NGHB#1385 75

CMPLX not

HVO_1412 mvaD phosphomevalonate decarboxylase

HVO_1413 ndh2 probable NADH dehydrogenase

//

>NGHB#1386 165

CMPLX not

HVO_1413 ndh2 probable NADH dehydrogenase

HVO_1414 - sensor box histidine kinase

//

>NGHB#1387 87

CMPLX not

HVO_1414 - sensor box histidine kinase

HVO_1415 - HAD superfamily hydrolase

//

>NGHB#1388 91

CMPLX not

HVO_1415 - HAD superfamily hydrolase

HVO_1416 - APH family phosphotransferase

//

>NGHB#1389 107

CMPLX not

HVO_1416 - APH family phosphotransferase

HVO_1418 mc1A nonhistone chromosomal protein (nonfunctional)

//

>NGHB#1392 123

CMPLX not

HVO_1419 - HTH-10 family transcription regulator

HVO_1421 - chimeric protein (nonfunctional)

//

>NGHB#1394 -4

CMPLX not

HVO_1422_A - homolog to pHK2-ORF12

HVO_1422 - XerC/D-like integrase

//

>NGHB#1396 150

CMPLX ---

HVO_1423 - homolog to phage PhiH1 repressor protein

HVO_1424 - conserved hypothetical protein

//

>NGHB#1398 4

CMPLX ---

HVO_1425 - conserved hypothetical protein

HVO_1424A - homolog to virus protein HRPV1-VP8 (nonfunctional)

//

>NGHB#1399 281

CMPLX ---

HVO_1426 - homolog to virus protein HRPV1-VP8

HVO_1425 - conserved hypothetical protein

//

>NGHB#1400 121

CMPLX not

HVO_1427 - homolog to pHK2-ORF8

HVO_1426 - homolog to virus protein HRPV1-VP8

//

>NGHB#1401 -4

CMPLX not

HVO_1428 - homolog to pHK2-ORF7

HVO_1427 - homolog to pHK2-ORF8

//

>NGHB#1402 -4

CMPLX not

HVO_1429 - homolog to pHK2-ORF6

HVO_1428 - homolog to pHK2-ORF7

//

>NGHB#1403 34

CMPLX not

HVO_1430 - homolog to pHK2-ORF5

HVO_1429 - homolog to pHK2-ORF6

//

>NGHB#1404 1

CMPLX not

HVO_1431 - homolog to virus structural protein HRPV1-VP4

HVO_1430 - homolog to pHK2-ORF5

//

>NGHB#1405 69

CMPLX not

HVO_1432 - homolog to virus structural protein HRPV1-VP3

HVO_1431 - homolog to virus structural protein HRPV1-VP4

//

>NGHB#1406 -1

CMPLX not

HVO_1433 - homolog to pHK2-ORF2

HVO_1432 - homolog to virus structural protein HRPV1-VP3

//

>NGHB#1407 86

CMPLX not

HVO_1434 - homolog to HRPV1-ORF1

HVO_1433 - homolog to pHK2-ORF2

//

>NGHB#1408 66

CMPLX ---

HVO_1434A - conserved hypothetical protein

HVO_1434 - homolog to HRPV1-ORF1

//

>NGHB#1410 0

CMPLX ---

HVO_1436 ubiA2 (S)-2,3-di-O-geranylgeranylglyceryl phosphate synthase

HVO_1436A - conserved hypothetical protein

//

>NGHB#1411 -8

CMPLX ---

HVO_1436A - conserved hypothetical protein

HVO_1437 - conserved hypothetical protein

//

>NGHB#1412 101

CMPLX ---

HVO_1437 - conserved hypothetical protein

HVO_1438 - YneT family protein

//

>NGHB#1414 186

CMPLX ---

HVO_1440 - conserved hypothetical protein

HVO_1439 cysK1 cysteine synthase

//

>NGHB#1415 95

CMPLX ---

HVO_1441 - ABC-type transport system permease protein

HVO_1440 - conserved hypothetical protein

//

>NGHB#1416 -8

CMPLX yes

HVO_1442 - ABC-type transport system permease protein

HVO_1441 - ABC-type transport system permease protein

//

>NGHB#1417 -4

CMPLX yes

HVO_1443 - ABC-type transport system ATP-binding protein

HVO_1442 - ABC-type transport system permease protein

//

>NGHB#1418 147

CMPLX not

HVO_1444 hbd3 3-hydroxyacyl-CoA dehydrogenase

HVO_1443 - ABC-type transport system ATP-binding protein

//

>NGHB#1420 303

CMPLX ---

HVO_1445 - conserved hypothetical protein

HVO_1446 fbp1 fructose-1,6-bisphosphatase

//

>NGHB#1421 159

CMPLX not

HVO_1446 fbp1 fructose-1,6-bisphosphatase

HVO_1447 pccB1 propionyl-CoA carboxylase carboxyltransferase component

//

>NGHB#1422 188

CMPLX ---

HVO_1447 pccB1 propionyl-CoA carboxylase carboxyltransferase component

HVO_1449 - conserved hypothetical protein

//

>NGHB#1424 96

CMPLX not

HVO_1450 maoC1 MaoC domain protein

HVO_1448 - DoxX domain protein

//

>NGHB#1426 51

CMPLX not

HVO_1451 gdhA1 glutamate dehydrogenase (NAD)

HVO_1452 citE homolog to citrate lyase beta subunit

//

>NGHB#1427 162

CMPLX not

HVO_1452 citE homolog to citrate lyase beta subunit

HVO_1453 gdhA2 glutamate dehydrogenase (NADP)

//

>NGHB#1428 263

CMPLX not

HVO_1453 gdhA2 glutamate dehydrogenase (NADP)

HVO_1454 pyrB aspartate carbamoyltransferase catalytic subunit

//

>NGHB#1429 -4

CMPLX yes

HVO_1454 pyrB aspartate carbamoyltransferase catalytic subunit

HVO_1455 pyrI aspartate carbamoyltransferase regulatory subunit

//

>NGHB#1433 74

CMPLX ---

HVO_1459 - ThuA family protein

HVO_1458 - conserved hypothetical protein

//

>NGHB#1434 76

CMPLX not

HVO_1460 fadA2 enoyl-CoA hydratase

HVO_1459 - ThuA family protein

//

>NGHB#1435 65

CMPLX not

HVO_1461 menC o-succinylbenzoate synthase

HVO_1460 fadA2 enoyl-CoA hydratase

//

>NGHB#1436 -4

CMPLX not

HVO_1462 menA 1,4-dihydroxy-2-naphthoate polyprenyltransferase

HVO_1461 menC o-succinylbenzoate synthase

//

>NGHB#1437 176

CMPLX not

HVO_1463 trxB4 oxidoreductase (homolog to thioredoxin-disulfide reductase)

HVO_1462 menA 1,4-dihydroxy-2-naphthoate polyprenyltransferase

//

>NGHB#1438 1

CMPLX not

HVO_1464 - ABC-type transport system periplasmic substrate-binding protein

HVO_1463 trxB4 oxidoreductase (homolog to thioredoxin-disulfide reductase)

//

>NGHB#1439 144

CMPLX not

HVO_1465 menB 1,4-dihydroxy-2-naphthoyl-CoA synthase

HVO_1464 - ABC-type transport system periplasmic substrate-binding protein

//

>NGHB#1441 -8

CMPLX ---

HVO_1466 - CopD domain protein

HVO_1467 - conserved hypothetical protein

//

>NGHB#1443 79

CMPLX ---

HVO_1469 menD 2-succinyl-5-enolpyruvyl-6-hydroxy-3-cyclohexene-1-carboxylate synthase

HVO_1468 - conserved hypothetical protein

//

>NGHB#1444 87

CMPLX not

HVO_1470 menF isochorismate synthase

HVO_1469 menD 2-succinyl-5-enolpyruvyl-6-hydroxy-3-cyclohexene-1-carboxylate synthase

//

>NGHB#1445 175

CMPLX not

HVO_1471 - YuiH family molybdopterin-binding domain protein

HVO_1470 menF isochorismate synthase

//

>NGHB#1447 58

CMPLX ---

HVO_1472 - conserved hypothetical protein

HVO_1473 - UPF0058 family protein

//

>NGHB#1448 140

CMPLX not

HVO_1473 - UPF0058 family protein

HVO_1474 - probable rhomboid family protease

//

>NGHB#1450 -1

CMPLX ---

HVO_1476 - conserved hypothetical protein

HVO_1475 - probable rRNA methyltransferase

//

>NGHB#1451 244

CMPLX ---

HVO_1477 - eDNA utilization nuclease

HVO_1476 - conserved hypothetical protein

//

>NGHB#1455 34

CMPLX ---

HVO_1480 - conserved hypothetical protein

HVO_1481 - UspA domain protein

//

>NGHB#1456 107

CMPLX not

HVO_1481 - UspA domain protein

HVO_1482 ferA2 ferredoxin (2Fe-2S)

//

>NGHB#1458 64

CMPLX not

HVO_1484 hemAT1, htr10a transducer protein HemAT

HVO_1483 - rhodanese domain protein / beta-lactamase domain protein (nonfunctional)

//

>NGHB#1460 100

CMPLX not

HVO_1485 - ferritin domain protein

HVO_1486 - MOSC domain protein

//

>NGHB#1461 -4

CMPLX not

HVO_1486 - MOSC domain protein

HVO_1487 galK galactokinase

//

>NGHB#1463 84

CMPLX ---

HVO_1489 - conserved hypothetical protein

HVO_1488 gnaD, iftA D-gluconate dehydratase

//

>NGHB#1468 200

CMPLX ---

HVO_1494 fba1 fructose-bisphosphate aldolase, class 2

HVO_1493 - conserved hypothetical protein

//

>NGHB#1469 165

CMPLX not

HVO_1495 ptfB phosphotransferase system component IIB, fructose-specific

HVO_1494 fba1 fructose-bisphosphate aldolase, class 2

//

>NGHB#1470 -4

CMPLX yes

HVO_1496 ptsI phosphotransferase system component I

HVO_1495 ptfB phosphotransferase system component IIB, fructose-specific

//

>NGHB#1471 3

CMPLX yes

HVO_1497 ptsH1 phosphocarrier protein HPr

HVO_1496 ptsI phosphotransferase system component I

//

>NGHB#1472 -1

CMPLX yes

HVO_1498 ptfA phosphotransferase system component IIA, fructose-specific

HVO_1497 ptsH1 phosphocarrier protein HPr

//

>NGHB#1473 44

CMPLX yes

HVO_1499 ptfC phosphotransferase system component IIC, fructose-specific

HVO_1498 ptfA phosphotransferase system component IIA, fructose-specific

//

>NGHB#1474 105

CMPLX not

HVO_1500 pfkB 1-phosphofructokinase

HVO_1499 ptfC phosphotransferase system component IIC, fructose-specific

//

>NGHB#1475 -4

CMPLX not

HVO_1501 glpR DeoR family transcription regulator GlpR

HVO_1500 pfkB 1-phosphofructokinase

//

>NGHB#1476 203

CMPLX not

HVO_1502 leuB 3-isopropylmalate dehydrogenase

HVO_1501 glpR DeoR family transcription regulator GlpR

//

>NGHB#1477 -8

CMPLX not

HVO_1503 leuD1 3-isopropylmalate dehydratase small subunit

HVO_1502 leuB 3-isopropylmalate dehydrogenase

//

>NGHB#1478 -4

CMPLX yes

HVO_1504 leuC1 3-isopropylmalate dehydratase large subunit

HVO_1503 leuD1 3-isopropylmalate dehydratase small subunit

//

>NGHB#1479 -8

CMPLX ---

HVO_1505 - conserved hypothetical protein

HVO_1504 leuC1 3-isopropylmalate dehydratase large subunit

//

>NGHB#1480 11

CMPLX ---

HVO_1506 ilvC ketol-acid reductoisomerase

HVO_1505 - conserved hypothetical protein

//

>NGHB#1481 -4

CMPLX not

HVO_1507 ilvN acetolactate synthase small subunit

HVO_1506 ilvC ketol-acid reductoisomerase

//

>NGHB#1482 -4

CMPLX yes

HVO_1508 ilvB1 acetolactate synthase large subunit

HVO_1507 ilvN acetolactate synthase small subunit

//

>NGHB#1483 142

CMPLX ---

HVO_1509 - conserved hypothetical protein

HVO_1508 ilvB1 acetolactate synthase large subunit

//

>NGHB#1484 120

CMPLX ---

HVO_1510 leuA2 2-isopropylmalate synthase

HVO_1509 - conserved hypothetical protein

//

>NGHB#1486 115

CMPLX ---

HVO_1512 - conserved hypothetical protein

HVO_1513 - beta-lactamase domain protein

//

>NGHB#1487 -1

CMPLX ---

HVO_1513 - beta-lactamase domain protein

HVO_1514 - conserved hypothetical protein

//

>NGHB#1492 102

CMPLX not

HVO_1523 aglQ N-glycosylation pathway protein AglQ

HVO_1522 aglP hexuronic acid methyltransferase AglP

//

>NGHB#1493 -4

CMPLX not

HVO_1523_A aglE glycosyltransferase AglE

HVO_1523 aglQ N-glycosylation pathway protein AglQ

//

>NGHB#1494 52

CMPLX not

HVO_1524 aglR probable flippase AglR

HVO_1523_A aglE glycosyltransferase AglE

//

>NGHB#1495 217

CMPLX not

HVO_1525 - ISH3-type transposase ISH51

HVO_1524 aglR probable flippase AglR

//

>NGHB#1497 168

CMPLX not

HVO_1526 aglS dolichyl-monophosphomannose--protein mannosyltransferase AglS

HVO_1527 aglF UTP--glucose-1-phosphate uridylyltransferase AglF

//

>NGHB#1498 48

CMPLX not

HVO_1527 aglF UTP--glucose-1-phosphate uridylyltransferase AglF

HVO_1528 aglI glycosyltransferase AglI

//

>NGHB#1504 167

CMPLX not

HVO_1534 - probable S-adenosylmethionine-dependent methyltransferase

HVO_1533 - small CPxCG-related zinc finger protein

//

>NGHB#1507 108

CMPLX not

HVO_1537 orc15 Orc1-type DNA replication protein

HVO_1536 - phosphoglycolate phosphatase

//

>NGHB#1509 -14

CMPLX yes

HVO_1538 glpA1 glycerol-3-phosphate dehydrogenase subunit A

HVO_1539 glpB1 glycerol-3-phosphate dehydrogenase subunit B

//

>NGHB#1510 -4

CMPLX yes

HVO_1539 glpB1 glycerol-3-phosphate dehydrogenase subunit B

HVO_1540 glpC1 glycerol-3-phosphate dehydrogenase subunit C

//

>NGHB#1511 363

CMPLX not

HVO_1540 glpC1 glycerol-3-phosphate dehydrogenase subunit C

HVO_1541 glpK glycerol kinase

//

>NGHB#1512 3

CMPLX ---

HVO_1541 glpK glycerol kinase

HVO_1542 - conserved hypothetical protein

//

>NGHB#1513 97

CMPLX ---

HVO_1542 - conserved hypothetical protein

HVO_1543 ptsH2 phosphocarrier protein HPr

//

>NGHB#1515 -4

CMPLX not

HVO_1545 dhaL phosphoenolpyruvate--dihydroxyacetone phosphotransferase subunit DhaL

HVO_1544 dhaM probable phosphoenolpyruvate-protein phosphoryltransferase

//

>NGHB#1516 123

CMPLX yes

HVO_1546 dhaK phosphoenolpyruvate--dihydroxyacetone phosphotransferase subunit DhaK

HVO_1545 dhaL phosphoenolpyruvate--dihydroxyacetone phosphotransferase subunit DhaL

//

>NGHB#1517 165

CMPLX not

HVO_1547 ileS isoleucine--tRNA ligase

HVO_1546 dhaK phosphoenolpyruvate--dihydroxyacetone phosphotransferase subunit DhaK

//

>NGHB#1518 380

CMPLX ---

HVO_1548 - conserved hypothetical protein

HVO_1547 ileS isoleucine--tRNA ligase

//

>NGHB#1519 57

CMPLX ---

HVO_1549 - conserved hypothetical protein

HVO_1548 - conserved hypothetical protein

//

>NGHB#1520 63

CMPLX ---

HVO_1550 - thioesterase domain protein

HVO_1549 - conserved hypothetical protein

//

>NGHB#1521 60

CMPLX not

HVO_1551 - DUF151 family protein

HVO_1550 - thioesterase domain protein

//

>NGHB#1522 81

CMPLX not

HVO_1552 - ArsR family transcription regulator

HVO_1551 - DUF151 family protein

//

>NGHB#1523 63

CMPLX not

HVO_1553 - glycerate 2-kinase

HVO_1552 - ArsR family transcription regulator

//

>NGHB#1525 -4

CMPLX not

HVO_1554 traB TraB family protein

HVO_1555 - M50 family metalloprotease

//

>NGHB#1528 72

CMPLX not

HVO_1557 purM phosphoribosylformylglycinamidine cyclo-ligase

HVO_1558 cyc2 cytochrome P450

//

>NGHB#1530 67

CMPLX not

HVO_1560 - UPF0212 family protein

HVO_1559 - HTH/CBS domain protein

//

>NGHB#1532 148

CMPLX ---

HVO_1561 - conserved hypothetical protein

HVO_1562 psmB proteasome beta subunit

//

>NGHB#1534 111

CMPLX ---

HVO_1564 - UCP012666 family protein

HVO_1563 - conserved hypothetical protein

//

>NGHB#1536 64

CMPLX ---

HVO_1565 ligA DNA ligase (ATP)

HVO_1566 - conserved hypothetical protein

//

>NGHB#1537 128

CMPLX ---

HVO_1566 - conserved hypothetical protein

HVO_1567 - metal-dependent hydrolase domain protein

//

>NGHB#1538 101

CMPLX not

HVO_1567 - metal-dependent hydrolase domain protein

HVO_1568 - alpha/beta hydrolase fold protein

//

>NGHB#1539 89

CMPLX ---

HVO_1568 - alpha/beta hydrolase fold protein

HVO_1569 - hypothetical protein

//

>NGHB#1541 -1

CMPLX yes

HVO_1571top6B DNA topoisomerase 6 subunit B

HVO_1570 top6A DNA topoisomerase 6 subunit A

//

>NGHB#1543 1

CMPLX yes

HVO_1572 gyrB DNA gyrase subunit B

HVO_1573 gyrA DNA gyrase subunit A

//

>NGHB#1544 101

CMPLX ---

HVO_1573 gyrA DNA gyrase subunit A

HVO_1573A - conserved hypothetical protein

//

>NGHB#1546 -1

CMPLX not

HVO_1575 rocF arginase

HVO_1574 - NUDIX family hydrolase

//

>NGHB#1547 104

CMPLX not

HVO_1576 - GalE family epimerase/dehydratase

HVO_1575 rocF arginase

//

>NGHB#1550 114

CMPLX not

HVO_1579 udp1 uridine phosphorylase

HVO_1578 ndh1 probable NADH dehydrogenase

//

>NGHB#1551 4

CMPLX not

HVO_1580 - peptidase M10 family protein

HVO_1579 udp1 uridine phosphorylase

//

>NGHB#1552 1

CMPLX not

HVO_1581 cdd cytidine deaminase

HVO_1580 - peptidase M10 family protein

//

>NGHB#1554 96

CMPLX ---

HVO_1582 - conserved hypothetical protein

HVO_1583 - conserved hypothetical protein

//

>NGHB#1558 143

CMPLX ---

HVO_1587 - conserved hypothetical protein

HVO_1586 gltP1 SDF family transport protein (probable substrate glutamate/aspartate)

//

>NGHB#1559 108

CMPLX ---

HVO_1588 - cupin 2 barrel domain protein

HVO_1587 - conserved hypothetical protein

//

>NGHB#1560 52

CMPLX not

HVO_1589 dnaJ molecular chaperone DnaJ

HVO_1588 - cupin 2 barrel domain protein

//

>NGHB#1561 144

CMPLX yes

HVO_1590 dnaK Hsp70-type molecular chaperone DnaK

HVO_1589 dnaJ molecular chaperone DnaJ

//

>NGHB#1562 221

CMPLX not

HVO_1591 - alpha/beta hydrolase fold protein

HVO_1590 dnaK Hsp70-type molecular chaperone DnaK

//

>NGHB#1563 93

CMPLX not

HVO_1592 grpE DnaJ/DnaK ATPase stimulator GrpE

HVO_1591 - alpha/beta hydrolase fold protein

//

>NGHB#1564 126

CMPLX not

HVO_1593 - PAC2 family protein

HVO_1592 grpE DnaJ/DnaK ATPase stimulator GrpE

//

>NGHB#1566 -4

CMPLX ---

HVO_1594 cna tRNA/rRNA cytosine-C5-methylase

HVO_1595 - conserved hypothetical protein

//

>NGHB#1572 34

CMPLX ---

HVO_1601 ssuB ABC-type transport system ATP-binding protein (probable substrate nitrate/sulfonate/bicarbonate)

HVO_1600 - conserved hypothetical protein

//

>NGHB#1573 -4

CMPLX yes

HVO_1602 ssuC ABC-type transport system permease protein (probable substrate nitrate/sulfonate/bicarbonate)

HVO_1601 ssuB ABC-type transport system ATP-binding protein (probable substrate nitrate/sulfonate/bicarbonate)

//

>NGHB#1574 -4

CMPLX yes

HVO_1603 ssuA ABC-type transport system periplasmic substrate-binding protein (probable substrate nitrate/sulfonate/bicarbonate)

HVO_1602 ssuC ABC-type transport system permease protein (probable substrate nitrate/sulfonate/bicarbonate)

//

>NGHB#1576 130

CMPLX not

HVO_1604 aspC1 pyridoxal phosphate-dependent aminotransferase

HVO_1605 nhaC5 probable NhaC-type sodium/proton antiporter

//

>NGHB#1580 82

CMPLX not

HVO_1609 - DUF4349 domain protein

HVO_1608 - GTP-binding protein

//

>NGHB#1581 122

CMPLX not

HVO_1610 - DUF583 domain protein

HVO_1609 - DUF4349 domain protein

//

>NGHB#1584 119

CMPLX not

HVO_1613 - dolichyl-phosphate hexosyltransferase

HVO_1612 - DUF3179 family protein

//

>NGHB#1585 75

CMPLX ---

HVO_1614 - conserved hypothetical protein

HVO_1613 - dolichyl-phosphate hexosyltransferase

//

>NGHB#1586 102

CMPLX ---

HVO_1615 - ribonuclease H domain protein

HVO_1614 - conserved hypothetical protein

//

>NGHB#1588 6

CMPLX ---

HVO_1616 - UPF0361 family protein

HVO_1617 - hypothetical protein

//

>NGHB#1589 142

CMPLX ---

HVO_1617 - hypothetical protein

HVO_1618 - hypothetical protein

//

>NGHB#1590 824

CMPLX ---

HVO_1618 - hypothetical protein

HVO_1619 - conserved hypothetical protein

//

>NGHB#1591 -11

CMPLX ---

HVO_1619 - conserved hypothetical protein

HVO_1620 - XerC/D-like integrase

//

>NGHB#1594 42

CMPLX ---

HVO_1622 - ArsR family transcription regulator

HVO_1624 - conserved hypothetical protein

//

>NGHB#1596 75

CMPLX ---

HVO_1625 - conserved hypothetical protein

HVO_1623 - conserved hypothetical protein

//

>NGHB#1597 153

CMPLX ---

HVO_1626 - metal-dependent hydrolase domain protein

HVO_1625 - conserved hypothetical protein

//

>NGHB#1599 153

CMPLX ---

HVO_1627 - conserved hypothetical protein

HVO_1628 - TIGR00725 family protein

//

>NGHB#1601 79

CMPLX ---

HVO_1630 - conserved hypothetical protein

HVO_1629 - conserved hypothetical protein

//

>NGHB#1602 163

CMPLX ---

HVO_1631 dph2 2-(3-amino-3-carboxypropyl)histidine synthase

HVO_1630 - conserved hypothetical protein

//

>NGHB#1604 81

CMPLX not

HVO_1632 - alpha/beta hydrolase fold protein

HVO_1633 - DUF964 family protein

//

>NGHB#1605 73

CMPLX not

HVO_1633 - DUF964 family protein

HVO_1634 minD3 MinD/ParA domain protein

//

>NGHB#1606 26

CMPLX ---

HVO_1634 minD3 MinD/ParA domain protein

HVO_1635 - conserved hypothetical protein

//

>NGHB#1608 73

CMPLX ---

HVO_1637 mtfK2 FKBP-type peptidylprolyl isomerase

HVO_1636 - conserved hypothetical protein

//

>NGHB#1610 1

CMPLX ---

HVO_1638 - conserved hypothetical protein

HVO_1639 - conserved hypothetical protein (nonfunctional)

//

>NGHB#1611 17

CMPLX ---

HVO_1639 - conserved hypothetical protein (nonfunctional)

HVO_1640 - conserved hypothetical protein (nonfunctional)

//

>NGHB#1612 20

CMPLX ---

HVO_1640 - conserved hypothetical protein (nonfunctional)

HVO_1640A - conserved hypothetical protein

//

>NGHB#1614 176

CMPLX ---

HVO_1642 - conserved hypothetical protein

HVO_1641 - conserved hypothetical protein

//

>NGHB#1615 216

CMPLX ---

HVO_1643 - conserved hypothetical protein

HVO_1642 - conserved hypothetical protein

//

>NGHB#1616 -4

CMPLX ---

HVO_1644 - terminal oxidase subunit IV domain protein

HVO_1643 - conserved hypothetical protein

//

>NGHB#1617 10

CMPLX yes

HVO_1645 coxAC2 cox-type terminal oxidase subunit I/III

HVO_1644 - terminal oxidase subunit IV domain protein

//

>NGHB#1618 -4

CMPLX yes

HVO_1646 coxB2 cox-type terminal oxidase subunit II

HVO_1645 coxAC2 cox-type terminal oxidase subunit I/III

//

>NGHB#1619 210

CMPLX ---

HVO_1647 - conserved hypothetical protein

HVO_1646 coxB2 cox-type terminal oxidase subunit II

//

>NGHB#1621 127

CMPLX not

HVO_1648 - adenylate cyclase domain protein

HVO_1649 mat S-adenosylmethionine synthase

//

>NGHB#1624 196

CMPLX ---

HVO_1651 thiI tRNA uracil 4-sulfurtransferase

HVO_1652 - conserved hypothetical protein

//

>NGHB#1625 42

CMPLX ---

HVO_1652 - conserved hypothetical protein

HVO_1653 - conserved hypothetical protein

//

>NGHB#1630 90

CMPLX not

HVO_1657 purD phosphoribosylamine--glycine ligase

HVO_1658 yvoF O-acetyltransferase (homolog to galactoside O-acetyltransferase)

//

>NGHB#1632 1

CMPLX not

HVO_1660 dacZ diadenylate cyclase

HVO_1659 mscS2 mechanosensitive channel protein MscS

//

>NGHB#1633 116

CMPLX not

HVO_1661 - globin family protein

HVO_1660 dacZ diadenylate cyclase

//

>NGHB#1635 337

CMPLX ---

HVO_1662 - conserved hypothetical protein

HVO_1664 - cyclin domain protein

//

>NGHB#1637 29

CMPLX ---

HVO_1666 coaD phosphopantetheine adenylyltransferase

HVO_1665 - conserved hypothetical protein

//

>NGHB#1638 117

CMPLX not

HVO_1667 - TrmB family transcription regulator

HVO_1666 coaD phosphopantetheine adenylyltransferase

//

>NGHB#1639 67

CMPLX not

HVO_1668 gshA glutamate--cysteine ligase

HVO_1667 - TrmB family transcription regulator

//

>NGHB#1640 168

CMPLX not

HVO_1669 fib fibrillarin-like rRNA/tRNA 2'-O-methyltransferase

HVO_1668 gshA glutamate--cysteine ligase

//

>NGHB#1641 -4

CMPLX not

HVO_1670 nop5 rRNA/tRNA 2'-O-methyltransferase complex protein Nop5

HVO_1669 fib fibrillarin-like rRNA/tRNA 2'-O-methyltransferase

//

>NGHB#1643 38

CMPLX ---

HVO_1671 - conserved hypothetical protein

HVO_1672 - conserved hypothetical protein

//

>NGHB#1644 103

CMPLX ---

HVO_1672 - conserved hypothetical protein

HVO_1673 - conserved hypothetical protein

//

>NGHB#1645 60

CMPLX ---

HVO_1673 - conserved hypothetical protein

HVO_1674 - conserved hypothetical protein

//

>NGHB#1646 -4

CMPLX ---

HVO_1674 - conserved hypothetical protein

HVO_1675 corA magnesium transport protein CorA

//

>NGHB#1647 221

CMPLX not

HVO_1675 corA magnesium transport protein CorA

HVO_1676 tfb2 transcription initiation factor TFB

//

>NGHB#1648 98

CMPLX not

HVO_1676 tfb2 transcription initiation factor TFB

HVO_1677 - small CPxCG-related zinc finger protein

//

>NGHB#1652 61

CMPLX not

HVO_1681 - DNA N-glycosylase

HVO_1680 - UPF0212 family protein

//

>NGHB#1653 113

CMPLX not

HVO_1682 mscS7 mechanosensitive channel protein MscS

HVO_1681 - DNA N-glycosylase

//

>NGHB#1654 66

CMPLX not

HVO_1683 malQ 4-alpha-glucanotransferase

HVO_1682 mscS7 mechanosensitive channel protein MscS

//

>NGHB#1657 123

CMPLX ---

HVO_1686 - major facilitator superfamily transport protein

HVO_1685 - conserved hypothetical protein

//

>NGHB#1662 182

CMPLX not

HVO_1692 - probable iron-sulfur protein (4Fe-4S)

HVO_1691 - PRC domain protein

//

>NGHB#1663 -8

CMPLX not

HVO_1693 - DUF162 family protein

HVO_1692 - probable iron-sulfur protein (4Fe-4S)

//

>NGHB#1664 78

CMPLX ---

HVO_1694 - conserved hypothetical protein

HVO_1693 - DUF162 family protein

//

>NGHB#1665 122

CMPLX ---

HVO_1695 - HTH domain protein

HVO_1694 - conserved hypothetical protein

//

>NGHB#1667 223

CMPLX not

HVO_1696 - LctP family transport protein

HVO_1697 - FAD-dependent oxidoreductase (GlcD/DLD_GlcF/GlpC domain fusion protein)

//

>NGHB#1668 139

CMPLX not

HVO_1697 - FAD-dependent oxidoreductase (GlcD/DLD_GlcF/GlpC domain fusion protein)

HVO_1698 - CBS/parB domain protein

//

>NGHB#1670 70

CMPLX not

HVO_1700 - HTH domain protein

HVO_1699 - pyridoxal phosphate-dependent aminotransferase (homolog to histidinol-phosphate aminotransferase / aspartate aminotransferase)

//

>NGHB#1671 222

CMPLX not

HVO_1701 - DUF123 domain protein

HVO_1700 - HTH domain protein

//

>NGHB#1672 22

CMPLX ---

HVO_1702 - conserved hypothetical protein

HVO_1701 - DUF123 domain protein

//

>NGHB#1674 127

CMPLX not

HVO_1703 hel2 probable DEAD/DEAH box helicase

HVO_1704 - homolog to arabinopyranose mutase

//

>NGHB#1675 122

CMPLX not

HVO_1704 - homolog to arabinopyranose mutase

HVO_1705 - ABC-type transport system periplasmic substrate-binding protein

//

>NGHB#1676 12

CMPLX yes

HVO_1705 - ABC-type transport system periplasmic substrate-binding protein

HVO_1706 - ABC-type transport system permease protein

//

>NGHB#1677 -11

CMPLX yes

HVO_1706 - ABC-type transport system permease protein

HVO_1707 - ABC-type transport system ATP-binding protein

//

>NGHB#1678 2

CMPLX ---

HVO_1707 - ABC-type transport system ATP-binding protein

HVO_1708 - conserved hypothetical protein

//

>NGHB#1680 630

CMPLX not

HVO_1710 amyA2 glycoside hydrolase domain protein

HVO_1709 nasB, narK major facilitator superfamily transport protein (probable substrate nitrate/nitrite) (nonfunctional)

//

>NGHB#1682 129

CMPLX not

HVO_1711 sga1 probable glucoamylase

HVO_1712 - START domain protein

//

>NGHB#1684 138

CMPLX not

HVO_1714 - DMT superfamily transport protein

HVO_1713 - receiver/sensor box histidine kinase

//

>NGHB#1685 78

CMPLX not

HVO_1715 - probable S-adenosylmethionine-dependent methyltransferase

HVO_1714 - DMT superfamily transport protein

//

>NGHB#1686 128

CMPLX not

HVO_1716 queC 7-cyano-7-deazaguanine synthase

HVO_1715 - probable S-adenosylmethionine-dependent methyltransferase

//

>NGHB#1687 102

CMPLX not

HVO_1717 queE 7-carboxy-7-deazaguanine synthase

HVO_1716 queC 7-cyano-7-deazaguanine synthase

//

>NGHB#1688 -1

CMPLX not

HVO_1718 queD 6-carboxy-5,6,7,8-tetrahydropterin synthase

HVO_1717 queE 7-carboxy-7-deazaguanine synthase

//

>NGHB#1689 115

CMPLX not

HVO_1719 - thioesterase domain protein

HVO_1718 queD 6-carboxy-5,6,7,8-tetrahydropterin synthase

//

>NGHB#1690 117

CMPLX ---

HVO_1720 - conserved hypothetical protein

HVO_1719 - thioesterase domain protein

//

>NGHB#1693 229

CMPLX ---

HVO_1724 - conserved hypothetical protein

HVO_1723 rad25d DNA repair helicase Rad25

//

>NGHB#1696 85

CMPLX not

HVO_1727 tbp2 TATA-binding transcription initiation factor

HVO_1726 - HTH domain protein

//

>NGHB#1698 -4

CMPLX ---

HVO_1733 - HTH domain protein

HVO_1734 - conserved hypothetical protein

//

>NGHB#1700 67

CMPLX not

HVO_1735 - SWIM zinc finger domain protein

HVO_1734A - restriction endonuclease domain protein

//

>NGHB#1702 -14

CMPLX not

HVO_1736 rad25b DNA repair helicase Rad25

HVO_1737 - DUF790 family protein

//

>NGHB#1704 -4

CMPLX ---

HVO_1739 - conserved hypothetical protein

HVO_1738 - CcbP family protein

//

>NGHB#1705 6

CMPLX ---

HVO_1740 - conserved hypothetical protein

HVO_1739 - conserved hypothetical protein

//

>NGHB#1706 59

CMPLX ---

HVO_1741 - conserved hypothetical protein

HVO_1740 - conserved hypothetical protein

//

>NGHB#1707 75

CMPLX ---

HVO_1742 - conserved hypothetical protein

HVO_1741 - conserved hypothetical protein

//

>NGHB#1708 81

CMPLX ---

HVO_1743 - SWIM zinc finger domain protein

HVO_1742 - conserved hypothetical protein

//

>NGHB#1709 32

CMPLX not

HVO_1744 hel1 probable DEAD/DEAH box helicase

HVO_1743 - SWIM zinc finger domain protein

//

>NGHB#1711 48

CMPLX ---

HVO_1745 - conserved hypothetical protein

HVO_1746 - DUF2800 family protein

//

>NGHB#1714 132

CMPLX ---

HVO_1751 copA P-type transport ATPase (probable substrate copper/metal cation)

HVO_1750 - conserved hypothetical protein

//

>NGHB#1715 141

CMPLX not

HVO_1752 - Lrp/AsnC family transcription regulator

HVO_1751 copA P-type transport ATPase (probable substrate copper/metal cation)

//

>NGHB#1717 122

CMPLX ---

HVO_1753 - HMA domain protein

HVO_1754 - conserved hypothetical protein

//

>NGHB#1718 108

CMPLX ---

HVO_1754 - conserved hypothetical protein

HVO_1755 - conserved hypothetical protein

//

>NGHB#1721 226

CMPLX not

HVO_1757 - DoxX domain protein

HVO_1758 trxB5 oxidoreductase (homolog to thioredoxin-disulfide reductase)

//

>NGHB#1722 -4

CMPLX not

HVO_1758 trxB5 oxidoreductase (homolog to thioredoxin-disulfide reductase)

HVO_1759 - ABC-type transport system permease protein

//

>NGHB#1723 -1

CMPLX yes

HVO_1759 - ABC-type transport system permease protein

HVO_1760 - ABC-type transport system ATP-binding protein

//

>NGHB#1725 63

CMPLX not

HVO_1762 - DnaJ N-terminal domain / ferredoxin fusion protein

HVO_1761 mtfK3 FKBP-type peptidylprolyl isomerase

//

>NGHB#1729 33

CMPLX not

HVO_1766 - ArsR family transcription regulator

HVO_1767 dppB3 ABC-type transport system permease protein (probable substrate dipeptide/oligopeptide)

//

>NGHB#1730 2

CMPLX yes

HVO_1767 dppB3 ABC-type transport system permease protein (probable substrate dipeptide/oligopeptide)

HVO_1768 dppD3 ABC-type transport system ATP-binding protein (probable substrate dipeptide/oligopeptide)

//

>NGHB#1731 -4

CMPLX yes

HVO_1768 dppD3 ABC-type transport system ATP-binding protein (probable substrate dipeptide/oligopeptide)

HVO_1769 dppF3 ABC-type transport system ATP-binding protein (probable substrate dipeptide/oligopeptide)

//

>NGHB#1732 -4

CMPLX ---

HVO_1769 dppF3 ABC-type transport system ATP-binding protein (probable substrate dipeptide/oligopeptide)

HVO_1770 - hypothetical protein

//

>NGHB#1733 -4

CMPLX ---

HVO_1770 - hypothetical protein

HVO_1771 - DUF917 family protein

//

>NGHB#1734 8

CMPLX not

HVO_1771 - DUF917 family protein

HVO_1772 - DUF1177 family protein

//

>NGHB#1735 1

CMPLX not

HVO_1772 - DUF1177 family protein

HVO_1773 - AroM family protein

//

>NGHB#1736 0

CMPLX not

HVO_1773 - AroM family protein

HVO_1774 pepB4 aminopeptidase (homolog to leucyl aminopeptidase / aminopeptidase T)

//

>NGHB#1737 -4

CMPLX not

HVO_1774 pepB4 aminopeptidase (homolog to leucyl aminopeptidase / aminopeptidase T)

HVO_1775 - nitroreductase family protein

//

>NGHB#1738 -1

CMPLX not

HVO_1775 - nitroreductase family protein

HVO_1776 - homolog to hydantoin racemase

//

>NGHB#1739 310

CMPLX not

HVO_1776 - homolog to hydantoin racemase

HVO_1778 katG catalase-peroxidase

//

>NGHB#1742 4

CMPLX ---

HVO_1780 - HTH domain protein

HVO_1781 - conserved hypothetical protein

//

>NGHB#1746 96

CMPLX ---

HVO_1785 - conserved hypothetical protein

HVO_1783 - conserved hypothetical protein

//

>NGHB#1747 -4

CMPLX ---

HVO_1786 - conserved hypothetical protein

HVO_1785 - conserved hypothetical protein

//

>NGHB#1749 88

CMPLX ---

HVO_1787 - conserved hypothetical protein

HVO_1788 nirA1 probable sulfite/nitrite reductase (ferredoxin)

//

>NGHB#1750 66

CMPLX ---

HVO_1788 nirA1 probable sulfite/nitrite reductase (ferredoxin)

HVO_1789 - conserved hypothetical protein

//

>NGHB#1751 318

CMPLX ---

HVO_1789 - conserved hypothetical protein

HVO_1790 - DMT superfamily transport protein

//

>NGHB#1754 44

CMPLX ---

HVO_1792 - Lrp/AsnC family transcription regulator

HVO_1792A - conserved hypothetical protein

//

>NGHB#1759 38

CMPLX ---

HVO_1800 - hypothetical protein

HVO_1799 - digeranylgeranylglycerophospholipid reductase / dolichol omega-reductase

//

>NGHB#1760 59

CMPLX ---

HVO_1801 - conserved hypothetical protein

HVO_1800 - hypothetical protein

//

>NGHB#1761 105

CMPLX ---

HVO_1802 - peptidase M10 family protein

HVO_1801 - conserved hypothetical protein

//

>NGHB#1764 77

CMPLX ---

HVO_1805 - conserved hypothetical protein

HVO_1804 pncB nicotinate phosphoribosyltransferase

//

>NGHB#1765 37

CMPLX ---

HVO_1806 - conserved hypothetical protein

HVO_1805 - conserved hypothetical protein

//

>NGHB#1766 44

CMPLX ---

HVO_1807 - conserved hypothetical protein

HVO_1806 - conserved hypothetical protein

//

>NGHB#1767 96

CMPLX ---

HVO_1808 - conserved hypothetical protein

HVO_1807 - conserved hypothetical protein

//

>NGHB#1771 60

CMPLX not

HVO_1811 - sensor box histidine kinase

HVO_1812 - probable ketohexokinase

//

>NGHB#1773 222

CMPLX not

HVO_1814 - peptidase M23 family protein

HVO_1813 - HTH-10 family transcription regulator

//

>NGHB#1775 64

CMPLX not

HVO_1815 - lipoate--protein ligase domain protein

HVO_1816 pyrC dihydroorotase

//

>NGHB#1780 -4

CMPLX not

HVO_1820 - UspA domain protein

HVO_1821 pat2 GNAT family acetyltransferase Pat2

//

>NGHB#1783 103

CMPLX ---

HVO_1823 - UspA domain protein

HVO_1824 - conserved hypothetical protein

//

>NGHB#1785 54

CMPLX ---

HVO_1826 - conserved hypothetical protein

HVO_1825 - conserved hypothetical protein

//

>NGHB#1786 24

CMPLX ---

HVO_1827 rps6e 30S ribosomal protein S6e

HVO_1826 - conserved hypothetical protein

//

>NGHB#1790 180

CMPLX not

HVO_1830 gndA 6-phosphogluconate dehydrogenase (NAD-dependent, decarboxylating)

HVO_1831 ferA4 ferredoxin (2Fe-2S)

//

>NGHB#1791 189

CMPLX not

HVO_1831 ferA4 ferredoxin (2Fe-2S)

HVO_1832 - MATE efflux family protein

//

>NGHB#1794 88

CMPLX not

HVO_1840 - small CPxCG-related zinc finger protein

HVO_1841 - homolog to HGPV1-ORF14

//

>NGHB#1795 316

CMPLX not

HVO_1841 - homolog to HGPV1-ORF14

HVO_1841_A - death-on-curing family protein

//

>NGHB#1801 82

CMPLX not

HVO_1848_A - small CPxCG-related zinc finger protein

HVO_1847 pepF oligoendopeptidase PepF

//

>NGHB#1804 119

CMPLX ---

HVO_1851 - HAD superfamily hydrolase

HVO_1850 - conserved hypothetical protein

//

>NGHB#1805 87

CMPLX not

HVO_1852 truA tRNA pseudouridine synthase TruA

HVO_1851 - HAD superfamily hydrolase

//

>NGHB#1806 71

CMPLX not

HVO_1853 - UspA domain protein

HVO_1852 truA tRNA pseudouridine synthase TruA

//

>NGHB#1807 122

CMPLX not

HVO_1854 hisS histidine--tRNA ligase

HVO_1853 - UspA domain protein

//

>NGHB#1808 54

CMPLX not

HVO_1855 - DMT superfamily transport protein

HVO_1854 hisS histidine--tRNA ligase

//

>NGHB#1809 102

CMPLX ---

HVO_1856 - conserved hypothetical protein

HVO_1855 - DMT superfamily transport protein

//

>NGHB#1810 3

CMPLX ---

HVO_1857 - PDCD5 family DNA-binding protein

HVO_1856 - conserved hypothetical protein

//

>NGHB#1811 105

CMPLX not

HVO_1858 rps19e 30S ribosomal protein S19e

HVO_1857 - PDCD5 family DNA-binding protein

//

>NGHB#1812 114

CMPLX not

HVO_1859 - UPF0104 family protein

HVO_1858 rps19e 30S ribosomal protein S19e

//

>NGHB#1815 449

CMPLX ---

HVO_1863 - conserved hypothetical protein

HVO_1862 - M50 family metalloprotease

//

>NGHB#1816 306

CMPLX ---

HVO_1864 moaE molybdopterin synthase catalytic subunit

HVO_1863 - conserved hypothetical protein

//

>NGHB#1817 24

CMPLX ---

HVO_1865 - conserved hypothetical protein

HVO_1864 moaE molybdopterin synthase catalytic subunit

//

>NGHB#1819 1

CMPLX not

HVO_1866 pyrH uridylate kinase

HVO_1867 lysS lysine--tRNA ligase

//

>NGHB#1822 148

CMPLX ---

HVO_1869 - conserved hypothetical protein

HVO_1870 - M50 family metalloprotease

//

>NGHB#1823 106

CMPLX not

HVO_1870 - M50 family metalloprotease

HVO_1871 hemQ, pitA coproheme decarboxylase HemQ

//

>NGHB#1825 280

CMPLX ---

HVO_1873 - conserved hypothetical protein

HVO_1872 - conserved hypothetical protein

//

>NGHB#1826 -1

CMPLX ---

HVO_1874 - probable oxidoreductase (aldo-keto reductase family protein)

HVO_1873 - conserved hypothetical protein

//

>NGHB#1828 111

CMPLX not

HVO_1875 - O-acetyltransferase (homolog to galactoside O-acetyltransferase)

HVO_1876 - small CPxCG-related zinc finger protein

//

>NGHB#1829 50

CMPLX ---

HVO_1876 - small CPxCG-related zinc finger protein

HVO_1876A - conserved hypothetical protein

//

>NGHB#1830 702

CMPLX ---

HVO_1876A - conserved hypothetical protein

HVO_1878 nadE NAD synthase, ammonia-dependent

//

>NGHB#1834 241

CMPLX not

HVO_1883 - DUF457 family protein

HVO_1882 - pectin esterase

//

>NGHB#1836 40

CMPLX ---

HVO_1884 - conserved hypothetical protein

HVO_1885 trkA3 TrkA domain protein

//

>NGHB#1838 -4

CMPLX yes

HVO_1887 tupB ABC-type transport system permease protein (probable substrate tungstate)

HVO_1886 tupC ABC-type transport system ATP-binding protein (probable substrate tungstate)

//

>NGHB#1839 24

CMPLX yes

HVO_1888 tupA ABC-type transport system periplasmic substrate-binding protein (probable substrate tungstate)

HVO_1887 tupB ABC-type transport system permease protein (probable substrate tungstate)

//

>NGHB#1841 -8

CMPLX ---

HVO_1888_A - conserved hypothetical protein

HVO_1889 - conserved hypothetical protein

//

>NGHB#1843 -4

CMPLX ---

HVO_1891 - conserved hypothetical protein

HVO_1890 - conserved hypothetical protein

//

>NGHB#1844 60

CMPLX ---

HVO_1892 - UCP008459 family protein

HVO_1891 - conserved hypothetical protein

//

>NGHB#1845 160

CMPLX not

HVO_1893 ham1 XTP/dITP diphosphatase

HVO_1892 - UCP008459 family protein

//

>NGHB#1846 54

CMPLX ---

HVO_1894 - conserved hypothetical protein

HVO_1893 ham1 XTP/dITP diphosphatase

//

>NGHB#1847 64

CMPLX ---

HVO_1895 kae1 KEOPS complex subunit Kae1/Bud32

HVO_1894 - conserved hypothetical protein

//

>NGHB#1848 33

CMPLX not

HVO_1896 rps24e 30S ribosomal protein S24e

HVO_1895 kae1 KEOPS complex subunit Kae1/Bud32

//

>NGHB#1849 68

CMPLX not

HVO_1897 - UPF0218 family protein

HVO_1896 rps24e 30S ribosomal protein S24e

//

>NGHB#1850 13

CMPLX not

HVO_1898 spt4, rpoE2 transcription elongation factor Spt4

HVO_1897 - UPF0218 family protein

//

>NGHB#1851 -1

CMPLX not

HVO_1899 rpoE1 DNA-directed RNA polymerase subunit E

HVO_1898 spt4, rpoE2 transcription elongation factor Spt4

//

>NGHB#1852 7

CMPLX not

HVO_1900 - DUF188 family protein

HVO_1899 rpoE1 DNA-directed RNA polymerase subunit E

//

>NGHB#1853 1

CMPLX not

HVO_1901 tif2c translation initiation factor aIF-2 gamma subunit

HVO_1900 - DUF188 family protein

//

>NGHB#1854 238

CMPLX ---

HVO_1902 - conserved hypothetical protein

HVO_1901 tif2c translation initiation factor aIF-2 gamma subunit

//

>NGHB#1857 -4

CMPLX ---

HVO_1905 - beta-lactamase domain protein

HVO_1904 - conserved hypothetical protein

//

>NGHB#1861 58

CMPLX not

HVO_1908 nasA assimilatory nitrate reductase

HVO_1909 nasC, mobA3 molybdenum cofactor guanylyltransferase (nonfunctional)

//

>NGHB#1862 262

CMPLX not

HVO_1909 nasC, mobA3 molybdenum cofactor guanylyltransferase (nonfunctional)

HVO_1911 nasD, nirA2 nitrite reductase (ferredoxin) (nonfunctional)

//

>NGHB#1863 2

CMPLX ---

HVO_1911 nasD, nirA2 nitrite reductase (ferredoxin) (nonfunctional)

HVO_1913 - hypothetical protein

//

>NGHB#1864 127

CMPLX ---

HVO_1913 - hypothetical protein

HVO_1914 acaB3 acetyl-CoA C-acyltransferase

//

>NGHB#1865 130

CMPLX ---

HVO_1914 acaB3 acetyl-CoA C-acyltransferase

HVO_1915 - conserved hypothetical protein

//

>NGHB#1867 129

CMPLX not

HVO_1917 acs6 acyl-CoA synthetase

HVO_1916 kef1 Kef-type transport system

//

>NGHB#1868 97

CMPLX not

HVO_1918 - beta-lactamase domain protein

HVO_1917 acs6 acyl-CoA synthetase

//

>NGHB#1870 31

CMPLX ---

HVO_1919 - conserved hypothetical protein

HVO_1920 - DASS family transport protein

//

>NGHB#1871 177

CMPLX not

HVO_1920 - DASS family transport protein

HVO_1921 serS serine--tRNA ligase

//

>NGHB#1877 90

CMPLX not

HVO_1927 - DUF389 family protein

HVO_1926 - SIMPL domain protein

//

>NGHB#1878 42

CMPLX not

HVO_1928 - probable COG0212-type thiamine metabolism protein

HVO_1927 - DUF389 family protein

//

>NGHB#1879 91

CMPLX not

HVO_1929 - HTH-10 family transcription regulator

HVO_1928 - probable COG0212-type thiamine metabolism protein

//

>NGHB#1881 138

CMPLX not

HVO_1930_A - HTH domain protein

HVO_1931 engB probable GTP-binding protein EngB

//

>NGHB#1883 20

CMPLX not

HVO_1933 - DUF3179 family protein (nonfunctional)

HVO_1932 ddh, serA3 D-2-hydroxyacid dehydrogenase (NADP)

//

>NGHB#1884 282

CMPLX not

HVO_1934 - NUDIX family hydrolase / eIF-2B domain protein

HVO_1933 - DUF3179 family protein (nonfunctional)

//

>NGHB#1886 117

CMPLX not

HVO_1935 - MJ0936 family phosphodiesterase

HVO_1936 cofE coenzyme F420:L-glutamate ligase

//

>NGHB#1887 52

CMPLX not

HVO_1936 cofE coenzyme F420:L-glutamate ligase

HVO_1937 mer probable 5,10-methylenetetrahydrofolate reductase

//

>NGHB#1891 -4

CMPLX yes

HVO_1940 mutS1a DNA mismatch repair protein MutS

HVO_1939 mutLa DNA mismatch repair protein MutL

//

>NGHB#1893 5

CMPLX ---

HVO_1941 - AAA-type ATPase (MoxR subfamily)

HVO_1942 - conserved hypothetical protein

//

>NGHB#1894 -4

CMPLX ---

HVO_1942 - conserved hypothetical protein

HVO_1943 - endoisopeptidase/DUF4129 domain protein

//

>NGHB#1895 61

CMPLX not

HVO_1943 - endoisopeptidase/DUF4129 domain protein

HVO_1944 - probable transmembrane glycoprotein / HTH domain protein

//

>NGHB#1896 147

CMPLX ---

HVO_1944 - probable transmembrane glycoprotein / HTH domain protein

HVO_1945 - conserved hypothetical protein

//

>NGHB#1897 66

CMPLX ---

HVO_1945 - conserved hypothetical protein

HVO_1946 tif1a translation initiation factor aIF-1 (SUI1 protein, bacterial-type IF3)

//

>NGHB#1899 0

CMPLX not

HVO_1948 - NUDIX family hydrolase

HVO_1947 - rhodanese domain protein

//

>NGHB#1900 -4

CMPLX ---

HVO_1949 - conserved hypothetical protein

HVO_1948 - NUDIX family hydrolase

//

>NGHB#1901 24

CMPLX ---

HVO_1950 - conserved hypothetical protein

HVO_1949 - conserved hypothetical protein

//

>NGHB#1902 266

CMPLX ---

HVO_1951 - conserved hypothetical protein

HVO_1950 - conserved hypothetical protein

//

>NGHB#1903 159

CMPLX ---

HVO_1953 - receiver/sensor/bat box HTH-10 family transcription regulator (nonfunctional)

HVO_1951 - conserved hypothetical protein

//

>NGHB#1904 55

CMPLX not

HVO_1954 - GNAT family acetyltransferase

HVO_1953 - receiver/sensor/bat box HTH-10 family transcription regulator (nonfunctional)

//

>NGHB#1905 153

CMPLX not

HVO_1955 citB1 aconitate hydratase

HVO_1954 - GNAT family acetyltransferase

//

>NGHB#1907 402

CMPLX not

HVO_1956 dut dUTP diphosphatase

HVO_1957 panB, pan2 proteasome-activating nucleotidase

//

>NGHB#1908 111

CMPLX not

HVO_1957 panB, pan2 proteasome-activating nucleotidase

HVO_1958 pdaD pyruvoyl-dependent arginine decarboxylase

//

>NGHB#1909 51

CMPLX not

HVO_1958 pdaD pyruvoyl-dependent arginine decarboxylase

HVO_1959 - histidine kinase

//

>NGHB#1910 131

CMPLX ---

HVO_1959 - histidine kinase

HVO_1960 - conserved hypothetical protein

//

>NGHB#1911 173

CMPLX ---

HVO_1960 - conserved hypothetical protein

HVO_1961 - conserved hypothetical protein

//

>NGHB#1913 117

CMPLX ---

HVO_1963 tif5B translation initiation factor aIF-5B (bacterial-type IF2)

HVO_1962 - conserved hypothetical protein

//

>NGHB#1915 18

CMPLX not

HVO_1964 - PRC domain protein

HVO_1965 nob1 rRNA maturation endonuclease Nob1

//

>NGHB#1917 186

CMPLX not

HVO_1967 pgi glucose-6-phosphate isomerase

HVO_1966 - Abi/CAAX domain protein

//

>NGHB#1920 -4

CMPLX ---

HVO_1970 - PHP domain protein

HVO_1969 - conserved hypothetical protein

//

>NGHB#1921 -4

CMPLX not

HVO_1971 pgsA4 CDP-alcohol 1-archaetidyltransferase

HVO_1970 - PHP domain protein

//

>NGHB#1922 127

CMPLX not

HVO_1972 - GNAT family acetyltransferase

HVO_1971 pgsA4 CDP-alcohol 1-archaetidyltransferase

//

>NGHB#1926 -4

CMPLX yes

HVO_1975 secF protein-export membrane protein SecF

HVO_1976 secD protein-export membrane protein SecD

//

>NGHB#1927 281

CMPLX ---

HVO_1976 secD protein-export membrane protein SecD

HVO_1977 - conserved hypothetical protein

//

>NGHB#1929 88

CMPLX not

HVO_1979 pus10 tRNA pseudouridine synthase Pus10

HVO_1978 rnhB ribonuclease H, type 2

//

>NGHB#1932 100

CMPLX ---

HVO_1982 - DUF4112 family protein (nonfunctional)

HVO_1981 - conserved hypothetical protein

//

>NGHB#1933 215

CMPLX not

HVO_1983 aceB1 bifunctional malyl-CoA synthase / malyl-CoA thioesterase

HVO_1982 - DUF4112 family protein (nonfunctional)

//

>NGHB#1934 -8

CMPLX not

HVO_1984 aceA isocitrate lyase

HVO_1983 aceB1 bifunctional malyl-CoA synthase / malyl-CoA thioesterase

//

>NGHB#1935 -4

CMPLX ---

HVO_1984A - conserved hypothetical protein

HVO_1984 aceA isocitrate lyase

//

>NGHB#1937 -4

CMPLX yes

HVO_1985 - ABC-type transport system ATP-binding protein

HVO_1986 - ABC-type transport system permease protein

//

>NGHB#1938 145

CMPLX not

HVO_1986 - ABC-type transport system permease protein

HVO_1987 sppA2 signal peptide peptidase SppA

//

>NGHB#1939 -14

CMPLX not

HVO_1987 sppA2 signal peptide peptidase SppA

HVO_1988 - GATase domain protein

//

>NGHB#1940 214

CMPLX not

HVO_1988 - GATase domain protein

HVO_1989 trmY tRNA (pseudouridine(54)-N(1))-methyltransferase

//

>NGHB#1941 703

CMPLX not

HVO_1989 trmY tRNA (pseudouridine(54)-N(1))-methyltransferase

HVO_1991 - ABC-type transport system periplasmic substrate-binding protein

//

>NGHB#1946 45

CMPLX ---

HVO_1995 - conserved hypothetical protein

HVO_1996 - NUDIX family hydrolase

//

>NGHB#1947 91

CMPLX not

HVO_1996 - NUDIX family hydrolase

HVO_1997 - Abi/CAAX domain protein

//

>NGHB#1948 48

CMPLX not

HVO_1997 - Abi/CAAX domain protein

HVO_1998 - DASS family transport protein

//

>NGHB#1950 -4

CMPLX ---

HVO_2000 - conserved hypothetical protein

HVO_1999 htr7 transducer protein Htr7

//

>NGHB#1952 72

CMPLX not

HVO_2001 tgtA1 tRNA-guanine(15) transglycosylase

HVO_2002 - ArsR family transcription regulator

//

>NGHB#1953 91

CMPLX ---

HVO_2002 - ArsR family transcription regulator

HVO_2003 - conserved hypothetical protein

//

>NGHB#1955 42

CMPLX ---

HVO_2005 - hypothetical protein

HVO_2004 cysE serine O-acetyltransferase

//

>NGHB#1957 268

CMPLX ---

HVO_2006 - probable secreted glycoprotein

HVO_2006A - conserved hypothetical protein (nonfunctional)

//

>NGHB#1960 237

CMPLX ---

HVO_2008 arcS, tgtA2 archaeosine synthase

HVO_2009 - conserved hypothetical protein

//

>NGHB#1961 102

CMPLX ---

HVO_2009 - conserved hypothetical protein

HVO_2010 - homolog to phage PhiH1 repressor protein

//

>NGHB#1962 184

CMPLX ---

HVO_2010 - homolog to phage PhiH1 repressor protein

HVO_2011 - conserved hypothetical protein

//

>NGHB#1964 127

CMPLX not

HVO_2013 cetZ5, ftsZ7 FtsZ family protein CetZ, type III

HVO_2012 - receiver box response regulator

//

>NGHB#1967 138

CMPLX ---

HVO_2016 - conserved hypothetical protein

HVO_2015 - conserved hypothetical protein

//

>NGHB#1968 107

CMPLX ---

HVO_2017 - conserved hypothetical protein

HVO_2016 - conserved hypothetical protein

//

>NGHB#1969 99

CMPLX ---

HVO_2018 - conserved hypothetical protein

HVO_2017 - conserved hypothetical protein

//

>NGHB#1970 270

CMPLX ---

HVO_2019 - PRC domain protein

HVO_2018 - conserved hypothetical protein

//

>NGHB#1971 262

CMPLX ---

HVO_2021 - conserved hypothetical protein

HVO_2020 - DUF502 family protein

//

>NGHB#1972 270

CMPLX ---

HVO_2022 - conserved hypothetical protein

HVO_2021 - conserved hypothetical protein

//

>NGHB#1973 64

CMPLX ---

HVO_2023 - hypothetical protein

HVO_2022 - conserved hypothetical protein

//

>NGHB#1974 659

CMPLX ---

HVO_2024 - conserved hypothetical protein

HVO_2023 - hypothetical protein

//

>NGHB#1976 102

CMPLX ---

HVO_2025 - receiver/sensor box protein

HVO_2026 - hypothetical protein

//

>NGHB#1981 57

CMPLX yes

HVO_2031 tsgA12 ABC-type transport system periplasmic substrate-binding protein (probable substrate sugar)

HVO_2032 tsgD12 ABC-type transport system ATP-binding protein (probable substrate sugar)

//

>NGHB#1982 -4

CMPLX yes

HVO_2032 tsgD12 ABC-type transport system ATP-binding protein (probable substrate sugar)

HVO_2033 tsgB12 ABC-type transport system permease protein (probable substrate sugar)

//

>NGHB#1983 -4

CMPLX yes

HVO_2033 tsgB12 ABC-type transport system permease protein (probable substrate sugar)

HVO_2034 tsgC12 ABC-type transport system permease protein (probable substrate sugar)

//

>NGHB#1984 115

CMPLX not

HVO_2034 tsgC12 ABC-type transport system permease protein (probable substrate sugar)

HVO_2035 - TrmB family transcription regulator

//

>NGHB#1985 67

CMPLX ---

HVO_2035 - TrmB family transcription regulator

HVO_2036 - conserved hypothetical protein

//

>NGHB#1987 147

CMPLX ---

HVO_2037B - conserved hypothetical protein

HVO_2037 - DUF2078 family protein

//

>NGHB#1988 110

CMPLX ---

HVO_2037_A - conserved hypothetical protein

HVO_2037B - conserved hypothetical protein

//

>NGHB#1989 111

CMPLX ---

HVO_2038 - ABC-type transport system periplasmic substrate-binding protein

HVO_2037_A - conserved hypothetical protein

//

>NGHB#1990 308

CMPLX ---

HVO_2039 - conserved hypothetical protein

HVO_2038 - ABC-type transport system periplasmic substrate-binding protein

//

>NGHB#1992 313

CMPLX ---

HVO_2040 - GalE family epimerase/dehydratase

HVO_2041 - conserved hypothetical protein

//

>NGHB#1993 1346

CMPLX ---

HVO_2041 - conserved hypothetical protein

HVO_2042 orc4 Orc1-type DNA replication protein

//

>NGHB#1995 59

CMPLX not

HVO_2044 I-endH intein-related probable LAGLIDADG endonuclease I-EndH

HVO_2043 - probable glycosyltransferase, type 1 (homolog to EPS biosynthetic glycosyltransferase)

//

>NGHB#1999 1

CMPLX not

HVO_2047 - AlkP-core domain protein

HVO_2048 agl9 low-salt glycan biosynthesis hexosyltransferase Agl9

//

>NGHB#2000 43

CMPLX not

HVO_2048 agl9 low-salt glycan biosynthesis hexosyltransferase Agl9

HVO_2049 agl10 low-salt glycan biosynthesis hexosyltransferase Agl10

//

>NGHB#2003 77

CMPLX ---

HVO_2051 - ISH5-type transposase ISHvo11

HVO_2052 - conserved hypothetical protein

//

>NGHB#2004 124

CMPLX ---

HVO_2052 - conserved hypothetical protein

HVO_2053 agl5 low-salt glycan biosynthesis hexosyltransferase Agl5

//

>NGHB#2006 50

CMPLX not

HVO_2055 agl15 probable low-salt glycan biosynthesis flippase Agl15

HVO_2054 - ISH3-type transposase ISH51

//

>NGHB#2008 62

CMPLX not

HVO_2056 agl13 dTDP-4-dehydrorhamnose 3,5-epimerase

HVO_2057 agl11, graD2 glucose-1-phosphate thymidylyltransferase Agl11

//

>NGHB#2009 2

CMPLX not

HVO_2057 agl11, graD2 glucose-1-phosphate thymidylyltransferase Agl11

HVO_2057A - small CPxCG-related zinc finger protein

//

>NGHB#2012 0

CMPLX not

HVO_2059 agl12, rfbB dTDP-glucose 4,6-dehydratase

HVO_2060 agl8 low-salt glycan biosynthesis protein Agl8

//

>NGHB#2014 486

CMPLX not

HVO_2062 pilA2 pilin PilA

HVO_2061 agl6 low-salt glycan biosynthesis hexosyltransferase Agl6

//

>NGHB#2015 685

CMPLX ---

HVO_2063 - conserved hypothetical protein

HVO_2062 pilA2 pilin PilA

//

>NGHB#2016 282

CMPLX ---

HVO_2064 - conserved hypothetical protein

HVO_2063 - conserved hypothetical protein

//

>NGHB#2018 160

CMPLX ---

HVO_2065 - conserved hypothetical protein

HVO_2066 - conserved hypothetical protein

//

>NGHB#2020 178

CMPLX not

HVO_2068 cetZ6, ftsZ8 FtsZ family protein CetZ, type III

HVO_2067 - TetR family transcription regulator

//

>NGHB#2021 120

CMPLX not

HVO_2069 - RND superfamily permease

HVO_2068 cetZ6, ftsZ8 FtsZ family protein CetZ, type III

//

>NGHB#2022 3

CMPLX ---

HVO_2070 - conserved hypothetical protein

HVO_2069 - RND superfamily permease

//

>NGHB#2024 450

CMPLX not

HVO_2071 - probable secreted glycoprotein

HVO_2072 csg S-layer glycoprotein

//

>NGHB#2027 143

CMPLX not

HVO_2074 - probable secreted glycoprotein

HVO_2076 - probable secreted glycoprotein (nonfunctional)

//

>NGHB#2029 28

CMPLX ---

HVO_2080 - conserved hypothetical protein

HVO_2079 - RND superfamily permease

//

>NGHB#2031 257

CMPLX ---

HVO_2081 - pectin lyase domain protein

HVO_2082 - conserved hypothetical protein

//

>NGHB#2032 1

CMPLX ---

HVO_2082 - conserved hypothetical protein

HVO_2083 - ABC-type transport system ATP-binding protein (probable substrate macrolides)

//

>NGHB#2033 -4

CMPLX yes

HVO_2083 - ABC-type transport system ATP-binding protein (probable substrate macrolides)

HVO_2084 - ABC-type transport system permease protein (probable substrate macrolides)

//

>NGHB#2037 410

CMPLX not

HVO_2088 - glycoside hydrolase domain protein

HVO_2087 - glycoside hydrolase domain protein

//

>NGHB#2040 246

CMPLX not

HVO_2092 - IclR family transcription regulator

HVO_2091 - pyridoxal phosphate-dependent aminotransferase

//

>NGHB#2043 125

CMPLX not

HVO_2095 qor4 NADPH:quinone reductase

HVO_2094 - ABC-type transport system periplasmic substrate-binding protein

//

>NGHB#2045 83

CMPLX not

HVO_2096 - homolog to NAD-dependent epimerase/dehydratase

HVO_2097 - major facilitator superfamily transport protein

//

>NGHB#2046 109

CMPLX ---

HVO_2097 - major facilitator superfamily transport protein

HVO_2098 - conserved hypothetical protein

//

>NGHB#2047 307

CMPLX ---

HVO_2098 - conserved hypothetical protein

HVO_2099 - dioxygenase family protein

//

>NGHB#2049 16

CMPLX ---

HVO_2101 ptsH3 phosphocarrier protein HPr

HVO_2100 - hypothetical protein

//

>NGHB#2050 232

CMPLX not

HVO_2102 - phosphotransferase system component IIA

HVO_2101 ptsH3 phosphocarrier protein HPr

//

>NGHB#2051 115

CMPLX yes

HVO_2103 - phosphotransferase system component IIC

HVO_2102 - phosphotransferase system component IIA

//

>NGHB#2052 123

CMPLX yes

HVO_2104 - phosphotransferase system component IIB

HVO_2103 - phosphotransferase system component IIC

//

>NGHB#2053 106

CMPLX not

HVO_2105 tpiA2 triosephosphate isomerase

HVO_2104 - phosphotransferase system component IIB

//

>NGHB#2054 28

CMPLX not

HVO_2106 - DeoC family aldolase

HVO_2105 tpiA2 triosephosphate isomerase

//

>NGHB#2055 2

CMPLX not

HVO_2107 - UPF0047 family protein

HVO_2106 - DeoC family aldolase

//

>NGHB#2057 347

CMPLX not

HVO_2108 - IclR family transcription regulator

HVO_2109 - homolog to xylulose kinase

//

>NGHB#2058 720

CMPLX not

HVO_2109 - homolog to xylulose kinase

HVO_2110 - IclR family transcription regulator

//

>NGHB#2060 -4

CMPLX not

HVO_2112 - probable D-threonate kinase

HVO_2111 pdxA probable D-threonate 4-phosphate dehydrogenase

//

>NGHB#2062 133

CMPLX not

HVO_2113 tsgA2 ABC-type transport system periplasmic substrate-binding protein (probable substrate sugar)

HVO_2114 - homolog to 4-hydroxy-tetrahydrodipicolinate synthase

//

>NGHB#2065 -4

CMPLX yes

HVO_2116 tsgB2 ABC-type transport system permease protein (probable substrate sugar)

HVO_2117 tsgC2 ABC-type transport system permease protein (probable substrate sugar)

//

>NGHB#2066 120

CMPLX yes

HVO_2117 tsgC2 ABC-type transport system permease protein (probable substrate sugar)

HVO_2118 tsgD2 ABC-type transport system ATP-binding protein (probable substrate sugar)

//

>NGHB#2067 11

CMPLX ---

HVO_2118 tsgD2 ABC-type transport system ATP-binding protein (probable substrate sugar)

HVO_2119 - conserved hypothetical protein

//

>NGHB#2068 63

CMPLX ---

HVO_2119 - conserved hypothetical protein

HVO_2120 - conserved hypothetical protein

//

>NGHB#2070 -4

CMPLX ---

HVO_2122 dppF4 ABC-type transport system ATP-binding protein (probable substrate dipeptide/oligopeptide)

HVO_2121 - conserved hypothetical protein

//

>NGHB#2071 -4

CMPLX yes

HVO_2123 dppD4 ABC-type transport system ATP-binding protein (probable substrate dipeptide/oligopeptide)

HVO_2122 dppF4 ABC-type transport system ATP-binding protein (probable substrate dipeptide/oligopeptide)

//

>NGHB#2072 -4

CMPLX yes

HVO_2124 dppC4 ABC-type transport system permease protein (probable substrate dipeptide/oligopeptide)

HVO_2123 dppD4 ABC-type transport system ATP-binding protein (probable substrate dipeptide/oligopeptide)

//

>NGHB#2073 1

CMPLX yes

HVO_2125 dppB4 ABC-type transport system permease protein (probable substrate dipeptide/oligopeptide)

HVO_2124 dppC4 ABC-type transport system permease protein (probable substrate dipeptide/oligopeptide)

//

>NGHB#2074 75

CMPLX yes

HVO_2126 dppA4 ABC-type transport system periplasmic substrate-binding protein (probable substrate dipeptide/oligopeptide)

HVO_2125 dppB4 ABC-type transport system permease protein (probable substrate dipeptide/oligopeptide)

//

>NGHB#2076 129

CMPLX not

HVO_2127 - M20 family amidohydrolase (homolog to indole-3-acetyl-aspartate hydrolase)

HVO_2128 amaB1 amidase (hydantoinase/carbamoylase family)

//

>NGHB#2077 -4

CMPLX ---

HVO_2128 amaB1 amidase (hydantoinase/carbamoylase family)

HVO_2129 - hypothetical protein

//

>NGHB#2078 23

CMPLX ---

HVO_2129 - hypothetical protein

HVO_2130 - IclR family transcription regulator

//

>NGHB#2080 312

CMPLX ---

HVO_2132 - conserved hypothetical protein

HVO_2131 fbp2 fructose-1,6-bisphosphatase

//

>NGHB#2081 54

CMPLX ---

HVO_2133 orc16 Orc1-type DNA replication protein

HVO_2132 - conserved hypothetical protein

//

>NGHB#2083 132

CMPLX ---

HVO_2134 - DUF2249 family protein

HVO_2135 - conserved hypothetical protein

//

>NGHB#2087 241

CMPLX ---

HVO_2139 - conserved hypothetical protein

HVO_2138 - conserved hypothetical protein

//

>NGHB#2090 169

CMPLX not

HVO_2142 - small CPxCG-related zinc finger protein

HVO_2141 nirK nitrite reductase, copper-containing

//

>NGHB#2091 140

CMPLX not

HVO_2143 - DUF2249 family protein

HVO_2142 - small CPxCG-related zinc finger protein

//

>NGHB#2093 85

CMPLX not

HVO_2144 ahbD, pqqE2 coproheme decarboxylase AhbD

HVO_2145 hcpF halocyanin

//

>NGHB#2094 96

CMPLX ---

HVO_2145 hcpF halocyanin

HVO_2146 - conserved hypothetical protein

//

>NGHB#2103 113

CMPLX not

HVO_2153 - probable copper-containing oxidoreductase

HVO_2154 thiC phosphomethylpyrimidine synthase

//

>NGHB#2104 117

CMPLX not

HVO_2154 thiC phosphomethylpyrimidine synthase

HVO_2155 - homolog to sodium/calcium antiporter

//

>NGHB#2106 88

CMPLX ---

HVO_2157 - conserved hypothetical protein

HVO_2156 - UspA domain protein

//

>NGHB#2107 119

CMPLX ---

HVO_2158 maeB1, mdh1 malic enzyme (NADP)

HVO_2157 - conserved hypothetical protein

//

>NGHB#2108 176

CMPLX ---

HVO_2159 - conserved hypothetical protein

HVO_2158 maeB1, mdh1 malic enzyme (NADP)

//

>NGHB#2109 450

CMPLX ---

HVO_2159A - conserved hypothetical protein

HVO_2159 - conserved hypothetical protein

//

>NGHB#2111 120

CMPLX not

HVO_2160 - probable secreted glycoprotein

HVO_2161 - probable secreted glycoprotein

//

>NGHB#2114 -8

CMPLX yes

HVO_2163 - ABC-type transport system ATP-binding protein

HVO_2164 - ABC-type transport system permease protein

//

>NGHB#2115 -8

CMPLX yes

HVO_2164 - ABC-type transport system permease protein

HVO_2165 - ABC-type transport system permease protein

//

>NGHB#2116 74

CMPLX ---

HVO_2165 - ABC-type transport system permease protein

HVO_2166 - conserved hypothetical protein

//

>NGHB#2117 0

CMPLX ---

HVO_2166 - conserved hypothetical protein

HVO_2167 - conserved hypothetical protein

//

>NGHB#2118 98

CMPLX ---

HVO_2167 - conserved hypothetical protein

HVO_2168 moxR3 AAA-type ATPase (MoxR subfamily)

//

>NGHB#2120 -4

CMPLX ---

HVO_2170 - conserved hypothetical protein

HVO_2169 - conserved hypothetical protein

//

>NGHB#2121 18

CMPLX ---

HVO_2171 - DUF58 family protein

HVO_2170 - conserved hypothetical protein

//

>NGHB#2123 61

CMPLX ---

HVO_2172 - conserved hypothetical protein

HVO_2173 - DUF1616 family protein

//

>NGHB#2124 191

CMPLX ---

HVO_2173 - DUF1616 family protein

HVO_2174 - conserved hypothetical protein

//

>NGHB#2125 35

CMPLX ---

HVO_2174 - conserved hypothetical protein

HVO_2175 sph3 Smc-like protein Sph3

//

>NGHB#2126 1

CMPLX not

HVO_2177 samp3 ubiquitin-like modifier protein SAMP3

HVO_2178 - ubiquitin-like protein

//

>NGHB#2127 -4

CMPLX ---

HVO_2178 - ubiquitin-like protein

HVO_2179 - conserved hypothetical protein

//

>NGHB#2128 36

CMPLX ---

HVO_2179 - conserved hypothetical protein

HVO_2180 - ABC-type transport system permease protein

//

>NGHB#2129 0

CMPLX yes

HVO_2180 - ABC-type transport system permease protein

HVO_2181 - ABC-type transport system accessory transmembrane protein

//

>NGHB#2130 76

CMPLX ---

HVO_2181 - ABC-type transport system accessory transmembrane protein

HVO_2182 - conserved hypothetical protein

//

>NGHB#2132 126

CMPLX ---

HVO_2184 - conserved hypothetical protein (nonfunctional)

HVO_2183 aor4 aldehyde ferredoxin oxidoreductase

//

>NGHB#2134 78

CMPLX not

HVO_2186 - DICT domain protein

HVO_2187 - TIGR01210 family protein

//

>NGHB#2135 275

CMPLX ---

HVO_2187 - TIGR01210 family protein

HVO_2187A - conserved hypothetical protein

//

>NGHB#2137 1

CMPLX yes

HVO_2189 purS phosphoribosylformylglycinamidine synthase subunit PurS

HVO_2188 purQ phosphoribosylformylglycinamidine synthase subunit PurQ

//

>NGHB#2139 145

CMPLX not

HVO_2190 - gluconate permease

HVO_2191 purU formyltetrahydrofolate deformylase

//

>NGHB#2140 76

CMPLX ---

HVO_2191 purU formyltetrahydrofolate deformylase

HVO_2192 - conserved hypothetical protein

//

>NGHB#2143 9

CMPLX not

HVO_2194 sir2 Sir2-type NAD-dependent protein deacetylase

HVO_2195 - sensor box histidine kinase (nonfunctional)

//

>NGHB#2146 1

CMPLX not

HVO_2198 cofH 5-amino-6-(D-ribitylamino)uracil--L-tyrosine 4-hydroxyphenyl transferase

HVO_2199 - DUF457 family protein

//

>NGHB#2147 132

CMPLX not

HVO_2199 - DUF457 family protein

HVO_2200 - DUF457 family protein

//

>NGHB#2149 315

CMPLX not

HVO_2202 cofC 2-phospho-L-lactate guanylyltransferase

HVO_2201 cofG 7,8-didemethyl-8-hydroxy-5-deazariboflavin synthase

//

>NGHB#2150 6

CMPLX ---

HVO_2203 - conserved hypothetical protein

HVO_2202 cofC 2-phospho-L-lactate guanylyltransferase

//

>NGHB#2151 -32

CMPLX ---

HVO_2204 cetZ1, ftsZ4 FtsZ family protein CetZ, type III

HVO_2203 - conserved hypothetical protein

//

>NGHB#2152 211

CMPLX not

HVO_2205 nolA arNOG06768 family NADH-binding domain protein

HVO_2204 cetZ1, ftsZ4 FtsZ family protein CetZ, type III

//

>NGHB#2153 146

CMPLX ---

HVO_2206 - conserved hypothetical protein

HVO_2205 nolA arNOG06768 family NADH-binding domain protein

//

>NGHB#2159 2

CMPLX not

HVO_2211 trkA4 TrkA domain protein

HVO_2212 - Lrp/AsnC family transcription regulator

//

>NGHB#2161 330

CMPLX ---

HVO_2214 htr36 transducer protein Htr36

HVO_2213 - conserved hypothetical protein

//

>NGHB#2162 113

CMPLX ---

HVO_2215 - conserved hypothetical protein

HVO_2214 htr36 transducer protein Htr36

//

>NGHB#2163 9

CMPLX ---

HVO_2219 - hypothetical protein

HVO_2218 - conserved hypothetical protein

//

>NGHB#2165 114

CMPLX ---

HVO_2220 htr38 transducer protein Htr38

HVO_2221 - conserved hypothetical protein

//

>NGHB#2168 90

CMPLX not

HVO_2223 - BASS family transport protein

HVO_2224 - DUF2892 family protein

//

>NGHB#2169 86

CMPLX not

HVO_2224 - DUF2892 family protein

HVO_2225 - AstE domain protein

//

>NGHB#2173 136

CMPLX not

HVO_2229 - flavin-containing amine-oxidoreductase

HVO_2228 - PHP domain protein

//

>NGHB#2175 -4

CMPLX not

HVO_2230 - HAD superfamily hydrolase

HVO_2231 - probable oxidoreductase (aldo-keto reductase family protein)

//

>NGHB#2176 86

CMPLX not

HVO_2231 - probable oxidoreductase (aldo-keto reductase family protein)

HVO_2232 cirD KaiC-type circadian clock protein CirD

//

>NGHB#2177 10

CMPLX ---

HVO_2232 cirD KaiC-type circadian clock protein CirD

HVO_2233 - conserved hypothetical protein

//

>NGHB#2178 63

CMPLX ---

HVO_2233 - conserved hypothetical protein

HVO_2234 msrB peptide methionine sulfoxide reductase MsrB (R-form specific)

//

>NGHB#2180 -4

CMPLX ---

HVO_2236 - ArsR family transcription regulator

HVO_2235 - conserved hypothetical protein

//

>NGHB#2181 197

CMPLX ---

HVO_2237 - hypothetical protein

HVO_2236 - ArsR family transcription regulator

//

>NGHB#2184 120

CMPLX ---

HVO_2240 - conserved hypothetical protein

HVO_2239 - UspA domain protein

//

>NGHB#2189 89

CMPLX ---

HVO_2245 - conserved hypothetical protein

HVO_2244 - fumarylacetoacetase family protein

//

>NGHB#2190 -4

CMPLX ---

HVO_2246 - hypothetical protein

HVO_2245 - conserved hypothetical protein

//

>NGHB#2191 -4

CMPLX ---

HVO_2247 - mvp-type potassium channel superfamily protein

HVO_2246 - hypothetical protein

//

>NGHB#2192 82

CMPLX ---

HVO_2248 - conserved hypothetical protein

HVO_2247 - mvp-type potassium channel superfamily protein

//

>NGHB#2193 130

CMPLX ---

HVO_2249 uraA2 xanthine/uracil permease family transport protein

HVO_2248 - conserved hypothetical protein

//

>NGHB#2194 174

CMPLX not

HVO_2250 apt3, hpt purine phosphoribosyltransferase (adenine phosphoribosyltransferase, xanthine-guanine phosphoribosyltransferase)

HVO_2249 uraA2 xanthine/uracil permease family transport protein

//

>NGHB#2195 69

CMPLX not

HVO_2251 - LctP family transport protein

HVO_2250 apt3, hpt purine phosphoribosyltransferase (adenine phosphoribosyltransferase, xanthine-guanine phosphoribosyltransferase)

//

>NGHB#2198 68

CMPLX ---

HVO_2254 - conserved hypothetical protein

HVO_2253 - conserved hypothetical protein

//

>NGHB#2200 62

CMPLX ---

HVO_2255 - conserved hypothetical protein

HVO_2256 - DUF262 family protein

//

>NGHB#2201 88

CMPLX ---

HVO_2256 - DUF262 family protein

HVO_2257 - conserved hypothetical protein

//

>NGHB#2202 108

CMPLX ---

HVO_2257 - conserved hypothetical protein

HVO_2258 - conserved hypothetical protein

//

>NGHB#2203 1

CMPLX ---

HVO_2258 - conserved hypothetical protein

HVO_2259 - XerC/D-like integrase

//

>NGHB#2204 13

CMPLX ---

HVO_2259 - XerC/D-like integrase

HVO_2260 - hypothetical protein

//

>NGHB#2205 371

CMPLX ---

HVO_2260 - hypothetical protein

HVO_2261 - hypothetical protein

//

>NGHB#2206 3

CMPLX ---

HVO_2261 - hypothetical protein

HVO_2262 - hypothetical protein

//

>NGHB#2207 153

CMPLX ---

HVO_2262 - hypothetical protein

HVO_2263 - HTH domain protein

//

>NGHB#2210 -8

CMPLX ---

HVO_2265 - conserved hypothetical protein

HVO_2266 - conserved hypothetical protein

//

>NGHB#2211 -17

CMPLX ---

HVO_2266 - conserved hypothetical protein

HVO_2267 - conserved hypothetical protein

//

>NGHB#2214 -4

CMPLX yes

HVO_2269 rmeR type I site-specific deoxyribonuclease subunit RmeR

HVO_2270 rmeM type I restriction-modification system DNA-methyltransferase RmeM

//

>NGHB#2215 -1

CMPLX yes

HVO_2270 rmeM type I restriction-modification system DNA-methyltransferase RmeM

HVO_2271 rmeS type I site-specific deoxyribonuclease subunit RmeS

//

>NGHB#2216 229

CMPLX ---

HVO_2271 rmeS type I site-specific deoxyribonuclease subunit RmeS

HVO_2272 - conserved hypothetical protein

//

>NGHB#2217 -1

CMPLX ---

HVO_2272 - conserved hypothetical protein

HVO_2273 - XerC/D-like integrase

//

>NGHB#2218 11

CMPLX ---

HVO_2273 - XerC/D-like integrase

HVO_2274 - hypothetical protein

//

>NGHB#2221 111

CMPLX ---

HVO_2275 - N-6 adenine-specific DNA methylase domain protein

HVO_2276 - conserved hypothetical protein

//

>NGHB#2223 -4

CMPLX ---

HVO_2278 - conserved hypothetical protein

HVO_2277 - conserved hypothetical protein

//

>NGHB#2224 -4

CMPLX ---

HVO_2279 - conserved hypothetical protein

HVO_2278 - conserved hypothetical protein

//

>NGHB#2225 -4

CMPLX ---

HVO_2280 - hypothetical protein

HVO_2279 - conserved hypothetical protein

//

>NGHB#2226 -4

CMPLX ---

HVO_2281 - conserved hypothetical protein

HVO_2280 - hypothetical protein

//

>NGHB#2227 -4

CMPLX ---

HVO_2282 - conserved hypothetical protein

HVO_2281 - conserved hypothetical protein

//

>NGHB#2228 -4

CMPLX ---

HVO_2283 - conserved hypothetical protein

HVO_2282 - conserved hypothetical protein

//

>NGHB#2229 6

CMPLX ---

HVO_2284 - conserved hypothetical protein

HVO_2283 - conserved hypothetical protein

//

>NGHB#2230 557

CMPLX ---

HVO_2284A - hypothetical protein

HVO_2284 - conserved hypothetical protein

//

>NGHB#2231 74

CMPLX ---

HVO_2285 - hypothetical protein

HVO_2284A - hypothetical protein

//

>NGHB#2232 404

CMPLX ---

HVO_2286 - conserved hypothetical protein

HVO_2285 - hypothetical protein

//

>NGHB#2234 932

CMPLX ---

HVO_2287 - DUF932 family protein

HVO_2288 - hypothetical protein

//

>NGHB#2235 76

CMPLX ---

HVO_2288 - hypothetical protein

HVO_2289 - conserved hypothetical protein

//

>NGHB#2236 542

CMPLX ---

HVO_2289 - conserved hypothetical protein

HVO_2290 - integrase family protein

//

>NGHB#2237 39

CMPLX ---

HVO_2290 - integrase family protein

HVO_2291 - hypothetical protein

//

>NGHB#2238 58

CMPLX ---

HVO_2291 - hypothetical protein

HVO_2291B - conserved hypothetical protein

//

>NGHB#2239 33

CMPLX ---

HVO_2291B - conserved hypothetical protein

HVO_2291_A - conserved hypothetical protein

//

>NGHB#2243 93

CMPLX ---

HVO_2295 - homolog to cationic amino acid transport protein N-terminal region

HVO_2294 - conserved hypothetical protein

//

>NGHB#2244 101

CMPLX not

HVO_2296 - HTH-10 family transcription regulator (nonfunctional)

HVO_2295 - homolog to cationic amino acid transport protein N-terminal region

//

>NGHB#2245 129

CMPLX not

HVO_2297 dhs1 deoxyhypusine synthase

HVO_2296 - HTH-10 family transcription regulator (nonfunctional)

//

>NGHB#2246 81

CMPLX not

HVO_2298 - GTP cyclohydrolase 1 domain protein

HVO_2297 dhs1 deoxyhypusine synthase

//

>NGHB#2247 141

CMPLX not

HVO_2299 speB agmatinase

HVO_2298 - GTP cyclohydrolase 1 domain protein

//

>NGHB#2248 15

CMPLX not

HVO_2300 tef5A translation elongation factor aEF-5A

HVO_2299 speB agmatinase

//

>NGHB#2249 94

CMPLX ---

HVO_2301 - conserved hypothetical protein

HVO_2300 tef5A translation elongation factor aEF-5A

//

>NGHB#2250 57

CMPLX ---

HVO_2302 - UbiB family protein

HVO_2301 - conserved hypothetical protein

//

>NGHB#2251 8

CMPLX not

HVO_2303 hsp20E Hsp20-type molecular chaperone

HVO_2302 - UbiB family protein

//

>NGHB#2253 -4

CMPLX not

HVO_2304 moeA1 molybdopterin molybdenumtransferase

HVO_2305 moeA2 molybdopterin molybdenumtransferase

//

>NGHB#2254 100

CMPLX not

HVO_2305 moeA2 molybdopterin molybdenumtransferase

HVO_2306 - receiver box response regulator

//

>NGHB#2256 46

CMPLX ---

HVO_2308 - conserved hypothetical protein

HVO_2307 - HAD superfamily hydrolase

//

>NGHB#2257 -4

CMPLX ---

HVO_2309 cad probable pterin-4-alpha-carbinolamine dehydratase

HVO_2308 - conserved hypothetical protein

//

>NGHB#2258 45

CMPLX ---

HVO_2310 - conserved hypothetical protein

HVO_2309 cad probable pterin-4-alpha-carbinolamine dehydratase

//

>NGHB#2259 94

CMPLX ---

HVO_2311 hemA glutamyl-tRNA reductase

HVO_2310 - conserved hypothetical protein

//

>NGHB#2260 -4

CMPLX not

HVO_2312 sirC precorrin-2 oxidase / ferrochelatase

HVO_2311 hemA glutamyl-tRNA reductase

//

>NGHB#2261 1

CMPLX not

HVO_2313 ahbB, nirGH siroheme decarboxylase AhbB

HVO_2312 sirC precorrin-2 oxidase / ferrochelatase

//

>NGHB#2262 180

CMPLX ---

HVO_2314 - conserved hypothetical protein

HVO_2313 ahbB, nirGH siroheme decarboxylase AhbB

//

>NGHB#2265 71

CMPLX ---

HVO_2317 - conserved hypothetical protein

HVO_2316 - serpin family protein

//

>NGHB#2266 140

CMPLX ---

HVO_2318 uppS2 tritrans,polycis-undecaprenyl-diphosphate synthase (geranylgeranyl-diphosphate specific)

HVO_2317 - conserved hypothetical protein

//

>NGHB#2268 -4

CMPLX not

HVO_2319 - DUF92 family protein

HVO_2320 - GNAT family acetyltransferase

//

>NGHB#2270 223

CMPLX not

HVO_2322 - YfiH family protein

HVO_2321 dnaG DNA primase DnaG

//

>NGHB#2272 -1

CMPLX not

HVO_2323 - DUF3311 family protein

HVO_2324 - SSSF family transport protein

//

>NGHB#2273 69

CMPLX ---

HVO_2324 - SSSF family transport protein

HVO_2325 - conserved hypothetical protein

//

>NGHB#2274 75

CMPLX ---

HVO_2325 - conserved hypothetical protein

HVO_2326 - GNAT family acetyltransferase

//

>NGHB#2275 116

CMPLX not

HVO_2326 - GNAT family acetyltransferase

HVO_2327 - YcgG family protein

//

>NGHB#2276 28

CMPLX not

HVO_2327 - YcgG family protein

HVO_2328 entB1 isochorismatase family protein

//

>NGHB#2277 75

CMPLX ---

HVO_2328 entB1 isochorismatase family protein

HVO_2329 - conserved hypothetical protein

//

>NGHB#2278 119

CMPLX ---

HVO_2329 - conserved hypothetical protein

HVO_2330 - sulfate permease family protein

//

>NGHB#2279 107

CMPLX not

HVO_2330 - sulfate permease family protein

HVO_2331 - ISH3-type transposase ISH51

//

>NGHB#2280 254

CMPLX ---

HVO_2331 - ISH3-type transposase ISH51

HVO_2332 - conserved hypothetical protein

//

>NGHB#2281 132

CMPLX ---

HVO_2332 - conserved hypothetical protein

HVO_2333 - conserved hypothetical protein

//

>NGHB#2283 43

CMPLX ---

HVO_2335 - DUF1405 family protein

HVO_2334 - conserved hypothetical protein

//

>NGHB#2286 133

CMPLX not

HVO_2338 thrB homoserine kinase

HVO_2337 - UspA domain protein

//

>NGHB#2287 66

CMPLX not

HVO_2339 - sensor box histidine kinase

HVO_2338 thrB homoserine kinase

//

>NGHB#2289 147

CMPLX not

HVO_2340 - amine oxidase domain protein

HVO_2341 - TetR family transcription regulator

//

>NGHB#2290 -4

CMPLX not

HVO_2341 - TetR family transcription regulator

HVO_2342 - MATE efflux family protein

//

>NGHB#2293 291

CMPLX not

HVO_2344 - HTH domain protein

HVO_2345 - flavin-dependent pyridine nucleotide oxidoreductase

//

>NGHB#2294 199

CMPLX ---

HVO_2345 - flavin-dependent pyridine nucleotide oxidoreductase

HVO_2346 - conserved hypothetical protein

//

>NGHB#2295 126

CMPLX ---

HVO_2346 - conserved hypothetical protein

HVO_2347 - conserved hypothetical protein

//

>NGHB#2296 81

CMPLX ---

HVO_2347 - conserved hypothetical protein

HVO_2348 mptA, folE2 GTP cyclohydrolase MptA

//

>NGHB#2298 74

CMPLX not

HVO_2350 - YyaL family protein

HVO_2349 - TrmB family transcription regulator

//

>NGHB#2300 577

CMPLX ---

HVO_2351 fxsA FxsA domain protein

HVO_2353 - hypothetical protein

//

>NGHB#2301 181

CMPLX ---

HVO_2353 - hypothetical protein

HVO_2354 - conserved hypothetical protein

//

>NGHB#2303 69

CMPLX not

HVO_2356 - GNAT family acetyltransferase

HVO_2355 - homolog to sodium/calcium antiporter

//

>NGHB#2305 164

CMPLX ---

HVO_2357 - conserved hypothetical protein

HVO_2358 - conserved hypothetical protein

//

>NGHB#2306 157

CMPLX ---

HVO_2358 - conserved hypothetical protein

HVO_2359 - conserved hypothetical protein

//

>NGHB#2309 152

CMPLX not

HVO_2361 carB carbamoyl-phosphate synthase (glutamine-hydrolyzing) large subunit

HVO_2362 - homolog to NAD-dependent epimerase/dehydratase

//

>NGHB#2313 162

CMPLX not

HVO_2366 - UspA domain protein

HVO_2365 - nitroreductase family protein

//

>NGHB#2314 58

CMPLX not

HVO_2367 - arNOG05179 family protein (DUF87-related AAA-type ATPase)

HVO_2366 - UspA domain protein

//

>NGHB#2319 74

CMPLX ---

HVO_2372 - DUF2240 family protein

HVO_2371 - conserved hypothetical protein

//

>NGHB#2323 63

CMPLX yes

HVO_2375 pstS1 ABC-type transport system periplasmic substrate-binding protein (probable substrate phosphate)

HVO_2376 pstC1 ABC-type transport system permease protein (probable substrate phosphate)

//

>NGHB#2324 1

CMPLX yes

HVO_2376 pstC1 ABC-type transport system permease protein (probable substrate phosphate)

HVO_2377 pstA1 ABC-type transport system permease protein (probable substrate phosphate)

//

>NGHB#2325 9

CMPLX yes

HVO_2377 pstA1 ABC-type transport system permease protein (probable substrate phosphate)

HVO_2378 pstB1 ABC-type transport system ATP-binding protein (probable substrate phosphate)

//

>NGHB#2326 189

CMPLX not

HVO_2378 pstB1 ABC-type transport system ATP-binding protein (probable substrate phosphate)

HVO_2379 phoU3 PhoU domain protein

//

>NGHB#2333 -11

CMPLX yes

HVO_2385 pilB5 type IV pilus biogenesis complex ATPase subunit

HVO_2386 pilC5 type IV pilus biogenesis complex membrane subunit

//

>NGHB#2334 4

CMPLX not

HVO_2386 pilC5 type IV pilus biogenesis complex membrane subunit

HVO_2387 - probable secreted glycoprotein

//

>NGHB#2335 3

CMPLX not

HVO_2387 - probable secreted glycoprotein

HVO_2388 - probable secreted glycoprotein

//

>NGHB#2336 119

CMPLX not

HVO_2388 - probable secreted glycoprotein

HVO_2389 hadL, dehII haloacid dehalogenase, type II

//

>NGHB#2337 81

CMPLX ---

HVO_2389 hadL, dehII haloacid dehalogenase, type II

HVO_2390 - conserved hypothetical protein

//

>NGHB#2341 259

CMPLX ---

HVO_2395 pduO ATP:cob(I)alamin adenosyltransferase

HVO_2394 - conserved hypothetical protein

//

>NGHB#2342 -4

CMPLX not

HVO_2396 grx4 glutaredoxin

HVO_2395 pduO ATP:cob(I)alamin adenosyltransferase

//

>NGHB#2344 -4

CMPLX yes

HVO_2397 znuA1 ABC-type transport system periplasmic substrate-binding protein (probable substrate zinc)

HVO_2398 znuC1 ABC-type transport system ATP-binding protein (probable substrate zinc)

//

>NGHB#2345 -4

CMPLX yes

HVO_2398 znuC1 ABC-type transport system ATP-binding protein (probable substrate zinc)

HVO_2399 znuB1 ABC-type transport system permease protein (probable substrate zinc)

//

>NGHB#2346 175

CMPLX not

HVO_2399 znuB1 ABC-type transport system permease protein (probable substrate zinc)

HVO_2400 - small CPxCG-related zinc finger protein

//

>NGHB#2348 -1

CMPLX yes

HVO_2402 gcvP1 glycine cleavage system protein P alpha subunit

HVO_2401 gcvP2 glycine cleavage system protein P beta subunit

//

>NGHB#2349 1

CMPLX yes

HVO_2403 gcvH glycine cleavage system protein H

HVO_2402 gcvP1 glycine cleavage system protein P alpha subunit

//

>NGHB#2350 2

CMPLX yes

HVO_2404 gcvT glycine cleavage system protein T

HVO_2403 gcvH glycine cleavage system protein H

//

>NGHB#2351 230

CMPLX not

HVO_2405 nikO ABC-type transport system ATP-binding protein (probable substrate nickel)

HVO_2404 gcvT glycine cleavage system protein T

//

>NGHB#2352 -13

CMPLX yes

HVO_2406 nikQ ABC-type transport system permease protein (probable substrate nickel)

HVO_2405 nikO ABC-type transport system ATP-binding protein (probable substrate nickel)

//

>NGHB#2353 -4

CMPLX yes

HVO_2407 nikN ABC-type transport system protein NikN (probable substrate nickel)

HVO_2406 nikQ ABC-type transport system permease protein (probable substrate nickel)

//

>NGHB#2354 -4

CMPLX yes

HVO_2408 nikM ABC-type transport system permease protein (probable substrate nickel)

HVO_2407 nikN ABC-type transport system protein NikN (probable substrate nickel)

//

>NGHB#2357 -11

CMPLX not

HVO_2411 mrpD2 Mrp-type sodium/proton antiporter system subunit D2

HVO_2410 - UPF0753 family protein

//

>NGHB#2358 377

CMPLX not

HVO_2412 - Lrp/AsnC family transcription regulator

HVO_2411 mrpD2 Mrp-type sodium/proton antiporter system subunit D2

//

>NGHB#2362 106

CMPLX not

HVO_2415 - DUF88 family protein

HVO_2416 tatD 3'-5' ssDNA/RNA exonuclease TatD

//

>NGHB#2363 152

CMPLX not

HVO_2416 tatD 3'-5' ssDNA/RNA exonuclease TatD

HVO_2417 - DUF2150 family protein

//

>NGHB#2364 57

CMPLX not

HVO_2417 - DUF2150 family protein

HVO_2418 - probable secreted glycoprotein

//

>NGHB#2365 62

CMPLX not

HVO_2418 - probable secreted glycoprotein

HVO_2419 hmgB, mvaB hydroxymethylglutaryl-CoA synthase

//

>NGHB#2367 139

CMPLX ---

HVO_2421 - cro/C1 family transcription regulator

HVO_2420 - conserved hypothetical protein (nonfunctional)

//

>NGHB#2369 88

CMPLX not

HVO_2422 - YdjM family protein

HVO_2423 - GNAT family acetyltransferase

//

>NGHB#2371 184

CMPLX ---

HVO_2425 - GNAT family acetyltransferase

HVO_2424 - conserved hypothetical protein

//

>NGHB#2373 138

CMPLX ---

HVO_2426 - conserved hypothetical protein

HVO_2427 rfcB replication factor C large subunit

//

>NGHB#2374 88

CMPLX not

HVO_2427 rfcB replication factor C large subunit

HVO_2428 adh1 alcohol dehydrogenase

//

>NGHB#2376 70

CMPLX not

HVO_2430 - ABC-type transport system ATP-binding protein (probable substrate glutamine/glutamate/polar amino acids)

HVO_2429 - small CPxCG-related zinc finger protein

//

>NGHB#2377 -4

CMPLX yes

HVO_2431 - ABC-type transport system permease protein (probable substrate glutamine/glutamate/polar amino acids)

HVO_2430 - ABC-type transport system ATP-binding protein (probable substrate glutamine/glutamate/polar amino acids)

//

>NGHB#2378 43

CMPLX yes

HVO_2432 - ABC-type transport system periplasmic substrate-binding protein (probable substrate glutamine/glutamate/polar amino acids)

HVO_2431 - ABC-type transport system permease protein (probable substrate glutamine/glutamate/polar amino acids)

//

>NGHB#2379 288

CMPLX not

HVO_2433 ctaA heme A synthase

HVO_2432 - ABC-type transport system periplasmic substrate-binding protein (probable substrate glutamine/glutamate/polar amino acids)

//

>NGHB#2381 219

CMPLX not

HVO_2434 - IS1341-type transposase ISHvo17

HVO_2435 - peptidase M24 family protein

//

>NGHB#2382 131

CMPLX not

HVO_2435 - peptidase M24 family protein

HVO_2436 maeB2, mdh2 malate dehydrogenase (oxaloacetate-decarboxylating)

//

>NGHB#2384 177

CMPLX ---

HVO_2438 rnhA1 ribonuclease H, type 1

HVO_2437 - conserved hypothetical protein

//

>NGHB#2385 6

CMPLX yes

HVO_2441 dppB5 ABC-type transport system permease protein (probable substrate dipeptide/oligopeptide) (nonfunctional)

HVO_2442 dppC5 ABC-type transport system permease protein (probable substrate dipeptide/oligopeptide) (nonfunctional)

//

>NGHB#2386 1

CMPLX yes

HVO_2442 dppC5 ABC-type transport system permease protein (probable substrate dipeptide/oligopeptide) (nonfunctional)

HVO_2443 dppD5 ABC-type transport system ATP-binding protein (probable substrate dipeptide/oligopeptide)

//

>NGHB#2387 -8

CMPLX yes

HVO_2443 dppD5 ABC-type transport system ATP-binding protein (probable substrate dipeptide/oligopeptide)

HVO_2444 dppF5 ABC-type transport system ATP-binding protein (probable substrate dipeptide/oligopeptide) (nonfunctional)

//

>NGHB#2388 110

CMPLX not

HVO_2444 dppF5 ABC-type transport system ATP-binding protein (probable substrate dipeptide/oligopeptide) (nonfunctional)

HVO_2446 - DMT superfamily transport protein

//

>NGHB#2391 188

CMPLX ---

HVO_2447A - conserved hypothetical protein

HVO_2448 - probable oxidoreductase (aldo-keto reductase family protein)

//

>NGHB#2392 60

CMPLX not

HVO_2448 - probable oxidoreductase (aldo-keto reductase family protein)

HVO_2449 - DUF1684 family protein

//

>NGHB#2393 53

CMPLX ---

HVO_2449 - DUF1684 family protein

HVO_2449_A - conserved hypothetical protein

//

>NGHB#2395 0

CMPLX yes

HVO_2451 pilA4 pilin PilA

HVO_2450 pilA3 pilin PilA

//

>NGHB#2396 453

CMPLX not

HVO_2452 nrdJ ribonucleoside-diphosphate reductase, adenosylcobalamin-dependent

HVO_2451 pilA4 pilin PilA

//

>NGHB#2399 -4

CMPLX yes

HVO_2454 trpE anthranilate synthase component 1

HVO_2453 trpG anthranilate synthase component 2

//

>NGHB#2400 -4

CMPLX not

HVO_2455 trpF N-(5'-phosphoribosyl)anthranilate isomerase

HVO_2454 trpE anthranilate synthase component 1

//

>NGHB#2401 1

CMPLX not

HVO_2456 trpD1 anthranilate phosphoribosyltransferase

HVO_2455 trpF N-(5'-phosphoribosyl)anthranilate isomerase

//

>NGHB#2404 112

CMPLX not

HVO_2461 - CBS domain protein

HVO_2460 - DUF2062 family protein

//

>NGHB#2405 1

CMPLX not

HVO_2462 htr37 transducer protein Htr37

HVO_2461 - CBS domain protein

//

>NGHB#2408 -4

CMPLX yes

HVO_2465 sucC succinate--CoA ligase (ADP-forming) beta subunit

HVO_2464 sucD succinate--CoA ligase (ADP-forming) alpha subunit

//

>NGHB#2409 491

CMPLX not

HVO_2466 - ISH3-type transposase ISH51

HVO_2465 sucC succinate--CoA ligase (ADP-forming) beta subunit

//

>NGHB#2411 108

CMPLX not

HVO_2467 qor3 NADPH:quinone reductase

HVO_2468 - probable oxidoreductase (short-chain dehydrogenase family)

//

>NGHB#2412 262

CMPLX not

HVO_2468 - probable oxidoreductase (short-chain dehydrogenase family)

HVO_2469 - SNF family transport protein

//

>NGHB#2413 172

CMPLX not

HVO_2469 - SNF family transport protein

HVO_2470 - SNF family transport protein

//

>NGHB#2414 226

CMPLX not

HVO_2470 - SNF family transport protein

HVO_2471 pccB2 propionyl-CoA carboxylase carboxyltransferase component

//

>NGHB#2415 -4

CMPLX yes

HVO_2471 pccB2 propionyl-CoA carboxylase carboxyltransferase component

HVO_2472 pccX propionyl-CoA carboxylase small subunit

//

>NGHB#2416 63

CMPLX ---

HVO_2472 pccX propionyl-CoA carboxylase small subunit

HVO_2473 - hypothetical protein

//

>NGHB#2418 208

CMPLX not

HVO_2475 rps17e 30S ribosomal protein S17e

HVO_2474 glpQ1, ugpQ1 glycerophosphodiester phosphodiesterase

//

>NGHB#2419 24

CMPLX not

HVO_2476 - DUF447 family protein

HVO_2475 rps17e 30S ribosomal protein S17e

//

>NGHB#2420 -4

CMPLX not

HVO_2477 citG triphosphoribosyl-dephospho-CoA synthase

HVO_2476 - DUF447 family protein

//

>NGHB#2421 -1

CMPLX not

HVO_2478 dpd putative dihydropyrimidine dehydrogenase

HVO_2477 citG triphosphoribosyl-dephospho-CoA synthase

//

>NGHB#2422 -1

CMPLX not

HVO_2479 cofD 2-phospho-L-lactate transferase

HVO_2478 dpd putative dihydropyrimidine dehydrogenase

//

>NGHB#2423 105

CMPLX ---

HVO_2480 - hypothetical protein

HVO_2479 cofD 2-phospho-L-lactate transferase

//

>NGHB#2425 83

CMPLX not

HVO_2481 - HD family hydrolase

HVO_2482 - HD family hydrolase

//

>NGHB#2426 52

CMPLX not

HVO_2482 - HD family hydrolase

HVO_2483 - amidohydrolase domain protein

//

>NGHB#2427 156

CMPLX not

HVO_2483 - amidohydrolase domain protein

HVO_2484 - UspA domain protein

//

>NGHB#2429 68

CMPLX not

HVO_2486 pccA propionyl-CoA carboxylase biotin carboxylase component

HVO_2485 birA HTH domain protein / biotin--[acetyl-CoA-carboxylase] ligase

//

>NGHB#2431 111

CMPLX not

HVO_2487 asd aspartate-semialdehyde dehydrogenase

HVO_2488 - HPP domain protein (nonfunctional)

//

>NGHB#2432 299

CMPLX not

HVO_2488 - HPP domain protein (nonfunctional)

HVO_2489 - small CPxCG-related zinc finger protein

//

>NGHB#2433 109

CMPLX ---

HVO_2489 - small CPxCG-related zinc finger protein

HVO_2490 - conserved hypothetical protein

//

>NGHB#2434 262

CMPLX ---

HVO_2490 - conserved hypothetical protein

HVO_2492 - conserved hypothetical protein

//

>NGHB#2436 2

CMPLX not

HVO_2494 cmk cytidylate kinase

HVO_2493 cbf5 tRNA/rRNA pseudouridine synthase Cbf5

//

>NGHB#2437 218

CMPLX not

HVO_2495 - DUF106 family protein

HVO_2494 cmk cytidylate kinase

//

>NGHB#2438 150

CMPLX not

HVO_2496 adk1 adenylate kinase

HVO_2495 - DUF106 family protein

//

>NGHB#2440 -4

CMPLX ---

HVO_2496A - conserved hypothetical protein

HVO_2497 - UPF0324 family protein

//

>NGHB#2441 91

CMPLX ---

HVO_2497 - UPF0324 family protein

HVO_2498 - conserved hypothetical protein

//

>NGHB#2442 191

CMPLX ---

HVO_2498 - conserved hypothetical protein

HVO_2499 - UspA domain protein

//

>NGHB#2443 -4

CMPLX not

HVO_2499 - UspA domain protein

HVO_2500 cat2 transport protein (probable substrate cationic amino acids)

//

>NGHB#2444 181

CMPLX not

HVO_2500 cat2 transport protein (probable substrate cationic amino acids)

HVO_2501 - alpha/beta hydrolase fold protein

//

>NGHB#2445 338

CMPLX not

HVO_2501 - alpha/beta hydrolase fold protein

HVO_2502 hypE2, hmf HypE family protein

//

>NGHB#2446 54

CMPLX not

HVO_2502 hypE2, hmf HypE family protein

HVO_2503 kynU kynureninase

//

>NGHB#2448 108

CMPLX not

HVO_2505 jamm1 desampylase Jamm1

HVO_2504 - probable oxidoreductase (short-chain dehydrogenase family)

//

>NGHB#2449 -14

CMPLX not

HVO_2506 idiA isopentenyl-diphosphate delta-isomerase, type I

HVO_2505 jamm1 desampylase Jamm1

//

>NGHB#2450 72

CMPLX not

HVO_2507 - Lrp/AsnC family transcription regulator

HVO_2506 idiA isopentenyl-diphosphate delta-isomerase, type I

//

>NGHB#2452 193

CMPLX not

HVO_2508 carA carbamoyl-phosphate synthase (glutamine-hydrolyzing) small subunit

HVO_2509 - sensor box histidine kinase

//

>NGHB#2454 4

CMPLX not

HVO_2511 gatD glutamyl-tRNA(Gln) amidotransferase subunit D

HVO_2510 - GNAT family acetyltransferase

//

>NGHB#2456 96

CMPLX ---

HVO_2512 - conserved hypothetical protein

HVO_2513 - conserved hypothetical protein

//

>NGHB#2458 65

CMPLX not

HVO_2515 - DUF1405 family protein

HVO_2514 cmi2 ArsR family transcription regulator

//

>NGHB#2463 2163

CMPLX ---

HVO_2521 - DUF21/CBS domain protein

HVO_2519 - conserved hypothetical protein

//

>NGHB#2465 624

CMPLX not

HVO_2522 - Lrp/AsnC family transcription regulator

HVO_2523 - small CPxCG-related zinc finger protein

//

>NGHB#2466 168

CMPLX not

HVO_2523 - small CPxCG-related zinc finger protein

HVO_2524 crtB phytoene synthase

//

>NGHB#2467 57

CMPLX not

HVO_2524 crtB phytoene synthase

HVO_2525 - HTH-10 family transcription regulator

//

>NGHB#2468 171

CMPLX not

HVO_2525 - HTH-10 family transcription regulator

HVO_2525A - DMT superfamily transport protein (nonfunctional)

//

>NGHB#2470 24

CMPLX not

HVO_2527 lyeJ, ubiA1 lycopene elongase/hydratase (dihydrobisanhydrobacterioruberin-forming)

HVO_2526 cruF bisanhydrobacterioruberin hydratase

//

>NGHB#2471 -4

CMPLX not

HVO_2528 crtD, crtI carotenoid 3,4-desaturase

HVO_2527 lyeJ, ubiA1 lycopene elongase/hydratase (dihydrobisanhydrobacterioruberin-forming)

//

>NGHB#2473 75

CMPLX ---

HVO_2529 - probable oxidoreductase (short-chain dehydrogenase family)

HVO_2531 - conserved hypothetical protein

//

>NGHB#2475 61

CMPLX ---

HVO_2532 - conserved hypothetical protein

HVO_2530 - homolog to phosphoribosylamine--glycine ligase

//

>NGHB#2476 -4

CMPLX ---

HVO_2533 - conserved hypothetical protein

HVO_2532 - conserved hypothetical protein

//

>NGHB#2478 50

CMPLX not

HVO_2533A - DUF1059 domain protein

HVO_2534 - UspA domain protein

//

>NGHB#2480 88

CMPLX ---

HVO_2536 - TIGR00300 family protein

HVO_2535 - conserved hypothetical protein

//

>NGHB#2481 110

CMPLX ---

HVO_2537 - conserved hypothetical protein

HVO_2536 - TIGR00300 family protein

//

>NGHB#2482 69

CMPLX ---

HVO_2538 - alpha/beta hydrolase fold protein

HVO_2537 - conserved hypothetical protein

//

>NGHB#2483 -4

CMPLX ---

HVO_2539 - conserved hypothetical protein

HVO_2538 - alpha/beta hydrolase fold protein

//

>NGHB#2484 101

CMPLX ---

HVO_2540 - conserved hypothetical protein

HVO_2539 - conserved hypothetical protein

//

>NGHB#2485 170

CMPLX ---

HVO_2541 secY1, sec61a1 protein translocase subunit SecY

HVO_2540 - conserved hypothetical protein

//

>NGHB#2486 3

CMPLX not

HVO_2542 rpl15 50S ribosomal protein L15

HVO_2541 secY1, sec61a1 protein translocase subunit SecY

//

>NGHB#2487 -4

CMPLX yes

HVO_2543 rpl30 50S ribosomal protein L30

HVO_2542 rpl15 50S ribosomal protein L15

//

>NGHB#2488 -1

CMPLX yes

HVO_2544 rps5 30S ribosomal protein S5

HVO_2543 rpl30 50S ribosomal protein L30

//

>NGHB#2489 -4

CMPLX yes

HVO_2545 rpl18 50S ribosomal protein L18

HVO_2544 rps5 30S ribosomal protein S5

//

>NGHB#2490 -1

CMPLX yes

HVO_2546 rpl19e 50S ribosomal protein L19e

HVO_2545 rpl18 50S ribosomal protein L18

//

>NGHB#2491 -1

CMPLX yes

HVO_2547 rpl32e 50S ribosomal protein L32e

HVO_2546 rpl19e 50S ribosomal protein L19e

//

>NGHB#2492 -1

CMPLX yes

HVO_2548 rpl6 50S ribosomal protein L6

HVO_2547 rpl32e 50S ribosomal protein L32e

//

>NGHB#2493 4

CMPLX yes

HVO_2549 rps8 30S ribosomal protein S8

HVO_2548 rpl6 50S ribosomal protein L6

//

>NGHB#2494 3

CMPLX yes

HVO_2550 rps14 30S ribosomal protein S14

HVO_2549 rps8 30S ribosomal protein S8

//

>NGHB#2495 -4

CMPLX yes

HVO_2551 rpl5 50S ribosomal protein L5

HVO_2550 rps14 30S ribosomal protein S14

//

>NGHB#2496 -4

CMPLX yes

HVO_2552 rps4e 30S ribosomal protein S4e

HVO_2551 rpl5 50S ribosomal protein L5

//

>NGHB#2497 -4

CMPLX yes

HVO_2553 rpl24 50S ribosomal protein L24

HVO_2552 rps4e 30S ribosomal protein S4e

//

>NGHB#2498 1

CMPLX yes

HVO_2554 rpl14 50S ribosomal protein L14

HVO_2553 rpl24 50S ribosomal protein L24

//

>NGHB#2499 -1

CMPLX yes

HVO_2555 rps17 30S ribosomal protein S17

HVO_2554 rpl14 50S ribosomal protein L14

//

>NGHB#2500 -10

CMPLX not

HVO_2556 rnp1 ribonuclease P protein component 1

HVO_2555 rps17 30S ribosomal protein S17

//

>NGHB#2501 -1

CMPLX not

HVO_2557 rpl29 50S ribosomal protein L29

HVO_2556 rnp1 ribonuclease P protein component 1

//

>NGHB#2502 0

CMPLX yes

HVO_2558 rps3 30S ribosomal protein S3

HVO_2557 rpl29 50S ribosomal protein L29

//

>NGHB#2503 -1

CMPLX yes

HVO_2559 rpl22 50S ribosomal protein L22

HVO_2558 rps3 30S ribosomal protein S3

//

>NGHB#2504 3

CMPLX yes

HVO_2560 rps19 30S ribosomal protein S19

HVO_2559 rpl22 50S ribosomal protein L22

//

>NGHB#2505 2

CMPLX yes

HVO_2561 rpl2 50S ribosomal protein L2

HVO_2560 rps19 30S ribosomal protein S19

//

>NGHB#2506 2

CMPLX yes

HVO_2562 rpl23 50S ribosomal protein L23

HVO_2561 rpl2 50S ribosomal protein L2

//

>NGHB#2507 -4

CMPLX yes

HVO_2563 rpl4 50S ribosomal protein L4

HVO_2562 rpl23 50S ribosomal protein L23

//

>NGHB#2508 3

CMPLX yes

HVO_2564 rpl3 50S ribosomal protein L3

HVO_2563 rpl4 50S ribosomal protein L4

//

>NGHB#2509 4

CMPLX not

HVO_2565 - DUF171 family protein

HVO_2564 rpl3 50S ribosomal protein L3

//

>NGHB#2510 984

CMPLX ---

HVO_2568 - conserved hypothetical protein

HVO_2565 - DUF171 family protein

//

>NGHB#2511 180

CMPLX ---

HVO_2569 - conserved hypothetical protein

HVO_2568 - conserved hypothetical protein

//

>NGHB#2512 76

CMPLX ---

HVO_2570 - small CPxCG-related zinc finger protein

HVO_2569 - conserved hypothetical protein

//

>NGHB#2513 39

CMPLX not

HVO_2571 - UPF0761 family protein

HVO_2570 - small CPxCG-related zinc finger protein

//

>NGHB#2514 92

CMPLX ---

HVO_2571A - conserved hypothetical protein

HVO_2571 - UPF0761 family protein

//

>NGHB#2516 132

CMPLX not

HVO_2572 - UPF0045 family protein

HVO_2573 mch probable methenyltetrahydrofolate cyclohydrolase

//

>NGHB#2517 -4

CMPLX ---

HVO_2573 mch probable methenyltetrahydrofolate cyclohydrolase

HVO_2574 - conserved hypothetical protein

//

>NGHB#2519 141

CMPLX not

HVO_2576 - DnaJ N-terminal domain protein

HVO_2575 - homolog to translation elongation factor aEF-1 alpha subunit

//

>NGHB#2520 90

CMPLX not

HVO_2577 pyrF orotidine-5'-phosphate decarboxylase

HVO_2576 - DnaJ N-terminal domain protein

//

>NGHB#2521 99

CMPLX not

HVO_2578 - major facilitator superfamily transport protein

HVO_2577 pyrF orotidine-5'-phosphate decarboxylase

//

>NGHB#2522 84

CMPLX not

HVO_2579 nadC nicotinate-nucleotide pyrophosphorylase (carboxylating)

HVO_2578 - major facilitator superfamily transport protein

//

>NGHB#2523 -14

CMPLX not

HVO_2580 nadB L-aspartate oxidase

HVO_2579 nadC nicotinate-nucleotide pyrophosphorylase (carboxylating)

//

>NGHB#2524 -4

CMPLX not

HVO_2581 nadA quinolinate synthase A

HVO_2580 nadB L-aspartate oxidase

//

>NGHB#2525 210

CMPLX not

HVO_2582 - amidohydrolase domain protein (nonfunctional)

HVO_2581 nadA quinolinate synthase A

//

>NGHB#2527 156

CMPLX ---

HVO_2583 hmgA hydroxymethylglutaryl-CoA reductase (NADPH)

HVO_2583A - conserved hypothetical protein

//

>NGHB#2530 187

CMPLX ---

HVO_2585 - cupin 2 barrel domain protein

HVO_2586 - conserved hypothetical protein

//

>NGHB#2531 183

CMPLX ---

HVO_2586 - conserved hypothetical protein

HVO_2587 - conserved hypothetical protein

//

>NGHB#2534 85

CMPLX not

HVO_2589 ansA asparaginase family protein

HVO_2590 - probable oxidoreductase (short-chain dehydrogenase family)

//

>NGHB#2535 69

CMPLX ---

HVO_2590 - probable oxidoreductase (short-chain dehydrogenase family)

HVO_2591 - conserved hypothetical protein

//

>NGHB#2537 115

CMPLX not

HVO_2593 - formate/nitrite family transport protein (nonfunctional)

HVO_2592 - thioredoxin domain protein

//

>NGHB#2539 -1

CMPLX yes

HVO_2595 oadhA2 2-oxoacid dehydrogenase E1 component alpha subunit

HVO_2596 oadhB2 2-oxoacid dehydrogenase E1 component beta subunit

//

>NGHB#2540 31

CMPLX yes

HVO_2596 oadhB2 2-oxoacid dehydrogenase E1 component beta subunit

HVO_2597 oadhL lipoyl-binding domain protein

//

>NGHB#2542 77

CMPLX not

HVO_2599 - metallophosphoesterase domain protein

HVO_2598 ppk2 polyphosphate kinase

//

>NGHB#2544 80

CMPLX not

HVO_2600 map methionine aminopeptidase

HVO_2601 hit2 histidine triad family protein (homolog to bis(5'-nucleosyl)-tetraphosphatase)

//

>NGHB#2545 81

CMPLX not

HVO_2601 hit2 histidine triad family protein (homolog to bis(5'-nucleosyl)-tetraphosphatase)

HVO_2602 - transport protein (probable substrate zinc/cadmium/cobalt)

//

>NGHB#2550 -4

CMPLX not

HVO_2606 - PQQ repeat protein

HVO_2607 - PQQ repeat protein

//

>NGHB#2552 105

CMPLX not

HVO_2610 deoC deoxyribose-phosphate aldolase

HVO_2609 - homolog to sodium/calcium antiporter (nonfunctional)

//

>NGHB#2554 120

CMPLX not

HVO_2611 - DUF63 family protein

HVO_2612 - PfkB family kinase

//

>NGHB#2555 68

CMPLX ---

HVO_2612 - PfkB family kinase

HVO_2613 - conserved hypothetical protein

//

>NGHB#2556 94

CMPLX ---

HVO_2613 - conserved hypothetical protein

HVO_2614 udp2 uridine phosphorylase

//

>NGHB#2557 9

CMPLX not

HVO_2614 udp2 uridine phosphorylase

HVO_2615 - HPP domain protein

//

>NGHB#2560 -1

CMPLX not

HVO_2617 - TrkA-C domain protein

HVO_2618 - TrkA-C domain protein

//

>NGHB#2561 23

CMPLX not

HVO_2618 - TrkA-C domain protein

HVO_2619 samp1 ubiquitin-like modifier protein SAMP1

//

>NGHB#2565 174

CMPLX not

HVO_2622 - probable oxidoreductase (aldo-keto reductase family protein)

HVO_2621A - small CPxCG-related zinc finger protein

//

>NGHB#2567 179

CMPLX not

HVO_2623 maa maltose O-acetyltransferase

HVO_2624 pyrG CTP synthase

//

>NGHB#2568 -1

CMPLX not

HVO_2624 pyrG CTP synthase

HVO_2625 guaAb GMP synthase (glutamine-hydrolyzing) subunit B

//

>NGHB#2569 -4

CMPLX ---

HVO_2625 guaAb GMP synthase (glutamine-hydrolyzing) subunit B

HVO_2626 - conserved hypothetical protein

//

>NGHB#2570 100

CMPLX ---

HVO_2626 - conserved hypothetical protein

HVO_2627 - DUF4397 family protein

//

>NGHB#2571 118

CMPLX not

HVO_2627 - DUF4397 family protein

HVO_2628 - GHMP family kinase (homolog to beta-ribofuranosylaminobenzene 5'-phosphate synthase)

//

>NGHB#2572 86

CMPLX ---

HVO_2628 - GHMP family kinase (homolog to beta-ribofuranosylaminobenzene 5'-phosphate synthase)

HVO_2629 - conserved hypothetical protein

//

>NGHB#2573 84

CMPLX ---

HVO_2629 - conserved hypothetical protein

HVO_2630 - conserved hypothetical protein

//

>NGHB#2574 15

CMPLX ---

HVO_2630 - conserved hypothetical protein

HVO_2631 - conserved hypothetical protein

//

>NGHB#2575 84

CMPLX ---

HVO_2631 - conserved hypothetical protein

HVO_2632 tfs2, rpoM2 transcription elongation factor TFS

//

>NGHB#2576 247

CMPLX ---

HVO_2632 tfs2, rpoM2 transcription elongation factor TFS

HVO_2633 - hypothetical protein

//

>NGHB#2578 108

CMPLX ---

HVO_2635 - alpha/beta hydrolase fold protein

HVO_2634 - conserved hypothetical protein

//

>NGHB#2580 103

CMPLX ---

HVO_2636 pspA PspA domain protein

HVO_2637 - conserved hypothetical protein

//

>NGHB#2582 0

CMPLX not

HVO_2639 - DUF1611 family protein

HVO_2638 - homolog to dipeptide epimerase

//

>NGHB#2583 126

CMPLX ---

HVO_2640 - conserved hypothetical protein

HVO_2639 - DUF1611 family protein

//

>NGHB#2584 96

CMPLX ---

HVO_2641 - conserved hypothetical protein

HVO_2640 - conserved hypothetical protein

//

>NGHB#2585 168

CMPLX ---

HVO_2642 - conserved hypothetical protein

HVO_2641 - conserved hypothetical protein

//

>NGHB#2586 -11

CMPLX ---

HVO_2643 qor1 NADPH:quinone reductase

HVO_2642 - conserved hypothetical protein

//

>NGHB#2588 100

CMPLX not

HVO_2644 - HTH domain protein

HVO_2645 moaB molybdopterin adenylyltransferase

//

>NGHB#2589 96

CMPLX not

HVO_2645 moaB molybdopterin adenylyltransferase

HVO_2646 ilvD dihydroxy-acid dehydratase

//

>NGHB#2591 124

CMPLX ---

HVO_2648 serA2 probable D-2-hydroxyacid dehydrogenase

HVO_2647 - conserved hypothetical protein

//

>NGHB#2592 55

CMPLX ---

HVO_2649 - conserved hypothetical protein

HVO_2648 serA2 probable D-2-hydroxyacid dehydrogenase

//

>NGHB#2594 42

CMPLX ---

HVO_2650 - FAD-dependent oxidoreductase (homolog to geranylgeranyl reductase)

HVO_2651 - conserved hypothetical protein

//

>NGHB#2596 75

CMPLX ---

HVO_2653 - conserved hypothetical protein

HVO_2652 - probable RfbX family transport protein

//

>NGHB#2597 37

CMPLX ---

HVO_2654 narC2 homolog to respiratory nitrate reductase b-type cytochrome subunit

HVO_2653 - conserved hypothetical protein

//

>NGHB#2598 1

CMPLX not

HVO_2655 narB2 Rieske-type (2Fe-2S) iron-sulfur protein

HVO_2654 narC2 homolog to respiratory nitrate reductase b-type cytochrome subunit

//

>NGHB#2599 14

CMPLX ---

HVO_2656 - conserved hypothetical protein

HVO_2655 narB2 Rieske-type (2Fe-2S) iron-sulfur protein

//

>NGHB#2604 76

CMPLX not

HVO_2661 - pyridoxal phosphate-dependent aminotransferase

HVO_2659 - probable tautomerase

//

>NGHB#2605 162

CMPLX not

HVO_2662 - glyoxalase domain protein

HVO_2661 - pyridoxal phosphate-dependent aminotransferase

//

>NGHB#2607 41

CMPLX not

HVO_2663 - probable oxidoreductase (aldo-keto reductase family protein)

HVO_2664 - probable S-adenosylmethionine-dependent methyltransferase

//

>NGHB#2609 105

CMPLX not

HVO_2666 thiD phosphomethylpyrimidine kinase / phosphomethylpyrimidine phosphate kinase

HVO_2665 - HpcH/HpaI aldolase family protein

//

>NGHB#2610 0

CMPLX not

HVO_2667 thiM hydroxyethylthiazole kinase

HVO_2666 thiD phosphomethylpyrimidine kinase / phosphomethylpyrimidine phosphate kinase

//

>NGHB#2611 -4

CMPLX not

HVO_2668 thiE thiamine-phosphate synthase

HVO_2667 thiM hydroxyethylthiazole kinase

//

>NGHB#2613 95

CMPLX not

HVO_2669 - flavodoxin domain protein

HVO_2670 - FAD-dependent oxidoreductase (GlcD/DLD_GlcF/GlpC domain fusion protein)

//

>NGHB#2614 114

CMPLX not

HVO_2670 - FAD-dependent oxidoreductase (GlcD/DLD_GlcF/GlpC domain fusion protein)

HVO_2671 - pyridoxal phosphate-dependent aminotransferase

//

>NGHB#2615 127

CMPLX ---

HVO_2671 - pyridoxal phosphate-dependent aminotransferase

HVO_2672 - conserved hypothetical protein

//

>NGHB#2616 71

CMPLX ---

HVO_2672 - conserved hypothetical protein

HVO_2673 - conserved hypothetical protein

//

>NGHB#2617 226

CMPLX ---

HVO_2673 - conserved hypothetical protein

HVO_2674 mntH1 NRAMP family transport protein MntH (probable substrate manganese)

//

>NGHB#2618 126

CMPLX not

HVO_2674 mntH1 NRAMP family transport protein MntH (probable substrate manganese)

HVO_2675 hisD histidinol dehydrogenase

//

>NGHB#2619 96

CMPLX not

HVO_2675 hisD histidinol dehydrogenase

HVO_2676 - HesB/IscA family iron-sulfur cluster assembly accessory protein

//

>NGHB#2620 39

CMPLX ---

HVO_2676 - HesB/IscA family iron-sulfur cluster assembly accessory protein

HVO_2677 - conserved hypothetical protein

//

>NGHB#2621 88

CMPLX ---

HVO_2677 - conserved hypothetical protein

HVO_2678 - conserved hypothetical protein

//

>NGHB#2622 56

CMPLX ---

HVO_2678 - conserved hypothetical protein

HVO_2679 - conserved hypothetical protein

//

>NGHB#2623 -4

CMPLX ---

HVO_2679 - conserved hypothetical protein

HVO_2680 - conserved hypothetical protein

//

>NGHB#2624 100

CMPLX ---

HVO_2680 - conserved hypothetical protein

HVO_2681 - HTH domain protein

//

>NGHB#2625 93

CMPLX not

HVO_2681 - HTH domain protein

HVO_2682 - dodecin

//

>NGHB#2626 117

CMPLX not

HVO_2682 - dodecin

HVO_2683 - 5'-nucleotidase family hydrolase

//

>NGHB#2627 84

CMPLX not

HVO_2683 - 5'-nucleotidase family hydrolase

HVO_2684 - UspA domain protein

//

>NGHB#2630 129

CMPLX not

HVO_2686 - PAC2 family protein (nonfunctional)

HVO_2688 trmB TrmB family transcription regulator TrmB

//

>NGHB#2632 42

CMPLX ---

HVO_2690 - GFO family oxidoreductase

HVO_2689 - conserved hypothetical protein

//

>NGHB#2633 154

CMPLX ---

HVO_2691 - conserved hypothetical protein

HVO_2690 - GFO family oxidoreductase

//

>NGHB#2634 115

CMPLX ---

HVO_2692 tsgD3 ABC-type transport system ATP-binding protein (probable substrate sugar)

HVO_2691 - conserved hypothetical protein

//

>NGHB#2635 2

CMPLX yes

HVO_2693 tsgC3 ABC-type transport system permease protein (probable substrate sugar)

HVO_2692 tsgD3 ABC-type transport system ATP-binding protein (probable substrate sugar)

//

>NGHB#2636 1

CMPLX yes

HVO_2694 tsgB3 ABC-type transport system permease protein (probable substrate sugar)

HVO_2693 tsgC3 ABC-type transport system permease protein (probable substrate sugar)

//

>NGHB#2637 50

CMPLX yes

HVO_2695 tsgA3 ABC-type transport system periplasmic substrate-binding protein (probable substrate sugar)

HVO_2694 tsgB3 ABC-type transport system permease protein (probable substrate sugar)

//

>NGHB#2638 154

CMPLX not

HVO_2696 - probable oxidoreductase (aldo-keto reductase family protein)

HVO_2695 tsgA3 ABC-type transport system periplasmic substrate-binding protein (probable substrate sugar)

//

>NGHB#2640 -4

CMPLX not

HVO_2697 priS DNA primase small subunit

HVO_2698 ginS DNA replication factor GINS

//

>NGHB#2641 36

CMPLX not

HVO_2698 ginS DNA replication factor GINS

HVO_2699 bcp1 peroxiredoxin

//

>NGHB#2643 0

CMPLX ---

HVO_2701 - conserved hypothetical protein

HVO_2700 cdc48b AAA-type ATPase (CDC48 subfamily)

//

>NGHB#2647 84

CMPLX ---

HVO_2704 - DUF1628 domain protein

HVO_2705 - conserved hypothetical protein

//

>NGHB#2648 94

CMPLX ---

HVO_2705 - conserved hypothetical protein

HVO_2706 - eIF-2B domain protein

//

>NGHB#2650 3

CMPLX not

HVO_2708 - YfiH family protein

HVO_2707 - GFO family oxidoreductase

//

>NGHB#2651 109

CMPLX not

HVO_2709 - GNAT family acetyltransferase

HVO_2708 - YfiH family protein

//

>NGHB#2653 91

CMPLX not

HVO_2710 - homolog to archaease

HVO_2711 - DoxX domain protein

//

>NGHB#2654 37

CMPLX not

HVO_2711 - DoxX domain protein

HVO_2712 rtcB tRNA-splicing ligase RtcB

//

>NGHB#2656 105

CMPLX ---

HVO_2714 - conserved hypothetical protein

HVO_2713 - conserved hypothetical protein

//

>NGHB#2657 209

CMPLX ---

HVO_2715 - HAD superfamily hydrolase

HVO_2714 - conserved hypothetical protein

//

>NGHB#2658 99

CMPLX not

HVO_2717 alaS2 alanine--tRNA ligase

HVO_2716 acd5 acyl-CoA dehydrogenase

//

>NGHB#2659 35

CMPLX not

HVO_2718 - cro/C1 family transcription regulator

HVO_2717 alaS2 alanine--tRNA ligase

//

>NGHB#2662 377

CMPLX not

HVO_2722 rpl37e 50S ribosomal protein L37e

HVO_2721 purF amidophosphoribosyltransferase

//

>NGHB#2663 -4

CMPLX not

HVO_2723 lsm, snp RNA-binding protein Lsm

HVO_2722 rpl37e 50S ribosomal protein L37e

//

>NGHB#2665 -1

CMPLX not

HVO_2724 rnj ribonuclease J

HVO_2725 idsA1 bifunctional short chain isoprenyl diphosphate synthase

//

>NGHB#2666 189

CMPLX not

HVO_2725 idsA1 bifunctional short chain isoprenyl diphosphate synthase

HVO_2726 gltS glutamate--tRNA(Glu/Gln) ligase

//

>NGHB#2667 66

CMPLX not

HVO_2726 gltS glutamate--tRNA(Glu/Gln) ligase

HVO_2727 - cyclase family protein

//

>NGHB#2670 130

CMPLX not

HVO_2729 ferB2 ferredoxin (3Fe-4S)(4Fe-4S), zinc-containing

HVO_2730 etfB2 electron transfer flavoprotein beta subunit

//

>NGHB#2671 2

CMPLX yes

HVO_2730 etfB2 electron transfer flavoprotein beta subunit

HVO_2731 etfA2 electron transfer flavoprotein alpha subunit

//

>NGHB#2672 -4

CMPLX not

HVO_2731 etfA2 electron transfer flavoprotein alpha subunit

HVO_2732 - FixC family protein

//

>NGHB#2673 13

CMPLX ---

HVO_2732 - FixC family protein

HVO_2733 - conserved hypothetical protein

//

>NGHB#2674 98

CMPLX ---

HVO_2733 - conserved hypothetical protein

HVO_2734 - GNAT family acetyltransferase

//

>NGHB#2676 78

CMPLX ---

HVO_2736 tmcA tRNA(Met) cytidine acetyltransferase TmcA

HVO_2735 - conserved hypothetical protein

//

>NGHB#2678 6

CMPLX yes

HVO_2737 rpl8e, rpl7ae 50S ribosomal protein L8e

HVO_2738 rps28e 30S ribosomal protein S28e

//

>NGHB#2679 2

CMPLX yes

HVO_2738 rps28e 30S ribosomal protein S28e

HVO_2739 rpl24e 50S ribosomal protein L24e

//

>NGHB#2680 0

CMPLX not

HVO_2739 rpl24e 50S ribosomal protein L24e

HVO_2740 ndk nucleoside-diphosphate kinase

//

>NGHB#2681 105

CMPLX ---

HVO_2740 ndk nucleoside-diphosphate kinase

HVO_2741 - hypothetical protein

//

>NGHB#2683 -4

CMPLX not

HVO_2743 metE2 5-methyltetrahydropteroyltriglutamate--homocysteine S-methyltransferase (methionine synthase II)

HVO_2742 metE1 5-methyltetrahydropteroyltriglutamate--homocysteine S-methyltransferase (methionine synthase II)

//

>NGHB#2684 59

CMPLX not

HVO_2744 prmC release factor glutamine methyltransferase PrmC

HVO_2743 metE2 5-methyltetrahydropteroyltriglutamate--homocysteine S-methyltransferase (methionine synthase II)

//

>NGHB#2685 -4

CMPLX not

HVO_2745 mscS5 mechanosensitive channel protein MscS

HVO_2744 prmC release factor glutamine methyltransferase PrmC

//

>NGHB#2686 3

CMPLX not

HVO_2746 ksgA ribosome biogenesis protein KsgA, 16S rRNA-methylating

HVO_2745 mscS5 mechanosensitive channel protein MscS

//

>NGHB#2687 42

CMPLX not

HVO_2747 - putative tRNA-specific adenosine deaminase

HVO_2746 ksgA ribosome biogenesis protein KsgA, 16S rRNA-methylating

//

>NGHB#2688 53

CMPLX not

HVO_2748 rpoF DNA-directed RNA polymerase subunit F

HVO_2747 - putative tRNA-specific adenosine deaminase

//

>NGHB#2689 3

CMPLX not

HVO_2749 rpl21e 50S ribosomal protein L21e

HVO_2748 rpoF DNA-directed RNA polymerase subunit F

//

>NGHB#2691 130

CMPLX ---

HVO_2750 metB1 cystathionine synthase/lyase (cystathionine gamma-synthase, cystathionine gamma-lyase, cystathionine beta-lyase)

HVO_2751 - conserved hypothetical protein

//

>NGHB#2693 2

CMPLX not

HVO_2753 - small CPxCG-related zinc finger protein

HVO_2752 tef1b translation elongation factor aEF-1 beta

//

>NGHB#2694 83

CMPLX not

HVO_2754 - DUF112 family protein

HVO_2753 - small CPxCG-related zinc finger protein

//

>NGHB#2695 208

CMPLX not

HVO_2755 rpl12 50S ribosomal protein L12

HVO_2754 - DUF112 family protein

//

>NGHB#2696 21

CMPLX yes

HVO_2756 rpl10 50S ribosomal protein L10

HVO_2755 rpl12 50S ribosomal protein L12

//

>NGHB#2697 -4

CMPLX yes

HVO_2757 rpl1 50S ribosomal protein L1

HVO_2756 rpl10 50S ribosomal protein L10

//

>NGHB#2698 305

CMPLX yes

HVO_2758 rpl11 50S ribosomal protein L11

HVO_2757 rpl1 50S ribosomal protein L1

//

>NGHB#2699 116

CMPLX not

HVO_2759 - peptidase M42 family protein

HVO_2758 rpl11 50S ribosomal protein L11

//

>NGHB#2701 159

CMPLX ---

HVO_2760 - conserved hypothetical protein

HVO_2761 mvk mevalonate kinase

//

>NGHB#2702 -4

CMPLX not

HVO_2761 mvk mevalonate kinase

HVO_2762 - isopentenyl phosphate kinase

//

>NGHB#2705 -4

CMPLX yes

HVO_2764 dppB6 ABC-type transport system permease protein (probable substrate dipeptide/oligopeptide)

HVO_2765 dppC6 ABC-type transport system permease protein (probable substrate dipeptide/oligopeptide)

//

>NGHB#2706 90

CMPLX not

HVO_2765 dppC6 ABC-type transport system permease protein (probable substrate dipeptide/oligopeptide)

HVO_2766 lhr1 ATP-dependent DNA helicase

//

>NGHB#2709 101

CMPLX ---

HVO_2768 - conserved hypothetical protein (nonfunctional)

HVO_2769 - conserved hypothetical protein

//

>NGHB#2713 174

CMPLX not

HVO_2773 rps2 30S ribosomal protein S2

HVO_2772 - rhodanese domain protein / beta-lactamase domain protein

//

>NGHB#2714 -4

CMPLX not

HVO_2774 eno enolase

HVO_2773 rps2 30S ribosomal protein S2

//

>NGHB#2715 4

CMPLX not

HVO_2775 rpoK DNA-directed RNA polymerase subunit K

HVO_2774 eno enolase

//

>NGHB#2716 -4

CMPLX yes

HVO_2776 rpoN DNA-directed RNA polymerase subunit N

HVO_2775 rpoK DNA-directed RNA polymerase subunit K

//

>NGHB#2717 12

CMPLX not

HVO_2777 rps9 30S ribosomal protein S9

HVO_2776 rpoN DNA-directed RNA polymerase subunit N

//

>NGHB#2718 -7

CMPLX yes

HVO_2778 rpl13 50S ribosomal protein L13

HVO_2777 rps9 30S ribosomal protein S9

//

>NGHB#2719 -4

CMPLX yes

HVO_2779 rpl18e 50S ribosomal protein L18e

HVO_2778 rpl13 50S ribosomal protein L13

//

>NGHB#2720 255

CMPLX not

HVO_2781 rpoD DNA-directed RNA polymerase subunit D

HVO_2779 rpl18e 50S ribosomal protein L18e

//

>NGHB#2721 6

CMPLX not

HVO_2782 rps11 30S ribosomal protein S11

HVO_2781 rpoD DNA-directed RNA polymerase subunit D

//

>NGHB#2722 -4

CMPLX yes

HVO_2783 rps4 30S ribosomal protein S4

HVO_2782 rps11 30S ribosomal protein S11

//

>NGHB#2723 -4

CMPLX yes

HVO_2784 rps13 30S ribosomal protein S13

HVO_2783 rps4 30S ribosomal protein S4

//

>NGHB#2726 247

CMPLX not

HVO_2788 - UspA domain protein

HVO_2787 - small CPxCG-related zinc finger protein

//

>NGHB#2727 52

CMPLX not

HVO_2789 moaA GTP 3',8-cyclase

HVO_2788 - UspA domain protein

//

>NGHB#2729 -4

CMPLX ---

HVO_2790 apbC1 Fe-S cluster carrier protein ApbC

HVO_2791 - conserved hypothetical protein

//

>NGHB#2730 272

CMPLX ---

HVO_2791 - conserved hypothetical protein

HVO_2792 udg2 uracil-DNA glycosylase superfamily protein

//

>NGHB#2731 82

CMPLX ---

HVO_2792 udg2 uracil-DNA glycosylase superfamily protein

HVO_2793 - conserved hypothetical protein

//

>NGHB#2736 119

CMPLX ---

HVO_2798 livJ3 ABC-type transport system periplasmic substrate-binding protein (probable substrate branched-chain amino acids)

HVO_2799 - conserved hypothetical protein

//

>NGHB#2738 -4

CMPLX yes

HVO_2801 livG3 ABC-type transport system ATP-binding protein (probable substrate branched-chain amino acids)

HVO_2800 livF3 ABC-type transport system ATP-binding protein (probable substrate branched-chain amino acids)

//

>NGHB#2739 -8

CMPLX yes

HVO_2802 livM3 ABC-type transport system permease protein (probable substrate branched-chain amino acids)

HVO_2801 livG3 ABC-type transport system ATP-binding protein (probable substrate branched-chain amino acids)

//

>NGHB#2740 -4

CMPLX yes

HVO_2803 livH3 ABC-type transport system permease protein (probable substrate branched-chain amino acids)

HVO_2802 livM3 ABC-type transport system permease protein (probable substrate branched-chain amino acids)

//

>NGHB#2745 466

CMPLX not

HVO_2808 sdhA succinate dehydrogenase subunit A

HVO_2806 - HTH domain protein

//

>NGHB#2746 55

CMPLX yes

HVO_2809 sdhB succinate dehydrogenase subunit B

HVO_2808 sdhA succinate dehydrogenase subunit A

//

>NGHB#2747 -1

CMPLX yes

HVO_2810 sdhD succinate dehydrogenase subunit D

HVO_2809 sdhB succinate dehydrogenase subunit B

//

>NGHB#2748 -1

CMPLX yes

HVO_2811 sdhC succinate dehydrogenase subunit C

HVO_2810 sdhD succinate dehydrogenase subunit D

//

>NGHB#2749 109

CMPLX not

HVO_2812 - AstE domain protein

HVO_2811 sdhC succinate dehydrogenase subunit C

//

>NGHB#2751 113

CMPLX not

HVO_2813 - RimK family protein

HVO_2814 - DNA N-glycosylase

//

>NGHB#2752 141

CMPLX not

HVO_2814 - DNA N-glycosylase

HVO_2815 hbd1 3-hydroxyacyl-CoA dehydrogenase / enoyl-CoA hydratase

//

>NGHB#2753 318

CMPLX not

HVO_2815 hbd1 3-hydroxyacyl-CoA dehydrogenase / enoyl-CoA hydratase

HVO_2815_A - XerC/D-like integrase

//

>NGHB#2754 530

CMPLX not

HVO_2815_A - XerC/D-like integrase

HVO_2817 - ISH3-type transposase ISH51

//

>NGHB#2755 187

CMPLX not

HVO_2819 - XerC/D-like integrase

HVO_2820 - ISH3-type transposase ISH51

//

>NGHB#2758 29

CMPLX ---

HVO_2822 - conserved hypothetical protein

HVO_2823 - conserved hypothetical protein

//

>NGHB#2759 -11

CMPLX ---

HVO_2823 - conserved hypothetical protein

HVO_2824 - conserved hypothetical protein

//

>NGHB#2760 65

CMPLX ---

HVO_2824 - conserved hypothetical protein

HVO_2825 - conserved hypothetical protein

//

>NGHB#2761 -4

CMPLX ---

HVO_2825 - conserved hypothetical protein

HVO_2826 - conserved hypothetical protein

//

>NGHB#2762 -11

CMPLX ---

HVO_2826 - conserved hypothetical protein

HVO_2827 - UvrD/REP family helicase

//

>NGHB#2764 368

CMPLX not

HVO_2830 - ISH8-type transposase ISHvo12

HVO_2829 - integrase family protein

//

>NGHB#2765 1267

CMPLX ---

HVO_2832 - conserved hypothetical protein (nonfunctional)

HVO_2830 - ISH8-type transposase ISHvo12

//

>NGHB#2766 0

CMPLX ---

HVO_2833 - ArsR family transcription regulator

HVO_2832 - conserved hypothetical protein (nonfunctional)

//

>NGHB#2767 119

CMPLX not

HVO_2834 mutY2 probable G/T mismatch glycosylase

HVO_2833 - ArsR family transcription regulator

//

>NGHB#2769 182

CMPLX ---

HVO_2834_A - conserved hypothetical protein

HVO_2835 - histidine kinase

//

>NGHB#2770 64

CMPLX not

HVO_2835 - histidine kinase

HVO_2836 - sensor/bat box HTH-10 family transcription regulator

//

>NGHB#2771 106

CMPLX ---

HVO_2836 - sensor/bat box HTH-10 family transcription regulator

HVO_2837 - conserved hypothetical protein

//

>NGHB#2774 322

CMPLX not

HVO_2839 - ArsR family transcription regulator

HVO_2840 - sensor box histidine kinase

//

>NGHB#2777 254

CMPLX not

HVO_2842 - DUF4010 family protein

HVO_2844 bdbC, dsbB disulfide bond formation protein

//

>NGHB#2779 104

CMPLX ---

HVO_2845 - conserved hypothetical protein

HVO_2843 phr1 deoxyribodipyrimidine photo-lyase

//

>NGHB#2780 8

CMPLX ---

HVO_2846 - SpoVR family protein

HVO_2845 - conserved hypothetical protein

//

>NGHB#2781 -4

CMPLX not

HVO_2847 - UPF0229 family protein

HVO_2846 - SpoVR family protein

//

>NGHB#2782 -10

CMPLX not

HVO_2848 prkA1 probable PrkA-type serine/threonine protein kinase

HVO_2847 - UPF0229 family protein

//

>NGHB#2783 2

CMPLX not

HVO_2849 prkA2 probable PrkA-type serine/threonine protein kinase

HVO_2848 prkA1 probable PrkA-type serine/threonine protein kinase

//

>NGHB#2785 24

CMPLX ---

HVO_2850 - conserved hypothetical protein

HVO_2851 - UPF0179 family protein

//

>NGHB#2787 119

CMPLX not

HVO_2853 - DUF309 family protein

HVO_2852 - AstE domain protein

//

>NGHB#2791 115

CMPLX not

HVO_2857 suhB1 probable inositol-1(or 4)-monophosphatase / fructose-1,6-bisphosphatase, archaeal-type

HVO_2858 - Lrp/AsnC family transcription regulator

//

>NGHB#2792 113

CMPLX not

HVO_2858 - Lrp/AsnC family transcription regulator

HVO_2859 - DUF63 family protein

//

>NGHB#2794 50

CMPLX ---

HVO_2861 - conserved hypothetical protein

HVO_2860 - DUF181 family protein

//

>NGHB#2796 97

CMPLX not

HVO_2862 glyA1 serine hydroxymethyltransferase

HVO_2863 - DUF211 family protein

//

>NGHB#2797 -1

CMPLX not

HVO_2863 - DUF211 family protein

HVO_2864 - DUF125 family protein

//

>NGHB#2798 99

CMPLX not

HVO_2864 - DUF125 family protein

HVO_2865 folD methylenetetrahydrofolate dehydrogenase / methenyltetrahydrofolate cyclohydrolase

//

>NGHB#2800 53

CMPLX ---

HVO_2867 - conserved hypothetical protein

HVO_2866 - conserved hypothetical protein

//

>NGHB#2802 637

CMPLX ---

HVO_2868 - conserved hypothetical protein

HVO_2869 - PadR family transcription regulator

//

>NGHB#2806 66

CMPLX ---

HVO_2873 fen1 flap endonuclease Fen1

HVO_2872 - conserved hypothetical protein

//

>NGHB#2807 27

CMPLX not

HVO_2874 - GNAT family acetyltransferase

HVO_2873 fen1 flap endonuclease Fen1

//

>NGHB#2809 21

CMPLX not

HVO_2875 - probable S-adenosylmethionine-dependent methyltransferase

HVO_2876 - DUF3054 family protein

//

>NGHB#2812 73

CMPLX ---

HVO_2878 - conserved hypothetical protein

HVO_2879 - ornithine cyclodeaminase family protein

//

>NGHB#2814 47

CMPLX ---

HVO_2880 pelA mRNA surveillance protein pelota

HVO_2879A - conserved hypothetical protein

//

>NGHB#2815 34

CMPLX not

HVO_2881 - DUF4013 family protein

HVO_2880 pelA mRNA surveillance protein pelota

//

>NGHB#2816 77

CMPLX not

HVO_2882 - DUF4013 family protein

HVO_2881 - DUF4013 family protein

//

>NGHB#2817 183

CMPLX not

HVO_2883 - DUF814 domain protein

HVO_2882 - DUF4013 family protein

//

>NGHB#2819 289

CMPLX ---

HVO_2884 - conserved hypothetical protein

HVO_2885 gltP2 SDF family transport protein (probable substrate glutamate/aspartate)

//

>NGHB#2820 87

CMPLX not

HVO_2885 gltP2 SDF family transport protein (probable substrate glutamate/aspartate)

HVO_2886 - GNAT family acetyltransferase

//

>NGHB#2822 83

CMPLX not

HVO_2888 elp3 homolog to elongator complex protein ELP3

HVO_2887 mscS4 mechanosensitive channel protein MscS

//

>NGHB#2823 134

CMPLX not

HVO_2889 - DHH/RecJ family phosphoesterase

HVO_2888 elp3 homolog to elongator complex protein ELP3

//

>NGHB#2824 126

CMPLX not

HVO_2890 - DUF649 family protein

HVO_2889 - DHH/RecJ family phosphoesterase

//

>NGHB#2825 8

CMPLX not

HVO_2891 tdk thymidine kinase

HVO_2890 - DUF649 family protein

//

>NGHB#2826 118

CMPLX not

HVO_2892_A - homolog to NAD(P)H dehydrogenase (quinone)

HVO_2891 tdk thymidine kinase

//

>NGHB#2829 34

CMPLX not

HVO_2895 - alpha/beta hydrolase fold protein

HVO_2894 - HTH-10 family transcription regulator

//

>NGHB#2830 108

CMPLX not

HVO_2896 mutY1 A/G-specific adenine glycosylase

HVO_2895 - alpha/beta hydrolase fold protein

//

>NGHB#2834 147

CMPLX not

HVO_2899 bolA BolA family protein

HVO_2900 fumC fumarate hydratase

//

>NGHB#2835 235

CMPLX not

HVO_2900 fumC fumarate hydratase

HVO_2901 - small CPxCG-related zinc finger protein

//

>NGHB#2836 47

CMPLX not

HVO_2901 - small CPxCG-related zinc finger protein

HVO_2902 gatE glutamyl-tRNA(Gln) amidotransferase subunit E

//

>NGHB#2838 1

CMPLX ---

HVO_2904 htpX2 HtpX-like protease

HVO_2903 - conserved hypothetical protein

//

>NGHB#2841 -4

CMPLX ---

HVO_2907 - conserved hypothetical protein

HVO_2906 - tRNA (cytidine/uridine-2'-O-)-methyltransferase

//

>NGHB#2843 188

CMPLX not

HVO_2908 folP2, dchpS dihydropteroate synthase

HVO_2909 mptE 6-hydroxymethyl-7,8-dihydropterin pyrophosphokinase MptE

//

>NGHB#2846 84

CMPLX not

HVO_2911 phr2 deoxyribodipyrimidine photo-lyase

HVO_2912 - ThiJ/PfpI domain protein

//

>NGHB#2847 137

CMPLX not

HVO_2912 - ThiJ/PfpI domain protein

HVO_2913 sod2 superoxide dismutase (Mn)

//

>NGHB#2850 101

CMPLX ---

HVO_2915 - conserved hypothetical protein

HVO_2916 - probable oxidoreductase (short-chain dehydrogenase family)

//

>NGHB#2853 -4

CMPLX not

HVO_2918 hts, thyA thymidylate synthase

HVO_2919 hdrB, folA2 dihydrofolate reductase

//

>NGHB#2854 -8

CMPLX not

HVO_2919 hdrB, folA2 dihydrofolate reductase

HVO_2920 - DUF4177 family protein

//

>NGHB#2855 116

CMPLX ---

HVO_2920 - DUF4177 family protein

HVO_2921 - conserved hypothetical protein

//

>NGHB#2856 75

CMPLX ---

HVO_2921 - conserved hypothetical protein

HVO_2922 - UPF0339 family protein

//

>NGHB#2857 159

CMPLX not

HVO_2922 - UPF0339 family protein

HVO_2923 psmA2 proteasome alpha subunit

//

>NGHB#2858 0

CMPLX not

HVO_2923 psmA2 proteasome alpha subunit

HVO_2924 - MOSC domain protein

//

>NGHB#2859 98

CMPLX not

HVO_2924 - MOSC domain protein

HVO_2926 - amidohydrolase domain protein (nonfunctional)

//

>NGHB#2862 108

CMPLX not

HVO_2929 - probable nucleoside deaminase

HVO_2930 - GNAT family acetyltransferase

//

>NGHB#2864 54

CMPLX ---

HVO_2932 - GTP-binding protein

HVO_2931 - conserved hypothetical protein

//

>NGHB#2866 18

CMPLX ---

HVO_2933 - conserved hypothetical protein

HVO_2934 - conserved hypothetical protein

//

>NGHB#2871 60

CMPLX ---

HVO_2942 - conserved hypothetical protein

HVO_2943 pyrD dihydroorotate dehydrogenase (quinone)

//

>NGHB#2872 94

CMPLX ---

HVO_2943 pyrD dihydroorotate dehydrogenase (quinone)

HVO_2944 - conserved hypothetical protein

//

>NGHB#2874 91

CMPLX not

HVO_2946 metB2 cystathionine synthase/lyase (cystathionine gamma-synthase, cystathionine gamma-lyase, cystathionine beta-lyase)

HVO_2945 valS valine--tRNA ligase

//

>NGHB#2875 247

CMPLX not

HVO_2947 pheT phenylalanine--tRNA ligase beta subunit

HVO_2946 metB2 cystathionine synthase/lyase (cystathionine gamma-synthase, cystathionine gamma-lyase, cystathionine beta-lyase)

//

>NGHB#2876 -1

CMPLX yes

HVO_2948 pheS phenylalanine--tRNA ligase alpha subunit

HVO_2947 pheT phenylalanine--tRNA ligase beta subunit

//

>NGHB#2877 279

CMPLX not

HVO_2949 - FAD-dependent oxidoreductase

HVO_2948 pheS phenylalanine--tRNA ligase alpha subunit

//

>NGHB#2880 -4

CMPLX not

HVO_2952 endA tRNA-splicing endonuclease

HVO_2951 trpS1 tryptophan--tRNA ligase

//

>NGHB#2881 185

CMPLX not

HVO_2953 - homolog to DNA topoisomerase 1

HVO_2952 endA tRNA-splicing endonuclease

//

>NGHB#2885 296

CMPLX not

HVO_2957 lipA lipoate synthase

HVO_2958 oadhA1 2-oxo-3-methylvalerate dehydrogenase E1 component alpha subunit

//

>NGHB#2886 -4

CMPLX yes

HVO_2958 oadhA1 2-oxo-3-methylvalerate dehydrogenase E1 component alpha subunit

HVO_2959 oadhB1 2-oxo-3-methylvalerate dehydrogenase E1 component beta subunit

//

>NGHB#2887 3

CMPLX yes

HVO_2959 oadhB1 2-oxo-3-methylvalerate dehydrogenase E1 component beta subunit

HVO_2960 dsa1 dihydrolipoamide S-acyltransferase

//

>NGHB#2888 -1

CMPLX not

HVO_2960 dsa1 dihydrolipoamide S-acyltransferase

HVO_2961 lpdA dihydrolipoyl dehydrogenase

//

>NGHB#2890 -4

CMPLX not

HVO_2963 - DUF2178 family protein

HVO_2962 - cro/C1 family transcription regulator

//

>NGHB#2891 204

CMPLX ---

HVO_2964 - conserved hypothetical protein

HVO_2963 - DUF2178 family protein

//

>NGHB#2892 51

CMPLX ---

HVO_2965 serB phosphoserine phosphatase

HVO_2964 - conserved hypothetical protein

//

>NGHB#2897 145

CMPLX ---

HVO_2969 thrC3 threonine synthase

HVO_2968A - conserved hypothetical protein

//

>NGHB#2898 94

CMPLX not

HVO_2970 - TrmB family transcription regulator

HVO_2969 thrC3 threonine synthase

//

>NGHB#2899 82

CMPLX ---

HVO_2971 - conserved hypothetical protein

HVO_2970 - TrmB family transcription regulator

//

>NGHB#2900 96

CMPLX ---

HVO_2972 - conserved hypothetical protein

HVO_2971 - conserved hypothetical protein

//

>NGHB#2901 72

CMPLX ---

HVO_2973 - conserved hypothetical protein

HVO_2972 - conserved hypothetical protein

//

>NGHB#2903 -4

CMPLX ---

HVO_2974 - cupin 2 barrel domain protein

HVO_2975 - conserved hypothetical protein

//

>NGHB#2905 134

CMPLX not

HVO_2977 arsA2 ArsA family ATPase

HVO_2976 cstA carbon starvation protein CstA

//

>NGHB#2906 1

CMPLX ---

HVO_2978 - conserved hypothetical protein

HVO_2977 arsA2 ArsA family ATPase

//

>NGHB#2908 -4

CMPLX ---

HVO_2979 - CobW domain protein

HVO_2980 - conserved hypothetical protein

//

>NGHB#2909 108

CMPLX ---

HVO_2980 - conserved hypothetical protein

HVO_2981 upp uracil phosphoribosyltransferase

//

>NGHB#2910 160

CMPLX not

HVO_2981 upp uracil phosphoribosyltransferase

HVO_2982 - small CPxCG-related zinc finger protein

//

>NGHB#2912 141

CMPLX ---

HVO_2983_A - conserved hypothetical protein

HVO_2983 - IMPACT family protein

//

>NGHB#2913 75

CMPLX ---

HVO_2984 trpY probable repressor of tryptophan metabolism TrpY

HVO_2983_A - conserved hypothetical protein

//

>NGHB#2920 242

CMPLX ---

HVO_2991 - conserved hypothetical protein

HVO_2990 - hypothetical protein

//

>NGHB#2921 90

CMPLX ---

HVO_2992 hisI phosphoribosyl-AMP cyclohydrolase

HVO_2991 - conserved hypothetical protein

//

>NGHB#2922 81

CMPLX not

HVO_2993 pibD prepilin/prearchaellin peptidase

HVO_2992 hisI phosphoribosyl-AMP cyclohydrolase

//

>NGHB#2923 24

CMPLX ---

HVO_2994 - conserved hypothetical protein (nonfunctional)

HVO_2993 pibD prepilin/prearchaellin peptidase

//

>NGHB#2924 118

CMPLX ---

HVO_2995 fdx, ferA5 ferredoxin (2Fe-2S)

HVO_2994 - conserved hypothetical protein (nonfunctional)

//

>NGHB#2925 140

CMPLX not

HVO_2996 - transport protein (probable substrate phosphate/sulfate)

HVO_2995 fdx, ferA5 ferredoxin (2Fe-2S)

//

>NGHB#2926 104

CMPLX not

HVO_2997 metY2 O-acetylhomoserine aminocarboxypropyltransferase (methionine synthase)

HVO_2996 - transport protein (probable substrate phosphate/sulfate)

//

>NGHB#2927 132

CMPLX not

HVO_2998 metX homoserine O-acetyltransferase

HVO_2997 metY2 O-acetylhomoserine aminocarboxypropyltransferase (methionine synthase)

//

>NGHB#2928 -4

CMPLX not

HVO_2999 metY1 O-acetylhomoserine aminocarboxypropyltransferase (methionine synthase)

HVO_2998 metX homoserine O-acetyltransferase

//

>NGHB#2931 -4

CMPLX yes

HVO_3002 - ABC-type transport system ATP-binding protein

HVO_3001 - ABC-type transport system permease protein

//

>NGHB#2932 114

CMPLX ---

HVO_3003 - conserved hypothetical protein

HVO_3002 - ABC-type transport system ATP-binding protein

//

>NGHB#2938 80

CMPLX not

HVO_3008 - UPF0148 family protein

HVO_3009 - UspA domain protein

//

>NGHB#2940 85

CMPLX ---

HVO_3011 - conserved hypothetical protein

HVO_3010 hef ATP-dependent RNA helicase/nuclease Hef

//

>NGHB#2941 146

CMPLX ---

HVO_3012 oapC origin-associated protein OapC

HVO_3011 - conserved hypothetical protein

//

>NGHB#2942 48

CMPLX not

HVO_3013 oapB origin-associated protein OapB

HVO_3012 oapC origin-associated protein OapC

//

>NGHB#2943 6

CMPLX not

HVO_3014 oapA origin-associated GTP-binding protein OapA

HVO_3013 oapB origin-associated protein OapB

//

>NGHB#2944 293

CMPLX ---

HVO_C0001 orc10 Orc1-type DNA replication protein

HVO_C0002 - conserved hypothetical protein

//

>NGHB#2945 285

CMPLX ---

HVO_C0002 - conserved hypothetical protein

HVO_C0003 - conserved hypothetical protein

//

>NGHB#2948 0

CMPLX ---

HVO_C0007 - conserved hypothetical protein

HVO_C0006 - UPF0395 family protein

//

>NGHB#2949 131

CMPLX ---

HVO_C0008 - hypothetical protein

HVO_C0007 - conserved hypothetical protein

//

>NGHB#2951 171

CMPLX ---

HVO_C0009 - hypothetical protein

HVO_C0010 - hypothetical protein

//

>NGHB#2952 219

CMPLX ---

HVO_C0010 - hypothetical protein

HVO_C0011 - hypothetical protein

//

>NGHB#2953 210

CMPLX ---

HVO_C0011 - hypothetical protein

HVO_C0012 - conserved hypothetical protein

//

>NGHB#2954 0

CMPLX ---

HVO_C0012 - conserved hypothetical protein

HVO_C0014 - conserved hypothetical protein (nonfunctional)

//

>NGHB#2955 27

CMPLX ---

HVO_C0016 - hypothetical protein

HVO_C0015 - conserved hypothetical protein

//

>NGHB#2957 2

CMPLX ---

HVO_C0017 parA1 ParA domain protein

HVO_C0018 - conserved hypothetical protein

//

>NGHB#2960 142

CMPLX ---

HVO_C0023 - hypothetical protein

HVO_C0022 - hypothetical protein

//

>NGHB#2961 203

CMPLX ---

HVO_C0025 - hypothetical protein

HVO_C0023 - hypothetical protein

//

>NGHB#2962 48

CMPLX ---

HVO_C0026 - hypothetical protein

HVO_C0025 - hypothetical protein

//

>NGHB#2963 4

CMPLX ---

HVO_C0028 - conserved hypothetical protein

HVO_C0029 - PIN domain protein

//

>NGHB#2964 25

CMPLX ---

HVO_C0029 - PIN domain protein

HVO_C0030 - hypothetical protein

//

>NGHB#2966 43

CMPLX ---

HVO_C0032 - hypothetical protein

HVO_C0031 - hypothetical protein

//

>NGHB#2968 61

CMPLX ---

HVO_C0033 - conserved hypothetical protein

HVO_C0034 - hypothetical protein

//

>NGHB#2969 36

CMPLX ---

HVO_C0034 - hypothetical protein

HVO_C0035 - conserved hypothetical protein

//

>NGHB#2970 -1

CMPLX ---

HVO_C0035 - conserved hypothetical protein

HVO_C0036 - conserved hypothetical protein

//

>NGHB#2971 444

CMPLX ---

HVO_C0036 - conserved hypothetical protein

HVO_C0037 - ISH3-type transposase ISHvo21

//

>NGHB#2974 380

CMPLX not

HVO_C0039 - ISH3-type transposase ISH51

HVO_C0040 - site-specific DNA-methyltransferase (cytosine-specific)

//

>NGHB#2975 203

CMPLX ---

HVO_C0040 - site-specific DNA-methyltransferase (cytosine-specific)

HVO_C0041 - conserved hypothetical protein

//

>NGHB#2976 104

CMPLX ---

HVO_C0041 - conserved hypothetical protein

HVO_C0042 - helicase domain protein

//

>NGHB#2977 94

CMPLX ---

HVO_C0042 - helicase domain protein

HVO_C0043 - conserved hypothetical protein

//

>NGHB#2978 26

CMPLX ---

HVO_C0043 - conserved hypothetical protein

HVO_C0044 - PLD domain protein

//

>NGHB#2979 144

CMPLX ---

HVO_C0044 - PLD domain protein

HVO_C0045 - conserved hypothetical protein

//

>NGHB#2981 50

CMPLX ---

HVO_C0047 - hypothetical protein

HVO_C0046 - conserved hypothetical protein

//

>NGHB#2983 52

CMPLX ---

HVO_C0048 - conserved hypothetical protein

HVO_C0049 - conserved hypothetical protein

//

>NGHB#2987 138

CMPLX ---

HVO_C0054_A - ISH14-type transposase HfIRS10 (nonfunctional)

HVO_C0054 - hypothetical protein

//

>NGHB#2988 694

CMPLX ---

HVO_C0055 - hypothetical protein

HVO_C0054_A - ISH14-type transposase HfIRS10 (nonfunctional)

//

>NGHB#2989 331

CMPLX ---

HVO_C0056 - conserved hypothetical protein

HVO_C0055 - hypothetical protein

//

>NGHB#2992 272

CMPLX ---

HVO_C0058 - ISH4-type transposase ISHvo6 (nonfunctional)

HVO_C0057A - conserved hypothetical protein (nonfunctional)

//

>NGHB#2994 0

CMPLX not

HVO_C0060 - CopG domain protein

HVO_C0061 - RelE family protein (nonfunctional)

//

>NGHB#2997 185

CMPLX not

HVO_C0064 - ISH4-type transposase ISHvo5

HVO_C0065 - ISH14-type transposase HfIRS9 (nonfunctional)

//

>NGHB#3001 74

CMPLX not

HVO_C0070 - ISH8-type transposase ISHvo13

HVO_C0069 - GFO family oxidoreductase

//

>NGHB#3002 262

CMPLX not

HVO_C0071 - ISH3-type transposase ISH51

HVO_C0070 - ISH8-type transposase ISHvo13

//

>NGHB#3003 158

CMPLX not

HVO_C0072 dppC16 ABC-type transport system permease protein (probable substrate dipeptide/oligopeptide)

HVO_C0071 - ISH3-type transposase ISH51

//

>NGHB#3004 -1

CMPLX yes

HVO_C0073 dppB16 ABC-type transport system permease protein (probable substrate dipeptide/oligopeptide)

HVO_C0072 dppC16 ABC-type transport system permease protein (probable substrate dipeptide/oligopeptide)

//

>NGHB#3007 386

CMPLX not

HVO_C0076 - IclR family transcription regulator

HVO_C0075 dppA16 ABC-type transport system periplasmic substrate-binding protein (probable substrate dipeptide/oligopeptide)

//

>NGHB#3010 7

CMPLX not

HVO_C0079 - ISH16-type transposase HfIRS4 (nonfunctional)

HVO_C0078 - ISHwa16-type transposase ISHvo16 (nonfunctional)

//

>NGHB#3011 10

CMPLX not

HVO_C0080 - ISH16-type transposase ISHvo8

HVO_C0079 - ISH16-type transposase HfIRS4 (nonfunctional)

//

>NGHB#3012 50

CMPLX ---

HVO_C0082 - hypothetical protein

HVO_C0081 - conserved hypothetical protein

//

>NGHB#3014 52

CMPLX ---

HVO_C0082_A - conserved hypothetical protein

HVO_C0083_A - conserved hypothetical protein

//

>NGHB#3016 52

CMPLX ---

HVO_C0085 - homolog to virus protein eHPD7-ORF00180

HVO_C0084 - hypothetical protein

//

>NGHB#3017 -4

CMPLX not

HVO_C0086 - small CPxCG-related zinc finger protein

HVO_C0085 - homolog to virus protein eHPD7-ORF00180

//

>NGHB#3018 -4

CMPLX ---

HVO_C0087 - hypothetical protein

HVO_C0086 - small CPxCG-related zinc finger protein

//

>NGHB#3019 8

CMPLX ---

HVO_C0088 - hypothetical protein

HVO_C0087 - hypothetical protein

//

>NGHB#3020 211

CMPLX ---

HVO_D0002 - hypothetical protein

HVO_D0003 - homolog to plasmid replication protein RepH

//

>NGHB#3021 193

CMPLX ---

HVO_D0003 - homolog to plasmid replication protein RepH

HVO_D0004 - conserved hypothetical protein

//

>NGHB#3024 -8

CMPLX ---

HVO_D0005 - HTH domain protein

HVO_D0006 - conserved hypothetical protein

//

>NGHB#3026 121

CMPLX not

HVO_B0001 orc6 Orc1-type DNA replication protein

HVO_B0002 - M20 family amidohydrolase (homolog to succinyl-diaminopimelate desuccinylase)

//

>NGHB#3028 234

CMPLX not

HVO_B0004 glyA2 serine hydroxymethyltransferase

HVO_B0003 - DUF336 family protein

//

>NGHB#3033 -8

CMPLX ---

HVO_B0009 - aromatic-ring-hydroxylating dioxygenase alpha subunit family protein

HVO_B0008 - conserved hypothetical protein

//

>NGHB#3035 290

CMPLX not

HVO_B0011 soxA1 sarcosine oxidase

HVO_B0012 opuD2, betL2 compatible solute transport protein (probable substrate choline/glycine betaine)

//

>NGHB#3036 36

CMPLX ---

HVO_B0012 opuD2, betL2 compatible solute transport protein (probable substrate choline/glycine betaine)

HVO_B0013 - conserved hypothetical protein

//

>NGHB#3037 213

CMPLX ---

HVO_B0013 - conserved hypothetical protein

HVO_B0014 dmg1 folate-binding FAD-dependent oxidoreductase (homolog to dimethylglycine oxidase)

//

>NGHB#3039 2

CMPLX ---

HVO_B0016 - conserved hypothetical protein

HVO_B0015 - PIN domain protein (nonfunctional)

//

>NGHB#3040 155

CMPLX ---

HVO_B0017 - conserved hypothetical protein

HVO_B0016 - conserved hypothetical protein

//

>NGHB#3041 -4

CMPLX ---

HVO_B0018 parA2 ParA domain protein

HVO_B0017 - conserved hypothetical protein

//

>NGHB#3043 158

CMPLX not

HVO_B0019 - probable sodium/anion symporter

HVO_B0020 - HTH-10 family transcription regulator

//

>NGHB#3044 117

CMPLX not

HVO_B0020 - HTH-10 family transcription regulator

HVO_B0021 dppA10 ABC-type transport system periplasmic substrate-binding protein (probable substrate dipeptide/oligopeptide)

//

>NGHB#3045 51

CMPLX yes

HVO_B0021 dppA10 ABC-type transport system periplasmic substrate-binding protein (probable substrate dipeptide/oligopeptide)

HVO_B0022 dppB10 ABC-type transport system permease protein (probable substrate dipeptide/oligopeptide)

//

>NGHB#3046 -4

CMPLX yes

HVO_B0022 dppB10 ABC-type transport system permease protein (probable substrate dipeptide/oligopeptide)

HVO_B0023 dppC10 ABC-type transport system permease protein (probable substrate dipeptide/oligopeptide)

//

>NGHB#3047 -4

CMPLX yes

HVO_B0023 dppC10 ABC-type transport system permease protein (probable substrate dipeptide/oligopeptide)

HVO_B0024 dppDF10 ABC-type transport system ATP-binding protein (probable substrate dipeptide/oligopeptide)

//

>NGHB#3050 785

CMPLX not

HVO_B0026 - probable oxidoreductase (short-chain dehydrogenase family)

HVO_B0027 xacE 2-keto-3-deoxy-D-xylonate dehydratase

//

>NGHB#3051 -4

CMPLX not

HVO_B0027 xacE 2-keto-3-deoxy-D-xylonate dehydratase

HVO_B0028 xacA, xdh xylose dehydrogenase (NADP)

//

>NGHB#3052 60

CMPLX not

HVO_B0028 xacA, xdh xylose dehydrogenase (NADP)

HVO_B0029 - probable sugar dehydrogenase (NADP)

//

>NGHB#3054 141

CMPLX not

HVO_B0031 - probable oxidoreductase (short-chain dehydrogenase family)

HVO_B0030 xacC D-xylonolactonase / L-arabinonolactonase

//

>NGHB#3056 256

CMPLX not

HVO_B0032 xacB L-arabinose 1-dehydrogenase (NAD(P))

HVO_B0034 tsgA7 ABC-type transport system periplasmic substrate-binding protein (probable substrate sugar)

//

>NGHB#3057 54

CMPLX yes

HVO_B0034 tsgA7 ABC-type transport system periplasmic substrate-binding protein (probable substrate sugar)

HVO_B0035 tsgB7 ABC-type transport system permease protein (probable substrate sugar)

//

>NGHB#3058 2

CMPLX yes

HVO_B0035 tsgB7 ABC-type transport system permease protein (probable substrate sugar)

HVO_B0036 tsgC7 ABC-type transport system permease protein (probable substrate sugar)

//

>NGHB#3059 3

CMPLX yes

HVO_B0036 tsgC7 ABC-type transport system permease protein (probable substrate sugar)

HVO_B0037 tsgE7 ABC-type transport system ATP-binding protein (probable substrate sugar)

//

>NGHB#3060 2

CMPLX yes

HVO_B0037 tsgE7 ABC-type transport system ATP-binding protein (probable substrate sugar)

HVO_B0038 tsgD7 ABC-type transport system ATP-binding protein (probable substrate sugar)

//

>NGHB#3064 658

CMPLX not

HVO_B0041 iucC siderophore biosynthesis protein IucC

HVO_B0040 xacR IclR family transcription regulator XacR

//

>NGHB#3065 8

CMPLX not

HVO_B0042 iucD probable 1,3-diaminopropane N-3-monooxygenase

HVO_B0041 iucC siderophore biosynthesis protein IucC

//

>NGHB#3066 -4

CMPLX not

HVO_B0043 iucB probable N4-hydroxy-1-aminopropane O-acetyltransferase

HVO_B0042 iucD probable 1,3-diaminopropane N-3-monooxygenase

//

>NGHB#3067 -4

CMPLX not

HVO_B0044 iucA siderophore biosynthesis protein IucA

HVO_B0043 iucB probable N4-hydroxy-1-aminopropane O-acetyltransferase

//

>NGHB#3068 -4

CMPLX not

HVO_B0045 bdb diaminobutyrate decarboxylase

HVO_B0044 iucA siderophore biosynthesis protein IucA

//

>NGHB#3069 -4

CMPLX not

HVO_B0046 dat diaminobutyrate--2-oxoglutarate aminotransferase

HVO_B0045 bdb diaminobutyrate decarboxylase

//

>NGHB#3070 457

CMPLX not

HVO_B0047 - ABC-type transport system periplasmic substrate-binding protein

HVO_B0046 dat diaminobutyrate--2-oxoglutarate aminotransferase

//

>NGHB#3071 88

CMPLX not

HVO_B0048 cbiE cobalt-precorrin-7 C5-methyltransferase

HVO_B0047 - ABC-type transport system periplasmic substrate-binding protein

//

>NGHB#3072 -8

CMPLX not

HVO_B0049 cbiC cobalt-precorrin-8 methylmutase

HVO_B0048 cbiE cobalt-precorrin-7 C5-methyltransferase

//

>NGHB#3073 -8

CMPLX not

HVO_B0050 cobN ATP-dependent cobaltochelatase subunit CobN

HVO_B0049 cbiC cobalt-precorrin-8 methylmutase

//

>NGHB#3076 4

CMPLX not

HVO_B0053 - DUF3209 family protein

HVO_B0052 - PQQ repeat protein

//

>NGHB#3077 2

CMPLX not

HVO_B0054 cbiX1 sirohydrochlorin cobaltochelatase

HVO_B0053 - DUF3209 family protein

//

>NGHB#3078 -4

CMPLX ---

HVO_B0055 - conserved hypothetical protein

HVO_B0054 cbiX1 sirohydrochlorin cobaltochelatase

//

>NGHB#3079 -4

CMPLX ---

HVO_B0056 - probable ferredoxin (4Fe-4S)

HVO_B0055 - conserved hypothetical protein

//

>NGHB#3080 1

CMPLX not

HVO_B0057 cbiH2, cobJ2 cobalt-factor-III C17-methyltransferase

HVO_B0056 - probable ferredoxin (4Fe-4S)

//

>NGHB#3081 3

CMPLX not

HVO_B0058 cbiH1, cobJ1 cobalt-factor-III C17-methyltransferase

HVO_B0057 cbiH2, cobJ2 cobalt-factor-III C17-methyltransferase

//

>NGHB#3082 -4

CMPLX not

HVO_B0059 cbiG cobalt-precorrin-5A hydrolase

HVO_B0058 cbiH1, cobJ1 cobalt-factor-III C17-methyltransferase

//

>NGHB#3083 -8

CMPLX not

HVO_B0060 cbiF cobalt-precorrin-4 C11-methyltransferase

HVO_B0059 cbiG cobalt-precorrin-5A hydrolase

//

>NGHB#3084 -4

CMPLX not

HVO_B0061 cbiL cobalt-factor-II C20-methyltransferase

HVO_B0060 cbiF cobalt-precorrin-4 C11-methyltransferase

//

>NGHB#3085 -4

CMPLX not

HVO_B0062 cbiT cobalt-precorrin-6B C15-methyltransferase (decarboxylating)

HVO_B0061 cbiL cobalt-factor-II C20-methyltransferase

//

>NGHB#3087 2

CMPLX yes

HVO_B0063 - CbtB family protein

HVO_B0064 - CbtA family protein

//

>NGHB#3088 -1

CMPLX not

HVO_B0064 - CbtA family protein

HVO_B0065 - thioredoxin domain protein

//

>NGHB#3089 124

CMPLX not

HVO_B0065 - thioredoxin domain protein

HVO_B0066 - Lrp/AsnC family transcription regulator

//

>NGHB#3091 127

CMPLX ---

HVO_B0068 - hypothetical protein

HVO_B0067 cat7 transport protein (probable substrate cationic amino acids)

//

>NGHB#3092 -4

CMPLX ---

HVO_B0069 gabD, aldH5 succinate-semialdehyde dehydrogenase

HVO_B0068 - hypothetical protein

//

>NGHB#3095 153

CMPLX not

HVO_B0072 - HTH-10 family transcription regulator

HVO_B0071 adh2 alcohol dehydrogenase (NADP)

//

>NGHB#3096 74

CMPLX not

HVO_B0073 - Lrp/AsnC family transcription regulator

HVO_B0072 - HTH-10 family transcription regulator

//

>NGHB#3097 212

CMPLX not

HVO_B0074 - DUF917 family protein

HVO_B0073 - Lrp/AsnC family transcription regulator

//

>NGHB#3098 -4

CMPLX not

HVO_B0075 - DUF917 family protein

HVO_B0074 - DUF917 family protein

//

>NGHB#3099 -1

CMPLX not

HVO_B0076 hyuA4 N-methylhydantoinase (ATP-hydrolyzing) A

HVO_B0075 - DUF917 family protein

//

>NGHB#3100 -8

CMPLX not

HVO_B0077 - DUF917 family protein

HVO_B0076 hyuA4 N-methylhydantoinase (ATP-hydrolyzing) A

//

>NGHB#3101 -4

CMPLX not

HVO_B0078 dppF11 ABC-type transport system ATP-binding protein (probable substrate dipeptide/oligopeptide)

HVO_B0077 - DUF917 family protein

//

>NGHB#3102 -8

CMPLX yes

HVO_B0079 dppD11 ABC-type transport system ATP-binding protein (probable substrate dipeptide/oligopeptide)

HVO_B0078 dppF11 ABC-type transport system ATP-binding protein (probable substrate dipeptide/oligopeptide)

//

>NGHB#3103 -4

CMPLX yes

HVO_B0080 dppC11 ABC-type transport system permease protein (probable substrate dipeptide/oligopeptide)

HVO_B0079 dppD11 ABC-type transport system ATP-binding protein (probable substrate dipeptide/oligopeptide)

//

>NGHB#3104 -4

CMPLX yes

HVO_B0081 dppB11 ABC-type transport system permease protein (probable substrate dipeptide/oligopeptide)

HVO_B0080 dppC11 ABC-type transport system permease protein (probable substrate dipeptide/oligopeptide)

//

>NGHB#3105 -4

CMPLX yes

HVO_B0082 dppA11 ABC-type transport system periplasmic substrate-binding protein (probable substrate dipeptide/oligopeptide)

HVO_B0081 dppB11 ABC-type transport system permease protein (probable substrate dipeptide/oligopeptide)

//

>NGHB#3108 175

CMPLX not

HVO_B0085 - glycoside hydrolase domain protein

HVO_B0084 - DUF336 family protein

//

>NGHB#3110 108

CMPLX not

HVO_B0086 - AlkP-core domain protein

HVO_B0087 - homolog to mandelate racemase / homolog to muconate lactonizing enzyme

//

>NGHB#3112 180

CMPLX ---

HVO_B0089 dppF12 ABC-type transport system ATP-binding protein (probable substrate dipeptide/oligopeptide)

HVO_B0088 - conserved hypothetical protein

//

>NGHB#3113 -4

CMPLX yes

HVO_B0090 dppD12 ABC-type transport system ATP-binding protein (probable substrate dipeptide/oligopeptide)

HVO_B0089 dppF12 ABC-type transport system ATP-binding protein (probable substrate dipeptide/oligopeptide)

//

>NGHB#3114 45

CMPLX yes

HVO_B0091 dppC12 ABC-type transport system permease protein (probable substrate dipeptide/oligopeptide)

HVO_B0090 dppD12 ABC-type transport system ATP-binding protein (probable substrate dipeptide/oligopeptide)

//

>NGHB#3115 2

CMPLX yes

HVO_B0092 dppB12 ABC-type transport system permease protein (probable substrate dipeptide/oligopeptide)

HVO_B0091 dppC12 ABC-type transport system permease protein (probable substrate dipeptide/oligopeptide)

//

>NGHB#3116 73

CMPLX yes

HVO_B0093 dppA12 ABC-type transport system periplasmic substrate-binding protein (probable substrate dipeptide/oligopeptide)

HVO_B0092 dppB12 ABC-type transport system permease protein (probable substrate dipeptide/oligopeptide)

//

>NGHB#3117 114

CMPLX ---

HVO_B0094 - conserved hypothetical protein

HVO_B0093 dppA12 ABC-type transport system periplasmic substrate-binding protein (probable substrate dipeptide/oligopeptide)

//

>NGHB#3121 643

CMPLX not

HVO_B0097 - dihydrodipicolinate synthase family protein

HVO_B0098 - SGNH-type esterase domain protein

//

>NGHB#3122 98

CMPLX not

HVO_B0098 - SGNH-type esterase domain protein

HVO_B0099 - auxin permease family transport protein

//

>NGHB#3123 140

CMPLX not

HVO_B0099 - auxin permease family transport protein

HVO_B0100 aldH3 alpha-ketoglutarate semialdehyde dehydrogenase

//

>NGHB#3126 100

CMPLX not

HVO_B0102 - glycoside hydrolase domain protein

HVO_B0103 - GFO family oxidoreductase

//

>NGHB#3129 124

CMPLX not

HVO_B0105 - amidohydrolase domain protein

HVO_B0106 tsgA8 ABC-type transport system periplasmic substrate-binding protein (probable substrate sugar)

//

>NGHB#3130 58

CMPLX yes

HVO_B0106 tsgA8 ABC-type transport system periplasmic substrate-binding protein (probable substrate sugar)

HVO_B0107 tsgB8 ABC-type transport system permease protein (probable substrate sugar)

//

>NGHB#3131 -1

CMPLX yes

HVO_B0107 tsgB8 ABC-type transport system permease protein (probable substrate sugar)

HVO_B0108 tsgC8 ABC-type transport system permease protein (probable substrate sugar)

//

>NGHB#3132 95

CMPLX yes

HVO_B0108 tsgC8 ABC-type transport system permease protein (probable substrate sugar)

HVO_B0109 tsgD8 ABC-type transport system ATP-binding protein (probable substrate sugar)

//

>NGHB#3133 168

CMPLX not

HVO_B0109 tsgD8 ABC-type transport system ATP-binding protein (probable substrate sugar)

HVO_B0110 - oxidoreductase (homolog to zinc-containing alcohol dehydrogenase)

//

>NGHB#3134 87

CMPLX not

HVO_B0110 - oxidoreductase (homolog to zinc-containing alcohol dehydrogenase)

HVO_B0111 - homolog to mandelate racemase / homolog to muconate lactonizing enzyme

//

>NGHB#3135 2

CMPLX not

HVO_B0111 - homolog to mandelate racemase / homolog to muconate lactonizing enzyme

HVO_B0113 - probable F420-dependent oxidoreductase

//

>NGHB#3136 215

CMPLX not

HVO_B0113 - probable F420-dependent oxidoreductase

HVO_B0114 - IclR family transcription regulator

//

>NGHB#3137 99

CMPLX not

HVO_B0114 - IclR family transcription regulator

HVO_B0115 - fumarylacetoacetase family protein

//

>NGHB#3138 51

CMPLX not

HVO_B0115 - fumarylacetoacetase family protein

HVO_B0116 - probable oxidoreductase (short-chain dehydrogenase family)

//

>NGHB#3139 791

CMPLX ---

HVO_B0116 - probable oxidoreductase (short-chain dehydrogenase family)

HVO_B0117 - conserved hypothetical protein

//

>NGHB#3140 90

CMPLX ---

HVO_B0117 - conserved hypothetical protein

HVO_B0118 sph2 Smc-like protein Sph2

//

>NGHB#3144 -8

CMPLX yes

HVO_B0123 dppB13 ABC-type transport system permease protein (probable substrate dipeptide/oligopeptide)

HVO_B0124 dppC13 ABC-type transport system permease protein (probable substrate dipeptide/oligopeptide)

//

>NGHB#3145 180

CMPLX yes

HVO_B0124 dppC13 ABC-type transport system permease protein (probable substrate dipeptide/oligopeptide)

HVO_B0125 dppD13 ABC-type transport system ATP-binding protein (probable substrate dipeptide/oligopeptide)

//

>NGHB#3147 99

CMPLX not

HVO_B0127 - oxidoreductase (luciferase family protein)

HVO_B0126 - probable polysaccharide deacetylase

//

>NGHB#3151 560

CMPLX not

HVO_B0130 - DUF296 family protein

HVO_B0131 - HTH domain protein

//

>NGHB#3152 110

CMPLX not

HVO_B0131 - HTH domain protein

HVO_B0132 - PQQ repeat protein

//

>NGHB#3154 -4

CMPLX ---

HVO_B0134 - ABC-type transport system ATP-binding protein

HVO_B0133 - conserved hypothetical protein

//

>NGHB#3155 86

CMPLX ---

HVO_B0135 - conserved hypothetical protein

HVO_B0134 - ABC-type transport system ATP-binding protein

//

>NGHB#3156 154

CMPLX ---

HVO_B0136 - alpha/beta hydrolase fold protein

HVO_B0135 - conserved hypothetical protein

//

>NGHB#3159 -4

CMPLX not

HVO_B0139 - PQQ repeat protein

HVO_B0138 - PQQ repeat protein

//

>NGHB#3161 -11

CMPLX not

HVO_B0140 - PQQ repeat protein

HVO_B0141 - PQQ repeat protein

//

>NGHB#3162 93

CMPLX not

HVO_B0141 - PQQ repeat protein

HVO_B0142 - ARM/HEAT repeat protein

//

>NGHB#3166 210

CMPLX not

HVO_B0146 - aminotransferase class V

HVO_B0145 cre4 creatininase domain protein

//

>NGHB#3168 168

CMPLX not

HVO_B0148 - HTH-10 family transcription regulator

HVO_B0149 ohyA oleate hydratase

//

>NGHB#3170 41

CMPLX not

HVO_B0150A - ARM/HEAT repeat protein (nonfunctional)

HVO_B0150 - ABC-type transport system periplasmic substrate-binding protein

//

>NGHB#3173 -4

CMPLX not

HVO_B0153_A - CapC domain protein

HVO_B0153 - CapB domain protein

//

>NGHB#3174 53

CMPLX not

HVO_B0153B - receiver box response regulator

HVO_B0153_A - CapC domain protein

//

>NGHB#3179 1248

CMPLX not

HVO_B0158 - small CPxCG-related zinc finger protein

HVO_B0157 - ISH5-type transposase ISHvo11

//

>NGHB#3180 82

CMPLX not

HVO_B0159 narO, boa9 HTH-10 family transcription regulator NarO

HVO_B0158 - small CPxCG-related zinc finger protein

//

>NGHB#3182 -1

CMPLX ---

HVO_B0160 - conserved hypothetical protein

HVO_B0161 narB1 respiratory nitrate reductase iron-sulfur subunit

//

>NGHB#3183 -1

CMPLX yes

HVO_B0161 narB1 respiratory nitrate reductase iron-sulfur subunit

HVO_B0162 narC1 respiratory nitrate reductase b-type cytochrome subunit

//

>NGHB#3184 -8

CMPLX ---

HVO_B0162 narC1 respiratory nitrate reductase b-type cytochrome subunit

HVO_B0163 - conserved hypothetical protein

//

>NGHB#3185 -4

CMPLX ---

HVO_B0163 - conserved hypothetical protein

HVO_B0164 narG respiratory nitrate reductase catalytic subunit

//

>NGHB#3186 -4

CMPLX yes

HVO_B0164 narG respiratory nitrate reductase catalytic subunit

HVO_B0165 narH respiratory nitrate reductase electron transfer subunit

//

>NGHB#3187 57

CMPLX yes

HVO_B0165 narH respiratory nitrate reductase electron transfer subunit

HVO_B0166 - respiratory nitrate reductase heme b subunit

//

>NGHB#3188 10

CMPLX not

HVO_B0166 - respiratory nitrate reductase heme b subunit

HVO_B0167 narJ chaperonin-like protein

//

>NGHB#3189 -4

CMPLX ---

HVO_B0167 narJ chaperonin-like protein

HVO_B0168 - conserved hypothetical protein

//

>NGHB#3190 -4

CMPLX ---

HVO_B0168 - conserved hypothetical protein

HVO_B0169 - HEAT-PBS family protein

//

>NGHB#3191 2

CMPLX not

HVO_B0169 - HEAT-PBS family protein

HVO_B0170 apbC2 Fe-S cluster carrier protein ApbC

//

>NGHB#3192 276

CMPLX ---

HVO_B0170 apbC2 Fe-S cluster carrier protein ApbC

HVO_B0171 - conserved hypothetical protein

//

>NGHB#3194 972

CMPLX not

HVO_B0173 sph4 Smc-like protein Sph4

HVO_B0172 - transport protein (probable substrate phosphate/sulfate) (nonfunctional)

//

>NGHB#3195 360

CMPLX ---

HVO_B0174 - conserved hypothetical protein

HVO_B0173 sph4 Smc-like protein Sph4

//

>NGHB#3196 120

CMPLX ---

HVO_B0175 - histidine kinase

HVO_B0174 - conserved hypothetical protein

//

>NGHB#3197 236

CMPLX not

HVO_B0176 - ABC-type transport system permease protein

HVO_B0175 - histidine kinase

//

>NGHB#3198 -4

CMPLX yes

HVO_B0177 - ABC-type transport system ATP-binding protein

HVO_B0176 - ABC-type transport system permease protein

//

>NGHB#3199 19

CMPLX ---

HVO_B0178 - conserved hypothetical protein

HVO_B0177 - ABC-type transport system ATP-binding protein

//

>NGHB#3200 282

CMPLX ---

HVO_B0179 - YfiH family protein

HVO_B0178 - conserved hypothetical protein

//

>NGHB#3201 -8

CMPLX not

HVO_B0180 - ComB family protein

HVO_B0179 - YfiH family protein

//

>NGHB#3203 97

CMPLX not

HVO_B0181 - DoxX domain protein

HVO_B0182 dhs2 deoxyhypusine synthase

//

>NGHB#3204 61

CMPLX not

HVO_B0182 dhs2 deoxyhypusine synthase

HVO_B0183 - probable amino acid racemase (homolog to homoserine racemase)

//

>NGHB#3205 284

CMPLX not

HVO_B0183 - probable amino acid racemase (homolog to homoserine racemase)

HVO_B0184 dppA14 ABC-type transport system periplasmic substrate-binding protein (probable substrate dipeptide/oligopeptide)

//

>NGHB#3206 82

CMPLX yes

HVO_B0184 dppA14 ABC-type transport system periplasmic substrate-binding protein (probable substrate dipeptide/oligopeptide)

HVO_B0185 dppB14 ABC-type transport system permease protein (probable substrate dipeptide/oligopeptide)

//

>NGHB#3207 -4

CMPLX yes

HVO_B0185 dppB14 ABC-type transport system permease protein (probable substrate dipeptide/oligopeptide)

HVO_B0186 dppC14 ABC-type transport system permease protein (probable substrate dipeptide/oligopeptide)

//

>NGHB#3208 373

CMPLX yes

HVO_B0186 dppC14 ABC-type transport system permease protein (probable substrate dipeptide/oligopeptide)

HVO_B0187 - ABC-type transport system periplasmic substrate-binding protein

//

>NGHB#3209 2

CMPLX yes

HVO_B0187 - ABC-type transport system periplasmic substrate-binding protein

HVO_B0188 - ABC-type transport system permease protein

//

>NGHB#3210 -4

CMPLX yes

HVO_B0188 - ABC-type transport system permease protein

HVO_B0189 - ABC-type transport system permease protein

//

>NGHB#3211 -1

CMPLX yes

HVO_B0189 - ABC-type transport system permease protein

HVO_B0190 - ABC-type transport system ATP-binding protein

//

>NGHB#3215 1

CMPLX ---

HVO_B0193 - ArsR family transcription regulator

HVO_B0192 - conserved hypothetical protein

//

>NGHB#3216 352

CMPLX not

HVO_B0194 - LppX domain protein

HVO_B0193 - ArsR family transcription regulator

//

>NGHB#3217 94

CMPLX ---

HVO_B0195 - hypothetical protein

HVO_B0194 - LppX domain protein

//

>NGHB#3220 6

CMPLX yes

HVO_B0198 - ABC-type transport system periplasmic substrate-binding protein

HVO_B0197 - ABC-type transport system permease protein

//

>NGHB#3222 863

CMPLX not

HVO_B0199 - deaminase domain protein

HVO_B0200 aceB2 bifunctional malyl-CoA synthase / malyl-CoA thioesterase

//

>NGHB#3224 182

CMPLX not

HVO_B0202 - sensor box protein

HVO_B0201 - IclR family transcription regulator

//

>NGHB#3226 199

CMPLX ---

HVO_B0203 - FAD-dependent oxidoreductase (GlcD/DLD_GlcF/GlpC domain fusion protein)

HVO_B0204 - conserved hypothetical protein

//

>NGHB#3228 135

CMPLX ---

HVO_B0206 - conserved hypothetical protein

HVO_B0205 lccA laccase

//

>NGHB#3231 95

CMPLX not

HVO_B0209 - UbiA prenyltransferase family protein

HVO_B0208 - YfiH family protein

//

>NGHB#3232 45

CMPLX not

HVO_B0210 - YfiH family protein

HVO_B0209 - UbiA prenyltransferase family protein

//

>NGHB#3233 3

CMPLX not

HVO_B0211 - TatD domain protein

HVO_B0210 - YfiH family protein

//

>NGHB#3235 81

CMPLX not

HVO_B0212 - small CPxCG-related zinc finger protein

HVO_B0213 - myo-inositol-1-phosphate synthase

//

>NGHB#3236 -4

CMPLX not

HVO_B0213 - myo-inositol-1-phosphate synthase

HVO_B0214 - E-NPP family protein

//

>NGHB#3239 207

CMPLX not

HVO_B0216 - UPF0261 family protein

HVO_B0217 livJ6 ABC-type transport system periplasmic substrate-binding protein (probable substrate branched-chain amino acids)

//

>NGHB#3240 282

CMPLX yes

HVO_B0217 livJ6 ABC-type transport system periplasmic substrate-binding protein (probable substrate branched-chain amino acids)

HVO_B0218 livH6 ABC-type transport system permease protein (probable substrate branched-chain amino acids)

//

>NGHB#3241 -4

CMPLX yes

HVO_B0218 livH6 ABC-type transport system permease protein (probable substrate branched-chain amino acids)

HVO_B0219 livM6 ABC-type transport system permease protein (probable substrate branched-chain amino acids)

//

>NGHB#3242 -1

CMPLX yes

HVO_B0219 livM6 ABC-type transport system permease protein (probable substrate branched-chain amino acids)

HVO_B0220 livG6 ABC-type transport system ATP-binding protein (probable substrate branched-chain amino acids)

//

>NGHB#3243 -4

CMPLX yes

HVO_B0220 livG6 ABC-type transport system ATP-binding protein (probable substrate branched-chain amino acids)

HVO_B0221 livF6 ABC-type transport system ATP-binding protein (probable substrate branched-chain amino acids)

//

>NGHB#3244 189

CMPLX not

HVO_B0221 livF6 ABC-type transport system ATP-binding protein (probable substrate branched-chain amino acids)

HVO_B0222 - PQQ repeat protein

//

>NGHB#3246 120

CMPLX ---

HVO_B0224 - cupin 2 barrel domain protein

HVO_B0223 - conserved hypothetical protein

//

>NGHB#3249 1486

CMPLX not

HVO_B0227 tsgD9 ABC-type transport system ATP-binding protein (probable substrate sugar)

HVO_B0226 - auxin permease family transport protein

//

>NGHB#3251 6

CMPLX yes

HVO_B0228 tsgA9 ABC-type transport system periplasmic substrate-binding protein (probable substrate sugar)

HVO_B0229 tsgB9 ABC-type transport system permease protein (probable substrate sugar)

//

>NGHB#3252 -8

CMPLX yes

HVO_B0229 tsgB9 ABC-type transport system permease protein (probable substrate sugar)

HVO_B0230 tsgC9 ABC-type transport system permease protein (probable substrate sugar)

//

>NGHB#3253 -4

CMPLX ---

HVO_B0230 tsgC9 ABC-type transport system permease protein (probable substrate sugar)

HVO_B0231 - conserved hypothetical protein

//

>NGHB#3255 78

CMPLX not

HVO_B0233 xsa alpha-L-arabinofuranosidase

HVO_B0232 - neuraminidase family protein

//

>NGHB#3257 770

CMPLX not

HVO_B0234 - ISH3-type transposase ISH51

HVO_B0234A - small CPxCG-related zinc finger protein

//

>NGHB#3259 612

CMPLX ---

HVO_B0235 - probable anaerobic dehydrogenase alpha subunit

HVO_B0234B - conserved hypothetical protein

//

>NGHB#3260 -4

CMPLX not

HVO_B0236 - homolog to NADH dehydrogenase 51K subunit

HVO_B0235 - probable anaerobic dehydrogenase alpha subunit

//

>NGHB#3264 80

CMPLX ---

HVO_B0239 fdh formaldehyde dehydrogenase

HVO_B0240 - conserved hypothetical protein

//

>NGHB#3266 -4

CMPLX not

HVO_B0242 - IS200-type transposase ISHvo18

HVO_B0241 - IS1341-type transposase ISHvo18

//

>NGHB#3267 142

CMPLX not

HVO_B0243 cobW CobW domain protein

HVO_B0242 - IS200-type transposase ISHvo18

//

>NGHB#3271 821

CMPLX ---

HVO_B0251 - major facilitator superfamily transport protein

HVO_B0252 - hypothetical protein

//

>NGHB#3272 308

CMPLX ---

HVO_B0252 - hypothetical protein

HVO_B0253 suhB3 probable inositol-1(or 4)-monophosphatase / fructose-1,6-bisphosphatase, archaeal-type

//

>NGHB#3273 689

CMPLX not

HVO_B0253 suhB3 probable inositol-1(or 4)-monophosphatase / fructose-1,6-bisphosphatase, archaeal-type

HVO_B0255 - IclR family transcription regulator

//

>NGHB#3276 18

CMPLX not

HVO_B0257 - pyridoxal phosphate-dependent aminotransferase

HVO_B0258 ridA3 enamine/imine deaminase

//

>NGHB#3279 172

CMPLX not

HVO_B0260 opuD1, betL1 compatible solute transport protein (probable substrate choline/glycine betaine)

HVO_B0261 - pyridoxal phosphate-dependent aminotransferase

//

>NGHB#3281 -4

CMPLX not

HVO_B0263 - M20 family amidohydrolase (homolog to indole-3-acetyl-aspartate hydrolase)

HVO_B0262 ilvA3 threonine ammonia-lyase

//

>NGHB#3283 6

CMPLX not

HVO_B0264 serA4 probable D-2-hydroxyacid dehydrogenase

HVO_B0265 - FAD-dependent oxidoreductase (GlcD/DLD_GlcF/GlpC domain fusion protein)

//

>NGHB#3285 26

CMPLX not

HVO_B0267 - peptidase M24 family protein (homolog to ectoine hydrolase)

HVO_B0266 gdhA3, gdhB glutamate dehydrogenase (NAD)

//

>NGHB#3287 65

CMPLX ---

HVO_B0268 - monooxygenase (homolog to alkanesulfonate monooxygenase)

HVO_B0269 - conserved hypothetical protein

//

>NGHB#3290 57

CMPLX ---

HVO_B0273 - sensor box histidine kinase

HVO_B0274 - conserved hypothetical protein

//

>NGHB#3291 -4

CMPLX ---

HVO_B0274 - conserved hypothetical protein

HVO_B0275 - conserved hypothetical protein

//

>NGHB#3295 67

CMPLX ---

HVO_B0279 - probable S-adenosylmethionine-dependent methyltransferase

HVO_B0278 - conserved hypothetical protein

//

>NGHB#3296 344

CMPLX ---

HVO_B0280 - conserved hypothetical protein

HVO_B0279 - probable S-adenosylmethionine-dependent methyltransferase

//

>NGHB#3298 11

CMPLX ---

HVO_B0281 - hypothetical protein

HVO_B0282 - probable transmembrane glycoprotein / HTH domain protein

//

>NGHB#3299 113

CMPLX not

HVO_B0282 - probable transmembrane glycoprotein / HTH domain protein

HVO_B0283 - HTH-10 family transcription regulator

//

>NGHB#3300 99

CMPLX not

HVO_B0283 - HTH-10 family transcription regulator

HVO_B0284 tif1b translation initiation factor aIF-1 (SUI1 protein, bacterial-type IF3)

//

>NGHB#3301 170

CMPLX not

HVO_B0284 tif1b translation initiation factor aIF-1 (SUI1 protein, bacterial-type IF3)

HVO_B0285 tfb3 transcription initiation factor TFB

//

>NGHB#3303 93

CMPLX ---

HVO_B0287 - TRAM domain protein

HVO_B0286 - conserved hypothetical protein

//

>NGHB#3305 141

CMPLX ---

HVO_B0288 - conserved hypothetical protein

HVO_B0289 bioB biotin synthase

//

>NGHB#3308 143

CMPLX not

HVO_B0291 glpQ2, ugpQ2 glycerophosphodiester phosphodiesterase

HVO_B0292 ugpB ABC-type transport system periplasmic substrate-binding protein (probable substrate glycerol-3-phosphate)

//

>NGHB#3309 22

CMPLX yes

HVO_B0292 ugpB ABC-type transport system periplasmic substrate-binding protein (probable substrate glycerol-3-phosphate)

HVO_B0293 ugpA ABC-type transport system permease protein (probable substrate glycerol-3-phosphate)

//

>NGHB#3310 4

CMPLX yes

HVO_B0293 ugpA ABC-type transport system permease protein (probable substrate glycerol-3-phosphate)

HVO_B0294 ugpE ABC-type transport system permease protein (probable substrate glycerol-3-phosphate)

//

>NGHB#3311 10

CMPLX yes

HVO_B0294 ugpE ABC-type transport system permease protein (probable substrate glycerol-3-phosphate)

HVO_B0295 ugpC ABC-type transport system ATP-binding protein (probable substrate glycerol-3-phosphate)

//

>NGHB#3313 111

CMPLX ---

HVO_B0297 - conserved hypothetical protein

HVO_B0296 - conserved hypothetical protein

//

>NGHB#3314 601

CMPLX ---

HVO_B0299 pucM 5-hydroxyisourate hydrolase

HVO_B0297 - conserved hypothetical protein

//

>NGHB#3315 1

CMPLX not

HVO_B0300 pucL1 uricase

HVO_B0299 pucM 5-hydroxyisourate hydrolase

//

>NGHB#3316 157

CMPLX not

HVO_B0301 pucL2 2-oxo-4-hydroxy-4-carboxy-5-ureidoimidazoline decarboxylase

HVO_B0300 pucL1 uricase

//

>NGHB#3317 83

CMPLX not

HVO_B0302 pucH1 probable allantoinase

HVO_B0301 pucL2 2-oxo-4-hydroxy-4-carboxy-5-ureidoimidazoline decarboxylase

//

>NGHB#3318 86

CMPLX not

HVO_B0303 uraA4 xanthine/uracil permease family transport protein

HVO_B0302 pucH1 probable allantoinase

//

>NGHB#3319 163

CMPLX ---

HVO_B0304 - hypothetical protein

HVO_B0303 uraA4 xanthine/uracil permease family transport protein

//

>NGHB#3320 64

CMPLX ---

HVO_B0305 - conserved hypothetical protein

HVO_B0304 - hypothetical protein

//

>NGHB#3321 26

CMPLX ---

HVO_B0306 amaB4 amidase (hydantoinase/carbamoylase family)

HVO_B0305 - conserved hypothetical protein

//

>NGHB#3323 704

CMPLX ---

HVO_B0307 - hypothetical protein

HVO_B0308 coxS molybdopterin-containing oxidoreductase small subunit

//

>NGHB#3324 1

CMPLX yes

HVO_B0308 coxS molybdopterin-containing oxidoreductase small subunit

HVO_B0309 coxL molybdopterin-containing oxidoreductase large subunit

//

>NGHB#3325 -4

CMPLX yes

HVO_B0309 coxL molybdopterin-containing oxidoreductase large subunit

HVO_B0310 coxM molybdopterin-containing oxidoreductase medium subunit

//

>NGHB#3326 115

CMPLX not

HVO_B0310 coxM molybdopterin-containing oxidoreductase medium subunit

HVO_B0311 - XdhC family protein (nonfunctional)

//

>NGHB#3327 -4

CMPLX not

HVO_B0311 - XdhC family protein (nonfunctional)

HVO_B0313 mobA3 molybdenum cofactor nucleotidyltransferase domain protein

//

>NGHB#3329 -4

CMPLX yes

HVO_B0315 tsgB13 ABC-type transport system permease protein (substrate glucose)

HVO_B0314 tsgC13 ABC-type transport system permease protein (substrate glucose)

//

>NGHB#3330 -4

CMPLX yes

HVO_B0316 tsgD13 ABC-type transport system ATP-binding protein (substrate glucose)

HVO_B0315 tsgB13 ABC-type transport system permease protein (substrate glucose)

//

>NGHB#3332 169

CMPLX not

HVO_B0317 adeC adenine deaminase

HVO_B0318 tsgA13 ABC-type transport system periplasmic substrate-binding protein (substrate glucose)

//

>NGHB#3333 333

CMPLX not

HVO_B0318 tsgA13 ABC-type transport system periplasmic substrate-binding protein (substrate glucose)

HVO_B0319 - IclR family transcription regulator

//

>NGHB#3335 103

CMPLX not

HVO_B0321 - GFO family oxidoreductase

HVO_B0320 - IclR family transcription regulator

//

>NGHB#3336 99

CMPLX not

HVO_B0322 - pectate lyase domain protein

HVO_B0321 - GFO family oxidoreductase

//

>NGHB#3338 51

CMPLX ---

HVO_B0323 - oxidoreductase domain protein

HVO_B0324 - conserved hypothetical protein

//

>NGHB#3340 6

CMPLX ---

HVO_B0326 - conserved hypothetical protein

HVO_B0325 - glycoside hydrolase domain protein

//

>NGHB#3341 490

CMPLX ---

HVO_B0327 - conserved hypothetical protein

HVO_B0326 - conserved hypothetical protein

//

>NGHB#3343 207

CMPLX yes

HVO_B0328 dppA15 ABC-type transport system periplasmic substrate-binding protein (probable substrate dipeptide/oligopeptide)

HVO_B0329 dppBC15 ABC-type transport system permease protein (probable substrate dipeptide/oligopeptide)

//

>NGHB#3346 -4

CMPLX yes

HVO_B0331 dppD15 ABC-type transport system ATP-binding protein (probable substrate dipeptide/oligopeptide)

HVO_B0332 dppF15 ABC-type transport system ATP-binding protein (probable substrate dipeptide/oligopeptide)

//

>NGHB#3348 78

CMPLX not

HVO_B0334 - GFO family oxidoreductase

HVO_B0333 - GFO family oxidoreductase

//

>NGHB#3349 132

CMPLX not

HVO_B0335 - major facilitator superfamily transport protein

HVO_B0334 - GFO family oxidoreductase

//

>NGHB#3350 90

CMPLX not

HVO_B0336 - probable hydro-lyase (homolog to altronate dehydratase) (nonfunctional)

HVO_B0335 - major facilitator superfamily transport protein

//

>NGHB#3351 -4

CMPLX not

HVO_B0337 - SAF domain protein

HVO_B0336 - probable hydro-lyase (homolog to altronate dehydratase) (nonfunctional)

//

>NGHB#3352 -4

CMPLX not

HVO_B0338 - probable hydro-lyase (homolog to altronate dehydratase)

HVO_B0337 - SAF domain protein

//

>NGHB#3354 93

CMPLX ---

HVO_B0339 - conserved hypothetical protein

HVO_B0340 - conserved hypothetical protein

//

>NGHB#3355 60

CMPLX ---

HVO_B0340 - conserved hypothetical protein

HVO_B0341 - conserved hypothetical protein

//

>NGHB#3357 82

CMPLX not

HVO_B0343 lplD alpha-galacturonidase

HVO_B0342 mer2 oxidoreductase (luciferase family protein)

//

>NGHB#3358 63

CMPLX not

HVO_B0344 - UspA domain protein

HVO_B0343 lplD alpha-galacturonidase

//

>NGHB#3359 108

CMPLX not

HVO_B0345 - homolog to mandelate racemase / homolog to muconate lactonizing enzyme

HVO_B0344 - UspA domain protein

//

>NGHB#3360 98

CMPLX not

HVO_B0346 - homolog to mandelate racemase / homolog to muconate lactonizing enzyme

HVO_B0345 - homolog to mandelate racemase / homolog to muconate lactonizing enzyme

//

>NGHB#3361 92

CMPLX not

HVO_B0347 - GFO family oxidoreductase

HVO_B0346 - homolog to mandelate racemase / homolog to muconate lactonizing enzyme

//

>NGHB#3363 214

CMPLX ---

HVO_B0348 - dihydrodipicolinate synthase family protein

HVO_B0349 - conserved hypothetical protein

//

>NGHB#3364 492

CMPLX ---

HVO_B0349 - conserved hypothetical protein

HVO_B0350 - ThuA family protein

//

>NGHB#3367 42

CMPLX not

HVO_B0352 - cupin 2 barrel domain protein

HVO_B0353 - probable carbon-nitrogen hydrolase

//

>NGHB#3369 219

CMPLX ---

HVO_B0355 - conserved hypothetical protein

HVO_B0354 - conserved hypothetical protein

//

>NGHB#3370 16

CMPLX ---

HVO_B0356 - DUF2617 family protein

HVO_B0355 - conserved hypothetical protein

//

>NGHB#3371 6

CMPLX not

HVO_B0357 - polyamine aminopropyltransferase

HVO_B0356 - DUF2617 family protein

//

>NGHB#3372 69

CMPLX ---

HVO_B0358 - conserved hypothetical protein

HVO_B0357 - polyamine aminopropyltransferase

//

>NGHB#3373 177

CMPLX ---

HVO_B0359 - YidE family protein

HVO_B0358 - conserved hypothetical protein

//

>NGHB#3374 248

CMPLX ---

HVO_B0360 - conserved hypothetical protein

HVO_B0359 - YidE family protein

//

>NGHB#3376 132

CMPLX ---

HVO_B0361 dmsR HTH-10 family transcription regulator DmsR

HVO_B0362 - conserved hypothetical protein

//

>NGHB#3377 -8

CMPLX ---

HVO_B0362 - conserved hypothetical protein

HVO_B0363 dmsA dimethylsulfoxide reductase subunit A

//

>NGHB#3378 2

CMPLX yes

HVO_B0363 dmsA dimethylsulfoxide reductase subunit A

HVO_B0364 dmsB dimethylsulfoxide reductase subunit B

//

>NGHB#3379 -1

CMPLX yes

HVO_B0364 dmsB dimethylsulfoxide reductase subunit B

HVO_B0365 dmsC dimethylsulfoxide reductase subunit C

//

>NGHB#3380 -4

CMPLX not

HVO_B0365 dmsC dimethylsulfoxide reductase subunit C

HVO_B0366 dmsD Tat proofreading chaperone DmsD

//

>NGHB#3381 151

CMPLX not

HVO_B0366 dmsD Tat proofreading chaperone DmsD

HVO_B0367 - molybdopterin-binding domain protein

//

>NGHB#3382 4

CMPLX not

HVO_B0367 - molybdopterin-binding domain protein

HVO_B0368 mobA2 molybdenum cofactor guanylyltransferase

//

>NGHB#3383 -4

CMPLX not

HVO_B0368 mobA2 molybdenum cofactor guanylyltransferase

HVO_B0369 - ABC-type transport system periplasmic substrate-binding protein (probable substrate molybdate)

//

>NGHB#3384 -20

CMPLX yes

HVO_B0369 - ABC-type transport system periplasmic substrate-binding protein (probable substrate molybdate)

HVO_B0370 - ABC-type transport system permease protein (probable substrate molybdate)

//

>NGHB#3386 181

CMPLX ---

HVO_B0372 - conserved hypothetical protein

HVO_B0374 - conserved hypothetical protein

//

>NGHB#3387 236

CMPLX ---

HVO_B0374 - conserved hypothetical protein

HVO_B0375 - Spo0M family protein

//

>NGHB#3388 81

CMPLX not

HVO_B0375 - Spo0M family protein

HVO_B0376 - probable oxidoreductase (aldo-keto reductase family protein)

//

>NGHB#3392 -7

CMPLX not

HVO_B0380 tenA1 aminopyrimidine aminohydrolase

HVO_B0379 - SSSF family transport protein

//

>NGHB#3393 -4

CMPLX not

HVO_B0381 tenA2 aminopyrimidine aminohydrolase

HVO_B0380 tenA1 aminopyrimidine aminohydrolase

//

>NGHB#3396 -8

CMPLX ---

HVO_A0003 parA3 ParA domain protein

HVO_A0002 - conserved hypothetical protein

//

>NGHB#3397 721

CMPLX not

HVO_A0004 msrA2 peptide methionine sulfoxide reductase MsrA (S-form specific) (nonfunctional)

HVO_A0003 parA3 ParA domain protein

//

>NGHB#3399 182

CMPLX not

HVO_A0005 - integrase family protein

HVO_A0006 - adenine specific DNA methyltransferase (nonfunctional)

//

>NGHB#3400 79

CMPLX not

HVO_A0006 - adenine specific DNA methyltransferase (nonfunctional)

HVO_A0007 - ISH5-type transposase ISHvo11

//

>NGHB#3401 16

CMPLX ---

HVO_A0007 - ISH5-type transposase ISHvo11

HVO_A0007_A - conserved hypothetical protein (nonfunctional)

//

>NGHB#3403 -4

CMPLX not

HVO_A0011 - ArsR family transcription regulator

HVO_A0010 - DUF2204 family protein

//

>NGHB#3404 351

CMPLX ---

HVO_A0012 - hypothetical protein

HVO_A0011 - ArsR family transcription regulator

//

>NGHB#3406 661

CMPLX ---

HVO_A0013 - conserved hypothetical protein

HVO_A0014 - ISH18-type transposase ISHvo9

//

>NGHB#3408 434

CMPLX ---

HVO_A0016 - ISH18-type transposase ISHvo10

HVO_A0015 - hypothetical protein

//

>NGHB#3410 84

CMPLX ---

HVO_A0017 - conserved hypothetical protein

HVO_A0018 - IS1341-type transposase HfIRS14

//

>NGHB#3411 870

CMPLX ---

HVO_A0018 - IS1341-type transposase HfIRS14

HVO_A0019 - conserved hypothetical protein

//

>NGHB#3415 122

CMPLX ---

HVO_A0022 - hypothetical protein

HVO_A0021 - conserved hypothetical protein

//

>NGHB#3416 76

CMPLX ---

HVO_A0023 - conserved hypothetical protein

HVO_A0022 - hypothetical protein

//

>NGHB#3418 121

CMPLX ---

HVO_A0024 - hypothetical protein

HVO_A0026 tfb9 transcription initiation factor TFB

//

>NGHB#3420 17

CMPLX ---

HVO_A0027 - HTH domain protein

HVO_A0025 - conserved hypothetical protein

//

>NGHB#3422 138

CMPLX ---

HVO_A0028 - conserved hypothetical protein

HVO_A0029 - conserved hypothetical protein

//

>NGHB#3423 91

CMPLX ---

HVO_A0029 - conserved hypothetical protein

HVO_A0030 - conserved hypothetical protein

//

>NGHB#3424 69

CMPLX ---

HVO_A0030 - conserved hypothetical protein

HVO_A0031 - conserved hypothetical protein

//

>NGHB#3425 74

CMPLX ---

HVO_A0031 - conserved hypothetical protein

HVO_A0032 - conserved hypothetical protein

//

>NGHB#3426 106

CMPLX ---

HVO_A0032 - conserved hypothetical protein

HVO_A0033 - conserved hypothetical protein

//

>NGHB#3427 3

CMPLX ---

HVO_A0033 - conserved hypothetical protein

HVO_A0034 - small CPxCG-related zinc finger protein

//

>NGHB#3429 193

CMPLX ---

HVO_A0035_A - conserved hypothetical protein

HVO_A0035 cetZ4, ftsZ6 FtsZ family protein CetZ, type III

//

>NGHB#3431 2

CMPLX ---

HVO_A0036 - ArsR family transcription regulator

HVO_A0037 - conserved hypothetical protein

//

>NGHB#3435 46

CMPLX ---

HVO_A0041 - hypothetical protein

HVO_A0040 - small CPxCG-related zinc finger protein

//

>NGHB#3436 207

CMPLX ---

HVO_A0042 - hypothetical protein

HVO_A0041 - hypothetical protein

//

>NGHB#3437 457

CMPLX ---

HVO_A0043 - UspA domain protein

HVO_A0042 - hypothetical protein

//

>NGHB#3438 290

CMPLX ---

HVO_A0044 - conserved hypothetical protein

HVO_A0043 - UspA domain protein

//

>NGHB#3439 0

CMPLX ---

HVO_A0045 htpX3 HtpX-like protease

HVO_A0044 - conserved hypothetical protein

//

>NGHB#3440 226

CMPLX ---

HVO_A0046 - conserved hypothetical protein

HVO_A0045 htpX3 HtpX-like protease

//

>NGHB#3444 67

CMPLX not

HVO_A0049 - DUF21/CBS domain protein

HVO_A0050 - small CPxCG-related zinc finger protein

//

>NGHB#3445 79

CMPLX ---

HVO_A0050 - small CPxCG-related zinc finger protein

HVO_A0051 - conserved hypothetical protein

//

>NGHB#3446 19

CMPLX ---

HVO_A0051 - conserved hypothetical protein

HVO_A0052 - conserved hypothetical protein

//

>NGHB#3447 49

CMPLX ---

HVO_A0052 - conserved hypothetical protein

HVO_A0053 - conserved hypothetical protein

//

>NGHB#3448 71

CMPLX ---

HVO_A0053 - conserved hypothetical protein

HVO_A0054 - conserved hypothetical protein

//

>NGHB#3449 -1

CMPLX ---

HVO_A0054 - conserved hypothetical protein

HVO_A0055 - conserved hypothetical protein

//

>NGHB#3450 -4

CMPLX ---

HVO_A0055 - conserved hypothetical protein

HVO_A0056 - conserved hypothetical protein (nonfunctional)

//

>NGHB#3451 -4

CMPLX ---

HVO_A0056 - conserved hypothetical protein (nonfunctional)

HVO_A0056_A - conserved hypothetical protein (nonfunctional)

//

>NGHB#3452 72

CMPLX ---

HVO_A0058 - conserved hypothetical protein

HVO_A0059 - conserved hypothetical protein

//

>NGHB#3453 86

CMPLX ---

HVO_A0059 - conserved hypothetical protein

HVO_A0060 - conserved hypothetical protein

//

>NGHB#3454 136

CMPLX ---

HVO_A0060 - conserved hypothetical protein

HVO_A0061 - SWIM zinc finger domain protein

//

>NGHB#3458 891

CMPLX not

HVO_A0065 polB2 DNA-directed DNA polymerase B2

HVO_A0064 orc13 Orc1-type DNA replication protein

//

>NGHB#3459 1

CMPLX not

HVO_A0066 - IS1341-type transposase ISHvo24

HVO_A0065 polB2 DNA-directed DNA polymerase B2

//

>NGHB#3460 173

CMPLX ---

HVO_A0067 - conserved hypothetical protein

HVO_A0066 - IS1341-type transposase ISHvo24

//

>NGHB#3461 2

CMPLX ---

HVO_A0068 - conserved hypothetical protein

HVO_A0067 - conserved hypothetical protein

//

>NGHB#3462 273

CMPLX ---

HVO_A0070 - ribonuclease H domain protein (nonfunctional)

HVO_A0068 - conserved hypothetical protein

//

>NGHB#3465 192

CMPLX ---

HVO_A0073 - conserved hypothetical protein

HVO_A0072 orc12 Orc1-type DNA replication protein

//

>NGHB#3467 -11

CMPLX ---

HVO_A0074 - AAA-type ATPase domain protein

HVO_A0076 - conserved hypothetical protein

//

>NGHB#3471 190

CMPLX not

HVO_A0082 - IclR family transcription regulator

HVO_A0081 - ISH3-type transposase ISH51

//

>NGHB#3472 367

CMPLX not

HVO_A0083 - Rieske-type iron-sulfur protein (2Fe-2S)

HVO_A0082 - IclR family transcription regulator

//

>NGHB#3473 6

CMPLX not

HVO_A0084 - amidohydrolase domain protein

HVO_A0083 - Rieske-type iron-sulfur protein (2Fe-2S)

//

>NGHB#3476 258

CMPLX not

HVO_A0087 - fumarylacetoacetase family protein

HVO_A0086 - UspA domain protein

//

>NGHB#3477 119

CMPLX not

HVO_A0088 - FAD-dependent oxidoreductase (GlcD/DLD_GlcF/GlpC domain fusion protein)

HVO_A0087 - fumarylacetoacetase family protein

//

>NGHB#3478 -8

CMPLX not

HVO_A0089 - small CPxCG-related zinc finger protein

HVO_A0088 - FAD-dependent oxidoreductase (GlcD/DLD_GlcF/GlpC domain fusion protein)

//

>NGHB#3479 119

CMPLX not

HVO_A0090 gdoA gentisate 1,2-dioxygenase

HVO_A0089 - small CPxCG-related zinc finger protein

//

>NGHB#3483 440

CMPLX ---

HVO_A0093 - IclR family transcription regulator

HVO_A0094 - conserved hypothetical protein

//

>NGHB#3484 56

CMPLX ---

HVO_A0094 - conserved hypothetical protein

HVO_A0095 - amidohydrolase domain protein

//

>NGHB#3485 133

CMPLX not

HVO_A0095 - amidohydrolase domain protein

HVO_A0096 acd7 acyl-CoA dehydrogenase

//

>NGHB#3486 126

CMPLX not

HVO_A0096 acd7 acyl-CoA dehydrogenase

HVO_A0097 hbd2 3-hydroxyacyl-CoA dehydrogenase / enoyl-CoA hydratase

//

>NGHB#3487 58

CMPLX not

HVO_A0097 hbd2 3-hydroxyacyl-CoA dehydrogenase / enoyl-CoA hydratase

HVO_A0097A - acyl-CoA synthetase

//

>NGHB#3489 133

CMPLX not

HVO_A0100 - ISHwa16-type transposase ISHvo16

HVO_A0098 tfb12 transcription initiation factor TFB

//

>NGHB#3492 68

CMPLX ---

HVO_A0104 - conserved hypothetical protein

HVO_A0102 - SWIM zinc finger domain protein

//

>NGHB#3493 73

CMPLX ---

HVO_A0105 - conserved hypothetical protein

HVO_A0104 - conserved hypothetical protein

//

>NGHB#3494 86

CMPLX ---

HVO_A0106 - small CPxCG-related zinc finger protein

HVO_A0105 - conserved hypothetical protein

//

>NGHB#3495 173

CMPLX ---

HVO_A0107 - conserved hypothetical protein

HVO_A0106 - small CPxCG-related zinc finger protein

//

>NGHB#3496 18

CMPLX ---

HVO_A0108 - conserved hypothetical protein

HVO_A0107 - conserved hypothetical protein

//

>NGHB#3498 172

CMPLX ---

HVO_A0113A - conserved hypothetical protein (nonfunctional)

HVO_A0112 - hypothetical protein

//

>NGHB#3499 61

CMPLX ---

HVO_A0114 - HTH domain protein

HVO_A0113A - conserved hypothetical protein (nonfunctional)

//

>NGHB#3500 -20

CMPLX ---

HVO_A0115 - conserved hypothetical protein

HVO_A0114 - HTH domain protein

//

>NGHB#3501 141

CMPLX ---

HVO_A0116 - ISH8-type transposase ISHvo13

HVO_A0115 - conserved hypothetical protein

//

>NGHB#3503 319

CMPLX not

HVO_A0117 - ISH9-type transposase ISHvo1

HVO_A0118 - PadR family transcription regulator

//

>NGHB#3505 81

CMPLX ---

HVO_A0120 - conserved hypothetical protein

HVO_A0119 - cyclase family protein

//

>NGHB#3508 136

CMPLX ---

HVO_A0123 - conserved hypothetical protein

HVO_A0122 - hypothetical protein

//

>NGHB#3509 301

CMPLX ---

HVO_A0124 - PQQ repeat protein (nonfunctional)

HVO_A0123 - conserved hypothetical protein

//

>NGHB#3515 3

CMPLX ---

HVO_A0129 - conserved hypothetical protein

HVO_A0130 - conserved hypothetical protein

//

>NGHB#3516 70

CMPLX ---

HVO_A0133 - conserved hypothetical protein

HVO_A0132_A - ISH9-type transposase HfIRS2 (nonfunctional)

//

>NGHB#3518 974

CMPLX not

HVO_A0134 - ISH9-type transposase HfIRS1 (nonfunctional)

HVO_A0135 - HTH domain protein

//

>NGHB#3519 -8

CMPLX not

HVO_A0135 - HTH domain protein

HVO_A0136 - PemK family protein

//

>NGHB#3523 173

CMPLX not

HVO_A0140 - HD family hydrolase

HVO_A0139 - SWIM zinc finger domain protein

//

>NGHB#3524 221

CMPLX ---

HVO_A0141 - conserved hypothetical protein

HVO_A0140 - HD family hydrolase

//

>NGHB#3525 73

CMPLX ---

HVO_A0142 - conserved hypothetical protein

HVO_A0141 - conserved hypothetical protein

//

>NGHB#3526 98

CMPLX ---

HVO_A0143 - small CPxCG-related zinc finger protein

HVO_A0142 - conserved hypothetical protein

//

>NGHB#3528 2

CMPLX yes

HVO_A0146 tsgB4 ABC-type transport system permease protein (probable substrate sugar)

HVO_A0145 tsgD4 ABC-type transport system ATP-binding protein (probable substrate sugar)

//

>NGHB#3529 -1

CMPLX yes

HVO_A0147 tsgC4 ABC-type transport system permease protein (probable substrate sugar)

HVO_A0146 tsgB4 ABC-type transport system permease protein (probable substrate sugar)

//

>NGHB#3530 63

CMPLX yes

HVO_A0148 tsgA4 ABC-type transport system periplasmic substrate-binding protein (probable substrate sugar)

HVO_A0147 tsgC4 ABC-type transport system permease protein (probable substrate sugar)

//

>NGHB#3532 111

CMPLX not

HVO_A0149 - glycoside transferase domain protein

HVO_A0150 trmB1 TrmB family transcription regulator

//

>NGHB#3533 212

CMPLX not

HVO_A0150 trmB1 TrmB family transcription regulator

HVO_A0151 - ISH3-type transposase ISH51

//

>NGHB#3535 89

CMPLX ---

HVO_A0153 - UspA domain protein

HVO_A0152 - conserved hypothetical protein

//

>NGHB#3536 3

CMPLX not

HVO_A0154 - SSSF family transport protein

HVO_A0153 - UspA domain protein

//

>NGHB#3537 -4

CMPLX not

HVO_A0155 - DUF4212 family protein

HVO_A0154 - SSSF family transport protein

//

>NGHB#3538 55

CMPLX not

HVO_A0156 acs7 acyl-CoA synthetase

HVO_A0155 - DUF4212 family protein

//

>NGHB#3539 198

CMPLX not

HVO_A0157 boa4 integrase family protein / bat box HTH-10 family transcription regulator

HVO_A0156 acs7 acyl-CoA synthetase

//

>NGHB#3540 143

CMPLX not

HVO_A0158 acs8 acyl-CoA synthetase

HVO_A0157 boa4 integrase family protein / bat box HTH-10 family transcription regulator

//

>NGHB#3544 -4

CMPLX not

HVO_A0161 - TetR family transcription regulator

HVO_A0162 - MATE efflux family protein

//

>NGHB#3546 -4

CMPLX ---

HVO_A0164 - conserved hypothetical protein

HVO_A0163 prr proline racemase

//

>NGHB#3547 1

CMPLX ---

HVO_A0165 - SNF family transport protein

HVO_A0164 - conserved hypothetical protein

//

>NGHB#3549 30

CMPLX ---

HVO_A0166 - conserved hypothetical protein

HVO_A0167 - small CPxCG-related zinc finger protein

//

>NGHB#3552 105

CMPLX ---

HVO_A0169 - DUF106 family protein

HVO_A0170 - conserved hypothetical protein

//

>NGHB#3554 76

CMPLX ---

HVO_A0172 - conserved hypothetical protein

HVO_A0171 clc chloride channel protein

//

>NGHB#3556 173

CMPLX ---

HVO_A0173 - conserved hypothetical protein

HVO_A0174 secY2, sec61a2 protein translocase subunit SecY

//

>NGHB#3557 177

CMPLX not

HVO_A0174 secY2, sec61a2 protein translocase subunit SecY

HVO_A0175 cat3 transport protein (probable substrate cationic amino acids)

//

>NGHB#3559 -4

CMPLX yes

HVO_A0177 - ABC-type transport system ATP-binding protein

HVO_A0176 - ABC-type transport system permease protein

//

>NGHB#3561 64

CMPLX ---

HVO_A0178 atpD2 A-type ATP synthase subunit D

HVO_A0179 - conserved hypothetical protein

//

>NGHB#3562 -4

CMPLX ---

HVO_A0179 - conserved hypothetical protein

HVO_A0180 sph1 Smc-like protein Sph1

//

>NGHB#3564 58

CMPLX ---

HVO_A0182 - conserved hypothetical protein

HVO_A0181 - conserved hypothetical protein

//

>NGHB#3568 26

CMPLX ---

HVO_A0185 - hypothetical protein

HVO_A0186 - ATP-grasp domain protein

//

>NGHB#3570 106

CMPLX ---

HVO_A0188 - homolog to endonuclease VapC

HVO_A0187 - conserved hypothetical protein

//

>NGHB#3571 -4

CMPLX ---

HVO_A0189 - conserved hypothetical protein

HVO_A0188 - homolog to endonuclease VapC

//

>NGHB#3572 148

CMPLX ---

HVO_A0190 - pectin lyase domain protein

HVO_A0189 - conserved hypothetical protein

//

>NGHB#3574 -4

CMPLX ---

HVO_A0191 - hypothetical protein

HVO_A0192 - polysaccharide deacetylase domain protein

//

>NGHB#3575 337

CMPLX not

HVO_A0192 - polysaccharide deacetylase domain protein

HVO_A0194 - probable glycosyltransferase, type 2

//

>NGHB#3576 73

CMPLX not

HVO_A0194 - probable glycosyltransferase, type 2

HVO_A0195 - DUF1616 family protein

//

>NGHB#3578 454

CMPLX not

HVO_A0197 - probable RfbX family transport protein

HVO_A0196 - pectin lyase domain protein

//

>NGHB#3579 319

CMPLX ---

HVO_A0198 - conserved hypothetical protein

HVO_A0197 - probable RfbX family transport protein

//

>NGHB#3581 3137

CMPLX not

HVO_A0200 - asparagine synthase domain protein

HVO_A0205 cas6 CRISPR-associated endoribonuclease Cas6

//

>NGHB#3582 -1

CMPLX yes

HVO_A0205 cas6 CRISPR-associated endoribonuclease Cas6

HVO_A0206 cas8b CRISPR-associated protein Cas8b

//

>NGHB#3583 20

CMPLX yes

HVO_A0206 cas8b CRISPR-associated protein Cas8b

HVO_A0207 cas7 CRISPR-associated protein Cas7

//

>NGHB#3584 10

CMPLX yes

HVO_A0207 cas7 CRISPR-associated protein Cas7

HVO_A0208 cas5 CRISPR-associated protein Cas5, Hmari subtype

//

>NGHB#3585 24

CMPLX yes

HVO_A0208 cas5 CRISPR-associated protein Cas5, Hmari subtype

HVO_A0209 cas3 CRISPR-associated nuclease/helicase Cas3

//

>NGHB#3586 189

CMPLX yes

HVO_A0209 cas3 CRISPR-associated nuclease/helicase Cas3

HVO_A0210 cas4 CRISPR-associated exonuclease Cas4

//

>NGHB#3587 3

CMPLX yes

HVO_A0210 cas4 CRISPR-associated exonuclease Cas4

HVO_A0211 cas1 CRISPR-associated endonuclease Cas1

//

>NGHB#3588 1

CMPLX yes

HVO_A0211 cas1 CRISPR-associated endonuclease Cas1

HVO_A0212 cas2 CRISPR-associated endonuclease Cas2

//

>NGHB#3590 174

CMPLX not

HVO_A0214 - DUF354 family protein

HVO_A0213 - probable glycosyltransferase, type 1

//

>NGHB#3592 182

CMPLX not

HVO_A0215 - DUF457 family protein

HVO_A0216 - LpxA family protein

//

>NGHB#3593 -4

CMPLX not

HVO_A0216 - LpxA family protein

HVO_A0217 - DegT family aminotransferase

//

>NGHB#3594 -4

CMPLX not

HVO_A0217 - DegT family aminotransferase

HVO_A0218 - GFO family oxidoreductase

//

>NGHB#3595 -4

CMPLX not

HVO_A0218 - GFO family oxidoreductase

HVO_A0219 wecC probable nucleotide sugar dehydrogenase

//

>NGHB#3596 -4

CMPLX not

HVO_A0219 wecC probable nucleotide sugar dehydrogenase

HVO_A0220 wecB probable nucleotide sugar epimerase

//

>NGHB#3597 -8

CMPLX ---

HVO_A0220 wecB probable nucleotide sugar epimerase

HVO_A0221 - conserved hypothetical protein

//

>NGHB#3598 62

CMPLX ---

HVO_A0221 - conserved hypothetical protein

HVO_A0222 - conserved hypothetical protein

//

>NGHB#3599 88

CMPLX ---

HVO_A0222 - conserved hypothetical protein

HVO_A0223 - hypothetical protein

//

>NGHB#3600 -4

CMPLX ---

HVO_A0223 - hypothetical protein

HVO_A0224 - FemAB family protein

//

>NGHB#3601 88

CMPLX not

HVO_A0224 - FemAB family protein

HVO_A0225 - FemAB family protein

//

>NGHB#3604 30

CMPLX ---

HVO_A0227 - hypothetical protein

HVO_A0228 surE2 5'-nucleotidase SurE

//

>NGHB#3608 87

CMPLX ---

HVO_A0232 - hypothetical protein

HVO_A0231 - conserved hypothetical protein

//

>NGHB#3609 364

CMPLX ---

HVO_A0233 - hypothetical protein

HVO_A0232 - hypothetical protein

//

>NGHB#3610 24

CMPLX ---

HVO_A0234 - homolog to phiCh1-ORF95

HVO_A0233 - hypothetical protein

//

>NGHB#3611 11

CMPLX ---

HVO_A0235 - hypothetical protein

HVO_A0234 - homolog to phiCh1-ORF95

//

>NGHB#3612 10

CMPLX ---

HVO_A0236 - hypothetical protein

HVO_A0235 - hypothetical protein

//

>NGHB#3613 329

CMPLX ---

HVO_A0237 - adenine specific DNA methyltransferase (nonfunctional)

HVO_A0236 - hypothetical protein

//

>NGHB#3614 14

CMPLX not

HVO_A0238 - ISH5-type transposase ISHvo11

HVO_A0237 - adenine specific DNA methyltransferase (nonfunctional)

//

>NGHB#3615 85

CMPLX ---

HVO_A0239_A - conserved hypothetical protein

HVO_A0238 - ISH5-type transposase ISHvo11

//

>NGHB#3618 23

CMPLX ---

HVO_A0243 - conserved hypothetical protein

HVO_A0242A - conserved hypothetical protein (nonfunctional)

//

>NGHB#3619 33

CMPLX ---

HVO_A0244 - death-on-curing family protein

HVO_A0243 - conserved hypothetical protein

//

>NGHB#3620 62

CMPLX not

HVO_A0245 - HTH domain protein

HVO_A0244 - death-on-curing family protein

//

>NGHB#3621 149

CMPLX ---

HVO_A0246 - conserved hypothetical protein

HVO_A0245 - HTH domain protein

//

>NGHB#3622 546

CMPLX ---

HVO_A0247 - conserved hypothetical protein (nonfunctional)

HVO_A0246 - conserved hypothetical protein

//

>NGHB#3623 -20

CMPLX ---

HVO_A0248 - conserved hypothetical protein (nonfunctional)

HVO_A0247 - conserved hypothetical protein (nonfunctional)

//

>NGHB#3624 -4

CMPLX ---

HVO_A0249 - TrmB family transcription regulator

HVO_A0248 - conserved hypothetical protein (nonfunctional)

//

>NGHB#3625 300

CMPLX ---

HVO_A0250 - ISH3-type transposase ISH51

HVO_A0249_A - conserved hypothetical protein

//

>NGHB#3626 221

CMPLX not

HVO_A0251 - HTH domain protein

HVO_A0250 - ISH3-type transposase ISH51

//

>NGHB#3630 149

CMPLX not

HVO_A0254 - DUF234 domain protein

HVO_A0254_A - small CPxCG-related zinc finger protein

//

>NGHB#3631 211

CMPLX ---

HVO_A0254_A - small CPxCG-related zinc finger protein

HVO_A0256 - conserved hypothetical protein

//

>NGHB#3633 1023

CMPLX not

HVO_A0258 - ISH4-type transposase ISHvo6

HVO_A0257 orc7 Orc1-type DNA replication protein

//

>NGHB#3635 428

CMPLX not

HVO_A0262 - ISH3-type transposase ISH51

HVO_A0263 - probable secreted glycoprotein

//

>NGHB#3637 780

CMPLX not

HVO_A0266 - IclR family transcription regulator

HVO_A0265 - ISH14-type transposase HfIRS8 (nonfunctional)

//

>NGHB#3638 66

CMPLX not

HVO_A0267 dgoD2 D-galactonate dehydratase

HVO_A0266 - IclR family transcription regulator

//

>NGHB#3640 194

CMPLX not

HVO_A0268 - class II aldolase (homolog to L-fuculose-phosphate aldolase)

HVO_A0269 glpA2 glycerol-3-phosphate dehydrogenase subunit A

//

>NGHB#3641 -11

CMPLX yes

HVO_A0269 glpA2 glycerol-3-phosphate dehydrogenase subunit A

HVO_A0270 glpB2 glycerol-3-phosphate dehydrogenase subunit B

//

>NGHB#3642 62

CMPLX yes

HVO_A0270 glpB2 glycerol-3-phosphate dehydrogenase subunit B

HVO_A0271 glpC2 glycerol-3-phosphate dehydrogenase subunit C

//

>NGHB#3643 67

CMPLX not

HVO_A0271 glpC2 glycerol-3-phosphate dehydrogenase subunit C

HVO_A0272 - DUF336 family protein

//

>NGHB#3644 240

CMPLX not

HVO_A0272 - DUF336 family protein

HVO_A0273 - ThuA family protein

//

>NGHB#3645 137

CMPLX not

HVO_A0273 - ThuA family protein

HVO_A0274 suhB2 probable inositol-1(or 4)-monophosphatase / fructose-1,6-bisphosphatase, archaeal-type

//

>NGHB#3647 -8

CMPLX ---

HVO_A0276 - FGGY family carbohydrate kinase

HVO_A0275 - conserved hypothetical protein

//

>NGHB#3649 98

CMPLX not

HVO_A0277 rnhA4 ribonuclease H, type 1 (nonfunctional)

HVO_A0278 sfsA SfsA family DNA-binding protein

//

>NGHB#3651 281

CMPLX not

HVO_A0279_A tfs3 transcription elongation factor TFS

HVO_A0279 - ISH18-type transposase ISHvo9

//

>NGHB#3652 716

CMPLX not

HVO_A0280 - IclR family transcription regulator

HVO_A0279_A tfs3 transcription elongation factor TFS

//

>NGHB#3654 1

CMPLX not

HVO_A0281 tsgD5 ABC-type transport system ATP-binding protein (probable substrate sugar)

HVO_A0282 cre3 creatininase domain protein

//

>NGHB#3655 73

CMPLX not

HVO_A0282 cre3 creatininase domain protein

HVO_A0283 tsgA5 ABC-type transport system periplasmic substrate-binding protein (probable substrate sugar)

//

>NGHB#3656 9

CMPLX yes

HVO_A0283 tsgA5 ABC-type transport system periplasmic substrate-binding protein (probable substrate sugar)

HVO_A0284 tsgB5 ABC-type transport system permease protein (probable substrate sugar)

//

>NGHB#3657 -4

CMPLX yes

HVO_A0284 tsgB5 ABC-type transport system permease protein (probable substrate sugar)

HVO_A0285 tsgC5 ABC-type transport system permease protein (probable substrate sugar)

//

>NGHB#3658 44

CMPLX not

HVO_A0285 tsgC5 ABC-type transport system permease protein (probable substrate sugar)

HVO_A0286 - DUF187 family protein

//

>NGHB#3659 41

CMPLX not

HVO_A0286 - DUF187 family protein

HVO_A0287 - homolog to mandelate racemase / homolog to muconate lactonizing enzyme

//

>NGHB#3660 51

CMPLX not

HVO_A0287 - homolog to mandelate racemase / homolog to muconate lactonizing enzyme

HVO_A0288 - probable oxidoreductase (short-chain dehydrogenase family)

//

>NGHB#3661 11

CMPLX not

HVO_A0288 - probable oxidoreductase (short-chain dehydrogenase family)

HVO_A0289 ridA4 enamine/imine deaminase

//

>NGHB#3664 71

CMPLX ---

HVO_A0290A kdgK3 2-keto-3-deoxygluconate kinase (nonfunctional)

HVO_A0291 - conserved hypothetical protein

//

>NGHB#3667 1

CMPLX yes

HVO_A0293 - ABC-type transport system ATP-binding protein

HVO_A0294 - ABC-type transport system ATP-binding protein

//

>NGHB#3668 161

CMPLX not

HVO_A0294 - ABC-type transport system ATP-binding protein

HVO_A0295 amaB2 amidase (hydantoinase/carbamoylase family)

//

>NGHB#3669 2

CMPLX not

HVO_A0295 amaB2 amidase (hydantoinase/carbamoylase family)

HVO_A0295_A - luciferase family protein

//

>NGHB#3670 97

CMPLX not

HVO_A0295_A - luciferase family protein

HVO_A0296 - probable oxidoreductase (short-chain dehydrogenase family)

//

>NGHB#3671 60

CMPLX not

HVO_A0296 - probable oxidoreductase (short-chain dehydrogenase family)

HVO_A0297 - ABC-type transport system permease protein

//

>NGHB#3674 57

CMPLX yes

HVO_A0299 - ABC-type transport system periplasmic substrate-binding protein

HVO_A0300 - ABC-type transport system permease protein

//

>NGHB#3675 97

CMPLX not

HVO_A0300 - ABC-type transport system permease protein

HVO_A0301 - probable polysaccharide deacetylase

//

>NGHB#3678 51

CMPLX not

HVO_A0303 pucH2 probable allantoinase

HVO_A0304 - homolog to thermosome

//

>NGHB#3681 48

CMPLX not

HVO_A0306 - pyridoxal phosphate-dependent aminotransferase

HVO_A0307 - Lrp/AsnC family transcription regulator

//

>NGHB#3683 19

CMPLX ---

HVO_A0311 - probable halocin (homolog to halocin C8)

HVO_A0310 - conserved hypothetical protein

//

>NGHB#3686 5

CMPLX ---

HVO_A0314 - hypothetical protein

HVO_A0313 - cro/C1 family transcription regulator

//

>NGHB#3687 1

CMPLX ---

HVO_A0315 - conserved hypothetical protein

HVO_A0314 - hypothetical protein

//

>NGHB#3688 454

CMPLX ---

HVO_A0316 - conserved hypothetical protein

HVO_A0315 - conserved hypothetical protein

//

>NGHB#3690 1

CMPLX ---

HVO_A0317 - ArsR family transcription regulator

HVO_A0318 - conserved hypothetical protein

//

>NGHB#3692 395

CMPLX ---

HVO_A0320 - conserved hypothetical protein

HVO_A0319 - hypothetical protein

//

>NGHB#3693 261

CMPLX ---

HVO_A0321 - conserved hypothetical protein

HVO_A0320 - conserved hypothetical protein

//

>NGHB#3695 -4

CMPLX yes

HVO_A0322 - ABC-type transport system permease protein

HVO_A0323 - ABC-type transport system ATP-binding protein

//

>NGHB#3696 -4

CMPLX ---

HVO_A0323 - ABC-type transport system ATP-binding protein

HVO_A0324 - conserved hypothetical protein

//

>NGHB#3698 522

CMPLX not

HVO_A0326 bgaH beta-D-galactosidase

HVO_A0325 - UPF0121 family protein

//

>NGHB#3700 -4

CMPLX ---

HVO_A0327 - hypothetical protein

HVO_A0328 kdgK2 2-keto-3-deoxygalactonate kinase

//

>NGHB#3702 81

CMPLX not

HVO_A0330 - GFO family oxidoreductase

HVO_A0329 kdgA3 2-dehydro-3-deoxy-phosphogluconate aldolase, bacterial-type

//

>NGHB#3704 67

CMPLX not

HVO_A0331 dgoD1 D-galactonate dehydratase

HVO_A0332 - IclR family transcription regulator

//

>NGHB#3705 1147

CMPLX not

HVO_A0332 - IclR family transcription regulator

HVO_A0333 - SprT family protein

//

>NGHB#3709 -4

CMPLX yes

HVO_A0337 dppC7 ABC-type transport system permease protein (probable substrate dipeptide/oligopeptide)

HVO_A0336 dppDF7 ABC-type transport system ATP-binding protein (probable substrate dipeptide/oligopeptide)

//

>NGHB#3710 -1

CMPLX yes

HVO_A0338 dppB7 ABC-type transport system permease protein (probable substrate dipeptide/oligopeptide)

HVO_A0337 dppC7 ABC-type transport system permease protein (probable substrate dipeptide/oligopeptide)

//

>NGHB#3711 15

CMPLX yes

HVO_A0339 dppA7 ABC-type transport system periplasmic substrate-binding protein (probable substrate dipeptide/oligopeptide)

HVO_A0338 dppB7 ABC-type transport system permease protein (probable substrate dipeptide/oligopeptide)

//

>NGHB#3712 155

CMPLX ---

HVO_A0340 - hypothetical protein

HVO_A0339 dppA7 ABC-type transport system periplasmic substrate-binding protein (probable substrate dipeptide/oligopeptide)

//

>NGHB#3714 424

CMPLX not

HVO_A0341 amaB3 amidase (hydantoinase/carbamoylase family)

HVO_A0342 - IclR family transcription regulator

//

>NGHB#3715 171

CMPLX ---

HVO_A0342 - IclR family transcription regulator

HVO_A0343 - hypothetical protein

//

>NGHB#3717 -8

CMPLX not

HVO_A0345 cat4 transport protein (probable substrate cationic amino acids) (nonfunctional)

HVO_A0344 - UspA domain protein (nonfunctional)

//

>NGHB#3720 327

CMPLX ---

HVO_A0348A - hypothetical protein

HVO_A0348 - ISH7-type transposase ISHvo15 (nonfunctional)

//

>NGHB#3721 260

CMPLX ---

HVO_A0349 - ISH3-type transposase ISHvo22

HVO_A0348A - hypothetical protein

//

>NGHB#3723 -1

CMPLX ---

HVO_A0350 - conserved hypothetical protein

HVO_A0351 - hypothetical protein

//

>NGHB#3724 47

CMPLX not

HVO_A0353 - ISH3-type transposase ISH51 (nonfunctional)

HVO_A0354 - ISH3-type transposase ISH51 (nonfunctional)

//

>NGHB#3725 77

CMPLX not

HVO_A0354 - ISH3-type transposase ISH51 (nonfunctional)

HVO_A0355 - ISH5-type transposase ISHvo11

//

>NGHB#3726 -4

CMPLX ---

HVO_A0357 - conserved hypothetical protein

HVO_A0358 - conserved hypothetical protein

//

>NGHB#3727 546

CMPLX ---

HVO_A0358 - conserved hypothetical protein

HVO_A0359 - conserved hypothetical protein (nonfunctional)

//

>NGHB#3731 288

CMPLX ---

HVO_A0363 - conserved hypothetical protein

HVO_A0365 - conserved hypothetical protein (nonfunctional)

//

>NGHB#3733 111

CMPLX not

HVO_A0368 - RelE family protein

HVO_A0367 - ISH3-type transposase ISH51

//

>NGHB#3734 -8

CMPLX not

HVO_A0369 - CopG domain protein

HVO_A0368 - RelE family protein

//

>NGHB#3736 -4

CMPLX ---

HVO_A0370 - conserved hypothetical protein (nonfunctional)

HVO_A0371 - conserved hypothetical protein

//

>NGHB#3739 199

CMPLX ---

HVO_A0374 - conserved hypothetical protein

HVO_A0375 tfb11 transcription initiation factor TFB

//

>NGHB#3741 -4

CMPLX not

HVO_A0377 hyuE hydantoin racemase

HVO_A0376 pepQ2 probable Xaa-Pro dipeptidase

//

>NGHB#3742 -1

CMPLX not

HVO_A0378 hyuB2 N-methylhydantoinase (ATP-hydrolyzing) B

HVO_A0377 hyuE hydantoin racemase

//

>NGHB#3743 -4

CMPLX yes

HVO_A0379 hyuA2 N-methylhydantoinase (ATP-hydrolyzing) A

HVO_A0378 hyuB2 N-methylhydantoinase (ATP-hydrolyzing) B

//

>NGHB#3745 10

CMPLX yes

HVO_A0380 dppA8 ABC-type transport system periplasmic substrate-binding protein (probable substrate dipeptide/oligopeptide)

HVO_A0381 dppB8 ABC-type transport system permease protein (probable substrate dipeptide/oligopeptide)

//

>NGHB#3746 7

CMPLX yes

HVO_A0381 dppB8 ABC-type transport system permease protein (probable substrate dipeptide/oligopeptide)

HVO_A0382 dppC8 ABC-type transport system permease protein (probable substrate dipeptide/oligopeptide)

//

>NGHB#3747 -1

CMPLX yes

HVO_A0382 dppC8 ABC-type transport system permease protein (probable substrate dipeptide/oligopeptide)

HVO_A0383 dppD8 ABC-type transport system ATP-binding protein (probable substrate dipeptide/oligopeptide)

//

>NGHB#3748 -4

CMPLX yes

HVO_A0383 dppD8 ABC-type transport system ATP-binding protein (probable substrate dipeptide/oligopeptide)

HVO_A0384 dppF8 ABC-type transport system ATP-binding protein (probable substrate dipeptide/oligopeptide)

//

>NGHB#3749 102

CMPLX not

HVO_A0384 dppF8 ABC-type transport system ATP-binding protein (probable substrate dipeptide/oligopeptide)

HVO_A0385 hyuA3 N-methylhydantoinase (ATP-hydrolyzing) A

//

>NGHB#3750 -1

CMPLX yes

HVO_A0385 hyuA3 N-methylhydantoinase (ATP-hydrolyzing) A

HVO_A0386 hyuB3 N-methylhydantoinase (ATP-hydrolyzing) B

//

>NGHB#3751 -4

CMPLX not

HVO_A0386 hyuB3 N-methylhydantoinase (ATP-hydrolyzing) B

HVO_A0387 - cupin 2 barrel domain protein

//

>NGHB#3753 -4

CMPLX ---

HVO_A0390_A - conserved hypothetical protein (nonfunctional)

HVO_A0391 - conserved hypothetical protein

//

>NGHB#3754 65

CMPLX ---

HVO_A0391 - conserved hypothetical protein

HVO_A0392 - death-on-curing family protein

//

>NGHB#3755 2

CMPLX ---

HVO_A0392 - death-on-curing family protein

HVO_A0393 - conserved hypothetical protein

//

>NGHB#3756 308

CMPLX ---

HVO_A0393 - conserved hypothetical protein

HVO_A0394 - HTH domain protein

//

>NGHB#3757 -4

CMPLX ---

HVO_A0394 - HTH domain protein

HVO_A0395 - conserved hypothetical protein

//

>NGHB#3758 2

CMPLX ---

HVO_A0395 - conserved hypothetical protein

HVO_A0396 - SWIM zinc finger domain protein

//

>NGHB#3761 139

CMPLX ---

HVO_A0398 - conserved hypothetical protein

HVO_A0399 - conserved hypothetical protein

//

>NGHB#3762 101

CMPLX ---

HVO_A0399 - conserved hypothetical protein

HVO_A0400 - conserved hypothetical protein

//

>NGHB#3764 156

CMPLX ---

HVO_A0402 - conserved hypothetical protein

HVO_A0401 - Fido domain protein

//

>NGHB#3769 224

CMPLX ---

HVO_A0409 - conserved hypothetical protein

HVO_A0410 - PQQ repeat protein

//

>NGHB#3770 301

CMPLX ---

HVO_A0410 - PQQ repeat protein

HVO_A0411 - conserved hypothetical protein

//

>NGHB#3771 135

CMPLX ---

HVO_A0411 - conserved hypothetical protein

HVO_A0412 - hypothetical protein

//

>NGHB#3774 81

CMPLX ---

HVO_A0414 - conserved hypothetical protein

HVO_A0415 - cyclase family protein

//

>NGHB#3777 -20

CMPLX ---

HVO_A0417 - conserved hypothetical protein

HVO_A0418 - HTH domain protein

//

>NGHB#3778 -8

CMPLX ---

HVO_A0419 - HTH domain protein

HVO_A0421 - conserved hypothetical protein (nonfunctional)

//

>NGHB#3779 308

CMPLX not

HVO_A0423 - ISH3-type transposase ISH51

HVO_A0424 boa6 HTH-10 family transcription regulator

//

>NGHB#3781 1

CMPLX yes

HVO_A0426 dppD9 ABC-type transport system ATP-binding protein (probable substrate dipeptide/oligopeptide)

HVO_A0425 dppF9 ABC-type transport system ATP-binding protein (probable substrate dipeptide/oligopeptide)

//

>NGHB#3782 3

CMPLX yes

HVO_A0426_A dppC9 ABC-type transport system permease protein (probable substrate dipeptide/oligopeptide)

HVO_A0426 dppD9 ABC-type transport system ATP-binding protein (probable substrate dipeptide/oligopeptide)

//

>NGHB#3783 1

CMPLX yes

HVO_A0427 dppB9 ABC-type transport system permease protein (probable substrate dipeptide/oligopeptide)

HVO_A0426_A dppC9 ABC-type transport system permease protein (probable substrate dipeptide/oligopeptide)

//

>NGHB#3784 27

CMPLX yes

HVO_A0428 dppA9 ABC-type transport system periplasmic substrate-binding protein (probable substrate dipeptide/oligopeptide)

HVO_A0427 dppB9 ABC-type transport system permease protein (probable substrate dipeptide/oligopeptide)

//

>NGHB#3785 119

CMPLX not

HVO_A0429 - homolog to D-aminoacylase

HVO_A0428 dppA9 ABC-type transport system periplasmic substrate-binding protein (probable substrate dipeptide/oligopeptide)

//

>NGHB#3786 109

CMPLX not

HVO_A0429_A - homolog to dipeptide epimerase

HVO_A0429 - homolog to D-aminoacylase

//

>NGHB#3787 -4

CMPLX not

HVO_A0430 - DUF1611 family protein

HVO_A0429_A - homolog to dipeptide epimerase

//

>NGHB#3789 150

CMPLX ---

HVO_A0430_A - conserved hypothetical protein

HVO_A0431 - ISH5-type transposase ISHvo11

//

>NGHB#3790 82

CMPLX ---

HVO_A0431 - ISH5-type transposase ISHvo11

HVO_A0432 - conserved hypothetical protein

//

>NGHB#3791 162

CMPLX ---

HVO_A0432 - conserved hypothetical protein

HVO_A0433 - ISH3-type transposase ISH51

//

>NGHB#3792 -4

CMPLX not

HVO_A0436 - ArsR family transcription regulator

HVO_A0435 - DUF2204 family protein

//

>NGHB#3794 -4

CMPLX ---

HVO_A0437 - conserved hypothetical protein

HVO_A0438 - conserved hypothetical protein

//

>NGHB#3795 406

CMPLX ---

HVO_A0438 - conserved hypothetical protein

HVO_A0439 - DUF2800 family protein

//

>NGHB#3797 -1

CMPLX not

HVO_A0441 rad25a DNA repair helicase Rad25

HVO_A0440 - DUF790 family protein

//

>NGHB#3798 116

CMPLX not

HVO_A0442 - CcbP family protein

HVO_A0441 rad25a DNA repair helicase Rad25

//

>NGHB#3799 297

CMPLX ---

HVO_A0443 - conserved hypothetical protein

HVO_A0442 - CcbP family protein

//

>NGHB#3800 -4

CMPLX ---

HVO_A0444 - conserved hypothetical protein

HVO_A0443 - conserved hypothetical protein

//

>NGHB#3801 158

CMPLX ---

HVO_A0444A - SWIM zinc finger domain protein (nonfunctional)

HVO_A0444 - conserved hypothetical protein

//

>NGHB#3803 276

CMPLX not

HVO_A0447 - peptidase S54 family protein

HVO_A0448 tfb10 transcription initiation factor TFB

//

>NGHB#3805 8

CMPLX not

HVO_A0451 - DASS family transport protein

HVO_A0452 - UspA domain protein

//

>NGHB#3806 168

CMPLX ---

HVO_A0452 - UspA domain protein

HVO_A0453 - conserved hypothetical protein

//

>NGHB#3807 141

CMPLX ---

HVO_A0453 - conserved hypothetical protein

HVO_A0454 - conserved hypothetical protein

//

>NGHB#3808 100

CMPLX ---

HVO_A0454 - conserved hypothetical protein

HVO_A0455 - conserved hypothetical protein

//

>NGHB#3809 66

CMPLX ---

HVO_A0455 - conserved hypothetical protein

HVO_A0456 - ISH8-type transposase ISHvo12

//

>NGHB#3812 -4

CMPLX ---

HVO_A0458 - ParA domain protein

HVO_A0459 - conserved hypothetical protein

//

>NGHB#3814 -1

CMPLX yes

HVO_A0461 phnE2 ABC-type transport system permease protein (probable substrate phosphate/phosphonate)

HVO_A0460 phnE3 ABC-type transport system permease protein (probable substrate phosphate/phosphonate)

//

>NGHB#3820 -1

CMPLX ---

HVO_A0466 - conserved hypothetical protein

HVO_A0467 - RND superfamily permease

//

>NGHB#3823 78

CMPLX not

HVO_A0469 - pirin domain protein

HVO_A0470 - glyoxalase domain protein

//

>NGHB#3824 -1

CMPLX not

HVO_A0470 - glyoxalase domain protein

HVO_A0471 - alpha/beta hydrolase fold protein

//

>NGHB#3828 221

CMPLX ---

HVO_A0475 sod1 superoxide dismutase (Mn)

HVO_A0474 - conserved hypothetical protein

//

>NGHB#3829 107

CMPLX ---

HVO_A0476 - conserved hypothetical protein

HVO_A0475 sod1 superoxide dismutase (Mn)

//

>NGHB#3831 62

CMPLX yes

HVO_A0477 pstS2 ABC-type transport system periplasmic substrate-binding protein (probable substrate phosphate)

HVO_A0478 pstC2 ABC-type transport system permease protein (probable substrate phosphate)

//

>NGHB#3832 -1

CMPLX yes

HVO_A0478 pstC2 ABC-type transport system permease protein (probable substrate phosphate)

HVO_A0479 pstA2 ABC-type transport system permease protein (probable substrate phosphate)

//

>NGHB#3833 2

CMPLX yes

HVO_A0479 pstA2 ABC-type transport system permease protein (probable substrate phosphate)

HVO_A0480 pstB2 ABC-type transport system ATP-binding protein (probable substrate phosphate)

//

>NGHB#3834 229

CMPLX not

HVO_A0480 pstB2 ABC-type transport system ATP-binding protein (probable substrate phosphate)

HVO_A0481 phoU4 PhoU domain protein

//

>NGHB#3839 169

CMPLX not

HVO_A0486 hypE1 HypE family protein

HVO_A0487 cbiA, cobB cobyrinate a,c-diamide synthase

//

>NGHB#3840 72

CMPLX not

HVO_A0487 cbiA, cobB cobyrinate a,c-diamide synthase

HVO_A0488 cobA, cobO cob(I)alamin adenosyltransferase

//

>NGHB#3842 0

CMPLX not

HVO_A0490 - family 3 CoA transferase

HVO_A0489 - homolog to homocitrate synthase

//

>NGHB#3843 -4

CMPLX not

HVO_A0491 tsgD6 ABC-type transport system ATP-binding protein (probable substrate sugar)

HVO_A0490 - family 3 CoA transferase

//

>NGHB#3844 -1

CMPLX yes

HVO_A0492 tsgC6 ABC-type transport system permease protein (probable substrate sugar)

HVO_A0491 tsgD6 ABC-type transport system ATP-binding protein (probable substrate sugar)

//

>NGHB#3845 1

CMPLX yes

HVO_A0493 tsgB6 ABC-type transport system permease protein (probable substrate sugar)

HVO_A0492 tsgC6 ABC-type transport system permease protein (probable substrate sugar)

//

>NGHB#3846 -4

CMPLX yes

HVO_A0494 tsgA6 ABC-type transport system periplasmic substrate-binding protein (probable substrate sugar)

HVO_A0493 tsgB6 ABC-type transport system permease protein (probable substrate sugar)

//

>NGHB#3847 166

CMPLX not

HVO_A0495 boa7 HTH-10 family transcription regulator

HVO_A0494 tsgA6 ABC-type transport system periplasmic substrate-binding protein (probable substrate sugar)

//

>NGHB#3848 141

CMPLX not

HVO_A0496 - UspA domain protein

HVO_A0495 boa7 HTH-10 family transcription regulator

//

>NGHB#3850 123

CMPLX ---

HVO_A0497 - conserved hypothetical protein

HVO_A0498 - conserved hypothetical protein

//

>NGHB#3851 82

CMPLX ---

HVO_A0498 - conserved hypothetical protein

HVO_A0499 - conserved hypothetical protein

//

>NGHB#3854 511

CMPLX not

HVO_A0501 - homolog to S-adenosylmethionine-dependent methyltransferase

HVO_A0503 - amidohydrolase domain protein

//

>NGHB#3855 107

CMPLX ---

HVO_A0503 - amidohydrolase domain protein

HVO_A0504 - conserved hypothetical protein

//

>NGHB#3857 81

CMPLX not

HVO_A0506 boa8 HTH-10 family transcription regulator

HVO_A0505 fadA4 enoyl-CoA hydratase

//

>NGHB#3859 5

CMPLX yes

HVO_A0507 paaA 1,2-phenylacetyl-CoA epoxidase subunit A

HVO_A0508 paaB 1,2-phenylacetyl-CoA epoxidase subunit B

//

>NGHB#3860 2

CMPLX yes

HVO_A0508 paaB 1,2-phenylacetyl-CoA epoxidase subunit B

HVO_A0509 paaC 1,2-phenylacetyl-CoA epoxidase subunit C

//

>NGHB#3861 -8

CMPLX not

HVO_A0509 paaC 1,2-phenylacetyl-CoA epoxidase subunit C

HVO_A0510 - DUF59 family protein

//

>NGHB#3862 3

CMPLX not

HVO_A0510 - DUF59 family protein

HVO_A0511 - small CPxCG-related zinc finger protein

//

>NGHB#3864 68

CMPLX not

HVO_A0513 - PaaI family protein

HVO_A0512 aldH4 aldehyde dehydrogenase

//

>NGHB#3865 79

CMPLX not

HVO_A0514 livF4 ABC-type transport system ATP-binding protein (probable substrate branched-chain amino acids)

HVO_A0513 - PaaI family protein

//

>NGHB#3866 -4

CMPLX yes

HVO_A0515 livG4 ABC-type transport system ATP-binding protein (probable substrate branched-chain amino acids)

HVO_A0514 livF4 ABC-type transport system ATP-binding protein (probable substrate branched-chain amino acids)

//

>NGHB#3867 -8

CMPLX yes

HVO_A0516 livHM4 ABC-type transport system permease protein (probable substrate branched-chain amino acids)

HVO_A0515 livG4 ABC-type transport system ATP-binding protein (probable substrate branched-chain amino acids)

//

>NGHB#3868 49

CMPLX not

HVO_A0517 - luciferase-type oxidoreductase

HVO_A0516 livHM4 ABC-type transport system permease protein (probable substrate branched-chain amino acids)

//

>NGHB#3872 -4

CMPLX not

HVO_A0522 acaB4 acetyl-CoA C-acyltransferase

HVO_A0523 - UPF0219 family protein

//

>NGHB#3873 86

CMPLX not

HVO_A0523 - UPF0219 family protein

HVO_A0524 hbd4 3-hydroxyacyl-CoA dehydrogenase

//

>NGHB#3874 -4

CMPLX not

HVO_A0524 hbd4 3-hydroxyacyl-CoA dehydrogenase

HVO_A0525 fadA5 enoyl-CoA hydratase

//

>NGHB#3876 243

CMPLX ---

HVO_A0527 - IclR family transcription regulator

HVO_A0526 - hypothetical protein

//

>NGHB#3878 4

CMPLX not

HVO_A0528 - EthD domain protein

HVO_A0529 ilvB2 acetolactate synthase large subunit

//

>NGHB#3879 89

CMPLX not

HVO_A0529 ilvB2 acetolactate synthase large subunit

HVO_A0530 - GalE family epimerase/dehydratase

//

>NGHB#3881 78

CMPLX ---

HVO_A0532 - cupin 2 barrel domain protein

HVO_A0531 - conserved hypothetical protein

//

>NGHB#3882 65

CMPLX not

HVO_A0533 - HD family hydrolase

HVO_A0532 - cupin 2 barrel domain protein

//

>NGHB#3883 3

CMPLX not

HVO_A0534 - EthD domain protein

HVO_A0533 - HD family hydrolase

//

>NGHB#3886 -4

CMPLX yes

HVO_A0537 hyuA1 N-methylhydantoinase (ATP-hydrolyzing) A

HVO_A0536 hyuB1 N-methylhydantoinase (ATP-hydrolyzing) B

//

>NGHB#3887 -1

CMPLX not

HVO_A0538 mntH2 NRAMP family transport protein (probable substrate divalent metal cation)

HVO_A0537 hyuA1 N-methylhydantoinase (ATP-hydrolyzing) A

//

>NGHB#3888 190

CMPLX not

HVO_A0539 - CopG domain protein

HVO_A0538 mntH2 NRAMP family transport protein (probable substrate divalent metal cation)

//

>NGHB#3889 103

CMPLX not

HVO_A0540 - homolog to receiver/sensor box histidine kinase

HVO_A0539 - CopG domain protein

//

>NGHB#3890 168

CMPLX not

HVO_A0541 - ABC-type transport system periplasmic substrate-binding protein

HVO_A0540 - homolog to receiver/sensor box histidine kinase

//

>NGHB#3891 85

CMPLX ---

HVO_A0542 - hypothetical protein

HVO_A0541 - ABC-type transport system periplasmic substrate-binding protein

//

>NGHB#3892 -4

CMPLX ---

HVO_A0543 - small CPxCG-related zinc finger protein

HVO_A0542 - hypothetical protein

//

>NGHB#3894 78

CMPLX ---

HVO_A0544 - conserved hypothetical protein

HVO_A0545 - TetR family transcription regulator

//

>NGHB#3898 105

CMPLX not

HVO_A0549 nosL1 NosL family protein

HVO_A0548 - DoxX domain protein

//

>NGHB#3899 168

CMPLX not

HVO_A0550 - receiver/sensor box histidine kinase

HVO_A0549 nosL1 NosL family protein

//

>NGHB#3900 122

CMPLX not

HVO_A0551 acs9 acyl-CoA synthetase

HVO_A0550 - receiver/sensor box histidine kinase

//

>NGHB#3904 327

CMPLX not

HVO_A0555 - DUF162 family protein

HVO_A0556 - small CPxCG-related zinc finger protein

//

>NGHB#3908 -8

CMPLX not

HVO_A0560 hutI imidazolonepropionase

HVO_A0559 hutH histidine ammonia-lyase

//

>NGHB#3909 -8

CMPLX not

HVO_A0561 hutG formimidoylglutamase

HVO_A0560 hutI imidazolonepropionase

//

>NGHB#3910 -4

CMPLX not

HVO_A0562 hutU urocanate hydratase

HVO_A0561 hutG formimidoylglutamase

//

>NGHB#3915 -4

CMPLX yes

HVO_A0567 leuC2 3-isopropylmalate dehydratase large subunit

HVO_A0566 leuD2 3-isopropylmalate dehydratase small subunit

//

>NGHB#3916 1

CMPLX not

HVO_A0568 - hydroxymethylglutaryl-CoA lyase

HVO_A0567 leuC2 3-isopropylmalate dehydratase large subunit

//

>NGHB#3917 -4

CMPLX not

HVO_A0569 - family 3 CoA transferase

HVO_A0568 - hydroxymethylglutaryl-CoA lyase

//

>NGHB#3918 195

CMPLX not

HVO_A0570 - probable metal-dependent hydrolase

HVO_A0569 - family 3 CoA transferase

//

>NGHB#3919 -4

CMPLX not

HVO_A0571 - isochorismatase family protein

HVO_A0570 - probable metal-dependent hydrolase

//

>NGHB#3920 66

CMPLX not

HVO_A0572 - probable flavin-dependent oxidoreductase

HVO_A0571 - isochorismatase family protein

//

>NGHB#3921 -4

CMPLX not

HVO_A0574 - DUF1486 family protein

HVO_A0572 - probable flavin-dependent oxidoreductase

//

>NGHB#3923 164

CMPLX not

HVO_A0575 - homolog to succinate dehydrogenase flavoprotein

HVO_A0576 livJ5 ABC-type transport system periplasmic substrate-binding protein (probable substrate branched-chain amino acids)

//

>NGHB#3924 94

CMPLX yes

HVO_A0576 livJ5 ABC-type transport system periplasmic substrate-binding protein (probable substrate branched-chain amino acids)

HVO_A0577 livH5 ABC-type transport system permease protein (probable substrate branched-chain amino acids)

//

>NGHB#3925 -4

CMPLX yes

HVO_A0577 livH5 ABC-type transport system permease protein (probable substrate branched-chain amino acids)

HVO_A0578 livM5 ABC-type transport system permease protein (probable substrate branched-chain amino acids)

//

>NGHB#3926 -1

CMPLX yes

HVO_A0578 livM5 ABC-type transport system permease protein (probable substrate branched-chain amino acids)

HVO_A0579 livG5 ABC-type transport system ATP-binding protein (probable substrate branched-chain amino acids)

//

>NGHB#3927 -8

CMPLX yes

HVO_A0579 livG5 ABC-type transport system ATP-binding protein (probable substrate branched-chain amino acids)

HVO_A0580 livF5 ABC-type transport system ATP-binding protein (probable substrate branched-chain amino acids)

//

>NGHB#3928 1

CMPLX not

HVO_A0580 livF5 ABC-type transport system ATP-binding protein (probable substrate branched-chain amino acids)

HVO_A0581 - PrpD family protein

//

>NGHB#3929 41

CMPLX not

HVO_A0581 - PrpD family protein

HVO_A0582 - PrpD family protein

//

>NGHB#3930 69

CMPLX not

HVO_A0582 - PrpD family protein

HVO_A0583 - IclR family transcription regulator

//

>NGHB#3933 100

CMPLX not

HVO_A0586 graD3 sugar nucleotidyltransferase

HVO_A0587 - receiver/sensor box protein

//

>NGHB#3934 215

CMPLX not

HVO_A0587 - receiver/sensor box protein

HVO_A0588 - HTH domain protein

//

>NGHB#3936 161

CMPLX ---

HVO_A0590 - conserved hypothetical protein

HVO_A0589 - HxlR family transcription regulator

//

>NGHB#3937 99

CMPLX ---

HVO_A0591 - DICT domain protein

HVO_A0590 - conserved hypothetical protein

//

>NGHB#3940 207

CMPLX not

HVO_A0594 glmS glutamine--fructose-6-phosphate aminotransferase (isomerizing)

HVO_A0593 - ArsR family transcription regulator

//

>NGHB#3941 -1

CMPLX not

HVO_A0595 graD4 sugar nucleotidyltransferase

HVO_A0594 glmS glutamine--fructose-6-phosphate aminotransferase (isomerizing)

//

>NGHB#3942 473

CMPLX ---

HVO_A0596 - conserved hypothetical protein

HVO_A0595 graD4 sugar nucleotidyltransferase

//

>NGHB#3943 471

CMPLX ---

HVO_A0597 - conserved hypothetical protein

HVO_A0596 - conserved hypothetical protein

//

>NGHB#3945 40

CMPLX ---

HVO_A0598 - PadR family transcription regulator

HVO_A0599 - hypothetical protein

//

>NGHB#3946 345

CMPLX ---

HVO_A0599 - hypothetical protein

HVO_A0600 - conserved hypothetical protein

//

>NGHB#3948 116

CMPLX not

HVO_A0602 pmm1 phosphohexomutase (phosphoglucomutase / phosphomannomutase)

HVO_A0601 nosL2 NosL family protein

//

>NGHB#3949 183

CMPLX ---

HVO_A0604 - conserved hypothetical protein

HVO_A0602 pmm1 phosphohexomutase (phosphoglucomutase / phosphomannomutase)

//

>NGHB#3950 176

CMPLX ---

HVO_A0605 - F420-dependent NADP oxidoreductase family protein

HVO_A0604 - conserved hypothetical protein

//

>NGHB#3951 148

CMPLX not

HVO_A0606 - major facilitator superfamily transport protein

HVO_A0605 - F420-dependent NADP oxidoreductase family protein

//

>NGHB#3952 300

CMPLX not

HVO_A0609 znuB2 ABC-type transport system permease protein (probable substrate zinc) (nonfunctional)

HVO_A0607A - methyl-accepting chemotaxis sensory transducer (nonfunctional)

//

>NGHB#3953 -4

CMPLX yes

HVO_A0610 znuC2 ABC-type transport system ATP-binding protein (probable substrate zinc)

HVO_A0609 znuB2 ABC-type transport system permease protein (probable substrate zinc) (nonfunctional)

//

>NGHB#3954 -4

CMPLX yes

HVO_A0611 znuA2 ABC-type transport system periplasmic substrate-binding protein (probable substrate zinc)

HVO_A0610 znuC2 ABC-type transport system ATP-binding protein (probable substrate zinc)

//

>NGHB#3955 172

CMPLX not

HVO_A0613 - DUF2306 family protein

HVO_A0611 znuA2 ABC-type transport system periplasmic substrate-binding protein (probable substrate zinc)

//

>NGHB#3958 154

CMPLX not

HVO_A0616 - TetR family transcription regulator

HVO_A0615 cspA5 cold shock protein

//

>NGHB#3959 82

CMPLX not

HVO_A0617 - DMT superfamily transport protein

HVO_A0616 - TetR family transcription regulator

//

>NGHB#3960 234

CMPLX not

HVO_A0618 - flavin-dependent pyridine nucleotide oxidoreductase (homolog to coenzyme A disulfide reductase)

HVO_A0617 - DMT superfamily transport protein

//

>NGHB#3961 110

CMPLX ---

HVO_A0619 - conserved hypothetical protein

HVO_A0618 - flavin-dependent pyridine nucleotide oxidoreductase (homolog to coenzyme A disulfide reductase)

//

>NGHB#3962 1

CMPLX ---

HVO_A0620 - conserved hypothetical protein

HVO_A0619 - conserved hypothetical protein

//

>NGHB#3964 38

CMPLX ---

HVO_A0621 - conserved hypothetical protein

HVO_A0622 - conserved hypothetical protein

//

>NGHB#3966 216

CMPLX not

HVO_A0624 cadA, zntA1 P-type transport ATPase (probable substrate zinc/cadmium)

HVO_A0623 - HiPIP domain protein

//

>NGHB#3967 95

CMPLX ---

HVO_A0625 - conserved hypothetical protein

HVO_A0624 cadA, zntA1 P-type transport ATPase (probable substrate zinc/cadmium)

//

>NGHB#3968 -4

CMPLX ---

HVO_A0626 - conserved hypothetical protein

HVO_A0625 - conserved hypothetical protein

//

>NGHB#3969 113

CMPLX ---

HVO_A0627 - conserved hypothetical protein

HVO_A0626 - conserved hypothetical protein

//

>NGHB#3973 422

CMPLX ---

HVO_A0631 - conserved hypothetical protein

HVO_A0632 pilA5 pilin PilA

//

>NGHB#3974 238

CMPLX yes

HVO_A0632 pilA5 pilin PilA

HVO_A0633 pilA6 pilin PilA

//

>NGHB#3975 164

CMPLX not

HVO_A0633 pilA6 pilin PilA

HVO_A0634 - M20 family amidohydrolase (homolog to succinyl-diaminopimelate desuccinylase)

//

>NGHB#3976 6

CMPLX not

HVO_A0634 - M20 family amidohydrolase (homolog to succinyl-diaminopimelate desuccinylase)

HVO_A0635 sufS2 probable cysteine desulfurase

//

>NGHB#3977 62

CMPLX not

HVO_A0635 sufS2 probable cysteine desulfurase

HVO_A0636 ybaK YbaK domain protein

//

>NGHB#3979 405

CMPLX ---

HVO_A0638 - hypothetical protein

HVO_A0637 tif1A2 translation initiation factor aIF-1A

//
