# Supplementary material for: Unidirectional gene pairs in archaea and bacteria require overlaps or very short intergenic distances for translational coupling via termination-reinitiation and often encode subunits of heteromeric complexes
Source: Front Microbiol. 2023 Nov 9;14:1291523. doi: 10.3389/fmicb.2023.1291523 (PMC10666635; doi:10.3389/fmicb.2023.1291523)
Supplement: Supplementary file 3 [file Table_2.DOCX]

**Supplementary Table S2.** Prediction of heteromeric complex formation for gene products of unidirectional gene pairs of *E. coli*. The genome of *E. coli* K-12 MG1655 was manually inspected. For all unidirectional gene pairs it was predicted whether their gene products are known to be subunits of heteromeric complexes (“CMPLX yes”) or whether no information about complex formation exists (“CMPLX not”). For all unidirectional gene pairs four result lines were generated, i.e. 1) the number of the gene pair and the intergenic distance of the genes, 2) the prediction of complex formation, 3) the gene number and functional annotation of the upstream gene, and 4) the gene number and functional annotation of the downstream gene.

>NGHB#1 81

CMPLX not

b0001 thrL thr operon leader peptide

b0002 thrA fused aspartate kinase/homoserine dehydrogenase1

//

>NGHB#2 1

CMPLX not

b0002 thrA fused aspartate kinase/homoserine dehydrogenase1

b0003 thrB homoserine kinase

//

>NGHB#3 0

CMPLX not

b0003 thrB homoserine kinase

b0004 thrC threonine synthase

//

>NGHB#4 213

CMPLX not

b0004 thrC threonine synthase

b0005 yaaX DUF2502 domain-containing protein YaaX

//

>NGHB#6 69

CMPLX not

b0007 yaaJ putative transporter YaaJ

b0006 yaaA peroxide stress resistance protein YaaA

//

>NGHB#8 114

CMPLX not

b0008 talB transaldolase B

b0009 mog molybdopterin adenylyltransferase

//

>NGHB#10 148

CMPLX not

b0011 yaaW putative enzyme-specific chaperone YaaW

b0010 satP acetate/succinate:H(+) symporter

//

>NGHB#14 88

CMPLX yes

b0014 dnaK chaperone protein DnaK

b0015 dnaJ chaperone protein DnaJ

//

>NGHB#15 146

CMPLX not

b0015 dnaJ chaperone protein DnaJ

b0016 insL1 IS186/IS421 transposase

//

>NGHB#17 -210

CMPLX not

b4412 hokC protein HokC

b0018 mokC regulatory protein MokC

//

>NGHB#19 59

CMPLX not

b0019 nhaA Na(+):H(+) antiporter NhaA

b0020 nhaR DNA-binding transcriptional activator NhaR

//

>NGHB#21 -82

CMPLX tnp

b0022 insA1 IS1 protein InsA

b0021 insB1 IS1 protein InsB

//

>NGHB#22 306

CMPLX not

b0023 rpsT 30S ribosomal subunit protein S20

b0022 insA1 IS1 protein InsA

//

>NGHB#24 7

CMPLX not

b0024 yaaY DUF2575 domain-containing protein YaaY

b0025 ribF bifunctional riboflavin kinase/FMNadenylyltransferase

//

>NGHB#25 42

CMPLX not

b0025 ribF bifunctional riboflavin kinase/FMNadenylyltransferase

b0026 ileS isoleucine--tRNA ligase

//

>NGHB#26 -1

CMPLX not

b0026 ileS isoleucine--tRNA ligase

b0027 lspA lipoprotein signal peptidase

//

>NGHB#27 124

CMPLX not

b0027 lspA lipoprotein signal peptidase

b0028 fkpB peptidyl-prolyl cis-trans isomerase FkpB

//

>NGHB#28 1

CMPLX not

b0028 fkpB peptidyl-prolyl cis-trans isomerase FkpB

b0029 ispH 1-hydroxy-2-methyl-2-(E)-butenyl 4-diphosphatereductase

//

>NGHB#29 65

CMPLX not

b0029 ispH 1-hydroxy-2-methyl-2-(E)-butenyl 4-diphosphatereductase

b0030 rihC ribonucleoside hydrolase RihC

//

>NGHB#30 166

CMPLX not

b0030 rihC ribonucleoside hydrolase RihC

b0031 dapB 4-hydroxy-tetrahydrodipicolinate reductase

//

>NGHB#31 455

CMPLX not

b0031 dapB 4-hydroxy-tetrahydrodipicolinate reductase

b0032 carA carbamoyl phosphate synthetase subunit alpha

//

>NGHB#32 17

CMPLX yes

b0032 carA carbamoyl phosphate synthetase subunit alpha

b0033 carB carbamoyl phosphate synthetase subunit beta

//

>NGHB#33 261

CMPLX not

b0033 carB carbamoyl phosphate synthetase subunit beta

b0034 caiF DNA-binding transcriptional activator CaiF

//

>NGHB#35 5

CMPLX not

b0036 caiD crotonobetainyl-CoA hydratase

b0035 caiE putative transferase CaiE

//

>NGHB#36 108

CMPLX not

b0037 caiC carnitine--CoA ligase

b0036 caiD crotonobetainyl-CoA hydratase

//

>NGHB#37 73

CMPLX not

b0038 caiB gamma-butyrobetainyl-CoA:carnitine CoAtransferase

b0037 caiC carnitine--CoA ligase

//

>NGHB#38 128

CMPLX not

b0039 caiA crotonobetainyl-CoA reductase

b0038 caiB gamma-butyrobetainyl-CoA:carnitine CoAtransferase

//

>NGHB#39 30

CMPLX not

b0040 caiT L-carnitine:gamma-butyrobetaine antiporter

b0039 caiA crotonobetainyl-CoA reductase

//

>NGHB#41 14

CMPLX yes

b0041 fixA putative electron transfer flavoprotein FixA

b0042 fixB putative electron transfer flavoprotein FixB

//

>NGHB#42 50

CMPLX yes

b0042 fixB putative electron transfer flavoprotein FixB

b0043 fixC putative oxidoreductase FixC

//

>NGHB#43 -4

CMPLX yes

b0043 fixC putative oxidoreductase FixC

b0044 fixX putative ferredoxin FixX

//

>NGHB#44 56

CMPLX not

b0044 fixX putative ferredoxin FixX

b0045 yaaU putative transporter YaaU

//

>NGHB#45 107

CMPLX not

b0045 yaaU putative transporter YaaU

b0046 kefF regulator of KefC-mediated potassium transportand quinone oxidoreductase

//

>NGHB#46 -8

CMPLX yes

b0046 kefF regulator of KefC-mediated potassium transportand quinone oxidoreductase

b0047 kefC K(+) : H(+) antiporter KefC

//

>NGHB#47 191

CMPLX not

b0047 kefC K(+) : H(+) antiporter KefC

b0048 folA dihydrofolate reductase

//

>NGHB#49 6

CMPLX not

b0050 apaG DUF525 domain-containing protein ApaG

b0049 apaH diadenosine tetraphosphatase

//

>NGHB#50 2

CMPLX not

b0051 rsmA 16S rRNA m(6)2A1518,m(6)2A1519dimethyltransferase

b0050 apaG DUF525 domain-containing protein ApaG

//

>NGHB#51 -4

CMPLX not

b0052 pdxA 4-hydroxythreonine-4-phosphate dehydrogenase

b0051 rsmA 16S rRNA m(6)2A1518,m(6)2A1519dimethyltransferase

//

>NGHB#52 -1

CMPLX not

b0053 surA chaperone SurA

b0052 pdxA 4-hydroxythreonine-4-phosphate dehydrogenase

//

>NGHB#53 52

CMPLX not

b0054 lptD lipopolysaccharide assembly protein LptD

b0053 surA chaperone SurA

//

>NGHB#55 294

CMPLX ---

b0055 djlA co-chaperone protein DjlA

b0056 yabP putative uncharacterized protein YabP

//

>NGHB#56 -4

CMPLX ---

b0056 yabP putative uncharacterized protein YabP

b0057 yabQ protein YabQ

//

>NGHB#58 11

CMPLX not

b0059 rapA RNA polymerase-binding ATPase and RNAP recyclingfactor

b0058 rluA 23S rRNA pseudouridine(746) and tRNApseudouridine(32) synthase

//

>NGHB#59 164

CMPLX not

b0060 polB DNA polymerase II

b0059 rapA RNA polymerase-binding ATPase and RNAP recyclingfactor

//

>NGHB#60 74

CMPLX not

b0061 araD L-ribulose-5-phosphate 4-epimerase AraD

b0060 polB DNA polymerase II

//

>NGHB#61 284

CMPLX not

b0062 araA L-arabinose isomerase

b0061 araD L-ribulose-5-phosphate 4-epimerase AraD

//

>NGHB#62 10

CMPLX not

b0063 araB ribulokinase

b0062 araA L-arabinose isomerase

//

>NGHB#64 85

CMPLX not

b0064 araC DNA-binding transcriptional dual regulator AraC

b0065 yabI DedA family protein YabI

//

>NGHB#66 -17

CMPLX yes

b0067 thiP thiamine ABC transporter membrane subunit

b0066 thiQ thiamine ABC transporter ATP binding subunit

//

>NGHB#67 -25

CMPLX yes

b0068 thiB thiamine ABC transporter periplasmic bindingprotein

b0067 thiP thiamine ABC transporter membrane subunit

//

>NGHB#68 163

CMPLX not

b0069 sgrR DNA-binding transcriptional dual regulator SgrR

b0068 thiB thiamine ABC transporter periplasmic bindingprotein

//

>NGHB#70 101

CMPLX not

b4662 sgrT PtsG glucose transporter inhibitor

b0070 setA sugar exporter SetA

//

>NGHB#72 10

CMPLX yes

b0072 leuC 3-isopropylmalate dehydratase subunit LeuC

b0071 leuD 3-isopropylmalate dehydratase subunit LeuD

//

>NGHB#73 2

CMPLX not

b0073 leuB 3-isopropylmalate dehydrogenase

b0072 leuC 3-isopropylmalate dehydratase subunit LeuC

//

>NGHB#74 -1

CMPLX not

b0074 leuA 2-isopropylmalate synthase

b0073 leuB 3-isopropylmalate dehydrogenase

//

>NGHB#75 92

CMPLX not

b0075 leuL leu operon leader peptide

b0074 leuA 2-isopropylmalate synthase

//

>NGHB#79 2

CMPLX yes

b0077 ilvI acetolactate synthase/acetohydroxybutanoatesynthase, catalytic subunit

b0078 ilvH acetolactate synthase/acetohydroxybutanoatesynthase, regulatory subunit

//

>NGHB#80 179

CMPLX not

b0078 ilvH acetolactate synthase/acetohydroxybutanoatesynthase, regulatory subunit

b0080 cra DNA-binding transcriptional dual regulator Cra

//

>NGHB#81 601

CMPLX not

b0080 cra DNA-binding transcriptional dual regulator Cra

b0081 mraZ DNA-binding transcriptional repressor MraZ

//

>NGHB#82 1

CMPLX not

b0081 mraZ DNA-binding transcriptional repressor MraZ

b0082 rsmH 16S rRNA m(4)C1402 methyltransferase

//

>NGHB#83 -4

CMPLX not

b0082 rsmH 16S rRNA m(4)C1402 methyltransferase

b0083 ftsL cell division protein FtsL

//

>NGHB#84 15

CMPLX not

b0083 ftsL cell division protein FtsL

b0084 ftsI peptidoglycan DD-transpeptidase FtsI

//

>NGHB#85 -14

CMPLX not

b0084 ftsI peptidoglycan DD-transpeptidase FtsI

b0085 murE UDP-N-acetylmuramoyl-L-alanyl-D-glutamate--2,6-diaminopimelate ligase

//

>NGHB#86 -4

CMPLX not

b0085 murE UDP-N-acetylmuramoyl-L-alanyl-D-glutamate--2,6-diaminopimelate ligase

b0086 murF D-alanyl-D-alanine-adding enzyme

//

>NGHB#87 -7

CMPLX not

b0086 murF D-alanyl-D-alanine-adding enzyme

b0087 mraY phospho-N-acetylmuramoyl-pentapeptide-transferase

//

>NGHB#88 2

CMPLX not

b0087 mraY phospho-N-acetylmuramoyl-pentapeptide-transferase

b0088 murD UDP-N-acetylmuramoyl-L-alanine--D-glutamateligase

//

>NGHB#89 -1

CMPLX not

b0088 murD UDP-N-acetylmuramoyl-L-alanine--D-glutamateligase

b0089 ftsW essential cell division protein FtsW

//

>NGHB#90 -4

CMPLX not

b0089 ftsW essential cell division protein FtsW

b0090 murG N-acetylglucosaminyl transferase

//

>NGHB#91 53

CMPLX not

b0090 murG N-acetylglucosaminyl transferase

b0091 murC UDP-N-acetylmuramate--L-alanine ligase

//

>NGHB#92 -8

CMPLX not

b0091 murC UDP-N-acetylmuramate--L-alanine ligase

b0092 ddlB D-alanine--D-alanine ligase B

//

>NGHB#93 1

CMPLX not

b0092 ddlB D-alanine--D-alanine ligase B

b0093 ftsQ cell division protein FtsQ

//

>NGHB#94 -4

CMPLX yes

b0093 ftsQ cell division protein FtsQ

b0094 ftsA cell division protein FtsA

//

>NGHB#95 60

CMPLX yes

b0094 ftsA cell division protein FtsA

b0095 ftsZ cell division protein FtsZ

//

>NGHB#96 100

CMPLX not

b0095 ftsZ cell division protein FtsZ

b0096 lpxC UDP-3-O-acyl-N-acetylglucosamine deacetylase

//

>NGHB#97 230

CMPLX not

b0096 lpxC UDP-3-O-acyl-N-acetylglucosamine deacetylase

b0097 secM SecA translation regulator

//

>NGHB#98 61

CMPLX yes

b0097 secM SecA translation regulator

b0098 secA protein translocation ATPase

//

>NGHB#99 59

CMPLX not

b0098 secA protein translocation ATPase

b0099 mutT 8-oxo-dGTP diphosphatase

//

>NGHB#101 9

CMPLX not

b0102 zapD cell division factor ZapD

b0101 yacG DNA gyrase inhibitor YacG

//

>NGHB#102 -1

CMPLX not

b0103 coaE dephospho-CoA kinase

b0102 zapD cell division factor ZapD

//

>NGHB#104 155

CMPLX not

b4727 yacM protein YacM

b0104 guaC GMP reductase

//

>NGHB#106 -11

CMPLX not

b0107 hofB T2SSE family protein

b0106 hofC inner membrane protein HofC

//

>NGHB#107 9

CMPLX not

b0108 ppdD prepilin-type N-terminal cleavage/methylationdomain-containing protein PpdD

b0107 hofB T2SSE family protein

//

>NGHB#108 202

CMPLX not

b0109 nadC quinolinate phosphoribosyltransferase(decarboxylating)

b0108 ppdD prepilin-type N-terminal cleavage/methylationdomain-containing protein PpdD

//

>NGHB#110 -4

CMPLX not

b0110 ampD 1,6-anhydro-N-acetylmuramoyl-L-alanine amidase

b0111 ampE protein AmpE

//

>NGHB#113 160

CMPLX not

b0113 pdhR DNA-binding transcriptional dual regulator PdhR

b0114 aceE pyruvate dehydrogenase E1 component

//

>NGHB#114 14

CMPLX yes

b0114 aceE pyruvate dehydrogenase E1 component

b0115 aceF pyruvate dehydrogenase, E2 subunit

//

>NGHB#115 324

CMPLX yes

b0115 aceF pyruvate dehydrogenase, E2 subunit

b0116 lpd lipoamide dehydrogenase

//

>NGHB#118 175

CMPLX ---

b0118 acnB hypothetical protein

b0119 yacL UPF0231 family protein YacL

//

>NGHB#120 15

CMPLX not

b0121 speE spermidine synthase

b0120 speD S-adenosylmethionine decarboxylase proenzyme

//

>NGHB#121 105

CMPLX not

b0122 yacC putative lipoprotein YacC

b0121 speE spermidine synthase

//

>NGHB#127 -4

CMPLX yes

b0127 yadG putative ABC transporter ATP-binding proteinYadG

b0128 yadH putative ABC transporter membrane subunit YadH

//

>NGHB#128 104

CMPLX not

b0128 yadH putative ABC transporter membrane subunit YadH

b0129 yadI putative PTS enzyme IIA component YadI

//

>NGHB#129 63

CMPLX not

b0129 yadI putative PTS enzyme IIA component YadI

b0130 yadE putative polysaccharide deacetylase lipoproteinYadE

//

>NGHB#133 11

CMPLX not

b0134 panB 3-methyl-2-oxobutanoatehydroxymethyltransferase

b0133 panC pantothenate synthetase

//

>NGHB#134 113

CMPLX not

b0135 yadC fimbrial tip-adhesin YadC

b0134 panB 3-methyl-2-oxobutanoatehydroxymethyltransferase

//

>NGHB#135 49

CMPLX not

b0136 yadK putative fimbrial protein YadK

b0135 yadC fimbrial tip-adhesin YadC

//

>NGHB#136 26

CMPLX yes

b0137 yadL putative fimbrial protein YadL

b0136 yadK putative fimbrial protein YadK

//

>NGHB#137 11

CMPLX yes

b0138 yadM putative fimbrial protein YadM

b0137 yadL putative fimbrial protein YadL

//

>NGHB#138 16

CMPLX not

b0139 htrE putative fimbrial usher protein HtrE

b0138 yadM putative fimbrial protein YadM

//

>NGHB#139 34

CMPLX not

b0140 yadV putative fimbrial chaperone YadV

b0139 htrE putative fimbrial usher protein HtrE

//

>NGHB#140 97

CMPLX not

b0141 yadN putative fimbrial protein YadN

b0140 yadV putative fimbrial chaperone YadV

//

>NGHB#141 369

CMPLX not

b0142 folK 2-amino-4-hydroxy-6-hydroxymethyldihydropteridine diphosphokinase

b0141 yadN putative fimbrial protein YadN

//

>NGHB#142 -4

CMPLX not

b0143 pcnB poly(A) polymerase I

b0142 folK 2-amino-4-hydroxy-6-hydroxymethyldihydropteridine diphosphokinase

//

>NGHB#143 59

CMPLX not

b0144 gluQ glutamyl-Q tRNA(Asp) synthetase

b0143 pcnB poly(A) polymerase I

//

>NGHB#144 36

CMPLX not

b0145 dksA RNA polymerase-binding transcription factorDksA

b0144 gluQ glutamyl-Q tRNA(Asp) synthetase

//

>NGHB#145 177

CMPLX not

b0146 sfsA putative DNA-binding transcriptional regulatorof maltose metabolism

b0145 dksA RNA polymerase-binding transcription factorDksA

//

>NGHB#146 14

CMPLX not

b0147 thpR RNA 2',3'-cyclic phosphodiesterase

b0146 sfsA putative DNA-binding transcriptional regulatorof maltose metabolism

//

>NGHB#148 330

CMPLX not

b0148 hrpB RNA-dependent NTPase HrpB

b0149 mrcB PBP1Bgamma

//

>NGHB#149 219

CMPLX not

b0149 mrcB PBP1Bgamma

b0150 fhuA ferrichrome outer membrane transporter/phagereceptor

//

>NGHB#150 50

CMPLX not

b0150 fhuA ferrichrome outer membrane transporter/phagereceptor

b0151 fhuC iron(III) hydroxamate ABC transporter ATPbinding subunit

//

>NGHB#151 -1

CMPLX yes

b0151 fhuC iron(III) hydroxamate ABC transporter ATPbinding subunit

b0152 fhuD iron(III) hydroxamate ABC transporterperiplasmic binding protein

//

>NGHB#152 -4

CMPLX yes

b0152 fhuD iron(III) hydroxamate ABC transporterperiplasmic binding protein

b0153 fhuB iron(III) hydroxamate ABC transporter membranesubunit

//

>NGHB#155 11

CMPLX not

b4765 yadX protein YadX

b0155 clcA chloride:H(+) antiporter ClcA

//

>NGHB#156 23

CMPLX not

b0155 clcA chloride:H(+) antiporter ClcA

b4728 yadW protein YadW

//

>NGHB#157 -8

CMPLX not

b4728 yadW protein YadW

b0156 erpA iron-sulfur cluster insertion protein ErpA

//

>NGHB#159 37

CMPLX not

b0158 btuF vitamin B12 ABC transporter periplasmic bindingprotein

b0157 yadS PF03458 family protein YadS

//

>NGHB#160 -8

CMPLX not

b0159 mtn 5'-methylthioadenosine/S-adenosylhomocysteinenucleosidase

b0158 btuF vitamin B12 ABC transporter periplasmic bindingprotein

//

>NGHB#162 129

CMPLX not

b0160 dgt dGTP triphosphohydrolase

b0161 degP periplasmic serine endoprotease DegP

//

>NGHB#163 154

CMPLX not

b0161 degP periplasmic serine endoprotease DegP

b0162 cdaR DNA-binding transcriptional activator CdaR

//

>NGHB#165 161

CMPLX not

b0164 yaeI phosphodiesterase YaeI

b0163 yaeH DUF3461 domain-containing protein YaeH

//

>NGHB#166 53

CMPLX not

b0166 dapD tetrahydrodipicolinate succinylase

b0164 yaeI phosphodiesterase YaeI

//

>NGHB#167 30

CMPLX not

b0167 glnD PII uridylyltransferase/uridylyl removingenzyme

b0166 dapD tetrahydrodipicolinate succinylase

//

>NGHB#168 61

CMPLX not

b0168 map methionine aminopeptidase

b0167 glnD PII uridylyltransferase/uridylyl removingenzyme

//

>NGHB#170 257

CMPLX not

b0169 rpsB 30S ribosomal subunit protein S2

b0170 tsf protein chain elongation factor EF-Ts

//

>NGHB#171 146

CMPLX not

b0170 tsf protein chain elongation factor EF-Ts

b0171 pyrH UMP kinase

//

>NGHB#172 291

CMPLX not

b0171 pyrH UMP kinase

b0172 frr ribosome-recycling factor

//

>NGHB#173 91

CMPLX not

b0172 frr ribosome-recycling factor

b0173 dxr 1-deoxy-D-xylulose 5-phosphate reductoisomerase

//

>NGHB#174 185

CMPLX not

b0173 dxr 1-deoxy-D-xylulose 5-phosphate reductoisomerase

b0174 uppS ditrans,polycis-undecaprenyl-diphosphatesynthase [(2E,6E)-farnesyl-diphosphate specific]

//

>NGHB#175 12

CMPLX not

b0174 uppS ditrans,polycis-undecaprenyl-diphosphatesynthase [(2E,6E)-farnesyl-diphosphate specific]

b0175 cdsA CDP-diglyceride synthetase

//

>NGHB#176 11

CMPLX not

b0175 cdsA CDP-diglyceride synthetase

b0176 rseP intramembrane zinc metalloprotease RseP

//

>NGHB#177 29

CMPLX not

b0176 rseP intramembrane zinc metalloprotease RseP

b0177 bamA outer membrane protein assembly factor BamA

//

>NGHB#178 121

CMPLX not

b0177 bamA outer membrane protein assembly factor BamA

b0178 skp periplasmic chaperone Skp

//

>NGHB#179 3

CMPLX not

b0178 skp periplasmic chaperone Skp

b0179 lpxD UDP-3-O-(3-hydroxymyristoyl)glucosamineN-acyltransferase

//

>NGHB#180 104

CMPLX not

b0179 lpxD UDP-3-O-(3-hydroxymyristoyl)glucosamineN-acyltransferase

b0180 fabZ 3-hydroxy-acyl-[acyl-carrier-protein]dehydratase

//

>NGHB#181 3

CMPLX not

b0180 fabZ 3-hydroxy-acyl-[acyl-carrier-protein]dehydratase

b0181 lpxA acyl-[acyl-carrier-protein]--UDP-N-acetylglucosamine O-acyltransferase

//

>NGHB#182 -1

CMPLX not

b0181 lpxA acyl-[acyl-carrier-protein]--UDP-N-acetylglucosamine O-acyltransferase

b0182 lpxB lipid A disaccharide synthase

//

>NGHB#183 -4

CMPLX not

b0182 lpxB lipid A disaccharide synthase

b0183 rnhB RNase HII

//

>NGHB#184 36

CMPLX not

b0183 rnhB RNase HII

b0184 dnaE DNA polymerase III subunit alpha

//

>NGHB#185 12

CMPLX not

b0184 dnaE DNA polymerase III subunit alpha

b0185 accA acetyl-CoA carboxyltransferase subunit alpha

//

>NGHB#186 98

CMPLX not

b0185 accA acetyl-CoA carboxyltransferase subunit alpha

b0186 ldcC lysine decarboxylase 2

//

>NGHB#187 56

CMPLX not

b0186 ldcC lysine decarboxylase 2

b0187 yaeR VOC domain-containing protein YaeR

//

>NGHB#188 64

CMPLX not

b0187 yaeR VOC domain-containing protein YaeR

b0188 tilS tRNA(Ile)-lysidine synthetase

//

>NGHB#190 -8

CMPLX not

b4406 yaeP UPF0253 family protein YaeP

b0189 rof modulator of Rho-dependent transcriptiontermination

//

>NGHB#192 -4

CMPLX ---

b0190 yaeQ uncharacterized protein YaeQ

b0191 arfB peptidyl-tRNA hydrolase, ribosome rescue factor

//

>NGHB#193 13

CMPLX not

b0191 arfB peptidyl-tRNA hydrolase, ribosome rescue factor

b0192 nlpE sensor lipoprotein NlpE

//

>NGHB#195 53

CMPLX not

b0194 proS proline--tRNA ligase

b0193 yaeF peptidase C92 family protein YaeF

//

>NGHB#196 111

CMPLX not

b0195 trmO tRNA m(6)t(6)A37 methyltransferase

b0194 proS proline--tRNA ligase

//

>NGHB#197 -4

CMPLX not

b0196 rcsF sensor lipoprotein RcsF

b0195 trmO tRNA m(6)t(6)A37 methyltransferase

//

>NGHB#198 117

CMPLX not

b0197 metQ L-methionine/D-methionine ABC transportermembrane anchored binding protein

b0196 rcsF sensor lipoprotein RcsF

//

>NGHB#199 39

CMPLX yes

b0198 metI L-methionine/D-methionine ABC transportermembrane subunit

b0197 metQ L-methionine/D-methionine ABC transportermembrane anchored binding protein

//

>NGHB#200 -8

CMPLX yes

b0199 metN L-methionine/D-methionine ABC transporter ATPbinding subunit

b0198 metI L-methionine/D-methionine ABC transportermembrane subunit

//

>NGHB#202 5758

CMPLX not

b0200 gmhB D-glycero-beta-D-manno-heptose-1,7-bisphosphate7-phosphatase

b0207 dkgB methylglyoxal reductase DkgB

//

>NGHB#205 3

CMPLX not

b0209 yafD endonuclease/exonuclease/phosphatasedomain-containing protein YafD

b0210 yafE putative S-adenosylmethionine-dependentmethyltransferase

//

>NGHB#207 71

CMPLX not

b0212 gloB hydroxyacylglutathione hydrolase GloB

b0211 mltD membrane-bound lytic murein transglycosylase D

//

>NGHB#211 536

CMPLX not

b0215 dnaQ DNA polymerase III subunit epsilon

b0217 yafT lipoprotein YafT

//

>NGHB#213 682

CMPLX ---

b0219 yafV 2-oxoglutaramate amidase

b4586 ykfM uncharacterized protein YkfM

//

>NGHB#217 205

CMPLX not

b0222 gmhA D-sedoheptulose 7-phosphate isomerase

b0223 yafJ putative glutamine amidotransferase YafJ

//

>NGHB#219 155

CMPLX not

b0225 yafQ ribosome-dependent mRNA interferase toxin YafQ

b0224 ldtF L,D-transpeptidase domain-containing proteinLdtF

//

>NGHB#220 2

CMPLX not

b0226 dinJ antitoxin/DNA-binding transcriptional repressorDinJ

b0225 yafQ ribosome-dependent mRNA interferase toxin YafQ

//

>NGHB#222 175

CMPLX not

b0227 yafL NlpC/P60 family protein YafL

b0228 rayT REP-associated tyrosine transposase

//

>NGHB#223 2763

CMPLX not

b0228 rayT REP-associated tyrosine transposase

b0231 dinB DNA polymerase IV

//

>NGHB#224 51

CMPLX not

b0231 dinB DNA polymerase IV

b0232 yafN antitoxin YafN

//

>NGHB#225 2

CMPLX yes

b0232 yafN antitoxin YafN

b0233 yafO ribosome-dependent mRNA interferase toxin YafO

//

>NGHB#226 9

CMPLX not

b0233 yafO ribosome-dependent mRNA interferase toxin YafO

b0234 yafP putative acyltransferase with acyl-CoAN-acyltransferase domain

//

>NGHB#229 91

CMPLX not

b0238 gpt xanthine-guanine phsophoribosyltransferase

b0239 frsA fermentation-respiration switch protein

//

>NGHB#230 57

CMPLX not

b0239 frsA fermentation-respiration switch protein

b0240 crl RNA polymerase holoenzyme assembly factor Crl

//

>NGHB#231 -82

CMPLX tnp

b4709 insA9 IS1 repressor TnpA

b4710 insB9 IS1 transposase B

//

>NGHB#233 11

CMPLX not

b0242 proB glutamate 5-kinase

b0243 proA glutamate-5-semialdehyde dehydrogenase

//

>NGHB#235 20

CMPLX yes

b0246 yafW antitoxin of the YkfI-YafW toxin-antitoxin pair

b0245 ykfI CP4-6 prophage; toxin of the YkfI-YafWtoxin-antitoxin system

//

>NGHB#236 18

CMPLX not

b4504 ykfH DUF987 domain-containing protein YkfH

b0246 yafW antitoxin of the YkfI-YafW toxin-antitoxin pair

//

>NGHB#237 8

CMPLX not

b0247 ykfG CP4-6 prophage; RadC-like JAB domain-containingprotein YkfG

b4504 ykfH DUF987 domain-containing protein YkfH

//

>NGHB#238 15

CMPLX not

b0248 yafX CP4-6 prophage; protein YafX

b0247 ykfG CP4-6 prophage; RadC-like JAB domain-containingprotein YkfG

//

>NGHB#239 97

CMPLX not

b0249 ykfF CP4-6 prophage; protein YkfF

b0248 yafX CP4-6 prophage; protein YafX

//

>NGHB#240 76

CMPLX not

b0250 ykfB CP4-6 prophage; protein YkfB

b0249 ykfF CP4-6 prophage; protein YkfF

//

>NGHB#241 22

CMPLX not

b0251 yafY CP4-6 prophage; inner membrane lipoprotein YafY

b0250 ykfB CP4-6 prophage; protein YkfB

//

>NGHB#242 630

CMPLX not

b0252 yafZ CP4-6 prophage; DUF932 domain-containing proteinYafZ

b0251 yafY CP4-6 prophage; inner membrane lipoprotein YafY

//

>NGHB#243 91

CMPLX not

b0253 ykfA CP4-6 prophage; putative GTP-binding proteinYkfA

b0252 yafZ CP4-6 prophage; DUF932 domain-containing proteinYafZ

//

>NGHB#244 328

CMPLX not

b0254 perR CP4-6 prophage; putative transcriptionalregulator PerR

b0253 ykfA CP4-6 prophage; putative GTP-binding proteinYkfA

//

>NGHB#248 -14

CMPLX not

b0260 mmuP CP4-6 prophage; S-methyl-L-methioninetransporter

b0261 mmuM CP4-6 prophage; homocysteineS-methyltransferase

//

>NGHB#250 375

CMPLX not

b0264 insB2 IS1 protein InsB

b0262 afuC CP4-6 prophage; ABC transporter ATP-bindingprotein AfuC

//

>NGHB#251 -82

CMPLX tnp

b0265 insA2 IS1 protein InsA

b0264 insB2 IS1 protein InsB

//

>NGHB#252 509

CMPLX not

b0266 yagB CP4-6 prophage; orphan antitoxin YagB

b0265 insA2 IS1 protein InsA

//

>NGHB#253 93

CMPLX not

b0267 yagA CP4-6 prophage; integrase core domain-containingprotein YagA

b0266 yagB CP4-6 prophage; orphan antitoxin YagB

//

>NGHB#255 14

CMPLX not

b0268 yagE CP4-6 prophage; putative2-dehydro-3-deoxygluconate aldolase

b0269 yagF CP4-6 prophage; D-xylonate dehydratase

//

>NGHB#256 226

CMPLX not

b0269 yagF CP4-6 prophage; D-xylonate dehydratase

b0270 yagG CP4-6 prophage; putative D-xylonate transporterYagG

//

>NGHB#257 11

CMPLX not

b0270 yagG CP4-6 prophage; putative D-xylonate transporterYagG

b0271 yagH CP4-6 prophage; putativexylosidase/arabinosidase

//

>NGHB#259 138

CMPLX not

b0273 argF CP4-6 prophage; ornithine carbamoyltransferaseArgF

b0272 xynR CP4-6 prophage; DNA-binding transcriptionalrepressor XynR

//

>NGHB#260 -31

CMPLX not

b4766 argL putative translational regulatory protein ArgL

b0273 argF CP4-6 prophage; ornithine carbamoyltransferaseArgF

//

>NGHB#263 -82

CMPLX tnp

b0275 insA3 IS1 protein InsA

b0274 insB3 IS1 protein InsB

//

>NGHB#266 271

CMPLX ---

b0278 yagL CP4-6 prophage; resolvase-like catalyticdomain-containing protein YagL

b0277 yagK CP4-6 prophage; uncharacterized protein YagK

//

>NGHB#267 26

CMPLX not

b0279 yagM CP4-6 prophage; protein YagM

b0278 yagL CP4-6 prophage; resolvase-like catalyticdomain-containing protein YagL

//

>NGHB#268 118

CMPLX not

b4729 ykgV CP4-6 prophage; protein YkgV

b0279 yagM CP4-6 prophage; protein YagM

//

>NGHB#269 -4

CMPLX not

b0280 yagN CP4-6 prophage; protein YagN

b4729 ykgV CP4-6 prophage; protein YkgV

//

>NGHB#270 116

CMPLX not

b0281 intF CP4-6 prophage; putative phage integrase

b0280 yagN CP4-6 prophage; protein YagN

//

>NGHB#271 284

CMPLX not

b0282 yagP putative LysR family substrate bindingdomain-containing protein YagP

b0281 intF CP4-6 prophage; putative phage integrase

//

>NGHB#272 -22

CMPLX not

b0283 paoD molybdenum cofactor insertion chaperone PaoD

b0282 yagP putative LysR family substrate bindingdomain-containing protein YagP

//

>NGHB#273 9

CMPLX not

b0284 paoC aldehyde dehydrogenase: molybdenumcofactor-binding subunit

b0283 paoD molybdenum cofactor insertion chaperone PaoD

//

>NGHB#274 -4

CMPLX yes

b0285 paoB aldehyde dehydrogenase, FAD-binding subunit

b0284 paoC aldehyde dehydrogenase: molybdenumcofactor-binding subunit

//

>NGHB#275 -4

CMPLX yes

b0286 paoA aldehyde dehydrogenase, Fe-S subunit

b0285 paoB aldehyde dehydrogenase, FAD-binding subunit

//

>NGHB#278 312

CMPLX not

b0289 ecpE putative fimbrial chaperone EcpE

b0288 ykgJ putative zinc- or iron-chelatingdomain-containing protein YkgJ

//

>NGHB#279 -32

CMPLX not

b0290 ecpD fimbrial adhesin EcpD

b0289 ecpE putative fimbrial chaperone EcpE

//

>NGHB#280 -11

CMPLX not

b0291 ecpC putative fimbrial usher protein EcpC

b0290 ecpD fimbrial adhesin EcpD

//

>NGHB#281 25

CMPLX not

b0292 ecpB putative fimbrial chaperone EcpB

b0291 ecpC putative fimbrial usher protein EcpC

//

>NGHB#282 57

CMPLX not

b0293 ecpA common pilus major subunit

b0292 ecpB putative fimbrial chaperone EcpB

//

>NGHB#283 74

CMPLX not

b0294 ecpR DNA-binding transcriptional dual regulator MatA

b0293 ecpA common pilus major subunit

//

>NGHB#286 -1

CMPLX yes

b0296 ykgM 50S ribosomal subunit protein L31B

b4506 ykgO 50S ribosomal subunit protein L36B

//

>NGHB#287 363

CMPLX not

b4671 ykgR putative membrane protein YkgR

b0296 ykgM 50S ribosomal subunit protein L31B

//

>NGHB#289 -4

CMPLX tnp

b0298 insE1 IS3 element protein InsE

b0299 insF1 IS3 element protein InsF

//

>NGHB#291 11

CMPLX not

b0303 rclB DUF1471 domain-containing protein RclB

b0301 rclC reactive chlorine species resistance protein C

//

>NGHB#292 108

CMPLX not

b0304 rclA cupric reductase RclA

b0303 rclB DUF1471 domain-containing protein RclB

//

>NGHB#294 526

CMPLX not

b0305 rclR DNA-binding transcriptional activator RclR

b0306 ykgE putative lactate utilization oxidoreductaseYkgE

//

>NGHB#295 10

CMPLX not

b0306 ykgE putative lactate utilization oxidoreductaseYkgE

b0307 ykgF putative amino acid dehydrogenase withNAD(P)-binding domain and ferridoxin-like domain

//

>NGHB#296 -8

CMPLX not

b0307 ykgF putative amino acid dehydrogenase withNAD(P)-binding domain and ferridoxin-like domain

b0308 ykgG DUF162 domain-containing lactate utilizationprotein YkgG

//

>NGHB#298 212

CMPLX ---

b0311 betA choline dehydrogenase

b0310 ykgH uncharacterized protein YkgH

//

>NGHB#299 13

CMPLX not

b0312 betB betaine aldehyde dehydrogenase

b0311 betA choline dehydrogenase

//

>NGHB#300 13

CMPLX not

b0313 betI DNA-binding transcriptional repressor BetI

b0312 betB betaine aldehyde dehydrogenase

//

>NGHB#302 305

CMPLX not

b0314 betT choline:H(+) symporter

b4730 yahV protein YahV

//

>NGHB#303 494

CMPLX not

b4730 yahV protein YahV

b0315 pdeL DNA-binding transcriptional activator/c-di-GMPphosphodiesterase PdeL

//

>NGHB#305 91

CMPLX ---

b0317 yahC uncharacterized protein YahC

b0316 yahB putative LysR-type DNA-binding transcriptionalregulator YahB

//

>NGHB#307 39

CMPLX not

b0318 yahD ankyrin repeat-containing protein YahD

b0319 yahE DUF2877 domain-containing protein YahE

//

>NGHB#308 -11

CMPLX not

b0319 yahE DUF2877 domain-containing protein YahE

b0320 yahF putative acyl-CoA synthetase YahF

//

>NGHB#309 -1

CMPLX not

b0320 yahF putative acyl-CoA synthetase YahF

b0321 yahG DUF1116 domain-containing protein YahG

//

>NGHB#310 25

CMPLX ---

b0321 yahG DUF1116 domain-containing protein YahG

b0322 yahH putative uncharacterized protein YahH

//

>NGHB#311 75

CMPLX ---

b0322 yahH putative uncharacterized protein YahH

b0323 yahI carbamate kinase-like protein YahI

//

>NGHB#312 9

CMPLX not

b0323 yahI carbamate kinase-like protein YahI

b0324 yahJ putative deaminase with metallo-dependenthydrolase domain

//

>NGHB#313 376

CMPLX not

b0324 yahJ putative deaminase with metallo-dependenthydrolase domain

b0325 yahK aldehyde reductase, NADPH-dependent

//

>NGHB#314 242

CMPLX ---

b0325 yahK aldehyde reductase, NADPH-dependent

b0326 yahL uncharacterized protein YahL

//

>NGHB#315 412

CMPLX ---

b0326 yahL uncharacterized protein YahL

b0327 yahM uncharacterized protein YahM

//

>NGHB#320 439

CMPLX not

b0331 prpB 2-methylisocitrate lyase

b0333 prpC 2-methylcitrate synthase

//

>NGHB#321 33

CMPLX not

b0333 prpC 2-methylcitrate synthase

b0334 prpD 2-methylcitrate dehydratase

//

>NGHB#322 39

CMPLX not

b0334 prpD 2-methylcitrate dehydratase

b0335 prpE propionyl-CoA synthetase

//

>NGHB#323 329

CMPLX not

b0335 prpE propionyl-CoA synthetase

b0336 codB cytosine transporter

//

>NGHB#324 -11

CMPLX not

b0336 codB cytosine transporter

b0337 codA cytosine/isoguanine deaminase

//

>NGHB#327 30

CMPLX not

b0339 cynT carbonic anhydrase 1

b0340 cynS cyanase

//

>NGHB#328 32

CMPLX not

b0340 cynS cyanase

b0341 cynX cyanate transporter

//

>NGHB#330 65

CMPLX not

b0343 lacY lactose permease

b0342 lacA galactoside O-acetyltransferase

//

>NGHB#331 51

CMPLX not

b0344 lacZ beta-galactosidase

b0343 lacY lactose permease

//

>NGHB#332 122

CMPLX not

b0345 lacI DNA-binding transcriptional repressor LacI

b0344 lacZ beta-galactosidase

//

>NGHB#333 76

CMPLX not

b0346 mhpR DNA-binding transcriptional activator MhpR

b0345 lacI DNA-binding transcriptional repressor LacI

//

>NGHB#335 1

CMPLX not

b0347 mhpA 3-(3-hydroxyphenyl)propanoate hydroxylase

b0348 mhpB 3-carboxyethylcatechol 2,3-dioxygenase

//

>NGHB#336 17

CMPLX not

b0348 mhpB 3-carboxyethylcatechol 2,3-dioxygenase

b0349 mhpC 2-hydroxy-6-ketonona-2,4-dienedioate hydrolase

//

>NGHB#337 9

CMPLX not

b0349 mhpC 2-hydroxy-6-ketonona-2,4-dienedioate hydrolase

b0350 mhpD 2-hydroxypentadienoate hydratase

//

>NGHB#338 -4

CMPLX not

b0350 mhpD 2-hydroxypentadienoate hydratase

b0351 mhpF acetaldehyde dehydrogenase (acetylating) MhpF

//

>NGHB#339 -4

CMPLX not

b0351 mhpF acetaldehyde dehydrogenase (acetylating) MhpF

b0352 mhpE 4-hydroxy-2-oxovalerate aldolase

//

>NGHB#340 577

CMPLX not

b0352 mhpE 4-hydroxy-2-oxovalerate aldolase

b0353 mhpT 3-hydroxyphenylpropionate/3-hydroxycinnamate:H(+) symporter

//

>NGHB#341 101

CMPLX not

b0353 mhpT 3-hydroxyphenylpropionate/3-hydroxycinnamate:H(+) symporter

b0354 yaiL DUF2058 domain-containing protein YaiL

//

>NGHB#343 93

CMPLX not

b0356 frmA S-(hydroxymethyl)glutathione dehydrogenase

b0355 frmB S-formylglutathione hydrolase FrmB

//

>NGHB#344 34

CMPLX not

b0357 frmR DNA-binding transcriptional repressor FrmR

b0356 frmA S-(hydroxymethyl)glutathione dehydrogenase

//

>NGHB#345 187

CMPLX not

b0358 yaiO outer membrane protein YaiO

b0357 frmR DNA-binding transcriptional repressor FrmR

//

>NGHB#346 1

CMPLX not

b4579 yaiX putative acyltransferase, N-terminal fragment

b0358 yaiO outer membrane protein YaiO

//

>NGHB#347 -43

CMPLX tnp

b0360 insC1 IS2 element protein InsA

b0361 insD1 IS2 element protein

//

>NGHB#351 12

CMPLX yes

b0365 tauA taurine ABC transporter periplasmic bindingprotein

b0366 tauB taurine ABC transporter ATP binding subunit

//

>NGHB#352 -4

CMPLX yes

b0366 tauB taurine ABC transporter ATP binding subunit

b0367 tauC taurine ABC transporter membrane subunit

//

>NGHB#353 -4

CMPLX not

b0367 tauC taurine ABC transporter membrane subunit

b0368 tauD taurine dioxygenase

//

>NGHB#356 90

CMPLX not

b0372 insF2 IS3 element protein InsF

b4767 ytiB protein YtiB

//

>NGHB#357 -4

CMPLX tnp

b0373 insE2 IS3 element protein InsE

b0372 insF2 IS3 element protein InsF

//

>NGHB#360 12

CMPLX not

b0377 sbmA peptide antibiotic/peptide nucleic acidtransporter

b0378 yaiW surface-exposed outer membrane lipoprotein

//

>NGHB#365 100

CMPLX not

b0382 iraP anti-adaptor protein for sigma(S) stabilization

b0383 phoA alkaline phosphatase

//

>NGHB#366 118

CMPLX not

b0383 phoA alkaline phosphatase

b0384 psiF PsiF family protein

//

>NGHB#367 101

CMPLX not

b0384 psiF PsiF family protein

b0385 dgcC diguanylate cyclase DgcC

//

>NGHB#370 182

CMPLX not

b0387 yaiI DUF188 domain-containing protein YaiI

b0388 aroL shikimate kinase 2

//

>NGHB#371 49

CMPLX not

b0388 aroL shikimate kinase 2

b0389 yaiA protein YaiA

//

>NGHB#372 257

CMPLX not

b0389 yaiA protein YaiA

b0390 aroM protein AroM

//

>NGHB#373 71

CMPLX not

b0390 aroM protein AroM

b0391 ppnP nucleoside phosphorylase PpnP

//

>NGHB#374 485

CMPLX not

b0391 ppnP nucleoside phosphorylase PpnP

b4732 ykiD protein YkiD

//

>NGHB#378 125

CMPLX not

b0397 sbcC ATP dependent, structure specific DNA nuclease -SbcC subunit

b0396 araJ putative transport protein AraJ

//

>NGHB#379 -4

CMPLX yes

b0398 sbcD ATP dependent, structure specific DNA nuclease -SbcD subunit

b0397 sbcC ATP dependent, structure specific DNA nuclease -SbcC subunit

//

>NGHB#381 57

CMPLX not

b0399 phoB DNA-binding transcriptional dual regulator PhoB

b0400 phoR sensor histidine kinase PhoR

//

>NGHB#382 406

CMPLX not

b0400 phoR sensor histidine kinase PhoR

b0401 brnQ branched chain amino acid transporter BrnQ

//

>NGHB#383 75

CMPLX not

b0401 brnQ branched chain amino acid transporter BrnQ

b0402 proY putative transporter ProY

//

>NGHB#384 158

CMPLX not

b0402 proY putative transporter ProY

b0403 malZ maltodextrin glucosidase

//

>NGHB#387 55

CMPLX not

b0405 queA tRNA preQ1(34) S-adenosylmethionineribosyltransferase-isomerase

b0406 tgt tRNA-guanine transglycosylase

//

>NGHB#388 22

CMPLX not

b0406 tgt tRNA-guanine transglycosylase

b0407 yajC Sec translocon accessory complex subunit YajC

//

>NGHB#389 27

CMPLX yes

b0407 yajC Sec translocon accessory complex subunit YajC

b0408 secD Sec translocon accessory complex subunit SecD

//

>NGHB#390 10

CMPLX yes

b0408 secD Sec translocon accessory complex subunit SecD

b0409 secF Sec translocon accessory complex subunit SecF

//

>NGHB#391 128

CMPLX not

b0409 secF Sec translocon accessory complex subunit SecF

b0410 yajD HNH nuclease family protein YajD

//

>NGHB#393 298

CMPLX not

b0412 yajI putative lipoprotein YajI

b0411 tsx nucleoside-specific channel-forming protein Tsx

//

>NGHB#395 3

CMPLX not

b0413 nrdR NrdR transcriptional repressor

b0414 ribD fused diaminohydroxyphosphoribosylaminopyrimidine deaminase/5-amino-6-(5-phosphoribosylamino)uracilreductase

//

>NGHB#396 88

CMPLX not

b0414 ribD fused diaminohydroxyphosphoribosylaminopyrimidine deaminase/5-amino-6-(5-phosphoribosylamino)uracilreductase

b0415 ribE 6,7-dimethyl-8-ribityllumazine synthase

//

>NGHB#397 19

CMPLX not

b0415 ribE 6,7-dimethyl-8-ribityllumazine synthase

b0416 nusB transcription antitermination protein NusB

//

>NGHB#398 77

CMPLX not

b0416 nusB transcription antitermination protein NusB

b0417 thiL thiamine monophosphate kinase

//

>NGHB#399 -23

CMPLX not

b0417 thiL thiamine monophosphate kinase

b0418 pgpA phosphatidylglycerophosphatase A

//

>NGHB#401 179

CMPLX not

b0420 dxs 1-deoxy-D-xylulose-5-phosphate synthase

b0419 yajO 1-deoxyxylulose-5-phosphate synthase YajO

//

>NGHB#402 24

CMPLX not

b0421 ispA geranyl diphosphate/farnesyl diphosphatesynthase

b0420 dxs 1-deoxy-D-xylulose-5-phosphate synthase

//

>NGHB#403 -1

CMPLX not

b0422 xseB exodeoxyribonuclease VII subunit XseB

b0421 ispA geranyl diphosphate/farnesyl diphosphatesynthase

//

>NGHB#406 -38

CMPLX not

b0425 panE 2-dehydropantoate 2-reductase

b0424 yajL protein/nucleic acid deglycase 3

//

>NGHB#409 148

CMPLX not

b0428 cyoE heme O synthase

b0427 yajR putative transport protein YajR

//

>NGHB#410 11

CMPLX not

b0429 cyoD cytochrome bo3 ubiquinol oxidase subunit 4

b0428 cyoE heme O synthase

//

>NGHB#411 -1

CMPLX yes

b0430 cyoC cytochrome bo3 ubiquinol oxidase subunit 3

b0429 cyoD cytochrome bo3 ubiquinol oxidase subunit 4

//

>NGHB#412 -11

CMPLX yes

b0431 cyoB cytochrome bo3 ubiquinol oxidase subunit 1

b0430 cyoC cytochrome bo3 ubiquinol oxidase subunit 3

//

>NGHB#413 21

CMPLX yes

b0432 cyoA cytochrome bo3 ubiquinol oxidase subunit 2

b0431 cyoB cytochrome bo3 ubiquinol oxidase subunit 1

//

>NGHB#414 459

CMPLX not

b0433 ampG muropeptide:H(+) symporter

b0432 cyoA cytochrome bo3 ubiquinol oxidase subunit 2

//

>NGHB#415 43

CMPLX not

b0434 yajG putative lipoprotein YajG

b0433 ampG muropeptide:H(+) symporter

//

>NGHB#417 343

CMPLX not

b0435 bolA DNA-binding transcriptional dual regulator BolA

b0436 tig trigger factor

//

>NGHB#418 245

CMPLX not

b0436 tig trigger factor

b0437 clpP ATP-dependent Clp protease proteolytic subunit

//

>NGHB#419 125

CMPLX yes

b0437 clpP ATP-dependent Clp protease proteolytic subunit

b0438 clpX ATP-dependent Clp protease ATP-binding subunitClpX

//

>NGHB#420 187

CMPLX not

b0438 clpX ATP-dependent Clp protease ATP-binding subunitClpX

b0439 lon Lon protease

//

>NGHB#421 208

CMPLX not

b0439 lon Lon protease

b0440 hupB DNA-binding protein HU-beta

//

>NGHB#422 191

CMPLX not

b0440 hupB DNA-binding protein HU-beta

b0441 ppiD periplasmic folding chaperone

//

>NGHB#423 150

CMPLX not

b0441 ppiD periplasmic folding chaperone

b0442 ybaV helix-hairpin-helix 3 family protein

//

>NGHB#424 93

CMPLX not

b0442 ybaV helix-hairpin-helix 3 family protein

b0443 fadM long-chain acyl-CoA thioesterase FadM

//

>NGHB#426 64

CMPLX ---

b0445 ybaE uncharacterized protein YbaE

b0444 queC 7-cyano-7-deazaguanine synthase

//

>NGHB#428 152

CMPLX not

b0446 cof HMP-PP phosphatase

b0447 decR DNA-binding transcriptional activator DecR

//

>NGHB#429 29

CMPLX not

b0447 decR DNA-binding transcriptional activator DecR

b0448 mdlA ABC transporter family protein MdlA

//

>NGHB#430 -8

CMPLX yes

b0448 mdlA ABC transporter family protein MdlA

b0449 mdlB ABC transporter family protein MdlB

//

>NGHB#431 180

CMPLX not

b0449 mdlB ABC transporter family protein MdlB

b0450 glnK nitrogen regulator GlnK

//

>NGHB#432 29

CMPLX not

b0450 glnK nitrogen regulator GlnK

b0451 amtB ammonia/ammonium transporter

//

>NGHB#438 163

CMPLX not

b0458 ylaC putative inner membrane protein

b0457 pdeB c-di-GMP phosphodiesterase PdeB

//

>NGHB#439 115

CMPLX not

b0459 maa maltose O-acetyltransferase

b0458 ylaC putative inner membrane protein

//

>NGHB#440 171

CMPLX not

b0460 hha hemolysin expression-modulating protein Hha

b0459 maa maltose O-acetyltransferase

//

>NGHB#441 25

CMPLX not

b0461 tomB Hha toxicity modulator TomB

b0460 hha hemolysin expression-modulating protein Hha

//

>NGHB#442 545

CMPLX not

b0462 acrB multidrug efflux pump RND permease AcrB

b0461 tomB Hha toxicity modulator TomB

//

>NGHB#443 22

CMPLX yes

b0463 acrA multidrug efflux pump membrane fusionlipoprotein AcrA

b0462 acrB multidrug efflux pump RND permease AcrB

//

>NGHB#445 127

CMPLX not

b0464 acrR DNA-binding transcriptional repressor AcrR

b0465 mscK potassium dependent, small conductancemechanosensitive channel

//

>NGHB#447 13

CMPLX not

b0467 priC primosomal replication protein N''

b0466 ybaM DUF2496 domain-containing protein YbaM

//

>NGHB#449 152

CMPLX not

b0468 ybaN DUF454 domain-containing inner membrane proteinYbaN

b0469 apt adenine phosphoribosyltransferase

//

>NGHB#450 128

CMPLX not

b0469 apt adenine phosphoribosyltransferase

b0470 dnaX DNA polymerase III subunit gamma

//

>NGHB#451 689

CMPLX not

b0470 dnaX DNA polymerase III subunit gamma

b0471 ybaB putative nucleoid-associated protein YbaB

//

>NGHB#452 -1

CMPLX not

b0471 ybaB putative nucleoid-associated protein YbaB

b0472 recR DNA repair protein RecR

//

>NGHB#453 109

CMPLX not

b0472 recR DNA repair protein RecR

b0473 htpG chaperone protein HtpG

//

>NGHB#454 180

CMPLX not

b0473 htpG chaperone protein HtpG

b0474 adk adenylate kinase

//

>NGHB#455 235

CMPLX not

b0474 adk adenylate kinase

b0475 hemH ferrochelatase

//

>NGHB#459 237

CMPLX not

b0479 fsr fosmidomycin efflux pump

b0478 ybaL putative transporter YbaL

//

>NGHB#462 203

CMPLX not

b0482 ybaP TraB family protein YbaP

b0481 ybaK Cys-tRNA(Pro) and Cys-tRNA(Cys) deacylase

//

>NGHB#466 2

CMPLX not

b0485 glsA glutaminase 1

b0486 ybaT putative transporter YbaT

//

>NGHB#467 124

CMPLX not

b0486 ybaT putative transporter YbaT

b0487 cueR DNA-binding transcriptional dual regulator CueR

//

>NGHB#469 -4

CMPLX not

b0489 qmcA PHB domain-containing protein QmcA

b0488 ybbJ NfeD-like family protein YbbJ

//

>NGHB#471 -14

CMPLX yes

b0490 fetA putative iron ABC exporter ATP-binding subunitFetA

b0491 fetB putative iron ABC exporter membrane subunitFetB

//

>NGHB#473 60

CMPLX not

b0493 ybbO NADP(+)-dependent aldehyde reductase YbbO

b0492 cnoX chaperedoxin

//

>NGHB#474 -11

CMPLX not

b0494 tesA multifunctional acyl-CoA thioesterase I andprotease I and lysophospholipase L1

b0493 ybbO NADP(+)-dependent aldehyde reductase YbbO

//

>NGHB#476 -4

CMPLX yes

b0495 ybbA putative ABC transporter ATP-binding proteinYbbA

b0496 ybbP putative ABC transporter membrane subunit YbbP

//

>NGHB#477 430

CMPLX not

b0496 ybbP putative ABC transporter membrane subunit YbbP

b0497 rhsD protein RhsD

//

>NGHB#478 39

CMPLX not

b0497 rhsD protein RhsD

b0498 ybbC PF15631 family protein YbbC

//

>NGHB#480 115

CMPLX not

b0503 selU tRNA 2-selenouridine synthase

b0502 ylbG putative DNA-binding transcriptional regulatorYlbG

//

>NGHB#481 68

CMPLX not

b0504 allS DNA-binding transcriptional activator AllS

b0503 selU tRNA 2-selenouridine synthase

//

>NGHB#483 77

CMPLX not

b0505 allA ureidoglycolate lyase

b0506 allR DNA-binding transcriptional repressor AllR

//

>NGHB#484 89

CMPLX not

b0506 allR DNA-binding transcriptional repressor AllR

b0507 gcl glyoxylate carboligase

//

>NGHB#485 12

CMPLX not

b0507 gcl glyoxylate carboligase

b0508 hyi hydroxypyruvate isomerase

//

>NGHB#486 99

CMPLX not

b0508 hyi hydroxypyruvate isomerase

b0509 glxR tartronate semialdehyde reductase 2

//

>NGHB#487 168

CMPLX not

b0509 glxR tartronate semialdehyde reductase 2

b0511 ybbW putative allantoin transporter

//

>NGHB#488 59

CMPLX not

b0511 ybbW putative allantoin transporter

b0512 allB allantoinase

//

>NGHB#489 56

CMPLX not

b0512 allB allantoinase

b0513 ybbY putative purine transporter

//

>NGHB#490 21

CMPLX not

b0513 ybbY putative purine transporter

b0514 glxK glycerate 2-kinase 2

//

>NGHB#492 10

CMPLX not

b0516 allC allantoate amidohydrolase

b0515 allE (S)-ureidoglycine aminohydrolase

//

>NGHB#493 21

CMPLX not

b0517 allD ureidoglycolate dehydrogenase

b0516 allC allantoate amidohydrolase

//

>NGHB#495 9

CMPLX not

b0518 fdrA putative acyl-CoA synthetase FdrA

b4572 ylbE DUF1116 domain-containing protein YlbE

//

>NGHB#496 10

CMPLX not

b4572 ylbE DUF1116 domain-containing protein YlbE

b0520 ylbF DUF2877 domain-containing protein YlbF

//

>NGHB#497 -4

CMPLX not

b0520 ylbF DUF2877 domain-containing protein YlbF

b0521 ybcF putative carbamate kinase

//

>NGHB#499 -4

CMPLX not

b0523 purE N(5)-carboxyaminoimidazole ribonucleotidemutase

b0522 purK 5-(carboxyamino)imidazole ribonucleotidesynthase

//

>NGHB#500 117

CMPLX not

b0524 lpxH UDP-2,3-diacylglucosamine diphosphatase

b0523 purE N(5)-carboxyaminoimidazole ribonucleotidemutase

//

>NGHB#501 2

CMPLX not

b0525 ppiB peptidyl-prolyl cis-trans isomerase B

b0524 lpxH UDP-2,3-diacylglucosamine diphosphatase

//

>NGHB#504 107

CMPLX not

b0528 ybcJ putative RNA-binding protein YbcJ

b0527 ybcI PF04307 family inner membrane protein YbcI

//

>NGHB#505 1

CMPLX not

b0529 folD bifunctional methylenetetrahydrofolatedehydrogenase/methenyltetrahydrofolate cyclohydrolase

b0528 ybcJ putative RNA-binding protein YbcJ

//

>NGHB#507 219

CMPLX not

b0530 sfmA putative fimbrial protein SfmA

b0531 sfmC putative fimbrial chaperone SfmC

//

>NGHB#508 30

CMPLX not

b0531 sfmC putative fimbrial chaperone SfmC

b0532 sfmD putative fimbrial usher protein SfmD

//

>NGHB#509 35

CMPLX not

b0532 sfmD putative fimbrial usher protein SfmD

b0533 sfmH putative fimbrial adhesin protein SfmH

//

>NGHB#510 10

CMPLX not

b0533 sfmH putative fimbrial adhesin protein SfmH

b0534 sfmF putative fimbrial protein SfmF

//

>NGHB#512 334

CMPLX not

b0537 intD DLP12 prophage; putative integrase

b0535 fimZ putative LuxR family transcriptional regulatorFimZ

//

>NGHB#514 -4

CMPLX tnp

b0540 insE3 DLP12 prophage; IS3 element protein InsE

b0541 insF3 DLP12 prophage; IS3 element protein InsF

//

>NGHB#515 47

CMPLX not

b0543 emrE DLP12 prophage; multidrug/betaine/choline effluxtransporter EmrE

b4733 ylcJ DLP12 prophage; protein YlcJ

//

>NGHB#516 57

CMPLX not

b4733 ylcJ DLP12 prophage; protein YlcJ

b0544 ybcK DLP12 prophage; putative recombinase YbcK

//

>NGHB#517 464

CMPLX not

b0544 ybcK DLP12 prophage; putative recombinase YbcK

b0545 ybcL DLP12 prophage; periplasmic protein YbcL

//

>NGHB#518 9

CMPLX not

b0545 ybcL DLP12 prophage; periplasmic protein YbcL

b0546 ybcM DLP12 prophage; putative DNA-bindingtranscriptional regulator

//

>NGHB#519 116

CMPLX ---

b0546 ybcM DLP12 prophage; putative DNA-bindingtranscriptional regulator

b4588 ylcH DLP12 prophage; uncharacterized protein YlcH

//

>NGHB#520 -4

CMPLX ---

b4588 ylcH DLP12 prophage; uncharacterized protein YlcH

b0547 ybcN DLP12 prophage; DNA base-flipping protein

//

>NGHB#521 -1

CMPLX not

b0547 ybcN DLP12 prophage; DNA base-flipping protein

b0548 ninE DLP12 prophage; NinE family prophage protein

//

>NGHB#522 -8

CMPLX not

b0548 ninE DLP12 prophage; NinE family prophage protein

b0549 ybcO DLP12 prophage; putative nuclease YbcO

//

>NGHB#523 -4

CMPLX not

b0549 ybcO DLP12 prophage; putative nuclease YbcO

b0550 rusA DLP12 prophage; crossover junctionendodeoxyribonuclease RusA

//

>NGHB#524 -4

CMPLX ---

b0550 rusA DLP12 prophage; crossover junctionendodeoxyribonuclease RusA

b4509 ylcG DLP12 prophage; uncharacterized protein YlcG

//

>NGHB#525 85

CMPLX ---

b4509 ylcG DLP12 prophage; uncharacterized protein YlcG

b0551 quuD DLP12 prophage; prophage antitermination proteinQ homolog QuuD

//

>NGHB#527 -1

CMPLX not

b0554 essD DLP12 prophage; putative phage lysis protein

b0555 rrrD DLP12 prophage; lysozyme

//

>NGHB#528 -4

CMPLX not

b0555 rrrD DLP12 prophage; lysozyme

b0556 rzpD DLP12 prophage; putative prophage endopeptidaseRzpD

//

>NGHB#529 -242

CMPLX not

b0556 rzpD DLP12 prophage; putative prophage endopeptidaseRzpD

b4510 rzoD DLP12 prophage; putative prophage lysislipoprotein RzoD

//

>NGHB#531 290

CMPLX not

b0558 ybcV DLP12 prophage; DUF1398 domain-containingprotein YbcV

b0557 borD DLP12 prophage; prophage lipoprotein BorD

//

>NGHB#535 154

CMPLX not

b0560 nohD DLP12 prophage; putative DNA-packaging proteinNohD

b0561 tfaD DLP12 prophage; putative tail fiber assemblyprotein TfaD

//

>NGHB#536 777

CMPLX not

b0561 tfaD DLP12 prophage; putative tail fiber assemblyprotein TfaD

b0563 tfaX DLP12 prophage; protein TfaX

//

>NGHB#537 620

CMPLX not

b0563 tfaX DLP12 prophage; protein TfaX

b0564 appY DLP12 prophage; DNA-binding transcriptionalactivator AppY

//

>NGHB#539 513

CMPLX not

b0566 envY DNA-binding transcriptional activator EnvY

b0565 ompT DLP12 prophage; protease 7

//

>NGHB#540 182

CMPLX not

b0567 ybcH DUF4434 domain-containing protein YbcH

b0566 envY DNA-binding transcriptional activator EnvY

//

>NGHB#541 0

CMPLX not

b0568 nfrA bacteriophage N4 outer membrane receptor

b0567 ybcH DUF4434 domain-containing protein YbcH

//

>NGHB#542 -14

CMPLX not

b0569 nfrB bacteriophage N4 adsorption protein

b0568 nfrA bacteriophage N4 outer membrane receptor

//

>NGHB#543 149

CMPLX not

b0570 cusS sensor histidine kinase CusS

b0569 nfrB bacteriophage N4 adsorption protein

//

>NGHB#544 -11

CMPLX not

b0571 cusR DNA-binding transcriptional activator CusR

b0570 cusS sensor histidine kinase CusS

//

>NGHB#546 157

CMPLX yes

b0572 cusC copper/silver export system outer membranechannel

b0573 cusF copper/silver export system periplasmic bindingprotein

//

>NGHB#547 15

CMPLX yes

b0573 cusF copper/silver export system periplasmic bindingprotein

b0574 cusB copper/silver export system membrane fusionprotein

//

>NGHB#548 11

CMPLX yes

b0574 cusB copper/silver export system membrane fusionprotein

b0575 cusA copper/silver export system RND permease

//

>NGHB#549 101

CMPLX not

b0575 cusA copper/silver export system RND permease

b0576 pheP phenylalanine:H(+) symporter PheP

//

>NGHB#551 107

CMPLX not

b0578 nfsB NAD(P)H nitroreductase NfsB

b0577 ybdG miniconductance mechanosensitive channel YbdG

//

>NGHB#552 93

CMPLX not

b0579 ybdF PF04237 family protein YbdF

b0578 nfsB NAD(P)H nitroreductase NfsB

//

>NGHB#553 64

CMPLX not

b0580 ybdJ DUF1158 domain-containing protein YbdJ

b0579 ybdF PF04237 family protein YbdF

//

>NGHB#554 65

CMPLX not

b0581 ybdK carboxylate-amine ligase

b0580 ybdJ DUF1158 domain-containing protein YbdJ

//

>NGHB#556 76

CMPLX not

b4415 hokE protein HokE

b0582 insL2 IS186/IS421 transposase

//

>NGHB#558 174

CMPLX not

b0584 fepA ferric enterobactin outer membrane transporter

b0583 entD phosphopantetheinyl transferase EntD

//

>NGHB#560 2

CMPLX not

b0585 fes ferric enterobactin esterase

b4511 ybdZ enterobactin biosynthesis protein YbdZ

//

>NGHB#561 -4

CMPLX not

b4511 ybdZ enterobactin biosynthesis protein YbdZ

b0586 entF apo-serine activating enzyme

//

>NGHB#562 215

CMPLX not

b0586 entF apo-serine activating enzyme

b0587 fepE polysaccharide co-polymerase family proteinFepE

//

>NGHB#564 -4

CMPLX yes

b0589 fepG ferric enterobactin ABC transporter membranesubunit FepG

b0588 fepC ferric enterobactin ABC transporter ATP bindingsubunit

//

>NGHB#565 -4

CMPLX yes

b0590 fepD ferric enterobactin ABC transporter membranesubunit FebD

b0589 fepG ferric enterobactin ABC transporter membranesubunit FepG

//

>NGHB#569 9

CMPLX not

b0593 entC isochorismate synthase EntC

b0594 entE 2,3-dihydroxybenzoate-AMP ligase

//

>NGHB#570 13

CMPLX not

b0594 entE 2,3-dihydroxybenzoate-AMP ligase

b0595 entB enterobactin synthase component B

//

>NGHB#571 -1

CMPLX not

b0595 entB enterobactin synthase component B

b0596 entA 2,3-dihydro-2,3-dihydroxybenzoate dehydrogenase

//

>NGHB#572 2

CMPLX not

b0596 entA 2,3-dihydro-2,3-dihydroxybenzoate dehydrogenase

b0597 entH proofreading thioesterase in enterobactinbiosynthesis

//

>NGHB#573 180

CMPLX not

b0597 entH proofreading thioesterase in enterobactinbiosynthesis

b0598 cstA carbon starvation protein A

//

>NGHB#574 182

CMPLX not

b0598 cstA carbon starvation protein A

b4512 ybdD PF04328 family protein YbdD

//

>NGHB#578 -28

CMPLX not

b0602 ybdN putative PAPS reductase/DUF3440domain-containing protein YbdN

b0601 ybdM ParB-like nuclease domain-containing proteinYbdM

//

>NGHB#579 146

CMPLX not

b0603 ybdO putative LysR family DNA-binding transcriptionalregulator YbdO

b0602 ybdN putative PAPS reductase/DUF3440domain-containing protein YbdN

//

>NGHB#580 208

CMPLX not

b0604 dsbG protein sulfenic acid reductase and chaperoneDsbG

b0603 ybdO putative LysR family DNA-binding transcriptionalregulator YbdO

//

>NGHB#582 244

CMPLX yes

b0605 ahpC alkyl hydroperoxide reductase, AhpC component

b0606 ahpF alkyl hydroperoxide reductase, AhpF component

//

>NGHB#586 137

CMPLX not

b0610 rnk nucleoside diphosphate kinase regulator

b4734 yldA protein YldA

//

>NGHB#587 229

CMPLX not

b0611 rna RNase I

b0610 rnk nucleoside diphosphate kinase regulator

//

>NGHB#588 113

CMPLX not

b0612 citT citrate:succinate antiporter

b0611 rna RNase I

//

>NGHB#589 50

CMPLX not

b0613 citG triphosphoribosyl-dephospho-CoA synthase

b0612 citT citrate:succinate antiporter

//

>NGHB#590 -26

CMPLX not

b0614 citX apo-citrate lyase phosphoribosyl-dephospho-CoAtransferase

b0613 citG triphosphoribosyl-dephospho-CoA synthase

//

>NGHB#591 3

CMPLX not

b0615 citF citrate lyase alpha subunit

b0614 citX apo-citrate lyase phosphoribosyl-dephospho-CoAtransferase

//

>NGHB#592 10

CMPLX yes

b0616 citE citrate lyase beta subunit

b0615 citF citrate lyase alpha subunit

//

>NGHB#593 -4

CMPLX yes

b0617 citD citrate lyase acyl carrier protein

b0616 citE citrate lyase beta subunit

//

>NGHB#594 14

CMPLX yes

b0618 citC citrate lyase synthetase

b0617 citD citrate lyase acyl carrier protein

//

>NGHB#596 -32

CMPLX not

b0619 dpiB sensor histidine kinase DpiB

b0620 dpiA DNA-binding transcriptional dual regulator DpiA

//

>NGHB#599 174

CMPLX not

b0622 pagP Lipid A palmitoyltransferase

b0623 cspE transcription antiterminator and regulator ofRNA stability

//

>NGHB#603 208

CMPLX not

b0629 ybeF putative LysR-type DNA-binding transcriptionalregulator YbeF

b0628 lipA lipoyl synthase

//

>NGHB#604 258

CMPLX not

b0630 lipB lipoyl(octanoyl) transferase

b0629 ybeF putative LysR-type DNA-binding transcriptionalregulator YbeF

//

>NGHB#605 100

CMPLX not

b0631 ybeD DUF493 domain-containing protein YbeD

b0630 lipB lipoyl(octanoyl) transferase

//

>NGHB#606 109

CMPLX not

b0632 dacA D-alanyl-D-alanine carboxypeptidase DacA

b0631 ybeD DUF493 domain-containing protein YbeD

//

>NGHB#607 138

CMPLX not

b0633 rlpA rare lipoprotein RlpA

b0632 dacA D-alanyl-D-alanine carboxypeptidase DacA

//

>NGHB#608 10

CMPLX not

b0634 mrdB SEDS family protein MrdB

b0633 rlpA rare lipoprotein RlpA

//

>NGHB#609 2

CMPLX not

b0635 mrdA peptidoglycan DD-transpeptidase MrdA

b0634 mrdB SEDS family protein MrdB

//

>NGHB#610 30

CMPLX not

b0636 rlmH 23S rRNA m(3)psi1915 methyltransferase

b0635 mrdA peptidoglycan DD-transpeptidase MrdA

//

>NGHB#611 3

CMPLX not

b0637 rsfS ribosomal silencing factor RsfS

b0636 rlmH 23S rRNA m(3)psi1915 methyltransferase

//

>NGHB#612 259

CMPLX not

b0638 cobC putative adenosylcobalaminphosphatase/alpha-ribazole phosphatase

b0637 rsfS ribosomal silencing factor RsfS

//

>NGHB#613 23

CMPLX not

b0639 nadD nicotinate-mononucleotide adenylyltransferase

b0638 cobC putative adenosylcobalaminphosphatase/alpha-ribazole phosphatase

//

>NGHB#614 1

CMPLX not

b0640 holA DNA polymerase III subunit delta

b0639 nadD nicotinate-mononucleotide adenylyltransferase

//

>NGHB#615 -1

CMPLX not

b0641 lptE lipopolysaccharide assembly protein LptE

b0640 holA DNA polymerase III subunit delta

//

>NGHB#616 14

CMPLX not

b0642 leuS leucine--tRNA ligase

b0641 lptE lipopolysaccharide assembly protein LptE

//

>NGHB#620 -4

CMPLX not

b0645 ybeR DUF1266 domain-containing protein YbeR

b0646 djlB J domain-containing protein DjlB

//

>NGHB#623 -4

CMPLX not

b0648 ybeU DUF1266 domain-containing protein YbeU

b0649 djlC co-chaperone DjlC

//

>NGHB#625 83

CMPLX not

b0651 rihA pyrimidine-specific ribonucleoside hydrolaseRihA

b0650 hscC chaperone protein HscC

//

>NGHB#626 117

CMPLX not

b0652 gltL glutamate/aspartate ABC transporter ATP bindingsubunit

b0651 rihA pyrimidine-specific ribonucleoside hydrolaseRihA

//

>NGHB#627 -1

CMPLX yes

b0653 gltK glutamate/aspartate ABC transporter membranesubunit GltK

b0652 gltL glutamate/aspartate ABC transporter ATP bindingsubunit

//

>NGHB#628 -1

CMPLX yes

b0654 gltJ glutamate/aspartate ABC transporter membranesubunit GltJ

b0653 gltK glutamate/aspartate ABC transporter membranesubunit GltK

//

>NGHB#629 169

CMPLX yes

b0655 gltI glutamate/aspartate ABC transporter periplasmicbinding protein

b0654 gltJ glutamate/aspartate ABC transporter membranesubunit GltJ

//

>NGHB#630 249

CMPLX not

b0656 insH3 IS5 transposase and trans-activator

b0655 gltI glutamate/aspartate ABC transporter periplasmicbinding protein

//

>NGHB#631 365

CMPLX not

b0657 lnt apolipoprotein N-acyltransferase

b0656 insH3 IS5 transposase and trans-activator

//

>NGHB#632 24

CMPLX not

b0658 ybeX CorC-HlyC family protein YbeX

b0657 lnt apolipoprotein N-acyltransferase

//

>NGHB#633 89

CMPLX not

b0659 ybeY endoribonuclease YbeY

b0658 ybeX CorC-HlyC family protein YbeX

//

>NGHB#634 -4

CMPLX not

b0660 ybeZ PhoH-like protein

b0659 ybeY endoribonuclease YbeY

//

>NGHB#635 152

CMPLX not

b0661 miaB isopentenyl-adenosine A37 tRNA methylthiolase

b0660 ybeZ PhoH-like protein

//

>NGHB#638 396

CMPLX not

b0675 umpH UMP phosphatase

b0674 asnB asparagine synthetase B

//

>NGHB#639 47

CMPLX not

b0676 nagC DNA-binding transcriptional dual regulator NagC

b0675 umpH UMP phosphatase

//

>NGHB#640 8

CMPLX not

b0677 nagA N-acetylglucosamine-6-phosphate deacetylase

b0676 nagC DNA-binding transcriptional dual regulator NagC

//

>NGHB#641 59

CMPLX not

b0678 nagB glucosamine-6-phosphate deaminase

b0677 nagA N-acetylglucosamine-6-phosphate deacetylase

//

>NGHB#643 202

CMPLX not

b0679 nagE N-acetylglucosamine-specific PTS enzyme IIABCcomponent

b0680 glnS glutamine--tRNA ligase

//

>NGHB#644 576

CMPLX not

b0680 glnS glutamine--tRNA ligase

b0681 chiP chitobiose outer membrane channel

//

>NGHB#645 49

CMPLX not

b0681 chiP chitobiose outer membrane channel

b0682 chiQ lipoprotein ChiQ

//

>NGHB#647 -8

CMPLX not

b4637 uof RyhB-regulated fur leader peptide

b0683 fur DNA-binding transcriptional dual regulator Fur

//

>NGHB#648 209

CMPLX not

b0684 fldA flavodoxin 1

b4637 uof RyhB-regulated fur leader peptide

//

>NGHB#649 139

CMPLX not

b0685 ybfE ribbon-helix-helix domain-containing proteinYbfE

b0684 fldA flavodoxin 1

//

>NGHB#650 139

CMPLX not

b0686 ybfF esterase

b0685 ybfE ribbon-helix-helix domain-containing proteinYbfE

//

>NGHB#652 25

CMPLX not

b0687 seqA negative modulator of initiation of replication

b0688 pgm phosphoglucomutase

//

>NGHB#653 213

CMPLX not

b0688 pgm phosphoglucomutase

b0689 ybfP lipoprotein YbfP

//

>NGHB#655 -4

CMPLX not

b0693 speF inducible ornithine decarboxylase

b0692 potE putrescine transporter PotE

//

>NGHB#656 257

CMPLX not

b4803 speFL leader peptide SpeFL

b0693 speF inducible ornithine decarboxylase

//

>NGHB#657 233

CMPLX not

b0694 kdpE DNA-binding transcriptional activator KdpE

b4803 speFL leader peptide SpeFL

//

>NGHB#658 -4

CMPLX not

b0695 kdpD sensor histidine kinase KdpD

b0694 kdpE DNA-binding transcriptional activator KdpE

//

>NGHB#659 -8

CMPLX not

b0696 kdpC K(+) transporting P-type ATPase subunit KdpC

b0695 kdpD sensor histidine kinase KdpD

//

>NGHB#660 8

CMPLX yes

b0697 kdpB K(+) transporting P-type ATPase subunit KdpB

b0696 kdpC K(+) transporting P-type ATPase subunit KdpC

//

>NGHB#661 22

CMPLX yes

b0698 kdpA K(+) transporting P-type ATPase subunit KdpA

b0697 kdpB K(+) transporting P-type ATPase subunit KdpB

//

>NGHB#662 -1

CMPLX yes

b4513 kdpF K(+) transporting P-type ATPase subunit KdpF

b0698 kdpA K(+) transporting P-type ATPase subunit KdpA

//

>NGHB#664 242

CMPLX not

b0699 ybfA DUF2517 domain-containing protein YbfA

b0700 rhsC rhs element protein RhsC

//

>NGHB#665 -1

CMPLX ---

b0700 rhsC rhs element protein RhsC

b0702 ybfB uncharacterized protein YbfB

//

>NGHB#666 1547

CMPLX ---

b0702 ybfB uncharacterized protein YbfB

b0704 ybfC uncharacterized protein YbfC

//

>NGHB#667 225

CMPLX ---

b0704 ybfC uncharacterized protein YbfC

b4514 ybfQ inactive transposase YbfQ

//

>NGHB#668 1392

CMPLX not

b4514 ybfQ inactive transposase YbfQ

b0706 ybfD H repeat-associated putative transposase YbfD

//

>NGHB#669 147

CMPLX not

b0706 ybfD H repeat-associated putative transposase YbfD

b0707 ybgA DUF1722 domain-containing protein YbgA

//

>NGHB#670 -4

CMPLX not

b0707 ybgA DUF1722 domain-containing protein YbgA

b0708 phr deoxyribodipyrimidine photolyase

//

>NGHB#673 22

CMPLX not

b0710 ybgI radiation resistance protein YbgI

b0711 pxpB 5-oxoprolinase component B

//

>NGHB#674 -7

CMPLX yes

b0711 pxpB 5-oxoprolinase component B

b0712 pxpC 5-oxoprolinase component C

//

>NGHB#675 -11

CMPLX yes

b0712 pxpC 5-oxoprolinase component C

b0713 pxpA 5-oxoprolinase component A

//

>NGHB#676 35

CMPLX not

b0713 pxpA 5-oxoprolinase component A

b0714 nei endonuclease VIII

//

>NGHB#678 151

CMPLX not

b0716 ybgO putative fimbrial protein YbgO

b0715 abrB putative regulator

//

>NGHB#679 -4

CMPLX not

b0717 ybgP putative fimbrial chaperone YbgP

b0716 ybgO putative fimbrial protein YbgO

//

>NGHB#680 14

CMPLX not

b0718 ybgQ putative fimbrial usher protein YbgQ

b0717 ybgP putative fimbrial chaperone YbgP

//

>NGHB#681 59

CMPLX not

b0719 ybgD putative fimbrial protein YbgD

b0718 ybgQ putative fimbrial usher protein YbgQ

//

>NGHB#682 389

CMPLX not

b0720 gltA citrate synthase

b0719 ybgD putative fimbrial protein YbgD

//

>NGHB#683 189

CMPLX not

b4768 ybgV protein YbgV

b0720 gltA citrate synthase

//

>NGHB#684 -17

CMPLX not

b4735 ybgU protein YbgU

b4768 ybgV protein YbgV

//

>NGHB#686 -7

CMPLX yes

b0721 sdhC succinate:quinone oxidoreductase, membraneprotein SdhC

b0722 sdhD succinate:quinone oxidoreductase, membraneprotein SdhD

//

>NGHB#687 -1

CMPLX yes

b0722 sdhD succinate:quinone oxidoreductase, membraneprotein SdhD

b0723 sdhA succinate:quinone oxidoreductase, FAD bindingprotein

//

>NGHB#688 15

CMPLX yes

b0723 sdhA succinate:quinone oxidoreductase, FAD bindingprotein

b0724 sdhB succinate:quinone oxidoreductase, iron-sulfurcluster binding protein

//

>NGHB#689 300

CMPLX not

b0724 sdhB succinate:quinone oxidoreductase, iron-sulfurcluster binding protein

b0726 sucA subunit of E1(0) component of 2-oxoglutaratedehydrogenase

//

>NGHB#690 14

CMPLX not

b0726 sucA subunit of E1(0) component of 2-oxoglutaratedehydrogenase

b0727 sucB dihydrolipoyltranssuccinylase

//

>NGHB#691 274

CMPLX not

b0727 sucB dihydrolipoyltranssuccinylase

b0728 sucC succinyl-CoA synthetase subunit beta

//

>NGHB#692 -1

CMPLX yes

b0728 sucC succinyl-CoA synthetase subunit beta

b0729 sucD succinyl-CoA synthetase subunit alpha

//

>NGHB#695 17

CMPLX not

b0731 mngA 2-O-alpha-mannosyl-D-glycerate specific PTSenzyme II

b0732 mngB alpha-mannosidase

//

>NGHB#696 846

CMPLX not

b0732 mngB alpha-mannosidase

b0733 cydA cytochrome bd-I ubiquinol oxidase subunit I

//

>NGHB#697 15

CMPLX yes

b0733 cydA cytochrome bd-I ubiquinol oxidase subunit I

b0734 cydB cytochrome bd-I ubiquinol oxidase subunit II

//

>NGHB#698 14

CMPLX yes

b0734 cydB cytochrome bd-I ubiquinol oxidase subunit II

b4515 cydX cytochrome bd-I ubiquinol oxidase accessorysubunit CydX

//

>NGHB#699 -1

CMPLX not

b4515 cydX cytochrome bd-I ubiquinol oxidase accessorysubunit CydX

b0735 ybgE PF09600 family protein YbgE

//

>NGHB#700 149

CMPLX not

b0735 ybgE PF09600 family protein YbgE

b0736 ybgC esterase/thioesterase

//

>NGHB#701 -4

CMPLX not

b0736 ybgC esterase/thioesterase

b0737 tolQ Tol-Pal system protein TolQ

//

>NGHB#702 3

CMPLX yes

b0737 tolQ Tol-Pal system protein TolQ

b0738 tolR Tol-Pal system protein TolR

//

>NGHB#703 64

CMPLX yes

b0738 tolR Tol-Pal system protein TolR

b0739 tolA Tol-Pal system protein TolA

//

>NGHB#704 132

CMPLX yes

b0739 tolA Tol-Pal system protein TolA

b0740 tolB Tol-Pal system periplasmic protein TolB

//

>NGHB#705 34

CMPLX not

b0740 tolB Tol-Pal system periplasmic protein TolB

b0741 pal peptidoglycan-associated outer membranelipoprotein Pal

//

>NGHB#706 9

CMPLX not

b0741 pal peptidoglycan-associated outer membranelipoprotein Pal

b0742 cpoB cell division coordinator CpoB

//

>NGHB#707 1695

CMPLX not

b0742 cpoB cell division coordinator CpoB

b0750 nadA quinolinate synthase

//

>NGHB#708 37

CMPLX not

b0750 nadA quinolinate synthase

b0751 pnuC nicotinamide riboside transporter

//

>NGHB#710 113

CMPLX not

b0753 ybgS PF13985 family protein YbgS

b0752 zitB Zn(2(+))/Cd(2(+))/Ni(2(+))/Cu(2(+)) exporter

//

>NGHB#713 201

CMPLX not

b0756 galM galactose-1-epimerase

b0755 gpmA 2,3-bisphosphoglycerate-dependentphosphoglycerate mutase

//

>NGHB#714 -7

CMPLX not

b0757 galK galactokinase

b0756 galM galactose-1-epimerase

//

>NGHB#715 3

CMPLX not

b0758 galT galactose-1-phosphate uridylyltransferase

b0757 galK galactokinase

//

>NGHB#716 9

CMPLX not

b0759 galE UDP-glucose 4-epimerase

b0758 galT galactose-1-phosphate uridylyltransferase

//

>NGHB#717 260

CMPLX not

b0760 modF ABC family protein ModF

b0759 galE UDP-glucose 4-epimerase

//

>NGHB#718 67

CMPLX not

b0761 modE DNA-binding transcriptional dual regulator ModE

b0760 modF ABC family protein ModF

//

>NGHB#720 166

CMPLX not

b0762 acrZ multidrug efflux pump accessory protein AcrZ

b0763 modA molybdate ABC transporter periplasmic bindingprotein

//

>NGHB#721 -1

CMPLX yes

b0763 modA molybdate ABC transporter periplasmic bindingprotein

b0764 modB molybdate ABC transporter membrane subunit

//

>NGHB#722 2

CMPLX yes

b0764 modB molybdate ABC transporter membrane subunit

b0765 modC molybdate ABC transporter ATP binding subunit

//

>NGHB#727 75

CMPLX not

b0769 ybhH putative isomerase YbhH

b0770 ybhI putative tricarboxylate transporter

//

>NGHB#728 182

CMPLX not

b0770 ybhI putative tricarboxylate transporter

b0771 ybhJ putative hydratase YbhJ

//

>NGHB#730 151

CMPLX not

b0773 ybhB putative kinase inhibitor

b0772 ybhC outer membrane lipoprotein YhbC

//

>NGHB#731 58

CMPLX not

b0774 bioA adenosylmethionine-8-amino-7-oxononanoateaminotransferase

b0773 ybhB putative kinase inhibitor

//

>NGHB#733 -4

CMPLX not

b0775 bioB biotin synthase

b0776 bioF 8-amino-7-oxononanoate synthase

//

>NGHB#734 -14

CMPLX not

b0776 bioF 8-amino-7-oxononanoate synthase

b0777 bioC malonyl-acyl carrier protein methyltransferase

//

>NGHB#735 -8

CMPLX not

b0777 bioC malonyl-acyl carrier protein methyltransferase

b0778 bioD dethiobiotin synthetase

//

>NGHB#736 578

CMPLX not

b0778 bioD dethiobiotin synthetase

b0779 uvrB excision nuclease subunit B

//

>NGHB#739 21

CMPLX not

b0781 moaA GTP 3',8'-cyclase

b0782 moaB protein MoaB

//

>NGHB#740 2

CMPLX not

b0782 moaB protein MoaB

b0783 moaC cyclic pyranopterin monophosphate synthase

//

>NGHB#741 -8

CMPLX not

b0783 moaC cyclic pyranopterin monophosphate synthase

b0784 moaD molybdopterin synthase sulfur carrier subunit

//

>NGHB#742 1

CMPLX yes

b0784 moaD molybdopterin synthase sulfur carrier subunit

b0785 moaE molybdopterin synthase catalytic subunit

//

>NGHB#743 136

CMPLX not

b0785 moaE molybdopterin synthase catalytic subunit

b0786 ybhL Bax1-I family protein YbhL

//

>NGHB#744 204

CMPLX not

b0786 ybhL Bax1-I family protein YbhL

b0787 ybhM Bax1-I family protein YbhM

//

>NGHB#746 -1

CMPLX not

b0789 clsB cardiolipin synthase B

b0788 ybhN PF03706 family inner membrane protein YbhN

//

>NGHB#747 -4

CMPLX not

b0790 ybhP endonuclease/exonuclease/phosphatasedomain-containing protein YbhP

b0789 clsB cardiolipin synthase B

//

>NGHB#750 10

CMPLX yes

b0793 ybhS ABC exporter membrane subunit YbhS

b0792 ybhR ABC exporter membrane subunit YbhR

//

>NGHB#751 -8

CMPLX yes

b0794 ybhF ABC exporter ATP binding subunit YbhF

b0793 ybhS ABC exporter membrane subunit YbhS

//

>NGHB#752 -8

CMPLX not

b0795 ybhG HlyD_D23 family protein YbhG

b0794 ybhF ABC exporter ATP binding subunit YbhF

//

>NGHB#753 -1

CMPLX not

b0796 cecR DNA-binding transcriptional dual regulator CecR

b0795 ybhG HlyD_D23 family protein YbhG

//

>NGHB#757 27

CMPLX not

b0799 dinG ATP-dependent DNA helicase DinG

b0800 ybiB nonspecific DNA-binding protein YbiB

//

>NGHB#758 140

CMPLX not

b0800 ybiB nonspecific DNA-binding protein YbiB

b0801 hcxB hydroxycarboxylate dehydrogenase B

//

>NGHB#759 66

CMPLX not

b0801 hcxB hydroxycarboxylate dehydrogenase B

b4769 ybiE protein YbiE

//

>NGHB#761 264

CMPLX not

b0803 ybiI zinc finger domain-containing protein YbiI

b0802 ybiJ DUF1471 domain-containing protein YbiJ

//

>NGHB#762 73

CMPLX not

b0804 ybiX PKHD-type hydroxylase YbiX

b0803 ybiI zinc finger domain-containing protein YbiI

//

>NGHB#763 41

CMPLX not

b0805 fiu iron catecholate outer membrane transporter Fiu

b0804 ybiX PKHD-type hydroxylase YbiX

//

>NGHB#764 264

CMPLX not

b0806 mcbA DUF1471 domain-containing protein McbA

b0805 fiu iron catecholate outer membrane transporter Fiu

//

>NGHB#767 260

CMPLX not

b0809 glnQ L-glutamine ABC transporter ATP binding subunit

b0808 ybiO moderate conductance mechanosensitive channelYbiO

//

>NGHB#768 -4

CMPLX yes

b0810 glnP L-glutamine ABC transporter membrane subunit

b0809 glnQ L-glutamine ABC transporter ATP binding subunit

//

>NGHB#769 138

CMPLX yes

b0811 glnH L-glutamine ABC transporter periplasmic bindingprotein

b0810 glnP L-glutamine ABC transporter membrane subunit

//

>NGHB#770 403

CMPLX not

b0812 dps DNA protection during starvation protein

b0811 glnH L-glutamine ABC transporter periplasmic bindingprotein

//

>NGHB#771 298

CMPLX not

b0813 rhtA L-threonine/L-homoserine exporter

b0812 dps DNA protection during starvation protein

//

>NGHB#773 52

CMPLX not

b4736 yliM protein YliM

b0814 ompX outer membrane protein X

//

>NGHB#775 271

CMPLX not

b4705 mntS small protein MntS

b0815 opgE phosphoethanolamine transferase

//

>NGHB#777 -4

CMPLX not

b0817 mntR DNA-binding transcriptional dual regulator MntR

b0818 ybiR putative transporter YbiR

//

>NGHB#781 151

CMPLX not

b0822 ybiV sugar phosphatase YbiV

b0821 ybiU DUF1479 domain-containing protein YbiU

//

>NGHB#782 145

CMPLX not

b0823 ybiW putative pyruvate formate lyase

b0822 ybiV sugar phosphatase YbiV

//

>NGHB#783 5

CMPLX yes

b0824 ybiY putative pyruvate formate-lyase activatingenzyme YbiY

b0823 ybiW putative pyruvate formate lyase

//

>NGHB#786 -1

CMPLX not

b0827 moeA molybdopterin molybdotransferase

b0826 moeB molybdopterin-synthase adenylyltransferase

//

>NGHB#788 -14

CMPLX not

b0828 iaaA isoaspartyl dipeptidase proenzyme

b0829 gsiA glutathione ABC transporter ATP binding subunitGsiA

//

>NGHB#789 19

CMPLX yes

b0829 gsiA glutathione ABC transporter ATP binding subunitGsiA

b0830 gsiB glutathione ABC transporter periplasmic bindingprotein

//

>NGHB#790 17

CMPLX yes

b0830 gsiB glutathione ABC transporter periplasmic bindingprotein

b0831 gsiC glutathione ABC transporter membrane subunitGsiC

//

>NGHB#791 2

CMPLX yes

b0831 gsiC glutathione ABC transporter membrane subunitGsiC

b0832 gsiD glutathione ABC transporter membrane subunitGsiD

//

>NGHB#792 177

CMPLX not

b0832 gsiD glutathione ABC transporter membrane subunitGsiD

b0833 pdeI putative c-di-GMP phosphodiesterase PdeI

//

>NGHB#793 7

CMPLX not

b0833 pdeI putative c-di-GMP phosphodiesterase PdeI

b0834 dgcI putative diguanylate cyclase DgcI

//

>NGHB#796 110

CMPLX not

b0836 bssR regulator of biofilm formation

b0837 yliI aldose sugar dehydrogenase YliI

//

>NGHB#800 57

CMPLX not

b0841 ybjG undecaprenyl pyrophosphate phosphatase

b0840 deoR DNA-binding transcriptional repressor DeoR

//

>NGHB#803 85

CMPLX ---

b0844 ybjI 5-amino-6-(5-phospho-D-ribitylamino)uracilphosphatase

b0843 ybjH uncharacterized protein YbjH

//

>NGHB#804 -1

CMPLX not

b0845 ybjJ inner membrane protein YbjJ

b0844 ybjI 5-amino-6-(5-phospho-D-ribitylamino)uracilphosphatase

//

>NGHB#810 -17

CMPLX not

b0850 ybjC DUF1418 domain-containing protein YbjC

b0851 nfsA NADPH-dependent nitroreductase NfsA

//

>NGHB#811 60

CMPLX not

b0851 nfsA NADPH-dependent nitroreductase NfsA

b0852 rimK ribosomal protein S6 modification protein

//

>NGHB#812 87

CMPLX not

b0852 rimK ribosomal protein S6 modification protein

b0853 ybjN protein YbjN

//

>NGHB#813 350

CMPLX not

b0853 ybjN protein YbjN

b0854 potF putrescine ABC transporter periplasmic bindingprotein

//

>NGHB#814 94

CMPLX yes

b0854 potF putrescine ABC transporter periplasmic bindingprotein

b0855 potG putrescine ABC transporter ATP binding subunit

//

>NGHB#815 9

CMPLX yes

b0855 potG putrescine ABC transporter ATP binding subunit

b0856 potH putrescine ABC transporter membrane subunitPotH

//

>NGHB#816 -4

CMPLX yes

b0856 potH putrescine ABC transporter membrane subunitPotH

b0857 potI putrescine ABC transporter membrane subunitPotI

//

>NGHB#817 59

CMPLX not

b0857 potI putrescine ABC transporter membrane subunitPotI

b0858 ybjO putative inner membrane protein

//

>NGHB#818 40

CMPLX not

b0858 ybjO putative inner membrane protein

b0859 rlmC 23S rRNA m(5)U747 methyltransferase

//

>NGHB#820 290

CMPLX yes

b0861 artM L-arginine ABC transporter membrane subunitArtM

b0860 artJ L-arginine ABC transporter periplasmic bindingprotein

//

>NGHB#821 -1

CMPLX yes

b0862 artQ L-arginine ABC transporter membrane subunitArtQ

b0861 artM L-arginine ABC transporter membrane subunitArtM

//

>NGHB#822 6

CMPLX not

b0863 artI putative ABC transporter periplasmic bindingprotein ArtI

b0862 artQ L-arginine ABC transporter membrane subunitArtQ

//

>NGHB#823 17

CMPLX not

b0864 artP L-arginine ABC transporter ATP binding subunit

b0863 artI putative ABC transporter periplasmic bindingprotein ArtI

//

>NGHB#824 217

CMPLX not

b0865 ybjP DUF3828 domain-containing lipoprotein YbjP

b0864 artP L-arginine ABC transporter ATP binding subunit

//

>NGHB#826 -4

CMPLX not

b0866 ybjQ putative heavy metal binding protein YbjQ

b0867 amiD N-acetylmuramoyl-L-alanine amidase D

//

>NGHB#828 98

CMPLX not

b0869 ybjT putative NAD(P)-dependent oxidoreductase YbjT

b0868 ybjS putative epimerase YbjS

//

>NGHB#829 10

CMPLX not

b0870 ltaE low-specificity L-threonine aldolase

b0869 ybjT putative NAD(P)-dependent oxidoreductase YbjT

//

>NGHB#830 36

CMPLX not

b0871 poxB pyruvate oxidase

b0870 ltaE low-specificity L-threonine aldolase

//

>NGHB#831 132

CMPLX not

b0872 hcr NADH oxidoreductase

b0871 poxB pyruvate oxidase

//

>NGHB#832 11

CMPLX not

b0873 hcp protein S-nitrosylase

b0872 hcr NADH oxidoreductase

//

>NGHB#833 143

CMPLX not

b0874 lysO L-lysine exporter

b0873 hcp protein S-nitrosylase

//

>NGHB#834 494

CMPLX not

b0875 aqpZ water channel AqpZ

b0874 lysO L-lysine exporter

//

>NGHB#838 -4

CMPLX yes

b0878 macA ABC-type tripartite efflux pump membrane fusionprotein

b0879 macB ABC-type tripartite efflux pump ATPbinding/membrane subunit

//

>NGHB#841 98

CMPLX not

b4770 yljB protein YljB

b0881 clpS specificity factor for ClpA-ClpPchaperone-protease complex

//

>NGHB#842 30

CMPLX not

b0881 clpS specificity factor for ClpA-ClpPchaperone-protease complex

b0882 clpA ATP-dependent Clp protease ATP-binding subunitClpA

//

>NGHB#844 284

CMPLX not

b0885 aat leucyl/phenylalanyl-tRNA--protein transferase

b0884 infA translation initiation factor IF-1

//

>NGHB#845 41

CMPLX not

b0886 cydC glutathione/L-cysteine ABC exporter subunitCydC

b0885 aat leucyl/phenylalanyl-tRNA--protein transferase

//

>NGHB#846 0

CMPLX yes

b0887 cydD glutathione/L-cysteine ABC exporter subunitCydD

b0886 cydC glutathione/L-cysteine ABC exporter subunitCydC

//

>NGHB#847 122

CMPLX not

b0888 trxB thioredoxin reductase

b0887 cydD glutathione/L-cysteine ABC exporter subunitCydD

//

>NGHB#849 134

CMPLX not

b0889 lrp DNA-binding transcriptional dual regulator Lrp

b0890 ftsK cell division DNA translocase FtsK

//

>NGHB#850 158

CMPLX not

b0890 ftsK cell division DNA translocase FtsK

b0891 lolA outer membrane lipoprotein carrier protein

//

>NGHB#851 10

CMPLX not

b0891 lolA outer membrane lipoprotein carrier protein

b0892 rarA replication-associated recombination protein A

//

>NGHB#852 90

CMPLX not

b0892 rarA replication-associated recombination protein A

b0893 serS serine--tRNA ligase

//

>NGHB#853 238

CMPLX not

b0893 serS serine--tRNA ligase

b0894 dmsA dimethyl sulfoxide reductase subunit A

//

>NGHB#854 10

CMPLX yes

b0894 dmsA dimethyl sulfoxide reductase subunit A

b0895 dmsB dimethyl sulfoxide reductase subunit B

//

>NGHB#855 1

CMPLX yes

b0895 dmsB dimethyl sulfoxide reductase subunit B

b0896 dmsC dimethyl sulfoxide reductase subunit C

//

>NGHB#858 209

CMPLX yes

b0898 ycaD putative transporter YcaD

b0899 ycaM putative transporter YcaM

//

>NGHB#862 191

CMPLX yes

b0903 pflB pyruvate formate-lyase

b0902 pflA pyruvate formate-lyase activating enzyme

//

>NGHB#863 54

CMPLX not

b0904 focA formate channel FocA

b0903 pflB pyruvate formate-lyase

//

>NGHB#864 405

CMPLX not

b0905 ycaO ribosomal protein S12 methylthiotransferaseaccessory factor YcaO

b0904 focA formate channel FocA

//

>NGHB#866 198

CMPLX not

b0906 ycaP DUF421 domain-containing protein YcaP

b0907 serC phosphoserine/phosphohydroxythreonineaminotransferase

//

>NGHB#867 70

CMPLX not

b0907 serC phosphoserine/phosphohydroxythreonineaminotransferase

b0908 aroA 3-phosphoshikimate 1-carboxyvinyltransferase

//

>NGHB#868 168

CMPLX not

b0908 aroA 3-phosphoshikimate 1-carboxyvinyltransferase

b0909 ycaL periplasmic protease YcaL

//

>NGHB#869 172

CMPLX not

b0909 ycaL periplasmic protease YcaL

b0910 cmk cytidylate kinase

//

>NGHB#870 110

CMPLX not

b0910 cmk cytidylate kinase

b0911 rpsA 30S ribosomal subunit protein S1

//

>NGHB#871 159

CMPLX not

b0911 rpsA 30S ribosomal subunit protein S1

b0912 ihfB integration host factor subunit beta

//

>NGHB#872 207

CMPLX not

b0912 ihfB integration host factor subunit beta

b0913 ycaI metallo-beta-lactamase superfamily innermembrane protein YcaI

//

>NGHB#873 36

CMPLX not

b0913 ycaI metallo-beta-lactamase superfamily innermembrane protein YcaI

b0914 msbA ATP-binding lipopolysaccharide transportprotein

//

>NGHB#874 -4

CMPLX not

b0914 msbA ATP-binding lipopolysaccharide transportprotein

b0915 lpxK tetraacyldisaccharide 4'-kinase

//

>NGHB#875 36

CMPLX not

b0915 lpxK tetraacyldisaccharide 4'-kinase

b0916 ycaQ interstrand DNA crosslink repair glycosylase

//

>NGHB#876 51

CMPLX not

b0916 ycaQ interstrand DNA crosslink repair glycosylase

b0917 ycaR UPF0434 family protein YcaR

//

>NGHB#877 -4

CMPLX not

b0917 ycaR UPF0434 family protein YcaR

b0918 kdsB 3-deoxy-manno-octulosonate cytidylyltransferase

//

>NGHB#878 153

CMPLX not

b0918 kdsB 3-deoxy-manno-octulosonate cytidylyltransferase

b0919 ycbJ putative phosphotransferase YcbJ

//

>NGHB#881 -4

CMPLX not

b0921 cmoM tRNA cmo(5)U34 methyltransferase

b0922 mukF chromosome partitioning protein MukF

//

>NGHB#882 -20

CMPLX yes

b0922 mukF chromosome partitioning protein MukF

b0923 mukE chromosome partitioning protein MukE

//

>NGHB#883 -1

CMPLX yes

b0923 mukE chromosome partitioning protein MukE

b0924 mukB chromosome partitioning protein MukB

//

>NGHB#884 260

CMPLX not

b0924 mukB chromosome partitioning protein MukB

b0925 ldtD L,D-transpeptidase LdtD

//

>NGHB#885 180

CMPLX not

b0925 ldtD L,D-transpeptidase LdtD

b0926 mepK peptidoglycan LD-endopeptidase

//

>NGHB#886 26

CMPLX not

b0926 mepK peptidoglycan LD-endopeptidase

b0927 gloC hydroxyacylglutathione hydrolase GloC

//

>NGHB#888 184

CMPLX not

b0929 ompF outer membrane porin F

b0928 aspC aspartate aminotransferase

//

>NGHB#889 602

CMPLX not

b0930 asnS asparagine--tRNA ligase

b0929 ompF outer membrane porin F

//

>NGHB#890 168

CMPLX not

b0931 pncB nicotinate phosphoribosyltransferase

b0930 asnS asparagine--tRNA ligase

//

>NGHB#893 -4

CMPLX yes

b0934 ssuC aliphatic sulfonate ABC transporter membranesubunit

b0933 ssuB aliphatic sulfonate ABC transporter ATP bindingsubunit

//

>NGHB#894 10

CMPLX not

b0935 ssuD FMNH2-dependent alkanesulfonate monooxygenase

b0934 ssuC aliphatic sulfonate ABC transporter membranesubunit

//

>NGHB#895 -4

CMPLX not

b0936 ssuA aliphatic sulfonate ABC transporter periplasmicbinding protein

b0935 ssuD FMNH2-dependent alkanesulfonate monooxygenase

//

>NGHB#896 -8

CMPLX not

b0937 ssuE NADPH-dependent FMN reductase

b0936 ssuA aliphatic sulfonate ABC transporter periplasmicbinding protein

//

>NGHB#898 82

CMPLX not

b0938 elfA putative laminin-binding fimbrial subunit

b0939 elfD putative fimbrial chaperone ElfD

//

>NGHB#899 24

CMPLX yes

b0939 elfD putative fimbrial chaperone ElfD

b0940 elfC putative fimbrial usher protein ElfC

//

>NGHB#900 -10

CMPLX not

b0940 elfC putative fimbrial usher protein ElfC

b0941 elfG putative fimbrial-like adhesin protein

//

>NGHB#901 11

CMPLX yes

b0941 elfG putative fimbrial-like adhesin protein

b0942 ycbU putative fimbrial protein YcbU

//

>NGHB#902 7

CMPLX yes

b0942 ycbU putative fimbrial protein YcbU

b0943 ycbV putative fimbrial protein YcbV

//

>NGHB#903 -8

CMPLX not

b0943 ycbV putative fimbrial protein YcbV

b0944 ycbF putative fimbrial chaperone YcbF

//

>NGHB#904 110

CMPLX not

b0944 ycbF putative fimbrial chaperone YcbF

b0945 pyrD dihydroorotate dehydrogenase, type 2

//

>NGHB#905 173

CMPLX not

b0945 pyrD dihydroorotate dehydrogenase, type 2

b0946 zapC cell division protein ZapC

//

>NGHB#908 11

CMPLX not

b0948 rlmL fused 23S rRNA m(2)G2445 methyltransferase and23S rRNA m(7)G2069 methyltransferase

b0949 uup ATP-binding protein Uup

//

>NGHB#909 129

CMPLX not

b0949 uup ATP-binding protein Uup

b0950 pqiA intermembrane transport protein PqiA

//

>NGHB#910 4

CMPLX yes

b0950 pqiA intermembrane transport protein PqiA

b0951 pqiB intermembrane transport protein PqiB

//

>NGHB#911 -4

CMPLX yes

b0951 pqiB intermembrane transport protein PqiB

b0952 pqiC intermembrane transport lipoprotein PqiC

//

>NGHB#912 255

CMPLX not

b0952 pqiC intermembrane transport lipoprotein PqiC

b0953 rmf ribosome modulation factor

//

>NGHB#914 68

CMPLX not

b0955 ycbZ putative ATP-dependent protease YcbZ

b0954 fabA beta-hydroxyacyl-acyl carrier proteindehydratase/isomerase

//

>NGHB#917 356

CMPLX not

b0958 sulA cell division inhibitor SulA

b0957 ompA outer membrane protein A

//

>NGHB#920 18

CMPLX not

b0961 yccF PF03733 family inner membrane protein YccF

b0960 yccS putative transporter YccS

//

>NGHB#923 95

CMPLX not

b0964 yccT DUF2057 domain-containing protein YccT

b0963 mgsA methylglyoxal synthase

//

>NGHB#926 57

CMPLX not

b0967 rlmI 23S rRNA m(5)C1962 methyltransferase

b0966 hspQ heat shock protein HspQ

//

>NGHB#929 90

CMPLX not

b0970 yccA modulator of FtsH protease

b0969 tusE sulfur transfer protein TusE

//

>NGHB#931 -4

CMPLX yes

b0972 hyaA hydrogenase 1 small subunit

b0973 hyaB hydrogenase 1 large subunit

//

>NGHB#932 18

CMPLX yes

b0973 hyaB hydrogenase 1 large subunit

b0974 hyaC hydrogenase 1 cytochrome b subunit

//

>NGHB#933 -4

CMPLX not

b0974 hyaC hydrogenase 1 cytochrome b subunit

b0975 hyaD putative hydrogenase 1 maturation protease HyaD

//

>NGHB#934 -4

CMPLX not

b0975 hyaD putative hydrogenase 1 maturation protease HyaD

b0976 hyaE putative HyaA chaperone

//

>NGHB#935 -4

CMPLX not

b0976 hyaE putative HyaA chaperone

b0977 hyaF protein HyaF

//

>NGHB#936 133

CMPLX not

b0977 hyaF protein HyaF

b0978 appC cytochrome bd-II ubiquinol oxidase subunit I

//

>NGHB#937 11

CMPLX yes

b0978 appC cytochrome bd-II ubiquinol oxidase subunit I

b0979 appB cytochrome bd-II ubiquinol oxidase subunit II

//

>NGHB#938 12

CMPLX not

b0979 appB cytochrome bd-II ubiquinol oxidase subunit II

b4592 appX small protein AppX

//

>NGHB#939 79

CMPLX not

b4592 appX small protein AppX

b0980 appA periplasmic phosphoanhydridephosphatase/multiple inositol-polyphosphate phosphatase

//

>NGHB#941 19

CMPLX not

b0982 etp phosphotyrosine-protein phosphatase Etp

b0981 etk protein-tyrosine kinase Etk

//

>NGHB#942 -13

CMPLX not

b0983 gfcE putative exopolysaccharide export lipoproteinGfcE

b0982 etp phosphotyrosine-protein phosphatase Etp

//

>NGHB#943 45

CMPLX yes

b0984 gfcD putative lipoprotein GfcD

b0983 gfcE putative exopolysaccharide export lipoproteinGfcE

//

>NGHB#944 -1

CMPLX not

b0985 gfcC capsule biosynthesis GfcC family protein

b0984 gfcD putative lipoprotein GfcD

//

>NGHB#945 -4

CMPLX not

b0986 gfcB lipoprotein GfcB

b0985 gfcC capsule biosynthesis GfcC family protein

//

>NGHB#946 106

CMPLX not

b0987 gfcA threonine-rich inner membrane protein GfcA

b0986 gfcB lipoprotein GfcB

//

>NGHB#948 -82

CMPLX tnp

b4516 insA4 IS1 protein InsA

b0988 insB4 IS1 protein InsB

//

>NGHB#951 10

CMPLX not

b0990 cspG cold shock protein CspG

b4723 ymcF protein YmcF

//

>NGHB#952 -26

CMPLX not

b4723 ymcF protein YmcF

b0991 ymcE protein YmcE

//

>NGHB#953 -11

CMPLX not

b0991 ymcE protein YmcE

b4517 gnsA putative phosphatidylethanolamine synthesisregulator GnsA

//

>NGHB#955 71

CMPLX not

b0993 torS sensory histidine kinase TorS

b0992 yccM putative electron transport protein YccM

//

>NGHB#959 -1

CMPLX not

b0996 torC cytochrome c menaquinol dehydrogenase TorC

b0997 torA trimethylamine N-oxide reductase 1

//

>NGHB#960 -4

CMPLX not

b0997 torA trimethylamine N-oxide reductase 1

b0998 torD trimethylamine-N-oxide reductase-specificchaperone

//

>NGHB#962 -1

CMPLX not

b1000 cbpA curved DNA-binding protein

b0999 cbpM chaperone modulator CbpM

//

>NGHB#964 292

CMPLX ---

b1001 yccE uncharacterized protein YccE

b1002 agp glucose-1-phosphatase

//

>NGHB#966 20

CMPLX not

b1004 wrbA NAD(P)H:quinone oxidoreductase

b1003 yccJ PF13993 family protein YccJ

//

>NGHB#969 20

CMPLX not

b1007 rutF FMN reductase RutF

b1006 rutG pyrimidine:H(+) symporter

//

>NGHB#970 10

CMPLX not

b1008 rutE putative malonic semialdehyde reductase

b1007 rutF FMN reductase RutF

//

>NGHB#971 9

CMPLX not

b1009 rutD putative aminoacrylate hydrolase RutD

b1008 rutE putative malonic semialdehyde reductase

//

>NGHB#972 7

CMPLX not

b1010 rutC putative aminoacrylate peracid reductase RutC

b1009 rutD putative aminoacrylate hydrolase RutD

//

>NGHB#973 11

CMPLX not

b1011 rutB peroxyureidoacrylate/ureidoacrylateamidohydrolase

b1010 rutC putative aminoacrylate peracid reductase RutC

//

>NGHB#974 -1

CMPLX not

b1012 rutA pyrimidine monooxygenase RutA

b1011 rutB peroxyureidoacrylate/ureidoacrylateamidohydrolase

//

>NGHB#978 158

CMPLX not

b4737 ymdG protein YmdG

b1015 putP proline:Na(+) symporter

//

>NGHB#979 1429

CMPLX not

b1015 putP proline:Na(+) symporter

b1018 efeO ferrous iron transport system protein EfeO

//

>NGHB#980 5

CMPLX not

b1018 efeO ferrous iron transport system protein EfeO

b1019 efeB heme-containing peroxidase/deferrochelatase

//

>NGHB#981 344

CMPLX not

b1019 efeB heme-containing peroxidase/deferrochelatase

b1020 phoH ATP-binding protein PhoH

//

>NGHB#983 1

CMPLX yes

b1022 pgaC poly-N-acetyl-D-glucosamine synthase subunitPgaC

b1021 pgaD poly-N-acetyl-D-glucosamine synthase subunitPgaD

//

>NGHB#984 -8

CMPLX not

b1023 pgaB poly-beta-1,6-N-acetyl-D-glucosamineN-deacetylase and beta-1,6 glycoside hydrolase

b1022 pgaC poly-N-acetyl-D-glucosamine synthase subunitPgaC

//

>NGHB#985 8

CMPLX not

b1024 pgaA partially deacetylatedpoly-beta-1,6-N-acetyl-D-glucosamine outer membrane porin

b1023 pgaB poly-beta-1,6-N-acetyl-D-glucosamineN-deacetylase and beta-1,6 glycoside hydrolase

//

>NGHB#988 -4

CMPLX tnp

b1027 insE4 IS3 element protein InsE

b1026 insF4 IS3 element protein InsF

//

>NGHB#990 1056

CMPLX ---

b1029 ycdU uncharacterized protein YcdU

b1033 ghrA glyoxylate/hydroxypyruvate reductase A

//

>NGHB#991 54

CMPLX not

b1033 ghrA glyoxylate/hydroxypyruvate reductase A

b1034 ycdX zinc-binding phosphatase YcdX

//

>NGHB#992 23

CMPLX not

b1034 ycdX zinc-binding phosphatase YcdX

b1035 ycdY chaperone protein YcdY

//

>NGHB#993 101

CMPLX not

b1035 ycdY chaperone protein YcdY

b1036 ycdZ inner membrane protein YcdZ

//

>NGHB#995 26

CMPLX not

b1038 csgF curli assembly component CsgF

b1037 csgG curli secretion channel

//

>NGHB#996 24

CMPLX yes

b1039 csgE curli assembly component CsgE

b1038 csgF curli assembly component CsgF

//

>NGHB#997 4

CMPLX not

b1040 csgD DNA-binding transcriptional dual regulator CsgD

b1039 csgE curli assembly component CsgE

//

>NGHB#999 40

CMPLX yes

b1041 csgB curlin, minor subunit

b1042 csgA curlin, major subunit

//

>NGHB#1000 58

CMPLX not

b1042 csgA curlin, major subunit

b1043 csgC curlin chaperone

//

>NGHB#1001 120

CMPLX ---

b1043 csgC curlin chaperone

b1044 ymdA uncharacterized protein YmdA

//

>NGHB#1002 94

CMPLX ---

b1044 ymdA uncharacterized protein YmdA

b1045 ymdB 2'-O-acetyl-ADP-ribose deacetylase, regulator ofRNase III activity

//

>NGHB#1003 1

CMPLX not

b1045 ymdB 2'-O-acetyl-ADP-ribose deacetylase, regulator ofRNase III activity

b1046 clsC cardiolipin synthase C

//

>NGHB#1006 -8

CMPLX yes

b1048 opgG osmoregulated periplasmic glucans (OPGs)biosynthesis protein G

b1049 opgH osmoregulated periplasmic glucans (OPGs)biosynthesis protein H

//

>NGHB#1007 172

CMPLX not

b1049 opgH osmoregulated periplasmic glucans (OPGs)biosynthesis protein H

b1050 yceK DUF1375 domain-containing lipoprotein YceK

//

>NGHB#1009 82

CMPLX not

b1053 mdtG efflux pump MdtG

b1051 msyB acidic protein MsyB

//

>NGHB#1010 171

CMPLX not

b1054 lpxL lauroyl acyltransferase

b1053 mdtG efflux pump MdtG

//

>NGHB#1013 3

CMPLX not

b1057 yceJ putative cytochrome b561 YceJ

b1056 yceI protein YceI

//

>NGHB#1014 260

CMPLX not

b1058 yceO DUF2770 domain-containing protein YceO

b1057 yceJ putative cytochrome b561 YceJ

//

>NGHB#1015 20

CMPLX not

b1059 solA N-methyl-L-tryptophan oxidase

b1058 yceO DUF2770 domain-containing protein YceO

//

>NGHB#1016 114

CMPLX not

b1060 bssS regulator of biofilm formation

b1059 solA N-methyl-L-tryptophan oxidase

//

>NGHB#1017 286

CMPLX not

b1061 dinI DNA damage-inducible protein I

b1060 bssS regulator of biofilm formation

//

>NGHB#1018 73

CMPLX not

b1062 pyrC dihydroorotase

b1061 dinI DNA damage-inducible protein I

//

>NGHB#1019 105

CMPLX not

b1063 yceB putative lipid-binding lipoprotein YceB

b1062 pyrC dihydroorotase

//

>NGHB#1020 133

CMPLX not

b1064 grxB reduced glutaredoxin 2

b1063 yceB putative lipid-binding lipoprotein YceB

//

>NGHB#1021 63

CMPLX not

b1065 mdtH multidrug efflux pump MdtH

b1064 grxB reduced glutaredoxin 2

//

>NGHB#1023 10

CMPLX not

b1066 rimJ ribosomal-protein-S5-alanineN-acetyltransferase

b1067 yceH DUF480 domain-containing protein YceH

//

>NGHB#1024 1

CMPLX not

b1067 yceH DUF480 domain-containing protein YceH

b1068 yceM putative oxidoreductase YceM

//

>NGHB#1025 109

CMPLX not

b1068 yceM putative oxidoreductase YceM

b1069 murJ lipid II flippase MurJ

//

>NGHB#1027 4

CMPLX not

b1071 flgM anti-sigma factor for FliA (sigma(28))

b1070 flgN flagellar biosynthesis protein FlgN

//

>NGHB#1028 75

CMPLX not

b1072 flgA flagellar basal body P-ring formation proteinFlgA

b1071 flgM anti-sigma factor for FliA (sigma(28))

//

>NGHB#1030 3

CMPLX yes

b1073 flgB flagellar basal-body rod protein FlgB

b1074 flgC flagellar basal-body rod protein FlgC

//

>NGHB#1031 11

CMPLX not

b1074 flgC flagellar basal-body rod protein FlgC

b1075 flgD flagellar biosynthesis, initiation of hookassembly

//

>NGHB#1032 24

CMPLX not

b1075 flgD flagellar biosynthesis, initiation of hookassembly

b1076 flgE flagellar hook protein FlgE

//

>NGHB#1033 19

CMPLX yes

b1076 flgE flagellar hook protein FlgE

b1077 flgF flagellar basal-body rod protein FlgF

//

>NGHB#1034 171

CMPLX yes

b1077 flgF flagellar basal-body rod protein FlgF

b1078 flgG flagellar basal-body rod protein FlgG

//

>NGHB#1035 52

CMPLX yes

b1078 flgG flagellar basal-body rod protein FlgG

b1079 flgH flagellar L-ring protein

//

>NGHB#1036 11

CMPLX yes

b1079 flgH flagellar L-ring protein

b1080 flgI flagellar P-ring protein

//

>NGHB#1037 -1

CMPLX not

b1080 flgI flagellar P-ring protein

b1081 flgJ putative peptidoglycan hydrolase FlgJ

//

>NGHB#1038 65

CMPLX not

b1081 flgJ putative peptidoglycan hydrolase FlgJ

b1082 flgK flagellar hook-filament junction protein 1

//

>NGHB#1039 11

CMPLX yes

b1082 flgK flagellar hook-filament junction protein 1

b1083 flgL flagellar hook-filament junction protein 2

//

>NGHB#1042 117

CMPLX not

b1085 yceQ DUF2655 domain-containing protein YceQ

b1086 rluC 23S rRNA pseudouridine(955/2504/2580) synthase

//

>NGHB#1045 51

CMPLX not

b1088 yceD DUF177 domain-containing protein YceD

b1089 rpmF 50S ribosomal subunit protein L32

//

>NGHB#1046 80

CMPLX not

b1089 rpmF 50S ribosomal subunit protein L32

b1090 plsX putative phosphate acyltransferase

//

>NGHB#1047 67

CMPLX not

b1090 plsX putative phosphate acyltransferase

b1091 fabH 3-oxoacyl-[acyl carrier protein] synthase 3

//

>NGHB#1048 15

CMPLX not

b1091 fabH 3-oxoacyl-[acyl carrier protein] synthase 3

b1092 fabD [acyl-carrier-protein] S-malonyltransferase

//

>NGHB#1049 12

CMPLX not

b1092 fabD [acyl-carrier-protein] S-malonyltransferase

b1093 fabG 3-oxoacyl-[acyl-carrier-protein] reductase FabG

//

>NGHB#1050 210

CMPLX not

b1093 fabG 3-oxoacyl-[acyl-carrier-protein] reductase FabG

b1094 acpP acyl carrier protein

//

>NGHB#1051 87

CMPLX not

b1094 acpP acyl carrier protein

b1095 fabF 3-oxoacyl-[acyl carrier protein] synthase 2

//

>NGHB#1052 119

CMPLX not

b1095 fabF 3-oxoacyl-[acyl carrier protein] synthase 2

b1096 pabC aminodeoxychorismate lyase

//

>NGHB#1053 2

CMPLX not

b1096 pabC aminodeoxychorismate lyase

b1097 mltG endolytic murein transglycosylase

//

>NGHB#1054 -11

CMPLX ---

b1097 mltG endolytic murein transglycosylase

b1098 tmk hypothetical protein

//

>NGHB#1055 -4

CMPLX ---

b1098 tmk hypothetical protein

b1099 holB DNA polymerase III subunit delta'

//

>NGHB#1056 10

CMPLX not

b1099 holB DNA polymerase III subunit delta'

b1100 ycfH putative metal-dependent hydrolase YcfH

//

>NGHB#1057 294

CMPLX not

b1100 ycfH putative metal-dependent hydrolase YcfH

b1101 ptsG glucose-specific PTS enzyme IIBC component

//

>NGHB#1060 2

CMPLX not

b1103 hinT purine nucleoside phosphoramidase

b1104 ycfL DUF1425 domain-containing protein YcfL

//

>NGHB#1061 13

CMPLX not

b1104 ycfL DUF1425 domain-containing protein YcfL

b1105 lpoB outer membrane lipoprotein - activator of MrcBactivity

//

>NGHB#1062 -20

CMPLX not

b1105 lpoB outer membrane lipoprotein - activator of MrcBactivity

b1106 thiK thiamine kinase

//

>NGHB#1063 10

CMPLX not

b1106 thiK thiamine kinase

b1107 nagZ beta-N-acetylhexosaminidase

//

>NGHB#1064 22

CMPLX not

b1107 nagZ beta-N-acetylhexosaminidase

b1108 ycfP UPF0227 protein YcfP

//

>NGHB#1065 399

CMPLX not

b1108 ycfP UPF0227 protein YcfP

b1109 ndh NADH:quinone oxidoreductase II

//

>NGHB#1066 209

CMPLX not

b1109 ndh NADH:quinone oxidoreductase II

b1110 ycfJ PF05433 family protein YcfJ

//

>NGHB#1070 143

CMPLX not

b1114 mfd transcription-repair coupling factor

b1113 ldtC L,D-transpeptidase LdtC

//

>NGHB#1071 127

CMPLX not

b1115 ycfT inner membrane protein YcfT

b1114 mfd transcription-repair coupling factor

//

>NGHB#1073 -8

CMPLX yes

b1116 lolC lipoprotein release complex - inner membranesubunit

b1117 lolD lipoprotein release complex - ATP bindingsubunit

//

>NGHB#1074 -1

CMPLX yes

b1117 lolD lipoprotein release complex - ATP bindingsubunit

b1118 lolE lipoprotein release complex - inner membranesubunit

//

>NGHB#1075 28

CMPLX not

b1118 lolE lipoprotein release complex - inner membranesubunit

b1119 nagK N-acetyl-D-glucosamine kinase

//

>NGHB#1076 126

CMPLX not

b1119 nagK N-acetyl-D-glucosamine kinase

b1120 cobB CobB-S

//

>NGHB#1078 -4

CMPLX yes

b1122 ymfA putative inner membrane protein YmfA

b1121 ycfZ putative inner membrane protein

//

>NGHB#1079 57

CMPLX not

b1123 potD spermidine preferential ABC transporterperiplasmic binding protein

b1122 ymfA putative inner membrane protein YmfA

//

>NGHB#1080 -4

CMPLX yes

b1124 potC spermidine preferential ABC transporter membranesubunit PotC

b1123 potD spermidine preferential ABC transporterperiplasmic binding protein

//

>NGHB#1081 -4

CMPLX yes

b1125 potB spermidine preferential ABC transporter membranesubunit PotB

b1124 potC spermidine preferential ABC transporter membranesubunit PotC

//

>NGHB#1082 13

CMPLX yes

b1126 potA spermidine preferential ABC transporter ATPbinding subunit

b1125 potB spermidine preferential ABC transporter membranesubunit PotB

//

>NGHB#1085 75

CMPLX not

b1129 phoQ sensor histidine kinase PhoQ

b1128 roxA ribosomal protein-arginine oxygenase

//

>NGHB#1086 -1

CMPLX not

b1130 phoP DNA-binding transcriptional dual regulator PhoP

b1129 phoQ sensor histidine kinase PhoQ

//

>NGHB#1087 168

CMPLX not

b1131 purB adenylosuccinate lyase

b1130 phoP DNA-binding transcriptional dual regulator PhoP

//

>NGHB#1088 3

CMPLX not

b1132 hflD lysogenization regulator

b1131 purB adenylosuccinate lyase

//

>NGHB#1089 35

CMPLX not

b1133 mnmA tRNA-specific 2-thiouridylase

b1132 hflD lysogenization regulator

//

>NGHB#1090 53

CMPLX not

b1134 nudJ phosphatase NudJ

b1133 mnmA tRNA-specific 2-thiouridylase

//

>NGHB#1091 9

CMPLX not

b1135 rluE 23S rRNA pseudouridine(2457) synthase

b1134 nudJ phosphatase NudJ

//

>NGHB#1094 0

CMPLX ---

b1138 ymfE e14 prophage; uncharacterized protein YmfE

b1137 ymfD e14 prophage; putative SAM-dependentmethyltransferase YmfD

//

>NGHB#1097 -20

CMPLX not

b1141 xisE e14 prophage; putative excisionase

b1140 intE e14 prophage; putative integrase

//

>NGHB#1098 36

CMPLX not

b1142 ymfH e14 prophage; putative protein YmfH

b1141 xisE e14 prophage; putative excisionase

//

>NGHB#1101 174

CMPLX ---

b1145 ymfK e14 prophage; putative repressor protein YmfK

b1144 ymfJ e14 prophage; uncharacterized protein YmfJ

//

>NGHB#1103 43

CMPLX ---

b1146 ymfT e14 prophage; putative DNA-bindingtranscriptional regulator YmfT

b1147 ymfL e14 prophage; uncharacterized protein YmfL

//

>NGHB#1104 -4

CMPLX ---

b1147 ymfL e14 prophage; uncharacterized protein YmfL

b1148 ymfM e14 prophage; uncharacterized protein YmfM

//

>NGHB#1105 9

CMPLX ---

b1148 ymfM e14 prophage; uncharacterized protein YmfM

b1149 ymfN e14 prophage; chimeric replication protein/phageterminase YmfN

//

>NGHB#1106 11

CMPLX not

b1149 ymfN e14 prophage; chimeric replication protein/phageterminase YmfN

b1150 ymfR e14 prophage; IPR020297 domain-containingprotein YmfR

//

>NGHB#1107 -1

CMPLX not

b1150 ymfR e14 prophage; IPR020297 domain-containingprotein YmfR

b1151 beeE e14 prophage; protein BeeE

//

>NGHB#1108 708

CMPLX not

b1151 beeE e14 prophage; protein BeeE

b1153 ymfQ e14 prophage; DUF2313 domain-containing proteinYmfQ

//

>NGHB#1109 3

CMPLX not

b1153 ymfQ e14 prophage; DUF2313 domain-containing proteinYmfQ

b1154 ycfK e14 prophage; protein StfP

//

>NGHB#1110 1

CMPLX not

b1154 ycfK e14 prophage; protein StfP

b1155 tfaP e14 prophage; putative tail fiber assemblyprotein TfaP

//

>NGHB#1113 106

CMPLX not

b1158 pinE e14 prophage; site-specific DNA recombinase

b1159 mcrA e14 prophage; 5-methylcytosine-specificrestriction enzyme McrA

//

>NGHB#1115 256

CMPLX not

b4738 ymgK protein YmgK

b1160 iraM anti-adaptor protein IraM, inhibitor of sigma(S)proteolysis

//

>NGHB#1116 235

CMPLX not

b4739 ymgL protein YmgL

b4738 ymgK protein YmgK

//

>NGHB#1117 52

CMPLX not

b1161 ycgX DUF1398 domain-containing protein YcgX

b4739 ymgL protein YmgL

//

>NGHB#1118 220

CMPLX not

b1162 bluR DNA-binding transcriptional repressor BluR

b1161 ycgX DUF1398 domain-containing protein YcgX

//

>NGHB#1119 204

CMPLX not

b1163 bluF blue light- and temperature-regulatedantirepressor BluF

b1162 bluR DNA-binding transcriptional repressor BluR

//

>NGHB#1121 42

CMPLX yes

b1164 ycgZ putative two-component system connector proteinYcgZ

b1165 ymgA putative two-component system connector proteinYmgA

//

>NGHB#1122 28

CMPLX yes

b1165 ymgA putative two-component system connector proteinYmgA

b1166 ariR putative two-component system connector proteinAriR

//

>NGHB#1123 112

CMPLX not

b1166 ariR putative two-component system connector proteinAriR

b1167 ymgC protein YmgC

//

>NGHB#1124 331

CMPLX not

b1167 ymgC protein YmgC

b1168 pdeG putative c-di-GMP phosphodiesterase PdeG

//

>NGHB#1125 131

CMPLX not

b1168 pdeG putative c-di-GMP phosphodiesterase PdeG

b4520 ymgF inner membrane protein YmgF

//

>NGHB#1127 9

CMPLX not

b1172 ymgG PF13488 family protein YmgG

b1171 ymgD PF16456 family protein YmgD

//

>NGHB#1128 1

CMPLX ---

b4593 ymgI uncharacterized protein YmgI

b1172 ymgG PF13488 family protein YmgG

//

>NGHB#1131 3

CMPLX yes

b1175 minD Z-ring positioning protein MinD

b1174 minE Z-ring positioning protein MinE

//

>NGHB#1132 23

CMPLX yes

b1176 minC Z-ring positioning protein MinC

b1175 minD Z-ring positioning protein MinD

//

>NGHB#1136 71

CMPLX not

b1179 ycgL PF05166 family protein YcgL

b1180 ycgM putative isomerase/hydrolase

//

>NGHB#1137 76

CMPLX not

b1180 ycgM putative isomerase/hydrolase

b1181 ycgN PF03693 family protein YcgN

//

>NGHB#1140 -1

CMPLX yes

b1183 umuD DNA polymerase V protein UmuD

b1184 umuC DNA polymerase V catalytic protein

//

>NGHB#1142 145

CMPLX not

b1186 nhaB Na(+):H(+) antiporter NhaB

b1185 dsbB protein thiol:quinone oxidoreductase DsbB

//

>NGHB#1146 9

CMPLX not

b1189 dadA D-amino acid dehydrogenase

b1190 dadX alanine racemase 2

//

>NGHB#1147 310

CMPLX not

b1190 dadX alanine racemase 2

b4740 ymgM protein YmgM

//

>NGHB#1149 94

CMPLX not

b1192 ldcA murein L,D-carboxypeptidase

b1191 cvrA putative K(+):H(+) antiporter

//

>NGHB#1153 177

CMPLX ---

b1195 ymgE PF04226 family protein YmgE

b1196 ycgY uncharacterized protein YcgY

//

>NGHB#1155 319

CMPLX not

b1198 dhaM dihydroxyacetone kinase subunit M

b1197 treA periplasmic trehalase

//

>NGHB#1156 10

CMPLX yes

b1199 dhaL dihydroxyacetone kinase subunit L

b1198 dhaM dihydroxyacetone kinase subunit M

//

>NGHB#1157 10

CMPLX yes

b1200 dhaK dihydroxyacetone kinase subunit K

b1199 dhaL dihydroxyacetone kinase subunit L

//

>NGHB#1160 768

CMPLX not

b1203 ychF redox-responsive ATPase YchF

b1202 ycgV putative autotransporter adhesin YcgV

//

>NGHB#1161 116

CMPLX not

b1204 pth peptidyl-tRNA hydrolase

b1203 ychF redox-responsive ATPase YchF

//

>NGHB#1164 124

CMPLX not

b1207 prs ribose-phosphate diphosphokinase

b1206 dauA aerobic C4-dicarboxylate transporter DauA

//

>NGHB#1165 150

CMPLX not

b1208 ispE 4-(cytidine5'-diphospho)-2-C-methyl-D-erythritol kinase

b1207 prs ribose-phosphate diphosphokinase

//

>NGHB#1166 -1

CMPLX not

b1209 lolB outer membrane lipoprotein LolB

b1208 ispE 4-(cytidine5'-diphospho)-2-C-methyl-D-erythritol kinase

//

>NGHB#1168 41

CMPLX not

b1210 hemA glutamyl-tRNA reductase

b1211 prfA peptide chain release factor RF1

//

>NGHB#1169 -1

CMPLX not

b1211 prfA peptide chain release factor RF1

b1212 prmC protein-(glutamine-N(5)) methyltransferase

//

>NGHB#1170 -4

CMPLX not

b1212 prmC protein-(glutamine-N(5)) methyltransferase

b1213 ychQ SirB family protein YchQ

//

>NGHB#1171 3

CMPLX not

b1213 ychQ SirB family protein YchQ

b1214 ychA transglutaminase-like/TPR repeat-containingprotein

//

>NGHB#1172 35

CMPLX not

b1214 ychA transglutaminase-like/TPR repeat-containingprotein

b1215 kdsA 3-deoxy-D-manno-octulosonate 8-phosphatesynthase

//

>NGHB#1174 427

CMPLX yes

b4421 ldrB small toxic polypeptide LdrB

b4419 ldrA small toxic polypeptide LdrA

//

>NGHB#1175 427

CMPLX yes

b4423 ldrC small toxic polypeptide LdrC

b4421 ldrB small toxic polypeptide LdrB

//

>NGHB#1176 403

CMPLX not

b1216 chaA Na(+)/K(+):H(+) antiporter ChaA

b4423 ldrC small toxic polypeptide LdrC

//

>NGHB#1178 157

CMPLX not

b1217 chaB putative cation transport regulator ChaB

b1218 chaC glutathione-specificgamma-glutamylcyclotransferase

//

>NGHB#1182 -8

CMPLX not

b1222 narX sensory histidine kinase NarX

b1221 narL DNA-binding transcriptional dual regulator NarL

//

>NGHB#1184 515

CMPLX not

b1223 narK nitrate:nitrite antiporter NarK

b1224 narG nitrate reductase A subunit alpha

//

>NGHB#1185 -4

CMPLX yes

b1224 narG nitrate reductase A subunit alpha

b1225 narH nitrate reductase A subunit beta

//

>NGHB#1186 -4

CMPLX not

b1225 narH nitrate reductase A subunit beta

b1226 narJ nitrate reductase 1 molybdenum cofactor assemblychaperone

//

>NGHB#1187 -1

CMPLX not

b1226 narJ nitrate reductase 1 molybdenum cofactor assemblychaperone

b1227 narI nitrate reductase A subunit gamma

//

>NGHB#1188 182

CMPLX ---

b1227 narI nitrate reductase A subunit gamma

b1228 ychS putative uncharacterized protein YchS

//

>NGHB#1190 605

CMPLX not

b1232 purU formyltetrahydrofolate deformylase

b1229 tpr protamine-like protein

//

>NGHB#1191 49

CMPLX not

b1233 ychJ NTF2-like domain-containing protein YchJ

b1232 purU formyltetrahydrofolate deformylase

//

>NGHB#1193 91

CMPLX not

b1234 rssA putative patatin-like phospholipase RssA

b1235 rssB regulator of RpoS

//

>NGHB#1194 201

CMPLX not

b1235 rssB regulator of RpoS

b1236 galU UTP--glucose-1-phosphate uridylyltransferase

//

>NGHB#1199 30

CMPLX not

b4771 ychT protein YchT

b1242 ychE MarC family putative inner membrane proteinYchE

//

>NGHB#1200 321

CMPLX not

b1242 ychE MarC family putative inner membrane proteinYchE

b4711 insH21 IS5 transposase and trans-activator

//

>NGHB#1201 634

CMPLX not

b4711 insH21 IS5 transposase and trans-activator

b1243 oppA oligopeptide ABC transporter periplasmic bindingprotein

//

>NGHB#1202 85

CMPLX not

b1243 oppA oligopeptide ABC transporter periplasmic bindingprotein

b1244 oppB murein tripeptide ABC transporter/oligopeptideABC transporter inner membrane subunit OppB

//

>NGHB#1203 14

CMPLX yes

b1244 oppB murein tripeptide ABC transporter/oligopeptideABC transporter inner membrane subunit OppB

b1245 oppC murein tripeptide ABC transporter/oligopeptideABC transporter inner membrane subunit OppC

//

>NGHB#1204 11

CMPLX yes

b1245 oppC murein tripeptide ABC transporter/oligopeptideABC transporter inner membrane subunit OppC

b1246 oppD murein tripeptide ABC transporter/oligopeptideABC transporter ATP binding subunit OppD

//

>NGHB#1205 -4

CMPLX yes

b1246 oppD murein tripeptide ABC transporter/oligopeptideABC transporter ATP binding subunit OppD

b1247 oppF murein tripeptide ABC transporter/oligopeptideABC transporter ATP binding subunit OppF

//

>NGHB#1207 34

CMPLX not

b1249 clsA cardiolipin synthase A

b1248 yciU DUF440 domain-containing protein YciU

//

>NGHB#1210 299

CMPLX not

b1251 yciI protein YciI

b1250 kch K(+) channel Kch

//

>NGHB#1213 104

CMPLX not

b1254 yciB inner membrane protein

b1253 yciA acyl-CoA thioesterase YciA

//

>NGHB#1214 29

CMPLX not

b1255 yciC putative inner membrane protein

b1254 yciB inner membrane protein

//

>NGHB#1217 45

CMPLX not

b1258 yciF DUF892 domain-containing protein YciF

b1257 yciE DUF892 domain-containing protein YciE

//

>NGHB#1218 85

CMPLX not

b1259 yciG stress-induced bacterial acidophilic repeatmotifs-containing protein YciG

b1258 yciF DUF892 domain-containing protein YciF

//

>NGHB#1219 380

CMPLX not

b1260 trpA tryptophan synthase subunit alpha

b1259 yciG stress-induced bacterial acidophilic repeatmotifs-containing protein YciG

//

>NGHB#1220 -1

CMPLX yes

b1261 trpB tryptophan synthase subunit beta

b1260 trpA tryptophan synthase subunit alpha

//

>NGHB#1221 11

CMPLX not

b1262 trpC fused indole-3-glycerol phosphatesynthase/phosphoribosylanthranilate isomerase

b1261 trpB tryptophan synthase subunit beta

//

>NGHB#1222 0

CMPLX not

b1263 trpD anthranilate synthase subunit TrpD

b1262 trpC fused indole-3-glycerol phosphatesynthase/phosphoribosylanthranilate isomerase

//

>NGHB#1223 -1

CMPLX yes

b1264 trpE anthranilate synthase subunit TrpE

b1263 trpD anthranilate synthase subunit TrpD

//

>NGHB#1224 91

CMPLX not

b1265 trpL trp operon leader peptide

b1264 trpE anthranilate synthase subunit TrpE

//

>NGHB#1226 -4

CMPLX not

b1266 yciV RNA/ssDNA 5'->3' exonuclease

b1267 yciO putative RNA-binding protein YciO

//

>NGHB#1227 27

CMPLX not

b1267 yciO putative RNA-binding protein YciO

b1268 yciQ DUF2207 domain-containing protein YciQ

//

>NGHB#1228 210

CMPLX not

b1268 yciQ DUF2207 domain-containing protein YciQ

b1269 rluB 23S rRNA pseudouridine(2605) synthase

//

>NGHB#1230 -4

CMPLX not

b1271 yciK putative oxidoreductase

b1270 btuR cobinamide/cobalamin adenosyltransferase

//

>NGHB#1234 209

CMPLX not

b1274 topA DNA topoisomerase 1

b1275 cysB DNA-binding transcriptional dual regulator CysB

//

>NGHB#1235 318

CMPLX ---

b1275 cysB DNA-binding transcriptional dual regulator CysB

b4522 ymiA uncharacterized protein YmiA

//

>NGHB#1236 2

CMPLX ---

b4522 ymiA uncharacterized protein YmiA

b4523 yciX uncharacterized protein YciX

//

>NGHB#1237 113

CMPLX ---

b4523 yciX uncharacterized protein YciX

b4741 ymiC protein YmiC

//

>NGHB#1238 163

CMPLX not

b4741 ymiC protein YmiC

b1276 acnA aconitate hydratase 1

//

>NGHB#1241 148

CMPLX not

b1278 pgpB phosphatidylglycerophosphatase B

b1279 lapA lipopolysaccharide assembly protein A

//

>NGHB#1242 6

CMPLX yes

b1279 lapA lipopolysaccharide assembly protein A

b1280 lapB lipopolysaccharide assembly protein B

//

>NGHB#1243 193

CMPLX not

b1280 lapB lipopolysaccharide assembly protein B

b1281 pyrF orotidine-5'-phosphate decarboxylase

//

>NGHB#1244 -1

CMPLX not

b1281 pyrF orotidine-5'-phosphate decarboxylase

b1282 yciH putative translation factor

//

>NGHB#1246 268

CMPLX not

b1284 yciT putative DNA-binding transcriptional regulatorYciT

b1283 osmB osmotically-inducible lipoprotein OsmB

//

>NGHB#1247 89

CMPLX not

b4596 yciZ UPF0509 protein YciZ

b1284 yciT putative DNA-binding transcriptional regulatorYciT

//

>NGHB#1252 67

CMPLX not

b1287 yciW putative oxidoreductase

b1286 rnb RNase II

//

>NGHB#1253 143

CMPLX not

b1288 fabI enoyl-[acyl-carrier-protein] reductase

b1287 yciW putative oxidoreductase

//

>NGHB#1254 367

CMPLX not

b1289 ycjD DUF559 domain-containing protein YcjD

b1288 fabI enoyl-[acyl-carrier-protein] reductase

//

>NGHB#1255 67

CMPLX not

b1290 sapF putrescine ABC exporter ATP binding proteinSapF

b1289 ycjD DUF559 domain-containing protein YcjD

//

>NGHB#1256 1

CMPLX yes

b1291 sapD putrescine ABC exporter ATP binding proteinSapD

b1290 sapF putrescine ABC exporter ATP binding proteinSapF

//

>NGHB#1257 -1

CMPLX yes

b1292 sapC putrescine ABC exporter membrane protein SapC

b1291 sapD putrescine ABC exporter ATP binding proteinSapD

//

>NGHB#1258 -14

CMPLX yes

b1293 sapB putrescine ABC exporter membrane subunit SapB

b1292 sapC putrescine ABC exporter membrane protein SapC

//

>NGHB#1259 -4

CMPLX yes

b1294 sapA putative periplasmic binding protein SapA

b1293 sapB putrescine ABC exporter membrane subunit SapB

//

>NGHB#1260 312

CMPLX not

b1295 ymjA DUF2543 domain-containing protein YmjA

b1294 sapA putative periplasmic binding protein SapA

//

>NGHB#1261 133

CMPLX not

b1296 puuP putrescine:H(+) symporter PuuP

b1295 ymjA DUF2543 domain-containing protein YmjA

//

>NGHB#1262 -11

CMPLX not

b4742 ymjE protein YmjE

b1296 puuP putrescine:H(+) symporter PuuP

//

>NGHB#1263 148

CMPLX not

b1297 puuA glutamate-putrescine ligase

b4742 ymjE protein YmjE

//

>NGHB#1265 26

CMPLX not

b1298 puuD gamma-glutamyl-gamma-aminobutyrate hydrolase

b1299 puuR DNA-binding transcriptional repressor PuuR

//

>NGHB#1266 274

CMPLX not

b1299 puuR DNA-binding transcriptional repressor PuuR

b1300 puuC gamma-glutamyl-gamma-aminobutyraldehydedehydrogenase

//

>NGHB#1267 1

CMPLX not

b1300 puuC gamma-glutamyl-gamma-aminobutyraldehydedehydrogenase

b1301 puuB gamma-glutamylputrescine oxidase

//

>NGHB#1268 37

CMPLX not

b1301 puuB gamma-glutamylputrescine oxidase

b1302 puuE 4-aminobutyrate aminotransferase PuuE

//

>NGHB#1271 53

CMPLX yes

b1304 pspA phage shock protein A

b1305 pspB phage shock protein B

//

>NGHB#1272 -1

CMPLX yes

b1305 pspB phage shock protein B

b1306 pspC phage shock protein C

//

>NGHB#1273 8

CMPLX yes

b1306 pspC phage shock protein C

b1307 pspD phage shock protein D

//

>NGHB#1274 74

CMPLX not

b1307 pspD phage shock protein D

b1308 pspE thiosulfate sulfurtransferase PspE

//

>NGHB#1275 212

CMPLX not

b1308 pspE thiosulfate sulfurtransferase PspE

b1309 ycjM glucosylglycerate phosphorylase

//

>NGHB#1276 13

CMPLX not

b1309 ycjM glucosylglycerate phosphorylase

b1310 ycjN putative ABC transporter periplasmic bindingprotein YcjN

//

>NGHB#1277 20

CMPLX yes

b1310 ycjN putative ABC transporter periplasmic bindingprotein YcjN

b1311 ycjO putative ABC transporter membrane subunit YcjO

//

>NGHB#1278 -14

CMPLX yes

b1311 ycjO putative ABC transporter membrane subunit YcjO

b1312 ycjP putative ABC transporter membrane subunit YcjP

//

>NGHB#1279 30

CMPLX not

b1312 ycjP putative ABC transporter membrane subunit YcjP

b1313 ycjQ D-guloside 3-dehydrogenase

//

>NGHB#1280 18

CMPLX not

b1313 ycjQ D-guloside 3-dehydrogenase

b1314 ycjR 3-dehydro-D-guloside 4-epimerase

//

>NGHB#1281 9

CMPLX not

b1314 ycjR 3-dehydro-D-guloside 4-epimerase

b1315 ycjS D-glucoside 3-dehydrogenase

//

>NGHB#1282 -4

CMPLX not

b1315 ycjS D-glucoside 3-dehydrogenase

b1316 ycjT kojibiose phosphorylase

//

>NGHB#1283 -4

CMPLX not

b1316 ycjT kojibiose phosphorylase

b1317 ycjU beta-phosphoglucomutase

//

>NGHB#1284 1139

CMPLX not

b1317 ycjU beta-phosphoglucomutase

b1319 ompG outer membrane porin G

//

>NGHB#1287 -4

CMPLX not

b1321 ycjX DUF463 domain-containing protein YcjX

b1322 ycjF DUF697 domain-containing inner membrane proteinYcjF

//

>NGHB#1288 147

CMPLX not

b1322 ycjF DUF697 domain-containing inner membrane proteinYcjF

b1323 tyrR DNA-binding transcriptional dual regulator TyrR

//

>NGHB#1292 81

CMPLX ---

b4525 ymjC putative uncharacterized protein YmjC

b1326 mpaA murein tripeptide amidase A

//

>NGHB#1293 5

CMPLX ---

b4673 ymjD uncharacterized protein YmjD

b4525 ymjC putative uncharacterized protein YmjC

//

>NGHB#1294 -1

CMPLX ---

b1327 ycjY putative hydrolase YcjY

b4673 ymjD uncharacterized protein YmjD

//

>NGHB#1296 336

CMPLX not

b1328 pgrR DNA-binding transcriptional repressor PgrR

b1329 mppA murein tripeptide ABC transporter periplasmicbinding protein

//

>NGHB#1299 272

CMPLX not

b1331 insH4 IS5 transposase and trans-activator

b1332 ynaJ DUF2534 domain-containing protein YnaJ

//

>NGHB#1301 151

CMPLX not

b1334 fnr DNA-binding transcriptional dual regulator FNR

b1333 uspE universal stress protein E

//

>NGHB#1302 194

CMPLX not

b1335 ogt methylated-DNA--[protein]-cysteineS-methyltransferase

b1334 fnr DNA-binding transcriptional dual regulator FNR

//

>NGHB#1303 10

CMPLX not

b1336 abgT p-aminobenzoyl glutamate:H(+) symporter

b1335 ogt methylated-DNA--[protein]-cysteineS-methyltransferase

//

>NGHB#1304 36

CMPLX not

b1337 abgB p-aminobenzoyl-glutamate hydrolase subunit B

b1336 abgT p-aminobenzoyl glutamate:H(+) symporter

//

>NGHB#1305 -1

CMPLX yes

b1338 abgA p-aminobenzoyl-glutamate hydrolase subunit A

b1337 abgB p-aminobenzoyl-glutamate hydrolase subunit B

//

>NGHB#1307 329

CMPLX not

b1339 abgR putative LysR-type DNA-binding transcriptionalregulator AbgR

b1340 smrA DNA endonuclease SmrA

//

>NGHB#1310 13

CMPLX not

b4773 ynaN protein YnaN

b1342 zntB Zn(2(+)):H(+) symporter

//

>NGHB#1311 274

CMPLX not

b1342 zntB Zn(2(+)):H(+) symporter

b4743 ynaL protein YnaL

//

>NGHB#1312 29

CMPLX not

b4743 ynaL protein YnaL

b1343 dbpA ATP-dependent RNA helicase DbpA

//

>NGHB#1314 51

CMPLX not

b1345 intR Rac prophage; putative integrase

b1344 ttcA tRNA cytosine(32) 2-sulfurtransferase TtcA

//

>NGHB#1315 1

CMPLX not

b1346 xisR Rac prophage; excisionase

b1345 intR Rac prophage; putative integrase

//

>NGHB#1316 78

CMPLX not

b1347 rcbA Rac prophage; double-strand break reductionprotein

b1346 xisR Rac prophage; excisionase

//

>NGHB#1317 -8

CMPLX not

b1348 ralR Rac prophage; endodeoxyribonuclease toxin RalR

b1347 rcbA Rac prophage; double-strand break reductionprotein

//

>NGHB#1318 56

CMPLX not

b1349 recT Rac prophage; recombinase RecT

b1348 ralR Rac prophage; endodeoxyribonuclease toxin RalR

//

>NGHB#1319 -8

CMPLX not

b1350 recE Rac prophage; exonuclease VIII, ds DNAexonuclease, 5' --> 3' specific

b1349 recT Rac prophage; recombinase RecT

//

>NGHB#1320 101

CMPLX not

b1351 racC Rac prophage; protein RacC

b1350 recE Rac prophage; exonuclease VIII, ds DNAexonuclease, 5' --> 3' specific

//

>NGHB#1321 74

CMPLX not

b4526 ydaE Rac prophage; zinc-binding protein

b1351 racC Rac prophage; protein RacC

//

>NGHB#1322 -1

CMPLX not

b1352 kilR Rac prophage; inhibitor of FtsZ

b4526 ydaE Rac prophage; zinc-binding protein

//

>NGHB#1325 10

CMPLX ---

b1355 ydaG Rac prophage; uncharacterized protein YdaG

b4527 ydaF Rac prophage; DUF1391 domain-containing proteinYdaF

//

>NGHB#1326 308

CMPLX ---

b1356 racR Rac prophage; DNA-binding transcriptionalrepressor RacR

b1355 ydaG Rac prophage; uncharacterized protein YdaG

//

>NGHB#1328 22

CMPLX not

b1357 ydaS Rac prophage; toxin YdaS

b1358 ydaT Rac prophage; protein YdaT

//

>NGHB#1329 12

CMPLX not

b1358 ydaT Rac prophage; protein YdaT

b1359 ydaU Rac prophage; DUF1376 domain-containing proteinYdaU

//

>NGHB#1330 6

CMPLX not

b1359 ydaU Rac prophage; DUF1376 domain-containing proteinYdaU

b1360 ydaV Rac prophage; putative ATP-binding protein YdaV

//

>NGHB#1331 22

CMPLX ---

b1360 ydaV Rac prophage; putative ATP-binding protein YdaV

b1361 ydaW Rac prophage; putative uncharacterized proteinYdaW

//

>NGHB#1332 87

CMPLX ---

b1361 ydaW Rac prophage; putative uncharacterized proteinYdaW

b4528 rzoR Rac prophage; putative prophage outer membranelipoprotein RzoR

//

>NGHB#1333 196

CMPLX not

b4528 rzoR Rac prophage; putative prophage outer membranelipoprotein RzoR

b1363 trkG Rac prophage; K(+) transporter TrkG

//

>NGHB#1334 137

CMPLX not

b1363 trkG Rac prophage; K(+) transporter TrkG

b1365 ynaK Rac prophage; ParB-like nucleasedomain-containing protein YnaK

//

>NGHB#1335 -20

CMPLX ---

b1365 ynaK Rac prophage; ParB-like nucleasedomain-containing protein YnaK

b1366 ydaY Rac prophage; putative uncharacterized proteinYdaY

//

>NGHB#1336 473

CMPLX ---

b1366 ydaY Rac prophage; putative uncharacterized proteinYdaY

b1368 ynaA Rac prophage; putative prophage tail length tapemeasure domain-containing protein YnaA

//

>NGHB#1337 -94

CMPLX not

b1368 ynaA Rac prophage; putative prophage tail length tapemeasure domain-containing protein YnaA

b4570 lomR Rac prophage; protein LomR_1

//

>NGHB#1338 -1

CMPLX not

b1372 stfR Rac prophage; putative prophage side tail fiberprotein StfR

b1373 tfaR Rac prophage; putative tail fiber assemblyprotein TfaR

//

>NGHB#1340 316

CMPLX ---

b1375 ynaE Rac prophage; uncharacterized protein YnaE

b1374 pinR Rac prophage; putative site-specificrecombinase

//

>NGHB#1341 68

CMPLX ---

b4744 ynaM Rac prophage; protein YnaM

b1375 ynaE Rac prophage; uncharacterized protein YnaE

//

>NGHB#1342 778

CMPLX not

b1376 uspF nucleotide binding filament protein

b4744 ynaM Rac prophage; protein YnaM

//

>NGHB#1343 140

CMPLX not

b1377 ompN outer membrane porin N

b1376 uspF nucleotide binding filament protein

//

>NGHB#1344 366

CMPLX not

b1378 ydbK putative pyruvate-flavodoxin oxidoreductase

b1377 ompN outer membrane porin N

//

>NGHB#1347 110

CMPLX not

b1380 ldhA D-lactate dehydrogenase

b1379 hslJ lipoprotein implicated in Novobiocin resistance

//

>NGHB#1349 -4

CMPLX not

b1381 ydbH PF11739 family protein YdbH

b1382 ynbE lipoprotein YnbE

//

>NGHB#1350 7

CMPLX not

b1382 ynbE lipoprotein YnbE

b1383 ydbL DUF1318 domain-containing protein YdbL

//

>NGHB#1354 247

CMPLX not

b1387 paaZ fused 3-oxo-5,6-dehydrosuberyl-CoA semialdehydedehydrogenase and oxepin-CoA hydrolase

b1386 tynA copper-containing amine oxidase

//

>NGHB#1356 11

CMPLX yes

b1388 paaA phenylacetyl-CoA 1,2-epoxidase, monooxygenasesubunit

b1389 paaB phenylacetyl-CoA 1,2-epoxidase subunit B

//

>NGHB#1357 8

CMPLX yes

b1389 paaB phenylacetyl-CoA 1,2-epoxidase subunit B

b1390 paaC phenylacetyl-CoA 1,2-epoxidase, structuralsubunit

//

>NGHB#1358 14

CMPLX not

b1390 paaC phenylacetyl-CoA 1,2-epoxidase, structuralsubunit

b1391 paaD phenylacetate degradation protein

//

>NGHB#1359 7

CMPLX not

b1391 paaD phenylacetate degradation protein

b1392 paaE phenylacetyl-CoA 1,2-epoxidase, reductasesubunit

//

>NGHB#1360 -4

CMPLX not

b1392 paaE phenylacetyl-CoA 1,2-epoxidase, reductasesubunit

b1393 paaF putative 2,3-dehydroadipyl-CoA hydratase

//

>NGHB#1361 -1

CMPLX not

b1393 paaF putative 2,3-dehydroadipyl-CoA hydratase

b1394 paaG putative ring 1,2-epoxyphenylacetyl-CoAisomerase (oxepin-CoA forming)

//

>NGHB#1362 1

CMPLX not

b1394 paaG putative ring 1,2-epoxyphenylacetyl-CoAisomerase (oxepin-CoA forming)

b1395 paaH 3-hydroxyadipyl-CoA dehydrogenase

//

>NGHB#1363 -11

CMPLX not

b1395 paaH 3-hydroxyadipyl-CoA dehydrogenase

b1396 paaI phenylacetyl-CoA thioesterase

//

>NGHB#1364 -1

CMPLX not

b1396 paaI phenylacetyl-CoA thioesterase

b1397 paaJ beta-ketoadipyl-CoA thiolase

//

>NGHB#1365 26

CMPLX not

b1397 paaJ beta-ketoadipyl-CoA thiolase

b1398 paaK phenylacetate-CoA ligase

//

>NGHB#1366 100

CMPLX not

b1398 paaK phenylacetate-CoA ligase

b1399 paaX DNA-binding transcriptional repressor PaaX

//

>NGHB#1367 -19

CMPLX not

b1399 paaX DNA-binding transcriptional repressor PaaX

b1400 paaY 2-hydroxycyclohepta-1,4,6-triene-1-carboxyl-CoAthioesterase

//

>NGHB#1370 -43

CMPLX tnp

b1403 insC2 IS2 insertion element repressor InsA

b1402 insD2 IS2 insertion element protein InsB

//

>NGHB#1371 62

CMPLX not

b1406 pdxI pyridoxine 4-dehydrogenase

b1407 ydbD DUF2773 domain-containing protein YdbD

//

>NGHB#1372 170

CMPLX not

b1407 ydbD DUF2773 domain-containing protein YdbD

b1408 ynbA CDP-alcohol phosphatidyltransferasedomain-containing protein YnbA

//

>NGHB#1373 -1

CMPLX not

b1408 ynbA CDP-alcohol phosphatidyltransferasedomain-containing protein YnbA

b1409 ynbB putative CDP-diglyceride synthase

//

>NGHB#1374 15

CMPLX not

b1409 ynbB putative CDP-diglyceride synthase

b1410 ynbC hydrolase/methyltransferase domain-containingprotein YnbC

//

>NGHB#1375 13

CMPLX not

b1410 ynbC hydrolase/methyltransferase domain-containingprotein YnbC

b1411 ynbD phosphatase domain-containing protein YnbD

//

>NGHB#1378 271

CMPLX not

b1413 hrpA ATP-dependent RNA helicase HrpA

b1414 ydcF DUF218 domain-containing protein YdcF

//

>NGHB#1379 196

CMPLX not

b1414 ydcF DUF218 domain-containing protein YdcF

b1415 aldA aldehyde dehydrogenase A

//

>NGHB#1380 1230

CMPLX not

b1415 aldA aldehyde dehydrogenase A

b1418 cybB superoxide oxidase

//

>NGHB#1381 244

CMPLX not

b1418 cybB superoxide oxidase

b1419 ydcA protein YdcA

//

>NGHB#1382 25

CMPLX not

b1419 ydcA protein YdcA

b4774 yncP protein YncP

//

>NGHB#1384 -110

CMPLX not

b1420 mokB putative regulatory protein MokB

b4428 hokB toxin HokB

//

>NGHB#1388 224

CMPLX not

b1423 ydcJ DUF1338 domain-containing protein YdcJ

b1424 opgD glucan biosynthesis protein D

//

>NGHB#1389 139

CMPLX not

b1424 opgD glucan biosynthesis protein D

b1426 ydcH protein YdcH

//

>NGHB#1390 62

CMPLX not

b1426 ydcH protein YdcH

b1427 rimL ribosomal-protein-L12-serineN-acetyltransferase

//

>NGHB#1393 -4

CMPLX not

b1429 tehA tellurite resistance protein

b1430 tehB tellurite methyltransferase

//

>NGHB#1394 301

CMPLX not

b1430 tehB tellurite methyltransferase

b1431 ydcL DUF3313 domain-containing lipoprotein YdcL

//

>NGHB#1395 591

CMPLX not

b1431 ydcL DUF3313 domain-containing lipoprotein YdcL

b1432 insQ putative insertion element transposase InsQ

//

>NGHB#1398 72

CMPLX not

b1434 sutR DNA-binding transcriptional dual regulator SutR

b1435 rlhA 23S rRNA 5-hydroxycytidine C2501 synthase

//

>NGHB#1401 45

CMPLX yes

b4532 hicA toxin of the HicA-HicB toxin-antitoxin system

b1438 hicB antitoxin of the HicA-HicB toxin-antitoxinsystem/ DNA-binding transcriptional repressor HicB

//

>NGHB#1402 78

CMPLX not

b1438 hicB antitoxin of the HicA-HicB toxin-antitoxinsystem/ DNA-binding transcriptional repressor HicB

b1439 ydcR fused putative DNA-binding transcriptionalregulator/putative aminotransferase YdcR

//

>NGHB#1403 244

CMPLX not

b1439 ydcR fused putative DNA-binding transcriptionalregulator/putative aminotransferase YdcR

b1440 ydcS putative ABC transporter periplasmic bindingprotein/polyhydroxybutyrate synthase

//

>NGHB#1404 17

CMPLX yes

b1440 ydcS putative ABC transporter periplasmic bindingprotein/polyhydroxybutyrate synthase

b1441 ydcT putative ABC transporter ATP-binding proteinYdcT

//

>NGHB#1405 0

CMPLX yes

b1441 ydcT putative ABC transporter ATP-binding proteinYdcT

b1442 ydcU putative ABC transporter membrane subunit YdcU

//

>NGHB#1406 -11

CMPLX yes

b1442 ydcU putative ABC transporter membrane subunit YdcU

b1443 ydcV putative ABC transporter membrane subunit YdcV

//

>NGHB#1407 21

CMPLX not

b1443 ydcV putative ABC transporter membrane subunit YdcV

b1444 patD gamma-aminobutyraldehyde dehydrogenase

//

>NGHB#1410 85

CMPLX not

b1445 ortT orphan toxin OrtT

b1446 ydcY DUF2526 domain-containing protein YdcY

//

>NGHB#1412 -4

CMPLX not

b1448 mnaT L-amino acid N-acyltransferase

b1447 ydcZ putative inner membrane protein YdcZ

//

>NGHB#1414 197

CMPLX not

b1449 curA NADPH-dependent curcumin/dihydrocurcuminreductase

b1450 mcbR DNA-binding transcriptional dual regulator McbR

//

>NGHB#1419 75

CMPLX not

b1454 yncG putative glutathione S-transferase YncG

b1455 yncH DUF5445 domain-containing protein YncH

//

>NGHB#1420 2769

CMPLX ---

b1455 yncH DUF5445 domain-containing protein YncH

b1457 ydcD uncharacterized protein YdcD

//

>NGHB#1423 99

CMPLX not

b1460 ydcC H repeat-associated putative transposase YdcC

b1461 pptA tautomerase PptA

//

>NGHB#1427 78

CMPLX not

b1465 narV nitrate reductase Z subunit gamma

b1464 yddE PF02567 family protein YddE

//

>NGHB#1428 -4

CMPLX not

b1466 narW NarW, putative private chaperone for NarZnitrate reductase subunit

b1465 narV nitrate reductase Z subunit gamma

//

>NGHB#1429 -1

CMPLX not

b1467 narY nitrate reductase Z subunit beta

b1466 narW NarW, putative private chaperone for NarZnitrate reductase subunit

//

>NGHB#1430 -4

CMPLX yes

b1468 narZ nitrate reductase Z subunit alpha

b1467 narY nitrate reductase Z subunit beta

//

>NGHB#1431 81

CMPLX not

b1469 narU nitrate/nitrite transporter NarU

b1468 narZ nitrate reductase Z subunit alpha

//

>NGHB#1432 697

CMPLX not

b1471 yddK leucine-rich repeat domain-containing proteinYddK

b1469 narU nitrate/nitrite transporter NarU

//

>NGHB#1433 23

CMPLX ---

b1472 yddL putative uncharacterized protein YddL

b1471 yddK leucine-rich repeat domain-containing proteinYddK

//

>NGHB#1434 259

CMPLX ---

b1473 yddG amino acid exporter YddG

b1472 yddL putative uncharacterized protein YddL

//

>NGHB#1436 12

CMPLX yes

b1474 fdnG formate dehydrogenase N subunit alpha

b1475 fdnH formate dehydrogenase N subunit beta

//

>NGHB#1437 -8

CMPLX yes

b1475 fdnH formate dehydrogenase N subunit beta

b1476 fdnI formate dehydrogenase N subunit gamma

//

>NGHB#1439 145

CMPLX not

b1478 adhP ethanol dehydrogenase/alcohol dehydrogenase

b1477 yddM DNA-binding transcriptional regulator YddM

//

>NGHB#1440 133

CMPLX not

b1479 maeA NAD(+)-dependent malate dehydrogenase

b1478 adhP ethanol dehydrogenase/alcohol dehydrogenase

//

>NGHB#1441 156

CMPLX not

b1480 sra 30S ribosomal subunit protein S22

b1479 maeA NAD(+)-dependent malate dehydrogenase

//

>NGHB#1442 101

CMPLX not

b1481 bdm biofilm-dependent modulation protein

b1480 sra 30S ribosomal subunit protein S22

//

>NGHB#1445 -8

CMPLX yes

b1484 ddpD putative D,D-dipeptide ABC transporterATP-binding subunit DdpD

b1483 ddpF putative D,D-dipeptide ABC transporterATP-binding subunit DdpF

//

>NGHB#1446 -4

CMPLX yes

b1485 ddpC putative D,D-dipeptide ABC transporter membranesubunit DdpC

b1484 ddpD putative D,D-dipeptide ABC transporterATP-binding subunit DdpD

//

>NGHB#1447 -4

CMPLX yes

b1486 ddpB putative D,D-dipeptide ABC transporter membranesubunit DdpB

b1485 ddpC putative D,D-dipeptide ABC transporter membranesubunit DdpC

//

>NGHB#1448 1

CMPLX yes

b1487 ddpA putative D,D-dipeptide ABC transporterperiplasmic binding protein

b1486 ddpB putative D,D-dipeptide ABC transporter membranesubunit DdpB

//

>NGHB#1449 13

CMPLX not

b1488 ddpX D-alanyl-D-alanine dipeptidase

b1487 ddpA putative D,D-dipeptide ABC transporterperiplasmic binding protein

//

>NGHB#1450 257

CMPLX not

b1489 dosP oxygen-sensing c-di-GMP phosphodiesterase DosP

b1488 ddpX D-alanyl-D-alanine dipeptidase

//

>NGHB#1451 24

CMPLX not

b1490 dosC diguanylate cyclase DosC

b1489 dosP oxygen-sensing c-di-GMP phosphodiesterase DosP

//

>NGHB#1452 37

CMPLX not

b4746 yddY protein YddY

b1490 dosC diguanylate cyclase DosC

//

>NGHB#1453 284

CMPLX not

b1491 digH divisome localized glycosyl hydrolase

b4746 yddY protein YddY

//

>NGHB#1454 130

CMPLX not

b1492 gadC L-glutamate:4-aminobutyrate antiporter

b1491 digH divisome localized glycosyl hydrolase

//

>NGHB#1455 155

CMPLX not

b1493 gadB glutamate decarboxylase B

b1492 gadC L-glutamate:4-aminobutyrate antiporter

//

>NGHB#1456 361

CMPLX not

b1494 pqqL periplasmic metalloprotease

b1493 gadB glutamate decarboxylase B

//

>NGHB#1457 44

CMPLX not

b1495 yddB putative TonB-dependent receptor YddB

b1494 pqqL periplasmic metalloprotease

//

>NGHB#1458 37

CMPLX not

b1496 yddA ABC transporter family protein YddA

b1495 yddB putative TonB-dependent receptor YddB

//

>NGHB#1461 51

CMPLX not

b1498 ydeN putative sulfatase YdeN

b1497 ydeM putative anaerobic sulfatase maturation enzymeYdeM

//

>NGHB#1462 401

CMPLX not

b1499 ydeO DNA-binding transcriptional dual regulator YdeO

b1498 ydeN putative sulfatase YdeN

//

>NGHB#1463 74

CMPLX not

b1500 safA two-component system connector SafA

b1499 ydeO DNA-binding transcriptional dual regulator YdeO

//

>NGHB#1464 247

CMPLX not

b1501 ydeP putative oxidoreductase YdeP

b1500 safA two-component system connector SafA

//

>NGHB#1465 333

CMPLX not

b1502 ydeQ putative fimbrial adhesin protein YdeQ

b1501 ydeP putative oxidoreductase YdeP

//

>NGHB#1466 58

CMPLX yes

b1503 ydeR putative fimbrial protein YdeR

b1502 ydeQ putative fimbrial adhesin protein YdeQ

//

>NGHB#1467 12

CMPLX yes

b1504 ydeS putative fimbrial protein YdeS

b1503 ydeR putative fimbrial protein YdeR

//

>NGHB#1468 13

CMPLX not

b1505 ydeT fimbrial usher domain-containing protein YdeT

b1504 ydeS putative fimbrial protein YdeS

//

>NGHB#1469 852

CMPLX not

b1507 hipA serine/threonine-protein kinase toxin HipA

b1505 ydeT fimbrial usher domain-containing protein YdeT

//

>NGHB#1470 -1

CMPLX yes

b1508 hipB antitoxin/DNA-binding transcriptional repressorHipB

b1507 hipA serine/threonine-protein kinase toxin HipA

//

>NGHB#1471 6174

CMPLX not

b1511 lsrK autoinducer-2 kinase

b1508 hipB antitoxin/DNA-binding transcriptional repressorHipB

//

>NGHB#1472 78

CMPLX not

b1512 lsrR DNA-binding transcriptional repressor LsrR

b1511 lsrK autoinducer-2 kinase

//

>NGHB#1474 -7

CMPLX yes

b1513 lsrA Autoinducer-2 ABC transporter ATP bindingsubunit

b1514 lsrC Autoinducer-2 ABC transporter membrane subunitLsrC

//

>NGHB#1475 -1

CMPLX yes

b1514 lsrC Autoinducer-2 ABC transporter membrane subunitLsrC

b1515 lsrD Autoinducer-2 ABC transporter membrane subunitLsrD

//

>NGHB#1476 11

CMPLX yes

b1515 lsrD Autoinducer-2 ABC transporter membrane subunitLsrD

b1516 lsrB Autoinducer-2 ABC transporter periplasmicbinding protein

//

>NGHB#1477 26

CMPLX not

b1516 lsrB Autoinducer-2 ABC transporter periplasmicbinding protein

b1517 lsrF 3-hydroxy-2,4-pentadione 5-phosphate thiolase

//

>NGHB#1478 23

CMPLX not

b1517 lsrF 3-hydroxy-2,4-pentadione 5-phosphate thiolase

b1518 lsrG (4S)-4-hydroxy-5-phosphonooxypentane-2,3-dioneisomerase

//

>NGHB#1479 56

CMPLX not

b1518 lsrG (4S)-4-hydroxy-5-phosphonooxypentane-2,3-dioneisomerase

b1519 tam trans-aconitate 2-methyltransferase

//

>NGHB#1481 206

CMPLX not

b1521 uxaB tagaturonate reductase

b1520 yneE PF01062 family inner membrane protein YneE

//

>NGHB#1482 226

CMPLX not

b1522 dgcF putative diguanylate cyclase DgcF

b1521 uxaB tagaturonate reductase

//

>NGHB#1483 111

CMPLX not

b1523 yneG DUF4186 domain-containing protein YneG

b1522 dgcF putative diguanylate cyclase DgcF

//

>NGHB#1484 -1

CMPLX not

b1524 glsB glutaminase 2

b1523 yneG DUF4186 domain-containing protein YneG

//

>NGHB#1485 63

CMPLX not

b1525 sad succinate semialdehyde dehydrogenase (NAD(P)(+))Sad

b1524 glsB glutaminase 2

//

>NGHB#1487 77

CMPLX not

b1526 yneJ putative LysR-type DNA-binding transcriptionalregulator YneJ

b1527 yneK protein YneK

//

>NGHB#1488 149

CMPLX not

b1527 yneK protein YneK

b1528 ydeA L-arabinose exporter

//

>NGHB#1491 19

CMPLX not

b1530 marR DNA-binding transcriptional repressor MarR

b1531 marA DNA-binding transcriptional dual regulator MarA

//

>NGHB#1492 31

CMPLX not

b1531 marA DNA-binding transcriptional dual regulator MarA

b1532 marB multiple antibiotic resistance protein MarB

//

>NGHB#1495 126

CMPLX not

b1534 ydeE dipeptide exporter

b4599 mgtS small protein MgtS

//

>NGHB#1496 -1

CMPLX not

b4599 mgtS small protein MgtS

b4775 mgtT protein MgtT

//

>NGHB#1498 254

CMPLX not

b1536 ydeI BOF family protein YdeI

b1535 dgcZ diguanylate cyclase DgcZ

//

>NGHB#1502 88

CMPLX not

b1539 ydfG 3-hydroxy acid dehydrogenase YdfG

b1540 ydfH DNA-binding transcriptional repressor YdfH

//

>NGHB#1503 176

CMPLX not

b1540 ydfH DNA-binding transcriptional repressor YdfH

b1541 ydfZ putative selenoprotein YdfZ

//

>NGHB#1505 88

CMPLX not

b1543 ydfJ putative transporter YdfJ

b1542 ydfI putative oxidoreductase YdfI

//

>NGHB#1507 68

CMPLX not

b4748 ynfT Qin prophage; protein YnfT

b1544 ydfK Qin prophage; cold shock protein YdfK

//

>NGHB#1508 316

CMPLX not

b1544 ydfK Qin prophage; cold shock protein YdfK

b1545 pinQ Qin prophage; putative recombinase PinQ

//

>NGHB#1510 -1

CMPLX not

b1547 stfQ Qin prophage; putative prophage side tail fiberprotein StfQ

b1546 tfaQ Qin prophage; putative tail fiber assemblyprotein TfaQ

//

>NGHB#1511 -50

CMPLX not

b1548 nohA Qin prophage; putative prophage DNA-packagingprotein NohA

b1547 stfQ Qin prophage; putative prophage side tail fiberprotein StfQ

//

>NGHB#1513 57

CMPLX not

b4533 ynfO Qin prophage; DUF3950 domain-containing proteinYnfO

b1549 ydfO Qin prophage; DUF1398 domain-containing proteinYdfO

//

>NGHB#1515 171

CMPLX not

b1551 ynfN Qin prophage; protein YnfN

b1550 gnsB Qin prophage; protein GnsB

//

>NGHB#1516 78

CMPLX not

b4749 ynfR Qin prophage; protein YnfR

b1551 ynfN Qin prophage; protein YnfN

//

>NGHB#1517 2

CMPLX not

b4724 ynfQ Qin prophage; protein YnfQ

b4749 ynfR Qin prophage; protein YnfR

//

>NGHB#1518 10

CMPLX not

b1552 cspI Qin prophage; cold shock-like protein CspI

b4724 ynfQ Qin prophage; protein YnfQ

//

>NGHB#1519 362

CMPLX not

b1553 rzpQ Qin prophage; DUF2514 domain-containing proteinRzpQ

b1552 cspI Qin prophage; cold shock-like protein CspI

//

>NGHB#1520 -448

CMPLX not

b4689 rzoQ Qin prophage; putative lipoprotein RzoQ

b1553 rzpQ Qin prophage; DUF2514 domain-containing proteinRzpQ

//

>NGHB#1521 189

CMPLX not

b1554 rrrQ Qin prophage; putative lysozyme

b4689 rzoQ Qin prophage; putative lipoprotein RzoQ

//

>NGHB#1522 -4

CMPLX not

b1555 ydfR Qin prophage; protein YdfR

b1554 rrrQ Qin prophage; putative lysozyme

//

>NGHB#1523 4

CMPLX not

b1556 essQ Qin prophage; putative prophage lysis proteinEssQ

b1555 ydfR Qin prophage; protein YdfR

//

>NGHB#1527 54

CMPLX not

b1558 cspF Qin prophage; cold shock protein CspF

b4750 ynfS Qin prophage; protein YnfS

//

>NGHB#1529 13

CMPLX not

b1560 ydfU Qin prophage; protein YdfU

b1559 ydfT Qin prophage; putative antitermination proteinQ

//

>NGHB#1530 346

CMPLX not

b1561 rem Qin prophage; protein Rem

b1560 ydfU Qin prophage; protein YdfU

//

>NGHB#1531 216

CMPLX not

b1562 hokD Qin prophage; toxic protein HokD

b1561 rem Qin prophage; protein Rem

//

>NGHB#1532 71

CMPLX not

b1563 relE Qin prophage; mRNA interferase toxin RelE

b1562 hokD Qin prophage; toxic protein HokD

//

>NGHB#1533 -1

CMPLX yes

b1564 relB Qin prophage; antitoxin/DNA-bindingtranscriptional repressor RelB

b1563 relE Qin prophage; mRNA interferase toxin RelE

//

>NGHB#1535 202

CMPLX not

b1565 ydfV Qin prophage; protein YdfV

b1566 flxA Qin prophage; protein FlxA

//

>NGHB#1537 22

CMPLX ---

b1568 ydfX Qin prophage; uncharacterized protein YdfX

b1567 ydfW Qin prophage; uncharacterized protein YdfW

//

>NGHB#1538 -17

CMPLX ---

b1569 dicC Qin prophage; DNA-binding transcriptionalregulator for DicB

b1568 ydfX Qin prophage; uncharacterized protein YdfX

//

>NGHB#1540 166

CMPLX not

b1570 dicA DNA-binding transcriptional dual regulator DicA

b1571 ydfA Qin prophage; DUF1391 domain-containing proteinYdfA

//

>NGHB#1541 1

CMPLX ---

b1571 ydfA Qin prophage; DUF1391 domain-containing proteinYdfA

b1572 ydfB Qin prophage; uncharacterized protein YdfB

//

>NGHB#1542 29

CMPLX ---

b1572 ydfB Qin prophage; uncharacterized protein YdfB

b1573 ydfC Qin prophage; uncharacterized protein YdfC

//

>NGHB#1543 567

CMPLX ---

b1573 ydfC Qin prophage; uncharacterized protein YdfC

b1575 dicB Qin prophage; cell division inhibition proteinDicB

//

>NGHB#1544 -4

CMPLX not

b1575 dicB Qin prophage; cell division inhibition proteinDicB

b1576 ydfD Qin prophage; lysis protein

//

>NGHB#1546 57

CMPLX not

b1580 rspB putative zinc-binding dehydrogenase RspB

b4534 ynfP protein YnfP

//

>NGHB#1547 11

CMPLX not

b1581 rspA mandelate racemase/muconate lactonizing enzymefamily protein RspA

b1580 rspB putative zinc-binding dehydrogenase RspB

//

>NGHB#1548 205

CMPLX not

b1582 ynfA putative transporter YnfA

b1581 rspA mandelate racemase/muconate lactonizing enzymefamily protein RspA

//

>NGHB#1550 34

CMPLX not

b1583 ynfB DUF1283 domain-containing protein YnfB

b1584 speG spermidine N-acetyltransferase

//

>NGHB#1553 198

CMPLX not

b1586 ynfD DUF1161 domain-containing protein YnfD

b1587 ynfE putative selenate reductase YnfE

//

>NGHB#1554 60

CMPLX yes

b1587 ynfE putative selenate reductase YnfE

b1588 ynfF putative selenate reductase YnfF

//

>NGHB#1555 10

CMPLX not

b1588 ynfF putative selenate reductase YnfF

b1589 ynfG putative oxidoreductase YnfG

//

>NGHB#1556 1

CMPLX not

b1589 ynfG putative oxidoreductase YnfG

b1590 ynfH putative menaquinol dehydrogenase

//

>NGHB#1557 42

CMPLX not

b1590 ynfH putative menaquinol dehydrogenase

b1591 dmsD redox enzyme maturation protein DmsD

//

>NGHB#1558 194

CMPLX not

b1591 dmsD redox enzyme maturation protein DmsD

b1592 clcB putative chloride:H(+) antiporter ClcB

//

>NGHB#1560 124

CMPLX not

b1594 mlc DNA-binding transcriptional repressor Mlc

b1593 ynfK putative dethiobiotin synthetase

//

>NGHB#1561 134

CMPLX not

b1595 ynfL putative DNA-binding transcriptional regulator

b1594 mlc DNA-binding transcriptional repressor Mlc

//

>NGHB#1563 423

CMPLX not

b1596 ynfM putative transporter YnfM

b1597 asr acid shock protein

//

>NGHB#1564 92

CMPLX ---

b1597 asr acid shock protein

b4601 ydgU uncharacterized protein YdgU

//

>NGHB#1565 99

CMPLX ---

b4601 ydgU uncharacterized protein YdgU

b1598 ydgD putative serine protease YdgD

//

>NGHB#1567 -14

CMPLX yes

b1600 mdtJ multidrug/spermidine efflux pump membranesubunit MdtJ

b1599 mdtI multidrug/spermidine efflux pump membranesubunit MdtI

//

>NGHB#1568 106

CMPLX not

b4777 ydgV protein YdgV

b1600 mdtJ multidrug/spermidine efflux pump membranesubunit MdtJ

//

>NGHB#1571 10

CMPLX yes

b1603 pntA pyridine nucleotide transhydrogenase subunitalpha

b1602 pntB pyridine nucleotide transhydrogenase subunitbeta

//

>NGHB#1573 185

CMPLX not

b1604 ydgH DUF1471 domain-containing protein YdgH

b1605 ydgI putative arginine:ornithine antiporter

//

>NGHB#1574 36

CMPLX not

b1605 ydgI putative arginine:ornithine antiporter

b1606 folM dihydromonapterin reductase

//

>NGHB#1577 3

CMPLX not

b1608 rstA DNA-binding transcriptional regulator RstA

b1609 rstB sensory histidine kinase RstB

//

>NGHB#1578 75

CMPLX not

b1609 rstB sensory histidine kinase RstB

b1610 tus DNA replication terminus site-binding protein

//

>NGHB#1580 142

CMPLX yes

b1612 fumA fumarase A

b1611 fumC fumarase C

//

>NGHB#1582 100

CMPLX not

b1613 manA mannose-6-phosphate isomerase

b1614 ydgA DUF945 domain-containing protein YdgA

//

>NGHB#1584 38

CMPLX not

b1616 uidB glucuronide:H(+) symporter

b1615 uidC outer membrane porin family protein UidC

//

>NGHB#1585 -4

CMPLX not

b1617 uidA beta-glucuronidase

b1616 uidB glucuronide:H(+) symporter

//

>NGHB#1586 390

CMPLX not

b1618 uidR DNA-binding transcriptional repressor UidR

b1617 uidA beta-glucuronidase

//

>NGHB#1587 220

CMPLX not

b1619 hdhA 7-alpha-hydroxysteroid dehydrogenase

b1618 uidR DNA-binding transcriptional repressor UidR

//

>NGHB#1588 111

CMPLX not

b1620 malI DNA-binding transcriptional repressor MalI

b1619 hdhA 7-alpha-hydroxysteroid dehydrogenase

//

>NGHB#1590 9

CMPLX not

b1621 malX PTS enzyme IIBC component MalX

b1622 malY negative regulator of MalTactivity/cystathionine beta-lyase

//

>NGHB#1591 103

CMPLX not

b1622 malY negative regulator of MalTactivity/cystathionine beta-lyase

b1623 add adenosine deaminase

//

>NGHB#1594 272

CMPLX not

b4409 blr beta-lactam resistance protein

b1625 cnu H-NS- and StpA-binding protein

//

>NGHB#1595 85

CMPLX not

b1625 cnu H-NS- and StpA-binding protein

b1626 ydgK DUF2569 domain-containing inner membrane proteinYdgK

//

>NGHB#1596 76

CMPLX not

b1626 ydgK DUF2569 domain-containing inner membrane proteinYdgK

b1627 rsxA SoxR [2Fe-2S] reducing system protein RsxA

//

>NGHB#1597 -1

CMPLX yes

b1627 rsxA SoxR [2Fe-2S] reducing system protein RsxA

b1628 rsxB SoxR [2Fe-2S] reducing system protein RsxB

//

>NGHB#1598 -8

CMPLX yes

b1628 rsxB SoxR [2Fe-2S] reducing system protein RsxB

b1629 rsxC SoxR [2Fe-2S] reducing system protein RsxC

//

>NGHB#1599 0

CMPLX yes

b1629 rsxC SoxR [2Fe-2S] reducing system protein RsxC

b1630 rsxD SoxR [2Fe-2S] reducing system protein RsxD

//

>NGHB#1600 3

CMPLX yes

b1630 rsxD SoxR [2Fe-2S] reducing system protein RsxD

b1631 rsxG SoxR [2Fe-2S] reducing system protein RsxG

//

>NGHB#1601 3

CMPLX yes

b1631 rsxG SoxR [2Fe-2S] reducing system protein RsxG

b1632 rsxE SoxR [2Fe-2S] reducing system protein RsxE

//

>NGHB#1602 -1

CMPLX not

b1632 rsxE SoxR [2Fe-2S] reducing system protein RsxE

b1633 nth endonuclease III

//

>NGHB#1603 610

CMPLX not

b1633 nth endonuclease III

b1634 dtpA dipeptide/tripeptide:H(+) symporter DtpA

//

>NGHB#1604 105

CMPLX not

b1634 dtpA dipeptide/tripeptide:H(+) symporter DtpA

b1635 gstA glutathione S-transferase GstA

//

>NGHB#1606 58

CMPLX not

b1637 tyrS tyrosine--tRNA ligase

b1636 pdxY pyridoxal kinase 2

//

>NGHB#1607 128

CMPLX not

b1638 pdxH pyridoxine/pyridoxamine 5'-phosphate oxidase

b1637 tyrS tyrosine--tRNA ligase

//

>NGHB#1608 58

CMPLX not

b1639 mliC inhibitor of c-type lysozyme, putativelipoprotein

b1638 pdxH pyridoxine/pyridoxamine 5'-phosphate oxidase

//

>NGHB#1609 97

CMPLX not

b1640 anmK anhydro-N-acetylmuramic acid kinase

b1639 mliC inhibitor of c-type lysozyme, putativelipoprotein

//

>NGHB#1613 2

CMPLX not

b1643 ydhI DUF1656 domain-containing protein YdhI

b1644 ydhJ putative membrane fusion protein YdhJ

//

>NGHB#1614 -1

CMPLX not

b1644 ydhJ putative membrane fusion protein YdhJ

b1645 ydhK putative transporter YdhK

//

>NGHB#1616 80

CMPLX not

b1647 ydhF putative oxidoreductase YdhF

b1646 sodC superoxide dismutase (Cu-Zn)

//

>NGHB#1617 48

CMPLX not

b1648 ydhL DUF1289 domain-containing protein YdhL

b1647 ydhF putative oxidoreductase YdhF

//

>NGHB#1619 36

CMPLX not

b1649 nemR DNA-binding transcriptional repressor NemR

b1650 nemA N-ethylmaleimide reductase

//

>NGHB#1620 80

CMPLX not

b1650 nemA N-ethylmaleimide reductase

b1651 gloA glyoxalase I

//

>NGHB#1621 102

CMPLX not

b1651 gloA glyoxalase I

b1652 rnt RNase T

//

>NGHB#1622 92

CMPLX not

b1652 rnt RNase T

b1653 lhr putative ATP-dependent helicase Lhr

//

>NGHB#1625 127

CMPLX not

b1655 mepH peptidoglycan DD-endopeptidase MepH

b1656 sodB superoxide dismutase (Fe)

//

>NGHB#1627 165

CMPLX not

b4602 cydH cytochrome bd-I ubiquinol oxidase accessorysubunit CydH

b1657 ydhP putative transporter YdhP

//

>NGHB#1631 290

CMPLX not

b1660 ydhC putative transporter YdhC

b1661 cfa cyclopropane fatty acyl phospholipid synthase

//

>NGHB#1636 125

CMPLX not

b1667 ydhR putative monooxygenase YdhR

b1668 ydhS FAD/NAD(P) binding domain-containing proteinYdhS

//

>NGHB#1638 3

CMPLX ---

b1670 ydhU putative cytochrome YdhU

b1669 ydhT uncharacterized protein YdhT

//

>NGHB#1639 -4

CMPLX not

b1671 ydhX putative 4Fe-4S ferredoxin-like protein YdhX

b1670 ydhU putative cytochrome YdhU

//

>NGHB#1640 63

CMPLX ---

b1672 ydhW uncharacterized protein YdhW

b1671 ydhX putative 4Fe-4S ferredoxin-like protein YdhX

//

>NGHB#1641 3

CMPLX ---

b1673 ydhV putative oxidoreductase YdhV

b1672 ydhW uncharacterized protein YdhW

//

>NGHB#1642 20

CMPLX not

b1674 ydhY putative 4Fe-4S ferredoxin-like protein YdhY

b1673 ydhV putative oxidoreductase YdhV

//

>NGHB#1643 454

CMPLX not

b1675 fumD fumarase D

b1674 ydhY putative 4Fe-4S ferredoxin-like protein YdhY

//

>NGHB#1645 310

CMPLX not

b1676 pykF pyruvate kinase I

b1677 lpp murein lipoprotein

//

>NGHB#1647 148

CMPLX not

b1679 sufE sulfur carrier protein SufE

b1678 ldtE L,D-transpeptidase LdtE

//

>NGHB#1648 12

CMPLX not

b1680 sufS L-cysteine desulfurase

b1679 sufE sulfur carrier protein SufE

//

>NGHB#1649 -4

CMPLX not

b1681 sufD Fe-S cluster scaffold complex subunit SufD

b1680 sufS L-cysteine desulfurase

//

>NGHB#1650 -26

CMPLX yes

b1682 sufC Fe-S cluster scaffold complex subunit SufC

b1681 sufD Fe-S cluster scaffold complex subunit SufD

//

>NGHB#1651 9

CMPLX yes

b1683 sufB Fe-S cluster scaffold complex subunit SufB

b1682 sufC Fe-S cluster scaffold complex subunit SufC

//

>NGHB#1652 8

CMPLX yes

b1684 sufA iron-sulfur cluster insertion protein SufA

b1683 sufB Fe-S cluster scaffold complex subunit SufB

//

>NGHB#1653 547

CMPLX ---

b1685 ydiH uncharacterized protein YdiH

b1684 sufA iron-sulfur cluster insertion protein SufA

//

>NGHB#1654 99

CMPLX ---

b1686 menI 1,4-dihydroxy-2-naphthoyl-CoA hydrolase

b1685 ydiH uncharacterized protein YdiH

//

>NGHB#1655 -4

CMPLX not

b1687 ydiJ putative FAD-linked oxidoreductase

b1686 menI 1,4-dihydroxy-2-naphthoyl-CoA hydrolase

//

>NGHB#1657 428

CMPLX not

b1688 ydiK putative transporter YdiK

b1689 ydiL DUF1870 domain-containing protein YdiL

//

>NGHB#1658 99

CMPLX not

b1689 ydiL DUF1870 domain-containing protein YdiL

b1690 ydiM putative exporter YdiM

//

>NGHB#1659 226

CMPLX not

b1690 ydiM putative exporter YdiM

b1691 ydiN putative transporter YdiN

//

>NGHB#1660 11

CMPLX not

b1691 ydiN putative transporter YdiN

b1692 ydiB quinate/shikimate dehydrogenase

//

>NGHB#1661 30

CMPLX not

b1692 ydiB quinate/shikimate dehydrogenase

b1693 aroD 3-dehydroquinate dehydratase

//

>NGHB#1662 142

CMPLX not

b1693 aroD 3-dehydroquinate dehydratase

b1694 ydiF putative acetate-CoA transferase YdiF

//

>NGHB#1663 13

CMPLX not

b1694 ydiF putative acetate-CoA transferase YdiF

b1695 ydiO putative acyl-CoA dehydrogenase YdiO

//

>NGHB#1666 19

CMPLX yes

b1697 ydiQ putative electron transfer flavoprotein subunitYdiQ

b1698 ydiR putative electron transfer flavoprotein subunitYdiR

//

>NGHB#1667 55

CMPLX yes

b1698 ydiR putative electron transfer flavoprotein subunitYdiR

b1699 ydiS putative electron transfer flavoprotein-quinoneoxidoreductase YdiS

//

>NGHB#1668 -4

CMPLX not

b1699 ydiS putative electron transfer flavoprotein-quinoneoxidoreductase YdiS

b1700 ydiT ferredoxin-like protein YdiT

//

>NGHB#1669 2

CMPLX not

b1700 ydiT ferredoxin-like protein YdiT

b1701 fadK short chain acyl-CoA synthetase

//

>NGHB#1672 156

CMPLX not

b1703 ppsR phosphoenolpyruvate synthetase regulatoryprotein

b1704 aroH 3-deoxy-7-phosphoheptulonate synthase,Trp-sensitive

//

>NGHB#1673 131

CMPLX not

b1704 aroH 3-deoxy-7-phosphoheptulonate synthase,Trp-sensitive

b1705 ydiE PF10636 family protein YdiE

//

>NGHB#1675 62

CMPLX not

b1707 rflP anti-FlhDC factor RflP

b1706 ydiU UPF0061 family protein YdiU

//

>NGHB#1676 246

CMPLX not

b1708 nlpC NlpC/P60 family lipoprotein NlpC

b1707 rflP anti-FlhDC factor RflP

//

>NGHB#1677 77

CMPLX not

b1709 btuD vitamin B12 ABC transporter ATP binding subunit

b1708 nlpC NlpC/P60 family lipoprotein NlpC

//

>NGHB#1678 -1

CMPLX not

b1710 btuE thioredoxin/glutathione peroxidase

b1709 btuD vitamin B12 ABC transporter ATP binding subunit

//

>NGHB#1679 62

CMPLX not

b1711 btuC vitamin B12 ABC transporter membrane subunit

b1710 btuE thioredoxin/glutathione peroxidase

//

>NGHB#1680 100

CMPLX not

b1712 ihfA integration host factor subunit alpha

b1711 btuC vitamin B12 ABC transporter membrane subunit

//

>NGHB#1681 4

CMPLX not

b1713 pheT phenylalanine--tRNA ligase subunit beta

b1712 ihfA integration host factor subunit alpha

//

>NGHB#1682 14

CMPLX yes

b1714 pheS phenylalanine--tRNA ligase subunit alpha

b1713 pheT phenylalanine--tRNA ligase subunit beta

//

>NGHB#1683 283

CMPLX not

b1715 pheM pheST-ihfA operon leader peptide

b1714 pheS phenylalanine--tRNA ligase subunit alpha

//

>NGHB#1684 122

CMPLX not

b1716 rplT 50S ribosomal subunit protein L20

b1715 pheM pheST-ihfA operon leader peptide

//

>NGHB#1685 52

CMPLX yes

b1717 rpmI 50S ribosomal subunit protein L35

b1716 rplT 50S ribosomal subunit protein L20

//

>NGHB#1686 96

CMPLX not

b1718 infC translation initiation factor IF-3

b1717 rpmI 50S ribosomal subunit protein L35

//

>NGHB#1687 3

CMPLX not

b1719 thrS threonine--tRNA ligase

b1718 infC translation initiation factor IF-3

//

>NGHB#1691 100

CMPLX not

b1723 pfkB 6-phosphofructokinase 2

b1724 ydiZ putative endoribonuclease YdiZ

//

>NGHB#1692 105

CMPLX not

b1724 ydiZ putative endoribonuclease YdiZ

b1725 yniA putative kinase YniA

//

>NGHB#1695 162

CMPLX not

b1727 hxpB hexitol phosphatase B

b1728 ydjM inner membrane protein YdjM

//

>NGHB#1696 132

CMPLX not

b1728 ydjM inner membrane protein YdjM

b1729 tcyP cystine/sulfocysteine:cation symporter

//

>NGHB#1698 288

CMPLX not

b1731 cedA cell division modulator

b1730 ydjO protein YdjO

//

>NGHB#1701 12

CMPLX not

b1734 chbF monoacetylchitobiose-6-phosphate hydrolase

b1733 chbG chitin disaccharide deacetylase

//

>NGHB#1702 104

CMPLX not

b1735 chbR DNA-binding transcriptional dual regulator ChbR

b1734 chbF monoacetylchitobiose-6-phosphate hydrolase

//

>NGHB#1703 7

CMPLX not

b1736 chbA N,N'-diacetylchitobiose-specific PTS enzyme IIAcomponent

b1735 chbR DNA-binding transcriptional dual regulator ChbR

//

>NGHB#1704 50

CMPLX yes

b1737 chbC N,N'-diacetylchitobiose-specific PTS enzyme IICcomponent

b1736 chbA N,N'-diacetylchitobiose-specific PTS enzyme IIAcomponent

//

>NGHB#1705 84

CMPLX yes

b1738 chbB N,N'-diacetylchitobiose-specific PTS enzyme IIBcomponent

b1737 chbC N,N'-diacetylchitobiose-specific PTS enzyme IICcomponent

//

>NGHB#1706 298

CMPLX not

b1739 osmE osmotically-inducible lipoprotein OsmE

b1738 chbB N,N'-diacetylchitobiose-specific PTS enzyme IIBcomponent

//

>NGHB#1708 229

CMPLX not

b1740 nadE NH3-dependent NAD(+) synthetase

b1741 cho excinuclease Cho

//

>NGHB#1710 202

CMPLX not

b1743 spy ATP-independent periplasmic chaperone

b1742 ves HutD family protein Ves

//

>NGHB#1711 329

CMPLX not

b1744 astE succinylglutamate desuccinylase

b1743 spy ATP-independent periplasmic chaperone

//

>NGHB#1712 -8

CMPLX not

b1745 astB N-succinylarginine dihydrolase

b1744 astE succinylglutamate desuccinylase

//

>NGHB#1713 -4

CMPLX not

b1746 astD aldehyde dehydrogenase

b1745 astB N-succinylarginine dihydrolase

//

>NGHB#1714 -4

CMPLX not

b1747 astA arginine N-succinyltransferase

b1746 astD aldehyde dehydrogenase

//

>NGHB#1715 -4

CMPLX not

b1748 astC succinylornithine transaminase

b1747 astA arginine N-succinyltransferase

//

>NGHB#1717 166

CMPLX not

b1749 xthA exodeoxyribonuclease III

b1750 ydjX DedA family protein YdjX

//

>NGHB#1718 4

CMPLX not

b1750 ydjX DedA family protein YdjX

b1751 ydjY 4Fe-4S ferredoxin-type domain-containing proteinYdjY

//

>NGHB#1719 14

CMPLX not

b1751 ydjY 4Fe-4S ferredoxin-type domain-containing proteinYdjY

b1752 ydjZ DedA family protein YdjZ

//

>NGHB#1720 -1

CMPLX not

b1752 ydjZ DedA family protein YdjZ

b1753 ynjA AhpD-like domain-containing protein YnjA

//

>NGHB#1721 9

CMPLX not

b1753 ynjA AhpD-like domain-containing protein YnjA

b1754 ynjB putative ABC transporter periplasmic bindingprotein YnjB

//

>NGHB#1722 -28

CMPLX yes

b1754 ynjB putative ABC transporter periplasmic bindingprotein YnjB

b1755 ynjC putative ABC transporter membrane subunit YnjC

//

>NGHB#1723 -1

CMPLX yes

b1755 ynjC putative ABC transporter membrane subunit YnjC

b1756 ynjD putative ABC transporter ATP-binding proteinYnjD

//

>NGHB#1724 66

CMPLX not

b1756 ynjD putative ABC transporter ATP-binding proteinYnjD

b1757 ynjE molybdopterin synthase sulfurtransferase

//

>NGHB#1730 127

CMPLX not

b1763 topB DNA topoisomerase III

b1762 ynjI DUF1266 domain-containing protein YnjI

//

>NGHB#1731 4

CMPLX not

b1764 selD selenide, water dikinase

b1763 topB DNA topoisomerase III

//

>NGHB#1732 116

CMPLX not

b1765 ydjA putative oxidoreductase YdjA

b1764 selD selenide, water dikinase

//

>NGHB#1734 166

CMPLX not

b1766 sppA protease IV, a signal peptide peptidase

b1767 ansA L-asparaginase 1

//

>NGHB#1735 10

CMPLX not

b1767 ansA L-asparaginase 1

b1768 pncA nicotinamidase

//

>NGHB#1737 116

CMPLX not

b1770 ydjF putative DNA-binding transcriptional regulatorYdjF

b1769 ydjE putative transporter YdjE

//

>NGHB#1738 136

CMPLX not

b1771 ydjG NADH-dependent methylglyoxal reductase

b1770 ydjF putative DNA-binding transcriptional regulatorYdjF

//

>NGHB#1739 9

CMPLX not

b1772 ydjH L-glycero-L-galacto-octuluronate kinase

b1771 ydjG NADH-dependent methylglyoxal reductase

//

>NGHB#1740 4

CMPLX not

b1773 ydjI L-glycero-L-galacto-octuluronate-1-phosphatealdolase

b1772 ydjH L-glycero-L-galacto-octuluronate kinase

//

>NGHB#1741 20

CMPLX not

b1774 ydjJ putative zinc-binding dehydrogenase YdjJ

b1773 ydjI L-glycero-L-galacto-octuluronate-1-phosphatealdolase

//

>NGHB#1742 16

CMPLX not

b1775 ydjK putative transporter YdjK

b1774 ydjJ putative zinc-binding dehydrogenase YdjJ

//

>NGHB#1743 26

CMPLX not

b1776 ydjL putative zinc-binding dehydrogenase YdjL

b1775 ydjK putative transporter YdjK

//

>NGHB#1744 369

CMPLX not

b1777 yeaC DUF1315 domain-containing protein YeaC

b1776 ydjL putative zinc-binding dehydrogenase YdjL

//

>NGHB#1745 41

CMPLX not

b1778 msrB methionine sulfoxide reductase B

b1777 yeaC DUF1315 domain-containing protein YeaC

//

>NGHB#1747 83

CMPLX not

b1779 gapA glyceraldehyde-3-phosphate dehydrogenase A

b1780 yeaD putative aldose 1-epimerase YeaD

//

>NGHB#1749 89

CMPLX not

b1782 mipA MltA-interacting protein

b1781 yeaE methylglyoxal reductase YeaE

//

>NGHB#1751 112

CMPLX not

b1783 yeaG protein kinase YeaG

b1784 yeaH DUF444 domain-containing protein YeaH

//

>NGHB#1752 146

CMPLX not

b1784 yeaH DUF444 domain-containing protein YeaH

b1785 cdgI putative c-di-GMP binding protein CdgI

//

>NGHB#1753 180

CMPLX not

b1785 cdgI putative c-di-GMP binding protein CdgI

b1786 dgcJ putative diguanylate cyclase DgcJ

//

>NGHB#1754 42

CMPLX not

b1786 dgcJ putative diguanylate cyclase DgcJ

b1787 yeaK mischarged aminoacyl-tRNA deacylase

//

>NGHB#1759 54

CMPLX not

b1791 nimT 2-nitroimidazole exporter

b1792 yeaO DUF488 domain-containing protein YeaO

//

>NGHB#1762 32

CMPLX not

b1794 dgcP diguanylate cyclase DgcP

b4676 yoaK putative membrane protein YoaK

//

>NGHB#1763 2

CMPLX ---

b4676 yoaK putative membrane protein YoaK

b4675 yoaJ uncharacterized protein YoaJ

//

>NGHB#1765 147

CMPLX not

b1796 yoaG DUF1869 domain-containing protein YoaG

b1795 yeaQ PF04226 family protein YeaQ

//

>NGHB#1766 3

CMPLX not

b1797 yeaR DUF1971 domain-containing protein YeaR

b1796 yoaG DUF1869 domain-containing protein YoaG

//

>NGHB#1767 172

CMPLX not

b1798 leuE leucine exporter

b1797 yeaR DUF1971 domain-containing protein YeaR

//

>NGHB#1768 126

CMPLX not

b1799 dmlR DNA-binding transcriptional regulator DmlR

b1798 leuE leucine exporter

//

>NGHB#1770 190

CMPLX not

b1800 dmlA D-malate/3-isopropylmalate dehydrogenase(decarboxylating)

b1801 yeaV putative transporter YeaV

//

>NGHB#1771 31

CMPLX not

b1801 yeaV putative transporter YeaV

b1802 yeaW carnitine monooxygenase subunit YeaW

//

>NGHB#1772 55

CMPLX yes

b1802 yeaW carnitine monooxygenase subunit YeaW

b1803 yeaX carnitine monooxygenase subunit YeaX

//

>NGHB#1774 69

CMPLX not

b1805 fadD long-chain-fatty-acid--CoA ligase

b1804 rnd RNase D

//

>NGHB#1775 204

CMPLX not

b1806 yeaY Slp family lipoprotein YeaY

b1805 fadD long-chain-fatty-acid--CoA ligase

//

>NGHB#1776 39

CMPLX not

b1807 tsaB N(6)-L-threonylcarbamoyladenine synthase, TsaBsubunit

b1806 yeaY Slp family lipoprotein YeaY

//

>NGHB#1777 57

CMPLX not

b1808 yoaA putative ATP-dependent 5' to 3' DNA helicase

b1807 tsaB N(6)-L-threonylcarbamoyladenine synthase, TsaBsubunit

//

>NGHB#1779 421

CMPLX not

b1809 yoaB RutC family protein YoaB

b1810 yoaC DUF1889 domain-containing protein YoaC

//

>NGHB#1782 3

CMPLX not

b1812 pabB aminodeoxychorismate synthase subunit 1

b1813 nudL putative NUDIX hydrolase with low3-phosphohydroxypyruvate phosphatase activity

//

>NGHB#1783 183

CMPLX not

b1813 nudL putative NUDIX hydrolase with low3-phosphohydroxypyruvate phosphatase activity

b1814 sdaA L-serine deaminase I

//

>NGHB#1784 130

CMPLX not

b1814 sdaA L-serine deaminase I

b1815 pdeD putative c-di-GMP phosphodiesterase PdeD

//

>NGHB#1786 -13

CMPLX not

b4751 yoaL protein YoaL

b1816 yoaE putative inner membrane protein YoaE

//

>NGHB#1788 62

CMPLX yes

b1817 manX mannose-specific PTS enzyme IIAB component

b1818 manY mannose-specific PTS enzyme IIC component

//

>NGHB#1789 12

CMPLX yes

b1818 manY mannose-specific PTS enzyme IIC component

b1819 manZ mannose-specific PTS enzyme IID component

//

>NGHB#1790 54

CMPLX not

b1819 manZ mannose-specific PTS enzyme IID component

b1820 yobD DUF986 domain-containing inner membrane proteinYobD

//

>NGHB#1791 428

CMPLX not

b1820 yobD DUF986 domain-containing inner membrane proteinYobD

b1821 mntP Mn(2(+)) exporter

//

>NGHB#1793 165

CMPLX not

b1823 cspC CspA family stress protein CspC

b1822 rlmA 23S rRNA m(1)G745 methyltransferase

//

>NGHB#1794 12

CMPLX not

b1824 yobF DUF2527 domain-containing protein YobF

b1823 cspC CspA family stress protein CspC

//

>NGHB#1795 669

CMPLX ---

b1825 yebO uncharacterized protein YebO

b1824 yobF DUF2527 domain-containing protein YobF

//

>NGHB#1796 74

CMPLX ---

b1826 mgrB PhoQ kinase inhibitor

b1825 yebO uncharacterized protein YebO

//

>NGHB#1801 191

CMPLX not

b1830 prc tail-specific protease

b1829 htpX protease HtpX

//

>NGHB#1802 19

CMPLX not

b1831 proQ RNA chaperone ProQ

b1830 prc tail-specific protease

//

>NGHB#1803 96

CMPLX not

b1832 msrC free methionine-(R)-sulfoxide reductase

b1831 proQ RNA chaperone ProQ

//

>NGHB#1805 -32

CMPLX not

b1833 letA intermembrane transport protein LetA

b1834 letB lipophilic envelope spanning tunnel

//

>NGHB#1806 79

CMPLX not

b1834 letB lipophilic envelope spanning tunnel

b1835 rsmF 16S rRNA m(5)C1407 methyltransferase

//

>NGHB#1807 117

CMPLX not

b1835 rsmF 16S rRNA m(5)C1407 methyltransferase

b1836 yebV DUF1480 domain-containing protein YebV

//

>NGHB#1808 104

CMPLX not

b1836 yebV DUF1480 domain-containing protein YebV

b1837 yebW DUF1482 domain-containing protein YebW

//

>NGHB#1810 395

CMPLX not

b1839 yebY DUF2511 domain-containing protein YebY

b1838 pphA phosphoprotein phosphatase 1

//

>NGHB#1811 12

CMPLX not

b1840 yebZ putative inner membrane protein

b1839 yebY DUF2511 domain-containing protein YebY

//

>NGHB#1812 3

CMPLX not

b1841 yobA CopC domain-containing protein

b1840 yebZ putative inner membrane protein

//

>NGHB#1814 101

CMPLX not

b1842 holE DNA polymerase III subunit theta

b1843 yobB putative carbon-nitrogen hydrolase familyprotein YobB

//

>NGHB#1815 23

CMPLX not

b1843 yobB putative carbon-nitrogen hydrolase familyprotein YobB

b1844 exoX exonuclease X

//

>NGHB#1817 208

CMPLX not

b1846 yebE DUF533 domain-containing inner membrane proteinYebE

b1845 ptrB oligopeptidase B

//

>NGHB#1818 326

CMPLX not

b1847 yebF secreted protein YebF

b1846 yebE DUF533 domain-containing inner membrane proteinYebE

//

>NGHB#1819 66

CMPLX not

b1848 yebG DNA damage-inducible protein YebG

b1847 yebF secreted protein YebF

//

>NGHB#1822 36

CMPLX not

b1851 edd phosphogluconate dehydratase

b1850 eda KHG/KDPG aldolase

//

>NGHB#1823 234

CMPLX not

b1852 zwf NADP(+)-dependent glucose-6-phosphatedehydrogenase

b1851 edd phosphogluconate dehydratase

//

>NGHB#1825 127

CMPLX not

b1853 yebK DNA-binding transcriptional repressor YebK

b1854 pykA pyruvate kinase II

//

>NGHB#1827 119

CMPLX not

b1856 mepM peptidoglycan DD-endopeptidase MepM

b1855 lpxM Lipid A biosynthesis myristoyltransferase

//

>NGHB#1828 15

CMPLX not

b1857 znuA Zn(2(+)) ABC transporter periplasmic bindingprotein

b1856 mepM peptidoglycan DD-endopeptidase MepM

//

>NGHB#1830 -4

CMPLX yes

b1858 znuC Zn(2(+)) ABC transporter ATP binding subunit

b1859 znuB Zn(2(+)) ABC transporter membrane subunit

//

>NGHB#1832 8

CMPLX yes

b1861 ruvA Holliday junction branch migration complexsubunit RuvA

b1860 ruvB Holliday junction branch migration complexsubunit RuvB

//

>NGHB#1833 138

CMPLX ---

b4677 yobI uncharacterized protein YobI

b1861 ruvA Holliday junction branch migration complexsubunit RuvA

//

>NGHB#1836 34

CMPLX not

b1864 yebC putative transcriptional regulator YebC

b1863 ruvC crossover junction endodeoxyribonuclease RuvC

//

>NGHB#1837 28

CMPLX not

b1865 nudB dihydroneopterin triphosphate diphosphatase

b1864 yebC putative transcriptional regulator YebC

//

>NGHB#1838 117

CMPLX not

b1866 aspS aspartate--tRNA ligase

b1865 nudB dihydroneopterin triphosphate diphosphatase

//

>NGHB#1840 -4

CMPLX not

b1867 yecD isochorismatase family protein YecD

b1868 yecE DUF72 domain-containing protein YecE

//

>NGHB#1841 52

CMPLX not

b1868 yecE DUF72 domain-containing protein YecE

b1869 yecN MAPEG family inner membrane protein YecN

//

>NGHB#1842 40

CMPLX not

b1869 yecN MAPEG family inner membrane protein YecN

b1870 cmoA carboxy-S-adenosyl-L-methionine synthase

//

>NGHB#1843 -4

CMPLX not

b1870 cmoA carboxy-S-adenosyl-L-methionine synthase

b1871 cmoB tRNA U34 carboxymethyltransferase

//

>NGHB#1845 24

CMPLX not

b1873 torY cytochrome c quinol dehydrogenase TorY

b1872 torZ trimethylamine N-oxide reductase 2

//

>NGHB#1846 387

CMPLX not

b1874 cutC protein CutC

b1873 torY cytochrome c quinol dehydrogenase TorY

//

>NGHB#1847 13

CMPLX not

b1875 yecM putative metal-binding enzyme YecM

b1874 cutC protein CutC

//

>NGHB#1852 -1

CMPLX not

b1879 flhA flagellar biosynthesis protein FlhA

b1878 flhE flagellar protein FlhE

//

>NGHB#1853 -8

CMPLX yes

b1880 flhB flagellar biosynthesis protein FlhB

b1879 flhA flagellar biosynthesis protein FlhA

//

>NGHB#1854 201

CMPLX not

b1881 cheZ chemotaxis protein CheZ

b1880 flhB flagellar biosynthesis protein FlhB

//

>NGHB#1855 10

CMPLX yes

b1882 cheY chemotaxis protein CheY

b1881 cheZ chemotaxis protein CheZ

//

>NGHB#1856 14

CMPLX not

b1883 cheB protein-glutamate methylesterase/proteinglutamine deamidase

b1882 cheY chemotaxis protein CheY

//

>NGHB#1857 2

CMPLX not

b1884 cheR chemotaxis protein methyltransferase

b1883 cheB protein-glutamate methylesterase/proteinglutamine deamidase

//

>NGHB#1858 18

CMPLX not

b1885 tap methyl-accepting chemotaxis protein Tap

b1884 cheR chemotaxis protein methyltransferase

//

>NGHB#1859 45

CMPLX yes

b1886 tar methyl-accepting chemotaxis protein Tar

b1885 tap methyl-accepting chemotaxis protein Tap

//

>NGHB#1860 144

CMPLX not

b1887 cheW chemotaxis protein CheW

b1886 tar methyl-accepting chemotaxis protein Tar

//

>NGHB#1861 20

CMPLX yes

b1888 cheA chemotaxis protein CheA

b1887 cheW chemotaxis protein CheW

//

>NGHB#1862 295

CMPLX not

b1889 motB motility protein B

b1888 cheA chemotaxis protein CheA

//

>NGHB#1863 -4

CMPLX yes

b1890 motA motility protein A

b1889 motB motility protein B

//

>NGHB#1864 126

CMPLX not

b1891 flhC DNA-binding transcriptional dual regulator FlhC

b1890 motA motility protein A

//

>NGHB#1865 2

CMPLX yes

b1892 flhD DNA-binding transcriptional dual regulator FlhD

b1891 flhC DNA-binding transcriptional dual regulator FlhC

//

>NGHB#1866 320

CMPLX not

b1893 insB5 IS1 protein InsB

b1892 flhD DNA-binding transcriptional dual regulator FlhD

//

>NGHB#1867 -82

CMPLX tnp

b1894 insA5 IS1 protein InsA

b1893 insB5 IS1 protein InsB

//

>NGHB#1870 -26

CMPLX not

b1897 otsB trehalose-6-phosphate phosphatase

b1896 otsA trehalose-6-phosphate synthase

//

>NGHB#1871 166

CMPLX not

b4460 araH arabinose ABC transporter membrane subunit

b1897 otsB trehalose-6-phosphate phosphatase

//

>NGHB#1872 14

CMPLX yes

b1900 araG arabinose ABC transporter ATP binding subunit

b4460 araH arabinose ABC transporter membrane subunit

//

>NGHB#1873 69

CMPLX yes

b1901 araF arabinose ABC transporter periplasmic bindingprotein

b1900 araG arabinose ABC transporter ATP binding subunit

//

>NGHB#1876 114

CMPLX ---

b4663 azuC uncharacterized protein AzuC

b4537 yecJ DUF2766 domain-containing protein YecJ

//

>NGHB#1878 170

CMPLX not

b1904 yecR lipoprotein YecR

b1905 ftnA ferritin iron storage protein

//

>NGHB#1882 649

CMPLX not

b1912 pgsA CDP-diacylglycerol--glycerol-3-phosphate3-phosphatidyltransferase

b1908 yecA UPF0149 family protein YecA

//

>NGHB#1883 56

CMPLX not

b1913 uvrC excision nuclease subunit C

b1912 pgsA CDP-diacylglycerol--glycerol-3-phosphate3-phosphatidyltransferase

//

>NGHB#1884 -4

CMPLX not

b1914 uvrY DNA-binding transcriptional activator UvrY

b1913 uvrC excision nuclease subunit C

//

>NGHB#1886 -14

CMPLX not

b4752 yecU protein YecU

b1915 yecF DUF2594 domain-containing protein YecF

//

>NGHB#1888 229

CMPLX not

b1917 tcyN cystine ABC transporter ATP binding subunit

b1916 sdiA DNA-binding transcriptional dual regulator SdiA

//

>NGHB#1889 -4

CMPLX yes

b1918 tcyL cystine ABC transporter membrane subunit

b1917 tcyN cystine ABC transporter ATP binding subunit

//

>NGHB#1890 14

CMPLX not

b1919 dcyD D-cysteine desulfhydrase

b1918 tcyL cystine ABC transporter membrane subunit

//

>NGHB#1891 104

CMPLX not

b1920 tcyJ cystine ABC transporter periplasmic bindingprotein

b1919 dcyD D-cysteine desulfhydrase

//

>NGHB#1892 87

CMPLX not

b1921 fliZ DNA-binding transcriptional regulator FliZ

b1920 tcyJ cystine ABC transporter periplasmic bindingprotein

//

>NGHB#1893 45

CMPLX not

b1922 fliA RNA polymerase, sigma 28 (sigma F) factor

b1921 fliZ DNA-binding transcriptional regulator FliZ

//

>NGHB#1894 320

CMPLX not

b1923 fliC flagellar filament structural protein

b1922 fliA RNA polymerase, sigma 28 (sigma F) factor

//

>NGHB#1896 24

CMPLX not

b1924 fliD flagellar filament capping protein

b1925 fliS flagellar biosynthesis protein FliS

//

>NGHB#1897 -1

CMPLX yes

b1925 fliS flagellar biosynthesis protein FliS

b1926 fliT flagellar biosynthesis protein FliT

//

>NGHB#1898 77

CMPLX not

b1926 fliT flagellar biosynthesis protein FliT

b1927 amyA alpha-amylase

//

>NGHB#1901 -4

CMPLX not

b1929 yedE putative selenium transporter YedE

b1930 yedF putative sulfurtransferase YedF

//

>NGHB#1902 108

CMPLX not

b1930 yedF putative sulfurtransferase YedF

b1931 yedK genome maintenance protein

//

>NGHB#1903 110

CMPLX not

b1931 yedK genome maintenance protein

b1932 yedL putative acetyltransferase YedL

//

>NGHB#1906 -8

CMPLX not

b1938 fliF flagellar basal-body MS-ring and collar protein

b1939 fliG flagellar motor switch protein FliG

//

>NGHB#1907 -8

CMPLX not

b1939 fliG flagellar motor switch protein FliG

b1940 fliH flagellar biosynthesis protein FliH

//

>NGHB#1908 -1

CMPLX not

b1940 fliH flagellar biosynthesis protein FliH

b1941 fliI flagellum-specific ATP synthase FliI

//

>NGHB#1909 18

CMPLX not

b1941 fliI flagellum-specific ATP synthase FliI

b1942 fliJ flagellar biosynthesis protein FliJ

//

>NGHB#1910 -4

CMPLX not

b1942 fliJ flagellar biosynthesis protein FliJ

b1943 fliK flagellar hook-length control protein

//

>NGHB#1911 104

CMPLX not

b1943 fliK flagellar hook-length control protein

b1944 fliL flagellar protein FliL

//

>NGHB#1912 4

CMPLX not

b1944 fliL flagellar protein FliL

b1945 fliM flagellar motor switch protein FliM

//

>NGHB#1913 -4

CMPLX yes

b1945 fliM flagellar motor switch protein FliM

b1946 fliN flagellar motor switch protein FliN

//

>NGHB#1914 2

CMPLX not

b1946 fliN flagellar motor switch protein FliN

b1947 fliO flagellar biosynthesis protein FliO

//

>NGHB#1915 -1

CMPLX not

b1947 fliO flagellar biosynthesis protein FliO

b1948 fliP flagellar biosynthesis protein FliP

//

>NGHB#1916 9

CMPLX not

b1948 fliP flagellar biosynthesis protein FliP

b1949 fliQ flagellar biosynthesis protein FliQ

//

>NGHB#1917 7

CMPLX not

b1949 fliQ flagellar biosynthesis protein FliQ

b1950 fliR flagellar biosynthesis protein FliR

//

>NGHB#1918 289

CMPLX not

b1950 fliR flagellar biosynthesis protein FliR

b1951 rcsA DNA-binding transcriptional activator RcsA

//

>NGHB#1921 297

CMPLX not

b1953 yodD stress-induced protein

b1955 yedP putative mannosyl-3-phosphoglyceratephosphatase

//

>NGHB#1923 170

CMPLX not

b1957 yodC protein YodC

b1956 dgcQ putative diguanylate cyclase DgcQ

//

>NGHB#1924 78

CMPLX not

b1958 yedI DUF808 domain-containing inner membrane proteinYedI

b1957 yodC protein YodC

//

>NGHB#1927 -20

CMPLX not

b1961 dcm DNA-cytosine methyltransferase

b1960 vsr DNA mismatch endonuclease Vsr

//

>NGHB#1928 66

CMPLX not

b1962 yedJ putative HD superfamily phosphohydrolase YedJ

b1961 dcm DNA-cytosine methyltransferase

//

>NGHB#1929 39

CMPLX not

b1963 yedR putative inner membrane protein YedR

b1962 yedJ putative HD superfamily phosphohydrolase YedJ

//

>NGHB#1932 -1

CMPLX not

b1969 hprR DNA-binding transcriptional dual regulator HprR

b1968 hprS sensor histidine kinase HprS

//

>NGHB#1934 108

CMPLX not

b1970 hiuH hydroxyisourate hydrolase/transthyretin-relatedprotein

b1971 msrP protein-L-methionine sulfoxide reductasecatalytic subunit MsrP

//

>NGHB#1935 0

CMPLX yes

b1971 msrP protein-L-methionine sulfoxide reductasecatalytic subunit MsrP

b1972 msrQ protein-L-methionine sulfoxide reductaseheme-binding subunit MsrQ

//

>NGHB#1936 256

CMPLX not

b1972 msrQ protein-L-methionine sulfoxide reductaseheme-binding subunit MsrQ

b1973 zinT metal-binding protein ZinT

//

>NGHB#1937 342

CMPLX not

b1973 zinT metal-binding protein ZinT

b1974 yodB putative cytochrome b561 YodB

//

>NGHB#1938 752

CMPLX not

b1974 yodB putative cytochrome b561 YodB

b1976 mtfA Mlc titration factor

//

>NGHB#1939 489

CMPLX not

b1976 mtfA Mlc titration factor

b1978 yeeJ inverse autotransporter adhesin

//

>NGHB#1940 1628

CMPLX not

b1978 yeeJ inverse autotransporter adhesin

b1981 shiA shikimate:H(+) symporter

//

>NGHB#1941 101

CMPLX not

b1981 shiA shikimate:H(+) symporter

b1982 amn AMP nucleosidase

//

>NGHB#1942 342

CMPLX not

b1982 amn AMP nucleosidase

b1983 yeeN putative transcriptional regulator YeeN

//

>NGHB#1944 117

CMPLX not

b1987 cbl DNA-binding transcriptional activator Cbl

b1985 yeeO FMN/FAD exporter

//

>NGHB#1945 101

CMPLX not

b1988 nac DNA-binding transcriptional dual regulator Nac

b1987 cbl DNA-binding transcriptional activator Cbl

//

>NGHB#1946 457

CMPLX not

b1990 ldtA L,D-transpeptidase LdtA

b1988 nac DNA-binding transcriptional dual regulator Nac

//

>NGHB#1947 64

CMPLX not

b1991 cobT nicotinate-nucleotide--dimethylbenzimidazolephosphoribosyltransferase

b1990 ldtA L,D-transpeptidase LdtA

//

>NGHB#1948 11

CMPLX not

b1992 cobS cobalamin 5'-phosphate synthase

b1991 cobT nicotinate-nucleotide--dimethylbenzimidazolephosphoribosyltransferase

//

>NGHB#1949 -4

CMPLX not

b1993 cobU cobinamide-P guanylyltransferase/cobinamidekinase

b1992 cobS cobalamin 5'-phosphate synthase

//

>NGHB#1950 540

CMPLX not

b1994 insH6 CP4-44 prophage; IS5 transposase andtrans-activator

b1993 cobU cobinamide-P guanylyltransferase/cobinamidekinase

//

>NGHB#1952 -43

CMPLX tnp

b1997 insC3 CP4-44 prophage; IS2 insertion element repressorInsA

b1996 insD3 CP4-44 prophage; IS2 insertion element proteinInsB

//

>NGHB#1953 120

CMPLX not

b2000 flu CP4-44 prophage; self recognizing antigen 43(Ag43) autotransporter

b2001 yeeR CP4-44 prophage; inner membrane protein YeeR

//

>NGHB#1954 -4

CMPLX not

b2001 yeeR CP4-44 prophage; inner membrane protein YeeR

b2002 yeeS CP4-44 prophage; RadC-like JAB domain-containingprotein YeeS

//

>NGHB#1955 62

CMPLX not

b2002 yeeS CP4-44 prophage; RadC-like JAB domain-containingprotein YeeS

b2003 yeeT CP4-44 prophage; DUF987 domain-containingprotein YeeT

//

>NGHB#1956 73

CMPLX not

b2003 yeeT CP4-44 prophage; DUF987 domain-containingprotein YeeT

b2004 cbeA CP4-44 prophage; cytoskeleton bundling-enhancingantitoxin CbeA

//

>NGHB#1957 88

CMPLX not

b2004 cbeA CP4-44 prophage; cytoskeleton bundling-enhancingantitoxin CbeA

b2005 cbtA CP4-44 prophage; cytoskeleton-binding toxinCbtA

//

>NGHB#1958 -4

CMPLX ---

b2005 cbtA CP4-44 prophage; cytoskeleton-binding toxinCbtA

b2006 yeeW CP4-44 prophage; putative uncharacterizedprotein YeeW

//

>NGHB#1959 440

CMPLX ---

b2006 yeeW CP4-44 prophage; putative uncharacterizedprotein YeeW

b4538 yoeF CP4-44 prophage; putative uncharacterizedprotein YoeF

//

>NGHB#1961 171

CMPLX not

b2008 yeeA putative transporter YeeA

b2007 yeeX DUF496 domain-containing protein YeeX

//

>NGHB#1962 197

CMPLX not

b2009 sbmC DNA gyrase inhibitor

b2008 yeeA putative transporter YeeA

//

>NGHB#1963 118

CMPLX not

b2010 dacD D-alanyl-D-alanine carboxypeptidase DacD

b2009 sbmC DNA gyrase inhibitor

//

>NGHB#1966 13

CMPLX not

b2013 yeeE inner membrane protein YeeE

b2012 yeeD putative sulfurtransferase YeeD

//

>NGHB#1967 178

CMPLX not

b2014 plaP putrescine:H(+) symporter PlaP

b2013 yeeE inner membrane protein YeeE

//

>NGHB#1968 -11

CMPLX ---

b4678 yoeI uncharacterized protein YoeI

b2014 plaP putrescine:H(+) symporter PlaP

//

>NGHB#1969 214

CMPLX ---

b2015 yeeY putative DNA-binding transcriptional regulatorYeeY

b4678 yoeI uncharacterized protein YoeI

//

>NGHB#1970 45

CMPLX not

b2016 yeeZ putative epimerase YeeZ

b2015 yeeY putative DNA-binding transcriptional regulatorYeeY

//

>NGHB#1971 82

CMPLX not

b4539 yoeB ribosome-dependent mRNA interferase toxin YoeB

b2016 yeeZ putative epimerase YeeZ

//

>NGHB#1972 -4

CMPLX not

b2017 yefM YefM antitoxin of the YoeB-YefM toxin-antitoxinpair and DNA binding transcriptional repressor

b4539 yoeB ribosome-dependent mRNA interferase toxin YoeB

//

>NGHB#1974 145

CMPLX not

b2018 hisL his operon leader peptide

b2019 hisG ATP phosphoribosyltransferase

//

>NGHB#1975 5

CMPLX not

b2019 hisG ATP phosphoribosyltransferase

b2020 hisD histidinal/histidinol dehydrogenase

//

>NGHB#1976 -4

CMPLX not

b2020 hisD histidinal/histidinol dehydrogenase

b2021 hisC histidinol-phosphate aminotransferase

//

>NGHB#1977 -1

CMPLX not

b2021 hisC histidinol-phosphate aminotransferase

b2022 hisB imidazoleglycerol-phosphatedehydratase/histidinol-phosphatase

//

>NGHB#1978 -1

CMPLX not

b2022 hisB imidazoleglycerol-phosphatedehydratase/histidinol-phosphatase

b2023 hisH imidazole glycerol phosphate synthase subunitHisH

//

>NGHB#1979 -1

CMPLX not

b2023 hisH imidazole glycerol phosphate synthase subunitHisH

b2024 hisA 1-(5-phosphoribosyl)-5-[(5- phosphoribosylamino)methylideneamino]imidazole-4-carboxamide isomerase

//

>NGHB#1980 -19

CMPLX not

b2024 hisA 1-(5-phosphoribosyl)-5-[(5- phosphoribosylamino)methylideneamino]imidazole-4-carboxamide isomerase

b2025 hisF imidazole glycerol phosphate synthase subunitHisF

//

>NGHB#1981 -7

CMPLX not

b2025 hisF imidazole glycerol phosphate synthase subunitHisF

b2026 hisI putative bifunctional phosphoribosyl-AMPcyclohydrolase/phosphoribosyl-ATP pyrophosphatase

//

>NGHB#1983 145

CMPLX not

b2028 ugd UDP-glucose 6-dehydrogenase

b2027 wzzB regulator of length of O-antigen component oflipopolysaccharide chains

//

>NGHB#1984 248

CMPLX not

b2029 gnd 6-phosphogluconate dehydrogenase,decarboxylating

b2028 ugd UDP-glucose 6-dehydrogenase

//

>NGHB#1985 127

CMPLX not

b4571 wbbL interrupted rhamnosyltransferase WbbL

b2029 gnd 6-phosphogluconate dehydrogenase,decarboxylating

//

>NGHB#1986 -16

CMPLX not

b2033 wbbJ putative lipopolysaccharide biosynthesisO-acetyl transferase WbbJ

b2032 wbbK putative glycosyltransferase WbbK

//

>NGHB#1987 -20

CMPLX not

b2034 wbbI beta-1,6-galactofuranosyltransferase WbbI

b2033 wbbJ putative lipopolysaccharide biosynthesisO-acetyl transferase WbbJ

//

>NGHB#1988 2

CMPLX not

b2035 wbbH putative O-antigen polymerase

b2034 wbbI beta-1,6-galactofuranosyltransferase WbbI

//

>NGHB#1989 -1

CMPLX not

b2036 glf UDP-galactopyranose mutase

b2035 wbbH putative O-antigen polymerase

//

>NGHB#1990 7

CMPLX not

b2037 rfbX polyisoprenol-linked O-antigen repeat unitflippase

b2036 glf UDP-galactopyranose mutase

//

>NGHB#1991 -4

CMPLX not

b2038 rfbC dTDP-4-dehydrorhamnose 3,5-epimerase

b2037 rfbX polyisoprenol-linked O-antigen repeat unitflippase

//

>NGHB#1992 -1

CMPLX not

b2039 rfbA dTDP-glucose pyrophosphorylase

b2038 rfbC dTDP-4-dehydrorhamnose 3,5-epimerase

//

>NGHB#1993 57

CMPLX not

b2040 rfbD dTDP-4-dehydrorhamnose reductase

b2039 rfbA dTDP-glucose pyrophosphorylase

//

>NGHB#1994 -1

CMPLX not

b2041 rfbB dTDP-glucose 4,6-dehydratase 1

b2040 rfbD dTDP-4-dehydrorhamnose reductase

//

>NGHB#1995 372

CMPLX not

b2042 galF UTP:glucose-1-phosphate uridylyltransferase, lowactivity

b2041 rfbB dTDP-glucose 4,6-dehydratase 1

//

>NGHB#1996 174

CMPLX not

b2043 wcaM putative colanic acid biosynthesis protein WcaM

b2042 galF UTP:glucose-1-phosphate uridylyltransferase, lowactivity

//

>NGHB#1997 10

CMPLX not

b2044 wcaL putative colanic biosynthesis glycosyltransferase

b2043 wcaM putative colanic acid biosynthesis protein WcaM

//

>NGHB#1998 -4

CMPLX not

b2045 wcaK putative colanic acid biosynthesis pyruvyltransferase WcaK

b2044 wcaL putative colanic biosynthesis glycosyltransferase

//

>NGHB#1999 275

CMPLX not

b2046 wzxC M-antigen undecaprenyl disphosphate flippase

b2045 wcaK putative colanic acid biosynthesis pyruvyltransferase WcaK

//

>NGHB#2000 1

CMPLX not

b2047 wcaJ UDP-glucose:undecaprenyl-phosphateglucose-1-phosphate transferase

b2046 wzxC M-antigen undecaprenyl disphosphate flippase

//

>NGHB#2001 54

CMPLX not

b2048 cpsG phosphomannomutase

b2047 wcaJ UDP-glucose:undecaprenyl-phosphateglucose-1-phosphate transferase

//

>NGHB#2002 104

CMPLX not

b2049 cpsB mannose-1-phosphate guanylyltransferase

b2048 cpsG phosphomannomutase

//

>NGHB#2003 2

CMPLX not

b2050 wcaI colanic acid biosynthesis fucosyltransferaseWcaI

b2049 cpsB mannose-1-phosphate guanylyltransferase

//

>NGHB#2004 -4

CMPLX not

b2051 gmm GDP-mannose mannosyl hydrolase

b2050 wcaI colanic acid biosynthesis fucosyltransferaseWcaI

//

>NGHB#2005 2

CMPLX not

b2052 fcl GDP-L-fucose synthase

b2051 gmm GDP-mannose mannosyl hydrolase

//

>NGHB#2006 2

CMPLX not

b2053 gmd GDP-mannose 4,6-dehydratase

b2052 fcl GDP-L-fucose synthase

//

>NGHB#2007 25

CMPLX not

b2054 wcaF colanic acid biosynthesis acetyltransferaseWcaF

b2053 gmd GDP-mannose 4,6-dehydratase

//

>NGHB#2008 15

CMPLX not

b2055 wcaE colanic acid biosynthesis fucosyltransferaseWcaE

b2054 wcaF colanic acid biosynthesis acetyltransferaseWcaF

//

>NGHB#2009 10

CMPLX not

b2056 wcaD putative colanic acid polymerase

b2055 wcaE colanic acid biosynthesis fucosyltransferaseWcaE

//

>NGHB#2010 -26

CMPLX not

b2057 wcaC colanic acid biosynthesis galactosyltransferaseWcaC

b2056 wcaD putative colanic acid polymerase

//

>NGHB#2011 -4

CMPLX not

b2058 wcaB colanic acid biosynthesis acetyltransferaseWcaB

b2057 wcaC colanic acid biosynthesis galactosyltransferaseWcaC

//

>NGHB#2012 2

CMPLX not

b2059 wcaA colanic acid biosynthesisglucuronosyltransferase WcaA

b2058 wcaB colanic acid biosynthesis acetyltransferaseWcaB

//

>NGHB#2013 92

CMPLX not

b2060 wzc protein-tyrosine kinase Wzc

b2059 wcaA colanic acid biosynthesisglucuronosyltransferase WcaA

//

>NGHB#2014 2

CMPLX not

b2061 wzb protein-tyrosine phosphatase

b2060 wzc protein-tyrosine kinase Wzc

//

>NGHB#2015 5

CMPLX not

b2062 wza outer membrane polysaccharide export proteinWza

b2061 wzb protein-tyrosine phosphatase

//

>NGHB#2018 21

CMPLX not

b2065 dcd dCTP deaminase

b2064 asmA putative assembly protein AsmA

//

>NGHB#2019 91

CMPLX not

b2066 udk uridine/cytidine kinase

b2065 dcd dCTP deaminase

//

>NGHB#2026 -4

CMPLX not

b2073 yegL IPR002035 domain-containing protein YegL

b2072 pphC protein-serine/threonine phosphatase PphC

//

>NGHB#2027 220

CMPLX not

b4667 ibsA toxic peptide IbsA

b2073 yegL IPR002035 domain-containing protein YegL

//

>NGHB#2028 272

CMPLX yes

b4668 ibsB putative toxic peptide IbsB

b4667 ibsA toxic peptide IbsA

//

>NGHB#2030 -1

CMPLX yes

b2074 mdtA multidrug efflux pump membrane fusion proteinMdtA

b2075 mdtB multidrug efflux pump RND permease subunit MdtB

//

>NGHB#2031 0

CMPLX yes

b2075 mdtB multidrug efflux pump RND permease subunit MdtB

b2076 mdtC multidrug efflux pump RND permease subunit MdtC

//

>NGHB#2032 0

CMPLX yes

b2076 mdtC multidrug efflux pump RND permease subunit MdtC

b2077 mdtD putative multidrug efflux pump MdtD

//

>NGHB#2033 -4

CMPLX not

b2077 mdtD putative multidrug efflux pump MdtD

b2078 baeS sensor histidine kinase BaeS

//

>NGHB#2034 -4

CMPLX not

b2078 baeS sensor histidine kinase BaeS

b2079 baeR DNA-binding transcriptional activator BaeR

//

>NGHB#2035 190

CMPLX not

b2079 baeR DNA-binding transcriptional activator BaeR

b2080 yegP DUF1508 domain-containing protein YegP

//

>NGHB#2036 146

CMPLX not

b2080 yegP DUF1508 domain-containing protein YegP

b2081 trhP tRNA wobble base hydroxylation protein TrhP

//

>NGHB#2038 468

CMPLX ---

b2085 yegR uncharacterized protein YegR

b2082 ogrK prophage P2 late control protein OgrK

//

>NGHB#2041 -4

CMPLX tnp

b2088 insE5 IS3 element protein InsE

b2089 insF5 IS3 element protein InsF

//

>NGHB#2042 1408

CMPLX not

b2093 gatB galactitol-specific PTS enzyme IIB component

b2091 gatD galactitol-1-phosphate 5-dehydrogenase

//

>NGHB#2043 30

CMPLX yes

b2094 gatA galactitol-specific PTS enzyme IIA component

b2093 gatB galactitol-specific PTS enzyme IIB component

//

>NGHB#2044 9

CMPLX not

b2095 gatZ tagatose-1,6-bisphosphate aldolase 2 subunitGatZ

b2094 gatA galactitol-specific PTS enzyme IIA component

//

>NGHB#2045 28

CMPLX yes

b2096 gatY tagatose-1,6-bisphosphate aldolase 2 subunitGatY

b2095 gatZ tagatose-1,6-bisphosphate aldolase 2 subunitGatZ

//

>NGHB#2046 307

CMPLX not

b2097 fbaB fructose-bisphosphate aldolase class I

b2096 gatY tagatose-1,6-bisphosphate aldolase 2 subunitGatY

//

>NGHB#2048 -4

CMPLX not

b2098 yegT putative transporter YegT

b2099 yegU putative ADP-ribosylglycohydrolase YegU

//

>NGHB#2049 -4

CMPLX not

b2099 yegU putative ADP-ribosylglycohydrolase YegU

b2100 yegV putative sugar kinase YegV

//

>NGHB#2051 51

CMPLX not

b2102 yegX putative glycosyl hydrolase YegX

b2101 yegW putative DNA-binding transcriptional regulatorYegW

//

>NGHB#2052 64

CMPLX not

b2103 thiD bifunctional hydroxymethylpyrimidinekinase/phosphomethylpyrimidine kinase

b2102 yegX putative glycosyl hydrolase YegX

//

>NGHB#2053 -4

CMPLX not

b2104 thiM hydroxyethylthiazole kinase

b2103 thiD bifunctional hydroxymethylpyrimidinekinase/phosphomethylpyrimidine kinase

//

>NGHB#2054 222

CMPLX not

b2105 rcnR DNA-binding transcriptional repressor RcnR

b2104 thiM hydroxyethylthiazole kinase

//

>NGHB#2056 218

CMPLX not

b2106 rcnA Ni(2(+))/Co(2(+)) exporter

b2107 rcnB periplasmic protein involved in nickel/cobaltexport

//

>NGHB#2058 15

CMPLX not

b2109 yehB putative fimbrial usher protein YehB

b2108 yehA putative fimbrial adhesin YehA

//

>NGHB#2059 15

CMPLX not

b2110 yehC putative fimbrial chaperone YehC

b2109 yehB putative fimbrial usher protein YehB

//

>NGHB#2060 34

CMPLX not

b2111 yehD putative fimbrial protein YehD

b2110 yehC putative fimbrial chaperone YehC

//

>NGHB#2061 292

CMPLX not

b2112 yehE DUF2574 domain-containing protein YehE

b2111 yehD putative fimbrial protein YehD

//

>NGHB#2062 262

CMPLX not

b2113 mrp P-loop NTPase family protein Mrp

b2112 yehE DUF2574 domain-containing protein YehE

//

>NGHB#2064 140

CMPLX not

b2114 metG methionine--tRNA ligase

b2115 yehF protein YehF

//

>NGHB#2065 2980

CMPLX not

b2115 yehF protein YehF

b2118 yehI DUF4132 domain-containing protein YehI

//

>NGHB#2066 60

CMPLX ---

b2118 yehI DUF4132 domain-containing protein YehI

b4541 yehK uncharacterized protein YehK

//

>NGHB#2067 306

CMPLX ---

b4541 yehK uncharacterized protein YehK

b2119 yehL putative AAA(+) MoxR family ATPase YehL

//

>NGHB#2068 10

CMPLX ---

b2119 yehL putative AAA(+) MoxR family ATPase YehL

b2120 yehM uncharacterized protein YehM

//

>NGHB#2069 -8

CMPLX ---

b2120 yehM uncharacterized protein YehM

b2121 yehP VWA domain-containing protein YehP

//

>NGHB#2070 -4

CMPLX not

b2121 yehP VWA domain-containing protein YehP

b2122 yehQ SWIM zinc finger domains-containing proteinYehQ

//

>NGHB#2071 280

CMPLX not

b2122 yehQ SWIM zinc finger domains-containing proteinYehQ

b2123 yehR DUF1307 domain-containing lipoprotein YehR

//

>NGHB#2073 46

CMPLX not

b2125 btsR DNA-binding transcriptional dual regulator BtsR

b2124 yehS DUF1456 domain-containing protein YehS

//

>NGHB#2074 -4

CMPLX not

b2126 btsS high-affinity pyruvate receptor

b2125 btsR DNA-binding transcriptional dual regulator BtsR

//

>NGHB#2076 59

CMPLX not

b2127 mlrA DNA-binding transcriptional activator MlrA

b4542 yohO UPF0387 family protein YohO

//

>NGHB#2078 4

CMPLX yes

b2129 yehX glycine betaine ABC transporter ATP bindingsubunit YehX

b2128 yehW glycine betaine ABC transporter membrane subunitYehW

//

>NGHB#2079 -8

CMPLX yes

b2130 yehY glycine betaine ABC transporter membrane subunitYehY

b2129 yehX glycine betaine ABC transporter ATP bindingsubunit YehX

//

>NGHB#2080 6

CMPLX yes

b2131 osmF glycine betaine ABC transporter periplasmicbinding protein OsmF

b2130 yehY glycine betaine ABC transporter membrane subunitYehY

//

>NGHB#2081 210

CMPLX not

b2132 bglX beta-D-glucoside glucohydrolase, periplasmic

b2131 osmF glycine betaine ABC transporter periplasmicbinding protein OsmF

//

>NGHB#2084 173

CMPLX not

b2135 yohC putative inner membrane protein

b2134 pbpG peptidoglycan DD-endopeptidase PbpG

//

>NGHB#2090 -4

CMPLX not

b2141 yohJ PF03788 family membrane protein YohJ

b2142 yohK PF04712 family membrane protein YohK

//

>NGHB#2091 129

CMPLX not

b2142 yohK PF04712 family membrane protein YohK

b2143 cdd cytidine/deoxycytidine deaminase

//

>NGHB#2092 149

CMPLX not

b2143 cdd cytidine/deoxycytidine deaminase

b2144 sanA DUF218 domain-containing protein SanA

//

>NGHB#2093 2

CMPLX not

b2144 sanA DUF218 domain-containing protein SanA

b2145 yeiS DUF2542 domain-containing protein YeiS

//

>NGHB#2094 193

CMPLX not

b2145 yeiS DUF2542 domain-containing protein YeiS

b2146 preT NAD-dependent dihydropyrimidine dehydrogenasesubunit PreT

//

>NGHB#2095 -7

CMPLX yes

b2146 preT NAD-dependent dihydropyrimidine dehydrogenasesubunit PreT

b2147 preA NAD-dependent dihydropyrimidine dehydrogenasesubunit PreA

//

>NGHB#2097 15

CMPLX yes

b2149 mglA D-galactose/methyl-galactoside ABC transporterATP binding subunit

b2148 mglC D-galactose/methyl-galactoside ABC transportermembrane subunit

//

>NGHB#2098 60

CMPLX yes

b2150 mglB D-galactose/methyl-galactoside ABC transporterperiplasmic binding protein

b2149 mglA D-galactose/methyl-galactoside ABC transporterATP binding subunit

//

>NGHB#2099 279

CMPLX not

b2151 galS DNA-binding transcriptional dual regulator GalS

b2150 mglB D-galactose/methyl-galactoside ABC transporterperiplasmic binding protein

//

>NGHB#2100 141

CMPLX not

b2152 yeiB DUF418 domain-containing protein YeiB

b2151 galS DNA-binding transcriptional dual regulator GalS

//

>NGHB#2101 16

CMPLX not

b2153 folE GTP cyclohydrolase 1

b2152 yeiB DUF418 domain-containing protein YeiB

//

>NGHB#2104 293

CMPLX not

b2156 lysP lysine:H(+) symporter

b2155 cirA iron-catecholate outer membrane transporterCirA

//

>NGHB#2105 204

CMPLX not

b2157 yeiE LysR-type transcriptional regulator YeiE

b2156 lysP lysine:H(+) symporter

//

>NGHB#2107 73

CMPLX not

b2158 yeiH PF03601 family inner membrane protein YeiH

b2159 nfo endonuclease IV

//

>NGHB#2108 2

CMPLX not

b2159 nfo endonuclease IV

b2160 yeiI putative sugar kinase YeiI

//

>NGHB#2110 99

CMPLX not

b2162 rihB pyrimidine-specific ribonucleoside hydrolaseRihB

b2161 nupX putative nucleoside transporter

//

>NGHB#2113 93

CMPLX not

b2165 psuG pseudouridine-5'-phosphate glycosidase

b2164 psuT putative pseudouridine transporter

//

>NGHB#2114 -13

CMPLX not

b2166 psuK putative pseudouridine kinase

b2165 psuG pseudouridine-5'-phosphate glycosidase

//

>NGHB#2115 422

CMPLX not

b2167 fruA fructose-specific PTS multiphosphoryl transferprotein FruA

b2166 psuK putative pseudouridine kinase

//

>NGHB#2116 16

CMPLX not

b2168 fruK 1-phosphofructokinase

b2167 fruA fructose-specific PTS multiphosphoryl transferprotein FruA

//

>NGHB#2117 -1

CMPLX not

b2169 fruB fructose-specific PTS multiphosphoryl transferprotein FruB

b2168 fruK 1-phosphofructokinase

//

>NGHB#2121 222

CMPLX not

b2171 yeiP elongation factor P-like protein YeiP

b2172 yeiQ putative oxidoreductase YeiQ

//

>NGHB#2122 117

CMPLX not

b2172 yeiQ putative oxidoreductase YeiQ

b2173 yeiR zinc-binding GTPase YeiR

//

>NGHB#2123 38

CMPLX not

b2173 yeiR zinc-binding GTPase YeiR

b2174 lpxT Kdo2-lipid A phosphotransferase

//

>NGHB#2124 411

CMPLX not

b2174 lpxT Kdo2-lipid A phosphotransferase

b2175 mepS peptidoglycan DD-endopeptidase/peptidoglycanLD-carboxypeptidase

//

>NGHB#2125 180

CMPLX not

b2175 mepS peptidoglycan DD-endopeptidase/peptidoglycanLD-carboxypeptidase

b2176 pdeN putative c-di-GMP phosphodiesterase PdeN

//

>NGHB#2126 81

CMPLX not

b2176 pdeN putative c-di-GMP phosphodiesterase PdeN

b2177 yejA putative oligopeptide ABC transporterperiplasmic binding protein YejA

//

>NGHB#2127 0

CMPLX yes

b2177 yejA putative oligopeptide ABC transporterperiplasmic binding protein YejA

b2178 yejB putative oligopeptide ABC transporter membranesubunit YejB

//

>NGHB#2128 -1

CMPLX yes

b2178 yejB putative oligopeptide ABC transporter membranesubunit YejB

b2179 yejE putative oligopeptide ABC transporter membranesubunit YejE

//

>NGHB#2129 1

CMPLX yes

b2179 yejE putative oligopeptide ABC transporter membranesubunit YejE

b2180 yejF putative oligopeptide ABC transporter ATPbinding subunit YejF

//

>NGHB#2131 332

CMPLX not

b2182 bcr multidrug efflux pump Bcr

b2181 yejG protein YejG

//

>NGHB#2132 27

CMPLX not

b2183 rsuA 16S rRNA pseudouridine(516) synthase

b2182 bcr multidrug efflux pump Bcr

//

>NGHB#2134 124

CMPLX not

b2184 radD putative DNA repair helicase RadD

b2185 rplY 50S ribosomal subunit protein L25

//

>NGHB#2137 19

CMPLX not

b2187 yejL DUF1414 domain-containing protein YejL

b2188 yejM LPS homeostasis protein YejM

//

>NGHB#2140 -4

CMPLX not

b2195 ccmG thiol:disulfide oxidoreductase CcmG

b2194 ccmH holocytochrome c synthase CcmH component

//

>NGHB#2141 -4

CMPLX not

b2196 ccmF holocytochrome c synthase CcmF component

b2195 ccmG thiol:disulfide oxidoreductase CcmG

//

>NGHB#2142 -4

CMPLX not

b2197 ccmE periplasmic heme chaperone

b2196 ccmF holocytochrome c synthase CcmF component

//

>NGHB#2143 -4

CMPLX not

b2198 ccmD cytochrome c maturation protein D

b2197 ccmE periplasmic heme chaperone

//

>NGHB#2144 -4

CMPLX yes

b2199 ccmC cytochrome c maturation protein C

b2198 ccmD cytochrome c maturation protein D

//

>NGHB#2145 41

CMPLX yes

b2200 ccmB cytochrome c maturation protein B

b2199 ccmC cytochrome c maturation protein C

//

>NGHB#2146 -4

CMPLX yes

b2201 ccmA cytochrome c maturation protein A

b2200 ccmB cytochrome c maturation protein B

//

>NGHB#2147 12

CMPLX not

b2202 napC periplasmic nitrate reductase cytochrome cprotein

b2201 ccmA cytochrome c maturation protein A

//

>NGHB#2148 9

CMPLX yes

b2203 napB periplasmic nitrate reductase cytochrome c550protein

b2202 napC periplasmic nitrate reductase cytochrome cprotein

//

>NGHB#2149 -4

CMPLX not

b2204 napH ferredoxin-type protein NapH

b2203 napB periplasmic nitrate reductase cytochrome c550protein

//

>NGHB#2150 -14

CMPLX yes

b2205 napG ferredoxin-type protein NapG

b2204 napH ferredoxin-type protein NapH

//

>NGHB#2151 6

CMPLX not

b2206 napA periplasmic nitrate reductase subunit NapA

b2205 napG ferredoxin-type protein NapG

//

>NGHB#2152 -4

CMPLX not

b2207 napD NapA signal peptide-binding chaperone NapD

b2206 napA periplasmic nitrate reductase subunit NapA

//

>NGHB#2153 -11

CMPLX not

b2208 napF ferredoxin-type protein

b2207 napD NapA signal peptide-binding chaperone NapD

//

>NGHB#2155 134

CMPLX ---

b4604 yojO uncharacterized protein YojO

b2209 eco serine protease inhibitor ecotin

//

>NGHB#2157 217

CMPLX not

b2211 yojI ABC transporter family protein/microcin J25efflux protein

b2210 mqo malate:quinone oxidoreductase

//

>NGHB#2158 75

CMPLX not

b2212 alkB DNA oxidative demethylase

b2211 yojI ABC transporter family protein/microcin J25efflux protein

//

>NGHB#2159 -1

CMPLX not

b2213 ada DNA-binding transcriptional dual regulator/DNArepair protein Ada

b2212 alkB DNA oxidative demethylase

//

>NGHB#2160 73

CMPLX not

b2214 ftp FAD:protein FMN transferase

b2213 ada DNA-binding transcriptional dual regulator/DNArepair protein Ada

//

>NGHB#2161 111

CMPLX not

b2215 ompC outer membrane porin C

b2214 ftp FAD:protein FMN transferase

//

>NGHB#2163 16

CMPLX not

b2216 rcsD RcsD phosphotransferase

b2217 rcsB DNA-binding transcriptional activator RcsB

//

>NGHB#2166 -4

CMPLX not

b2219 atoS sensor histidine kinase AtoS

b2220 atoC DNA-binding transcriptional activator/ornithinedecarboxylase inhibitor AtoC

//

>NGHB#2167 195

CMPLX not

b2220 atoC DNA-binding transcriptional activator/ornithinedecarboxylase inhibitor AtoC

b2221 atoD acetyl-CoA:acetoacetyl-CoA transferase subunitalpha

//

>NGHB#2168 -1

CMPLX yes

b2221 atoD acetyl-CoA:acetoacetyl-CoA transferase subunitalpha

b2222 atoA acetyl-CoA:acetoacetyl-CoA transferase subunitbeta

//

>NGHB#2169 -4

CMPLX not

b2222 atoA acetyl-CoA:acetoacetyl-CoA transferase subunitbeta

b2223 atoE short chain fatty acid transporter

//

>NGHB#2170 30

CMPLX not

b2223 atoE short chain fatty acid transporter

b2224 atoB acetyl-CoA acetyltransferase

//

>NGHB#2172 4

CMPLX not

b2226 yfaQ tandem DUF2300 domain-containing protein YfaQ

b2225 yfaP DUF2135 domain-containing protein YfaP

//

>NGHB#2173 4538

CMPLX not

b2229 yfaT DUF1175 domain-containing protein YfaT

b2226 yfaQ tandem DUF2300 domain-containing protein YfaQ

//

>NGHB#2174 -4

CMPLX not

b2230 yfaA DUF2138 domain-containing protein YfaA

b2229 yfaT DUF1175 domain-containing protein YfaT

//

>NGHB#2175 148

CMPLX not

b2231 gyrA DNA gyrase subunit A

b2230 yfaA DUF2138 domain-containing protein YfaA

//

>NGHB#2178 424

CMPLX not

b4605 ypaB protein YpaB

b2233 yfaL putative autotransporter adhesin YfaL

//

>NGHB#2182 -1

CMPLX not

b2235 nrdB ribonucleoside-diphosphate reductase 1 subunitbeta

b2236 yfaE ferredoxin-like diferric-tyrosyl radicalcofactor maintenance protein YfaE

//

>NGHB#2184 462

CMPLX not

b2239 glpQ glycerophosphoryl diester phosphodiesteraseGlpQ

b2237 inaA putative lipopolysaccharide kinase InaA

//

>NGHB#2185 4

CMPLX not

b2240 glpT sn-glycerol 3-phosphate:phosphate antiporter

b2239 glpQ glycerophosphoryl diester phosphodiesteraseGlpQ

//

>NGHB#2187 -11

CMPLX yes

b2241 glpA anaerobic glycerol-3-phosphate dehydrogenasesubunit A

b2242 glpB anaerobic glycerol-3-phosphate dehydrogenasesubunit B

//

>NGHB#2188 -4

CMPLX yes

b2242 glpB anaerobic glycerol-3-phosphate dehydrogenasesubunit B

b2243 glpC anaerobic glycerol-3-phosphate dehydrogenasesubunit C

//

>NGHB#2189 192

CMPLX not

b2243 glpC anaerobic glycerol-3-phosphate dehydrogenasesubunit C

b2244 rpnE inactive recombination-promoting nuclease-likeprotein RpnE

//

>NGHB#2191 17

CMPLX not

b2246 yfaV putative transporter YfaV

b2245 yfaU 2-keto-3-deoxy-L-rhamnonate aldolase

//

>NGHB#2192 56

CMPLX not

b2247 rhmD L-rhamnonate dehydratase

b2246 yfaV putative transporter YfaV

//

>NGHB#2193 14

CMPLX not

b2248 yfaX putative DNA-binding transcriptional regulatorYfaX

b2247 rhmD L-rhamnonate dehydratase

//

>NGHB#2194 219

CMPLX not

b2249 yfaY IPR008135 CinA family protein YfaY

b2248 yfaX putative DNA-binding transcriptional regulatorYfaX

//

>NGHB#2195 99

CMPLX not

b2250 yfaZ putative porin YfaZ

b2249 yfaY IPR008135 CinA family protein YfaY

//

>NGHB#2199 3

CMPLX not

b2253 arnB UDP-4-amino-4-deoxy-L-arabinoseaminotransferase

b2254 arnC undecaprenyl-phosphate4-deoxy-4-formamido-L-arabinose transferase

//

>NGHB#2200 -1

CMPLX not

b2254 arnC undecaprenyl-phosphate4-deoxy-4-formamido-L-arabinose transferase

b2255 arnA fused UDP-4-amino-4-deoxy-L-arabinoseformyltransferase/UDP-glucuronate dehydrogenase

//

>NGHB#2201 -4

CMPLX not

b2255 arnA fused UDP-4-amino-4-deoxy-L-arabinoseformyltransferase/UDP-glucuronate dehydrogenase

b2256 arnD putative 4-deoxy-4-formamido-L-arabinose-phosphoundecaprenoldeformylase ArnD

//

>NGHB#2202 -1

CMPLX not

b2256 arnD putative 4-deoxy-4-formamido-L-arabinose-phosphoundecaprenoldeformylase ArnD

b2257 arnT lipid IVA4-amino-4-deoxy-L-arabinosyltransferase

//

>NGHB#2203 -4

CMPLX not

b2257 arnT lipid IVA4-amino-4-deoxy-L-arabinosyltransferase

b4544 arnE undecaprenyl-phosphate-alpha-L-Ara4N flippase -ArnE subunit

//

>NGHB#2204 -1

CMPLX yes

b4544 arnE undecaprenyl-phosphate-alpha-L-Ara4N flippase -ArnE subunit

b2258 arnF undecaprenyl-phosphate-alpha-L-Ara4N flippase -ArnF subunit

//

>NGHB#2206 109

CMPLX not

b2260 menE o-succinylbenzoate--CoA ligase

b2259 pmrD signal transduction protein PmrD

//

>NGHB#2207 -4

CMPLX not

b2261 menC o-succinylbenzoate synthase

b2260 menE o-succinylbenzoate--CoA ligase

//

>NGHB#2208 -1

CMPLX not

b2262 menB 1,4-dihydroxy-2-naphthoyl-CoA synthase

b2261 menC o-succinylbenzoate synthase

//

>NGHB#2209 14

CMPLX not

b2263 menH 2-succinyl-6-hydroxy-2,4-cyclohexadiene-1-carboxylate synthase

b2262 menB 1,4-dihydroxy-2-naphthoyl-CoA synthase

//

>NGHB#2210 -4

CMPLX not

b2264 menD 2-succinyl-5-enolpyruvyl-6-hydroxy-3-cyclohexene-1-carboxylate synthase

b2263 menH 2-succinyl-6-hydroxy-2,4-cyclohexadiene-1-carboxylate synthase

//

>NGHB#2211 88

CMPLX not

b2265 menF isochorismate synthase MenF

b2264 menD 2-succinyl-5-enolpyruvyl-6-hydroxy-3-cyclohexene-1-carboxylate synthase

//

>NGHB#2212 78

CMPLX not

b2266 elaB tail-anchored inner membrane protein ElaB

b2265 menF isochorismate synthase MenF

//

>NGHB#2213 54

CMPLX not

b2267 elaA putative N-acetyltransferase ElaA

b2266 elaB tail-anchored inner membrane protein ElaB

//

>NGHB#2215 187

CMPLX not

b2268 rbn ribonuclease BN

b2269 elaD protease ElaD

//

>NGHB#2218 102

CMPLX not

b2271 yfbL putative peptidase YfbL

b2272 yfbM DUF1877 domain-containing protein YfbM

//

>NGHB#2221 58

CMPLX ---

b2274 yfbO uncharacterized protein YfbO

b2275 yfbP uncharacterized protein YfbP

//

>NGHB#2223 6

CMPLX yes

b2277 nuoM NADH:quinone oxidoreductase subunit M

b2276 nuoN NADH:quinone oxidoreductase subunit N

//

>NGHB#2224 163

CMPLX yes

b2278 nuoL NADH:quinone oxidoreductase subunit L

b2277 nuoM NADH:quinone oxidoreductase subunit M

//

>NGHB#2225 -4

CMPLX yes

b2279 nuoK NADH:quinone oxidoreductase subunit K

b2278 nuoL NADH:quinone oxidoreductase subunit L

//

>NGHB#2226 -4

CMPLX yes

b2280 nuoJ NADH:quinone oxidoreductase subunit J

b2279 nuoK NADH:quinone oxidoreductase subunit K

//

>NGHB#2227 11

CMPLX yes

b2281 nuoI NADH:quinone oxidoreductase subunit I

b2280 nuoJ NADH:quinone oxidoreductase subunit J

//

>NGHB#2228 14

CMPLX yes

b2282 nuoH NADH:quinone oxidoreductase subunit H

b2281 nuoI NADH:quinone oxidoreductase subunit I

//

>NGHB#2229 -4

CMPLX yes

b2283 nuoG NADH:quinone oxidoreductase subunit G

b2282 nuoH NADH:quinone oxidoreductase subunit H

//

>NGHB#2230 52

CMPLX yes

b2284 nuoF NADH:quinone oxidoreductase subunit F

b2283 nuoG NADH:quinone oxidoreductase subunit G

//

>NGHB#2231 -4

CMPLX yes

b2285 nuoE NADH:quinone oxidoreductase subunit E

b2284 nuoF NADH:quinone oxidoreductase subunit F

//

>NGHB#2232 2

CMPLX yes

b2286 nuoC NADH:quinone oxidoreductase subunit CD

b2285 nuoE NADH:quinone oxidoreductase subunit E

//

>NGHB#2233 105

CMPLX yes

b2287 nuoB NADH:quinone oxidoreductase subunit B

b2286 nuoC NADH:quinone oxidoreductase subunit CD

//

>NGHB#2234 15

CMPLX yes

b2288 nuoA NADH:quinone oxidoreductase subunit A

b2287 nuoB NADH:quinone oxidoreductase subunit B

//

>NGHB#2235 630

CMPLX not

b2289 lrhA DNA-binding transcriptional dual regulator LrhA

b2288 nuoA NADH:quinone oxidoreductase subunit A

//

>NGHB#2237 83

CMPLX not

b2290 alaA glutamate--pyruvate aminotransferase AlaA

b2291 yfbR dCMP phosphohydrolase

//

>NGHB#2239 86

CMPLX not

b2293 hxpA hexitol phosphatase A

b2292 yfbS putative transporter YfbS

//

>NGHB#2240 10

CMPLX not

b2294 yfbU UPF0304 family protein YfbU

b2293 hxpA hexitol phosphatase A

//

>NGHB#2241 82

CMPLX not

b2295 yfbV UPF0208 membrane protein YfbV

b2294 yfbU UPF0304 family protein YfbU

//

>NGHB#2243 74

CMPLX not

b2296 ackA acetate kinase

b2297 pta phosphate acetyltransferase

//

>NGHB#2244 189

CMPLX not

b2297 pta phosphate acetyltransferase

b2298 yfcC putative transporter YfcC

//

>NGHB#2246 57

CMPLX not

b2300 yfcE phosphodiesterase YfcE

b2299 yfcD putative Nudix hydrolase YfcD

//

>NGHB#2247 52

CMPLX not

b2301 yfcF glutathione S-transferase YfcF

b2300 yfcE phosphodiesterase YfcE

//

>NGHB#2249 56

CMPLX not

b2302 yfcG disulfide reductase

b2303 folX dihydroneopterin triphosphate 2'-epimerase

//

>NGHB#2250 20

CMPLX not

b2303 folX dihydroneopterin triphosphate 2'-epimerase

b2304 yfcH putative NAD-dependent epimerase YfcH

//

>NGHB#2252 196

CMPLX not

b2306 hisP lysine/arginine/ornithine ABC transporter/histidine ABC transporter, ATP bindingsubunit

b2305 rpnB recombination-promoting nuclease RpnB

//

>NGHB#2253 7

CMPLX yes

b2307 hisM lysine/arginine/ornithine ABC transporter/histidine ABC transporter, membrane subunitHisM

b2306 hisP lysine/arginine/ornithine ABC transporter/histidine ABC transporter, ATP bindingsubunit

//

>NGHB#2254 -4

CMPLX yes

b2308 hisQ lysine/arginine/ornithine ABC transporter/histidine ABC transporter, membrane subunitHisQ

b2307 hisM lysine/arginine/ornithine ABC transporter/histidine ABC transporter, membrane subunitHisM

//

>NGHB#2255 89

CMPLX yes

b2309 hisJ histidine ABC transporter periplasmic bindingprotein

b2308 hisQ lysine/arginine/ornithine ABC transporter/histidine ABC transporter, membrane subunitHisQ

//

>NGHB#2256 220

CMPLX not

b2310 argT lysine/arginine/ornithine ABC transporterperiplasmic binding protein

b2309 hisJ histidine ABC transporter periplasmic bindingprotein

//

>NGHB#2257 265

CMPLX not

b2311 ubiX flavin prenyltransferase

b2310 argT lysine/arginine/ornithine ABC transporterperiplasmic binding protein

//

>NGHB#2258 94

CMPLX not

b2312 purF amidophosphoribosyltransferase

b2311 ubiX flavin prenyltransferase

//

>NGHB#2259 36

CMPLX not

b2313 cvpA colicin V production protein

b2312 purF amidophosphoribosyltransferase

//

>NGHB#2260 258

CMPLX not

b2314 dedD cell division protein DedD

b2313 cvpA colicin V production protein

//

>NGHB#2261 -11

CMPLX not

b2315 folC bifunctional folylpolyglutamatesynthetase/dihydrofolate synthetase

b2314 dedD cell division protein DedD

//

>NGHB#2262 69

CMPLX not

b2316 accD acetyl-CoA carboxyltransferase subunit beta

b2315 folC bifunctional folylpolyglutamatesynthetase/dihydrofolate synthetase

//

>NGHB#2263 155

CMPLX not

b2317 dedA DedA family protein DedA

b2316 accD acetyl-CoA carboxyltransferase subunit beta

//

>NGHB#2264 82

CMPLX not

b2318 truA tRNA pseudouridine(38-40) synthase

b2317 dedA DedA family protein DedA

//

>NGHB#2265 -1

CMPLX not

b2319 usg putative semialdehyde dehydrogenase Usg

b2318 truA tRNA pseudouridine(38-40) synthase

//

>NGHB#2266 65

CMPLX not

b2320 pdxB erythronate-4-phosphate dehydrogenase

b2319 usg putative semialdehyde dehydrogenase Usg

//

>NGHB#2269 264

CMPLX not

b2323 fabB 3-oxoacyl-[acyl carrier protein] synthase 1

b2322 yfcJ putative transporter YfcJ

//

>NGHB#2272 33

CMPLX not

b2326 epmC EF-P-Lys34 hydroxylase

b2325 yfcL PF08891 family protein YfcL

//

>NGHB#2273 -1

CMPLX not

b2327 yfcA putative transporter YfcA

b2326 epmC EF-P-Lys34 hydroxylase

//

>NGHB#2274 -1

CMPLX not

b2328 mepA peptidoglycan DD-endopeptidase/peptidoglycanLD-endopeptidase

b2327 yfcA putative transporter YfcA

//

>NGHB#2275 3

CMPLX not

b2329 aroC chorismate synthase

b2328 mepA peptidoglycan DD-endopeptidase/peptidoglycanLD-endopeptidase

//

>NGHB#2276 34

CMPLX not

b2330 prmB 50S ribosomal subunit protein L3 N(5)-glutaminemethyltransferase

b2329 aroC chorismate synthase

//

>NGHB#2279 1

CMPLX not

b2333 yfcP putative fimbrial protein YfcP

b2332 yfcO DUF2544 domain-containing protein YfcO

//

>NGHB#2280 -4

CMPLX yes

b2334 yfcQ putative fimbrial protein YfcQ

b2333 yfcP putative fimbrial protein YfcP

//

>NGHB#2281 -4

CMPLX yes

b2335 yfcR putative fimbrial protein YfcR

b2334 yfcQ putative fimbrial protein YfcQ

//

>NGHB#2282 -1

CMPLX not

b2336 yfcS putative fimbrial chaperone YfcS

b2335 yfcR putative fimbrial protein YfcR

//

>NGHB#2283 2746

CMPLX not

b2339 yfcV putative fimbrial protein YfcV

b2336 yfcS putative fimbrial chaperone YfcS

//

>NGHB#2284 680

CMPLX not

b2340 sixA phosphohistidine phosphatase SixA

b2339 yfcV putative fimbrial protein YfcV

//

>NGHB#2285 202

CMPLX not

b2341 fadJ 3-hydroxyacyl-CoA dehydrogenase FadJ

b2340 sixA phosphohistidine phosphatase SixA

//

>NGHB#2286 -1

CMPLX not

b2342 fadI 3-ketoacyl-CoA thiolase FadI

b2341 fadJ 3-hydroxyacyl-CoA dehydrogenase FadJ

//

>NGHB#2287 180

CMPLX not

b2343 yfcZ DUF406 domain-containing protein YfcZ

b2342 fadI 3-ketoacyl-CoA thiolase FadI

//

>NGHB#2289 365

CMPLX not

b2344 fadL long-chain fatty acid outer membranechannel/bacteriophage T2 receptor

b2345 yfdF protein YfdF

//

>NGHB#2292 311

CMPLX not

b2347 yfdC inner membrane protein YfdC

b2349 intS CPS-53 (KpLE1) prophage; prophage CPS-53integrase

//

>NGHB#2293 152

CMPLX not

b2349 intS CPS-53 (KpLE1) prophage; prophage CPS-53integrase

b2350 yfdG CPS-53 (KpLE1) prophage; putativebactoprenol-linked glucose translocase

//

>NGHB#2294 -4

CMPLX not

b2350 yfdG CPS-53 (KpLE1) prophage; putativebactoprenol-linked glucose translocase

b2351 yfdH CPS-53 (KpLE1) prophage; bactoprenol glucosyltransferase

//

>NGHB#2295 -4

CMPLX not

b2351 yfdH CPS-53 (KpLE1) prophage; bactoprenol glucosyltransferase

b2352 yfdI CPS-53 (KpLE1) prophage; serotype-specificglucosyl transferase YfdI

//

>NGHB#2297 433

CMPLX not

b2354 yfdK CPS-53 (KpLE1) prophage; putative tail fiberassembly protein YfdK

b4780 yodE protein YodE

//

>NGHB#2298 594

CMPLX not

b2356 yfdM CPS-53 (KpLE1) prophage; putativemethyltransferase YfdM

b2354 yfdK CPS-53 (KpLE1) prophage; putative tail fiberassembly protein YfdK

//

>NGHB#2299 -1

CMPLX ---

b2357 yfdN CPS-53 (KpLE1) prophage; uncharacterized proteinYfdN

b2356 yfdM CPS-53 (KpLE1) prophage; putativemethyltransferase YfdM

//

>NGHB#2300 -4

CMPLX ---

b2358 yfdO CPS-53 (KpLE1) prophage; protein YdfO

b2357 yfdN CPS-53 (KpLE1) prophage; uncharacterized proteinYfdN

//

>NGHB#2302 65

CMPLX ---

b2359 yfdP CPS-53 (KpLE1) prophage; uncharacterized proteinYfdP

b2360 yfdQ CPS-53 (KpLE1) prophage; DUF2303domain-containing protein YfdQ

//

>NGHB#2303 127

CMPLX not

b2360 yfdQ CPS-53 (KpLE1) prophage; DUF2303domain-containing protein YfdQ

b2361 yfdR CPS-53 (KpLE1) prophage; 5'-deoxynucleotidase

//

>NGHB#2304 -10

CMPLX not

b2361 yfdR CPS-53 (KpLE1) prophage; 5'-deoxynucleotidase

b2362 yfdS CPS-53 (KpLE1) prophage; protein YfdS

//

>NGHB#2305 -1

CMPLX not

b2362 yfdS CPS-53 (KpLE1) prophage; protein YfdS

b2363 yfdT CPS-53 (KpLE1) prophage; protein YfdT

//

>NGHB#2306 -85

CMPLX ---

b2363 yfdT CPS-53 (KpLE1) prophage; protein YfdT

b4545 ypdJ CPS-53 (KpLE1) prophage; putativeuncharacterized protein YpdJ

//

>NGHB#2307 75

CMPLX ---

b4545 ypdJ CPS-53 (KpLE1) prophage; putativeuncharacterized protein YpdJ

b4501 torI CPS-53 (KpLE1) prophage; prophage CPS-53 recombination directionality factor and response regulatorinhibitor

//

>NGHB#2310 17

CMPLX not

b2365 dsdX D-serine transporter

b2366 dsdA D-serine ammonia-lyase

//

>NGHB#2312 -1

CMPLX yes

b2368 emrK tripartite efflux pump membrane fusion proteinEmrK

b2367 emrY tripartite efflux pump membrane subunit EmrY

//

>NGHB#2314 -4

CMPLX not

b4781 evgL protein EvgL

b2369 evgA DNA-binding transcriptional activator EvgA

//

>NGHB#2315 4

CMPLX not

b2369 evgA DNA-binding transcriptional activator EvgA

b2370 evgS sensor histidine kinase EvgS

//

>NGHB#2317 73

CMPLX not

b2372 yfdV putative transport protein YfdV

b2371 yfdE acetyl-CoA:oxalate CoA-transferase

//

>NGHB#2318 69

CMPLX not

b2373 oxc oxalyl-CoA decarboxylase

b2372 yfdV putative transport protein YfdV

//

>NGHB#2319 53

CMPLX not

b2374 frc formyl-CoA transferase

b2373 oxc oxalyl-CoA decarboxylase

//

>NGHB#2320 512

CMPLX not

b2375 yfdX protein YfdX

b2374 frc formyl-CoA transferase

//

>NGHB#2324 355

CMPLX not

b2378 lpxP palmitoleoyl acyltransferase

b4680 ypdK putative membrane protein YpdK

//

>NGHB#2327 14

CMPLX not

b2380 pyrS sensor histidine kinase PyrS

b2381 pyrR DNA-binding transcriptional activator PyrR

//

>NGHB#2328 12

CMPLX not

b2381 pyrR DNA-binding transcriptional activator PyrR

b2382 ypdC putative AraC-type DNA-binding transcriptionalregulator YpdC

//

>NGHB#2330 24

CMPLX not

b2384 ypdE aminopeptidase YpdE

b2383 fryA putative PTS multiphosphoryl transfer proteinFryA

//

>NGHB#2331 -1

CMPLX not

b2385 ypdF aminopeptidase YpdF

b2384 ypdE aminopeptidase YpdE

//

>NGHB#2332 14

CMPLX not

b2386 fryC putative PTS enzyme IIC component FryC

b2385 ypdF aminopeptidase YpdF

//

>NGHB#2333 21

CMPLX yes

b2387 fryB putative PTS enzyme IIB component FryB

b2386 fryC putative PTS enzyme IIC component FryC

//

>NGHB#2334 218

CMPLX not

b2388 glk glucokinase

b2387 fryB putative PTS enzyme IIB component FryB

//

>NGHB#2336 114

CMPLX not

b2389 yfeO putative transport protein YfeO

b2390 ypeC DUF2502 domain-containing protein YpeC

//

>NGHB#2339 86

CMPLX not

b2393 nupC nucleoside:H(+) symporter NupC

b2394 insL3 putative IS186/IS421 transposase

//

>NGHB#2342 1

CMPLX not

b2398 yfeC putative DNA-binding transcriptional regulatorYfeC

b2399 yfeD putative DNA-binding transcriptional regulatorYfeD

//

>NGHB#2344 920

CMPLX not

b2405 xapR DNA-binding transcriptional activator XapR

b2400 gltX glutamate--tRNA ligase

//

>NGHB#2345 251

CMPLX not

b2406 xapB xanthosine:H(+) symporter XapB

b2405 xapR DNA-binding transcriptional activator XapR

//

>NGHB#2346 59

CMPLX not

b2407 xapA xanthosine phosphorylase

b2406 xapB xanthosine:H(+) symporter XapB

//

>NGHB#2351 1

CMPLX not

b2411 ligA DNA ligase

b4546 ypeB PF12843 family protein YpeB

//

>NGHB#2352 70

CMPLX not

b2412 zipA cell division protein ZipA

b2411 ligA DNA ligase

//

>NGHB#2354 184

CMPLX not

b2413 cysZ sulfate:H(+) symporter

b2414 cysK cysteine synthase A

//

>NGHB#2355 383

CMPLX not

b2414 cysK cysteine synthase A

b2415 ptsH phosphocarrier protein HPr

//

>NGHB#2356 44

CMPLX yes

b2415 ptsH phosphocarrier protein HPr

b2416 ptsI PTS enzyme I

//

>NGHB#2357 40

CMPLX yes

b2416 ptsI PTS enzyme I

b2417 crr Enzyme IIA(Glc)

//

>NGHB#2360 32

CMPLX not

b2419 yfeK DUF5329 domain-containing protein YfeK

b2420 yfeS PF05406 family protein YfeS

//

>NGHB#2362 133

CMPLX not

b2422 cysA sulfate/thiosulfate ABC transporter ATP bindingsubunit

b2421 cysM cysteine synthase B

//

>NGHB#2363 -11

CMPLX yes

b2423 cysW sulfate/thiosulfate ABC transporter innermembrane subunit CysW

b2422 cysA sulfate/thiosulfate ABC transporter ATP bindingsubunit

//

>NGHB#2364 -1

CMPLX yes

b2424 cysU sulfate/thiosulfate ABC transporter innermembrane subunit CysU

b2423 cysW sulfate/thiosulfate ABC transporter innermembrane subunit CysW

//

>NGHB#2365 -1

CMPLX yes

b2425 cysP thiosulfate/sulfate ABC transporter periplasmicbinding protein CysP

b2424 cysU sulfate/thiosulfate ABC transporter innermembrane subunit CysU

//

>NGHB#2366 303

CMPLX not

b2426 ucpA oxidoreductase UcpA

b2425 cysP thiosulfate/sulfate ABC transporter periplasmicbinding protein CysP

//

>NGHB#2367 128

CMPLX not

b2427 murR DNA-binding transcriptional dual regulator MurR

b2426 ucpA oxidoreductase UcpA

//

>NGHB#2369 3

CMPLX not

b2428 murQ N-acetylmuramic acid 6-phosphate etherase

b2429 murP N-acetylmuramic acid-specific PTS enzyme IICBcomponent/anhydro-N-acetylmuramic acid transporter

//

>NGHB#2370 4

CMPLX not

b2429 murP N-acetylmuramic acid-specific PTS enzyme IICBcomponent/anhydro-N-acetylmuramic acid transporter

b2430 yfeW penicillin binding protein 4B

//

>NGHB#2372 95

CMPLX not

b2432 yfeY DUF1131 domain-containing lipoprotein YfeY

b2431 yfeX porphyrinogen peroxidase

//

>NGHB#2373 60

CMPLX not

b2433 yfeZ putative inner membrane protein

b2432 yfeY DUF1131 domain-containing lipoprotein YfeY

//

>NGHB#2374 -14

CMPLX not

b2434 ypeA putative acetyltransferase YpeA

b2433 yfeZ putative inner membrane protein

//

>NGHB#2376 3

CMPLX not

b2435 amiA N-acetylmuramoyl-L-alanine amidase A

b2436 hemF coproporphyrinogen III oxidase

//

>NGHB#2378 45

CMPLX not

b2438 eutK putative structural protein, ethanolamineutilization microcompartment

b2437 eutR putative AraC-type transcriptional regulatorEutR

//

>NGHB#2379 12

CMPLX yes

b2439 eutL putative structural protein, ethanolamineutilization microcompartment

b2438 eutK putative structural protein, ethanolamineutilization microcompartment

//

>NGHB#2380 9

CMPLX not

b2440 eutC ethanolamine ammonia-lyase subunit beta

b2439 eutL putative structural protein, ethanolamineutilization microcompartment

//

>NGHB#2381 20

CMPLX yes

b2441 eutB ethanolamine ammonia-lyase subunit alpha

b2440 eutC ethanolamine ammonia-lyase subunit beta

//

>NGHB#2383 190

CMPLX ---

b2442 intZ CPZ-55 prophage; putative phage integrase IntZ

b2443 yffL CPZ-55 prophage; uncharacterized protein YffL

//

>NGHB#2384 469

CMPLX ---

b2443 yffL CPZ-55 prophage; uncharacterized protein YffL

b2444 yffM CPZ-55 prophage; uncharacterized protein YffM

//

>NGHB#2385 11

CMPLX ---

b2444 yffM CPZ-55 prophage; uncharacterized protein YffM

b2445 yffN CPZ-55 prophage; uncharacterized protein YffN

//

>NGHB#2386 117

CMPLX ---

b2445 yffN CPZ-55 prophage; uncharacterized protein YffN

b2446 yffO CPZ-55 prophage; uncharacterized protein YffO

//

>NGHB#2387 -4

CMPLX ---

b2446 yffO CPZ-55 prophage; uncharacterized protein YffO

b2447 yffP CPZ-55 prophage; uncharacterized protein YffP

//

>NGHB#2388 474

CMPLX ---

b2447 yffP CPZ-55 prophage; uncharacterized protein YffP

b2448 yffQ CPZ-55 prophage; uncharacterized protein YffQ

//

>NGHB#2389 10

CMPLX ---

b2448 yffQ CPZ-55 prophage; uncharacterized protein YffQ

b2449 yffR CPZ-55 prophage; uncharacterized protein YffR

//

>NGHB#2390 150

CMPLX ---

b2449 yffR CPZ-55 prophage; uncharacterized protein YffR

b2450 yffS CPZ-55 prophage; uncharacterized protein YffS

//

>NGHB#2392 -4

CMPLX not

b2452 eutH putative ethanolamine permease EutH

b2451 eutA ethanolamine ammonia-lyase reactivase EutA

//

>NGHB#2393 216

CMPLX not

b2453 eutG putative alcohol dehydrogenase EutG

b2452 eutH putative ethanolamine permease EutH

//

>NGHB#2394 -11

CMPLX not

b2454 eutJ putative ethanolamine utilization chaperoninEutJ

b2453 eutG putative alcohol dehydrogenase EutG

//

>NGHB#2395 10

CMPLX not

b2455 eutE acetaldehyde dehydrogenase (acetylating) EutE

b2454 eutJ putative ethanolamine utilization chaperoninEutJ

//

>NGHB#2396 11

CMPLX not

b2456 eutN putative ethanolamine catabolic microcompartmentshell protein EutN

b2455 eutE acetaldehyde dehydrogenase (acetylating) EutE

//

>NGHB#2397 106

CMPLX yes

b2457 eutM putative ethanolamine catabolic microcompartmentshell protein EutM

b2456 eutN putative ethanolamine catabolic microcompartmentshell protein EutN

//

>NGHB#2398 38

CMPLX not

b2458 eutD phosphate acetyltransferase EutD

b2457 eutM putative ethanolamine catabolic microcompartmentshell protein EutM

//

>NGHB#2399 -4

CMPLX not

b2459 eutT putative ethanolamine utilization cobalaminadenosyltransferase

b2458 eutD phosphate acetyltransferase EutD

//

>NGHB#2400 -4

CMPLX not

b2460 eutQ putative ethanolamine utilization acetate kinaseEutQ

b2459 eutT putative ethanolamine utilization cobalaminadenosyltransferase

//

>NGHB#2401 -26

CMPLX yes

b2461 eutP putative ethanolamine utilization acetate kinaseEutP

b2460 eutQ putative ethanolamine utilization acetate kinaseEutQ

//

>NGHB#2402 12

CMPLX not

b2462 eutS putative ethanolamine catabolic microcompartmentshell protein EutS

b2461 eutP putative ethanolamine utilization acetate kinaseEutP

//

>NGHB#2403 292

CMPLX not

b2463 maeB NADP(+)-dependent malate dehydrogenase

b2462 eutS putative ethanolamine catabolic microcompartmentshell protein EutS

//

>NGHB#2405 19

CMPLX not

b2464 talA transaldolase A

b2465 tktB transketolase 2

//

>NGHB#2407 125

CMPLX not

b2467 nudK GDP-mannose hydrolase

b2466 ypfG DUF1176 domain-containing protein YpfG

//

>NGHB#2408 67

CMPLX not

b2468 aegA putative oxidoreductase AegA

b2467 nudK GDP-mannose hydrolase

//

>NGHB#2410 163

CMPLX not

b2469 narQ sensory histidine kinase NarQ

b2470 acrD multidrug efflux pump RND permease AcrD

//

>NGHB#2413 3

CMPLX not

b2471 yffB putative reductase YffB

b2472 dapE succinyl-diaminopimelate desuccinylase

//

>NGHB#2414 27

CMPLX not

b2472 dapE succinyl-diaminopimelate desuccinylase

b4547 ypfN UPF0370 protein YpfN

//

>NGHB#2416 73

CMPLX not

b2474 tmcA tRNA(Met) cytidine acetyltransferase

b2473 ypfH esterase YpfH

//

>NGHB#2417 14

CMPLX ---

b2475 ypfJ uncharacterized protein YpfJ

b2474 tmcA tRNA(Met) cytidine acetyltransferase

//

>NGHB#2418 167

CMPLX ---

b2476 purC phosphoribosylaminoimidazole-succinocarboxamidesynthase

b2475 ypfJ uncharacterized protein YpfJ

//

>NGHB#2419 212

CMPLX not

b2477 bamC outer membrane protein assembly factor BamC

b2476 purC phosphoribosylaminoimidazole-succinocarboxamidesynthase

//

>NGHB#2420 16

CMPLX not

b2478 dapA 4-hydroxy-tetrahydrodipicolinate synthase

b2477 bamC outer membrane protein assembly factor BamC

//

>NGHB#2422 -1

CMPLX not

b2479 gcvR putative transcriptional regulator GcvR

b2480 bcp thiol peroxidase

//

>NGHB#2423 252

CMPLX not

b2480 bcp thiol peroxidase

b2481 hyfA hydrogenase 4 component A

//

>NGHB#2424 -1

CMPLX yes

b2481 hyfA hydrogenase 4 component A

b2482 hyfB hydrogenase 4 component B

//

>NGHB#2425 10

CMPLX yes

b2482 hyfB hydrogenase 4 component B

b2483 hyfC hydrogenase 4 component C

//

>NGHB#2426 16

CMPLX yes

b2483 hyfC hydrogenase 4 component C

b2484 hyfD hydrogenase 4 component D

//

>NGHB#2427 11

CMPLX yes

b2484 hyfD hydrogenase 4 component D

b2485 hyfE hydrogenase 4 component E

//

>NGHB#2428 4

CMPLX yes

b2485 hyfE hydrogenase 4 component E

b2486 hyfF hydrogenase 4 component F

//

>NGHB#2429 -11

CMPLX yes

b2486 hyfF hydrogenase 4 component F

b2487 hyfG hydrogenase 4 catalytic subunit HyfG

//

>NGHB#2430 9

CMPLX yes

b2487 hyfG hydrogenase 4 catalytic subunit HyfG

b2488 hyfH hydrogenase 4 component H

//

>NGHB#2431 -4

CMPLX yes

b2488 hyfH hydrogenase 4 component H

b2489 hyfI hydrogenase 4 catalytic subunit HyfI

//

>NGHB#2432 -8

CMPLX not

b2489 hyfI hydrogenase 4 catalytic subunit HyfI

b2490 hyfJ putative hydrogenase 4 assembly protein

//

>NGHB#2433 29

CMPLX not

b2490 hyfJ putative hydrogenase 4 assembly protein

b2491 hyfR DNA-binding transcriptional activator HyfR

//

>NGHB#2434 21

CMPLX not

b2491 hyfR DNA-binding transcriptional activator HyfR

b2492 focB formate channel FocB

//

>NGHB#2437 20

CMPLX not

b2494 bepA beta-barrel assembly-enhancing protease

b2495 yfgD putative oxidoreductase YfgD

//

>NGHB#2439 94

CMPLX not

b2497 uraA uracil:H(+) symporter UraA

b2496 hda inibitor of reinitiation of DNA replication

//

>NGHB#2440 85

CMPLX not

b2498 upp uracil phosphoribosyltransferase

b2497 uraA uracil:H(+) symporter UraA

//

>NGHB#2442 -1

CMPLX not

b2499 purM phosphoribosylformylglycinamide cyclo-ligase

b2500 purN phosphoribosylglycinamide formyltransferase 1

//

>NGHB#2443 171

CMPLX not

b2500 purN phosphoribosylglycinamide formyltransferase 1

b2501 ppk polyphosphate kinase

//

>NGHB#2444 4

CMPLX not

b2501 ppk polyphosphate kinase

b2502 ppx exopolyphosphatase

//

>NGHB#2447 310

CMPLX not

b2504 yfgG nickel/cobalt stress response protein YfgG

b2505 yfgH lipoprotein YfgH

//

>NGHB#2448 15

CMPLX not

b2505 yfgH lipoprotein YfgH

b2506 yfgI nalidixic acid resistance protein YfgI

//

>NGHB#2450 68

CMPLX not

b2508 guaB inosine 5'-monophosphate dehydrogenase

b2507 guaA GMP synthetase

//

>NGHB#2453 69

CMPLX not

b2511 der 50S ribosomal subunit stability factor

b2510 yfgJ zinc ribbon domain-containing protein YfgJ

//

>NGHB#2454 117

CMPLX not

b2512 bamB outer membrane protein assembly factor BamB

b2511 der 50S ribosomal subunit stability factor

//

>NGHB#2455 10

CMPLX not

b2513 yfgM ancillary SecYEG translocon subunit

b2512 bamB outer membrane protein assembly factor BamB

//

>NGHB#2456 17

CMPLX not

b2514 hisS histidine--tRNA ligase

b2513 yfgM ancillary SecYEG translocon subunit

//

>NGHB#2457 110

CMPLX not

b2515 ispG (E)-4-hydroxy-3-methylbut-2-enyl-diphosphatesynthase (flavodoxin)

b2514 hisS histidine--tRNA ligase

//

>NGHB#2458 26

CMPLX not

b2516 rodZ cytoskeleton protein RodZ

b2515 ispG (E)-4-hydroxy-3-methylbut-2-enyl-diphosphatesynthase (flavodoxin)

//

>NGHB#2459 284

CMPLX not

b2517 rlmN 23S rRNA m(2)A2503 methyltransferase/tRNAm(2)A37 methyltransferase

b2516 rodZ cytoskeleton protein RodZ

//

>NGHB#2460 149

CMPLX not

b2518 ndk nucleoside diphosphate kinase

b2517 rlmN 23S rRNA m(2)A2503 methyltransferase/tRNAm(2)A37 methyltransferase

//

>NGHB#2461 148

CMPLX not

b2519 pbpC peptidoglycan glycosyltransferase PbpC

b2518 ndk nucleoside diphosphate kinase

//

>NGHB#2462 0

CMPLX not

b2520 yfhM alpha2-macroglobulin

b2519 pbpC peptidoglycan glycosyltransferase PbpC

//

>NGHB#2465 141

CMPLX not

b2523 pepB aminopeptidase B

b2522 sseB protein SseB

//

>NGHB#2466 177

CMPLX not

b2524 iscX accessory iron-sulfur cluster assembly proteinIscX

b2523 pepB aminopeptidase B

//

>NGHB#2467 11

CMPLX not

b2525 fdx reduced ferredoxin

b2524 iscX accessory iron-sulfur cluster assembly proteinIscX

//

>NGHB#2468 1

CMPLX not

b2526 hscA iron-sulfur cluster biosynthesis chaperone HscA

b2525 fdx reduced ferredoxin

//

>NGHB#2469 16

CMPLX yes

b2527 hscB [Fe-S] cluster biosynthesis co-chaperone HscB

b2526 hscA iron-sulfur cluster biosynthesis chaperone HscA

//

>NGHB#2470 95

CMPLX not

b2528 iscA iron-sulfur cluster insertion protein IscA

b2527 hscB [Fe-S] cluster biosynthesis co-chaperone HscB

//

>NGHB#2471 16

CMPLX yes

b2529 iscU scaffold protein for iron-sulfur clusterassembly

b2528 iscA iron-sulfur cluster insertion protein IscA

//

>NGHB#2472 27

CMPLX not

b2530 iscS cysteine desulfurase IscS

b2529 iscU scaffold protein for iron-sulfur clusterassembly

//

>NGHB#2473 111

CMPLX not

b2531 iscR DNA-binding transcriptional dual regulator IscR

b2530 iscS cysteine desulfurase IscS

//

>NGHB#2474 451

CMPLX not

b2532 trmJ tRNA Cm32/Um32 methyltransferase

b2531 iscR DNA-binding transcriptional dual regulator IscR

//

>NGHB#2476 144

CMPLX not

b2533 suhB inositol-phosphate phosphatase

b2534 yfhR putative peptidase

//

>NGHB#2477 190

CMPLX not

b2534 yfhR putative peptidase

b2535 csiE stationary phase inducible protein CsiE

//

>NGHB#2479 159

CMPLX not

b2537 hcaR DNA-binding transcriptional dual regulator HcaR

b2536 hcaT putative 3-phenylpropionate transporter

//

>NGHB#2480 -891

CMPLX not

b4706 iroK protein IroK

b2537 hcaR DNA-binding transcriptional dual regulator HcaR

//

>NGHB#2482 -4

CMPLX yes

b2538 hcaE putative 3-phenylpropionate/cinnamatedioxygenase subunit alpha

b2539 hcaF putative 3-phenylpropionate/cinnamatedioxygenase subunit beta

//

>NGHB#2483 -1

CMPLX yes

b2539 hcaF putative 3-phenylpropionate/cinnamatedioxygenase subunit beta

b2540 hcaC putative 3-phenylpropionate/cinnamatedioxygenase ferredoxin subunit

//

>NGHB#2484 -4

CMPLX not

b2540 hcaC putative 3-phenylpropionate/cinnamatedioxygenase ferredoxin subunit

b2541 hcaB 2,3-dihydroxy-2,3-dihydrophenylpropionatedehydrogenase

//

>NGHB#2485 9

CMPLX not

b2541 hcaB 2,3-dihydroxy-2,3-dihydrophenylpropionatedehydrogenase

b2542 hcaD putative 3-phenylpropionate/cinnamatedioxygenase ferredoxin reductase subunit

//

>NGHB#2486 96

CMPLX not

b2542 hcaD putative 3-phenylpropionate/cinnamatedioxygenase ferredoxin reductase subunit

b2543 yphA putative inner membrane protein

//

>NGHB#2488 11

CMPLX not

b2545 yphC putative zinc-binding dehydrogenase YphC

b2544 yphB putative aldose 1-epimerase YphB

//

>NGHB#2489 65

CMPLX not

b2546 yphD putative ABC transporter membrane subunit YphD

b2545 yphC putative zinc-binding dehydrogenase YphC

//

>NGHB#2490 24

CMPLX yes

b2547 yphE putative ABC transporter ATP-binding proteinYphE

b2546 yphD putative ABC transporter membrane subunit YphD

//

>NGHB#2491 22

CMPLX yes

b2548 yphF putative ABC transporter periplasmic bindingprotein YphF

b2547 yphE putative ABC transporter ATP-binding proteinYphE

//

>NGHB#2492 96

CMPLX yes

b2549 yphG DUF5107 domain-containing protein YphG

b2548 yphF putative ABC transporter periplasmic bindingprotein YphF

//

>NGHB#2497 60

CMPLX not

b2554 glrR DNA-binding transcriptional activator GlrR

b2553 glnB nitrogen regulatory protein PII-1

//

>NGHB#2498 -11

CMPLX not

b2555 qseG outer membrane lipoprotein QseG

b2554 glrR DNA-binding transcriptional activator GlrR

//

>NGHB#2499 164

CMPLX not

b2556 glrK histidine kinase GlrK

b2555 qseG outer membrane lipoprotein QseG

//

>NGHB#2500 557

CMPLX not

b2557 purL phosphoribosylformylglycinamide synthetase

b2556 glrK histidine kinase GlrK

//

>NGHB#2503 57

CMPLX not

b2560 pgpC phosphatidylglycerophosphatase C

b2559 tadA tRNA adenosine(34) deaminase

//

>NGHB#2505 55

CMPLX not

b2561 yfhH putative DNA-binding transcriptional regulatorYfhH

b2562 yfhL putative 4Fe-4S cluster-containing protein YfhL

//

>NGHB#2507 420

CMPLX not

b2563 acpS holo-[acyl-carrier-protein] synthase

b4687 shoB toxic peptide ShoB

//

>NGHB#2508 -1

CMPLX not

b2564 pdxJ pyridoxine 5'-phosphate synthase

b2563 acpS holo-[acyl-carrier-protein] synthase

//

>NGHB#2509 11

CMPLX not

b2565 recO DNA repair protein RecO

b2564 pdxJ pyridoxine 5'-phosphate synthase

//

>NGHB#2510 11

CMPLX not

b2566 era 30S ribosomal subunit maturation GTPase Era

b2565 recO DNA repair protein RecO

//

>NGHB#2511 -4

CMPLX not

b2567 rnc RNase III

b2566 era 30S ribosomal subunit maturation GTPase Era

//

>NGHB#2512 271

CMPLX not

b2568 lepB signal peptidase I

b2567 rnc RNase III

//

>NGHB#2513 15

CMPLX not

b2569 lepA 30S ribosomal subunit biogenesis factor LepA

b2568 lepB signal peptidase I

//

>NGHB#2514 197

CMPLX not

b2570 rseC protein RseC

b2569 lepA 30S ribosomal subunit biogenesis factor LepA

//

>NGHB#2515 -4

CMPLX yes

b2571 rseB anti-sigma factor stabilizing protein RseB

b2570 rseC protein RseC

//

>NGHB#2516 -1

CMPLX yes

b2572 rseA anti-sigma-E factor RseA

b2571 rseB anti-sigma factor stabilizing protein RseB

//

>NGHB#2517 32

CMPLX yes

b2573 rpoE RNA polymerase sigma E factor

b2572 rseA anti-sigma-E factor RseA

//

>NGHB#2518 -4

CMPLX not

b4725 rseD rpoE leader peptide

b2573 rpoE RNA polymerase sigma E factor

//

>NGHB#2528 68

CMPLX not

b2582 trxC reduced thioredoxin 2

b2583 tapT tRNA 3-amino-3-carboxypropyltransferase

//

>NGHB#2529 31

CMPLX not

b2583 tapT tRNA 3-amino-3-carboxypropyltransferase

b2584 patZ peptidyl-lysine N-acetyltransferase

//

>NGHB#2530 88

CMPLX not

b2584 patZ peptidyl-lysine N-acetyltransferase

b4782 pssL protein PssL

//

>NGHB#2531 -17

CMPLX not

b4782 pssL protein PssL

b2585 pssA phosphatidylserine synthase

//

>NGHB#2532 45

CMPLX not

b2585 pssA phosphatidylserine synthase

b2586 yfiM protein YfiM

//

>NGHB#2536 573

CMPLX not

b2593 pgeF polyphenol oxidase YfiH

b2592 clpB ClpB80

//

>NGHB#2537 -4

CMPLX not

b2594 rluD 23S rRNA pseudouridine(1911/1915/1917) synthase

b2593 pgeF polyphenol oxidase YfiH

//

>NGHB#2539 270

CMPLX not

b2595 bamD outer membrane protein assembly factor BamD

b2597 raiA ribosome-associated inhibitor A

//

>NGHB#2540 103

CMPLX not

b2597 raiA ribosome-associated inhibitor A

b2598 pheL phe operon leader peptide

//

>NGHB#2541 98

CMPLX not

b2598 pheL phe operon leader peptide

b2599 pheA fused chorismate mutase/prephenate dehydratase

//

>NGHB#2543 10

CMPLX not

b2601 aroF 3-deoxy-7-phosphoheptulonate synthase,Tyr-sensitive

b2600 tyrA fused chorismate mutase/prephenatedehydrogenase

//

>NGHB#2545 149

CMPLX not

b2602 yfiL DUF2799 domain-containing lipoprotein YfiL

b2603 yfiR DUF4154 domain-containing protein YfiR

//

>NGHB#2546 -11

CMPLX not

b2603 yfiR DUF4154 domain-containing protein YfiR

b2604 dgcN diguanylate cyclase DgcN

//

>NGHB#2547 15

CMPLX not

b2604 dgcN diguanylate cyclase DgcN

b2605 yfiB lipoprotein YfiB

//

>NGHB#2549 41

CMPLX not

b2607 trmD tRNA m(1)G37 methyltransferase

b2606 rplS 50S ribosomal subunit protein L19

//

>NGHB#2550 30

CMPLX not

b2608 rimM ribosome maturation factor RimM

b2607 trmD tRNA m(1)G37 methyltransferase

//

>NGHB#2551 18

CMPLX not

b2609 rpsP 30S ribosomal subunit protein S16

b2608 rimM ribosome maturation factor RimM

//

>NGHB#2552 248

CMPLX not

b2610 ffh signal recognition particle protein component

b2609 rpsP 30S ribosomal subunit protein S16

//

>NGHB#2554 20

CMPLX not

b2611 ypjD cytochrome c assembly family protein

b4461 yfjD UPF0053 family inner membrane protein YfjD

//

>NGHB#2557 85

CMPLX not

b2615 nadK NAD kinase

b2616 recN DNA repair protein RecN

//

>NGHB#2558 148

CMPLX not

b2616 recN DNA repair protein RecN

b2617 bamE outer membrane protein assembly factor BamE

//

>NGHB#2560 -11

CMPLX not

b2619 ratA ribosome association toxin RatA

b2618 yfjF putative component of the Rsx system

//

>NGHB#2562 780

CMPLX not

b2620 smpB SsrA-binding protein

b2622 intA CP4-57 prophage; integrase IntA

//

>NGHB#2565 128

CMPLX not

b2624 alpA CP4-57 prophage; DNA-binding transcriptionalactivator AlpA

b2625 yfjI CP4-57 prophage; protein YfjI

//

>NGHB#2566 152

CMPLX not

b2625 yfjI CP4-57 prophage; protein YfjI

b2626 yfjJ CP4-57 prophage; protein YfjJ

//

>NGHB#2568 -4

CMPLX not

b2628 abpA CP4-57 prophage; anti-bacteriophage protein

b2627 abpB CP4-57 prophage; putative helicase YfjK

//

>NGHB#2569 359

CMPLX not

b2629 yfjM CP4-57 prophage; protein YfjM

b2628 abpA CP4-57 prophage; anti-bacteriophage protein

//

>NGHB#2571 -8

CMPLX yes

b2630 rnlA CP4-57 prophage; RNase LS, toxin of the RnlABtoxin-antitoxin system

b2631 rnlB CP4-57 prophage; antitoxin RnlB

//

>NGHB#2572 354

CMPLX not

b2631 rnlB CP4-57 prophage; antitoxin RnlB

b2632 yfjP CP4-57 prophage; putative GTP-binding proteinYfjP

//

>NGHB#2573 91

CMPLX not

b2632 yfjP CP4-57 prophage; putative GTP-binding proteinYfjP

b2633 yfjQ CP4-57 prophage; DUF932 domain-containingprotein YfjQ

//

>NGHB#2574 216

CMPLX not

b2633 yfjQ CP4-57 prophage; DUF932 domain-containingprotein YfjQ

b2634 yfjR CP4-57 prophage; putative DNA-bindingtranscriptional regulator YfjR

//

>NGHB#2575 40

CMPLX ---

b2634 yfjR CP4-57 prophage; putative DNA-bindingtranscriptional regulator YfjR

b2635 ypjK CP4-57 prophage; uncharacterized protein YpjK

//

>NGHB#2576 -1

CMPLX ---

b2635 ypjK CP4-57 prophage; uncharacterized protein YpjK

b2636 yfjS CP4-57 prophage; inner membrane lipoproteinYfjS

//

>NGHB#2577 23

CMPLX ---

b2636 yfjS CP4-57 prophage; inner membrane lipoproteinYfjS

b2637 yfjT CP4-57 prophage; uncharacterized protein YfjT

//

>NGHB#2580 897

CMPLX ---

b2642 yfjW CP4-57 prophage; uncharacterized protein YfjW

b2643 yfjX CP4-57 prophage; putative antirestrictionprotein YfjX

//

>NGHB#2581 8

CMPLX not

b2643 yfjX CP4-57 prophage; putative antirestrictionprotein YfjX

b2644 yfjY CP4-57 prophage; RadC-like JAB domain-containingprotein YfjY

//

>NGHB#2582 8

CMPLX not

b2644 yfjY CP4-57 prophage; RadC-like JAB domain-containingprotein YfjY

b4548 ypjJ CP4-57 prophage; DUF987 domain-containingprotein YpjJ

//

>NGHB#2583 37

CMPLX not

b4548 ypjJ CP4-57 prophage; DUF987 domain-containingprotein YpjJ

b2645 yfjZ CP4-57 prophage; putative antitoxin of theYpjF-YfjZ toxin-antitoxin system

//

>NGHB#2584 20

CMPLX yes

b2645 yfjZ CP4-57 prophage; putative antitoxin of theYpjF-YfjZ toxin-antitoxin system

b2646 ypjF CP4-57 prophage; toxin of the YpjF-YfjZtoxin-antitoxin system

//

>NGHB#2586 911

CMPLX not

b2649 ypjB DUF5508 domain-containing protein YpjB

b2647 ypjA adhesin-like autotransporter YpjA

//

>NGHB#2587 99

CMPLX not

b2650 ypjC DUF5507 domain-containing protein YpjC

b2649 ypjB DUF5508 domain-containing protein YpjB

//

>NGHB#2589 19

CMPLX not

b2659 glaH glutarate hydroxylase GlaH

b2660 lhgD L-2-hydroxyglutarate dehydrogenase

//

>NGHB#2590 22

CMPLX not

b2660 lhgD L-2-hydroxyglutarate dehydrogenase

b2661 gabD succinate-semialdehyde dehydrogenase (NADP(+))GabD

//

>NGHB#2591 13

CMPLX not

b2661 gabD succinate-semialdehyde dehydrogenase (NADP(+))GabD

b2662 gabT 4-aminobutyrate aminotransferase GabT

//

>NGHB#2592 237

CMPLX not

b2662 gabT 4-aminobutyrate aminotransferase GabT

b2663 gabP 4-aminobutanoate:H(+) symporter

//

>NGHB#2593 20

CMPLX not

b2663 gabP 4-aminobutanoate:H(+) symporter

b2664 glaR DNA-binding transcriptional repressor GlaR

//

>NGHB#2595 83

CMPLX not

b2666 yqaE Pmp3 family protein YqaE

b2665 kbp K(+) binding protein

//

>NGHB#2597 9

CMPLX not

b2667 ygaV putative DNA-binding transcriptional regulatorYgaV

b2668 ygaP thiosulfate sulfurtransferase YgaP

//

>NGHB#2602 247

CMPLX not

b2672 ygaM DUF883 domain-containing protein YgaM

b2673 nrdH glutaredoxin-like protein NrdH

//

>NGHB#2603 -4

CMPLX not

b2673 nrdH glutaredoxin-like protein NrdH

b2674 nrdI dimanganese-tyrosyl radical cofactor maintenanceflavodoxin NrdI

//

>NGHB#2604 -28

CMPLX not

b2674 nrdI dimanganese-tyrosyl radical cofactor maintenanceflavodoxin NrdI

b2675 nrdE ribonucleoside-diphosphate reductase 2 subunitalpha

//

>NGHB#2605 9

CMPLX yes

b2675 nrdE ribonucleoside-diphosphate reductase 2 subunitalpha

b2676 nrdF ribonucleoside-diphosphate reductase 2 subunitbeta

//

>NGHB#2606 353

CMPLX not

b2676 nrdF ribonucleoside-diphosphate reductase 2 subunitbeta

b2677 proV glycine betaine ABC transporter ATP bindingsubunit ProV

//

>NGHB#2607 -8

CMPLX yes

b2677 proV glycine betaine ABC transporter ATP bindingsubunit ProV

b2678 proW glycine betaine ABC transporter membrane subunitProW

//

>NGHB#2608 57

CMPLX yes

b2678 proW glycine betaine ABC transporter membrane subunitProW

b2679 proX glycine betaine ABC transporter periplasmicbinding protein ProX

//

>NGHB#2609 1492

CMPLX not

b2679 proX glycine betaine ABC transporter periplasmicbinding protein ProX

b2682 ygaZ L-valine exporter, YgaZ component

//

>NGHB#2610 -11

CMPLX yes

b2682 ygaZ L-valine exporter, YgaZ component

b2683 ygaH L-valine exporter, YgaH component

//

>NGHB#2611 90

CMPLX not

b2683 ygaH L-valine exporter, YgaH component

b2684 mprA DNA-binding transcriptional repressor MprA

//

>NGHB#2612 126

CMPLX not

b2684 mprA DNA-binding transcriptional repressor MprA

b2685 emrA multidrug efflux pump membrane fusion proteinEmrA

//

>NGHB#2613 16

CMPLX yes

b2685 emrA multidrug efflux pump membrane fusion proteinEmrA

b2686 emrB multidrug efflux pump membrane subunit EmrB

//

>NGHB#2615 149

CMPLX not

b2688 gshA glutamate--cysteine ligase

b2687 luxS S-ribosylhomocysteine lyase

//

>NGHB#2616 72

CMPLX not

b2689 yqaA DedA family protein YqaA

b2688 gshA glutamate--cysteine ligase

//

>NGHB#2617 -4

CMPLX not

b2690 yqaB fructose-1-phosphate phosphatase YqaB

b2689 yqaA DedA family protein YqaA

//

>NGHB#2618 1457

CMPLX not

b2696 csrA carbon storage regulator

b2690 yqaB fructose-1-phosphate phosphatase YqaB

//

>NGHB#2619 234

CMPLX not

b2697 alaS alanine--tRNA ligase/DNA-binding transcriptionalrepressor

b2696 csrA carbon storage regulator

//

>NGHB#2620 127

CMPLX not

b2698 recX RecA inhibitor RecX

b2697 alaS alanine--tRNA ligase/DNA-binding transcriptionalrepressor

//

>NGHB#2621 68

CMPLX not

b2699 recA DNA recombination/repair protein RecA

b2698 recX RecA inhibitor RecX

//

>NGHB#2622 79

CMPLX not

b2700 pncC NMN aminohydrolase

b2699 recA DNA recombination/repair protein RecA

//

>NGHB#2623 144

CMPLX not

b2701 mltB membrane-bound lytic murein transglycosylase B

b2700 pncC NMN aminohydrolase

//

>NGHB#2625 -4

CMPLX yes

b2702 srlA sorbitol-specific PTS enzyme IIC2 component

b2703 srlE sorbitol-specific PTS enzyme IIBC1 component

//

>NGHB#2626 10

CMPLX yes

b2703 srlE sorbitol-specific PTS enzyme IIBC1 component

b2704 srlB sorbitol-specific PTS enzyme IIA component

//

>NGHB#2627 3

CMPLX not

b2704 srlB sorbitol-specific PTS enzyme IIA component

b2705 srlD sorbitol-6-phosphate 2-dehydrogenase

//

>NGHB#2628 104

CMPLX not

b2705 srlD sorbitol-6-phosphate 2-dehydrogenase

b2706 gutM DNA-binding transcriptional activator GutM

//

>NGHB#2629 66

CMPLX not

b2706 gutM DNA-binding transcriptional activator GutM

b2707 srlR DNA-binding transcriptional repressor SrlR

//

>NGHB#2630 -8

CMPLX not

b2707 srlR DNA-binding transcriptional repressor SrlR

b2708 gutQ D-arabinose 5-phosphate isomerase GutQ

//

>NGHB#2633 -4

CMPLX not

b2710 norV anaerobic nitric oxide reductaseflavorubredoxin

b2711 norW NADH:flavorubredoxin reductase

//

>NGHB#2635 152

CMPLX not

b2713 hydN putative electron transport protein HydN

b2712 hypF carbamoyl--[HypE] ligase

//

>NGHB#2636 148

CMPLX not

b2714 ascG DNA-binding transcriptional repressor AscG

b2713 hydN putative electron transport protein HydN

//

>NGHB#2638 8

CMPLX not

b2715 ascF beta-glucoside specific PTS enzyme IIBCcomponent

b2716 ascB 6-phospho-beta-glucosidase AscB

//

>NGHB#2640 -8

CMPLX not

b2718 hycH formate hydrogenlyase assembly protein

b2717 hycI hydrogenase 3 maturation protease

//

>NGHB#2641 -4

CMPLX not

b2719 hycG formate hydrogenlyase subunit HycG

b2718 hycH formate hydrogenlyase assembly protein

//

>NGHB#2642 -1

CMPLX yes

b2720 hycF formate hydrogenlyase subunit HycF

b2719 hycG formate hydrogenlyase subunit HycG

//

>NGHB#2643 9

CMPLX yes

b2721 hycE formate hydrogenlyase subunit HycE

b2720 hycF formate hydrogenlyase subunit HycF

//

>NGHB#2644 17

CMPLX yes

b2722 hycD formate hydrogenlyase subunit HycD

b2721 hycE formate hydrogenlyase subunit HycE

//

>NGHB#2645 2

CMPLX yes

b2723 hycC formate hydrogenlyase subunit HycC

b2722 hycD formate hydrogenlyase subunit HycD

//

>NGHB#2646 -4

CMPLX yes

b2724 hycB formate hydrogenlyase subunit HycB

b2723 hycC formate hydrogenlyase subunit HycC

//

>NGHB#2647 124

CMPLX not

b2725 hycA regulator of the transcriptional regulator FhlA

b2724 hycB formate hydrogenlyase subunit HycB

//

>NGHB#2649 3

CMPLX not

b2726 hypA hydrogenase 3 nickel incorporation protein HypA

b2727 hypB hydrogenase isoenzymes nickel incorporationprotein HypB

//

>NGHB#2650 -10

CMPLX yes

b2727 hypB hydrogenase isoenzymes nickel incorporationprotein HypB

b2728 hypC hydrogenase 3 maturation protein HypC

//

>NGHB#2651 -1

CMPLX yes

b2728 hypC hydrogenase 3 maturation protein HypC

b2729 hypD Fe-(CN)2CO cofactor assembly scaffold proteinHypD

//

>NGHB#2652 -4

CMPLX yes

b2729 hypD Fe-(CN)2CO cofactor assembly scaffold proteinHypD

b2730 hypE hydrogenase maturation protein, carbamoyldehydratase

//

>NGHB#2653 73

CMPLX not

b2730 hypE hydrogenase maturation protein, carbamoyldehydratase

b2731 fhlA DNA-binding transcriptional activator FhlA

//

>NGHB#2656 105

CMPLX not

b2733 mutS DNA mismatch repair protein MutS

b2734 pphB phosphoprotein phosphatase 2

//

>NGHB#2659 -4

CMPLX not

b2736 ygbJ putative L-threonate dehydrogenase

b2737 ygbK putative 3-oxo-tetronate kinase YgbK

//

>NGHB#2660 91

CMPLX not

b2737 ygbK putative 3-oxo-tetronate kinase YgbK

b2738 ygbL putative 3-oxo-tetronate 4-phosphatedecarboxylase YgbL

//

>NGHB#2661 4

CMPLX not

b2738 ygbL putative 3-oxo-tetronate 4-phosphatedecarboxylase YgbL

b2739 ygbM putative 2-oxo-tetronate isomerase YgbM

//

>NGHB#2662 88

CMPLX not

b2739 ygbM putative 2-oxo-tetronate isomerase YgbM

b2740 ygbN putative transporter YgbN

//

>NGHB#2664 62

CMPLX not

b2742 nlpD murein hydrolase activator NlpD

b2741 rpoS RNA polymerase, sigma S (sigma 38) factor

//

>NGHB#2665 139

CMPLX not

b2743 pcm L-isoaspartate protein carboxylmethyltransferasetype II

b2742 nlpD murein hydrolase activator NlpD

//

>NGHB#2666 -7

CMPLX not

b2744 umpG broad specificity 5'(3')-nucleotidase andpolyphosphatase

b2743 pcm L-isoaspartate protein carboxylmethyltransferasetype II

//

>NGHB#2667 -20

CMPLX not

b2745 truD tRNA pseudouridine(13) synthase

b2744 umpG broad specificity 5'(3')-nucleotidase andpolyphosphatase

//

>NGHB#2668 -4

CMPLX not

b2746 ispF 2-C-methyl-D-erythritol 2,4-cyclodiphosphatesynthase

b2745 truD tRNA pseudouridine(13) synthase

//

>NGHB#2669 -1

CMPLX not

b2747 ispD 2-C-methyl-D-erythritol 4-phosphatecytidylyltransferase

b2746 ispF 2-C-methyl-D-erythritol 2,4-cyclodiphosphatesynthase

//

>NGHB#2670 18

CMPLX not

b2748 ftsB cell division protein FtsB

b2747 ispD 2-C-methyl-D-erythritol 4-phosphatecytidylyltransferase

//

>NGHB#2671 193

CMPLX not

b2749 ygbE DUF3561 domain-containing inner membrane proteinYgbE

b2748 ftsB cell division protein FtsB

//

>NGHB#2672 49

CMPLX not

b2750 cysC adenylyl-sulfate kinase

b2749 ygbE DUF3561 domain-containing inner membrane proteinYgbE

//

>NGHB#2673 -1

CMPLX not

b2751 cysN sulfate adenylyltransferase subunit 1

b2750 cysC adenylyl-sulfate kinase

//

>NGHB#2674 1

CMPLX yes

b2752 cysD sulfate adenylyltransferase subunit 2

b2751 cysN sulfate adenylyltransferase subunit 1

//

>NGHB#2677 1

CMPLX yes

b2755 cas1 multifunctional nuclease Cas1

b2754 cas2 CRISPR-associated endoribonuclease Cas2

//

>NGHB#2678 15

CMPLX yes

b2756 casE pre-CRISPR RNA endonuclease

b2755 cas1 multifunctional nuclease Cas1

//

>NGHB#2679 -14

CMPLX yes

b2757 casD type I-E CRISPR system Cascade subunit CasD

b2756 casE pre-CRISPR RNA endonuclease

//

>NGHB#2680 2

CMPLX yes

b2758 casC type I-E CRISPR system Cascade subunit CasC

b2757 casD type I-E CRISPR system Cascade subunit CasD

//

>NGHB#2681 12

CMPLX yes

b2759 casB type I-E CRISPR system Cascade subunit CasB

b2758 casC type I-E CRISPR system Cascade subunit CasC

//

>NGHB#2682 -8

CMPLX yes

b2760 casA type I-E CRISPR system Cascade subunit CasA

b2759 casB type I-E CRISPR system Cascade subunit CasB

//

>NGHB#2683 414

CMPLX yes

b2761 cas3 CRISPR-associated endonuclease/helicase Cas3

b2760 casA type I-E CRISPR system Cascade subunit CasA

//

>NGHB#2684 358

CMPLX not

b2762 cysH phosphoadenosine phosphosulfate reductase

b2761 cas3 CRISPR-associated endonuclease/helicase Cas3

//

>NGHB#2685 74

CMPLX not

b2763 cysI sulfite reductase, hemoprotein subunit

b2762 cysH phosphoadenosine phosphosulfate reductase

//

>NGHB#2686 -1

CMPLX yes

b2764 cysJ sulfite reductase, flavoprotein subunit

b2763 cysI sulfite reductase, hemoprotein subunit

//

>NGHB#2688 77

CMPLX not

b2765 queD 6-carboxy-5,6,7,8-tetrahydropterin synthase

b2766 ygcN putative oxidoreductase with FAD/NAD(P)-bindingdomain

//

>NGHB#2689 -10

CMPLX not

b2766 ygcN putative oxidoreductase with FAD/NAD(P)-bindingdomain

b2767 ygcO putative 4Fe-4S cluster-containing protein

//

>NGHB#2690 16

CMPLX not

b2767 ygcO putative 4Fe-4S cluster-containing protein

b2768 ygcP putative anti-terminator regulatory protein

//

>NGHB#2692 -4

CMPLX not

b2770 ygcR putative flavoprotein

b2769 ygcQ putative flavoprotein

//

>NGHB#2693 -23

CMPLX not

b2771 ygcS putative transporter YgcS

b2770 ygcR putative flavoprotein

//

>NGHB#2694 93

CMPLX not

b4463 ygcU putative FAD-containing dehydrogenase

b2771 ygcS putative transporter YgcS

//

>NGHB#2695 69

CMPLX not

b2774 ygcW putative deoxygluconate dehydrogenase

b4463 ygcU putative FAD-containing dehydrogenase

//

>NGHB#2697 26

CMPLX not

b2775 yqcE putative transport protein YqcE

b2776 ygcE putative sugar kinase YgcE

//

>NGHB#2700 13

CMPLX not

b4682 yqcG cell envelope stress response protein YqcG

b2778 ygcG protein YgcG

//

>NGHB#2702 87

CMPLX not

b2780 pyrG CTP synthetase

b2779 eno enolase

//

>NGHB#2703 227

CMPLX not

b2781 mazG nucleoside triphosphate pyrophosphohydrolase

b2780 pyrG CTP synthetase

//

>NGHB#2704 70

CMPLX not

b2782 mazF endoribonuclease toxin MazF

b2781 mazG nucleoside triphosphate pyrophosphohydrolase

//

>NGHB#2705 -1

CMPLX yes

b2783 mazE antitoxin of the MazF-MazE toxin-antitoxinsystem MazE

b2782 mazF endoribonuclease toxin MazF

//

>NGHB#2706 77

CMPLX not

b2784 relA GDP/GTP pyrophosphokinase

b2783 mazE antitoxin of the MazF-MazE toxin-antitoxinsystem MazE

//

>NGHB#2707 47

CMPLX not

b2785 rlmD 23S rRNA m(5)U1939 methyltransferase

b2784 relA GDP/GTP pyrophosphokinase

//

>NGHB#2710 20

CMPLX not

b2788 gudX glucarate dehydratase-related protein

b2787 gudD D-glucarate dehydratase

//

>NGHB#2711 1

CMPLX not

b2789 gudP galactarate/D-glucarate transporter GudP

b2788 gudX glucarate dehydratase-related protein

//

>NGHB#2712 434

CMPLX not

b2790 yqcA putative flavodoxin YqcA

b2789 gudP galactarate/D-glucarate transporter GudP

//

>NGHB#2713 17

CMPLX not

b2791 truC tRNA pseudouridine(65) synthase

b2790 yqcA putative flavodoxin YqcA

//

>NGHB#2714 -1

CMPLX not

b2792 yqcC DUF446 domain-containing protein YqcC

b2791 truC tRNA pseudouridine(65) synthase

//

>NGHB#2715 621

CMPLX not

b2793 syd SecY-interacting protein

b2792 yqcC DUF446 domain-containing protein YqcC

//

>NGHB#2717 111

CMPLX not

b2794 queF 7-cyano-7-deazaguanine reductase

b2795 ppnN nucleotide 5'-monophosphate nucleosidase

//

>NGHB#2718 556

CMPLX not

b2795 ppnN nucleotide 5'-monophosphate nucleosidase

b2796 sdaC L-serine:H(+) symporter SdaC

//

>NGHB#2719 57

CMPLX not

b2796 sdaC L-serine:H(+) symporter SdaC

b2797 sdaB L-serine deaminase II

//

>NGHB#2720 111

CMPLX not

b2797 sdaB L-serine deaminase II

b2798 ygdG flap endonuclease

//

>NGHB#2722 27

CMPLX not

b2800 fucA L-fuculose-phosphate aldolase

b2799 fucO L-1,2-propanediol oxidoreductase

//

>NGHB#2724 32

CMPLX not

b2801 fucP L-fucose:H(+) symporter

b2802 fucI L-fucose isomerase

//

>NGHB#2725 108

CMPLX not

b2802 fucI L-fucose isomerase

b2803 fucK L-fuculokinase

//

>NGHB#2726 1

CMPLX not

b2803 fucK L-fuculokinase

b2804 fucU L-fucose mutarotase

//

>NGHB#2727 57

CMPLX not

b2804 fucU L-fucose mutarotase

b2805 fucR DNA-binding transcriptional activator FucR

//

>NGHB#2729 -8

CMPLX not

b2807 ygdD DUF423 domain-containing inner membrane proteinYgdD

b2806 rlmM 23S rRNA 2'-O-ribose C2498 methyltransferase

//

>NGHB#2730 18

CMPLX not

b2808 gcvA DNA-binding transcriptional dual regulator GcvA

b2807 ygdD DUF423 domain-containing inner membrane proteinYgdD

//

>NGHB#2731 350

CMPLX not

b2809 ygdI DUF903 domain-containing lipoprotein YgdI

b2808 gcvA DNA-binding transcriptional dual regulator GcvA

//

>NGHB#2733 -1

CMPLX not

b2810 csdA cysteine sulfinate desulfinase

b2811 csdE sulfur acceptor protein CsdE

//

>NGHB#2735 238

CMPLX not

b2813 mltA membrane-bound lytic murein transglycosylase A

b2812 tcdA tRNA threonylcarbamoyladenosine dehydratase

//

>NGHB#2736 578

CMPLX not

b2817 amiC N-acetylmuramoyl-L-alanine amidase C

b2813 mltA membrane-bound lytic murein transglycosylase A

//

>NGHB#2739 -1

CMPLX yes

b2820 recB exodeoxyribonuclease V subunit RecB

b2819 recD exodeoxyribonuclease V subunit RecD

//

>NGHB#2740 -8

CMPLX not

b2821 ptrA protease 3

b2820 recB exodeoxyribonuclease V subunit RecB

//

>NGHB#2741 175

CMPLX not

b2822 recC exodeoxyribonuclease V subunit RecC

b2821 ptrA protease 3

//

>NGHB#2742 12

CMPLX not

b2823 ppdC prepilin-type N-terminal cleavage/methylationdomain-containing protein PpdC

b2822 recC exodeoxyribonuclease V subunit RecC

//

>NGHB#2743 -16

CMPLX not

b2824 ygdB DUF2509 domain-containing protein YgdB

b2823 ppdC prepilin-type N-terminal cleavage/methylationdomain-containing protein PpdC

//

>NGHB#2744 -4

CMPLX not

b2825 ppdB prepilin-type N-terminal cleavage/methylationdomain-containing protein PpdB

b2824 ygdB DUF2509 domain-containing protein YgdB

//

>NGHB#2745 -10

CMPLX not

b2826 ppdA prepilin-type N-terminal cleavage/methylationdomain-containing protein PpdA

b2825 ppdB prepilin-type N-terminal cleavage/methylationdomain-containing protein PpdB

//

>NGHB#2746 183

CMPLX not

b2827 thyA thymidylate synthase

b2826 ppdA prepilin-type N-terminal cleavage/methylationdomain-containing protein PpdA

//

>NGHB#2747 6

CMPLX not

b2828 lgt phosphatidylglycerol--prolipoproteindiacylglyceryl transferase

b2827 thyA thymidylate synthase

//

>NGHB#2748 150

CMPLX not

b2829 ptsP phosphoenolpyruvate-protein phosphotransferasePtsP

b2828 lgt phosphatidylglycerol--prolipoproteindiacylglyceryl transferase

//

>NGHB#2749 12

CMPLX not

b2830 rppH RNA pyrophosphohydrolase

b2829 ptsP phosphoenolpyruvate-protein phosphotransferasePtsP

//

>NGHB#2750 352

CMPLX not

b4610 ygdT YgdT

b2830 rppH RNA pyrophosphohydrolase

//

>NGHB#2752 68

CMPLX not

b2831 mutH DNA mismatch repair protein MutH

b2832 ygdQ UPF0053 inner membrane protein YgdQ

//

>NGHB#2753 137

CMPLX not

b2832 ygdQ UPF0053 inner membrane protein YgdQ

b2833 ygdR DUF903 domain-containing lipoprotein YgdR

//

>NGHB#2754 107

CMPLX not

b2833 ygdR DUF903 domain-containing lipoprotein YgdR

b2834 tas putative NADP(H)-dependent aldo-keto reductaseTas

//

>NGHB#2756 -8

CMPLX not

b2836 aas fused 2-acylglycerophospho-ethanolamineacyltransferase/acyl-acyl carrier protein synthetase

b2835 lplT lysophospholipid transporter

//

>NGHB#2761 128

CMPLX not

b2841 araE arabinose:H(+) symporter

b2840 ygeA amino acid racemase YgeA

//

>NGHB#2762 314

CMPLX not

b2842 kduD putative 2-keto-3-deoxy-D-gluconatedehydrogenase

b2841 araE arabinose:H(+) symporter

//

>NGHB#2763 29

CMPLX not

b2843 kduI 5-dehydro-4-deoxy-D-glucuronate isomerase

b2842 kduD putative 2-keto-3-deoxy-D-gluconatedehydrogenase

//

>NGHB#2764 286

CMPLX not

b2844 yqeF putative acyltransferase

b2843 kduI 5-dehydro-4-deoxy-D-glucuronate isomerase

//

>NGHB#2766 459

CMPLX not

b2845 yqeG putative transporter YqeG

b2846 yqeH putative LuxR family transcriptional regulatorYqeH

//

>NGHB#2767 333

CMPLX not

b2846 yqeH putative LuxR family transcriptional regulatorYqeH

b2847 yqeI putative transcriptional regulator YqeI

//

>NGHB#2768 -8

CMPLX not

b2847 yqeI putative transcriptional regulator YqeI

b2848 yqeJ protein YqeJ

//

>NGHB#2770 35

CMPLX ---

b2849 yqeK protein YqeK

b4683 yqeL uncharacterized protein YqeL

//

>NGHB#2772 334

CMPLX not

b2851 ygeG TPR repeat-containing putative chaperone YgeG

b2852 ygeH putative transcriptional regulator YgeH

//

>NGHB#2773 167

CMPLX not

b2852 ygeH putative transcriptional regulator YgeH

b2853 ygeI protein YgeI

//

>NGHB#2775 -43

CMPLX tnp

b2861 insC4 IS2 insertion element repressor InsA

b2860 insD4 IS2 insertion element protein InsB

//

>NGHB#2776 433

CMPLX not

b2863 ygeQ putative lipoprotein YgeQ

b2861 insC4 IS2 insertion element repressor InsA

//

>NGHB#2777 307

CMPLX not

b2865 ygeR LysM domain-containing putative peptidaselipoprotein YgeR

b2863 ygeQ putative lipoprotein YgeQ

//

>NGHB#2779 10

CMPLX yes

b2866 xdhA putative xanthine dehydrogenasemolybdenum-binding subunit XdhA

b2867 xdhB putative xanthine dehydrogenase FAD-bindingsubunit XdhB

//

>NGHB#2780 -4

CMPLX yes

b2867 xdhB putative xanthine dehydrogenase FAD-bindingsubunit XdhB

b2868 xdhC putative xanthine dehydrogenaseiron-sulfur-binding subunit XdhC

//

>NGHB#2783 57

CMPLX not

b2870 ygeW putative carbamoyltransferase YgeW

b2871 ygeX 2,3-diaminopropionate ammonia-lyase

//

>NGHB#2784 57

CMPLX not

b2871 ygeX 2,3-diaminopropionate ammonia-lyase

b2872 ygeY putative peptidase YgeY

//

>NGHB#2785 52

CMPLX not

b2872 ygeY putative peptidase YgeY

b2873 hyuA phenylhydantoinase

//

>NGHB#2786 47

CMPLX not

b2873 hyuA phenylhydantoinase

b2874 yqeA putative amino acid kinase YqeA

//

>NGHB#2788 47

CMPLX ---

b2876 yqeC uncharacterized protein YqeC

b2875 yqeB XdhC-CoxI family protein YqeB

//

>NGHB#2790 321

CMPLX not

b2877 mocA molybdenum cofactor cytidylyltransferase

b2878 ygfK putative oxidoreductase, Fe-S subunit

//

>NGHB#2791 2

CMPLX not

b2878 ygfK putative oxidoreductase, Fe-S subunit

b2879 ssnA putative aminohydrolase SsnA

//

>NGHB#2792 50

CMPLX not

b2879 ssnA putative aminohydrolase SsnA

b2880 ygfM putative oxidoreductase

//

>NGHB#2793 -4

CMPLX not

b2880 ygfM putative oxidoreductase

b2881 xdhD fused putative xanthine/hypoxanthine oxidase:molybdopterin-binding subunit and Fe-S binding subunit

//

>NGHB#2794 164

CMPLX not

b2881 xdhD fused putative xanthine/hypoxanthine oxidase:molybdopterin-binding subunit and Fe-S binding subunit

b2882 xanQ xanthine:H(+) symporter XanQ

//

>NGHB#2795 14

CMPLX not

b2882 xanQ xanthine:H(+) symporter XanQ

b2883 guaD guanine deaminase

//

>NGHB#2796 35

CMPLX not

b2883 guaD guanine deaminase

b4464 ghxQ guanine/hypoxanthine transporter GhxQ

//

>NGHB#2798 -1

CMPLX not

b2887 ygfT putative oxidoreductase YgfT

b2886 ygfS putative electron transport protein YgfS

//

>NGHB#2800 1

CMPLX ---

b2888 uacT urate:H(+) symporter

b4684 yqfG uncharacterized protein YqfG

//

>NGHB#2804 9

CMPLX not

b2891 prfB peptide chain release factor RF2

b2890 lysS lysine--tRNA ligase, constitutive

//

>NGHB#2805 90

CMPLX not

b2892 recJ ssDNA-specific exonuclease RecJ

b2891 prfB peptide chain release factor RF2

//

>NGHB#2806 5

CMPLX not

b2893 dsbC protein disulfide isomerase DsbC

b2892 recJ ssDNA-specific exonuclease RecJ

//

>NGHB#2807 24

CMPLX not

b2894 xerD site-specific recombinase

b2893 dsbC protein disulfide isomerase DsbC

//

>NGHB#2810 -20

CMPLX not

b2897 sdhE FAD assembly factor

b2896 ygfX protein YgfX

//

>NGHB#2813 163

CMPLX not

b2900 yqfB N(4)-acetylcytidine amidohydrolase

b2899 yqfA hemolysin-III family protein YqfA

//

>NGHB#2816 266

CMPLX not

b2903 gcvP glycine decarboxylase

b2902 ygfF putative oxidoreductase YgfF

//

>NGHB#2817 118

CMPLX yes

b2904 gcvH glycine cleavage system H protein

b2903 gcvP glycine decarboxylase

//

>NGHB#2818 23

CMPLX yes

b2905 gcvT aminomethyltransferase

b2904 gcvH glycine cleavage system H protein

//

>NGHB#2821 22

CMPLX not

b2907 ubiH 2-octaprenyl-6-methoxyphenol 4-hydroxylase

b2906 ubiI 2-octaprenylphenol 6-hydroxylase

//

>NGHB#2822 -4

CMPLX not

b2908 pepP Xaa-Pro aminopeptidase

b2907 ubiH 2-octaprenyl-6-methoxyphenol 4-hydroxylase

//

>NGHB#2823 25

CMPLX not

b2909 ygfB UPF0149 family protein YgfB

b2908 pepP Xaa-Pro aminopeptidase

//

>NGHB#2825 299

CMPLX not

b2910 zapA cell division protein ZapA

b2912 fau putative 5-formyltetrahydrofolate cyclo-ligase

//

>NGHB#2827 228

CMPLX not

b2913 serA phosphoglycerate dehydrogenase

b4665 ibsC toxic peptide IbsC

//

>NGHB#2828 255

CMPLX not

b2914 rpiA ribose-5-phosphate isomerase A

b2913 serA phosphoglycerate dehydrogenase

//

>NGHB#2830 203

CMPLX not

b2916 argP DNA-binding transcriptional dual regulator ArgP

b2917 scpA methylmalonyl-CoA mutase

//

>NGHB#2831 -8

CMPLX not

b2917 scpA methylmalonyl-CoA mutase

b2918 argK methylmalonyl-CoA mutase-interacting GTPaseYgfD

//

>NGHB#2832 10

CMPLX not

b2918 argK methylmalonyl-CoA mutase-interacting GTPaseYgfD

b2919 scpB methylmalonyl-CoA decarboxylase

//

>NGHB#2833 23

CMPLX not

b2919 scpB methylmalonyl-CoA decarboxylase

b2920 scpC propionyl-CoA:succinate CoA transferase

//

>NGHB#2835 166

CMPLX not

b2922 yggE DUF541 domain-containing protein YggE

b2921 ygfI putative LysR-type DNA-binding transcriptionalregulator YgfI

//

>NGHB#2836 92

CMPLX not

b2923 argO L-arginine exporter

b2922 yggE DUF541 domain-containing protein YggE

//

>NGHB#2837 138

CMPLX not

b2924 mscS small conductance mechanosensitive channel MscS

b2923 argO L-arginine exporter

//

>NGHB#2838 357

CMPLX not

b2925 fbaA fructose-bisphosphate aldolase class II

b2924 mscS small conductance mechanosensitive channel MscS

//

>NGHB#2839 214

CMPLX not

b2926 pgk phosphoglycerate kinase

b2925 fbaA fructose-bisphosphate aldolase class II

//

>NGHB#2840 49

CMPLX not

b2927 epd D-erythrose-4-phosphate dehydrogenase

b2926 pgk phosphoglycerate kinase

//

>NGHB#2841 284

CMPLX not

b2928 yggC P-loop NTPase domain-containing protein YggC

b2927 epd D-erythrose-4-phosphate dehydrogenase

//

>NGHB#2842 -4

CMPLX not

b2929 fumE fumarase E

b2928 yggC P-loop NTPase domain-containing protein YggC

//

>NGHB#2843 21

CMPLX not

b2930 yggF fructose 1,6-bisphosphatase YggF

b2929 fumE fumarase E

//

>NGHB#2844 -4

CMPLX not

b4465 yggP putative zinc-binding dehydrogenase YggP

b2930 yggF fructose 1,6-bisphosphatase YggF

//

>NGHB#2845 14

CMPLX not

b2933 cmtA mannitol-specific PTS enzyme IICB componentCmtA

b4465 yggP putative zinc-binding dehydrogenase YggP

//

>NGHB#2846 27

CMPLX yes

b2934 cmtB mannitol-specific PTS enzyme IIA component CmtB

b2933 cmtA mannitol-specific PTS enzyme IICB componentCmtA

//

>NGHB#2847 313

CMPLX not

b2935 tktA transketolase 1

b2934 cmtB mannitol-specific PTS enzyme IIA component CmtB

//

>NGHB#2850 137

CMPLX not

b2938 speA arginine decarboxylase, biosynthetic

b2937 speB agmatinase

//

>NGHB#2851 8

CMPLX not

b2939 yqgB acid stress response protein YqgB

b2938 speA arginine decarboxylase, biosynthetic

//

>NGHB#2852 93

CMPLX not

b4784 yqgG protein YqgG

b2939 yqgB acid stress response protein YqgB

//

>NGHB#2856 423

CMPLX not

b2942 metK methionine adenosyltransferase

b2943 galP galactose:H(+) symporter

//

>NGHB#2857 76

CMPLX not

b2943 galP galactose:H(+) symporter

b2944 yggI protein YggI

//

>NGHB#2858 94

CMPLX not

b2944 yggI protein YggI

b2945 endA DNA-specific endonuclease I

//

>NGHB#2859 79

CMPLX not

b2945 endA DNA-specific endonuclease I

b2946 rsmE 16S rRNA m(3)U1498 methyltransferase

//

>NGHB#2860 12

CMPLX not

b2946 rsmE 16S rRNA m(3)U1498 methyltransferase

b2947 gshB glutathione synthetase

//

>NGHB#2861 108

CMPLX not

b2947 gshB glutathione synthetase

b2948 yqgE DUF179 domain-containing protein YqgE

//

>NGHB#2862 -1

CMPLX not

b2948 yqgE DUF179 domain-containing protein YqgE

b2949 yqgF ribonuclease H-like domain containing nuclease

//

>NGHB#2865 17

CMPLX ---

b2951 yggS PLP homeostasis protein

b2952 yggT uncharacterized protein YggT

//

>NGHB#2866 -4

CMPLX ---

b2952 yggT uncharacterized protein YggT

b2953 yggU DUF167 domain-containing protein YggU

//

>NGHB#2867 7

CMPLX not

b2953 yggU DUF167 domain-containing protein YggU

b2954 rdgB dITP/XTP pyrophosphatase

//

>NGHB#2868 -8

CMPLX not

b2954 rdgB dITP/XTP pyrophosphatase

b2955 hemW heme chaperone HemW

//

>NGHB#2870 116

CMPLX not

b2957 ansB L-asparaginase 2

b2956 yggM DUF1202 domain-containing protein YggM

//

>NGHB#2871 175

CMPLX not

b2958 yggN DUF2884 domain-containing protein YggN

b2957 ansB L-asparaginase 2

//

>NGHB#2872 183

CMPLX not

b2959 yggL protein YggL

b2958 yggN DUF2884 domain-containing protein YggN

//

>NGHB#2873 -1

CMPLX not

b2960 trmB tRNA m(7)G46 methyltransferase

b2959 yggL protein YggL

//

>NGHB#2875 27

CMPLX not

b2961 mutY adenine DNA glycosylase

b2962 yggX putative Fe(2(+))-trafficking protein

//

>NGHB#2876 64

CMPLX not

b2962 yggX putative Fe(2(+))-trafficking protein

b2963 mltC membrane-bound lytic murein transglycosylase C

//

>NGHB#2877 201

CMPLX not

b2963 mltC membrane-bound lytic murein transglycosylase C

b2964 nupG nucleoside:H(+) symporter NupG

//

>NGHB#2879 161

CMPLX not

b4785 yqgH protein YqgH

b2965 speC constitutive ornithine decarboxylase

//

>NGHB#2882 1

CMPLX yes

b2969 yghE putative type II secretion system L-type proteinYghE

b2968 yghD putative type II secretion system M-type proteinYghD

//

>NGHB#2885 17

CMPLX not

b2971 yghG lipoprotein YghG

b2970 yghF putative type II secretion system C-type proteinYghF

//

>NGHB#2886 65

CMPLX not

b2972 pppA prepilin peptidase

b2971 yghG lipoprotein YghG

//

>NGHB#2887 197

CMPLX not

b4466 yghJ putative lipoprotein YghJ

b2972 pppA prepilin peptidase

//

>NGHB#2888 484

CMPLX not

b2975 glcA glycolate/lactate:H(+) symporter GlcA

b4466 yghJ putative lipoprotein YghJ

//

>NGHB#2889 354

CMPLX not

b2976 glcB malate synthase G

b2975 glcA glycolate/lactate:H(+) symporter GlcA

//

>NGHB#2890 21

CMPLX not

b2977 glcG putative heme-binding protein GlcG

b2976 glcB malate synthase G

//

>NGHB#2891 4

CMPLX not

b4467 glcF glycolate dehydrogenase, putative iron-sulfursubunit

b2977 glcG putative heme-binding protein GlcG

//

>NGHB#2892 10

CMPLX yes

b4468 glcE glycolate dehydrogenase, putative FAD-bindingsubunit

b4467 glcF glycolate dehydrogenase, putative iron-sulfursubunit

//

>NGHB#2893 -1

CMPLX yes

b2979 glcD glycolate dehydrogenase, putative FAD-linkedsubunit

b4468 glcE glycolate dehydrogenase, putative FAD-bindingsubunit

//

>NGHB#2898 45

CMPLX not

b2984 yghR putative ATP-binding protein YghR

b2983 yghQ putative transport protein YghQ

//

>NGHB#2899 31

CMPLX not

b2985 yghS putative ATP-binding protein YghS

b2984 yghR putative ATP-binding protein YghR

//

>NGHB#2902 291

CMPLX not

b2988 gss fused glutathionylspermidineamidase/glutathionylspermidine synthetase

b2987 pitB metal phosphate:H(+) symporter PitB

//

>NGHB#2905 12

CMPLX yes

b2991 hybF hydrogenase maturation protein HybF

b2990 hybG hydrogenase maturation factor HybG

//

>NGHB#2906 -8

CMPLX not

b2992 hybE hydrogenase 2-specific chaperone

b2991 hybF hydrogenase maturation protein HybF

//

>NGHB#2907 -8

CMPLX not

b2993 hybD hydrogenase 2 maturation protease

b2992 hybE hydrogenase 2-specific chaperone

//

>NGHB#2908 -1

CMPLX not

b2994 hybC hydrogenase 2 large subunit

b2993 hybD hydrogenase 2 maturation protease

//

>NGHB#2909 -4

CMPLX yes

b2995 hybB hydrogenase 2 membrane subunit

b2994 hybC hydrogenase 2 large subunit

//

>NGHB#2910 -11

CMPLX yes

b2996 hybA hydrogenase 2 iron-sulfur protein

b2995 hybB hydrogenase 2 membrane subunit

//

>NGHB#2911 2

CMPLX yes

b2997 hybO hydrogenase 2 small subunit

b2996 hybA hydrogenase 2 iron-sulfur protein

//

>NGHB#2912 188

CMPLX not

b2998 yghW DUF2623 domain-containing protein YghW

b2997 hybO hydrogenase 2 small subunit

//

>NGHB#2913 859

CMPLX not

b4755 yqhI protein YqhI

b2998 yghW DUF2623 domain-containing protein YghW

//

>NGHB#2918 6

CMPLX yes

b3006 exbB Ton complex subunit ExbB

b3005 exbD Ton complex subunit ExbD

//

>NGHB#2920 139

CMPLX not

b3008 metC cystathionine beta-lyase/L-cysteinedesulfhydrase/alanine racemase

b3009 yghB DedA family protein YghB

//

>NGHB#2923 104

CMPLX not

b3011 yqhD NADPH-dependent aldehyde reductase YqhD

b3012 dkgA methylglyoxal reductase DkgA

//

>NGHB#2924 199

CMPLX not

b3012 dkgA methylglyoxal reductase DkgA

b3013 yqhG DUF3828 domain-containing protein YqhG

//

>NGHB#2925 50

CMPLX not

b3013 yqhG DUF3828 domain-containing protein YqhG

b3014 yqhH lipoprotein YqhH

//

>NGHB#2927 110

CMPLX not

b3017 ftsP cell division protein required during stressconditions

b4469 ygiQ radical SAM superfamily protein YgiQ

//

>NGHB#2928 74

CMPLX not

b3018 plsC 1-acylglycerol-3-phosphate O-acyltransferasePlsC

b3017 ftsP cell division protein required during stressconditions

//

>NGHB#2929 233

CMPLX not

b3019 parC DNA topoisomerase IV subunit A

b3018 plsC 1-acylglycerol-3-phosphate O-acyltransferasePlsC

//

>NGHB#2930 137

CMPLX not

b3020 ygiS putative deoxycholate binding periplasmicprotein

b3019 parC DNA topoisomerase IV subunit A

//

>NGHB#2931 132

CMPLX not

b3021 mqsA antitoxin of the MqsRA toxin-antitoxinsystem/DNA-binding transcriptional repressor MqsA

b3020 ygiS putative deoxycholate binding periplasmicprotein

//

>NGHB#2932 1

CMPLX yes

b3022 mqsR mRNA interferase toxin MqsR

b3021 mqsA antitoxin of the MqsRA toxin-antitoxinsystem/DNA-binding transcriptional repressor MqsA

//

>NGHB#2933 204

CMPLX not

b3023 ygiV DNA-binding transcriptional repressor YgiV

b3022 mqsR mRNA interferase toxin MqsR

//

>NGHB#2934 52

CMPLX not

b3024 ygiW BOF family protein YgiW

b3023 ygiV DNA-binding transcriptional repressor YgiV

//

>NGHB#2936 -4

CMPLX not

b3025 qseB DNA-binding transcriptional activator QseB

b3026 qseC sensor histidine kinase QseC

//

>NGHB#2939 30

CMPLX not

b3028 mdaB NADPH:quinone oxidoreductase MdaB

b3029 ygiN putative quinol monooxygenase YgiN

//

>NGHB#2941 28

CMPLX not

b3031 yqiA esterase YqiA

b3030 parE DNA topoisomerase IV subunit B

//

>NGHB#2942 -1

CMPLX not

b3032 cpdA cAMP phosphodiesterase

b3031 yqiA esterase YqiA

//

>NGHB#2943 24

CMPLX not

b3033 yqiB DUF1249 domain-containing protein YqiB

b3032 cpdA cAMP phosphodiesterase

//

>NGHB#2944 0

CMPLX not

b3034 nudF ADP-sugar pyrophosphatase

b3033 yqiB DUF1249 domain-containing protein YqiB

//

>NGHB#2946 147

CMPLX not

b3035 tolC outer membrane channel TolC

b3037 ygiB DUF1190 domain-containing protein YgiB

//

>NGHB#2947 5

CMPLX not

b3037 ygiB DUF1190 domain-containing protein YgiB

b3038 ygiC putative acid--amine ligase YgiC

//

>NGHB#2951 261

CMPLX not

b3041 ribB 3,4-dihydroxy-2-butanone-4-phosphate synthase

b4756 yqiD protein YqiD

//

>NGHB#2953 283

CMPLX not

b3042 ubiK ubiquinone biosynthesis accessory factor UbiK

b3043 ygiL putative fimbrial protein YgiL

//

>NGHB#2954 59

CMPLX not

b3043 ygiL putative fimbrial protein YgiL

b3046 yqiG putative outer membrane usher protein YqiG

//

>NGHB#2955 -43

CMPLX tnp

b3044 insC5 IS2 insertion element repressor InsA

b3045 insD5 IS2 insertion element protein InsB

//

>NGHB#2956 1

CMPLX not

b3047 yqiH putative fimbrial chaperone YqiH

b3048 yqiI putative fimbrial protein YqiI

//

>NGHB#2959 26

CMPLX not

b3050 yqiJ DUF1449 domain-containing inner membrane proteinYqiJ

b3051 yqiK flotillin family inner membrane protein YqiK

//

>NGHB#2960 240

CMPLX not

b3051 yqiK flotillin family inner membrane protein YqiK

b4664 ibsD putative toxic peptide IbsD

//

>NGHB#2961 315

CMPLX yes

b4664 ibsD putative toxic peptide IbsD

b4666 ibsE toxic peptide IbsE

//

>NGHB#2963 47

CMPLX not

b3053 glnE fused glutamine synthetase deadenylase/glutaminesynthetase adenylyltransferase

b3052 hldE fused heptose 7-phosphate kinase/heptose1-phosphate adenyltransferase

//

>NGHB#2964 22

CMPLX not

b3054 ygiF inorganic triphosphatase

b3053 glnE fused glutamine synthetase deadenylase/glutaminesynthetase adenylyltransferase

//

>NGHB#2966 63

CMPLX not

b3055 ygiM putative signal transduction protein (SH3domain)

b3056 cca fused CCA tRNAnucleotidyltransferase/phosphohydrolase

//

>NGHB#2968 89

CMPLX not

b3058 folB dihydroneopterin aldolase

b3057 bacA undecaprenyl pyrophosphate phosphatase

//

>NGHB#2972 -4

CMPLX yes

b3061 ttdA L(+)-tartrate dehydratase subunit alpha

b3062 ttdB L(+)-tartrate dehydratase subunit beta

//

>NGHB#2973 47

CMPLX not

b3062 ttdB L(+)-tartrate dehydratase subunit beta

b3063 ttdT L-tartrate:succinate antiporter

//

>NGHB#2976 110

CMPLX not

b3065 rpsU 30S ribosomal subunit protein S21

b3066 dnaG DNA primase

//

>NGHB#2977 194

CMPLX not

b3066 dnaG DNA primase

b3067 rpoD RNA polymerase, sigma 70 (sigma D) factor

//

>NGHB#2979 253

CMPLX not

b3070 nfeF NADPH-dependent ferric-chelate reductase

b3068 mug stationary phase mismatch/uracil DNAglycosylase

//

>NGHB#2985 183

CMPLX not

b3075 ebgR DNA-binding transcriptional repressor EbgR

b3076 ebgA evolved beta-D-galactosidase subunit alpha

//

>NGHB#2986 -4

CMPLX not

b3076 ebgA evolved beta-D-galactosidase subunit alpha

b3077 ebgC DUF386 domain-containing evolvedbeta-D-galactosidase subunit beta

//

>NGHB#2987 62

CMPLX not

b3077 ebgC DUF386 domain-containing evolvedbeta-D-galactosidase subunit beta

b3078 ygjI putative transporter YgjI

//

>NGHB#2988 133

CMPLX not

b3078 ygjI putative transporter YgjI

b3079 ygjJ protein YgjJ

//

>NGHB#2989 16

CMPLX not

b3079 ygjJ protein YgjJ

b3080 ygjK glycoside hydrolase

//

>NGHB#2990 425

CMPLX not

b3080 ygjK glycoside hydrolase

b3081 fadH 2,4-dienoyl-CoA reductase

//

>NGHB#2992 -4

CMPLX not

b3083 higB ribosome-dependent mRNA interferase toxin HigB

b3082 higA antitoxin/DNA-binding transcriptional repressorHigA

//

>NGHB#2993 283

CMPLX not

b3084 rlmG 23S rRNA m(2)G1835 methyltransferase

b3083 higB ribosome-dependent mRNA interferase toxin HigB

//

>NGHB#2995 76

CMPLX not

b3085 ygjP putative metal-dependent hydrolase

b3086 ygjQ DUF218 domain-containing protein YgjQ

//

>NGHB#2996 78

CMPLX not

b3086 ygjQ DUF218 domain-containing protein YgjQ

b3087 ygjR putative oxidoreductase YgjR

//

>NGHB#2997 282

CMPLX not

b3087 ygjR putative oxidoreductase YgjR

b3088 alx putative membrane-bound redox modulator Alx

//

>NGHB#2998 398

CMPLX not

b3088 alx putative membrane-bound redox modulator Alx

b3089 sstT serine/threonine:Na(+) symporter

//

>NGHB#3000 82

CMPLX not

b3091 uxaA D-altronate dehydratase

b3090 ygjV inner membrane protein

//

>NGHB#3001 14

CMPLX not

b3092 uxaC D-glucoronate/D-galacturonate isomerase

b3091 uxaA D-altronate dehydratase

//

>NGHB#3003 129

CMPLX not

b3093 exuT hexuronate transporter

b3094 exuR DNA-binding transcriptional repressor ExuR

//

>NGHB#3004 239

CMPLX not

b3094 exuR DNA-binding transcriptional repressor ExuR

b4787 yqiM protein YqiM

//

>NGHB#3005 21

CMPLX not

b4787 yqiM protein YqiM

b3095 yqjA DedA family protein YqjA

//

>NGHB#3006 3

CMPLX not

b3095 yqjA DedA family protein YqjA

b3096 mzrA modulator protein MzrA

//

>NGHB#3007 146

CMPLX not

b3096 mzrA modulator protein MzrA

b3097 yqjC DUF1090 domain-containing protein YqjC

//

>NGHB#3008 37

CMPLX not

b3097 yqjC DUF1090 domain-containing protein YqjC

b3098 yqjD ribosome- and membrane-associated DUF883domain-containing protein YqjD

//

>NGHB#3009 2

CMPLX not

b3098 yqjD ribosome- and membrane-associated DUF883domain-containing protein YqjD

b3099 yqjE inner membrane protein YqjE

//

>NGHB#3010 -11

CMPLX not

b3099 yqjE inner membrane protein YqjE

b3100 yqjK PF13997 family protein YqjK

//

>NGHB#3011 185

CMPLX not

b3100 yqjK PF13997 family protein YqjK

b3101 yqjF DoxX family protein

//

>NGHB#3012 69

CMPLX not

b3101 yqjF DoxX family protein

b3102 yqjG glutathionyl-hydroquinone reductase YqjG

//

>NGHB#3013 293

CMPLX not

b3102 yqjG glutathionyl-hydroquinone reductase YqjG

b3103 yhaH putative inner membrane protein

//

>NGHB#3014 241

CMPLX not

b3103 yhaH putative inner membrane protein

b3104 yhaI putative inner membrane protein

//

>NGHB#3017 22

CMPLX ---

b3106 yhaK bicupin-related protein

b3107 yhaL uncharacterized protein YhaL

//

>NGHB#3019 27

CMPLX not

b3110 cyuP cysteine detoxification protein CyuP

b4470 cyuA putative L-cysteine desulfidase CyuA

//

>NGHB#3020 274

CMPLX not

b4471 tdcG L-serine deaminase III

b3110 cyuP cysteine detoxification protein CyuP

//

>NGHB#3021 71

CMPLX not

b3113 tdcF putative enamine/imine deaminase

b4471 tdcG L-serine deaminase III

//

>NGHB#3022 13

CMPLX not

b3114 tdcE 2-ketobutyrate formate-lyase/pyruvateformate-lyase 4

b3113 tdcF putative enamine/imine deaminase

//

>NGHB#3023 33

CMPLX not

b3115 tdcD propionate kinase

b3114 tdcE 2-ketobutyrate formate-lyase/pyruvateformate-lyase 4

//

>NGHB#3024 25

CMPLX not

b3116 tdcC threonine/serine:H(+) symporter

b3115 tdcD propionate kinase

//

>NGHB#3025 21

CMPLX not

b3117 tdcB catabolic threonine dehydratase

b3116 tdcC threonine/serine:H(+) symporter

//

>NGHB#3026 98

CMPLX not

b3118 tdcA DNA-binding transcriptional activator TdcA

b3117 tdcB catabolic threonine dehydratase

//

>NGHB#3028 255

CMPLX not

b3119 tdcR DNA-binding transcriptional activator TdcR

b3120 yhaB protein YhaB

//

>NGHB#3029 21

CMPLX ---

b3120 yhaB protein YhaB

b3121 yhaC uncharacterized protein YhaC

//

>NGHB#3031 96

CMPLX not

b3125 garR tartronate semialdehyde reductase

b3124 garK glycerate 2-kinase 1

//

>NGHB#3032 35

CMPLX not

b3126 garL alpha-dehydro-beta-deoxy-D-glucarate aldolase

b3125 garR tartronate semialdehyde reductase

//

>NGHB#3033 15

CMPLX not

b3127 garP galactarate/D-glucarate transporter GarP

b3126 garL alpha-dehydro-beta-deoxy-D-glucarate aldolase

//

>NGHB#3035 148

CMPLX ---

b3128 garD hypothetical protein

b3129 prlF antitoxin PrlF

//

>NGHB#3036 -1

CMPLX yes

b3129 prlF antitoxin PrlF

b3130 yhaV ribosome-dependent mRNA interferase toxin YhaV

//

>NGHB#3039 22

CMPLX not

b3132 kbaZ tagatose-1,6-bisphosphate aldolase 1 subunitKbaZ

b3133 agaV N-acetyl-D-galactosamine specific PTS enzyme IIBcomponent

//

>NGHB#3040 1285

CMPLX not

b3133 agaV N-acetyl-D-galactosamine specific PTS enzyme IIBcomponent

b3136 agaS putative galactosamine-6-phosphatedeaminase/isomerase

//

>NGHB#3041 12

CMPLX not

b3136 agaS putative galactosamine-6-phosphatedeaminase/isomerase

b3137 kbaY tagatose-1,6-bisphosphate aldolase 1 subunitKbaY

//

>NGHB#3042 166

CMPLX not

b3137 kbaY tagatose-1,6-bisphosphate aldolase 1 subunitKbaY

b3138 agaB galactosamine-specific PTS enzyme IIB component

//

>NGHB#3043 38

CMPLX yes

b3138 agaB galactosamine-specific PTS enzyme IIB component

b3139 agaC galactosamine-specific PTS enzyme IIC component

//

>NGHB#3044 -11

CMPLX yes

b3139 agaC galactosamine-specific PTS enzyme IIC component

b3140 agaD galactosamine-specific PTS enzyme IID component

//

>NGHB#3045 0

CMPLX not

b3140 agaD galactosamine-specific PTS enzyme IID component

b3141 agaI putative deaminase AgaI

//

>NGHB#3046 400

CMPLX not

b3141 agaI putative deaminase AgaI

b3142 yraH putative fimbrial protein YraH

//

>NGHB#3047 79

CMPLX not

b3142 yraH putative fimbrial protein YraH

b3143 yraI putative fimbrial chaperone YraI

//

>NGHB#3048 28

CMPLX not

b3143 yraI putative fimbrial chaperone YraI

b3144 yraJ putative fimbrial usher protein YraJ

//

>NGHB#3049 10

CMPLX not

b3144 yraJ putative fimbrial usher protein YraJ

b3145 yraK putative fimbrial adhesin YraK

//

>NGHB#3052 -43

CMPLX not

b3147 lpoA outer membrane lipoprotein - activator of MrcAactivity

b3148 yraN UPF0102 family protein YraN

//

>NGHB#3053 19

CMPLX not

b3148 yraN UPF0102 family protein YraN

b3149 diaA DnaA initiator-associating factor forreplication initiation

//

>NGHB#3054 9

CMPLX not

b3149 diaA DnaA initiator-associating factor forreplication initiation

b3150 yraP divisome-associated lipoprotein YraP

//

>NGHB#3056 72

CMPLX not

b3152 yraR putative nucleoside-diphosphate-sugar epimerase

b3151 yraQ permease family protein YraQ

//

>NGHB#3061 -7

CMPLX not

b3157 ubiT anaerobic ubiquinone biosynthesis accessoryfactor UbiT

b3156 yhbS putative N-acetyltransferase YhbS

//

>NGHB#3063 8

CMPLX not

b3158 ubiU ubiquinone biosynthesis protein UbiU

b3159 ubiV ubiquinone biosynthesis protein UbiV

//

>NGHB#3064 80

CMPLX not

b3159 ubiV ubiquinone biosynthesis protein UbiV

b3160 yhbW putative luciferase-like monooxygenase YhbW

//

>NGHB#3066 153

CMPLX not

b3162 deaD ATP-dependent RNA helicase DeaD

b3161 mtr tryptophan:H(+) symporter Mtr

//

>NGHB#3067 -8

CMPLX ---

b4685 yrbN uncharacterized protein YrbN

b3162 deaD ATP-dependent RNA helicase DeaD

//

>NGHB#3068 106

CMPLX ---

b3163 nlpI lipoprotein NlpI

b4685 yrbN uncharacterized protein YrbN

//

>NGHB#3069 108

CMPLX not

b3164 pnp polynucleotide phosphorylase

b3163 nlpI lipoprotein NlpI

//

>NGHB#3070 246

CMPLX not

b3165 rpsO 30S ribosomal subunit protein S15

b3164 pnp polynucleotide phosphorylase

//

>NGHB#3071 148

CMPLX not

b3166 truB tRNA pseudouridine(55) synthase

b3165 rpsO 30S ribosomal subunit protein S15

//

>NGHB#3072 -1

CMPLX not

b3167 rbfA 30S ribosome binding factor

b3166 truB tRNA pseudouridine(55) synthase

//

>NGHB#3073 163

CMPLX not

b3168 infB translation initiation factor IF-2

b3167 rbfA 30S ribosome binding factor

//

>NGHB#3074 24

CMPLX not

b3169 nusA transcription termination/antiterminationprotein NusA

b3168 infB translation initiation factor IF-2

//

>NGHB#3075 27

CMPLX not

b3170 rimP ribosome maturation factor RimP

b3169 nusA transcription termination/antiterminationprotein NusA

//

>NGHB#3078 559

CMPLX not

b3175 secG Sec translocon subunit SecG

b3173 yhbX putative hydrolase YhbX

//

>NGHB#3079 227

CMPLX not

b3176 glmM phosphoglucosamine mutase

b3175 secG Sec translocon subunit SecG

//

>NGHB#3080 -8

CMPLX not

b3177 folP dihydropteroate synthase

b3176 glmM phosphoglucosamine mutase

//

>NGHB#3081 89

CMPLX not

b3178 ftsH ATP-dependent zinc metalloprotease FtsH

b3177 folP dihydropteroate synthase

//

>NGHB#3082 99

CMPLX not

b3179 rlmE 23S rRNA 2'-O-ribose U2552 methyltransferase

b3178 ftsH ATP-dependent zinc metalloprotease FtsH

//

>NGHB#3087 15

CMPLX not

b3184 yhbE putative transporter YhbE

b3183 obgE GTPase ObgE

//

>NGHB#3088 126

CMPLX not

b3185 rpmA 50S ribosomal subunit protein L27

b3184 yhbE putative transporter YhbE

//

>NGHB#3089 20

CMPLX yes

b3186 rplU 50S ribosomal subunit protein L21

b3185 rpmA 50S ribosomal subunit protein L27

//

>NGHB#3091 227

CMPLX not

b3187 ispB all-trans-octaprenyl-diphosphate synthase

b3188 sfsB putative transcriptional regulator SfsB

//

>NGHB#3093 54

CMPLX not

b3190 ibaG acid stress protein IbaG

b3189 murA UDP-N-acetylglucosamine1-carboxyvinyltransferase

//

>NGHB#3094 159

CMPLX not

b3191 mlaB intermembrane phospholipid transport systemprotein MlaB

b3190 ibaG acid stress protein IbaG

//

>NGHB#3095 -1

CMPLX yes

b3192 mlaC intermembrane phospholipid transport system -periplasmic binding protein

b3191 mlaB intermembrane phospholipid transport systemprotein MlaB

//

>NGHB#3096 18

CMPLX yes

b3193 mlaD intermembrane phospholipid transport system,substrate binding protein MlaD

b3192 mlaC intermembrane phospholipid transport system -periplasmic binding protein

//

>NGHB#3097 4

CMPLX yes

b3194 mlaE intermembrane phospholipid transport system,integral membrane subunit MlaE

b3193 mlaD intermembrane phospholipid transport system,substrate binding protein MlaD

//

>NGHB#3098 7

CMPLX yes

b3195 mlaF intermembrane phospholipid transport system, ATPbinding subunit MlaF

b3194 mlaE intermembrane phospholipid transport system,integral membrane subunit MlaE

//

>NGHB#3100 13

CMPLX not

b3196 yrbG putative transport protein YrbG

b3197 kdsD D-arabinose 5-phosphate isomerase KdsD

//

>NGHB#3101 20

CMPLX not

b3197 kdsD D-arabinose 5-phosphate isomerase KdsD

b3198 kdsC 3-deoxy-D-manno-octulosonate 8-phosphatephosphatase KdsC

//

>NGHB#3102 -4

CMPLX not

b3198 kdsC 3-deoxy-D-manno-octulosonate 8-phosphatephosphatase KdsC

b3199 lptC lipopolysaccharide transport system proteinLptC

//

>NGHB#3103 -32

CMPLX yes

b3199 lptC lipopolysaccharide transport system proteinLptC

b3200 lptA lipopolysaccharide transport system proteinLptA

//

>NGHB#3104 6

CMPLX yes

b3200 lptA lipopolysaccharide transport system proteinLptA

b3201 lptB lipopolysaccharide transport system ATP bindingprotein LptB

//

>NGHB#3105 47

CMPLX not

b3201 lptB lipopolysaccharide transport system ATP bindingprotein LptB

b3202 rpoN RNA polymerase, sigma 54 (sigma N) factor

//

>NGHB#3106 22

CMPLX not

b3202 rpoN RNA polymerase, sigma 54 (sigma N) factor

b3203 hpf ribosome hibernation-promoting factor

//

>NGHB#3107 117

CMPLX not

b3203 hpf ribosome hibernation-promoting factor

b3204 ptsN phosphotransferase system enzyme IIA(Ntr)

//

>NGHB#3108 45

CMPLX not

b3204 ptsN phosphotransferase system enzyme IIA(Ntr)

b3205 rapZ RNase adaptor protein RapZ

//

>NGHB#3109 -4

CMPLX not

b3205 rapZ RNase adaptor protein RapZ

b3206 npr phosphorelay protein NPr

//

>NGHB#3110 213

CMPLX not

b3206 npr phosphorelay protein NPr

b3207 yrbL protein kinase-like domain-containing proteinYrbL

//

>NGHB#3112 -4

CMPLX not

b3209 elbB low activity glyoxalase ElbB

b3208 mtgA peptidoglycan glycosyltransferase MtgA

//

>NGHB#3113 229

CMPLX not

b3210 arcB sensor histidine kinase ArcB

b3209 elbB low activity glyoxalase ElbB

//

>NGHB#3114 95

CMPLX not

b3211 yhcC radical SAM family oxidoreductase YhcC

b3210 arcB sensor histidine kinase ArcB

//

>NGHB#3116 12

CMPLX yes

b3212 gltB glutamate synthase subunit GltB

b3213 gltD glutamate synthase subunit GltD

//

>NGHB#3117 559

CMPLX not

b3213 gltD glutamate synthase subunit GltD

b3214 gltF periplasmic protein GltF

//

>NGHB#3118 171

CMPLX not

b3214 gltF periplasmic protein GltF

b3215 yhcA putative fimbrial chaperone YhcA

//

>NGHB#3119 20

CMPLX not

b3215 yhcA putative fimbrial chaperone YhcA

b3216 yhcD putative fimbrial usher protein YhcD

//

>NGHB#3120 -4

CMPLX ---

b3216 yhcD putative fimbrial usher protein YhcD

b4569 yhcE putative uncharacterized protein YhcE

//

>NGHB#3121 184

CMPLX not

b3219 yhcF DUF1120 domain-containing protein YhcF

b3220 yhcG DUF1016 domain-containing protein YhcG

//

>NGHB#3123 -4

CMPLX not

b3222 nanK N-acetylmannosamine kinase

b3221 yhcH DUF386 domain-containing protein YhcH

//

>NGHB#3124 -4

CMPLX not

b3223 nanE putative N-acetylmannosamine-6-phosphateepimerase

b3222 nanK N-acetylmannosamine kinase

//

>NGHB#3125 47

CMPLX not

b3224 nanT N-acetylneuraminate:H(+) symporter

b3223 nanE putative N-acetylmannosamine-6-phosphateepimerase

//

>NGHB#3126 108

CMPLX not

b3225 nanA N-acetylneuraminate lyase

b3224 nanT N-acetylneuraminate:H(+) symporter

//

>NGHB#3127 121

CMPLX not

b3226 nanR DNA-binding transcriptional dual regulator NanR

b3225 nanA N-acetylneuraminate lyase

//

>NGHB#3130 5

CMPLX not

b3229 sspA stringent starvation protein A

b3228 sspB ClpXP protease specificity-enhancing factor

//

>NGHB#3131 394

CMPLX not

b3230 rpsI 30S ribosomal subunit protein S9

b3229 sspA stringent starvation protein A

//

>NGHB#3132 15

CMPLX yes

b3231 rplM 50S ribosomal subunit protein L13

b3230 rpsI 30S ribosomal subunit protein S9

//

>NGHB#3133 218

CMPLX not

b3232 zapE cell division protein ZapE

b3231 rplM 50S ribosomal subunit protein L13

//

>NGHB#3135 153

CMPLX not

b3233 yhcB DUF1043 domain-containing inner membrane proteinYhcB

b3234 degQ periplasmic serine endoprotease

//

>NGHB#3136 89

CMPLX not

b3234 degQ periplasmic serine endoprotease

b3235 degS serine endoprotease

//

>NGHB#3139 364

CMPLX not

b3237 argR DNA-binding transcriptional dual regulator ArgR

b3238 yhcN DUF1471 domain-containing stress-induced proteinYhcN

//

>NGHB#3141 91

CMPLX not

b3240 aaeB aromatic carboxylic acid efflux pump subunitAaeB

b3239 yhcO putative barnase inhibitor

//

>NGHB#3142 5

CMPLX yes

b3241 aaeA aromatic carboxylic acid efflux pump membranefusion protein

b3240 aaeB aromatic carboxylic acid efflux pump subunitAaeB

//

>NGHB#3143 7

CMPLX not

b3242 aaeX DUF1656 domain-containing protein AaeX

b3241 aaeA aromatic carboxylic acid efflux pump membranefusion protein

//

>NGHB#3146 429

CMPLX not

b4472 yhdP outer membrane permeability factor YhdP

b3244 tldD metalloprotease subunit TldD

//

>NGHB#3147 67

CMPLX not

b3247 rng RNase G

b4472 yhdP outer membrane permeability factor YhdP

//

>NGHB#3148 -11

CMPLX not

b3248 yhdE nucleoside triphosphate pyrophosphatase YhdE

b3247 rng RNase G

//

>NGHB#3149 8

CMPLX not

b3249 mreD cell shape determining protein MreD

b3248 yhdE nucleoside triphosphate pyrophosphatase YhdE

//

>NGHB#3150 -1

CMPLX yes

b3250 mreC cell shape determining protein MreC

b3249 mreD cell shape determining protein MreD

//

>NGHB#3151 65

CMPLX yes

b3251 mreB dynamic cytoskeletal protein MreB

b3250 mreC cell shape determining protein MreC

//

>NGHB#3152 304

CMPLX not

b3252 csrD regulator of CsrB and CsrC decay

b3251 mreB dynamic cytoskeletal protein MreB

//

>NGHB#3154 977

CMPLX not

b3253 yhdH acrylyl-CoA reductase

b3255 accB biotin carboxyl carrier protein

//

>NGHB#3155 10

CMPLX not

b3255 accB biotin carboxyl carrier protein

b3256 accC biotin carboxylase

//

>NGHB#3156 108

CMPLX not

b3256 accC biotin carboxylase

b3257 yhdT DUF997 domain-containing protein YhdT

//

>NGHB#3157 -11

CMPLX not

b3257 yhdT DUF997 domain-containing protein YhdT

b3258 panF pantothenate:Na(+) symporter

//

>NGHB#3158 11

CMPLX not

b3258 panF pantothenate:Na(+) symporter

b3259 prmA ribosomal protein L11 methyltransferase

//

>NGHB#3159 328

CMPLX not

b3259 prmA ribosomal protein L11 methyltransferase

b3260 dusB tRNA-dihydrouridine synthase B

//

>NGHB#3160 25

CMPLX not

b3260 dusB tRNA-dihydrouridine synthase B

b3261 fis DNA-binding transcriptional dual regulator Fis

//

>NGHB#3161 85

CMPLX not

b3261 fis DNA-binding transcriptional dual regulator Fis

b3262 yhdJ DNA adenine methyltransferase

//

>NGHB#3162 83

CMPLX not

b3262 yhdJ DNA adenine methyltransferase

b3263 yhdU DUF2556 domain-containing protein YhdU

//

>NGHB#3165 11

CMPLX yes

b3265 acrE multidrug efflux pump membrane fusionlipoprotein AcrE

b3266 acrF multidrug efflux pump RND permease AcrF

//

>NGHB#3166 252

CMPLX not

b3266 acrF multidrug efflux pump RND permease AcrF

b3267 yhdV lipoprotein YhdV

//

>NGHB#3167 1522

CMPLX not

b3267 yhdV lipoprotein YhdV

b3269 yhdX putative ABC transporter membrane subunit YhdX

//

>NGHB#3168 9

CMPLX yes

b3269 yhdX putative ABC transporter membrane subunit YhdX

b3270 yhdY putative ABC transporter membrane subunit YhdY

//

>NGHB#3169 7

CMPLX yes

b3270 yhdY putative ABC transporter membrane subunit YhdY

b3271 yhdZ putative ABC transporter ATP-binding subunitYhdZ

//

>NGHB#3170 6041

CMPLX not

b3271 yhdZ putative ABC transporter ATP-binding subunitYhdZ

b3279 yrdA hexapeptide repeat-containing protein YrdA

//

>NGHB#3172 -4

CMPLX not

b3281 aroE shikimate dehydrogenase

b3280 yrdB DUF1488 domain-containing protein YrdB

//

>NGHB#3173 4

CMPLX not

b3282 tsaC threonylcarbamoyl-AMP synthase

b3281 aroE shikimate dehydrogenase

//

>NGHB#3174 4

CMPLX not

b3283 yrdD putative DNA topoisomerase

b3282 tsaC threonylcarbamoyl-AMP synthase

//

>NGHB#3175 28

CMPLX not

b3284 smg DUF494 domain-containing protein Smg

b3283 yrdD putative DNA topoisomerase

//

>NGHB#3176 -29

CMPLX not

b4473 smf protein Smf

b3284 smg DUF494 domain-containing protein Smg

//

>NGHB#3178 14

CMPLX not

b3287 def peptide deformylase

b3288 fmt 10-formyltetrahydrofolate:L-methionyl-tRNA(fMet)N-formyltransferase

//

>NGHB#3179 45

CMPLX not

b3288 fmt 10-formyltetrahydrofolate:L-methionyl-tRNA(fMet)N-formyltransferase

b3289 rsmB 16S rRNA m(5)C967 methyltransferase

//

>NGHB#3180 21

CMPLX not

b3289 rsmB 16S rRNA m(5)C967 methyltransferase

b3290 trkA NAD-binding component of Trk potassiumtransporters

//

>NGHB#3181 129

CMPLX not

b3290 trkA NAD-binding component of Trk potassiumtransporters

b3291 mscL large conductance mechanosensitive channel

//

>NGHB#3183 55

CMPLX not

b3292 zntR DNA-binding transcriptional activator ZntR

b4550 arfA alternative ribosome-rescue factor A

//

>NGHB#3184 10

CMPLX not

b3293 yhdN DUF1992 domain-containing protein YhdN

b3292 zntR DNA-binding transcriptional activator ZntR

//

>NGHB#3185 106

CMPLX not

b3294 rplQ 50S ribosomal subunit protein L17

b3293 yhdN DUF1992 domain-containing protein YhdN

//

>NGHB#3186 40

CMPLX not

b3295 rpoA RNA polymerase subunit alpha

b3294 rplQ 50S ribosomal subunit protein L17

//

>NGHB#3187 25

CMPLX not

b3296 rpsD 30S ribosomal subunit protein S4

b3295 rpoA RNA polymerase subunit alpha

//

>NGHB#3188 33

CMPLX yes

b3297 rpsK 30S ribosomal subunit protein S11

b3296 rpsD 30S ribosomal subunit protein S4

//

>NGHB#3189 16

CMPLX yes

b3298 rpsM 30S ribosomal subunit protein S13

b3297 rpsK 30S ribosomal subunit protein S11

//

>NGHB#3190 146

CMPLX yes

b3299 rpmJ 50S ribosomal subunit protein L36

b3298 rpsM 30S ribosomal subunit protein S13

//

>NGHB#3191 31

CMPLX not

b3300 secY Sec translocon subunit SecY

b3299 rpmJ 50S ribosomal subunit protein L36

//

>NGHB#3192 7

CMPLX not

b3301 rplO 50S ribosomal subunit protein L15

b3300 secY Sec translocon subunit SecY

//

>NGHB#3193 3

CMPLX yes

b3302 rpmD 50S ribosomal subunit protein L30

b3301 rplO 50S ribosomal subunit protein L15

//

>NGHB#3194 3

CMPLX yes

b3303 rpsE 30S ribosomal subunit protein S5

b3302 rpmD 50S ribosomal subunit protein L30

//

>NGHB#3195 14

CMPLX yes

b3304 rplR 50S ribosomal subunit protein L18

b3303 rpsE 30S ribosomal subunit protein S5

//

>NGHB#3196 9

CMPLX yes

b3305 rplF 50S ribosomal subunit protein L6

b3304 rplR 50S ribosomal subunit protein L18

//

>NGHB#3197 12

CMPLX yes

b3306 rpsH 30S ribosomal subunit protein S8

b3305 rplF 50S ribosomal subunit protein L6

//

>NGHB#3198 33

CMPLX yes

b3307 rpsN 30S ribosomal subunit protein S14

b3306 rpsH 30S ribosomal subunit protein S8

//

>NGHB#3199 14

CMPLX yes

b3308 rplE 50S ribosomal subunit protein L5

b3307 rpsN 30S ribosomal subunit protein S14

//

>NGHB#3200 14

CMPLX yes

b3309 rplX 50S ribosomal subunit protein L24

b3308 rplE 50S ribosomal subunit protein L5

//

>NGHB#3201 10

CMPLX yes

b3310 rplN 50S ribosomal subunit protein L14

b3309 rplX 50S ribosomal subunit protein L24

//

>NGHB#3202 164

CMPLX yes

b3311 rpsQ 30S ribosomal subunit protein S17

b3310 rplN 50S ribosomal subunit protein L14

//

>NGHB#3203 -1

CMPLX yes

b3312 rpmC 50S ribosomal subunit protein L29

b3311 rpsQ 30S ribosomal subunit protein S17

//

>NGHB#3204 -1

CMPLX yes

b3313 rplP 50S ribosomal subunit protein L16

b3312 rpmC 50S ribosomal subunit protein L29

//

>NGHB#3205 12

CMPLX yes

b3314 rpsC 30S ribosomal subunit protein S3

b3313 rplP 50S ribosomal subunit protein L16

//

>NGHB#3206 17

CMPLX yes

b3315 rplV 50S ribosomal subunit protein L22

b3314 rpsC 30S ribosomal subunit protein S3

//

>NGHB#3207 14

CMPLX yes

b3316 rpsS 30S ribosomal subunit protein S19

b3315 rplV 50S ribosomal subunit protein L22

//

>NGHB#3208 16

CMPLX yes

b3317 rplB 50S ribosomal subunit protein L2

b3316 rpsS 30S ribosomal subunit protein S19

//

>NGHB#3209 17

CMPLX yes

b3318 rplW 50S ribosomal subunit protein L23

b3317 rplB 50S ribosomal subunit protein L2

//

>NGHB#3210 -4

CMPLX yes

b3319 rplD 50S ribosomal subunit protein L4

b3318 rplW 50S ribosomal subunit protein L23

//

>NGHB#3211 10

CMPLX yes

b3320 rplC 50S ribosomal subunit protein L3

b3319 rplD 50S ribosomal subunit protein L4

//

>NGHB#3212 32

CMPLX yes

b3321 rpsJ 30S ribosomal subunit protein S10

b3320 rplC 50S ribosomal subunit protein L3

//

>NGHB#3213 237

CMPLX not

b3322 gspB putative general secretion pathway protein B

b3321 rpsJ 30S ribosomal subunit protein S10

//

>NGHB#3214 1

CMPLX yes

b3323 gspA Type II secretion system protein GspA

b3322 gspB putative general secretion pathway protein B

//

>NGHB#3216 -17

CMPLX yes

b3324 gspC Type II secretion system protein GspC

b3325 gspD Type II secretion system protein GspD

//

>NGHB#3217 9

CMPLX yes

b3325 gspD Type II secretion system protein GspD

b3326 gspE Type II secretion system protein GspE

//

>NGHB#3218 -4

CMPLX yes

b3326 gspE Type II secretion system protein GspE

b3327 gspF Type II secretion system protein GspF

//

>NGHB#3219 9

CMPLX yes

b3327 gspF Type II secretion system protein GspF

b3328 gspG Type II secretion system protein GspG

//

>NGHB#3220 7

CMPLX yes

b3328 gspG Type II secretion system protein GspG

b3329 gspH Type II secretion system protein GspH

//

>NGHB#3221 -4

CMPLX yes

b3329 gspH Type II secretion system protein GspH

b3330 gspI Type II secretion system protein GspI

//

>NGHB#3222 -8

CMPLX yes

b3330 gspI Type II secretion system protein GspI

b3331 gspJ Type II secretion system protein GspJ

//

>NGHB#3223 -8

CMPLX yes

b3331 gspJ Type II secretion system protein GspJ

b3332 gspK Type II secretion system protein GspK

//

>NGHB#3224 14

CMPLX yes

b3332 gspK Type II secretion system protein GspK

b3333 gspL Type II secretion system protein GspL

//

>NGHB#3225 -4

CMPLX yes

b3333 gspL Type II secretion system protein GspL

b3334 gspM Type II secretion system protein GspM

//

>NGHB#3226 -1

CMPLX not

b3334 gspM Type II secretion system protein GspM

b3335 gspO Type II secretion system prepilin peptidase

//

>NGHB#3228 71

CMPLX yes

b3337 bfd bacterioferritin-associated ferredoxin

b3336 bfr bacterioferritin

//

>NGHB#3229 168

CMPLX not

b3338 chiA endochitinase

b3337 bfd bacterioferritin-associated ferredoxin

//

>NGHB#3230 291

CMPLX not

b3339 tufA translation elongation factor Tu 1

b3338 chiA endochitinase

//

>NGHB#3231 70

CMPLX not

b3340 fusA elongation factor G

b3339 tufA translation elongation factor Tu 1

//

>NGHB#3232 27

CMPLX not

b3341 rpsG 30S ribosomal subunit protein S7

b3340 fusA elongation factor G

//

>NGHB#3233 96

CMPLX yes

b3342 rpsL 30S ribosomal subunit protein S12

b3341 rpsG 30S ribosomal subunit protein S7

//

>NGHB#3234 125

CMPLX not

b3343 tusB sulfurtransferase complex subunit TusB

b3342 rpsL 30S ribosomal subunit protein S12

//

>NGHB#3235 7

CMPLX yes

b3344 tusC sulfurtransferase complex subunit TusC

b3343 tusB sulfurtransferase complex subunit TusB

//

>NGHB#3236 -1

CMPLX yes

b3345 tusD sulfurtransferase complex subunit TusD

b3344 tusC sulfurtransferase complex subunit TusC

//

>NGHB#3237 -1

CMPLX not

b3346 yheO DNA-binding transcriptional regulator YheO

b3345 tusD sulfurtransferase complex subunit TusD

//

>NGHB#3238 166

CMPLX not

b3347 fkpA peptidyl-prolyl cis-trans isomerase FkpA

b3346 yheO DNA-binding transcriptional regulator YheO

//

>NGHB#3241 94

CMPLX not

b4551 yheV DUF2387 domain-containing protein YheV

b3349 slyD FKBP-type peptidyl-prolyl cis-trans isomeraseSlyD

//

>NGHB#3242 9

CMPLX not

b3350 kefB K(+) : H(+) antiporter KefB

b4551 yheV DUF2387 domain-containing protein YheV

//

>NGHB#3243 -1

CMPLX not

b3351 kefG glutathione-regulated potassium-efflux systemancillary protein KefG

b3350 kefB K(+) : H(+) antiporter KefB

//

>NGHB#3245 -1

CMPLX not

b3352 yheS putative ATP-binding protein YheS

b3353 yheT putative hydrolase YheT

//

>NGHB#3246 -7

CMPLX not

b3353 yheT putative hydrolase YheT

b3354 yheU UPF0270 family protein YheU

//

>NGHB#3247 53

CMPLX not

b3354 yheU UPF0270 family protein YheU

b3355 prkB putative phosphoribulokinase

//

>NGHB#3250 38

CMPLX not

b3357 crp DNA-binding transcriptional dual regulator CRP

b3358 yhfK putative transporter YhfK

//

>NGHB#3252 85

CMPLX not

b3360 pabA aminodeoxychorismate synthase subunit 2

b3359 argD N-acetylornithine aminotransferase/N-succinyldiaminopimelateaminotransferase

//

>NGHB#3253 31

CMPLX not

b3361 fic putative adenosine monophosphate--proteintransferase Fic

b3360 pabA aminodeoxychorismate synthase subunit 2

//

>NGHB#3254 -11

CMPLX not

b3362 yhfG DUF2559 domain-containing protein YhfG

b3361 fic putative adenosine monophosphate--proteintransferase Fic

//

>NGHB#3255 104

CMPLX not

b3363 ppiA peptidyl-prolyl cis-trans isomerase A

b3362 yhfG DUF2559 domain-containing protein YhfG

//

>NGHB#3257 261

CMPLX not

b3364 tsgA putative transporter TsgA

b3365 nirB nitrite reductase catalytic subunit NirB

//

>NGHB#3258 -4

CMPLX yes

b3365 nirB nitrite reductase catalytic subunit NirB

b3366 nirD nitrite reductase subunit NirD

//

>NGHB#3259 125

CMPLX not

b3366 nirD nitrite reductase subunit NirD

b3367 nirC nitrite transporter NirC

//

>NGHB#3260 18

CMPLX not

b3367 nirC nitrite transporter NirC

b3368 cysG siroheme synthase

//

>NGHB#3261 246

CMPLX not

b3368 cysG siroheme synthase

b3369 yhfL DUF4223 domain-containing lipoprotein YhfL

//

>NGHB#3262 294

CMPLX not

b3369 yhfL DUF4223 domain-containing lipoprotein YhfL

b3370 frlA fructoselysine/psicoselysine transporter

//

>NGHB#3263 20

CMPLX not

b3370 frlA fructoselysine/psicoselysine transporter

b3371 frlB fructoselysine 6-phosphate deglycase

//

>NGHB#3264 49

CMPLX not

b3371 frlB fructoselysine 6-phosphate deglycase

b4474 frlC fructoselysine 3-epimerase

//

>NGHB#3265 -4

CMPLX not

b4474 frlC fructoselysine 3-epimerase

b3374 frlD fructoselysine 6-kinase

//

>NGHB#3266 99

CMPLX not

b3374 frlD fructoselysine 6-kinase

b3375 frlR putative DNA-binding transcriptional regulatorFrlR

//

>NGHB#3268 11

CMPLX ---

b3377 yhfT uncharacterized protein YhfT

b3376 yhfS putative aminotransferase YhfS

//

>NGHB#3269 11

CMPLX ---

b3378 yhfU DUF2620 domain-containing protein YhfU

b3377 yhfT uncharacterized protein YhfT

//

>NGHB#3270 10

CMPLX not

b3379 php putative hydrolase

b3378 yhfU DUF2620 domain-containing protein YhfU

//

>NGHB#3271 -4

CMPLX not

b3380 yhfW putative mutase YhfW

b3379 php putative hydrolase

//

>NGHB#3272 -1

CMPLX not

b3381 yhfX putative PLP-binding protein YhfX

b3380 yhfW putative mutase YhfW

//

>NGHB#3273 83

CMPLX not

b3382 yhfY PRD domain-containing protein YhfY

b3381 yhfX putative PLP-binding protein YhfX

//

>NGHB#3274 16

CMPLX not

b3383 yhfZ putative DNA-binding transcriptional regulatorYhfZ

b3382 yhfY PRD domain-containing protein YhfY

//

>NGHB#3275 289

CMPLX not

b3384 trpS tryptophan--tRNA ligase

b3383 yhfZ putative DNA-binding transcriptional regulatorYhfZ

//

>NGHB#3276 -8

CMPLX not

b3385 gph phosphoglycolate phosphatase

b3384 trpS tryptophan--tRNA ligase

//

>NGHB#3277 -8

CMPLX not

b3386 rpe ribulose-phosphate 3-epimerase

b3385 gph phosphoglycolate phosphatase

//

>NGHB#3278 17

CMPLX not

b3387 dam DNA adenine methyltransferase

b3386 rpe ribulose-phosphate 3-epimerase

//

>NGHB#3279 106

CMPLX not

b3388 damX cell division protein DamX

b3387 dam DNA adenine methyltransferase

//

>NGHB#3280 91

CMPLX not

b3389 aroB 3-dehydroquinate synthase

b3388 damX cell division protein DamX

//

>NGHB#3281 56

CMPLX not

b3390 aroK shikimate kinase 1

b3389 aroB 3-dehydroquinate synthase

//

>NGHB#3282 400

CMPLX not

b3391 hofQ DNA utilization protein HofQ

b3390 aroK shikimate kinase 1

//

>NGHB#3283 -89

CMPLX yes

b3392 hofP DNA utilization protein HofP

b3391 hofQ DNA utilization protein HofQ

//

>NGHB#3284 -11

CMPLX yes

b3393 hofO DNA utilization protein HofO

b3392 hofP DNA utilization protein HofP

//

>NGHB#3285 -17

CMPLX yes

b3394 hofN DNA utilization protein HofN

b3393 hofO DNA utilization protein HofO

//

>NGHB#3286 -1

CMPLX yes

b3395 hofM DNA utilization protein HofM

b3394 hofN DNA utilization protein HofN

//

>NGHB#3290 64

CMPLX not

b3398 igaA inner membrane protein IgaA

b3399 yrfG purine nucleotidase

//

>NGHB#3291 10

CMPLX not

b3399 yrfG purine nucleotidase

b3400 hslR heat shock protein Hsp15

//

>NGHB#3292 24

CMPLX not

b3400 hslR heat shock protein Hsp15

b3401 hslO molecular chaperone Hsp33

//

>NGHB#3296 -4

CMPLX not

b3405 ompR DNA-binding transcriptional dual regulator OmpR

b3404 envZ sensor histidine kinase EnvZ

//

>NGHB#3298 96

CMPLX not

b3406 greB transcription elongation factor GreB

b3407 yhgF putative RNA-binding protein YhgF

//

>NGHB#3299 456

CMPLX not

b3407 yhgF putative RNA-binding protein YhgF

b3408 feoA ferrous iron transport protein A

//

>NGHB#3300 16

CMPLX yes

b3408 feoA ferrous iron transport protein A

b3409 feoB Fe(2(+)) transporter FeoB

//

>NGHB#3301 -1

CMPLX yes

b3409 feoB Fe(2(+)) transporter FeoB

b3410 feoC ferrous iron transport protein FeoC

//

>NGHB#3302 202

CMPLX not

b3410 feoC ferrous iron transport protein FeoC

b3411 rpnA recombination-promoting nuclease RpnA

//

>NGHB#3305 58

CMPLX not

b3413 yhgH DNA utilization protein YhgH

b3414 nfuA iron-sulfur cluster carrier protein NfuA

//

>NGHB#3306 359

CMPLX not

b3414 nfuA iron-sulfur cluster carrier protein NfuA

b3415 gntT high-affinity gluconate transporter

//

>NGHB#3308 9

CMPLX not

b3417 malP maltodextrin phosphorylase

b3416 malQ 4-alpha-glucanotransferase

//

>NGHB#3311 3

CMPLX not

b3421 rtcB RNA-splicing ligase

b4475 rtcA RNA 3'-terminal phosphate cyclase

//

>NGHB#3314 44

CMPLX not

b3425 glpE thiosulfate sulfurtransferase GlpE

b3424 glpG rhomboid protease GlpG

//

>NGHB#3317 128

CMPLX ---

b3428 glgP glycogen phosphorylase

b3427 yzgL putative uncharacterized protein YzgL

//

>NGHB#3318 18

CMPLX not

b3429 glgA glycogen synthase

b3428 glgP glycogen phosphorylase

//

>NGHB#3319 -1

CMPLX not

b3430 glgC glucose-1-phosphate adenylyltransferase

b3429 glgA glycogen synthase

//

>NGHB#3320 17

CMPLX not

b3431 glgX limit dextrin alpha-1,6-glucohydrolase

b3430 glgC glucose-1-phosphate adenylyltransferase

//

>NGHB#3321 -4

CMPLX not

b3432 glgB 1,4-alpha-glucan branching enzyme

b3431 glgX limit dextrin alpha-1,6-glucohydrolase

//

>NGHB#3322 157

CMPLX not

b4788 yhgO protein YhgO

b3432 glgB 1,4-alpha-glucan branching enzyme

//

>NGHB#3323 -23

CMPLX not

b4789 yhgP protein YhgP

b4788 yhgO protein YhgO

//

>NGHB#3324 66

CMPLX not

b3433 asd aspartate-semialdehyde dehydrogenase

b4789 yhgP protein YhgP

//

>NGHB#3327 3

CMPLX not

b3437 gntK D-gluconate kinase, thermostable

b4476 gntU low-affinity gluconate transporter

//

>NGHB#3328 138

CMPLX not

b3438 gntR DNA-binding transcriptional repressor GntR

b3437 gntK D-gluconate kinase, thermostable

//

>NGHB#3329 223

CMPLX not

b3439 yhhW quercetin 2,3-dioxygenase

b3438 gntR DNA-binding transcriptional repressor GntR

//

>NGHB#3330 122

CMPLX not

b3440 yhhX putative oxidoreductase YhhX

b3439 yhhW quercetin 2,3-dioxygenase

//

>NGHB#3332 236

CMPLX not

b3441 yhhY N-acetyltransferase YhhY

b3442 yhhZ putative endonuclease YhhZ

//

>NGHB#3333 -4

CMPLX ---

b3442 yhhZ putative endonuclease YhhZ

b3443 yrhA putative uncharacterized protein YrhA

//

>NGHB#3334 -82

CMPLX tnp

b3444 insA6 IS1 protein InsA

b3445 insB6 IS1 protein InsB

//

>NGHB#3335 199

CMPLX ---

b4612 yrhD uncharacterized protein YrhD

b3446 yrhB putative heat shock chaperone

//

>NGHB#3339 -4

CMPLX not

b3450 ugpC sn-glycerol 3-phosphate ABC transporter ATPbinding subunit

b3449 ugpQ glycerophosphodiester phosphodiesterase UgpQ

//

>NGHB#3340 1

CMPLX yes

b3451 ugpE sn-glycerol 3-phosphate ABC transporter membranesubunit UgpE

b3450 ugpC sn-glycerol 3-phosphate ABC transporter ATPbinding subunit

//

>NGHB#3341 -4

CMPLX yes

b3452 ugpA sn-glycerol 3-phosphate ABC transporter membranesubunit UgpA

b3451 ugpE sn-glycerol 3-phosphate ABC transporter membranesubunit UgpE

//

>NGHB#3342 97

CMPLX yes

b3453 ugpB sn-glycerol 3-phosphate ABC transporterperiplasmic binding protein

b3452 ugpA sn-glycerol 3-phosphate ABC transporter membranesubunit UgpA

//

>NGHB#3343 398

CMPLX not

b3454 livF branched chain amino acid/phenylalanine ABCtransporter ATP binding subunit LivF

b3453 ugpB sn-glycerol 3-phosphate ABC transporterperiplasmic binding protein

//

>NGHB#3344 1

CMPLX yes

b3455 livG branched chain amino acid/phenylalanine ABCtransporter ATP binding subunit LivG

b3454 livF branched chain amino acid/phenylalanine ABCtransporter ATP binding subunit LivF

//

>NGHB#3345 -4

CMPLX yes

b3456 livM branched chain amino acid/phenylalanine ABCtransporter membrane subunit LivM

b3455 livG branched chain amino acid/phenylalanine ABCtransporter ATP binding subunit LivG

//

>NGHB#3346 -4

CMPLX yes

b3457 livH branched chain amino acid/phenylalanine ABCtransporter membrane subunit LivH

b3456 livM branched chain amino acid/phenylalanine ABCtransporter membrane subunit LivM

//

>NGHB#3347 47

CMPLX yes

b3458 livK L-leucine/L-phenylalanine ABC transporterperiplasmic binding protein

b3457 livH branched chain amino acid/phenylalanine ABCtransporter membrane subunit LivH

//

>NGHB#3350 270

CMPLX not

b3461 rpoH RNA polymerase, sigma 32 (sigma H) factor

b3460 livJ branched chain amino acid/phenylalanine ABCtransporter periplasmic binding protein

//

>NGHB#3351 244

CMPLX not

b3462 ftsX cell division protein FtsX

b3461 rpoH RNA polymerase, sigma 32 (sigma H) factor

//

>NGHB#3352 -8

CMPLX not

b3463 ftsE cell division protein FtsE

b3462 ftsX cell division protein FtsX

//

>NGHB#3353 2

CMPLX not

b3464 ftsY signal recognition particle receptor

b3463 ftsE cell division protein FtsE

//

>NGHB#3355 -11

CMPLX not

b3465 rsmD 16S rRNA m(2)G966 methyltransferase

b3466 yhhL DUF1145 domain-containing protein YhhL

//

>NGHB#3358 73

CMPLX not

b3468 yhhN PF07947 family protein YhhN

b3469 zntA Zn(2(+))/Cd(2(+))/Pb(2(+)) exporting P-typeATPase

//

>NGHB#3361 72

CMPLX not

b3471 yhhQ queuosine precursor transporter

b3472 dcrB periplasmic bacteriophage sensitivity proteinDcrB

//

>NGHB#3364 54

CMPLX not

b3474 yhhT putative transporter YhhT

b3475 acpT holo-[acyl carrier protein] synthase 2

//

>NGHB#3365 110

CMPLX not

b3475 acpT holo-[acyl carrier protein] synthase 2

b3476 nikA Ni(2(+)) ABC transporter periplasmic bindingprotein

//

>NGHB#3366 -1

CMPLX yes

b3476 nikA Ni(2(+)) ABC transporter periplasmic bindingprotein

b3477 nikB Ni(2(+)) ABC transporter membrane subunit NikB

//

>NGHB#3367 -4

CMPLX yes

b3477 nikB Ni(2(+)) ABC transporter membrane subunit NikB

b3478 nikC Ni(2(+)) ABC transporter membrane subunit NikC

//

>NGHB#3368 -1

CMPLX yes

b3478 nikC Ni(2(+)) ABC transporter membrane subunit NikC

b3479 nikD Ni(2(+)) ABC transporter ATP binding subunitNikD

//

>NGHB#3369 -4

CMPLX yes

b3479 nikD Ni(2(+)) ABC transporter ATP binding subunitNikD

b3480 nikE Ni(2(+)) ABC transporter ATP binding subunitNikE

//

>NGHB#3370 5

CMPLX not

b3480 nikE Ni(2(+)) ABC transporter ATP binding subunitNikE

b3481 nikR DNA-binding transcriptional repressor NikR

//

>NGHB#3371 202

CMPLX not

b3481 nikR DNA-binding transcriptional repressor NikR

b3482 rhsB rhs element protein RhsB

//

>NGHB#3372 -29

CMPLX not

b3482 rhsB rhs element protein RhsB

b3483 yhhH PF15631 family protein YhhH

//

>NGHB#3373 595

CMPLX not

b3483 yhhH PF15631 family protein YhhH

b3484 yhhI putative transposase

//

>NGHB#3375 -1

CMPLX not

b3486 rbbA ribosome-associated ATPase

b3485 yhhJ ABC transporter family protein YhhJ

//

>NGHB#3376 -4

CMPLX not

b3487 yhiI putative membrane fusion protein YhiI

b3486 rbbA ribosome-associated ATPase

//

>NGHB#3377 62

CMPLX not

b4790 yhiY protein YhiY

b3487 yhiI putative membrane fusion protein YhiI

//

>NGHB#3378 189

CMPLX not

b3488 yhiJ DUF4049 domain-containing protein YhiJ

b4790 yhiY protein YhiY

//

>NGHB#3385 -44

CMPLX not

b4792 yriB protein YriB

b4791 yriA protein YriA

//

>NGHB#3388 7

CMPLX not

b3498 prlC oligopeptidase A

b3497 rsmJ 16S rRNA m(2)G1516 methyltransferase

//

>NGHB#3390 71

CMPLX not

b3499 rlmJ 23S rRNA m(6)A2030 methyltransferase

b3500 gor glutathione reductase (NADPH)

//

>NGHB#3393 53

CMPLX not

b3501 arsR DNA-binding transcriptional repressor ArsR

b3502 arsB arsenite/antimonite:H(+) antiporter

//

>NGHB#3394 12

CMPLX not

b3502 arsB arsenite/antimonite:H(+) antiporter

b3503 arsC arsenate reductase

//

>NGHB#3395 628

CMPLX ---

b3503 arsC arsenate reductase

b3504 yhiS putative uncharacterized protein YhiS

//

>NGHB#3396 155

CMPLX not

b3506 slp starvation lipoprotein

b3507 dctR putative DNA-binding transcriptional regulatorDctR

//

>NGHB#3398 63

CMPLX not

b3509 hdeB periplasmic acid stress chaperone HdeB

b3508 yhiD inner membrane protein YhiD

//

>NGHB#3399 115

CMPLX yes

b3510 hdeA periplasmic acid stress chaperone HdeA

b3509 hdeB periplasmic acid stress chaperone HdeB

//

>NGHB#3401 798

CMPLX not

b3511 hdeD acid-resistance membrane protein

b3512 gadE DNA-binding transcriptional activator GadE

//

>NGHB#3402 338

CMPLX not

b3512 gadE DNA-binding transcriptional activator GadE

b3513 mdtE multidrug efflux pump membrane fusion proteinMdtE

//

>NGHB#3403 24

CMPLX yes

b3513 mdtE multidrug efflux pump membrane fusion proteinMdtE

b3514 mdtF multidrug efflux pump RND permease MdtF

//

>NGHB#3405 367

CMPLX not

b3516 gadX DNA-binding transcriptional dual regulator GadX

b3515 gadW DNA-binding transcriptional dual regulator GadW

//

>NGHB#3406 369

CMPLX not

b3517 gadA glutamate decarboxylase A

b3516 gadX DNA-binding transcriptional dual regulator GadX

//

>NGHB#3407 210

CMPLX not

b3518 ccp cytochrome c peroxidase

b3517 gadA glutamate decarboxylase A

//

>NGHB#3411 48

CMPLX not

b3521 rcdB putative DNA-binding transcriptional regulatorYhjC

b3522 yhjD putative transporter YhjD

//

>NGHB#3412 410

CMPLX yes

b3522 yhjD putative transporter YhjD

b3523 yhjE putative transporter YhjE

//

>NGHB#3414 69

CMPLX not

b3525 pdeH c-di-GMP phosphodiesterase PdeH

b3524 yhjG AsmA family protein YhjG

//

>NGHB#3417 220

CMPLX not

b3528 dctA C4 dicarboxylate/orotate:H(+) symporter

b3527 yhjJ peptidase M16 family protein YhjJ

//

>NGHB#3418 182

CMPLX not

b3529 pdeK c-di-GMP phosphodiesterase PdeK

b3528 dctA C4 dicarboxylate/orotate:H(+) symporter

//

>NGHB#3419 81

CMPLX not

b3530 bcsC cellulose synthase outer membrane channel

b3529 pdeK c-di-GMP phosphodiesterase PdeK

//

>NGHB#3420 -19

CMPLX not

b3531 bcsZ endo-1,4-D-glucanase

b3530 bcsC cellulose synthase outer membrane channel

//

>NGHB#3421 6

CMPLX not

b3532 bcsB cellulose synthase periplasmic subunit

b3531 bcsZ endo-1,4-D-glucanase

//

>NGHB#3422 10

CMPLX yes

b3533 bcsA cellulose synthase catalytic subunit

b3532 bcsB cellulose synthase periplasmic subunit

//

>NGHB#3423 760

CMPLX not

b3535 yhjR PF10945 family protein YhjR

b3533 bcsA cellulose synthase catalytic subunit

//

>NGHB#3425 -4

CMPLX not

b3536 bcsE c-di-GMP-binding protein BcsE

b3537 bcsF putative cellulose biosynthesis protein BcsF

//

>NGHB#3426 -4

CMPLX not

b3537 bcsF putative cellulose biosynthesis protein BcsF

b3538 bcsG cellulose phosphoethanolamine transferase

//

>NGHB#3430 -4

CMPLX yes

b3541 dppD dipeptide ABC transporter ATP binding subunitDppD

b3540 dppF dipeptide ABC transporter ATP binding subunitDppF

//

>NGHB#3431 10

CMPLX yes

b3542 dppC dipeptide ABC transporter membrane subunit DppC

b3541 dppD dipeptide ABC transporter ATP binding subunitDppD

//

>NGHB#3432 9

CMPLX yes

b3543 dppB dipeptide ABC transporter membrane subunit DppB

b3542 dppC dipeptide ABC transporter membrane subunit DppC

//

>NGHB#3433 307

CMPLX yes

b3544 dppA dipeptide ABC transporter periplasmic bindingprotein

b3543 dppB dipeptide ABC transporter membrane subunit DppB

//

>NGHB#3434 1078

CMPLX not

b3546 eptB Kdo2-lipid A phosphoethanolamine7''-transferase

b3544 dppA dipeptide ABC transporter periplasmic bindingprotein

//

>NGHB#3435 323

CMPLX not

b3547 yhjX putative transporter YhjX

b3546 eptB Kdo2-lipid A phosphoethanolamine7''-transferase

//

>NGHB#3436 228

CMPLX not

b3548 yhjY putative outer membrane protein YhjY

b3547 yhjX putative transporter YhjX

//

>NGHB#3438 -4

CMPLX not

b3549 tag 3-methyl-adenine DNA glycosylase I,constitutive

b3550 yiaC peptidyl-lysine N-acetyltransferase YiaC

//

>NGHB#3441 103

CMPLX not

b3552 yiaD PF13488 family lipoprotein YiaD

b3553 ghrB glyoxylate reductase

//

>NGHB#3444 280

CMPLX not

b3555 yiaG putative DNA-binding transcriptional regulatorYiaG

b3556 cspA cold shock protein CspA

//

>NGHB#3445 138

CMPLX not

b3556 cspA cold shock protein CspA

b4793 ysaE protein YsaE

//

>NGHB#3448 -4

CMPLX tnp

b3557 insJ insertion element IS150 protein InsA

b3558 insK IS150 conserved protein InsB

//

>NGHB#3450 9

CMPLX yes

b3560 glyQ glycine--tRNA ligase subunit alpha

b3559 glyS glycine--tRNA ligase subunit beta

//

>NGHB#3451 94

CMPLX not

b4553 ysaB putative lipoprotein YsaB

b3560 glyQ glycine--tRNA ligase subunit alpha

//

>NGHB#3454 45

CMPLX yes

b3563 yiaB PF05360 family inner membrane protein YiaB

b3562 yiaA PF05360 family inner membrane protein YiaA

//

>NGHB#3455 168

CMPLX not

b3564 xylB xylulokinase

b3563 yiaB PF05360 family inner membrane protein YiaB

//

>NGHB#3456 71

CMPLX not

b3565 xylA xylose isomerase

b3564 xylB xylulokinase

//

>NGHB#3458 77

CMPLX yes

b3566 xylF xylose ABC transporter periplasmic bindingprotein

b3567 xylG xylose ABC transporter ATP binding subunit

//

>NGHB#3459 -23

CMPLX yes

b3567 xylG xylose ABC transporter ATP binding subunit

b3568 xylH xylose ABC transporter membrane subunit

//

>NGHB#3460 77

CMPLX not

b3568 xylH xylose ABC transporter membrane subunit

b3569 xylR DNA-binding transcriptional dual regulator XylR

//

>NGHB#3462 -1

CMPLX not

b4794 baxL putative translational regulatory protein BaxL

b3570 bax putative glycoside hydrolase Bax

//

>NGHB#3464 177

CMPLX not

b3571 malS alpha-amylase

b3572 avtA valine--pyruvate aminotransferase

//

>NGHB#3466 101

CMPLX not

b3574 plaR DNA-binding transcriptional repressor PlaR

b3573 ysaA putative electron transport protein YsaA

//

>NGHB#3468 11

CMPLX not

b3575 yiaK 2,3-diketo-L-gulonate reductase

b3576 yiaL DUF386 domain-containing protein YiaL

//

>NGHB#3469 117

CMPLX not

b3576 yiaL DUF386 domain-containing protein YiaL

b3577 yiaM 2,3-diketo-L-gulonate:Na(+) symporter - membranesubunit

//

>NGHB#3470 2

CMPLX yes

b3577 yiaM 2,3-diketo-L-gulonate:Na(+) symporter - membranesubunit

b3578 yiaN 2,3-diketo-L-gulonate:Na(+) symporter - membranesubunit

//

>NGHB#3471 12

CMPLX yes

b3578 yiaN 2,3-diketo-L-gulonate:Na(+) symporter - membranesubunit

b3579 yiaO 2,3-diketo-L-gulonate:Na(+) symporter -periplasmic binding protein

//

>NGHB#3472 3

CMPLX not

b3579 yiaO 2,3-diketo-L-gulonate:Na(+) symporter -periplasmic binding protein

b3580 lyxK L-xylulose kinase

//

>NGHB#3473 -4

CMPLX not

b3580 lyxK L-xylulose kinase

b3581 sgbH 3-keto-L-gulonate-6-phosphate decarboxylaseSgbH

//

>NGHB#3474 -8

CMPLX not

b3581 sgbH 3-keto-L-gulonate-6-phosphate decarboxylaseSgbH

b3582 sgbU putative L-xylulose 5-phosphate 3-epimerase

//

>NGHB#3475 -7

CMPLX not

b3582 sgbU putative L-xylulose 5-phosphate 3-epimerase

b3583 sgbE L-ribulose-5-phosphate 4-epimerase SgbE

//

>NGHB#3479 5

CMPLX not

b3587 yiaW DUF3302 domain-containing protein YiaW

b3586 yiaV putative membrane fusion protein YiaV

//

>NGHB#3480 544

CMPLX not

b3588 aldB aldehyde dehydrogenase B

b3587 yiaW DUF3302 domain-containing protein YiaW

//

>NGHB#3481 164

CMPLX not

b3589 yiaY L-threonine dehydrogenase

b3588 aldB aldehyde dehydrogenase B

//

>NGHB#3482 189

CMPLX not

b3590 selB selenocysteyl-tRNA-specific translationelongation factor

b3589 yiaY L-threonine dehydrogenase

//

>NGHB#3483 -4

CMPLX not

b3591 selA selenocysteine synthase

b3590 selB selenocysteyl-tRNA-specific translationelongation factor

//

>NGHB#3484 97

CMPLX not

b3592 yibF glutathione transferase-like protein YibF

b3591 selA selenocysteine synthase

//

>NGHB#3486 20

CMPLX not

b3593 rhsA rhs element protein RhsA

b3594 yibA putative lyase containing HEAT-repeat

//

>NGHB#3487 997

CMPLX not

b3594 yibA putative lyase containing HEAT-repeat

b3596 yibG tetratricopeptide-like domain-containing proteinYibG

//

>NGHB#3488 706

CMPLX not

b3596 yibG tetratricopeptide-like domain-containing proteinYibG

b4615 yibV PF15596 family protein YibV

//

>NGHB#3490 2

CMPLX not

b3598 yibI DUF3302 domain-containing protein YibI

b3597 yibH inner membrane protein YibH

//

>NGHB#3492 229

CMPLX not

b3599 mtlA mannitol-specific PTS enzyme IICBA component

b3600 mtlD mannitol-1-phosphate 5-dehydrogenase

//

>NGHB#3493 -1

CMPLX not

b3600 mtlD mannitol-1-phosphate 5-dehydrogenase

b3601 mtlR transcriptional repressor MtlR

//

>NGHB#3496 371

CMPLX not

b3602 yibL DUF2810 domain-containing protein YibL

b3603 lldP lactate/glycolate:H(+) symporter LldP

//

>NGHB#3497 -1

CMPLX not

b3603 lldP lactate/glycolate:H(+) symporter LldP

b3604 lldR DNA-binding transcriptional dual regulator LldR

//

>NGHB#3498 -4

CMPLX not

b3604 lldR DNA-binding transcriptional dual regulator LldR

b3605 lldD L-lactate dehydrogenase

//

>NGHB#3499 197

CMPLX not

b3605 lldD L-lactate dehydrogenase

b3606 trmL tRNA (cytidine/uridine-2'-O)-ribosemethyltransferase

//

>NGHB#3501 79

CMPLX not

b3608 gpsA glycerol-3-phosphate dehydrogenase

b3607 cysE serine acetyltransferase

//

>NGHB#3502 -1

CMPLX not

b3609 secB protein export chaperone SecB

b3608 gpsA glycerol-3-phosphate dehydrogenase

//

>NGHB#3503 62

CMPLX not

b3610 grxC glutaredoxin 3

b3609 secB protein export chaperone SecB

//

>NGHB#3504 141

CMPLX not

b3611 yibN putative sulfurtransferase YibN

b3610 grxC glutaredoxin 3

//

>NGHB#3506 33

CMPLX not

b3612 gpmM 2,3-bisphosphoglycerate-independentphosphoglycerate mutase

b3613 envC murein hydrolase activator EnvC

//

>NGHB#3507 3

CMPLX not

b3613 envC murein hydrolase activator EnvC

b3614 yibQ divergent polysaccharide deacetylasedomain-containing protein YibQ

//

>NGHB#3509 238

CMPLX not

b3616 tdh threonine dehydrogenase

b3615 waaH UDP-glucuronate:LPS(HepIII) glycosyltransferase

//

>NGHB#3510 9

CMPLX not

b3617 kbl 2-amino-3-ketobutyrate CoA ligase

b3616 tdh threonine dehydrogenase

//

>NGHB#3511 274

CMPLX not

b3618 htrL protein HtrL

b3617 kbl 2-amino-3-ketobutyrate CoA ligase

//

>NGHB#3513 9

CMPLX not

b3619 rfaD ADP-L-glycero-D-mannoheptose 6-epimerase

b3620 waaF ADP-heptose--LPS heptosyltransferase 2

//

>NGHB#3514 3

CMPLX not

b3620 waaF ADP-heptose--LPS heptosyltransferase 2

b3621 waaC ADP-heptose:LPS heptosyltransferase 1

//

>NGHB#3515 9

CMPLX not

b3621 waaC ADP-heptose:LPS heptosyltransferase 1

b3622 waaL O-antigen ligase

//

>NGHB#3517 192

CMPLX not

b4796 yibY protein YibY

b4795 yibX protein YibX-S

//

>NGHB#3518 89

CMPLX not

b3623 waaU putative ADP-heptose:LPS heptosyltransferase 4

b4796 yibY protein YibY

//

>NGHB#3519 32

CMPLX not

b3624 waaZ lipopolysaccharide core biosynthesis proteinWaaZ

b3623 waaU putative ADP-heptose:LPS heptosyltransferase 4

//

>NGHB#3520 70

CMPLX not

b3625 waaY lipopolysaccharide core heptose (II) kinase

b3624 waaZ lipopolysaccharide core biosynthesis proteinWaaZ

//

>NGHB#3521 17

CMPLX not

b3626 waaJ UDP-glucose:(glucosyl)LPSalpha-1,2-glucosyltransferase

b3625 waaY lipopolysaccharide core heptose (II) kinase

//

>NGHB#3522 39

CMPLX not

b3627 waaO UDP-D-glucose:(glucosyl)LPSalpha-1,3-glucosyltransferase

b3626 waaJ UDP-glucose:(glucosyl)LPSalpha-1,2-glucosyltransferase

//

>NGHB#3523 -1

CMPLX not

b3628 waaB UDP-D-galactose:(glucosyl)lipopolysaccharide-1,6-D-galactosyltransferase

b3627 waaO UDP-D-glucose:(glucosyl)LPSalpha-1,3-glucosyltransferase

//

>NGHB#3524 43

CMPLX not

b3629 waaS lipopolysaccharide core biosynthesis proteinWaaS

b3628 waaB UDP-D-galactose:(glucosyl)lipopolysaccharide-1,6-D-galactosyltransferase

//

>NGHB#3525 36

CMPLX not

b3630 waaP lipopolysaccharide core heptose (I) kinase

b3629 waaS lipopolysaccharide core biosynthesis proteinWaaS

//

>NGHB#3526 -8

CMPLX not

b3631 waaG lipopolysaccharide glucosyltransferase I

b3630 waaP lipopolysaccharide core heptose (I) kinase

//

>NGHB#3527 -4

CMPLX not

b3632 waaQ lipopolysaccharide core heptosyltransferase 3

b3631 waaG lipopolysaccharide glucosyltransferase I

//

>NGHB#3529 7

CMPLX not

b3633 waaA KDO transferase

b3634 coaD pantetheine-phosphate adenylyltransferase

//

>NGHB#3531 97

CMPLX not

b3636 rpmG 50S ribosomal subunit protein L33

b3635 mutM DNA-formamidopyrimidine glycosylase

//

>NGHB#3532 20

CMPLX yes

b3637 rpmB 50S ribosomal subunit protein L28

b3636 rpmG 50S ribosomal subunit protein L33

//

>NGHB#3533 216

CMPLX not

b3638 yicR RadC-like JAB domain-containing protein YicR

b3637 rpmB 50S ribosomal subunit protein L28

//

>NGHB#3535 -23

CMPLX not

b3639 dfp fused 4'-phosphopantothenoylcysteinedecarboxylase and phosphopantothenoylcysteine synthetase

b3640 dut dUTP diphosphatase

//

>NGHB#3536 106

CMPLX not

b3640 dut dUTP diphosphatase

b3641 slmA nucleoid occlusion factor SlmA

//

>NGHB#3538 94

CMPLX not

b3643 rph truncated RNase PH

b3642 pyrE orotate phosphoribosyltransferase

//

>NGHB#3540 220

CMPLX not

b3644 yicC UPF0701 family protein YicC

b3645 dinD DNA damage-inducible protein D

//

>NGHB#3541 289

CMPLX not

b3645 dinD DNA damage-inducible protein D

b3646 yicG PF03458 family inner membrane protein YicG

//

>NGHB#3544 54

CMPLX not

b3648 gmk guanylate kinase

b3649 rpoZ RNA polymerase subunit omega

//

>NGHB#3545 18

CMPLX not

b3649 rpoZ RNA polymerase subunit omega

b3650 spoT bifunctional (p)ppGpp synthase/hydrolase SpoT

//

>NGHB#3546 6

CMPLX not

b3650 spoT bifunctional (p)ppGpp synthase/hydrolase SpoT

b3651 trmH tRNA (Gm18) 2'-O-methyltransferase

//

>NGHB#3547 5

CMPLX not

b3651 trmH tRNA (Gm18) 2'-O-methyltransferase

b3652 recG ATP-dependent DNA helicase RecG

//

>NGHB#3550 120

CMPLX not

b3654 xanP xanthine:H(+) symporter XanP

b3655 yicH AsmA family protein YicH

//

>NGHB#3552 9

CMPLX not

b3657 yicJ putative xyloside transporter YicJ

b3656 yicI alpha-D-xyloside xylohydrolase

//

>NGHB#3554 110

CMPLX not

b3659 setC putative arabinose exporter

b3660 yicL putative transporter YicL

//

>NGHB#3556 473

CMPLX not

b3661 nlpA lipoprotein-28

b4797 yicU protein YicU

//

>NGHB#3559 210

CMPLX not

b3663 yicN DUF1198 domain-containing protein YicN

b3662 nepI purine ribonucleoside exporter

//

>NGHB#3560 52

CMPLX not

b3664 adeQ adenine transporter

b3663 yicN DUF1198 domain-containing protein YicN

//

>NGHB#3563 137

CMPLX not

b3667 uhpC inner membrane protein sensingglucose-6-phosphate

b3666 uhpT hexose-6-phosphate:phosphate antiporter

//

>NGHB#3564 9

CMPLX not

b3668 uhpB sensory histidine kinase UhpB

b3667 uhpC inner membrane protein sensingglucose-6-phosphate

//

>NGHB#3565 -1

CMPLX not

b3669 uhpA DNA-binding transcriptional activator UhpA

b3668 uhpB sensory histidine kinase UhpB

//

>NGHB#3566 75

CMPLX not

b3670 ilvN acetohydroxy acid synthase I subunit IlvN

b3669 uhpA DNA-binding transcriptional activator UhpA

//

>NGHB#3567 3

CMPLX yes

b3671 ilvB acetohydroxy acid synthase I subunit IlvB

b3670 ilvN acetohydroxy acid synthase I subunit IlvN

//

>NGHB#3568 105

CMPLX not

b3672 ivbL ilvBN operon leader peptide

b3671 ilvB acetohydroxy acid synthase I subunit IlvB

//

>NGHB#3573 -4

CMPLX ---

b3675 yidG inner membrane protein YidG

b3674 yidF uncharacterized protein YidF

//

>NGHB#3574 -11

CMPLX not

b3676 yidH DUF202 domain-containing inner membrane proteinYidH

b3675 yidG inner membrane protein YidG

//

>NGHB#3577 -4

CMPLX not

b3679 yidK putative transporter YidK

b3678 yidJ putative sulfatase/phosphatase YidJ

//

>NGHB#3580 -1

CMPLX not

b3682 glvB putative PTS enzyme II component GlvB

b3681 glvG putative inactive phospho-glucosidase

//

>NGHB#3581 24

CMPLX yes

b3683 glvC putative PTS enzyme II component GlvC

b3682 glvB putative PTS enzyme II component GlvB

//

>NGHB#3584 195

CMPLX not

b3686 ibpB small heat shock protein IbpB

b3685 yidE putative transport protein YidE

//

>NGHB#3585 111

CMPLX yes

b3687 ibpA small heat shock protein IbpA

b3686 ibpB small heat shock protein IbpB

//

>NGHB#3590 119

CMPLX not

b4478 dgoD D-galactonate dehydratase

b3691 dgoT D-galactonate:H(+) symporter

//

>NGHB#3591 -4

CMPLX not

b4477 dgoA 2-dehydro-3-deoxy-6-phosphogalactonate aldolase

b4478 dgoD D-galactonate dehydratase

//

>NGHB#3592 -17

CMPLX not

b3693 dgoK 2-dehydro-3-deoxygalactonokinase

b4477 dgoA 2-dehydro-3-deoxy-6-phosphogalactonate aldolase

//

>NGHB#3593 -4

CMPLX not

b4479 dgoR DNA-binding transcriptional regulator DgoR

b3693 dgoK 2-dehydro-3-deoxygalactonokinase

//

>NGHB#3596 114

CMPLX not

b3698 yidB DUF937 domain-containing protein YidB

b3697 yidA sugar phosphatase YidA

//

>NGHB#3597 239

CMPLX not

b3699 gyrB DNA gyrase subunit B

b3698 yidB DUF937 domain-containing protein YidB

//

>NGHB#3598 28

CMPLX not

b3700 recF DNA repair protein RecF

b3699 gyrB DNA gyrase subunit B

//

>NGHB#3599 -1

CMPLX not

b3701 dnaN beta sliding clamp

b3700 recF DNA repair protein RecF

//

>NGHB#3600 4

CMPLX not

b3702 dnaA chromosomal replication initiator protein DnaA

b3701 dnaN beta sliding clamp

//

>NGHB#3602 229

CMPLX not

b4757 ysdD protein YsdD

b3703 rpmH 50S ribosomal subunit protein L34

//

>NGHB#3603 16

CMPLX not

b3703 rpmH 50S ribosomal subunit protein L34

b3704 rnpA RNase P protein component

//

>NGHB#3604 -37

CMPLX not

b3704 rnpA RNase P protein component

b4557 yidD membrane protein insertion efficiency factor

//

>NGHB#3605 2

CMPLX yes

b4557 yidD membrane protein insertion efficiency factor

b3705 yidC membrane protein insertase YidC

//

>NGHB#3606 105

CMPLX not

b3705 yidC membrane protein insertase YidC

b3706 mnmE 5-carboxymethylaminomethyluridine-tRNA synthaseGTPase subunit

//

>NGHB#3607 242

CMPLX not

b3706 mnmE 5-carboxymethylaminomethyluridine-tRNA synthaseGTPase subunit

b3707 tnaC tnaAB operon leader peptide

//

>NGHB#3608 220

CMPLX not

b3707 tnaC tnaAB operon leader peptide

b3708 tnaA tryptophanase

//

>NGHB#3609 90

CMPLX not

b3708 tnaA tryptophanase

b3709 tnaB tryptophan:H(+) symporter TnaB

//

>NGHB#3610 131

CMPLX not

b3709 tnaB tryptophan:H(+) symporter TnaB

b3710 mdtL efflux pump MdtL

//

>NGHB#3611 -26

CMPLX not

b3710 mdtL efflux pump MdtL

b3711 yidZ putative LysR-type transcriptional regulatorYidZ

//

>NGHB#3612 156

CMPLX not

b3711 yidZ putative LysR-type transcriptional regulatorYidZ

b3712 yieE putative phosphopantetheinyl transferase

//

>NGHB#3613 21

CMPLX not

b3712 yieE putative phosphopantetheinyl transferase

b3713 yieF chromate reductase

//

>NGHB#3616 66

CMPLX not

b3715 yieH 6-phosphogluconate phosphatase

b3716 cbrB putative inner membrane protein

//

>NGHB#3617 48

CMPLX not

b3716 cbrB putative inner membrane protein

b3717 cbrC UPF0167 family colicin E2 tolerance proteinCbrC

//

>NGHB#3619 14

CMPLX not

b3719 yieL putative hydrolase YieL

b3718 yieK putative glucosamine-6-phosphate deaminase YieK

//

>NGHB#3620 26

CMPLX not

b3720 bglH carbohydrate-specific outer membrane porin,cryptic

b3719 yieL putative hydrolase YieL

//

>NGHB#3621 68

CMPLX not

b3721 bglB 6-phospho-beta-glucosidase B

b3720 bglH carbohydrate-specific outer membrane porin,cryptic

//

>NGHB#3622 18

CMPLX not

b3722 bglF beta-glucoside specific PTS enzyme II/BglGkinase/BglG phosphatase

b3721 bglB 6-phospho-beta-glucosidase B

//

>NGHB#3623 133

CMPLX not

b3723 bglG transcriptional antiterminator BglG

b3722 bglF beta-glucoside specific PTS enzyme II/BglGkinase/BglG phosphatase

//

>NGHB#3624 285

CMPLX not

b3724 phoU negative regulator of the pho regulon

b3723 bglG transcriptional antiterminator BglG

//

>NGHB#3625 14

CMPLX not

b3725 pstB phosphate ABC transporter ATP binding subunit

b3724 phoU negative regulator of the pho regulon

//

>NGHB#3626 182

CMPLX yes

b3726 pstA phosphate ABC transporter membrane subunit PstA

b3725 pstB phosphate ABC transporter ATP binding subunit

//

>NGHB#3627 -1

CMPLX yes

b3727 pstC phosphate ABC transporter membrane subunit PstC

b3726 pstA phosphate ABC transporter membrane subunit PstA

//

>NGHB#3628 86

CMPLX yes

b3728 pstS phosphate ABC transporter periplasmic bindingprotein

b3727 pstC phosphate ABC transporter membrane subunit PstC

//

>NGHB#3629 313

CMPLX not

b3729 glmS L-glutamine--D-fructose-6-phosphateaminotransferase

b3728 pstS phosphate ABC transporter periplasmic bindingprotein

//

>NGHB#3630 161

CMPLX not

b3730 glmU fused N-acetylglucosamine-1-phosphate uridyltransferase and glucosamine-1-phosphateacetyltransferase

b3729 glmS L-glutamine--D-fructose-6-phosphateaminotransferase

//

>NGHB#3631 352

CMPLX not

b3731 atpC ATP synthase F1 complex subunit epsilon

b3730 glmU fused N-acetylglucosamine-1-phosphate uridyltransferase and glucosamine-1-phosphateacetyltransferase

//

>NGHB#3632 20

CMPLX yes

b3732 atpD ATP synthase F1 complex subunit beta

b3731 atpC ATP synthase F1 complex subunit epsilon

//

>NGHB#3633 26

CMPLX yes

b3733 atpG ATP synthase F1 complex subunit gamma

b3732 atpD ATP synthase F1 complex subunit beta

//

>NGHB#3634 50

CMPLX yes

b3734 atpA ATP synthase F1 complex subunit alpha

b3733 atpG ATP synthase F1 complex subunit gamma

//

>NGHB#3635 12

CMPLX yes

b3735 atpH ATP synthase F1 complex subunit delta

b3734 atpA ATP synthase F1 complex subunit alpha

//

>NGHB#3636 14

CMPLX yes

b3736 atpF ATP synthase Fo complex subunit b

b3735 atpH ATP synthase F1 complex subunit delta

//

>NGHB#3637 61

CMPLX yes

b3737 atpE ATP synthase Fo complex subunit c

b3736 atpF ATP synthase Fo complex subunit b

//

>NGHB#3638 46

CMPLX yes

b3738 atpB ATP synthase Fo complex subunit a

b3737 atpE ATP synthase Fo complex subunit c

//

>NGHB#3639 8

CMPLX yes

b3739 atpI ATP synthase accessory factor AtpI

b3738 atpB ATP synthase Fo complex subunit a

//

>NGHB#3640 616

CMPLX not

b3740 rsmG 16S rRNA m(7)G527 methyltransferase

b3739 atpI ATP synthase accessory factor AtpI

//

>NGHB#3641 63

CMPLX not

b3741 mnmG 5-carboxymethylaminomethyluridine-tRNA synthasesubunit MnmG

b3740 rsmG 16S rRNA m(7)G527 methyltransferase

//

>NGHB#3642 378

CMPLX not

b3742 mioC flavoprotein MioC

b3741 mnmG 5-carboxymethylaminomethyluridine-tRNA synthasesubunit MnmG

//

>NGHB#3643 89

CMPLX not

b3743 asnC DNA-binding transcriptional dual regulator AsnC

b3742 mioC flavoprotein MioC

//

>NGHB#3646 -7

CMPLX not

b3746 ravA regulatory ATPase RavA

b3745 viaA protein ViaA

//

>NGHB#3648 166

CMPLX not

b3747 kup K(+):H(+) symporter Kup

b3748 rbsD D-ribose pyranase

//

>NGHB#3649 7

CMPLX not

b3748 rbsD D-ribose pyranase

b3749 rbsA ribose ABC transporter ATP binding subunit

//

>NGHB#3650 4

CMPLX yes

b3749 rbsA ribose ABC transporter ATP binding subunit

b3750 rbsC ribose ABC transporter membrane subunit

//

>NGHB#3651 24

CMPLX yes

b3750 rbsC ribose ABC transporter membrane subunit

b3751 rbsB ribose ABC transporter periplasmic bindingprotein

//

>NGHB#3652 125

CMPLX not

b3751 rbsB ribose ABC transporter periplasmic bindingprotein

b3752 rbsK ribokinase

//

>NGHB#3653 3

CMPLX not

b3752 rbsK ribokinase

b3753 rbsR DNA-binding transcriptional dual regulator RbsR

//

>NGHB#3655 22

CMPLX not

b3755 yieP DNA-binding transcriptional regulator YieP

b3754 hsrA putative transporter HsrA

//

>NGHB#3656 5800

CMPLX not

b4480 hdfR DNA-binding transcriptional dual regulator HdfR

b3755 yieP DNA-binding transcriptional regulator YieP

//

>NGHB#3660 86

CMPLX ---

b3766 ilvL ilvXGMEDA operon leader peptide

b4669 ilvX uncharacterized protein IlvX

//

>NGHB#3661 1643

CMPLX ---

b4669 ilvX uncharacterized protein IlvX

b3769 ilvM acetolactate synthase II subunit IlvM

//

>NGHB#3662 19

CMPLX not

b3769 ilvM acetolactate synthase II subunit IlvM

b3770 ilvE branched-chain-amino-acid aminotransferase

//

>NGHB#3663 64

CMPLX not

b3770 ilvE branched-chain-amino-acid aminotransferase

b3771 ilvD dihydroxy-acid dehydratase

//

>NGHB#3664 2

CMPLX not

b3771 ilvD dihydroxy-acid dehydratase

b3772 ilvA threonine deaminase

//

>NGHB#3670 135

CMPLX not

b3780 rhlB ATP-dependent RNA helicase RhlB

b3779 gpp guanosine-5'-triphosphate,3'-diphosphatephosphatase

//

>NGHB#3672 140

CMPLX not

b3781 trxA thioredoxin 1

b3782 rhoL rho operon leader peptide

//

>NGHB#3673 84

CMPLX not

b3782 rhoL rho operon leader peptide

b3783 rho transcription termination factor Rho

//

>NGHB#3674 239

CMPLX not

b3783 rho transcription termination factor Rho

b3784 rfe UDP-N-acetylglucosamine--undecaprenyl-phosphateN-acetylglucosaminephosphotransferase

//

>NGHB#3675 11

CMPLX not

b3784 rfe UDP-N-acetylglucosamine--undecaprenyl-phosphateN-acetylglucosaminephosphotransferase

b3785 wzzE enterobacterial common antigen polysaccharideco-polymerase

//

>NGHB#3676 55

CMPLX not

b3785 wzzE enterobacterial common antigen polysaccharideco-polymerase

b3786 wecB UDP-N-acetylglucosamine 2-epimerase

//

>NGHB#3677 -4

CMPLX not

b3786 wecB UDP-N-acetylglucosamine 2-epimerase

b3787 wecC UDP-N-acetyl-D-mannosamine dehydrogenase

//

>NGHB#3678 -1

CMPLX not

b3787 wecC UDP-N-acetyl-D-mannosamine dehydrogenase

b3788 rffG dTDP-glucose 4,6-dehydratase 2

//

>NGHB#3679 18

CMPLX not

b3788 rffG dTDP-glucose 4,6-dehydratase 2

b3789 rffH dTDP-glucose pyrophosphorylase 2

//

>NGHB#3680 -23

CMPLX not

b3789 rffH dTDP-glucose pyrophosphorylase 2

b3790 rffC dTDP-4-amino-4,6-dideoxy-D-galactoseacyltransferase

//

>NGHB#3681 4

CMPLX not

b3790 rffC dTDP-4-amino-4,6-dideoxy-D-galactoseacyltransferase

b3791 wecE dTDP-4-dehydro-6-deoxy-D-glucose transaminase

//

>NGHB#3682 1

CMPLX not

b3791 wecE dTDP-4-dehydro-6-deoxy-D-glucose transaminase

b3792 wzxE lipid III flippase

//

>NGHB#3683 -4

CMPLX not

b3792 wzxE lipid III flippase

b4481 wecF TDP-N-acetylfucosamine:lipid IIN-acetylfucosaminyltransferase

//

>NGHB#3684 -4

CMPLX not

b4481 wecF TDP-N-acetylfucosamine:lipid IIN-acetylfucosaminyltransferase

b3793 wzyE putative enterobacterial common antigenpolymerase

//

>NGHB#3685 2

CMPLX not

b3793 wzyE putative enterobacterial common antigenpolymerase

b3794 rffM UDP-N-acetyl-D-mannosaminuronic acidtransferase

//

>NGHB#3686 190

CMPLX not

b3794 rffM UDP-N-acetyl-D-mannosaminuronic acidtransferase

b3795 yifK putative transporter YifK

//

>NGHB#3687 685

CMPLX not

b3795 yifK putative transporter YifK

b3800 aslB putative anaerobic sulfatase maturation enzymeAslB

//

>NGHB#3689 678

CMPLX not

b3802 hemY protein HemY

b3801 aslA putative sulfatase AslA

//

>NGHB#3690 2

CMPLX not

b3803 hemX PF04375 family protein HemX

b3802 hemY protein HemY

//

>NGHB#3691 21

CMPLX not

b3804 hemD uroporphyrinogen-III synthase

b3803 hemX PF04375 family protein HemX

//

>NGHB#3692 -4

CMPLX not

b3805 hemC hydroxymethylbilane synthase

b3804 hemD uroporphyrinogen-III synthase

//

>NGHB#3696 186

CMPLX not

b3808 yzcX protein YzcX

b4558 yifL putative lipoprotein YifL

//

>NGHB#3697 36

CMPLX not

b4558 yifL putative lipoprotein YifL

b3809 dapF diaminopimelate epimerase

//

>NGHB#3698 -4

CMPLX not

b3809 dapF diaminopimelate epimerase

b3810 yigA DUF484 domain-containing protein YigA

//

>NGHB#3699 -4

CMPLX not

b3810 yigA DUF484 domain-containing protein YigA

b3811 xerC site-specific tyrosine recombinase

//

>NGHB#3700 -1

CMPLX not

b3811 xerC site-specific tyrosine recombinase

b3812 yigB 5-amino-6-(5-phospho-D-ribitylamino)uracilphosphatase

//

>NGHB#3701 83

CMPLX not

b3812 yigB 5-amino-6-(5-phospho-D-ribitylamino)uracilphosphatase

b3813 uvrD DNA helicase II

//

>NGHB#3704 151

CMPLX not

b4799 ysgD protein YsgD

b3816 corA Ni(2(+))/Co(2(+))/Mg(2(+)) transporter

//

>NGHB#3706 13

CMPLX not

b3818 yigG inner membrane protein

b3817 yigF DUF2628 domain-containing protein YigF

//

>NGHB#3707 94

CMPLX not

b3819 rarD putative transporter RarD

b3818 yigG inner membrane protein

//

>NGHB#3708 51

CMPLX not

b3820 yigI putative thioesterase YigI

b3819 rarD putative transporter RarD

//

>NGHB#3710 132

CMPLX not

b3821 pldA outer membrane phospholipase A

b3822 recQ ATP-dependent DNA helicase RecQ

//

>NGHB#3711 63

CMPLX not

b3822 recQ ATP-dependent DNA helicase RecQ

b3823 rhtC L-threonine exporter

//

>NGHB#3714 7

CMPLX not

b3825 pldB lysophospholipase L2

b3826 yigL phosphosugar phosphatase YigL

//

>NGHB#3715 75

CMPLX not

b3826 yigL phosphosugar phosphatase YigL

b3827 bioP biotin transporter

//

>NGHB#3720 140

CMPLX not

b3831 udp uridine phosphorylase

b3832 rmuC putative recombination limiting protein RmuC

//

>NGHB#3721 94

CMPLX not

b3832 rmuC putative recombination limiting protein RmuC

b3833 ubiE bifunctional 2-octaprenyl-6-methoxy-1,4-benzoquinol methylase anddemethylmenaquinone methyltransferase

//

>NGHB#3722 13

CMPLX not

b3833 ubiE bifunctional 2-octaprenyl-6-methoxy-1,4-benzoquinol methylase anddemethylmenaquinone methyltransferase

b3834 ubiJ ubiquinone biosynthesis accessory factor UbiJ

//

>NGHB#3723 -4

CMPLX not

b3834 ubiJ ubiquinone biosynthesis accessory factor UbiJ

b3835 ubiB ubiquinone biosynthesis protein UbiB

//

>NGHB#3724 78

CMPLX not

b3835 ubiB ubiquinone biosynthesis protein UbiB

b3836 tatA twin arginine protein translocation system -TatA protein

//

>NGHB#3725 3

CMPLX yes

b3836 tatA twin arginine protein translocation system -TatA protein

b3838 tatB twin arginine protein translocation system -TatB protein

//

>NGHB#3726 2

CMPLX yes

b3838 tatB twin arginine protein translocation system -TatB protein

b3839 tatC twin arginine protein translocation system -TatC protein

//

>NGHB#3727 41

CMPLX not

b3839 tatC twin arginine protein translocation system -TatC protein

b4483 tatD 3' -> 5' ssDNA/RNA exonuclease TatD

//

>NGHB#3730 45

CMPLX not

b3843 ubiD 3-octaprenyl-4-hydroxybenzoate decarboxylase

b3844 fre NAD(P)H-flavin reductase

//

>NGHB#3732 9

CMPLX not

b3846 fadB multifunctional enoyl-CoA hydratase, 3-hydroxyacyl-CoA epimerase, Delta(3)-cis- Delta(2)-trans-enoyl-CoA isomerase, L-3-hydroxyacyl-CoAdehydrogenase

b3845 fadA 3-ketoacyl-CoA thiolase

//

>NGHB#3734 -1

CMPLX not

b3847 pepQ Xaa-Pro dipeptidase

b3848 yigZ IMPACT family member YigZ

//

>NGHB#3735 38

CMPLX not

b3848 yigZ IMPACT family member YigZ

b3849 trkH K(+) transporter TrkH

//

>NGHB#3736 11

CMPLX not

b3849 trkH K(+) transporter TrkH

b3850 hemG protoporphyrinogen oxidase

//

>NGHB#3738 -19

CMPLX not

b3857 mobA molybdenum cofactor guanylyltransferase

b3856 mobB molybdopterin-guanine dinucleotide biosynthesisadaptor protein

//

>NGHB#3740 76

CMPLX not

b3858 yihD DUF1040 domain-containing protein YihD

b3859 srkA stress response kinase A

//

>NGHB#3741 16

CMPLX not

b3859 srkA stress response kinase A

b3860 dsbA thiol:disulfide oxidoreductase DsbA

//

>NGHB#3742 154

CMPLX not

b3860 dsbA thiol:disulfide oxidoreductase DsbA

b3861 yihF DUF945 domain-containing protein YihF

//

>NGHB#3747 188

CMPLX not

b3866 yihI Der GTPase-activating protein YihI

b3867 hemN coproporphyrinogen III dehydrogenase

//

>NGHB#3749 111

CMPLX ---

b3868 glnG DNA-binding transcriptional dual regulator NtrC

b4686 yshB uncharacterized protein YshB

//

>NGHB#3750 11

CMPLX not

b3869 glnL sensory histidine kinase NtrB

b3868 glnG DNA-binding transcriptional dual regulator NtrC

//

>NGHB#3751 285

CMPLX not

b3870 glnA glutamine synthetase

b3869 glnL sensory histidine kinase NtrB

//

>NGHB#3753 216

CMPLX not

b3871 bipA 50S ribosomal subunit assembly factor BipA

b3872 yihL putative transcriptional regulator YihL

//

>NGHB#3754 7

CMPLX not

b3872 yihL putative transcriptional regulator YihL

b3873 yihM putative TIM barrel domain-containing proteinYihM

//

>NGHB#3755 101

CMPLX not

b3873 yihM putative TIM barrel domain-containing proteinYihM

b3874 yihN putative transporter YihN

//

>NGHB#3757 67

CMPLX not

b3876 yihO putative sulfoquinovose transporter

b3875 ompL putative outer membrane porin L

//

>NGHB#3758 42

CMPLX not

b3877 yihP putative 2,3-dihydroxypropane-1-sulfonate exportprotein

b3876 yihO putative sulfoquinovose transporter

//

>NGHB#3759 45

CMPLX not

b3878 yihQ sulfoquinovosidase

b3877 yihP putative 2,3-dihydroxypropane-1-sulfonate exportprotein

//

>NGHB#3760 198

CMPLX not

b3879 yihR putative sulfoquinovose mutarotase YihR

b3878 yihQ sulfoquinovosidase

//

>NGHB#3761 113

CMPLX not

b3880 yihS sulfoquinovose isomerase

b3879 yihR putative sulfoquinovose mutarotase YihR

//

>NGHB#3762 16

CMPLX not

b3881 yihT 6-deoxy-6-sulfofructose-1-phosphate aldolase

b3880 yihS sulfoquinovose isomerase

//

>NGHB#3763 23

CMPLX not

b3882 yihU 3-sulfolactaldehyde reductase

b3881 yihT 6-deoxy-6-sulfofructose-1-phosphate aldolase

//

>NGHB#3765 33

CMPLX not

b3883 yihV 6-deoxy-6-sulfofructose kinase

b3884 csqR DNA-binding transcriptional dual regulator CsqR

//

>NGHB#3766 98

CMPLX not

b3884 csqR DNA-binding transcriptional dual regulator CsqR

b3885 yihX alpha-D-glucose-1-phosphate phosphatase YihX

//

>NGHB#3767 -7

CMPLX not

b3885 yihX alpha-D-glucose-1-phosphate phosphatase YihX

b3886 yihY PF03631 family membrane protein YihY

//

>NGHB#3768 -4

CMPLX not

b3886 yihY PF03631 family membrane protein YihY

b3887 dtd D-aminoacyl-tRNA deacylase

//

>NGHB#3769 -4

CMPLX not

b3887 dtd D-aminoacyl-tRNA deacylase

b3888 fabY putative fatty acid biosynthesis enzyme FabY

//

>NGHB#3770 852

CMPLX not

b3888 fabY putative fatty acid biosynthesis enzyme FabY

b3889 yiiE putative DNA-binding transcriptional regulatorYiiE

//

>NGHB#3771 241

CMPLX not

b3889 yiiE putative DNA-binding transcriptional regulatorYiiE

b3890 yiiF protein YiiF

//

>NGHB#3773 -4

CMPLX not

b3892 fdoI formate dehydrogenase O subunit gamma

b3891 fdhE formate dehydrogenase formation protein

//

>NGHB#3774 -4

CMPLX yes

b3893 fdoH formate dehydrogenase O subunit beta

b3892 fdoI formate dehydrogenase O subunit gamma

//

>NGHB#3775 12

CMPLX yes

b3894 fdoG formate dehydrogenase O subunit alpha

b3893 fdoH formate dehydrogenase O subunit beta

//

>NGHB#3777 152

CMPLX not

b3895 fdhD sulfurtransferase for molybdenum cofactorsulfuration

b3896 yiiG DUF3829 domain-containing lipoprotein YiiG

//

>NGHB#3779 -1

CMPLX not

b3898 frvX peptidase M42 family protein FrvX

b3897 frvR putative transcriptional regulator FrvR

//

>NGHB#3780 -11

CMPLX not

b3899 frvB putative PTS enzyme IIBC component FrvB

b3898 frvX peptidase M42 family protein FrvX

//

>NGHB#3781 10

CMPLX yes

b3900 frvA putative PTS enzyme IIA component FrvA

b3899 frvB putative PTS enzyme IIBC component FrvB

//

>NGHB#3782 300

CMPLX not

b3901 rhaM L-rhamnose mutarotase

b3900 frvA putative PTS enzyme IIA component FrvA

//

>NGHB#3783 9

CMPLX not

b3902 rhaD rhamnulose-1-phosphate aldolase

b3901 rhaM L-rhamnose mutarotase

//

>NGHB#3784 450

CMPLX not

b3903 rhaA L-rhamnose isomerase

b3902 rhaD rhamnulose-1-phosphate aldolase

//

>NGHB#3785 -4

CMPLX not

b3904 rhaB rhamnulokinase

b3903 rhaA L-rhamnose isomerase

//

>NGHB#3787 73

CMPLX yes

b3905 rhaS DNA-binding transcriptional activator RhaS

b3906 rhaR DNA-binding transcriptional activator RhaR

//

>NGHB#3790 259

CMPLX not

b3908 sodA superoxide dismutase (Mn)

b3909 kdgT 2-dehydro-3-deoxy-D-gluconate:H(+) symporter

//

>NGHB#3791 148

CMPLX not

b3909 kdgT 2-dehydro-3-deoxy-D-gluconate:H(+) symporter

b3910 yiiM 2-amino-6-N-hydroxylaminopurine resistanceprotein

//

>NGHB#3793 -4

CMPLX not

b3912 cpxR DNA-binding transcriptional dual regulator CpxR

b3911 cpxA sensor histidine kinase CpxA

//

>NGHB#3795 148

CMPLX not

b4484 cpxP periplasmic protein CpxP

b3915 fieF Zn(2(+))/Fe(2(+))/Cd(2(+)) exporter

//

>NGHB#3796 180

CMPLX not

b3915 fieF Zn(2(+))/Fe(2(+))/Cd(2(+)) exporter

b3916 pfkA 6-phosphofructokinase 1

//

>NGHB#3797 319

CMPLX not

b3916 pfkA 6-phosphofructokinase 1

b3917 sbp sulfate/thiosulfate ABC transporter periplasmicbinding protein Sbp

//

>NGHB#3798 106

CMPLX not

b3917 sbp sulfate/thiosulfate ABC transporter periplasmicbinding protein Sbp

b3918 cdh CDP-diacylglycerol diphosphatase

//

>NGHB#3800 107

CMPLX not

b3920 yiiQ DUF1454 domain-containing protein YiiQ

b3919 tpiA triose-phosphate isomerase

//

>NGHB#3802 211

CMPLX not

b3921 yiiR DUF805 domain-containing protein YiiR

b3922 yiiS DUF406 domain-containing protein YiiS

//

>NGHB#3803 26

CMPLX not

b3922 yiiS DUF406 domain-containing protein YiiS

b3923 uspD universal stress protein D

//

>NGHB#3805 96

CMPLX not

b3925 glpX fructose-1,6-bisphosphatase 2

b3924 fpr flavodoxin/ferredoxin-NADP(+) reductase

//

>NGHB#3806 134

CMPLX not

b3926 glpK glycerol kinase

b3925 glpX fructose-1,6-bisphosphatase 2

//

>NGHB#3807 22

CMPLX not

b3927 glpF glycerol facilitator

b3926 glpK glycerol kinase

//

>NGHB#3810 92

CMPLX not

b3930 menA 1,4-dihydroxy-2-naphthoateoctaprenyltransferase

b3929 rraA ribonuclease E inhibitor protein A

//

>NGHB#3811 66

CMPLX not

b3931 hslU ATPase component of the HslVU protease

b3930 menA 1,4-dihydroxy-2-naphthoateoctaprenyltransferase

//

>NGHB#3812 9

CMPLX not

b3932 hslV peptidase component of the HslVU protease

b3931 hslU ATPase component of the HslVU protease

//

>NGHB#3813 92

CMPLX not

b3933 ftsN cell division protein FtsN

b3932 hslV peptidase component of the HslVU protease

//

>NGHB#3814 91

CMPLX not

b3934 cytR DNA-binding transcriptional repressor CytR

b3933 ftsN cell division protein FtsN

//

>NGHB#3815 155

CMPLX not

b3935 priA primosome factor N'

b3934 cytR DNA-binding transcriptional repressor CytR

//

>NGHB#3818 183

CMPLX not

b3938 metJ DNA-binding transcriptional repressor MetJ

b3937 yiiX putative lipid-binding hydrolase YiiX

//

>NGHB#3820 2

CMPLX not

b3939 metB O-succinylhomoserine(thiol)-lyase/O-succinylhomoserine lyase

b3940 metL fused aspartate kinase/homoserine dehydrogenase2

//

>NGHB#3821 348

CMPLX not

b3940 metL fused aspartate kinase/homoserine dehydrogenase2

b3941 metF 5,10-methylenetetrahydrofolate reductase

//

>NGHB#3822 328

CMPLX not

b3941 metF 5,10-methylenetetrahydrofolate reductase

b3942 katG catalase/hydroperoxidase HPI

//

>NGHB#3823 92

CMPLX not

b3942 katG catalase/hydroperoxidase HPI

b3943 yijE cystine exporter

//

>NGHB#3825 274

CMPLX not

b3945 gldA L-1,2-propanediol dehydrogenase/glyceroldehydrogenase

b3944 yijF DUF1287 domain-containing protein YijF

//

>NGHB#3826 10

CMPLX not

b3946 fsaB fructose-6-phosphate aldolase 2

b3945 gldA L-1,2-propanediol dehydrogenase/glyceroldehydrogenase

//

>NGHB#3827 11

CMPLX not

b3947 ptsA putative PTS multiphosphoryl transfer proteinPtsA

b3946 fsaB fructose-6-phosphate aldolase 2

//

>NGHB#3829 14

CMPLX yes

b3949 frwC putative PTS enzyme IIC component FrwC

b3950 frwB putative PTS enzyme IIB component FrwB

//

>NGHB#3830 50

CMPLX not

b3950 frwB putative PTS enzyme IIB component FrwB

b3951 pflD putative formate acetyltransferase 2

//

>NGHB#3831 -35

CMPLX not

b3951 pflD putative formate acetyltransferase 2

b3952 pflC putative pyruvate formate-lyase 2 activatingenzyme PflC

//

>NGHB#3832 1

CMPLX not

b3952 pflC putative pyruvate formate-lyase 2 activatingenzyme PflC

b3953 frwD putative PTS enzyme IIB component FrwD

//

>NGHB#3834 214

CMPLX not

b3955 eptC phosphoethanolamine transferase EptC

b3954 yijO putative DNA-binding transcriptional regulatorYijO

//

>NGHB#3835 181

CMPLX not

b3956 ppc phosphoenolpyruvate carboxylase

b3955 eptC phosphoethanolamine transferase EptC

//

>NGHB#3836 597

CMPLX not

b3957 argE acetylornithine deacetylase

b3956 ppc phosphoenolpyruvate carboxylase

//

>NGHB#3838 7

CMPLX not

b3958 argC N-acetylglutamylphosphate reductase

b3959 argB acetylglutamate kinase

//

>NGHB#3839 60

CMPLX not

b3959 argB acetylglutamate kinase

b3960 argH argininosuccinate lyase

//

>NGHB#3840 266

CMPLX not

b3960 argH argininosuccinate lyase

b3961 oxyR DNA-binding transcriptional dual regulator OxyR

//

>NGHB#3843 -1

CMPLX not

b3963 fabR DNA-binding transcriptional repressor FabR

b3964 yijD DUF1422 domain-containing inner membrane proteinYijD

//

>NGHB#3846 -56

CMPLX not

b3966 btuB cobalamin outer membrane transporter

b3967 murI glutamate racemase

//

>NGHB#3847 5771

CMPLX not

b3967 murI glutamate racemase

b3972 murB UDP-N-acetylenolpyruvoylglucosamine reductase

//

>NGHB#3848 -4

CMPLX not

b3972 murB UDP-N-acetylenolpyruvoylglucosamine reductase

b3973 birA DNA-binding transcriptionalrepressor/biotin-[acetyl-CoA-carboxylase] ligase BirA

//

>NGHB#3851 229

CMPLX not

b3980 tufB translation elongation factor Tu 2

b3981 secE Sec translocon subunit SecE

//

>NGHB#3852 1

CMPLX not

b3981 secE Sec translocon subunit SecE

b3982 nusG transcription termination factor NusG

//

>NGHB#3853 158

CMPLX not

b3982 nusG transcription termination factor NusG

b3983 rplK 50S ribosomal subunit protein L11

//

>NGHB#3854 3

CMPLX yes

b3983 rplK 50S ribosomal subunit protein L11

b3984 rplA 50S ribosomal subunit protein L1

//

>NGHB#3855 412

CMPLX yes

b3984 rplA 50S ribosomal subunit protein L1

b3985 rplJ 50S ribosomal subunit protein L10

//

>NGHB#3856 66

CMPLX yes

b3985 rplJ 50S ribosomal subunit protein L10

b3986 rplL 50S ribosomal subunit protein L12

//

>NGHB#3857 319

CMPLX not

b3986 rplL 50S ribosomal subunit protein L12

b3987 rpoB RNA polymerase subunit beta

//

>NGHB#3858 76

CMPLX yes

b3987 rpoB RNA polymerase subunit beta

b3988 rpoC RNA polymerase subunit beta'

//

>NGHB#3859 212

CMPLX not

b3988 rpoC RNA polymerase subunit beta'

b3989 yjaZ protein YjaZ

//

>NGHB#3861 -4

CMPLX not

b3991 thiG 1-deoxy-D-xylulose 5-phosphate:thiolsulfurtransferase

b3990 thiH 2-iminoacetate synthase

//

>NGHB#3862 1

CMPLX not

b4407 thiS sulfur carrier protein ThiS

b3991 thiG 1-deoxy-D-xylulose 5-phosphate:thiolsulfurtransferase

//

>NGHB#3863 -17

CMPLX not

b3992 thiF sulfur carrier protein ThiS adenylyltransferase

b4407 thiS sulfur carrier protein ThiS

//

>NGHB#3864 -8

CMPLX not

b3993 thiE thiamine phosphate synthase

b3992 thiF sulfur carrier protein ThiS adenylyltransferase

//

>NGHB#3865 -1

CMPLX not

b3994 thiC phosphomethylpyrimidine synthase

b3993 thiE thiamine phosphate synthase

//

>NGHB#3866 232

CMPLX not

b3995 rsd regulator of sigma D

b3994 thiC phosphomethylpyrimidine synthase

//

>NGHB#3868 39

CMPLX not

b3996 nudC RNA decapping hydrolase

b3997 hemE uroporphyrinogen decarboxylase

//

>NGHB#3869 9

CMPLX not

b3997 hemE uroporphyrinogen decarboxylase

b3998 nfi endonuclease V

//

>NGHB#3870 42

CMPLX not

b3998 nfi endonuclease V

b3999 yjaG DUF416 domain-containing protein YjaG

//

>NGHB#3871 186

CMPLX not

b3999 yjaG DUF416 domain-containing protein YjaG

b4000 hupA DNA-binding protein HU-alpha

//

>NGHB#3872 12

CMPLX not

b4000 hupA DNA-binding protein HU-alpha

b4001 yjaH DUF1481 domain-containing protein YjaH

//

>NGHB#3875 -4

CMPLX not

b4003 zraS sensor histidine kinase ZraS

b4004 zraR DNA-binding transcriptional activator ZraR

//

>NGHB#3877 11

CMPLX not

b4006 purH bifunctional AICAR transformylase/IMPcyclohydrolase

b4005 purD phosphoribosylamine--glycine ligase

//

>NGHB#3881 268

CMPLX not

b4013 metA homoserine O-succinyltransferase

b4014 aceB malate synthase A

//

>NGHB#3882 29

CMPLX not

b4014 aceB malate synthase A

b4015 aceA isocitrate lyase

//

>NGHB#3883 182

CMPLX not

b4015 aceA isocitrate lyase

b4016 aceK isocitrate dehydrogenase kinase/phosphatase

//

>NGHB#3885 316

CMPLX not

b4018 iclR DNA-binding transcriptional repressor IclR

b4017 arpA regulator of acetyl CoA synthetase

//

>NGHB#3887 219

CMPLX not

b4019 metH cobalamin-dependent methionine synthase

b4020 yjbB putative inorganic phosphate export proteinYjbB

//

>NGHB#3891 252

CMPLX not

b4024 lysC aspartate kinase III

b4023 yjbD DUF3811 domain-containing protein YjbD

//

>NGHB#3893 498

CMPLX ---

b4025 pgi glucose-6-phosphate isomerase

b4026 yjbE uncharacterized protein YjbE

//

>NGHB#3894 113

CMPLX ---

b4026 yjbE uncharacterized protein YjbE

b4027 yjbF lipoprotein YjbF

//

>NGHB#3895 -4

CMPLX not

b4027 yjbF lipoprotein YjbF

b4028 yjbG capsule biosynthesis GfcC family protein YjbG

//

>NGHB#3896 -1

CMPLX not

b4028 yjbG capsule biosynthesis GfcC family protein YjbG

b4029 yjbH YjbH family protein

//

>NGHB#3900 371

CMPLX not

b4032 malG maltose ABC transporter membrane subunit MalG

b4031 xylE D-xylose:H(+) symporter

//

>NGHB#3901 14

CMPLX yes

b4033 malF maltose ABC transporter membrane subunit MalF

b4032 malG maltose ABC transporter membrane subunit MalG

//

>NGHB#3902 153

CMPLX yes

b4034 malE maltose ABC transporter periplasmic bindingprotein

b4033 malF maltose ABC transporter membrane subunit MalF

//

>NGHB#3904 71

CMPLX not

b4035 malK maltose ABC transporter ATP binding subunit

b4036 lamB maltose outer membrane channel/phage lambdareceptor protein

//

>NGHB#3905 242

CMPLX not

b4036 lamB maltose outer membrane channel/phage lambdareceptor protein

b4037 malM maltose regulon periplasmic protein

//

>NGHB#3906 2031

CMPLX not

b4037 malM maltose regulon periplasmic protein

b4039 ubiC chorismate lyase

//

>NGHB#3907 12

CMPLX not

b4039 ubiC chorismate lyase

b4040 ubiA 4-hydroxybenzoate octaprenyltransferase

//

>NGHB#3910 109

CMPLX not

b4042 dgkA diacylglycerol kinase

b4043 lexA DNA-binding transcriptional repressor LexA

//

>NGHB#3911 18

CMPLX not

b4043 lexA DNA-binding transcriptional repressor LexA

b4044 dinF DNA damage-inducible protein F

//

>NGHB#3912 115

CMPLX not

b4044 dinF DNA damage-inducible protein F

b4045 yjbJ putative stress response protein YjbJ

//

>NGHB#3915 23

CMPLX ---

b4047 yjbL uncharacterized protein YjbL

b4048 yjbM DUF2713 domain-containing protein YjbM

//

>NGHB#3916 362

CMPLX not

b4048 yjbM DUF2713 domain-containing protein YjbM

b4049 dusA tRNA-dihydrouridine synthase A

//

>NGHB#3917 133

CMPLX not

b4049 dusA tRNA-dihydrouridine synthase A

b4050 pspG phage shock protein G

//

>NGHB#3920 52

CMPLX not

b4052 dnaB replicative DNA helicase

b4053 alr alanine racemase 1

//

>NGHB#3921 252

CMPLX not

b4053 alr alanine racemase 1

b4054 tyrB tyrosine aminotransferase

//

>NGHB#3924 110

CMPLX not

b4055 aphA acid phosphatase/phosphotransferase

b4056 yjbQ UPF0047 protein YjbQ

//

>NGHB#3925 3

CMPLX not

b4056 yjbQ UPF0047 protein YjbQ

b4057 yjbR PF04237 family protein YjbR

//

>NGHB#3932 545

CMPLX not

b4063 soxR DNA-binding transcriptional dual regulator SoxR

b4064 ghxP guanine/hypoxanthine transporter GhxP

//

>NGHB#3933 151

CMPLX not

b4064 ghxP guanine/hypoxanthine transporter GhxP

b4065 yjcE putative transporter YjcE

//

>NGHB#3935 177

CMPLX not

b4067 actP acetate/glycolate:cation symporter

b4066 yjcF pentapeptide repeat-containing protein YjcF

//

>NGHB#3936 -4

CMPLX not

b4068 yjcH DUF485 domain-containing inner membrane proteinYjcH

b4067 actP acetate/glycolate:cation symporter

//

>NGHB#3937 199

CMPLX not

b4069 acs acetyl-CoA synthetase (AMP-forming)

b4068 yjcH DUF485 domain-containing inner membrane proteinYjcH

//

>NGHB#3939 44

CMPLX not

b4070 nrfA cytochrome c552 nitrite reductase

b4071 nrfB periplasmic nitrite reductase penta-heme c-typecytochrome

//

>NGHB#3940 -4

CMPLX not

b4071 nrfB periplasmic nitrite reductase penta-heme c-typecytochrome

b4072 nrfC putative menaquinol-cytochrome c reductase4Fe-4S subunit

//

>NGHB#3941 -4

CMPLX yes

b4072 nrfC putative menaquinol-cytochrome c reductase4Fe-4S subunit

b4073 nrfD putative menaquinol-cytochrome c reductasesubunit NrfD

//

>NGHB#3942 79

CMPLX not

b4073 nrfD putative menaquinol-cytochrome c reductasesubunit NrfD

b4074 nrfE putative cytochrome c-type biogenesis proteinNrfE

//

>NGHB#3943 -8

CMPLX not

b4074 nrfE putative cytochrome c-type biogenesis proteinNrfE

b4075 nrfF putative formate-dependent nitrite reductasecomplex subunit NrfF

//

>NGHB#3944 -4

CMPLX yes

b4075 nrfF putative formate-dependent nitrite reductasecomplex subunit NrfF

b4076 nrfG putative formate-dependent nitrite reductasecomplex subunit NrfG

//

>NGHB#3945 341

CMPLX not

b4076 nrfG putative formate-dependent nitrite reductasecomplex subunit NrfG

b4077 gltP glutamate/aspartate : H(+) symporter GltP

//

>NGHB#3947 93

CMPLX not

b4079 fdhF formate dehydrogenase H

b4078 yjcO Sel1 repeat-containing protein YjcO

//

>NGHB#3948 197

CMPLX not

b4080 mdtP putative multidrug efflux pump outer membranechannel

b4079 fdhF formate dehydrogenase H

//

>NGHB#3949 -4

CMPLX yes

b4081 mdtO putative multidrug efflux pump subunit MdtO

b4080 mdtP putative multidrug efflux pump outer membranechannel

//

>NGHB#3950 -1

CMPLX yes

b4082 mdtN putative multidrug efflux pump membrane fusionprotein

b4081 mdtO putative multidrug efflux pump subunit MdtO

//

>NGHB#3951 18

CMPLX not

b4622 ytcA putative lipoprotein YtcA

b4082 mdtN putative multidrug efflux pump membrane fusionprotein

//

>NGHB#3952 208

CMPLX not

b4083 yjcS linear primary-alkylsulfatase

b4622 ytcA putative lipoprotein YtcA

//

>NGHB#3953 272

CMPLX not

b4084 alsK D-allose kinase

b4083 yjcS linear primary-alkylsulfatase

//

>NGHB#3954 -17

CMPLX not

b4085 alsE D-allulose-6-phosphate 3-epimerase

b4084 alsK D-allose kinase

//

>NGHB#3955 10

CMPLX not

b4086 alsC D-allose ABC transporter membrane subunit

b4085 alsE D-allulose-6-phosphate 3-epimerase

//

>NGHB#3956 -22

CMPLX yes

b4087 alsA D-allose ABC transporter ATP binding subunit

b4086 alsC D-allose ABC transporter membrane subunit

//

>NGHB#3957 126

CMPLX yes

b4088 alsB D-allose ABC transporter periplasmic bindingprotein

b4087 alsA D-allose ABC transporter ATP binding subunit

//

>NGHB#3958 58

CMPLX not

b4089 alsR DNA-binding transcriptional repressor AlsR

b4088 alsB D-allose ABC transporter periplasmic bindingprotein

//

>NGHB#3960 68

CMPLX not

b4090 rpiB allose-6-phosphate isomerase/ribose-5-phosphateisomerase B

b4487 yjdP protein YjdP

//

>NGHB#3962 1

CMPLX not

b4093 phnO aminoalkylphosphonate N-acetyltransferase

b4092 phnP 5-phospho-alpha-D-ribosyl 1,2-cyclic phosphatephosphodiesterase

//

>NGHB#3963 -14

CMPLX not

b4094 phnN ribose 1,5-bisphosphate phosphokinase

b4093 phnO aminoalkylphosphonate N-acetyltransferase

//

>NGHB#3964 -1

CMPLX not

b4095 phnM RPnTP hydrolase

b4094 phnN ribose 1,5-bisphosphate phosphokinase

//

>NGHB#3965 -4

CMPLX not

b4096 phnL methylphosphonate degradation complex subunitPhnL

b4095 phnM RPnTP hydrolase

//

>NGHB#3966 110

CMPLX not

b4097 phnK carbon-phosphorus lyase subunit PhnK

b4096 phnL methylphosphonate degradation complex subunitPhnL

//

>NGHB#3967 -4

CMPLX yes

b4098 phnJ carbon-phosphorus lyase core complex subunitPhnJ

b4097 phnK carbon-phosphorus lyase subunit PhnK

//

>NGHB#3968 -8

CMPLX yes

b4099 phnI carbon-phosphorus lyase core complex subunitPhnI

b4098 phnJ carbon-phosphorus lyase core complex subunitPhnJ

//

>NGHB#3969 -1

CMPLX yes

b4100 phnH carbon-phosphorus lyase core complex subunitPhnH

b4099 phnI carbon-phosphorus lyase core complex subunitPhnI

//

>NGHB#3970 -4

CMPLX yes

b4101 phnG carbon-phosphorus lyase core complex subunitPhnG

b4100 phnH carbon-phosphorus lyase core complex subunitPhnH

//

>NGHB#3971 0

CMPLX not

b4102 phnF putative transcriptional regulator PhnF

b4101 phnG carbon-phosphorus lyase core complex subunitPhnG

//

>NGHB#3972 913

CMPLX not

b4105 phnD phosphonate/phosphate ABC transporterperiplasmic binding protein

b4102 phnF putative transcriptional regulator PhnF

//

>NGHB#3973 24

CMPLX yes

b4106 phnC phosphonate/phosphate ABC transporter ATPbinding subunit

b4105 phnD phosphonate/phosphate ABC transporterperiplasmic binding protein

//

>NGHB#3974 132

CMPLX not

b4107 yjdN PF06983 family protein YjdN

b4106 phnC phosphonate/phosphate ABC transporter ATPbinding subunit

//

>NGHB#3975 657

CMPLX not

b4108 yjdM zinc ribbon domain-containing protein YjdM

b4107 yjdN PF06983 family protein YjdN

//

>NGHB#3977 -4

CMPLX yes

b4109 rdcA regulator of diguanylate cyclase RdcA

b4110 rdcB regulator of diguanylate cyclase RdcB

//

>NGHB#3978 263

CMPLX not

b4110 rdcB regulator of diguanylate cyclase RdcB

b4111 proP osmolyte:H(+) symporter ProP

//

>NGHB#3979 111

CMPLX not

b4111 proP osmolyte:H(+) symporter ProP

b4703 pmrR putative bitopic inner membrane protein

//

>NGHB#3981 9

CMPLX not

b4113 basR DNA-binding transcriptional dual regulator BasR

b4112 basS sensor histidine kinase BasS

//

>NGHB#3982 -4

CMPLX not

b4114 eptA phosphoethanolamine transferase EptA

b4113 basR DNA-binding transcriptional dual regulator BasR

//

>NGHB#3983 103

CMPLX not

b4115 adiC arginine:agmatine antiporter

b4114 eptA phosphoethanolamine transferase EptA

//

>NGHB#3984 136

CMPLX not

b4116 adiY DNA-binding transcriptional activator AdiY

b4115 adiC arginine:agmatine antiporter

//

>NGHB#3985 324

CMPLX not

b4117 adiA arginine decarboxylase, degradative

b4116 adiY DNA-binding transcriptional activator AdiY

//

>NGHB#3986 198

CMPLX not

b4118 melR DNA-binding transcriptional dual regulator MelR

b4117 adiA arginine decarboxylase, degradative

//

>NGHB#3988 102

CMPLX not

b4119 melA alpha-galactosidase

b4120 melB melibiose:H(+)/Na(+)/Li(+) symporter

//

>NGHB#3990 121

CMPLX not

b4122 fumB fumarase B

b4121 yjdF DUF2238 domain-containing inner membrane proteinYjdF

//

>NGHB#3991 77

CMPLX not

b4123 dcuB anaerobic C4-dicarboxylate transporter DcuB

b4122 fumB fumarase B

//

>NGHB#3992 570

CMPLX not

b4124 dcuR DNA-binding transcriptional activator DcuR

b4123 dcuB anaerobic C4-dicarboxylate transporter DcuB

//

>NGHB#3993 -4

CMPLX not

b4125 dcuS sensor histidine kinase DcuS

b4124 dcuR DNA-binding transcriptional activator DcuR

//

>NGHB#3995 11

CMPLX not

b4126 yjdI PF06902 family protein YjdI

b4127 yjdJ putative N-acetyltransferase YjdJ

//

>NGHB#3996 226

CMPLX not

b4127 yjdJ putative N-acetyltransferase YjdJ

b4128 ghoS antitoxin of the GhoTS toxin-antitoxin system

//

>NGHB#3997 27

CMPLX yes

b4128 ghoS antitoxin of the GhoTS toxin-antitoxin system

b4559 ghoT toxin of the GhoTS toxin-antitoxin system

//

>NGHB#3999 236

CMPLX not

b4130 dtpC dipeptide/tripeptide:H(+) symporter DtpC

b4129 lysU lysine--tRNA ligase/Ap4A synthetase/Ap3Asynthetase

//

>NGHB#4000 58

CMPLX not

b4131 cadA lysine decarboxylase 1

b4130 dtpC dipeptide/tripeptide:H(+) symporter DtpC

//

>NGHB#4001 79

CMPLX not

b4132 cadB lysine:cadaverine antiporter

b4131 cadA lysine decarboxylase 1

//

>NGHB#4002 364

CMPLX not

b4133 cadC DNA-binding transcriptional activator CadC

b4132 cadB lysine:cadaverine antiporter

//

>NGHB#4003 798

CMPLX not

b4135 yjdC putative DNA-binding transcriptional regulatorYjdC

b4133 cadC DNA-binding transcriptional activator CadC

//

>NGHB#4004 36

CMPLX not

b4136 dsbD thiol-disulfide exchange protein DsbD

b4135 yjdC putative DNA-binding transcriptional regulatorYjdC

//

>NGHB#4005 -25

CMPLX not

b4137 cutA copper binding protein CutA

b4136 dsbD thiol-disulfide exchange protein DsbD

//

>NGHB#4006 115

CMPLX not

b4138 dcuA C4-dicarboxylate transporter DcuA

b4137 cutA copper binding protein CutA

//

>NGHB#4007 117

CMPLX not

b4139 aspA aspartate ammonia-lyase

b4138 dcuA C4-dicarboxylate transporter DcuA

//

>NGHB#4011 43

CMPLX yes

b4142 groS cochaperonin GroES

b4143 groL chaperonin GroEL

//

>NGHB#4012 137

CMPLX not

b4143 groL chaperonin GroEL

b4144 yjeI DUF4156 domain-containing lipoprotein YjeI

//

>NGHB#4014 394

CMPLX not

b4146 epmB lysine 2,3-aminomutase

b4145 yjeJ protein YjeJ

//

>NGHB#4016 51

CMPLX not

b4147 efp protein chain elongation factor EF-P

b4410 ecnA entericidin A lipoprotein, antidote toentericidin B

//

>NGHB#4017 110

CMPLX yes

b4410 ecnA entericidin A lipoprotein, antidote toentericidin B

b4411 ecnB bacteriolytic entericidin B lipoprotein

//

>NGHB#4018 175

CMPLX not

b4411 ecnB bacteriolytic entericidin B lipoprotein

b4148 gdx guanidinium exporter

//

>NGHB#4020 88

CMPLX not

b4150 ampC beta-lactamase

b4149 blc outer membrane lipoprotein Blc

//

>NGHB#4021 62

CMPLX not

b4151 frdD fumarate reductase membrane protein FrdD

b4150 ampC beta-lactamase

//

>NGHB#4022 10

CMPLX yes

b4152 frdC fumarate reductase membrane protein FrdC

b4151 frdD fumarate reductase membrane protein FrdD

//

>NGHB#4023 10

CMPLX yes

b4153 frdB fumarate reductase iron-sulfur protein

b4152 frdC fumarate reductase membrane protein FrdC

//

>NGHB#4024 -8

CMPLX yes

b4154 frdA fumarate reductase flavoprotein subunit

b4153 frdB fumarate reductase iron-sulfur protein

//

>NGHB#4026 218

CMPLX not

b4155 epmA EF-P-lysine lysyltransferase

b4156 yjeM putative transporter YjeM

//

>NGHB#4027 51

CMPLX ---

b4156 yjeM putative transporter YjeM

b4157 yjeN uncharacterized protein YjeN

//

>NGHB#4028 -4

CMPLX ---

b4157 yjeN uncharacterized protein YjeN

b4158 yjeO DUF2645 domain-containing inner membrane proteinYjeO

//

>NGHB#4030 21

CMPLX not

b4160 psd phosphatidylserine decarboxylase proenzyme

b4159 mscM miniconductance mechanosensitive channel MscM

//

>NGHB#4031 96

CMPLX not

b4161 rsgA ribosome small subunit-dependent GTPase A

b4160 psd phosphatidylserine decarboxylase proenzyme

//

>NGHB#4033 742

CMPLX ---

b4162 orn oligoribonuclease

b4670 yjeV uncharacterized protein YjeV

//

>NGHB#4036 -29

CMPLX not

b4167 nnr NAD(P)HX epimerase/NAD(P)HX dehydratase

b4168 tsaE N(6)-L-threonylcarbamoyladenine synthase, TsaEsubunit

//

>NGHB#4037 18

CMPLX not

b4168 tsaE N(6)-L-threonylcarbamoyladenine synthase, TsaEsubunit

b4169 amiB N-acetylmuramoyl-L-alanine amidase B

//

>NGHB#4038 9

CMPLX not

b4169 amiB N-acetylmuramoyl-L-alanine amidase B

b4170 mutL DNA mismatch repair protein MutL

//

>NGHB#4039 -8

CMPLX not

b4170 mutL DNA mismatch repair protein MutL

b4171 miaA tRNA dimethylallyltransferase

//

>NGHB#4040 85

CMPLX not

b4171 miaA tRNA dimethylallyltransferase

b4172 hfq RNA-binding protein Hfq

//

>NGHB#4041 75

CMPLX not

b4172 hfq RNA-binding protein Hfq

b4173 hflX ribosome rescue factor HflX

//

>NGHB#4042 85

CMPLX not

b4173 hflX ribosome rescue factor HflX

b4174 hflK regulator of FtsH protease

//

>NGHB#4043 2

CMPLX not

b4174 hflK regulator of FtsH protease

b4175 hflC regulator of FtsH protease

//

>NGHB#4044 81

CMPLX not

b4175 hflC regulator of FtsH protease

b4176 yjeT DUF2065 domain-containing protein YjeT

//

>NGHB#4045 103

CMPLX not

b4176 yjeT DUF2065 domain-containing protein YjeT

b4177 purA adenylosuccinate synthetase

//

>NGHB#4046 204

CMPLX not

b4177 purA adenylosuccinate synthetase

b4178 nsrR DNA-binding transcriptional repressor NsrR

//

>NGHB#4047 38

CMPLX not

b4178 nsrR DNA-binding transcriptional repressor NsrR

b4179 rnr RNase R

//

>NGHB#4048 179

CMPLX not

b4179 rnr RNase R

b4180 rlmB 23S rRNA 2'-O-ribose G2251 methyltransferase

//

>NGHB#4049 126

CMPLX not

b4180 rlmB 23S rRNA 2'-O-ribose G2251 methyltransferase

b4181 yjfI DUF2170 domain-containing protein YjfI

//

>NGHB#4050 18

CMPLX not

b4181 yjfI DUF2170 domain-containing protein YjfI

b4182 yjfJ PspA family protein YjfJ

//

>NGHB#4051 50

CMPLX not

b4182 yjfJ PspA family protein YjfJ

b4183 yjfK DUF2491 domain-containing protein YjfK

//

>NGHB#4052 17

CMPLX not

b4183 yjfK DUF2491 domain-containing protein YjfK

b4184 yjfL DUF350 domain-containing inner membrane proteinYjfL

//

>NGHB#4053 9

CMPLX not

b4184 yjfL DUF350 domain-containing inner membrane proteinYjfL

b4185 yjfM DUF1190 domain-containing protein YjfM

//

>NGHB#4054 2

CMPLX not

b4185 yjfM DUF1190 domain-containing protein YjfM

b4186 yjfC putative acid--amine ligase YjfC

//

>NGHB#4055 83

CMPLX not

b4186 yjfC putative acid--amine ligase YjfC

b4187 aidB putative acyl-CoA dehydrogenase AidB

//

>NGHB#4057 148

CMPLX not

b4189 bsmA DUF1471 domain-containing putative lipoproteinBsmA

b4188 yjfN protease activator YjfN

//

>NGHB#4060 107

CMPLX not

b4192 ulaG L-ascorbate-6-phosphate lactonase

b4191 ulaR DNA-binding transcriptional repressor UlaR

//

>NGHB#4062 15

CMPLX yes

b4193 ulaA L-ascorbate specific PTS enzyme IIC component

b4194 ulaB L-ascorbate specific PTS enzyme IIB component

//

>NGHB#4063 9

CMPLX yes

b4194 ulaB L-ascorbate specific PTS enzyme IIB component

b4195 ulaC L-ascorbate specific PTS enzyme IIA component

//

>NGHB#4064 13

CMPLX not

b4195 ulaC L-ascorbate specific PTS enzyme IIA component

b4196 ulaD 3-keto-L-gulonate-6-phosphate decarboxylaseUlaD

//

>NGHB#4065 9

CMPLX not

b4196 ulaD 3-keto-L-gulonate-6-phosphate decarboxylaseUlaD

b4197 ulaE L-ribulose-5-phosphate 3-epimerase UlaE

//

>NGHB#4066 -1

CMPLX not

b4197 ulaE L-ribulose-5-phosphate 3-epimerase UlaE

b4198 ulaF L-ribulose-5-phosphate 4-epimerase UlaF

//

>NGHB#4069 6

CMPLX not

b4200 rpsF 30S ribosomal subunit protein S6

b4201 priB primosomal replication protein N

//

>NGHB#4070 4

CMPLX not

b4201 priB primosomal replication protein N

b4202 rpsR 30S ribosomal subunit protein S18

//

>NGHB#4071 41

CMPLX yes

b4202 rpsR 30S ribosomal subunit protein S18

b4203 rplI 50S ribosomal subunit protein L9

//

>NGHB#4073 656

CMPLX not

b4206 ytfB cell division protein YtfB

b4204 yjfZ protein YjfZ

//

>NGHB#4075 308

CMPLX not

b4207 fklB FKBP-type peptidyl-prolyl cis-trans isomeraseFklB

b4208 cycA D-serine/alanine/glycine/:H(+)symporter

//

>NGHB#4077 107

CMPLX not

b4210 ytfF inner membrane protein YtfF

b4209 ytfE iron-sulfur cluster repair protein YtfE

//

>NGHB#4078 107

CMPLX not

b4211 qorB NAD(P)H:quinone oxidoreductase

b4210 ytfF inner membrane protein YtfF

//

>NGHB#4082 211

CMPLX not

b4214 cysQ 3'(2'),5'-bisphosphate nucleotidase

b4215 ytfI protein YtfI

//

>NGHB#4086 322

CMPLX not

b4219 msrA methionine sulfoxide reductase A

b4218 ytfL UPF0053 family inner membrane protein YtfL

//

>NGHB#4088 -4

CMPLX yes

b4220 tamA translocation and assembly module subunit TamA

b4221 tamB translocation and assembly module subunit TamB

//

>NGHB#4089 2

CMPLX not

b4221 tamB translocation and assembly module subunit TamB

b4222 ytfP gamma-glutamylamine cyclotransferase familyprotein YtfP

//

>NGHB#4090 211

CMPLX not

b4222 ytfP gamma-glutamylamine cyclotransferase familyprotein YtfP

b4224 chpS ChpS antitoxin of the ChpB-ChpS toxin-antitoxinsystem

//

>NGHB#4091 -7

CMPLX not

b4224 chpS ChpS antitoxin of the ChpB-ChpS toxin-antitoxinsystem

b4225 chpB endoribonuclease toxin ChpB

//

>NGHB#4094 139

CMPLX yes

b4227 ytfQ galactofuranose ABC transporter periplasmicbinding protein

b4485 ytfR galactofuranose ABC transporter putative ATPbinding subunit

//

>NGHB#4095 10

CMPLX yes

b4485 ytfR galactofuranose ABC transporter putative ATPbinding subunit

b4230 ytfT galactofuranose ABC transporter putativemembrane subunit YtfT

//

>NGHB#4096 -14

CMPLX yes

b4230 ytfT galactofuranose ABC transporter putativemembrane subunit YtfT

b4231 yjfF galactofuranose ABC transporter putativemembrane subunit YjtF

//

>NGHB#4102 157

CMPLX yes

b4238 nrdD anaerobic ribonucleoside-triphosphate reductase

b4237 nrdG anaerobic ribonucleoside-triphosphate reductaseactivating protein

//

>NGHB#4103 393

CMPLX not

b4239 treC trehalose-6-phosphate hydrolase

b4238 nrdD anaerobic ribonucleoside-triphosphate reductase

//

>NGHB#4104 49

CMPLX not

b4240 treB trehalose-specific PTS enzyme IIBC component

b4239 treC trehalose-6-phosphate hydrolase

//

>NGHB#4105 118

CMPLX not

b4241 treR DNA-binding transcriptional repressor TreR

b4240 treB trehalose-specific PTS enzyme IIBC component

//

>NGHB#4107 140

CMPLX not

b4702 mgtL leader peptide MgtL

b4242 mgtA Mg(2(+)) importing P-type ATPase

//

>NGHB#4109 72

CMPLX not

b4244 pyrI aspartate carbamoyltransferase, PyrI subunit

b4243 ridA enamine/imine deaminase, redox-regulatedchaperone

//

>NGHB#4110 12

CMPLX yes

b4245 pyrB aspartate carbamoyltransferase catalyticsubunit

b4244 pyrI aspartate carbamoyltransferase, PyrI subunit

//

>NGHB#4111 3

CMPLX not

b4246 pyrL pyrBI operon leader peptide

b4245 pyrB aspartate carbamoyltransferase catalyticsubunit

//

>NGHB#4112 280

CMPLX not

b4248 yjgH RutC family protein YjgH

b4246 pyrL pyrBI operon leader peptide

//

>NGHB#4113 130

CMPLX not

b4249 bdcA c-di-GMP-binding biofilm dispersal mediatorprotein

b4248 yjgH RutC family protein YjgH

//

>NGHB#4115 144

CMPLX not

b4251 bdcR putative transcriptional regulator BdcR

b4252 tabA DUF386 domain-containing toxin-antitoxin biofilmprotein TabA

//

>NGHB#4116 122

CMPLX not

b4252 tabA DUF386 domain-containing toxin-antitoxin biofilmprotein TabA

b4253 yjgL protein YjgL

//

>NGHB#4122 -1

CMPLX not

b4259 holC DNA polymerase III subunit chi

b4258 valS valine--tRNA ligase

//

>NGHB#4123 159

CMPLX not

b4260 pepA aminopeptidase A/I

b4259 holC DNA polymerase III subunit chi

//

>NGHB#4125 62

CMPLX not

b4800 ytgA protein YtgA

b4261 lptF lipopolysaccharide transport system proteinLptF

//

>NGHB#4126 -1

CMPLX yes

b4261 lptF lipopolysaccharide transport system proteinLptF

b4262 lptG lipopolysaccharide transport system proteinLptG

//

>NGHB#4128 77

CMPLX not

b4264 idnR DNA-binding transcriptional dual regulator IdnR

b4263 yjgR DUF853 domain-containing protein YjgR

//

>NGHB#4129 66

CMPLX not

b4265 idnT L-idonate/5-ketogluconate/gluconate transporter

b4264 idnR DNA-binding transcriptional dual regulator IdnR

//

>NGHB#4130 61

CMPLX not

b4266 idnO 5-keto-D-gluconate 5-reductase

b4265 idnT L-idonate/5-ketogluconate/gluconate transporter

//

>NGHB#4131 23

CMPLX not

b4267 idnD L-idonate 5-dehydrogenase

b4266 idnO 5-keto-D-gluconate 5-reductase

//

>NGHB#4135 -43

CMPLX tnp

b4272 insC6 KpLE2 phage-like element; IS2 insertion elementrepressor InsA

b4273 insD6 KpLE2 phage-like element; IS2 insertion elementprotein InsB

//

>NGHB#4136 1759

CMPLX ---

b4273 insD6 KpLE2 phage-like element; IS2 insertion elementprotein InsB

b4277 yjgZ KpLE2 phage-like element; uncharacterizedprotein YjgZ

//

>NGHB#4139 11

CMPLX not

b4279 nanX sialic acid transporter NanX

b4280 nanY KpLE2 phage-like element;2,7-anhydro-N-acetylneuraminate hydratase

//

>NGHB#4140 42

CMPLX ---

b4280 nanY KpLE2 phage-like element;2,7-anhydro-N-acetylneuraminate hydratase

b4655 ythA KpLE2 phage-like element; uncharacterizedprotein YthA

//

>NGHB#4141 623

CMPLX ---

b4655 ythA KpLE2 phage-like element; uncharacterizedprotein YthA

b4623 insO KpLE2 phage-like element; IS911B regulatorfragment

//

>NGHB#4142 0

CMPLX yes

b4288 fecD ferric citrate ABC transporter membrane subunitFecD

b4287 fecE ferric citrate ABC transporter ATP bindingsubunit

//

>NGHB#4143 -4

CMPLX yes

b4289 fecC ferric citrate ABC transporter membrane subunitFecC

b4288 fecD ferric citrate ABC transporter membrane subunitFecD

//

>NGHB#4144 -4

CMPLX yes

b4290 fecB ferric citrate ABC transporter periplasmicbinding protein

b4289 fecC ferric citrate ABC transporter membrane subunitFecC

//

>NGHB#4145 44

CMPLX yes

b4291 fecA ferric citrate outer membrane transporter

b4290 fecB ferric citrate ABC transporter periplasmicbinding protein

//

>NGHB#4146 86

CMPLX not

b4292 fecR ferric citrate regulator FecR

b4291 fecA ferric citrate outer membrane transporter

//

>NGHB#4147 -4

CMPLX not

b4293 fecI RNA polymerase sigma factor FecI

b4292 fecR ferric citrate regulator FecR

//

>NGHB#4149 86

CMPLX not

b4801 ykiE protein YkiE

b4294 insA7 KpLE2 phage-like element; IS1 repressor proteinInsA

//

>NGHB#4151 346

CMPLX not

b4296 yjhF KpLE2 phage-like element; putative transporterYjhF

b4295 yjhU KpLE2 phage-like element; putative DNA-bindingtranscriptional regulator YjhU

//

>NGHB#4152 106

CMPLX not

b4297 yjhG KpLE2 phage-like element; D-xylonatedehydratase

b4296 yjhF KpLE2 phage-like element; putative transporterYjhF

//

>NGHB#4153 10

CMPLX not

b4298 yjhH KpLE2 phage-like element; putative2-dehydro-3-deoxy-D-pentonate aldolase

b4297 yjhG KpLE2 phage-like element; D-xylonatedehydratase

//

>NGHB#4154 4

CMPLX not

b4299 yjhI KpLE2 phage-like element; putative DNA-bindingtranscriptional regulator YjhI

b4298 yjhH KpLE2 phage-like element; putative2-dehydro-3-deoxy-D-pentonate aldolase

//

>NGHB#4155 302

CMPLX not

b4300 sgcR KpLE2 phage-like element; putative DNA-bindingtranscriptional regulator SgcR

b4299 yjhI KpLE2 phage-like element; putative DNA-bindingtranscriptional regulator YjhI

//

>NGHB#4156 16

CMPLX not

b4301 sgcE KpLE2 phage-like element; putative epimeraseSgcE

b4300 sgcR KpLE2 phage-like element; putative DNA-bindingtranscriptional regulator SgcR

//

>NGHB#4157 11

CMPLX not

b4302 sgcA putative PTS enzyme IIA component SgcA

b4301 sgcE KpLE2 phage-like element; putative epimeraseSgcE

//

>NGHB#4158 130

CMPLX not

b4303 sgcQ KpLE2 phage-like element; putative nucleosidetriphosphatase

b4302 sgcA putative PTS enzyme IIA component SgcA

//

>NGHB#4159 12

CMPLX not

b4304 sgcC putative PTS enzyme IIC component SgcC

b4303 sgcQ KpLE2 phage-like element; putative nucleosidetriphosphatase

//

>NGHB#4160 11

CMPLX yes

b4565 sgcB putative PTS enzyme IIB component SgcB

b4304 sgcC putative PTS enzyme IIC component SgcC

//

>NGHB#4161 -4

CMPLX not

b4305 sgcX KpLE2 phage-like element; putative endoglucanasewith Zn-dependent exopeptidase domain

b4565 sgcB putative PTS enzyme IIB component SgcB

//

>NGHB#4162 785

CMPLX not

b4306 yjhP KpLE2 phage-like element; putativemethyltransferase YjhP

b4305 sgcX KpLE2 phage-like element; putative endoglucanasewith Zn-dependent exopeptidase domain

//

>NGHB#4163 55

CMPLX not

b4307 yjhQ KpLE2 phage-like element; putativeacetyltransferase TopAI antitoxin YjhQ

b4306 yjhP KpLE2 phage-like element; putativemethyltransferase YjhP

//

>NGHB#4164 11

CMPLX not

b4566 topAI KpLE2 phage-like element; toxin of theTopAI-YjhQ toxin-antitoxin system, TopA inhibitor

b4307 yjhQ KpLE2 phage-like element; putativeacetyltransferase TopAI antitoxin YjhQ

//

>NGHB#4167 64

CMPLX not

b4310 nanM N-acetylneuraminate mutarotase

b4309 nanS N-acetyl-9-O-acetylneuraminate esterase

//

>NGHB#4168 19

CMPLX not

b4311 nanC N-acetylneuraminic acid outer membrane channel

b4310 nanM N-acetylneuraminate mutarotase

//

>NGHB#4170 477

CMPLX not

b4312 fimB regulator for fimA

b4313 fimE regulator for fimA

//

>NGHB#4171 481

CMPLX not

b4313 fimE regulator for fimA

b4314 fimA type 1 fimbriae major subunit

//

>NGHB#4172 64

CMPLX yes

b4314 fimA type 1 fimbriae major subunit

b4315 fimI putative fimbrial protein FimI

//

>NGHB#4173 36

CMPLX not

b4315 fimI putative fimbrial protein FimI

b4316 fimC type 1 fimbriae periplasmic chaperone

//

>NGHB#4174 66

CMPLX not

b4316 fimC type 1 fimbriae periplasmic chaperone

b4317 fimD type I fimbriae usher protein

//

>NGHB#4175 9

CMPLX not

b4317 fimD type I fimbriae usher protein

b4318 fimF type 1 fimbriae minor subunit FimF

//

>NGHB#4176 12

CMPLX yes

b4318 fimF type 1 fimbriae minor subunit FimF

b4319 fimG type 1 fimbriae minor subunit FimG

//

>NGHB#4177 19

CMPLX not

b4319 fimG type 1 fimbriae minor subunit FimG

b4320 fimH type 1 fimbriae D-mannose specific adhesin

//

>NGHB#4180 80

CMPLX not

b4322 uxuA D-mannonate dehydratase

b4323 uxuB D-mannonate oxidoreductase

//

>NGHB#4181 214

CMPLX not

b4323 uxuB D-mannonate oxidoreductase

b4324 uxuR DNA-binding transcriptional repressor UxuR

//

>NGHB#4184 -22

CMPLX not

b4720 ytiC protein YtiC

b4721 ytiD protein YtiD

//

>NGHB#4185 115

CMPLX not

b4721 ytiD protein YtiD

b4722 idlP iraD leader peptide

//

>NGHB#4186 -4

CMPLX not

b4722 idlP iraD leader peptide

b4326 iraD anti-adaptor protein IraD, inhibitor of sigma(S)proteolysis

//

>NGHB#4188 64

CMPLX not

b4328 iadA isoaspartyl dipeptidase

b4327 hypT DNA-binding transcriptional dual regulator HypT

//

>NGHB#4189 12

CMPLX not

b4329 yjiG Gate family protein YjiG

b4328 iadA isoaspartyl dipeptidase

//

>NGHB#4190 -4

CMPLX not

b4330 yjiH Gate family protein YjiH

b4329 yjiG Gate family protein YjiG

//

>NGHB#4193 67

CMPLX ---

b4333 yjiK uncharacterized protein YjiK

b4332 yjiJ putative transporter YjiJ

//

>NGHB#4194 64

CMPLX ---

b4715 ytiA uncharacterized protein YtiA

b4333 yjiK uncharacterized protein YjiK

//

>NGHB#4195 -4

CMPLX ---

b4334 yjiL putative ATPase, activator of(R)-hydroxyglutaryl-CoA dehdratase

b4715 ytiA uncharacterized protein YtiA

//

>NGHB#4196 9

CMPLX not

b4335 yjiM putative dehydratase subunit

b4334 yjiL putative ATPase, activator of(R)-hydroxyglutaryl-CoA dehdratase

//

>NGHB#4197 115

CMPLX not

b4336 yjiN DUF445 domain-containing protein YjiN

b4335 yjiM putative dehydratase subunit

//

>NGHB#4198 40

CMPLX not

b4337 mdtM multidrug efflux pump/bile salt:H(+)antiporter/Na(+):H(+) antiporter/K(+):H(+) antiporter

b4336 yjiN DUF445 domain-containing protein YjiN

//

>NGHB#4199 1642

CMPLX not

b4340 yjiR fused putative DNA-binding transcriptionalregulator/putative aminotransferase YjiR

b4337 mdtM multidrug efflux pump/bile salt:H(+)antiporter/Na(+):H(+) antiporter/K(+):H(+) antiporter

//

>NGHB#4201 498

CMPLX ---

b4341 yjiS DUF1127 domain-containing protein YjiS

b4342 yjiT putative uncharacterized protein YjiT

//

>NGHB#4203 -1

CMPLX not

b4346 mcrB McrBS

b4345 mcrC 5-methylcytosine-specific restriction enzymesubunit McrC

//

>NGHB#4204 644

CMPLX not

b4347 symE toxic protein SymE

b4346 mcrB McrBS

//

>NGHB#4205 227

CMPLX not

b4348 hsdS type I restriction enzyme EcoKI specificityprotein

b4347 symE toxic protein SymE

//

>NGHB#4206 -4

CMPLX yes

b4349 hsdM type I restriction enzyme EcoKImethyltransferase component

b4348 hsdS type I restriction enzyme EcoKI specificityprotein

//

>NGHB#4207 200

CMPLX yes

b4350 hsdR type I restriction enzyme EcoKI endonucleasecomponent

b4349 hsdM type I restriction enzyme EcoKImethyltransferase component

//

>NGHB#4210 10

CMPLX not

b4353 yjiX PF04328 family protein YjiX

b4352 yjiA P-loop guanosine triphosphatase YjiA

//

>NGHB#4211 49

CMPLX not

b4354 btsT pyruvate:H(+) symporter

b4353 yjiX PF04328 family protein YjiX

//

>NGHB#4214 214

CMPLX not

b4357 lgoR putative DNA-binding transcriptional regulatorLgoR

b4356 lgoT galactonate:H(+) symporter

//

>NGHB#4217 253

CMPLX not

b4360 yjjA DUF2501 domain-containing protein YjjA

b4359 opgB phosphoglycerol transferase I

//

>NGHB#4218 48

CMPLX not

b4361 dnaC DNA replication protein DnaC

b4360 yjjA DUF2501 domain-containing protein YjjA

//

>NGHB#4219 2

CMPLX not

b4362 dnaT primosomal protein DnaT

b4361 dnaC DNA replication protein DnaC

//

>NGHB#4220 106

CMPLX not

b4363 yjjB putative succinate exporter YjjB

b4362 dnaT primosomal protein DnaT

//

>NGHB#4221 -10

CMPLX yes

b4364 yjjP putative succinate exporter YjjP

b4363 yjjB putative succinate exporter YjjB

//

>NGHB#4223 -43

CMPLX not

b4365 yjjQ DNA-binding transcriptional repressor YjjQ

b4366 bglJ DNA-binding transcriptional regulator BglJ

//

>NGHB#4228 -32

CMPLX not

b4372 holD DNA polymerase III subunit psi

b4373 rimI protein N-acetyltransferase RimI

//

>NGHB#4229 14

CMPLX not

b4373 rimI protein N-acetyltransferase RimI

b4374 yjjG pyrimidine 5'-nucleotidase YjjG

//

>NGHB#4230 90

CMPLX not

b4374 yjjG pyrimidine 5'-nucleotidase YjjG

b4375 prfC peptide chain release factor RF3

//

>NGHB#4231 392

CMPLX not

b4375 prfC peptide chain release factor RF3

b4376 osmY periplasmic chaperone OsmY

//

>NGHB#4232 126

CMPLX not

b4376 osmY periplasmic chaperone OsmY

b4568 ytjA DUF1328 domain-containing protein YtjA

//

>NGHB#4233 121

CMPLX not

b4568 ytjA DUF1328 domain-containing protein YtjA

b4377 yjjU putative patatin-like phospholipase YjjU

//

>NGHB#4234 -4

CMPLX not

b4377 yjjU putative patatin-like phospholipase YjjU

b4378 yjjV putative DNase YjjV

//

>NGHB#4236 -29

CMPLX not

b4380 yjjI DUF3029 domain-containing protein YjjI

b4379 yjjW putative glycyl-radical enzyme activating enzymeYjjW

//

>NGHB#4238 126

CMPLX not

b4381 deoC deoxyribose-phosphate aldolase

b4382 deoA thymidine phosphorylase

//

>NGHB#4239 51

CMPLX not

b4382 deoA thymidine phosphorylase

b4383 deoB phosphopentomutase

//

>NGHB#4240 56

CMPLX not

b4383 deoB phosphopentomutase

b4384 deoD purine nucleoside phosphorylase

//

>NGHB#4241 166

CMPLX not

b4384 deoD purine nucleoside phosphorylase

b4385 yjjJ toxin YjjJ

//

>NGHB#4243 27

CMPLX not

b4387 ytjB protein Smp

b4386 lplA lipoate--protein ligase A

//

>NGHB#4245 48

CMPLX not

b4388 serB phosphoserine phosphatase

b4389 radA DNA recombination protein

//

>NGHB#4246 20

CMPLX not

b4389 radA DNA recombination protein

b4390 nadR DNA-binding transcriptional repressor/NMNadenylyltransferase NadR

//

>NGHB#4249 89

CMPLX not

b4392 slt soluble lytic murein transglycosylase

b4393 trpR DNA-binding transcriptional repressor TrpR

//

>NGHB#4254 12

CMPLX not

b4397 creA PF05981 family protein CreA

b4398 creB DNA-binding transcriptional regulator CreB

//

>NGHB#4255 -1

CMPLX not

b4398 creB DNA-binding transcriptional regulator CreB

b4399 creC sensory histidine kinase CreC

//

>NGHB#4256 57

CMPLX not

b4399 creC sensory histidine kinase CreC

b4400 creD putative inner membrane protein CreD

//

>NGHB#4259 399

CMPLX ---

b4402 yjjY uncharacterized protein YjjY

b4403 yjtD putative rRNA methyltransferase

//
