# Supplementary material for: Unidirectional gene pairs in archaea and bacteria require overlaps or very short intergenic distances for translational coupling via termination-reinitiation and often encode subunits of heteromeric complexes
Source: Front Microbiol. 2023 Nov 9;14:1291523. doi: 10.3389/fmicb.2023.1291523 (PMC10666635; doi:10.3389/fmicb.2023.1291523)
Supplement: Supplementary file 7 [file Table_6.DOCX]

**Supplementary Table S6.** List of oligonucleotides used for cloning, mutagenesis, sequencing, and generation of probes for Northern blots.

1.1 Primer List for Northern-Blot Probes

| Name | Sequence (5-3) |
| --- | --- |
| AraDH fw | CGGGAGAGTCACGGTCGAAG |
| AraDH rev | GTCGCCGAGTTGTCTCGC |
| Dhfr fw | TCGTCTCTGTCGCCGCGCTC |
| Dhfr rev | AACTATCTGGACGACGCCGACCG |
| GusA_fw | GGGTGGACGATATCACCGTGGTGACG |
| GusA_rev | CAATCACCACGATGCCATGTTCATCTGC |
| GlpD_fw | CGCGGTTTATCCGTGCTGATGCTGGAGG |
| GlpD_rv | GCCGGTATCGATATCTTCCGCTTCCACAATCCAC |

1.2 Primer List for construction of pCW1 (pBJ2 (Huber 2019) derived double reporter plasmid with AraDH/DHFR Reporter genes)

| Name | Sequence (5-3) |
| --- | --- |
| HVO_B0032_PstI_for | GATCCTGCAGATGGCACGTATTGCGGTCAC |
| HVO_B0032_NcoI_rev | GATCCCATGGCTCCACGACGGTCGCCGAGTTG |
| HVO_B0032_KpnI_rev | GATCGGTACCTCACTCCACGACGGTCGCCGAGTT |
| HVO_2431_NcoI_NdeI_for | GATCCCATGGGATTGGGGAGGTGACCGCATATGACGCTCGTCTC |
| HVO_B0032_Linker_NcoI_rev | GATCCCATGGGGATCCGCCGCCACCCGACCCACCACCGCCCGAGCCACCGCCACCCTCCACGACGGTCGCCGAGTTG |
| DHFR_KpnI_rev | GCGAATTGGGTACCAAAAAAAAAAACTATCTGGACG |
| P1_HVO_B0032_for | ACATGCTCCCGAGGAAGACGAG |
| P2_HVO_B0032_rev | GAATCGCTCGCTGACGAGATTC |
| P3_HVO_B0032_for | TCGGCGACCGTCGTGGAGTGAG |
| P4_HVO_B0032_rev | TCACGTTCGCGCTCGCACCGAC |

1.3 Primer List for mutagenesis of premature stop-codons in *H. volcanii*

| Name | Sequence (5-3) | |
| --- | --- | --- |
| HVO_0685-TGA+5_for | GCCGACGGGAGGTGAGTGAGATG | |
| HVO_0685-TGA+5_rev | CATCTCACTCACCTCCCGTCGGC | |
| HVO_0685-TGA+13_for | TGAGGAGGTGAGTGAGATGTCGATGTC | |
| HVO_0685-TGA+13_rev | CCTCCTCAGGCCGCGGC | |
| HVO_0685-TGA+19_for | TGAGCCGACGGAGGTGAGTGAGATG | |
| HVO_0685-TGA+19_rev | TCCGTCGGCTCAGGCGGTG | |
| HVO_0685-TGA+22_for | TGAGCGGCCGACGGAGGTGAG | |
| HVO_0685-TGA+22_rev | GCCGCTCAGGTGTCGCGG | |
| HVO_0685-TGA+34_for | TGACGCGACACCGCCGCG | |
| HVO_0685-TGA+34_rev | TCGCGTCAGAACACGGGCG | |
| 2431_TGA+14_fw | CGGATTGAGGAGGTGACCGCCG | |
| 2431_TGA+14_rev | TCAATCCGAGCGCTCCTC | |
| 2431_TGA+23_fw | GGAGTGATCGGATTGGGGAGGTG | |
| 2431_TGA+23_rev | TCACTCCTCCAAGTAGGTCACGAG | |
| 2555_TGA+14_fw. | GAGATTTGAGGAGGTGACGAGTGATGG | |
| 2555_TGA+14_rev. | TCAAATCTCGACGACGACGTG | |
| 2555_TGA+23_fw. | CGTCTGAGAGATTATGGGAGGTGACGAG |  |
| 2555_TGA+23_rev. | TCAGACGACGTGAGATTTCGTCTTC |  |

1.4 Primer List for mutagenesis of premature stop-codons in *E.Coli*

| Name | Sequence (5-3) |
| --- | --- |
| ydbH/ynbE TGA2 fw | GAGGAATGACAATGAAAATTTTACTGG |
| ydbH/ynbE TGA2 rev | TCATTCCTCACACTCTTTTCCTT |
| ydbH/ynbE TGA14 fw | GGAAAATGATGTGAGGAAAAAC |
| ydbH/ynbE TGA14 rev | TCATTTTCCTTGCGGACAG |
| ydbH/ynbE TGA38 fw | CTGCCGTGAAATGACTGTCCGCAAG |
| ydbH/ynbE TGA38 rev | TCACGGCAGACGTGCGTTCTGCT |
| hyfH/hyfI TGA2 fw | AGGAGTGACTATGAGTCCAGTGC |
| hyfH/hyfI TGA2 rev | CATAGTCACTCCTTAGCCACCAGC |
| hyfH/hyfI TGA14 fw | TGCTGTGAGCTAAGGAGCAGCTATG |
| hyfH/hyfI TGA14 rev | CTTAGCTCACAGCAGTACATCTGTATC |
| hyfH/hyfI TGA38 fw | TGATCTGAGACGATACAGATGTACTGCTG |
| hyfH/hyfI TGA38 rev | GTCTCAGATCAGCGTCGCGCGTTG |
| menD/menH TGA2 Fw | TGATTATGATCCTGCACGCGCAGG |
| menD/menH TGA2 rev | CATAATCAGCTTACCTGCGCCAG |
| menD/menH TGA14 fw | TGACAGGTAAGCCATTTATGA |
| menD/menH TGA14 rev | CCTGTCACAGAAGTTGCTGGAG |
| menD/menH TGA38 fw | ATGGTTGACAAACGCTCCAGCAAC |
| menD/menH TGA38 rev | TCAACCATCGGTGTCGTTAACCAC |

1.5 Primer List for contsruction of truncated gene versions in *E.Coli*

| Name | Sequence (5-3) |
| --- | --- |
| PmenH_XhoI | CAGGCAAAACACGGAC |
| PmenH_rev | TCGAGTCCGTGTTTTGCCTGCGCGTGCAGGATCAT |
| Pmen_DH_NcoI | CATGGCAGGTAAGCCATTTATGATCCTGCACGCG |
| Pmen_DH | CAGGTAAGCCATTTATGATCCTGCACGCG |
| Pmen_DH15_NcoI_rev | AAATGGCTTACCTGC |
| Pmen_DH36_NcoI_rev | AAATGGCTTACCTGCGCCAGAAGTTGCTGGAGCGTC |
| Pmen_DH36_NcoI_fw | CATGGACGCTCCAGCAACTTCTGGCG |
| Pmen_DH57_NcoI_rev | AAATGGCTTACCTGCGCCAGAAGTTGCTGGAGCGTTTGCGCACCATC  GGTGTCGTTC |
| Pmen_DH57_NcoI_fw | CATGGAACGACACCGATGGTGCGCAAACGCTCCAGCAACTTCTGGCG |
| Pmen_DH84_NcoI_rev | AAATGGCTTACCTGCGCCAGAAGTTGCTGGAGCGTTTGCGCACCATCG  GTGTCGTTAACCACCATTTCAATCACCGTGGTGGTC |
| Pmen_DH84_NcoI_fw | CATGGACCACCACGGTGATTGAAATGGTGGTTAACGACACCGATGGTG  CGCAAACGCTCCAGCAACTTCTGGCG |
| PhyfI_XhoI | ACACAACATGTCAGCC |
| PhyfI_rev | TCGAGGCTGACATGTTGTGTAAGCACTGGACTCAT |
| Phyf_HI_NcoI | CATGGGCTAAGGAGCAGCTATGAGTCCAGTGCTT |
| Phyf_HI | GCTAAGGAGCAGCTATGAGTCCAGTGCTT |
| Phyf_HI_15_rev_NcoI | AGCTGCTCCTTAGCC |
| Phyf_HI_33_rev_NcoI | AGCTGCTCCTTAGCCACCAGCAGTACATCTGTC |
| Phyf_HI_33_fw_NcoI | CATGGACAGATGTACTGCTGGTG |
| Phyf_HI_45_rev_NcoI | AGCTGCTCCTTAGCCACCAGCAGTACATCTGTATCGTCGTTGATC |
| Phyf_HI_45_fw_NcoI | CATGGATCAACGACGATACAGATGTACTGCTGGTG |
| Phyf_HI_81_rev_NcoI | AGCTGCTCCTTAGCCACCAGCAGTACATCTGTATCGTCGTTGATCAGCGTCG  CGCGTTGTTTGCATTCCGGGCAGACGCTC |
| Phyf_HI_81_fw_NcoI | CATGGAGCGTCTGCCCGGAATGCAAACAACGCGCGACGCTGATCAACGACG  ATACAGATGTACTGCTGGTG |
